# MS-DAP Platform for Downstream Data Analysis of Label-Free Proteomics Uncovers Optimal Workflows in Benchmark Data Sets and Increased Sensitivity in Analysis of Alzheimer’s Biomarker Data

**DOI:** 10.1021/acs.jproteome.2c00513

**Published:** 2022-12-21

**Authors:** Frank Koopmans, Ka Wan Li, Remco V. Klaassen, August B. Smit

**Affiliations:** † Department of Molecular and Cellular Neurobiology, Center for Neurogenomics and Cognitive Research, Amsterdam Neuroscience, VU University, 1081 HV Amsterdam, The Netherlands

**Keywords:** proteomics, bioinformatics, software, benchmarking, Alzheimer’s disease

## Abstract

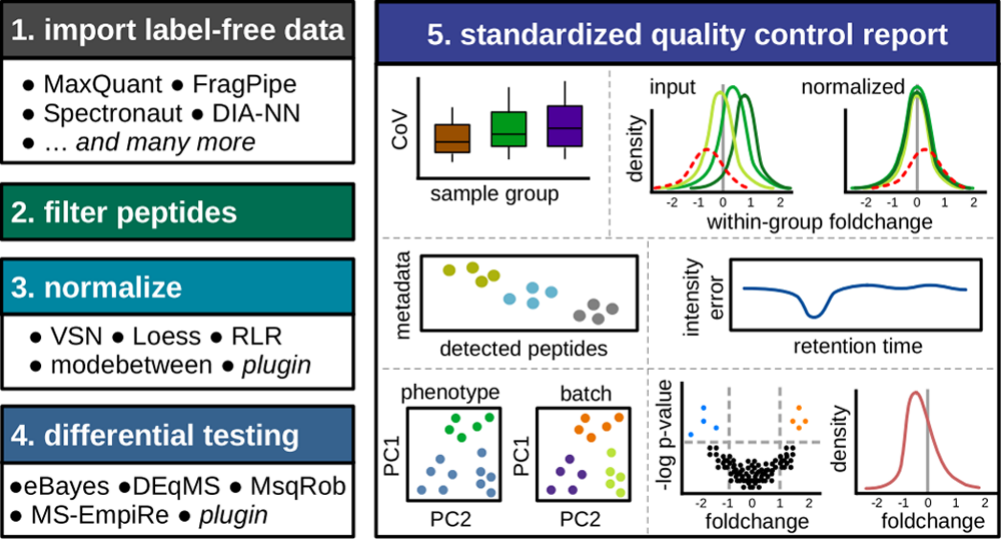

In the rapidly moving proteomics field, a diverse patchwork of data analysis pipelines and algorithms for data normalization and differential expression analysis is used by the community. We generated a mass spectrometry downstream analysis pipeline (MS-DAP) that integrates both popular and recently developed algorithms for normalization and statistical analyses. Additional algorithms can be easily added in the future as plugins. MS-DAP is open-source and facilitates transparent and reproducible proteome science by generating extensive data visualizations and quality reporting, provided as standardized PDF reports. Second, we performed a systematic evaluation of methods for normalization and statistical analysis on a large variety of data sets, including additional data generated in this study, which revealed key differences. Commonly used approaches for differential testing based on moderated t-statistics were consistently outperformed by more recent statistical models, all integrated in MS-DAP. Third, we introduced a novel normalization algorithm that rescues deficiencies observed in commonly used normalization methods. Finally, we used the MS-DAP platform to reanalyze a recently published large-scale proteomics data set of CSF from AD patients. This revealed increased sensitivity, resulting in additional significant target proteins which improved overlap with results reported in related studies and includes a large set of new potential AD biomarkers in addition to previously reported.

## Introduction

Mass spectrometry-based label-free proteomics has seen a rapid evolution in various fields of science and is now widely used as a high-throughput approach for the quantitative characterization of proteomes in biology and clinical research.^1−3^ Both instruments and raw data interpretation software have seen systematic improvements over the years.^4−8^ Common approaches to label-free proteomics are Data Dependent Analysis (DDA) and Data Independent Analysis (DIA). The former aims to both identify and quantify peptides whereas the latter aims to optimize sensitivity and reproducibility for quantitative studies through customized data acquisition strategies and posthoc data mining.^9,10^ Recent innovations have greatly improved identification of peptides directly from DIA data^7,11^ and using hybrid spectral libraries from both DDA and DIA data.^12^ Output data from the mass spectrometer (“raw data”) is first processed into a set of peptides and proteins, each with a confidence score and quantitative value per analyzed sample. FragPipe,^13^ MaxQuant,^14^ Skyline,^15^ ProteomeDiscoverer, MetaMorpheus,^16^ Spectronaut^17^ and DIA-NN^7^ are some of the commonly used tools. The next step is downstream analysis, which typically consists of three steps; removing low quality peptides and/or proteins (e.g., low confidence score, or identified sporadically), data normalization and statistical analyses to identify differentially expressed proteins.

Normalization algorithms aim to reduce variation in abundance levels between (biological) replicates, for instance, caused by sample loading differences, while maintaining differences in expression between conditions. Accurately scaling abundance values between groups is especially challenging in data sets with asymmetric protein foldchanges. In proteomics and gene expression studies alike, there is a mean-variance relationship and some normalization approaches take this into account, such as Variance Stabilizing Normalization (VSN),^18^ while other approaches scale all abundance values per sample by a constant factor such as the foldchange-based normalization algorithm included in the MS-EmpiRe R package.^19^ Normalization has a major impact on the outcome of statistical analyses for both proteomics^20^ and RNA-seq data,^21^ so it is imperative that researchers select an algorithm (from the many available) that is appropriate for the data set at hand. To this end, comparative data analyses of such algorithms using representative benchmarking data sets can reveal which algorithms generally outperform and are a good starting point when analyzing real-world data sets.

Recent innovations in statistical models for label-free proteomics data empower quantitative proteomics with increased sensitivity. The DEqMS^22^ model extends the common strategy of moderated t-statistics applied to protein abundances, popular implementations include limma eBayes^23^ and Perseus,^24^ by incorporating the number of quantified peptides per protein into the DEqMS model. Statistical models such as MSqRob^25^ and MS-EmpiRe^19^ operate directly on peptide abundance values to estimate differential expression likelihood at the protein-level and do not require peptide to protein rollup. By estimating the confidence level of peptides these models can find the consensus effect-size of each protein with more tolerance to outlier data points, as compared to models that require a priori protein rollup (e.g., using the MaxLFQ algorithm^26^). MsqRobSum^27^ is a hybrid approach that uses the MSqRob peptide-level regression model to summarize peptide-level data to the protein-level, after which statistics can be applied to protein abundance values.

There are many tools that facilitate these downstream analysis steps, some are provided as graphical user interfaces, e.g., Perseus,^24^ ProtExA^28^ and LFQ-Analyst,^29^ or as R packages such as MSstats,^30^ DEP,^31^ NormalyzerDE^32^ and protti.^33^ However, many of these existing pipelines implement few algorithms for normalization and statistical analyses without the ability for the user community to apply additional algorithms (e.g., methods not supported when the pipeline was built/published, or novel methods published in years after). Moreover, some are specific to work only with input data generated by 1 raw data processing software such as MaxQuant. For instance, most pipelines described previously support differential expression analysis (DEA) using moderated t-statistics (e.g., limma eBayes or analogous implementations) but do not support algorithms that improve on this, such as MSqRob,^25^ MS-EmpiRe^19^ or DEqMS.^22^ The recently published StatsPro^34^ implements multiple protein-level DEA algorithms, including aforementioned MSqRobSum, limma eBayes and DEqMS, but not MS-EmpiRe and MSqRob. Building in-house scripts to accommodate a combination of normalization and statistical model of choice is laborious and requires in-depth bioinformatics expertise to ensure construction of high-quality data workflows. This hampers the adoption of state-of-the-art algorithms by the wider proteomics community.

Therefore, we generated a downstream analysis pipeline for label-free proteomics data sets, named Mass Spectrometry Downstream Analysis Pipeline (MS-DAP), that encompasses different stages of quantitative data interpretation. Current state-of-the-art algorithms for normalization and differential testing are integrated, and future algorithmic innovations can be easily plugged in. The pipeline produces standardized PDF reports with extensive data visualizations, quality reporting that is automatically generated from user-provided experiment metadata (e.g., sample measurement order, experiment batches, cohorts, etc.) and includes documentation for each analysis.

We used MS-DAP to perform systematic evaluation of all embedded algorithms using a wide variety of benchmark data sets (DDA, DIA, in-silico, various instruments, 22 statistical contrasts in total). An additional spike-in data set was created using a state-of-the-art mass spectrometer to extend our benchmark analyses and support investigation of false positive rates. Evaluation of several different types of label-free proteomics data sets revealed that normalization algorithms had a major impact om subsequent statistical analyses with large variability in their performance across data sets. We introduced a novel normalization algorithm that can be used in conjunction with other normalization algorithms to rebalance protein foldchanges between experimental conditions, which improved ROC performance for all evaluated normalization algorithms in all data sets (median improvement in partial AUC over data sets of respective algorithms was 4–20%) and strongly reduced between-data set performance (standard deviation over data sets decreased from 0.11–0.20 of respective algorithms to 0.02–0.03). The recently introduced DEqMS, MSqRob and MS-EmpiRe improved over the commonly used moderated *t* test (limma eBayes) in 19 of the 22 statistical contrasts. In our generated benchmark data set, particularly challenging due to low sample loading, short gradients and minor spike-in foldchanges of 20 and 25%, these modern DEA algorithms uncovered hundreds of significant hits whereas the former recovered only a fraction thereof or none at all. Elaborate analyses of DEA algorithm results presented here provide insights for future algorithmic developments and generation of benchmark data sets representative of real-world data.

Finally, MS-DAP was used to reanalyze a large-scale Alzheimer’s disease (AD) biofluid proteomics data set to demonstrate gain in sensitivity, leading to potential new biomarkers, and ease of use. Cohort-specific regulations were readily identified from data visualizations in the MS-DAP report and incorporated in statistical analyses, resulting in 159 unique significant hits at 0.1% FDR as compared to 43 proteins reported in the original study. These included the vast majority of originally reported proteins, many protein family members thereof as well as strong overlap with results from previously reported AD biofluid studies. Literature search uncovered AD association for 25 of the MS-DAP exclusive hits. Taken together, application of MS-DAP yielded additional highly significant proteins that both corroborated AD associated proteins and yielded potentially new biomarkers.

## Materials and Methods

### Variation Within, Mode Between

The Variation Within (VW) and Mode Between (MB) normalization algorithm consists of two consecutive steps; first samples are scaled within each group to minimize variation among replicates and then scaled at sample-group-level such that the mode of between-group log-foldchanges is zero. Input data are a numerical input matrix where columns represent samples and rows represent features (e.g., peptides or proteins) together with sample group assignments for each column in this matrix.

VW: within the subset of matrix columns that match sample group *g*, each column is scaled such that the median of variation estimates for all rows is minimized.

MB: the foldchange between a pair of sample groups for each feature is computed using the respective mean value over samples within each group. The mode thereof is computed for all pairs of sample groups. The sum of the absolute values (FC modes) is minimized in the between-group normalization step.

Mode Within and Mode Between (MWMB) is a variant of the VWMB implementation where within-group samples are normalized by their pairwise log-foldchange modes, the between-group part of the algorithm is the same as with VWMB.

These algorithms are included in the open-source MS-DAP R package and available on the MS-DAP GitHub repository. The implementation of specifically this algorithm is available at: https://github.com/ftwkoopmans/msdap/blob/master/R/normalize_vwmb.R.

To perform protein-level normalization for a peptide abundance matrix, available in MS-DAP as normalization parameter “modebetween_protein” (referred to as MBprot in this manuscript), first a roll up from peptide- to protein-level is performed and then between-group scaling levels with the “normalize_vwmb” normalization function are computed (disabling within-group scaling).

For reference, we also implemented a naïve approach that minimizes variation of all peptides over the entire data set (denoted as “var_overall”), which is effectively the VW algorithm under the assumption that all samples belong to one sample group.

### Benchmarking Data Sets

The LFQbench2016^35^ data was downloaded from the PRIDE^36^ repository (identifier PXD002952). The “5600” data set refers to data acquired on a SCIEX TripleTOF 5600 configured for SWATH-MS with 64 variable windows. Respective raw files were “HYE124_TTOF5600_64var_lgillet_L150206_<ID>” with IDs 007–012. Analogously, the “6600” data set refers to data acquired on a SCIEX TripleTOF 6600 configured for SWATH-MS with 64 variable windows. Respective raw files were “HYE124_TTOF6600_64var_lgillet_I150211_<ID>” with IDs 008–013. The LFQbench2022^37^ data was downloaded from the PRIDE repository (identifier PXD028735). The O’Connell et al. data^38^ was downloaded from PRIDE repository PXD007683 (label-free quantification raw data files only). The Shen et al. data^39^ was downloaded from the PRIDE repository (identifier PXD003881).

Proteomes in FASTA format (including canonical and additional isoforms, Swiss-Prot and TrEMBL) were downloaded from UniProt (release 2022-02). Raw data for all DDA data sets were reanalyzed using MaxQuant 2.1.1.0 with match-between-runs enabled. Raw data for all DIA data sets were reanalyzed using DIA-NN 1.8, using in-silico predicted spectral libraries.

The in-silico data sets benchmarked in this study are based on the Lim et al. data set^40^ available at PRIDE repository PXD014415. Samples that only contain human cells were reanalyzed with MaxQuant, then processed as follows: (1) import the data set into MS-DAP 1.0.3, (2) split 16 of the samples that only contain human cells randomly into groups A and B, (3) select 10% of the proteins at random and increase their respective peptide abundances in group B by 1.2-fold. The result is an artificial data set with 8 replicate samples and *a priori* known differentially expressed proteins (with real-world variation and potentially some difficult to detect true positives due to random selection of noisy proteins/peptides). Additionally, we created subsets of this data set with 6 or 4 replicates, respectively.

MS-DAP version 1.0.3 was used for all benchmarking analyses. For both LFQ bench data sets, which are 3-proteome mixtures (Human, Yeast, *E. coli*), we included the *E. coli* proteome in all data analysis steps but disregarded these for ROC analyses because the spike-in ratio was very high (4-fold) as compared to Human∼Yeast (2-fold, which should already be a relatively easy test).

### LC-MS

A two-proteome spike-in series was created using 50 ng HeLa per sample and adding 12.5 ng, 15.625 or 18.75 Yeast (depending on experimental condition). Each sample of tryptic digest was redissolved in 100 μL of 0.1% formic acid; the peptide solution was transferred to an Evotip, and run on a 15 cm × 75 μm, 1.9 μm Performance Column (EV1112 from EvoSep) using an Evosep One liquid chromatography system with the 30 samples per day program. Peptides were electro-sprayed into the timsTOF Pro 2 mass spectrometer and analyzed with parallel accumulation–serial fragmentation combined with diaPASEF. The MS scan was between 100 and 1700 *m*/*z*. The tims settings were 1/Ko from start to end between 0.6 and 1.6 V·s/cm^2^, ramp time 100 ms, accumulate time 100 ms and ramp rate 9.42 Hz. The same set of samples was also analyzed in ddaPASEF mode. The mass spectrometry proteomics data have been deposited to the ProteomeXchange Consortium via the PRIDE partner repository^36^ with the data set identifier PXD036134.

### The Bader et al. AD-CSF biomarker data set

The Bader et al. data set^41^ was acquired from the PRIDE repository (identifier PXD016278). The Spectronaut^17^ result file in .sne format was downloaded (“main study_three cohorts_Spectronaut session.zip”), imported into Spectronaut and exported as a plain text report compatible with MS-DAP 1.0.3. The sample metadata table was downloaded (“annotation of samples_AM1.5.11.zip”) and some of the columns were renamed for compatibility with MS-DAP (filenames as “sample_id” and sample classifications as “group”).

The decision rules for “exclude” samples, which are included in all MS-DAP data visualizations and quality control analyses but excluded from differential expression analysis, were as follows: (1) lacking either clinical AD diagnosis or biochemical AD classification (flagged as exclude in original study), (2) inconsistency between clinical AD diagnosis and biochemical AD classification, (3) technical replicates, (4) samples that were clear outliers compared to respective within-group biological replicates in the MS-DAP QC report.

MS-DAP 1.0.3 was applied with feature selection such that each peptide is detected (Spectronaut Qvalue ≤0.01) in at least 8 samples in each sample group (*filter_min_detect = 8*), by-contrast filtering enabled (*filter_by_contrast = TRUE*), normalization set to a combination of VSN and protein-level mode-between (*norm_algorithm = c(“vsn”, “modebetween_protein”)*), MSqRob as DEA algorithm (*dea_algorithm = “msqrob”*) and remaining settings at default. The full MS-DAP report file contains all data visualizations, documentation thereof and the R code used to run the pipeline (Supporting Data 1).

## Results

### Overview of MS-DAP Features

**Figure 1:**
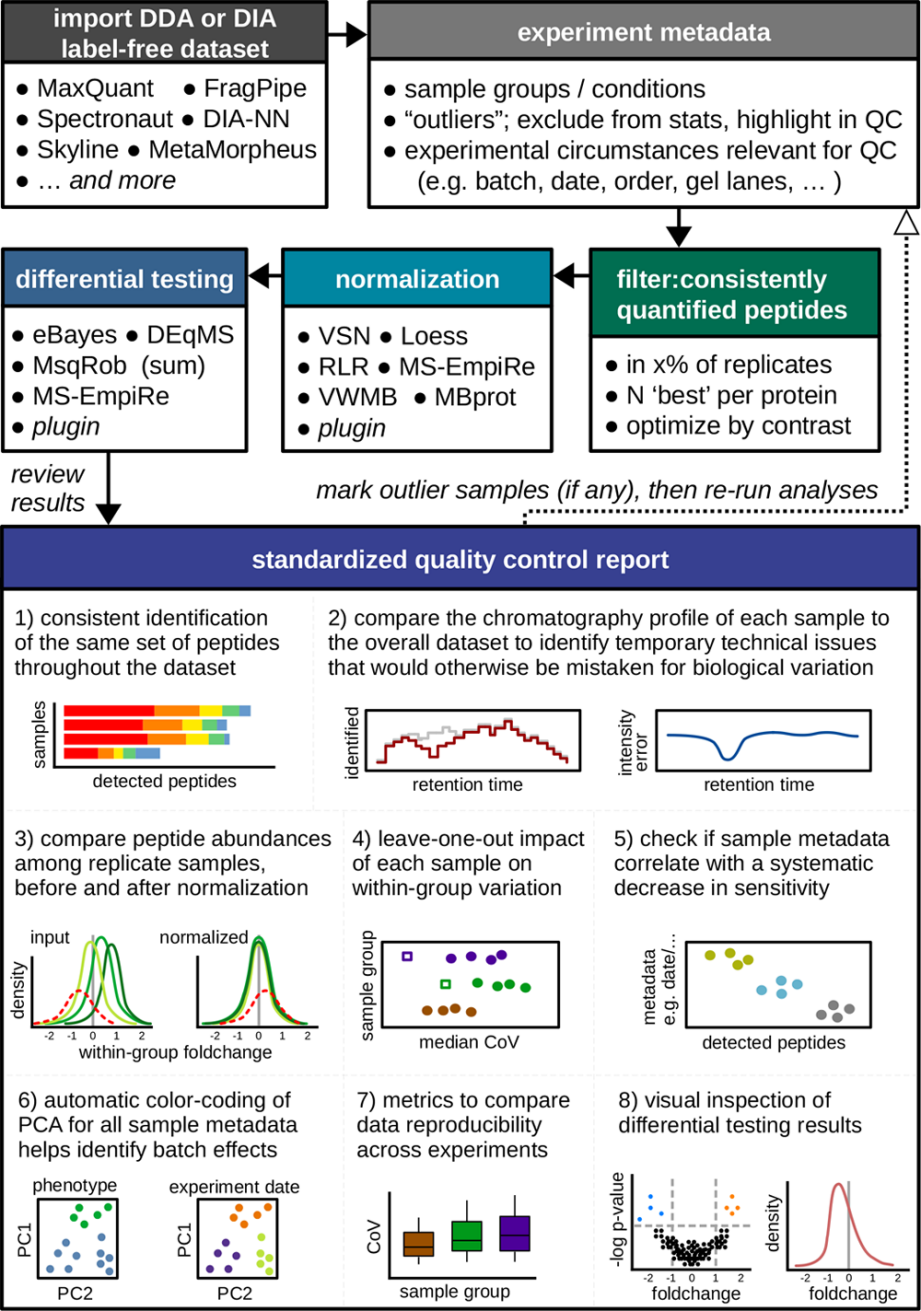
Overview of the MS-DAP workflow and data visualizations. MS-DAP is implemented as an open-source R package and provides a standardized solution for the analysis of label-free proteomics data sets generated from many software platforms. The pipeline facilitates multiple stages of quantitative data processing (filtering, normalization and stats) and conveniences application of pre-existing algorithms while also enabling bioinformaticians to provide custom functions (plugins) for normalization and differential testing. A comprehensive quality control report is generated that provides a multifaceted perspective on quality metrics, reproducibility, potential batch-effects and visualization of statistical results. User provided metadata describing experimental conditions, such as wet-lab sample handling steps or mass-spectrometry measurement order, are used to automatically generate respective quality control figures. The report is provided as a single PDF file that contains dozens of unique analyses, documentation thereof and information needed to reproduce results.

We here introduce MS-DAP, implemented in R that facilitates reproducible proteome science by integrating many state-of-the-art algorithms for data filtering, normalization and differential testing (Figure 1). This pipeline is independent of (and compatible with a wide range of) raw data processing software, integrates previously published state-of-the-art algorithms and includes both quality control and differential testing. The reporting is designed as a systematic assessment of data set quality metrics, from individual samples to group- and data set-wide effects, and can be copublished with proteomics studies. The R code that is used to analyze a data set with MS-DAP is automatically included within the PDF report as an audit log and to facilitate accurate reproduction of the results. Custom functions for normalization or differential expression analysis can be used as a plugin to facilitate the development of future algorithmic innovations (tutorials with example code are available online). Besides analysis of proteomics data sets, this also makes MS-DAP suitable as a platform to jump-start benchmarking studies. The pipeline is open-source and available as an R package and Docker container at https://github.com/ftwkoopmans/msdap and comes with extensive documentation.

Together with the integration of existing tools, the pipeline introduces a number of unique features. At the start of the pipeline, the goal of feature selection is to ensure downstream statistical comparisons between experimental conditions based on reliable data features. For instance, peptides consistently observed throughout the data set might be favored over those sparingly observed. Users can opt to remove peptides identified in less than *x*% of replicates per sample group, remove proteins with less than N peptides and/or keep only the top M peptides per protein.

For large data sets with many sample groups, the typical strategy of selecting only those peptides that are consistently identified throughout the data set may be overzealous. For example, let peptide *p* be observed in sample groups *A* and *B* but not in groups *C–E*. Selecting only peptides observed in all groups would remove *p* while it could have potentially been used in a contrast of group *A versus B*. MS-DAP can counter this problem by applying feature selection rules and subsequent normalization in each contrast separately, thereby maximizing the number of reliable features.

For differential testing, MS-DAP supports statistical models that operate on either peptide-level (e.g., MSqRob or MS-EmpiRe) or protein-level data (e.g., eBayes or DEqMS). In addition to a *q*-value threshold, users may also provide a foldchange threshold to define significant hits or let MS-DAP estimate a foldchange threshold from a permutation test.^42^ MS-DAP’s flexible framework allows users to apply a DEA of choice, or multiple DEA methods in parallel and compare their results downstream. Besides these integrated methods, custom functions for normalization or differential expression analysis can be used as a plugin to facilitate benchmarking studies and encourage inclusion of future algorithmic innovations (tutorials with example code are available online).

### Data visualization and standardized reporting

In addition to typical quality control figures, MS-DAP automatically generates visualizations for all user-provided sample metadata (Figure 1). For example, if sample metadata denotes the preparative step used for each sample, the data visualizations will indicate preparations that resulted in more/less identified peptides, produced outlier samples as compared to replicates in others or whether samples from the same preparation cluster in PCA. This allows MS-DAP users to quickly identify potential improvements to experiment protocols and iterate pilot experiments to evaluate improvements in terms of reproducibility and elimination of confounding factors. Furthermore, observed batch effects (e.g., cohorts) can be easily taken into account in statistical analyses by modeling sample metadata as random variables in regression models such as MSqRob or DEqMS. An example of this is demonstrated later on in Figure 4.

To facilitate transparency, we favor to not fully remove “outlier samples” from published data sets but instead copublish these data and only omit respective samples from statistical analyses. Users can indicate which samples should be excluded from DEA; these samples will subsequently be highlighted in quality control figures to provide transparency regarding the rationale for exclusion (examples in Figure S1, complete report with real-world data in Supporting Data 1).

The MS-DAP report also features unique visualizations of chromatography profiles for detecting temporary or systematic effects throughout chromatographic retention time. These can reveal chromatographic shifts between samples and peptide abundance alterations that correlate to some moment in elution time (example in Figure S1C, complete report with real-world data in Supporting Data 1). Various perspectives on outlier analysis are provided, such as identified peptide counts per sample and sample-group, peptide abundance distributions, peptide foldchange distributions among replicates, leave-one-out impact on within-group variation and sample clustering by probabilistic-PCA.

### Novel Mode-Between Normalization Augments Current Methods

Data normalization is a crucial preprocessing step with major impact on statistical analysis of high-throughput omics data.^20,21^ Prior to statistical analyses, normalization algorithms are applied to label-free proteomics data in order to minimize variation among samples (e.g., caused by differences in sample loading). However, it is important that these algorithms also ensure the data set adheres to assumptions that underly downstream statistical tests; when comparing the abundance of a protein between two conditions, the null hypothesis is that the two means are equal and the alternative is that they are not. Ergo, after normalization we expect (most) proteins to have a log foldchange of zero. The MS-EmpiRe normalization method explicitly aims to achieve this. But commonly used algorithms like VSN, Loess or naïve algorithms that simply minimize variation for most proteins, do not. Especially for asymmetric data sets where the number of up- and down-regulated proteins is not the same, this can be challenging but normalization algorithms should be able to deal with these as well (e.g., suppose we compare control samples to a condition with induced cell death for a subset of cells such that the majority of proteins remains unchanged and differential regulation is all toward down-regulation). To inform MS-DAP users on recommended settings, we first performed extensive benchmarking analyses of 5 normalization algorithms; VSN,^20^ Robust Linear Regression (RLR), MS-EmpiRe’s normalization,^19^ Loess^23,43^ and an algorithm that naively minimizes peptide variation over all samples.

**Figure 2:**
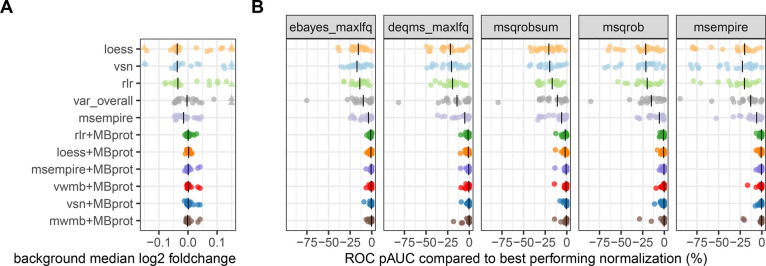
A novel normalization strategy included with MS-DAP rescues deficiencies observed in current algorithms. (A) The median value of the protein-level log2 foldchange distributions for the subset of background proteins represents how well centered the data set is post normalization (should be zero, i.e., typical background proteins should not be changed between experimental conditions). After peptide-level normalization with any algorithm of choice, adding our protein-level mode-between normalization (denoted here as +MBprot) strongly improved results in all data sets. Extreme values depicted at plot limits as a triangle. (B) The normalization performance from panel A was evaluated in the context of downstream statistical analysis using various DEA algorithms; DEqMS after MaxLFQ rollup, eBayes after MaxLFQ rollup, MSqRobSum, MSqRob and MS-EmpiRe. The *x*-axis shows how far the partial Area Under Curve (pAUC) of ROC analysis is from the best performing normalization approach of the same data set and DEA algorithm combination (closer to zero is better, implying other normalization algorithms do not achieved a better pAUC score). Similar to panel A, after posthoc application of MBprot the performance of all normalization strategies was similar and much improved from initial application of these algorithms as-is. In both panels A and B, each point represents one of the 22 evaluated statistical contrasts (Table S1) and median values over all contrasts are shown as vertical black lines.

**Figure 3:**
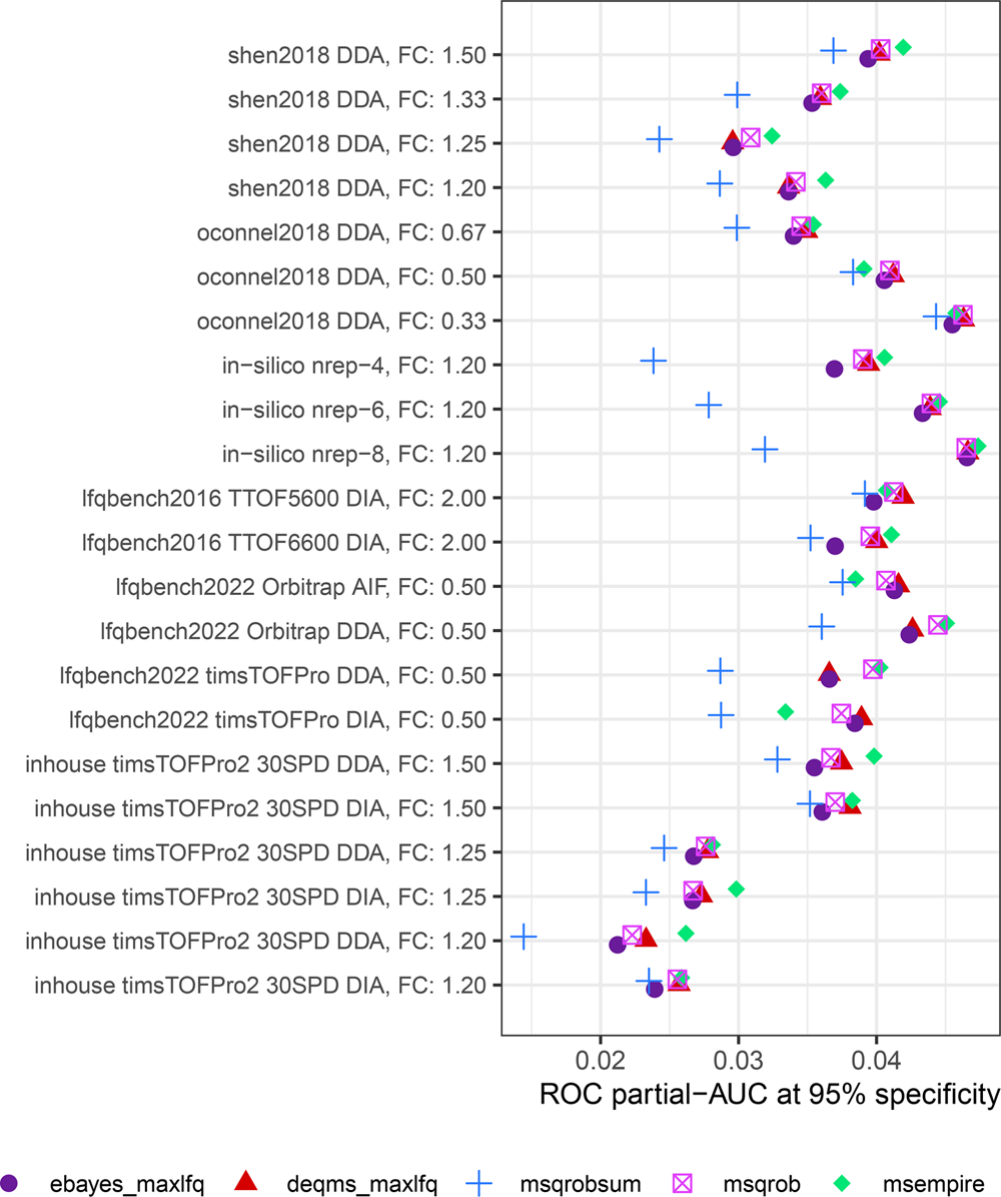
Benchmarking differential expression analysis algorithms. Partial Area Under Curve (pAUC) at 95% specificity was used to quantify how well the estimated *p*-values from each DEA algorithm discriminated true- from false-positives in each of the 22 statistical contrasts. The spike-in ratio between experimental conditions is included in the label for each contrast (denoted as FC). Among this diverse collection of data sets, MS-EmpiRe outperformed other methods most often.

We used a diverse collection of benchmark data sets as to avoid bias in our evaluation of normalization algorithms toward a particular data set (which might suit some algorithm in particular). In total we included 22 statistical contrasts, originating from published spike-in DDA and DIA proteomics data sets, a simulated in-silico data set and a challenging data set we created in-house with minor (20% and 25%) spike-in ratios that was measured in both DDA and DIA mode using a Bruker timsTOF Pro 2 (Figures 2 and 3, Table S1, see further Materials and Methods).

Raw data of each data set was first reanalyzed with recent versions of MaxQuant and DIA-NN, after which results were imported into MS-DAP and respective normalization algorithms were selected prior to DEA with (in parallel) eBayes, DEqMS, MS-EmpiRe and MSqRob. As a result, we obtained a log2 foldchange and p-value from these 4 DEA methods, for all proteins in each of the 22 statistical contrasts. Because these are spike-in data sets, the background set of proteins (e.g., HeLa cells present in each sample at the same concentration) is expected not to change between experimental conditions. However, we observed many contrasts where the log2 foldchanges of the background proteins were not centered around zero (Figure 2A).

Density plots of the log-transformed protein foldchange distributions, included in the MS-DAP quality control report for every statistical test, showed that for some data sets the most frequent protein foldchange (the mode of the log foldchange distribution) was not zero and therefore the assumption that it is most common for proteins to be unchanged (null hypothesis) was violated. It is hard to read this from a volcano plot as many proteins, especially those with foldchanges close to zero, will be drawn on top of each other so foldchange distribution plots are a more reliable way to assess this.

We first implemented an algorithm that scales samples such that the variation of peptide intensity values within each group is minimized, and then uses the peptide foldchange distribution to normalize between groups such that the log foldchange mode is closest to zero. We refer to this approach as Variation Within, Mode Between (VWMB). The MS-EmpiRe paper^19^ also described foldchange-based normalization and used mode-foldchange scaling both within and between groups, and was also applied to peptide-level abundance matrix prior to application of the MS-EmpiRe DEA algorithm. Key differences with our implementation include our emphasis on efficiently scaling to many groups in large data sets and flexibility to minimize either “variation over replicates” (VWMB) or “mode-foldchange” (MWMB) for the initial within-group normalization step. Minimizing variation over replicates (the within-group part of VWMB) assumes there are no cofactors among samples within a sample-group that affect many peptides, if there are one would risk “averaging out” some of the biological variation in this normalization step. In which case the MWMB variant would be more applicable, which also uses mode-foldchange normalization for the within-group normalization and therefore might be more robust when dealing with cofactors not included in the sample-group definition. Furthermore, in comparison to VWMB/MWMB, MS-EmpiRe normalization uses clustering to iteratively determine reference samples to scale to whereas our implementation minimizes overall distance (e.g., between all pairs of sample groups). The consequence of these different approaches might not be revealed in homogeneous benchmarking data sets that only have a few technical replicates and few experimental conditions.

While this worked pretty well overall, in some data sets the protein-level log foldchange distributions obtained from statistical models were not centered around zero. We hypothesized this might be due to a small set of differentially expressed proteins that has a large number of peptides, and therefore the centered peptide foldchange distribution did not result in centered protein foldchange distributions after application of peptide-level statistical models nor after peptide-to-protein rollup followed by protein-level statistical models. Note that typically, the distribution of the number of peptides per protein looks like a power law (i.e., huge number of proteins have 1 peptide, minor set of proteins has 10+ peptides but some proteins may have hundreds of peptides). So, to ultimately correct for deficiencies in between-group normalization while starting from peptide-level data, we developed a novel normalization algorithm that scales the abundance value matrix such that the mode of protein-level log foldchanges between sample groups is zero (MBprot). In MS-DAP it can be used in conjunction with existing normalization approaches, which typically aim to reduce variation between samples, to posthoc balance protein foldchanges between groups (denoted throughout this study as +MBprot, e.g., VSN+MBprot). Posthoc application of our MBprot normalization algorithm strongly improved results for all evaluated normalization algorithms, on all data sets and statistical contrasts (Figure 2A).

Next, we evaluated the impact of normalization algorithms on subsequent differential testing between experimental conditions; spike-in proteins should have a stronger (lower) *p*-value than background proteins which should be unchanged between conditions. We quantified this by computing Receiver Operating Characteristic (ROC) curves for each of the 22 statistical contrasts, for each (combination of) normalization algorithm(s), for 4 DEA algorithms. Then we computed the partial area under the curve (pAUC) at 95% specificity, a metric for how well the ordered *p*-values (of some DEA algorithm in some contrast) prioritize true- over false-positives and compared the performance of each normalization against the best pAUC over all normalizations (within same contrast and DEA). This allowed us to see if the improvement that our MBprot algorithm showed in the evaluation of background foldchange distributions (Figure 2A) also resulted in better background/foreground separation in DEA, and indeed it did in all cases (Figure 2B). Application of MBprot on peptide-level data that was normalized with any commonly used normalization approach improved ROC performance over evaluated statistical contrasts, with median improvement of ROC pAUC; MS-EmpiRe 3.93%, variance minimization 12.0%, RLR 17.8%, VSN 19.4%. Furthermore, differences between normalization algorithms (when evaluating the same statistical contrast) strongly decreased (standard deviation of pAUC distances were 0.11–0.20 when using normalization algorithms as-is and 0.02–0.03 after application of MBprot) which implied protein-level between-group scaling was a key aspect of successful normalization throughout all evaluations, even for data sets with only minor asymmetry such as the in-silico generated data set.

Unfortunately, all normalization methods may potentially introduce unwanted bias because in real-world data sets the actual foldchange null distribution (proteins unchanged between conditions in vivo) is unknown and therefore it is difficult to verify (in a real-world data set that is being analyzed) how accurately mode-between/VSN/Loess/RLR centers the null distribution. For instance, when mode-between normalization is applied to data sets with highly convoluted bimodal distributions of background and foreground proteins it may slightly bias the estimation of “zero log-foldchange” toward the foreground proteins thereby reducing the absolute foldchange of foreground proteins and inducing some unwanted foldchange in the set of background proteins.

Taken together, we recommend that the DEA results from every analyzed proteomics data set are carefully inspected for off-centered log foldchange distributions (e.g., using data visualizations included in standardized MS-DAP reports). Regarding choice of normalization algorithms, we recommend VSN combined with protein mode-between (MBprot) normalization as the default starting point when analyzing a data set. However, note that VSN tends to “shrink” foldchanges which in practice results in slightly underestimated foldchange magnitudes. If this is undesirable, we suggest “vwmb” (minimizes variation within group/replicates) for data sets without (strong) cofactors, or “mwmb” otherwise (scales the mode-foldchange between pairs within-group samples), both should be combined with protein mode-between (MBprot) normalization.

### Imputation

Imputation of missing values is sometimes applied in the analysis of proteomics data set, but it is difficult to assess whether missingness in a data set is missing at random (MAR), missing not at random (MNAR) or some combination of both (e.g., high abundant peptides are missing at random, low abundant are more likely to be missing not at random/left-censored). Furthermore, DIA data sets typically have a lower fraction of missing values than DDA data sets and it is likely that this model of missingness is different between DDA and DIA data sets. Given a real-world data set where ground-truth is not known, it may prove quite difficult to assess the mechanism of missingness and subsequently select the appropriate imputation algorithm. Consequentially, one may risk selecting an imputation method with a different assumption on the model of missingness than the (unknown) true generative model of the data set and thereby introduce bias. Imputation algorithms come with their own set of assumptions, might introduce bias and large differences in the effectiveness of various imputation algorithms has recently been shown.^44^ We have not implemented imputation in MS-DAP as of yet, for now assuming unobserved peptides to be missing at random and let the statistical models assess the data as is. Future extensions to MS-DAP could implement imputation using the plugin architecture. Even with the option of imputation, we expect it will still be beneficial to first filter stringently as to remove peptides where most values are missing, and then apply imputation on the remaining data set which should have a relatively low fraction of missing values. Extensive benchmarking across both DDA and DIA data sets, as well as interaction of imputation methods with subsequent DEA algorithms, would be required to establish which approach performs best over a wide range of data sets and introduces least bias.

### Benchmarking Differential Expression Analysis Algorithms: Peptide-Level Statistical Models Are More Sensitive

After evaluation of normalization algorithms, we proceeded with benchmarking Differential Expression Analysis (DEA) algorithms. The same data sets and contrasts were used, covering a wide range of label-free proteomics data sets (DDA, DIA, slightly older and current data sets, different mass-specs vendors, etc.), but now compared the performance of DEA algorithms within each data set. Following results from the previous section, we used VSN combined with protein-level mode-between normalization (MBprot) throughout.

Comparing DEA algorithms using ROC within each of the 22 statistical contrasts, again using the metric of partial area under the curve (pAUC) at 95% specificity, we found that the recently introduced algorithms DEqMS, MSqRob and MS-EmpiRe performed better than the approach used in most proteomics pipelines, protein-level moderated t-statistics here evaluated using the popular limma eBayes implementation, in 19 out of 22 statistical contrasts (Figure 3).

For protein-level statistical models, the method used to collapse peptide-level data per protein (rollup) is crucial. In MS-DAP, all data analyses start at the peptide-level, so we implemented and compared the traditional protein summarization “sum” approach (protein abundance = sum of respective peptide intensities per sample), rollup by applying Tukey’s median polish (TMP) to respective peptide*sample subsets of the data matrix, and the MaxLFQ algorithm as provided by the iq^45^ R package. Peptide to protein rollup with both MaxLFQ and TMP systematically outperformed the naïve “sum” approach for both eBayes and DEqMS, whereas MaxLFQ and TMP performance was generally on par (Supporting Data 2).

Computation times for eBayes and DEqMS were below 10 s on all data sets, whereas peptide-level models MS-EmpiRe and especially MSqRob would take 10–30 min per contrast depending on the number of peptides (Figure S2). DEqMS showed consistent improvement over the eBayes method with varying degrees of magnitude, which was expected as the former method is an extension of the latter. The DEqMS paper^22^ reported similar results albeit on a limited set of experimental data whereas our benchmarking cross-compared more DEA algorithms and includes many more, and diverse, data sets. Overall, MS-EmpiRe performed best in the ROC analyses (mean pAUC over all contrasts improved by 5.5% as compared to eBayes) with MSqRob (mean improvement 2.9%) and DEqMS (mean improvement 3.1%) closely behind.

While MS-EmpiRe performed best overall and had a slight edge over MSqRob and DEqMS on most evaluated data sets (Figure 3), a major advantage to the latter two is the flexibility to add random variables to the regression model to account for additional experimental features such as batch effects, while MS-EmpiRe is only suited for A/B testing. We observed a superior performance of MS-EmpiRe mostly on DDA data sets (and less so for DIA) and hypothesize this could relate to differences in the peptide variation versus intensity relation, which is a crucial aspect of the MS-EmpiRe model, that is different between these acquisition modes. Combining the strengths of both MSqRob and MS-EmpiRe seems a promising avenue for future work on DEA algorithms. In conclusion, our extensive evaluation of DEA algorithms makes a strong case for updating pre-existing proteomics workflows that still employ generic moderated t-statistics to protein abundances in favor of recent proteomics DEA methods DEqMS, MSqRob and/or MS-EmpiRe.

### Generation of a Challenging Spike-In Benchmark Data Set

We generated in-house benchmark data sets to further evaluate DEA algorithms under challenging conditions that may better represent real-world experiments. We used low sample loading to both mimic “single cell” proteomics and avoid ion suppression at saturation loadings (60–70 ng whereas the Bruker timsTOFpro 2 could easily process more than double the sample load), short gradients to represent high-throughput proteomics (Evosep, 30 samples per day) and minor spike-in foldchanges of 20, 25 and 50% to simulate foldchanges expected in biological data sets.

The resulting data showed that it is possible to generate DIA benchmark data sets that exhibit false-positive rates in line with the DDA data sets (using the exact same raw data processing software and MS-DAP settings as the LFQbench data sets). The modern DEA algorithms DEqMS, MSqRob and MS-EmpiRe outperformed eBayes in ROC analyses in all cases with varying degrees of magnitude (Figure 3) and importantly, their sensitivity was much better. At 1% FDR, these 3 algorithms uncovered hundreds of true positive significant hits whereas eBayes recovered only a fraction thereof or none at all for some contrasts (Figure S3).

While spike-in experiments and synthetic data sets are a common approach to benchmark computational pipelines, as these contain both data and ground truth which allows for evaluation of true/false-positives and -negatives, these are not without flaws. Such benchmarking data sets may not be representative of challenges faced in real-world experiments, which typically have more complicated experimental designs than replicates with only technical variation (e.g., covariates like “batch” or “sex”) and a single ratio for all differentially expressed proteins (e.g., more complicated foldchange distributions for up/down-regulated proteins).

We generated an in-house benchmark data set with reduced sample loading and only minor foldchanges between spike-in conditions in an attempt to better simulate mass-spectrometric conditions of differentially expressed proteins in a benchmark data set. This data set exhibited different characteristics compared to other benchmark data sets, such as reduced false-positive-rates in evaluation of DEA algorithms, when compared to other benchmark data sets.

Our results underline the importance of the data sets used to benchmark algorithms for normalization and DEA, and suggest more work is needed on crafting benchmark data sets that better mimic real-world experiments. Future research should also investigate the root cause of high false positive rates observed here for DIA data sets by interrogating the entire process of data acquisition, raw data processing and downstream analyses, which might require a series of additional experiments at increasing sample loadings and varying mass-spectrometer configurations.

### MS-DAP Settings and Interpreting Results from Statistical Analyses

While ROC analyses quantify how well the (ranked) *p*-values from differential expression testing prioritize (a priori known) true positives over false-positives, this metric may not reflect how users ultimately interpret the data. Typical interpretations of DEA results from proteomics studies are Geneset Enrichment Analyses (GSEAs), which may use *p*-values as is or rank transformed data, and researchers interpreting the top-hits from the study. However, in most cases an arbitrary cutoff is applied to the FDR corrected *p*-values (typically 0.05, 0.01 or 0.001) and the remaining subset of “significant hits” is then used to interpret the study results. Hence, it is important that DEA algorithms have well calibrated *p*-values, i.e., one expects that a benchmark data set with a 1% FDR cutoff applied indeed yields 1% false-positives (background proteins) and the remainder are true positives (spike-in proteins).

We compared the number of true- and false-positive proteins in each statistical contrast at 1% FDR cutoff for each DEA algorithm and found that results were mostly in line with expectations from the ROC analyses in Figure 3 (Figure S3). However, some of the DIA data sets yielded a large number of false positives at the 1% FDR cutoff for all evaluated DEA algorithms (Figure S3) and results were similar for all evaluated normalization algorithms.

A complicating factor is that the typical spike-in benchmark data sets generated for label-free proteomics are not perfect ground-truth, due to a myriad of technical challenges. For instance, peptides might be mis-identified or peak-picking might be challenging for low abundant peptides. Or, background proteins may produce peptides that are low abundant, or ionize poorly and therefore are have low ion intensity, and as a result are more susceptible to ion suppression that varies between experimental conditions due to coeluting high abundant spike-in peptides. Both the 2016 and 2022 LFQbench data sets were 3-proteome mixtures (Human as background, Yeast at a 2-fold ratio, *E. coli* at a 4-fold ratio). We hypothesize that high sample complexity combined with high sample loading contributed to the unexpected false-positive-rates observed for these data sets, as this may lead to highly complex spectra (i.e., challenging for raw data processing software) and increased likelihood of ion suppression (e.g., 4-fold increase of a large set of *E. coli* peptides affects the population of background proteins).

Compared to other DIA data sets, we observed a well-controlled false-positive-rate with our in-house data set at low sample loading and modest spike-in differences. Especially when additional foldchange cutoffs were applied to the 1% FDR filtered results (Figure S3B). In typical proteomics data sets, as much as ∼20–30% of proteins are quantified by only 1 peptide. At the trade-off of losing information about this large subset of proteins, filtering the data set such that only proteins with multiple peptides were used for statistical analyses improved the ROC pAUC results as expected and showed DEqMS, MSqRob and MS-EmpiRe outperformed eBayes in this scenario as well (Figure S4).

Extensive data visualizations are provided in Supporting Data 2 which include for each statistical contrast; log2 foldchange distributions, ROC curves for all proteins and the subset of proteins with at least 2 peptides, bar graphs for the number of significant true/false-positive proteins at various significance criteria (all proteins/at least 2 peptides, with/without additional filtering by bootstrapping estimated foldchanges).

In data sets where researchers are concerned with inflated false-positives from the increasingly sensitive peptide-level models as compared to, e.g., protein-level DEqMS, we suggest to restrict DEA results to proteins with at least 2 peptides and apply unbiased foldchange thresholds estimated from bootstrapping procedures as an extra posthoc filter (both filtering steps are easily performed with MS-DAP) as this combination of stringencies controlled false positive rates very well in our challenging in-house data set and still yielded more true positive hits than protein-level models without these additional filters.

Informed choices have to be made when analyzing proteomics data, from selecting and configuring raw data processing software, to configuring various downstream analysis steps that are here described as part of MS-DAP, such as data filtering, normalization and differential expression analysis. The best (combination of) tools for the job may depend on the experiment and mass spectrometry platform. For example, proteomics data sets may exhibit different characteristics depending on the acquisition method, number of identified/quantified features, experimental and biological variation, number of available replicates, etcetera and, consequentially, data pipeline configurations that work well for one data set may not be optimal for another.

In this paper we have used a wide range of benchmark data sets to evaluate algorithms integrated in MS-DAP. The methods for normalization and DEA that were among the top-tier best performing over many tests are our recommendation as a starting point for analyzing data sets with MS-DAP. However, as there was no algorithm that was the single best in every benchmark analysis we encourage MS-DAP users to make use of its flexibility in applying various algorithms. Considering both the interpretation of differences in statistical analysis results between different MS-DAP configurations and a comparison of data visualizations included in the standardized PDF reports (e.g., outlier sample analyses and log-foldchange distribution plots), MS-DAP users can make informed choices on adjusting the recommended settings as appropriate for their data sets.

### Reanalysis of AD Studies with MS-DAP

Benchmark data sets with large artificial differences between experimental conditions may not represent real-world conditions. Therefore, we turned to a recent large-scale study by Bader et al.^41^ on AD in which cerebrospinal fluid (CSF) between AD and control cases was compared using label-free proteomics. Samples from 197 subjects in three independent studies (cohorts) were analyzed in DIA mode and the raw mass spectrometry data was processed with Spectronaut.^17^ In the original study, statistical tests were first applied on each cohort independently and then the subset of proteins that differed significantly (*p*-value < 0.05, without multiple-testing correction) in each cohort was put forth as a shortlist of 43 proteins-of-interest (Bader et al. Figure 3F^41^).

Reanalysis in MS-DAP was performed in a two-step process. First, MS-DAP was applied to the Spectronaut report provided with the original study at default settings, but without performing DEA, to generate the MS-DAP quality control report. A few clear outlier samples were observed, which we subsequently flagged as “exclude” in the sample metadata Excel table that was used for further analyses (meaning these are included in all data visualizations but excluded from statistical analysis). For example, two outlier samples from the AD group in the Berlin consortium, as compared to respective biological replicates, were observed while the vast majority of all samples in this study showed excellent reproducibility. As part of the standardized MS-DAP quality control report (Supporting Data 1), a rare example of chromatographic aberration, in which peptide abundances are shifted from expected values as a function of retention time, was observed in one of the samples (“A6_82545313”). This was of no effect to the outcome of the study since this sample was not classified as either control or AD in the sample metadata provided with the original study, thus not part of statistical analyses, but it nicely illustrated how MS-DAP quality reporting uncovers such aberrations.

**Figure 4:**
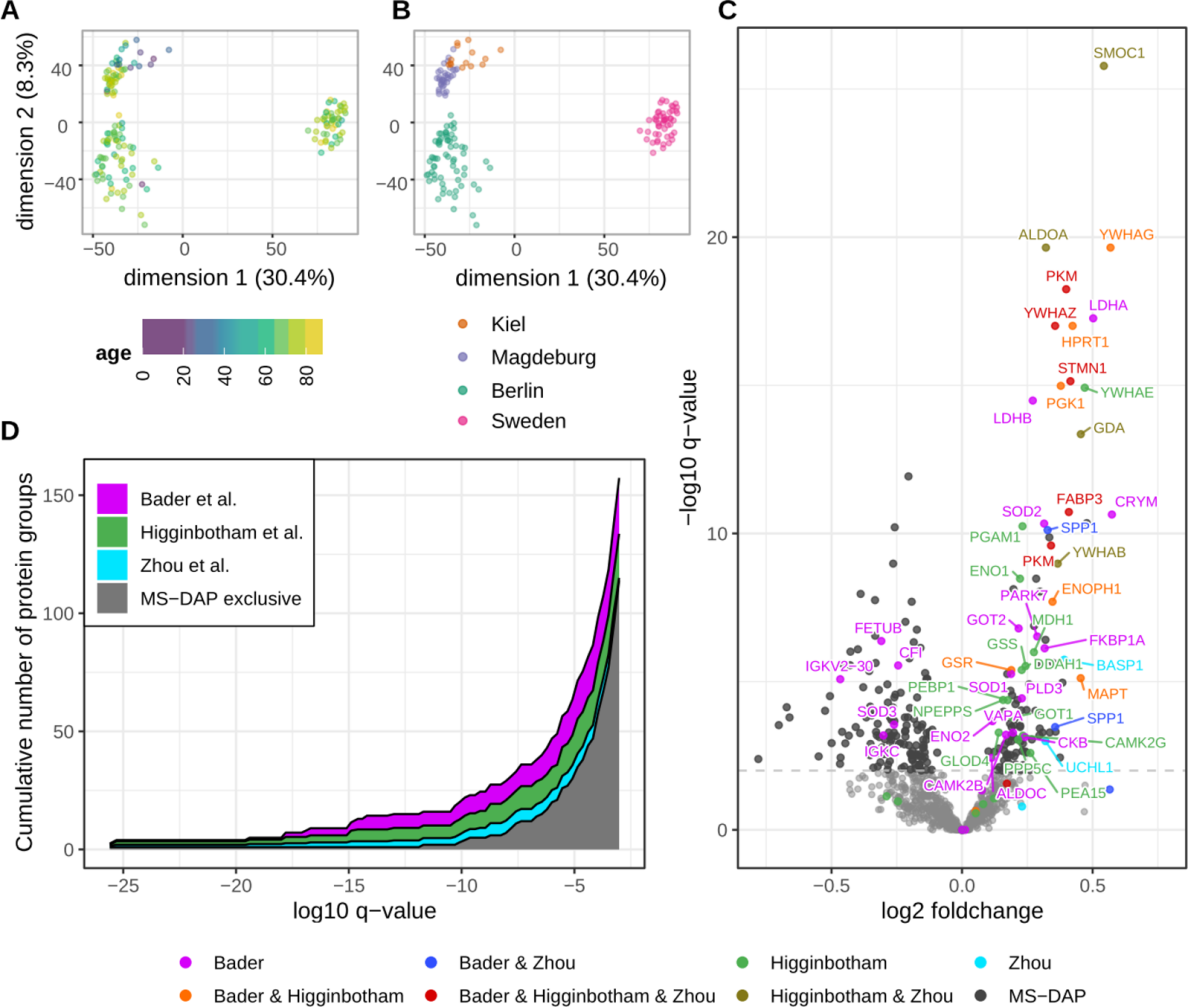
MS-DAP reanalysis of the Bader et al. AD CSF biomarker data set. (A,B) The MS-DAP report includes PCA visualizations that are automatically color-coded by all user-provided sample metadata. In the examples highlighted here, individuals with similar age do not cluster together while a clustering of individuals from the same cohort is apparent (as expected). (C) Volcano plot visualization of the differential testing results produced by MS-DAP. The 1% FDR significance threshold is shown as a dashed horizontal line. Proteins that reached significance in the original study prior to multiple testing correction are highlighted (“Bader”), together with top-hits from other recent AD CSF proteomics studies (Zhou et al. and Higginbotham et al.). Proteins labeled “MS-DAP” did not overlap with any of the proteins reported by these 3 AD CSF studies. (D) Analogous to panel C, the stacked area chart shows that previously reported top-hits from the original Bader et al. paper and 2 other AD CSF studies are among the highly significant proteins in our MS-DAP reanalysis of the Bader et al. data set.

In a second step, MS-DAP was reapplied with the updated sample metadata table (i.e., some samples “excluded” from DEA). As expected, the batch effect of cohorts in this study was clearly observed in PCA visualizations (Figure 4A,B), whereas age had no pronounced effect on sample clustering (Figure 4A). So, in this a real-world example the MS-DAP standardized quality control reporting effectively corroborated a priori expected batch effects (multiple laboratories/cohorts). Related to this, we strongly recommend users to always include relevant experimental circumstances in the sample metadata table, and inspect these (and other) automatically generated data visualizations to check for expected and unexpected covariates that may need to be accounted for in statistical analyses. The MSqRob peptide-level regression model was used to identify differentially expressed proteins between control and AD cases using all samples in the data set in a single analysis (apart from those flagged as “exclude” based on QC), accounting for batch effects by adding “cohort” as a random variable in the regression model (simple MS-DAP parameter).

Increased sensitivity of the MS-DAP workflow, revealing 245 unique proteins at 1% FDR and 159 highly significant at 0.1% FDR as compared with 43 proteins-of-interest highlighted in the original study (no multiple testing correction was applied there), was confirmed by the detection of paralogs of proteins found in the original study and increased overlap with related AD studies (Figure 4C,D).

From the set of 159 highly significant proteins detected by MS-DAP at 0.1% FDR, 113 did not overlap with previously reported results from the original Bader et al. study nor with 2 other AD CSF studies (Figure 4, Table S2). The MS-DAP exclusive hits include 20 proteins from the immunoglobulin family (30 among all significant at 1% FDR), 2 of which were also reported by Bader et al. Where YWHAG and YWHAZ were previously found by Bader et al., MS-DAP significant hits included these and additional paralogs YWHAB and YWHAE. Similarly, CAMK2B, CAMK2G and CAMK2D are all significant according to MS-DAP whereas only CAMK2B was found by Bader and colleagues. Furthermore, a large set of these proteins contain catalytic activities (e.g., *MMP2*, *CTSZ*) or are enzyme inhibitors (*Serpin-A1*, -*A6*, -*A7*, -*E2*, -*F1*, -*G1*). We found for 25 proteins (of 113 MS-DAP exclusive hits) a described association with Alzheimer’s disease in literature (the full list is appended to Table S2). For instance, macrophage migration inhibitory factor (MIF) is a pro-inflammatory cytokine. Zhang and colleagues suggest that neuronal secretion of MIF may serve as a defense mechanism to compensate for declined cognitive function in AD, and increased MIF level could be a potential AD biomarker.^46^ Transthyretin (TTR) has been described as an amyloid-β protein, preventing its deposition and toxicity.^47^ It has been suggested to transport proteins over the blood brain barrier using *LRP1*.^48^ The *FAM3* superfamily member *FAM3C* ameliorates AD-like pathology by destabilizing the amyloid-β precursor.^49^

In the list of MS-DAP exclusive hits just beyond the top-hits at 0.1% FDR, we also observed proteins previously associated with AD. For instance, *SorCS2* (*q*-value 0.002) belongs to the *Vps10p*-domain family of multiligand receptors, and is implicated as genetic risk factor in sporadic and autosomal dominant forms of neurodegenerative diseases, including AD. In addition to its function in protein trafficking, the protein may function as cell surface receptor to mediate acute responses to proneurotrophins.^50^ Expression of *C1ql3* (*q*-value 0.003) has been shown in discrete neuronal populations to control efferent synapse numbers; as such it may have a role in synaptic loss in AD.^51^

Taken together, this application of MS-DAP to a real-world data set showed (1) the importance of extensive data visualizations and inspection for covariates that should be included in statistical analyses, (2) that the combined normalization and DEA algorithms that performed well in benchmarking analyses in previous sections also worked well in application to a real-world data set, resulting in increased significant hits as well as increased overlap with related studies and AD associated proteins and (3) that this is easily achieved by using MS-DAP.

## Conclusions

We here introduced MS-DAP, a downstream analysis pipeline for label-free proteomics that in a transparent manner integrates commonly used and recently published methods for normalization and statistical workflows to facilitate reproducible proteome science. Custom functions for normalization or differential expression analysis can be used as a plugin to facilitate the development of future algorithmic innovations, making MS-DAP a suitable platform to jump-start benchmarking studies. Moreover, MS-DAP produces extensive data visualizations and automatically generates quality control visualizations for all user-provided sample metadata in a standardized PDF report that includes documentation. The open-source R package is available at https://github.com/ftwkoopmans/msdap.

To inform users on recommended settings to start their data analyses, a systematic evaluation of common approaches to normalization and statistical analysis in label-free proteomics was performed on a large variety of data sets and revealed key differences. In particular, shortcomings in current normalization approaches were revealed. A novel algorithm was made to rescue their performance in evaluated benchmark data sets. Differential testing using protein-level moderated t-statistics, a commonly used approach in the field, was consistently outperformed by more recent statistical models. In particular for challenging data sets where differences between experimental conditions are minor, represented here by newly generated DDA and DIA spike-in data set generated in-house, we encourage adopting modern statistical methods.

Application of MS-DAP on data derived from large-scale studies of AD cerebral spinal fluid proteomics, with optimal settings derived from our benchmark analyses, uncovered many additional significant proteins. These included increased overlap between related AD studies and previously reported AD associated genes demonstrating the effectiveness and relevance of MS-DAP. Moreover, it is of interest that many of the CSF proteins additionally detected by MS-DAP in this study seem to have a role in the progression of the disease itself, and thus are likely candidates for follow-up biomarker analysis.

## Data Availability

The mass spectrometry proteomics data have been deposited to the ProteomeXchange Consortium via the PRIDE partner repository^36^ with the data set identifier PXD036134.

## Supporting Information Available

Figure S1: Examples of typical quality control data visualizations included in the standardized MS-DAP report; Figure S2: Computation time for DEA algorithms; Figure S3: Number of significant proteins in each benchmark analysis at 1% FDR cutoff; Figure S4: ROC analyses for the subset of proteins quantified with multiple peptides (PDF)
Supporting Table 1: Overview of spike-in data sets used for benchmarking analyses (XLSX)
Supporting Table 2: MS-DAP output table with statistical results for Bader et al. data set (XLSX)
Supporting Data 1: MS-DAP report for Bader et al. data set (PDF)
Supporting Data 2: Data visualizations for benchmarking analyses of all spike-in data sets (PDF)
