# Supplementary material for: MS-DAP Platform for Downstream Data Analysis of Label-Free Proteomics Uncovers Optimal Workflows in Benchmark Data Sets and Increased Sensitivity in Analysis of Alzheimer’s Biomarker Data
Source: J Proteome Res. 2022 Dec 21;22(2):374–86. doi: 10.1021/acs.jproteome.2c00513 (PMC9903323; doi:10.1021/acs.jproteome.2c00513)
Supplement: Supplementary file 1 — pr2c00513_si_001.pdf [file pr2c00513_si_001.pdf]

Supporting Information

MS-DAP platform for downstream data analysis of label-free proteomics uncovers optimal workflows in benchmark datasets and increased sensitivity in analysis of Alzheimer's biomarker data

Frank Koopmans[1], Ka Wan Li[1], Remco V. Klaassen[1], August B. Smit[1]*

[1] Department of Molecular and Cellular Neurobiology, Center for Neurogenomics and Cognitive Research, Amsterdam Neuroscience, VU University, 1081HV Amsterdam, The Netherlands.

Table of Contents

Figure S1: Examples of typical quality control data visualizations included in the standardized MS-DAP report

Figure S2: Computation time for DEA algorithms

Figure S3: Number of significant proteins in each benchmark analysis at 1% FDR cutoff

Figure S4: ROC analyses for the subset of proteins quantified with multiple peptides (PDF)

Supporting Table 1: Overview of spike-in datasets used for benchmarking analyses (Excel)

Supporting Table 2: MS-DAP output table with statistical results for Bader et al. dataset (Excel)

Supporting Data 1: MS-DAP report for Bader et al. dataset (PDF)

Supporting Data 2: Data visualizations for benchmarking analyses of all spike-in datasets (PDF)

A

B

C

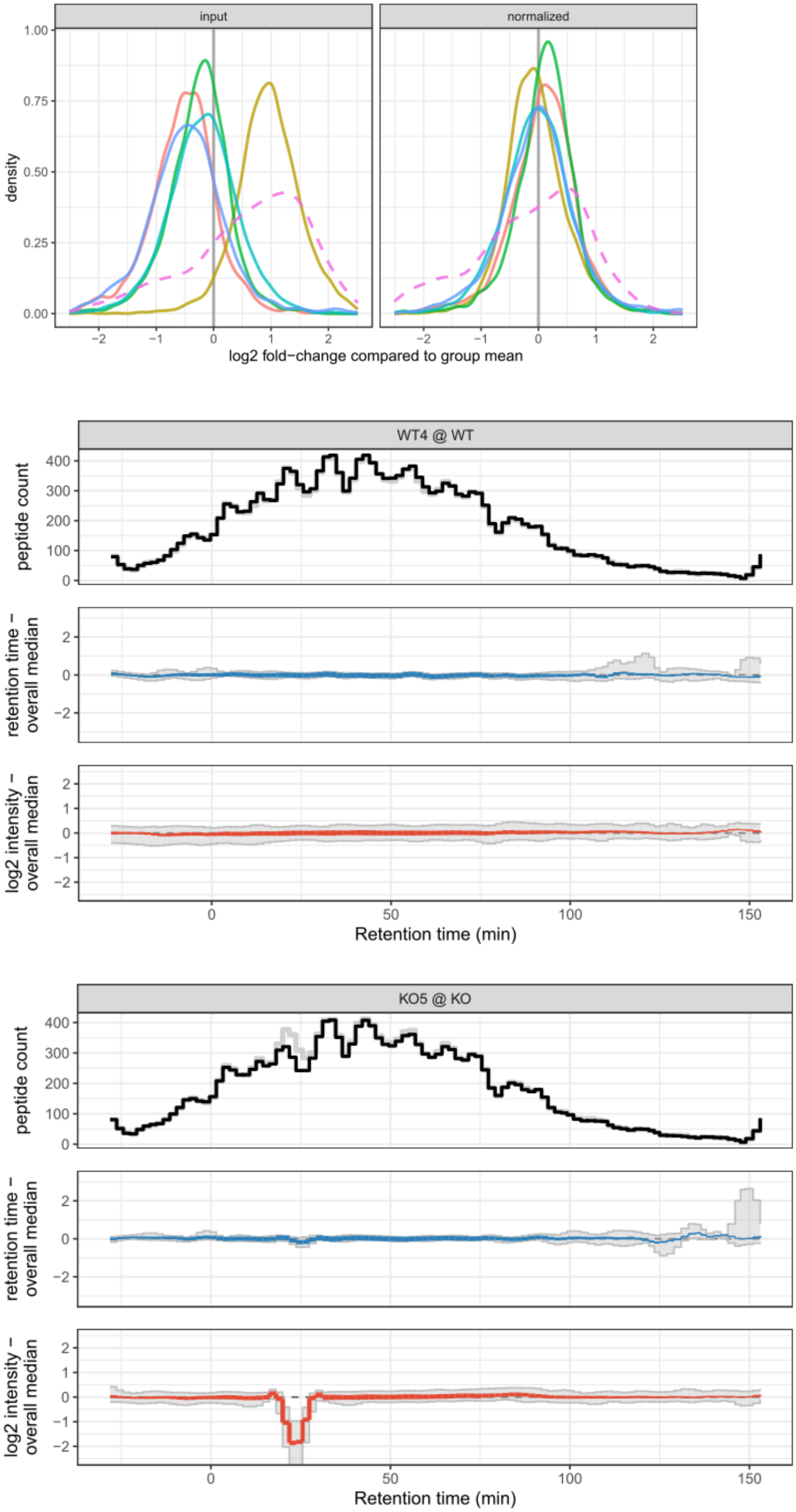
input
normalized
density
1.00
0.75
0.50
0.25
0.00
−2
−1
0
1
2
log2 fold−change compared to group mean
WT4 @ WT
peptide count
400
300
200
100
retention time − overall median
log2 intensity − overall median
50
100
150
Retention time (min)
KO5 @ KO

Figure S1. Examples of typical quality control data visualizations included in the standardized MS-DAP report.

A) Replicate samples from an in-house dataset were compared before and after normalization algorithms were applied (similar figure is included in every MS-DAP report). Peptide abundance values in each sample are compared to the respective mean values over all samples within the sample group. The distribution of foldchanges for each sample is shown as a line. After normalization (right panel), samples are much more similar except for the individual sample we (manually) marked as an ‘outlier’ sample (dashed line). Note that samples marked as ‘outlier’ are retained in the dataset, highlighted in each quality control analysis but ultimately excluded from downstream differential expression analysis.

B, C) Retention time dependent error is reported for every sample in the MS-DAP report. Here 2 samples are shown from an in-house dataset to illustrate expected results (panel B) and how MS-DAP data visualizations can help identify temporary technical noise (e.g., ionization spray issues) that might otherwise be mistaken for biologically meaningful differences between samples. Variation in peptide abundance as compared to the overall median abundance in the entire dataset can be observed at certain moments in elution time (red line, bottom panel). The standardized retention time (iRT) is shown on the x-axis, line thickness indicates the number of identified peptides and the grey shaded area shows the 5-95 % quantiles.

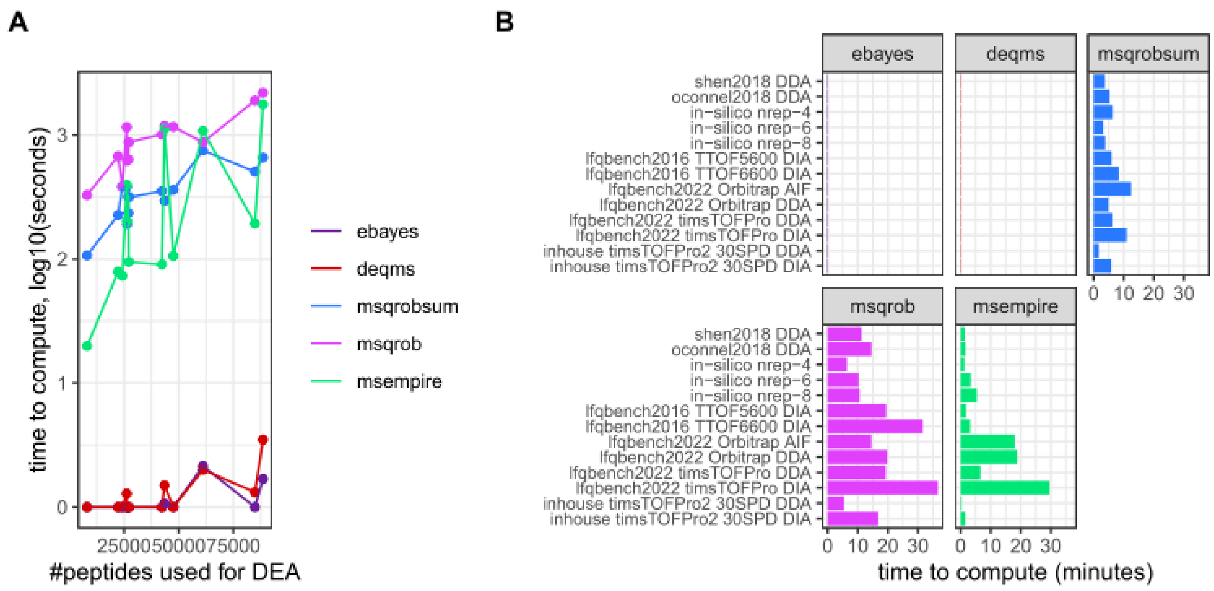

Figure S2. Computation time for DEA algorithms (relating to Figure 3).

A) Time to complete computation with each DEA algorithm on every dataset (median value for datasets that have multiple contrasts). limma eBayes and DEqMS completed in less than 10 seconds in every evaluated statistical contrast, whereas other methods were substantially slower and their required computation time scaled with the number of peptides.

B) Analogous to panel A, but here results are shown per dataset.

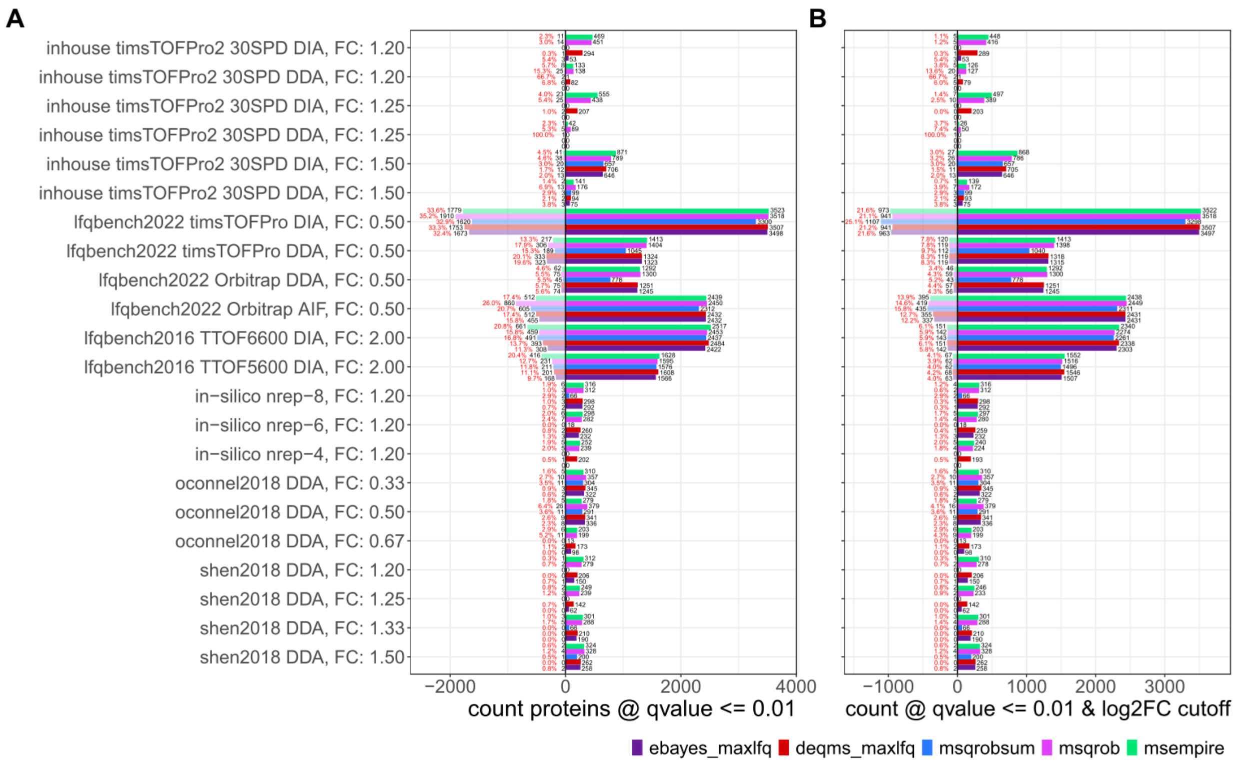

Figure S3. Number of significant proteins in each benchmark analysis at 1% FDR cutoff (relating to Figure 3).

Statistical contrasts from all datasets are shown on the y-axis. False positive and true positive protein-group counts are shown on the x-axis (the former is shown as negative numbers), with False Positive Rates (FPR) shown as red text. A) Results at 1% FDR cutoff, B) additional filtering by protein log2 foldchange cutoffs as estimated by MS-DAP bootstrapping algorithm.

Some datasets suffer have many false positives even though the ROC analyses in Figure 3 showed relatively good performance. For instance, the LFQbench study from 2016 using a SCIEX TTOF660 mass-spec has a pAUC of ~4% at 95% specificity (so 5% would be perfect score) which is reasonably good, however, here we see the number of false negatives is quite high. This implies p-value calibration is off; where the statistical model estimates 1% FDR, we find in this benchmark dataset with ground-truth that the actual empirical false discovery rate is much higher.

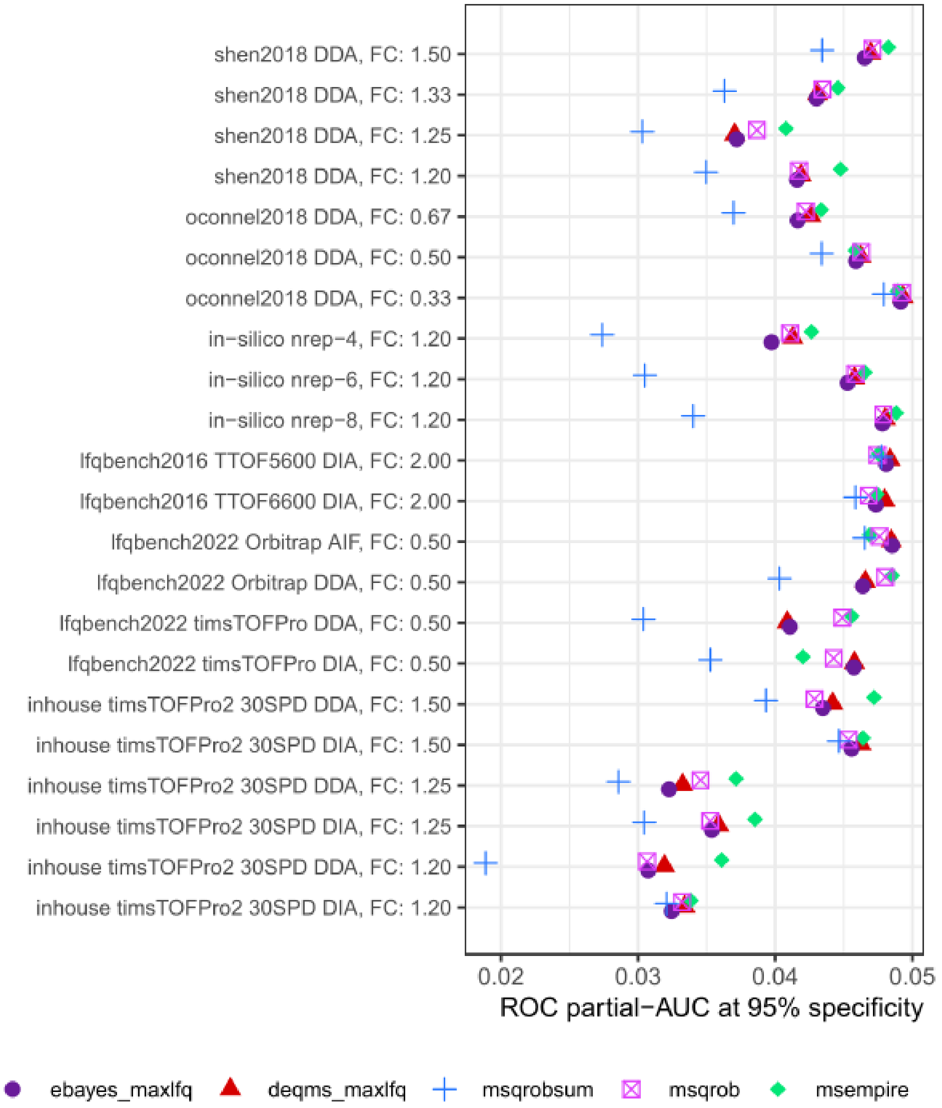

Figure S4. ROC analyses for the subset of proteins quantified with multiple peptides (relating to Figure 3).

The partial Area Under Curve (pAUC) at 95% specificity was used to quantify how well the estimated p-values from each DEA algorithm discriminated true- from false-positives in each of the 22 statistical contrasts. The spike-in ratio between experimental conditions is included in the label for each contrast (denoted as FC). Whereas the related Figure 3 used all proteins in the dataset, here proteins quantified with only 1 peptide were discarded prior to ROC computation.
