# Supplementary material for: MS-DAP Platform for Downstream Data Analysis of Label-Free Proteomics Uncovers Optimal Workflows in Benchmark Data Sets and Increased Sensitivity in Analysis of Alzheimer’s Biomarker Data
Source: J Proteome Res. 2022 Dec 21;22(2):374–86. doi: 10.1021/acs.jproteome.2c00513 (PMC9903323; doi:10.1021/acs.jproteome.2c00513)
Supplement: Supplementary file 4 — pr2c00513_si_004.pdf [file pr2c00513_si_004.pdf]

MS-DAP: Mass Spectrometry Downstream Analysis Pipeline

version: 1.0.3 https://github.com/ftwkoopmans/msdap/

Contents

1 Quality control **2**

1.1 DIA confidence score distributions 2

1.2 number of peptides and proteins 26

1.3 data completeness 34

1.4 abundance distributions 36

1.5 retention time 45

1.6 variation among replicates 152

1.7 PCA 178

2 Differential abundance analysis **194**

2.1 control__Berlin,control__Magdeburg... vs AD__Berlin,AD__Magdeburg,AD__Sweden 194

3 Summary of differential testing **200**

4 log **201**

5 R command history **202**

6 R session info **203**

1 Quality control

The quality control figures in this section enable you to investigate reproducibility and global clustering of samples by visualizing:

- number of peptides/proteins detected in each sample
- dataset completeness
- local effects in HPLC peptide retention time per sample
- reproducibility of peptide quantification among replicates
- PCA of all samples to visualize clustering

The first set of quality control figures describes individual samples, thereafter group-level quality metrics are described and finally sample clustering is used to highlight structure in the entire dataset.

1.1 DIA confidence score distributions

DIA data was used as input for this analysis. Below histograms visualize both target (green) and decoy (grey) cscores in each sample, indicating how confident the input software was in the identification of peptides from the spectral library in the raw DIA data. Samples are ordered by the number of precursors quantified at q-value confidence threshold 0.01. At this threshold, the respective cscore and number of peptides is shown.

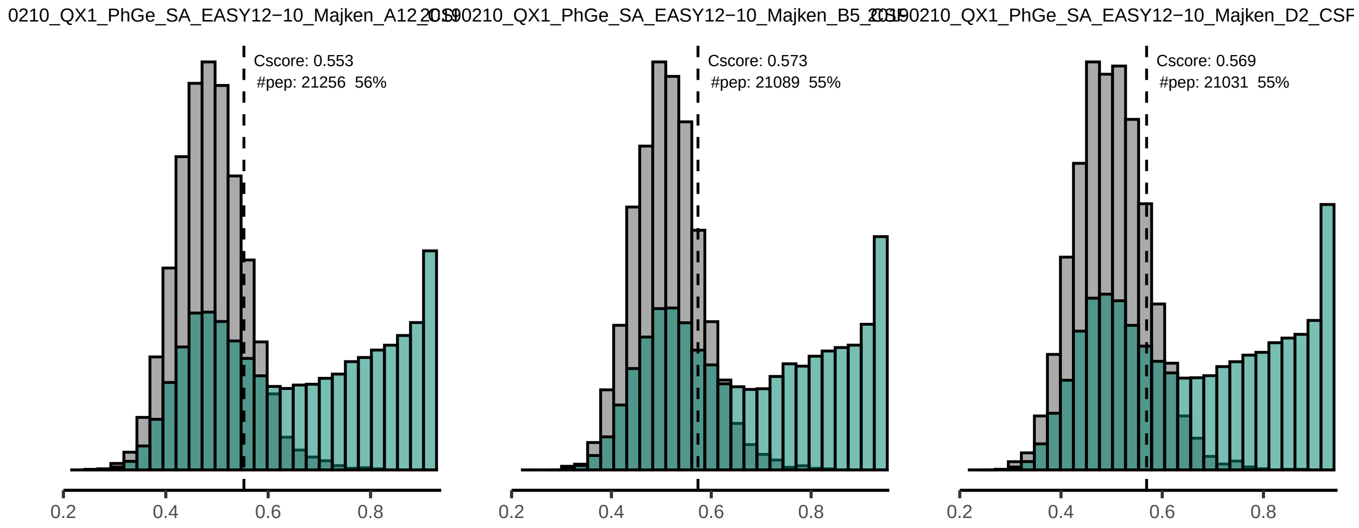

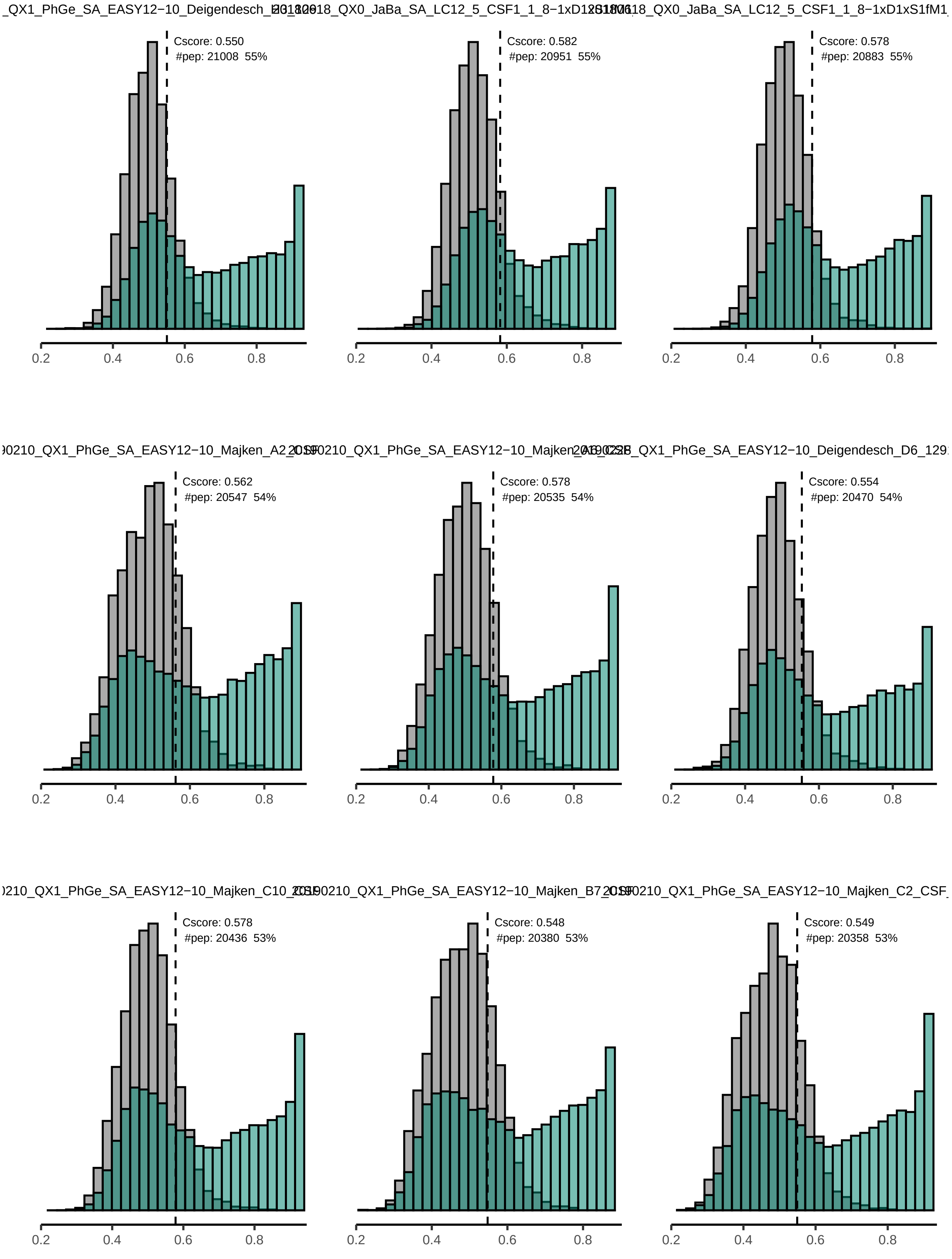

Cscore: 0.550
#pep: 21008 55%
Cscore: 0.582
#pep: 20951 55%
Cscore: 0.578
#pep: 20883 55%
Cscore: 0.562
#pep: 20547 54%
Cscore: 0.578
#pep: 20535 54%
Cscore: 0.554
#pep: 20470 54%
Cscore: 0.578
#pep: 20436 53%
Cscore: 0.548
#pep: 20380 53%
Cscore: 0.549
#pep: 20358 53%
0.2
0.4
0.6
0.8

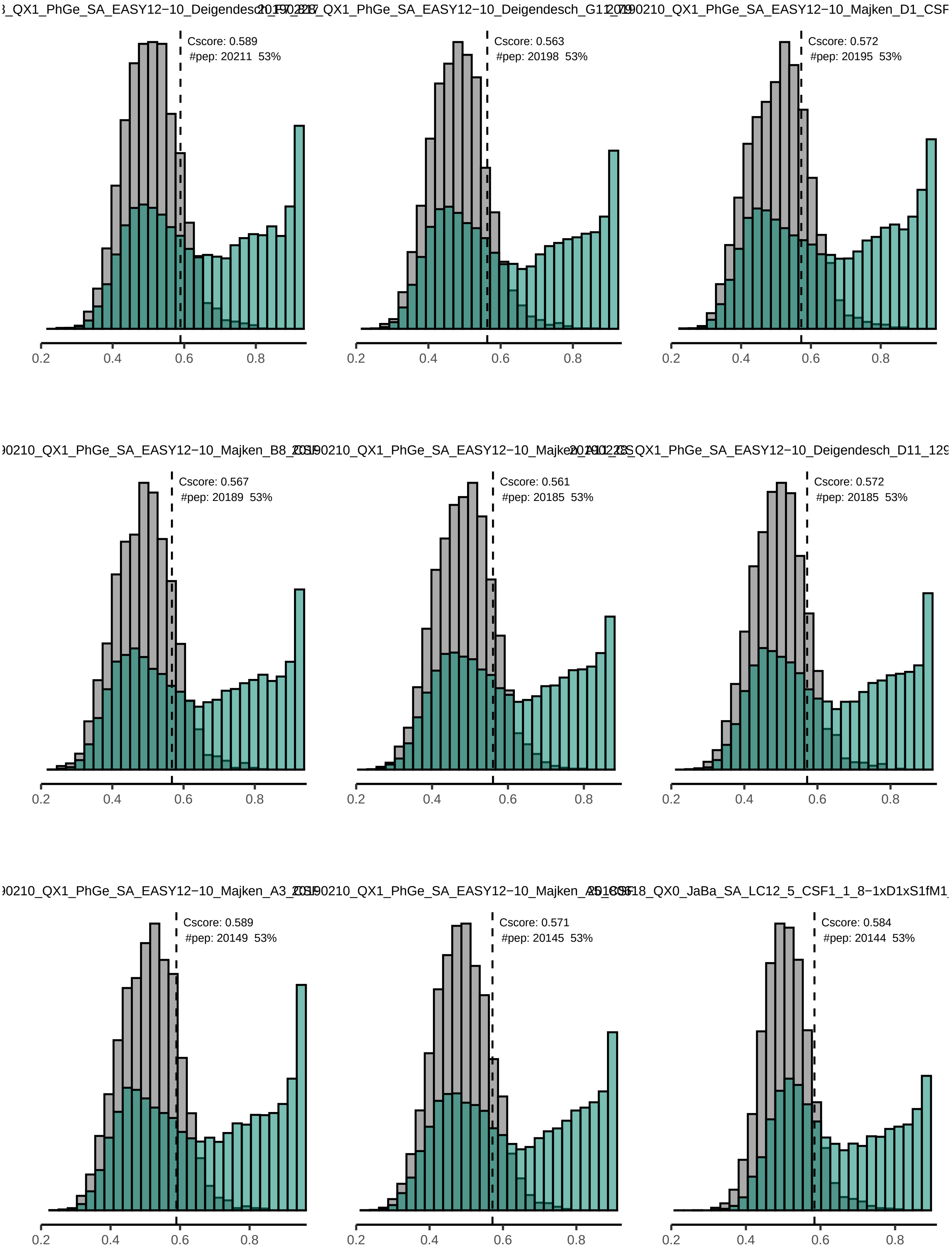
Cscore: 0.589
#pep: 20211 53%
Cscore: 0.563
#pep: 20198 53%
Cscore: 0.572
#pep: 20195 53%
Cscore: 0.567
#pep: 20189 53%
Cscore: 0.561
#pep: 20185 53%
Cscore: 0.572
#pep: 20185 53%
Cscore: 0.589
#pep: 20149 53%
Cscore: 0.571
#pep: 20145 53%
Cscore: 0.584
#pep: 20144 53%
0.2
0.4
0.6
0.8

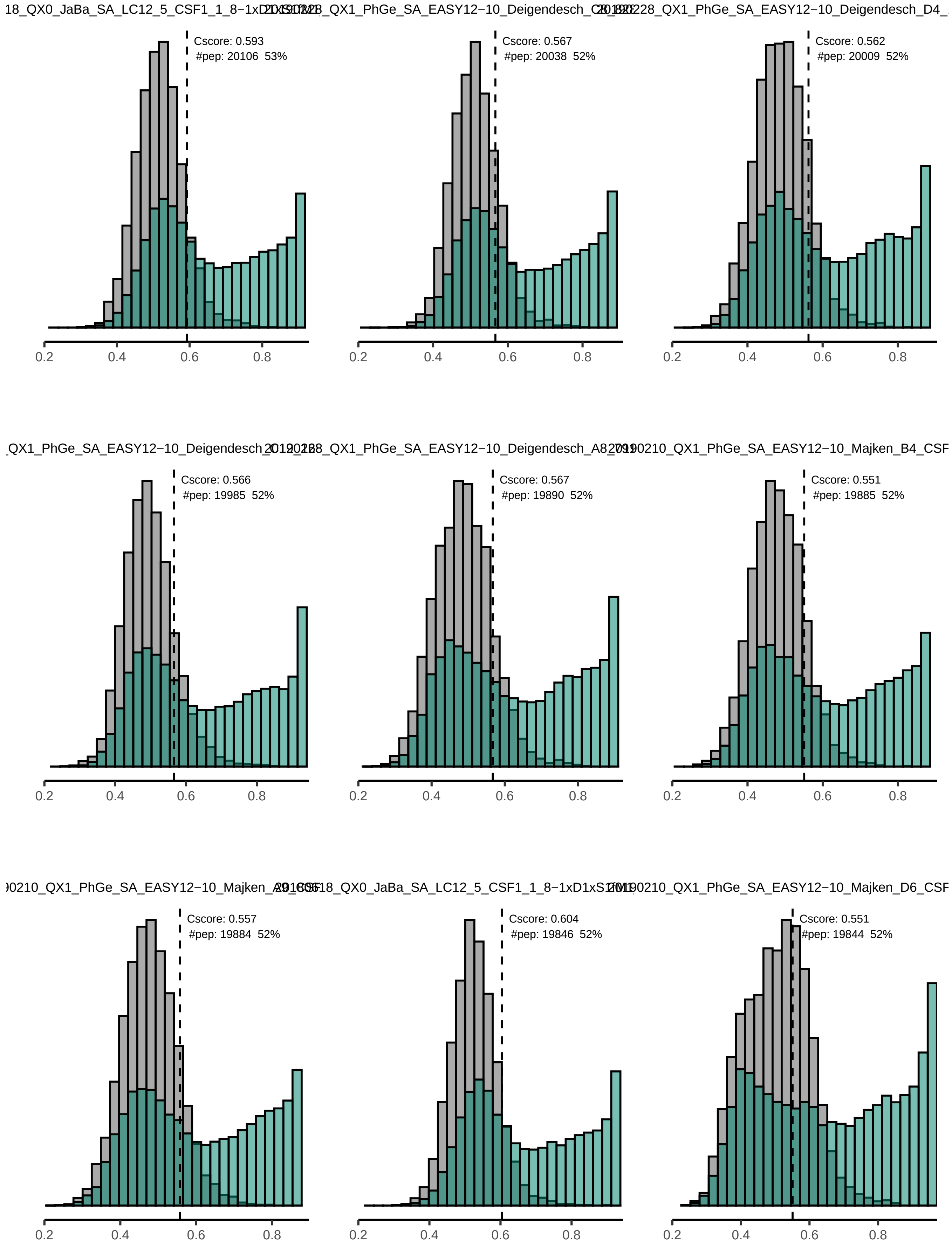
Cscore: 0.593
#pep: 20106 53%
Cscore: 0.567
#pep: 20038 52%
Cscore: 0.562
#pep: 20009 52%
Cscore: 0.566
#pep: 19985 52%
Cscore: 0.567
#pep: 19890 52%
Cscore: 0.551
#pep: 19885 52%
Cscore: 0.557
#pep: 19884 52%
Cscore: 0.604
#pep: 19846 52%
Cscore: 0.551
#pep: 19844 52%

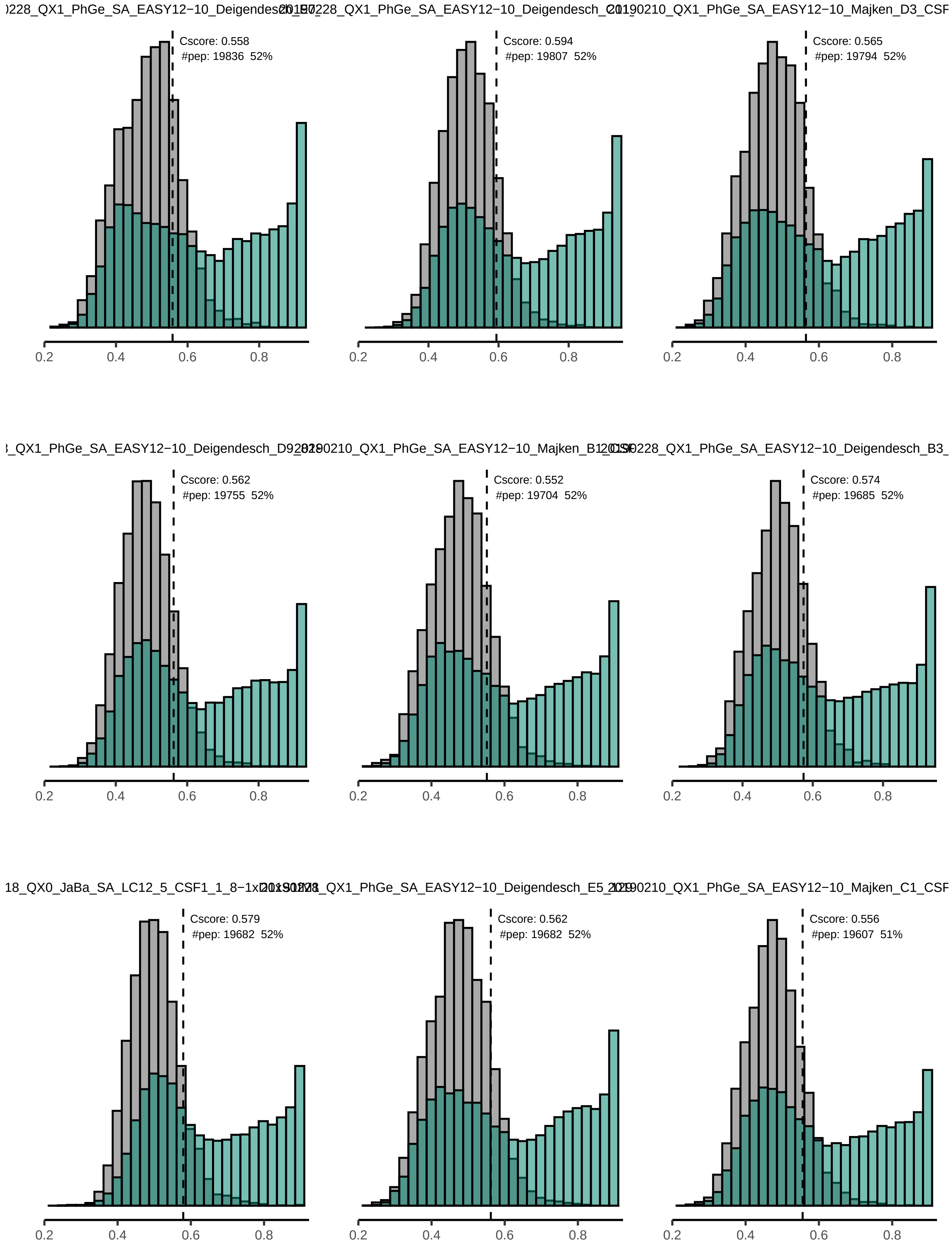
Cscore: 0.558
#pep: 19836 52%
Cscore: 0.594
#pep: 19807 52%
Cscore: 0.565
#pep: 19794 52%
Cscore: 0.562
#pep: 19755 52%
Cscore: 0.552
#pep: 19704 52%
Cscore: 0.574
#pep: 19685 52%
Cscore: 0.579
#pep: 19682 52%
Cscore: 0.562
#pep: 19682 52%
Cscore: 0.556
#pep: 19607 51%
0.2
0.4
0.6
0.8

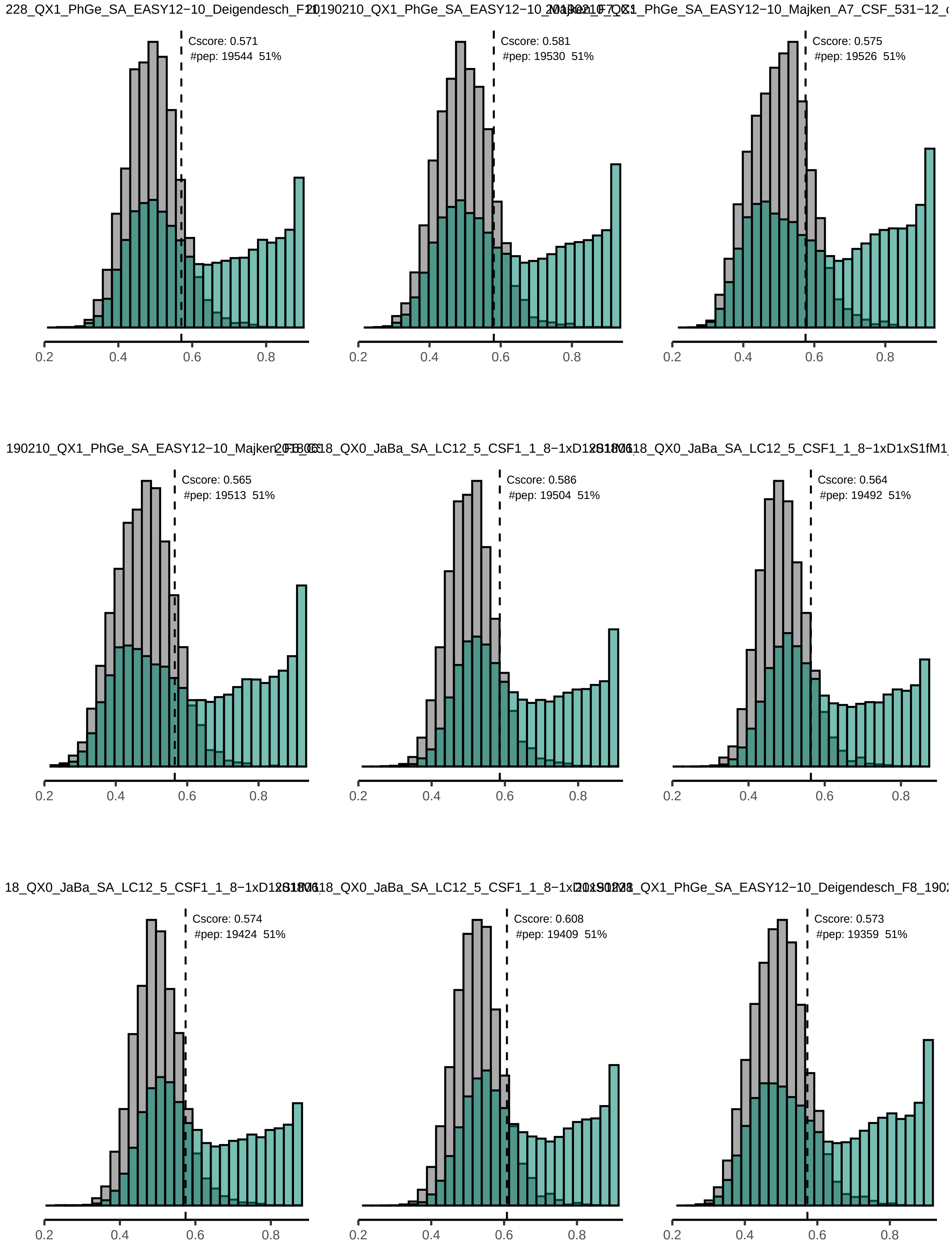
228_QX1_PhGe_SA_EASY12−10_Deigendesch_F1120190210_QX1_PhGe_SA_EASY12−10_Majken_F7_QX1_PhGe_SA_EASY12−10_Majken_A7_CSF_531−12_(
Cscore: 0.571
#pep: 19544 51%
Cscore: 0.581
#pep: 19530 51%
Cscore: 0.575
#pep: 19526 51%
0.2
0.4
0.6
0.8
190210_QX1_PhGe_SA_EASY12−10_Majken_F6_20180618_QX0_JaBa_SA_LC12_5_CSF1_1_8−1xD1xS1fM120180618_QX0_JaBa_SA_LC12_5_CSF1_1_8−1xD1xS1fM1_
Cscore: 0.565
#pep: 19513 51%
Cscore: 0.586
#pep: 19504 51%
Cscore: 0.564
#pep: 19492 51%
18_QX0_JaBa_SA_LC12_5_CSF1_1_8−1xD1xS1fM120180618_QX0_JaBa_SA_LC12_5_CSF1_1_8−1xD120190228_QX1_PhGe_SA_EASY12−10_Deigendesch_F8_190:
Cscore: 0.574
#pep: 19424 51%
Cscore: 0.608
#pep: 19409 51%
Cscore: 0.573
#pep: 19359 51%

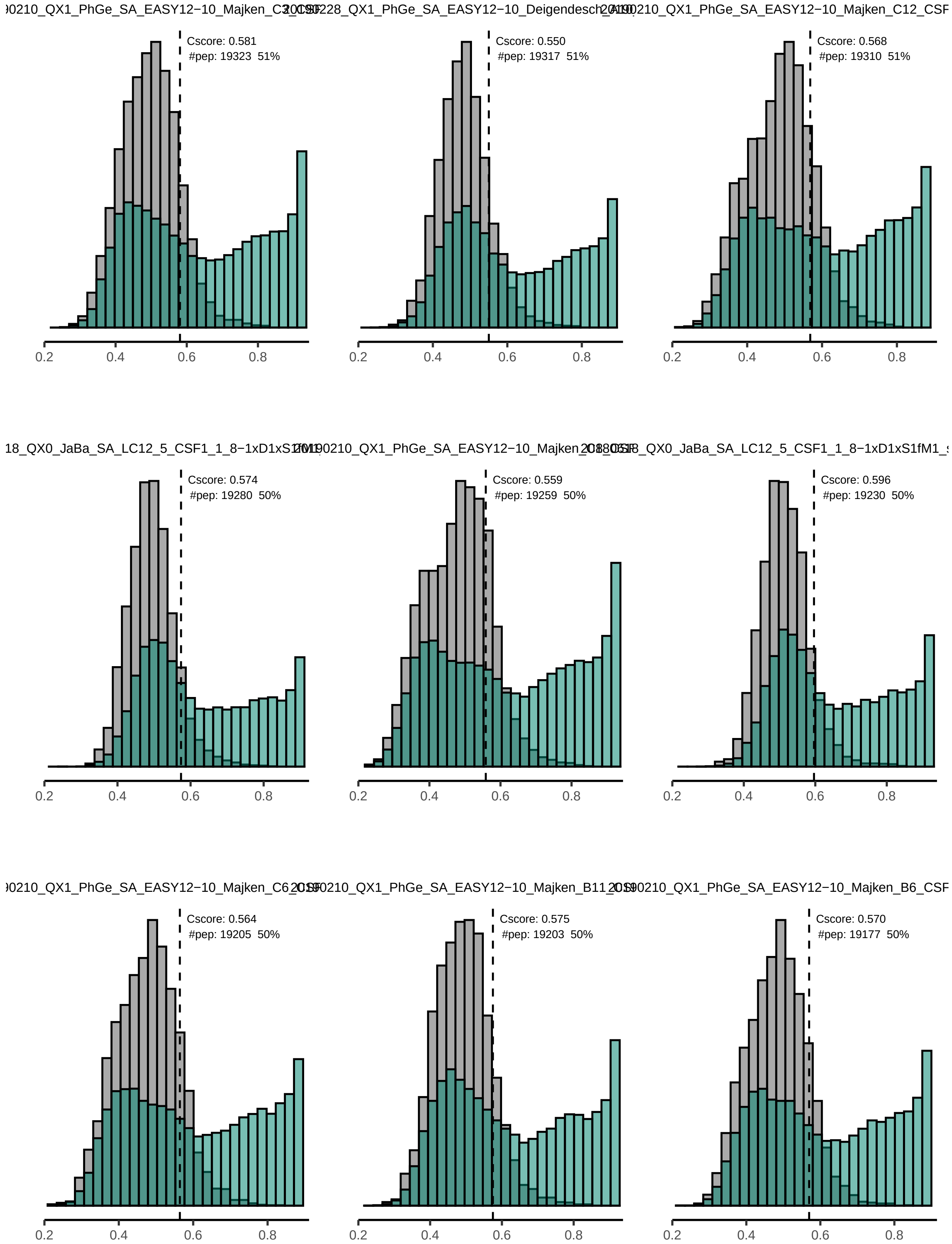
Cscore: 0.581
#pep: 19323 51%
Cscore: 0.550
#pep: 19317 51%
Cscore: 0.568
#pep: 19310 51%
Cscore: 0.574
#pep: 19280 50%
Cscore: 0.559
#pep: 19259 50%
Cscore: 0.596
#pep: 19230 50%
Cscore: 0.564
#pep: 19205 50%
Cscore: 0.575
#pep: 19203 50%
Cscore: 0.570
#pep: 19177 50%
0.2
0.4
0.6
0.8

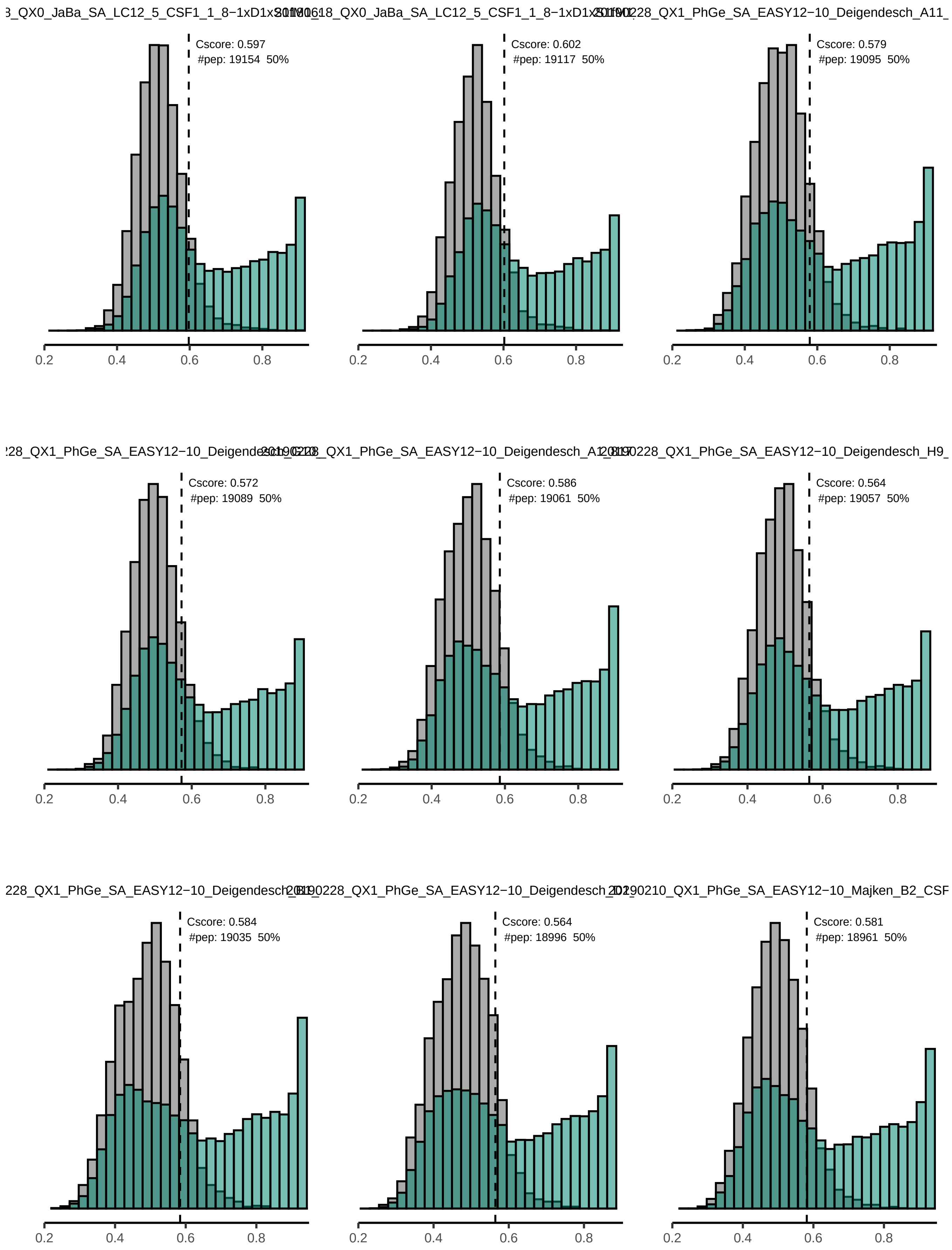

Cscore: 0.597
#pep: 19154 50%
Cscore: 0.602
#pep: 19117 50%
Cscore: 0.579
#pep: 19095 50%
Cscore: 0.572
#pep: 19089 50%
Cscore: 0.586
#pep: 19061 50%
Cscore: 0.564
#pep: 19057 50%
Cscore: 0.584
#pep: 19035 50%
Cscore: 0.564
#pep: 18996 50%
Cscore: 0.581
#pep: 18961 50%
0.2
0.4
0.6
0.8

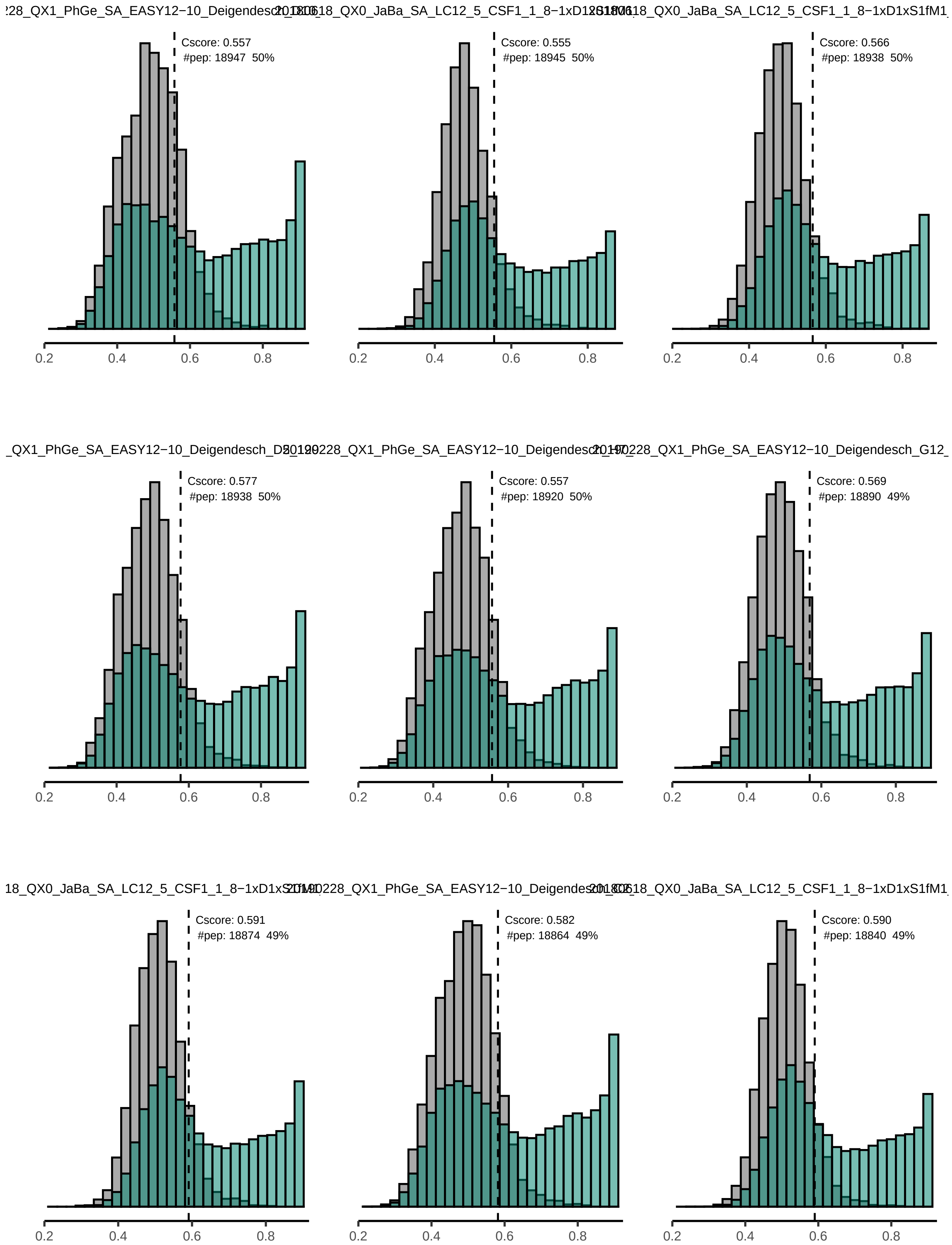
Cscore: 0.557
#pep: 18947 50%
Cscore: 0.555
#pep: 18945 50%
Cscore: 0.566
#pep: 18938 50%
Cscore: 0.577
#pep: 18938 50%
Cscore: 0.557
#pep: 18920 50%
Cscore: 0.569
#pep: 18890 49%
Cscore: 0.591
#pep: 18874 49%
Cscore: 0.582
#pep: 18864 49%
Cscore: 0.590
#pep: 18840 49%
0.2
0.4
0.6
0.8

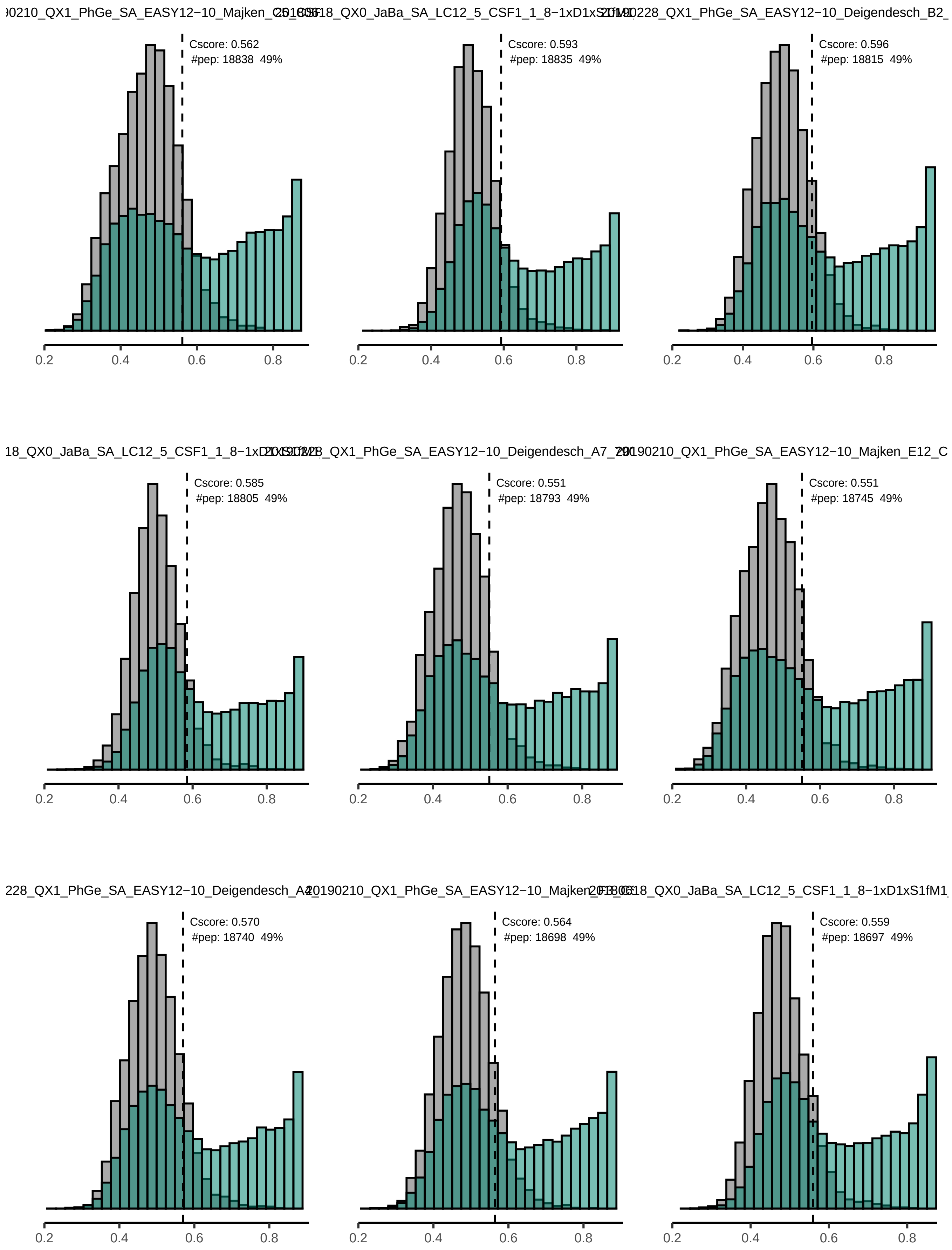

Cscore: 0.562
#pep: 18838 49%
Cscore: 0.593
#pep: 18835 49%
Cscore: 0.596
#pep: 18815 49%
Cscore: 0.585
#pep: 18805 49%
Cscore: 0.551
#pep: 18793 49%
Cscore: 0.551
#pep: 18745 49%
Cscore: 0.570
#pep: 18740 49%
Cscore: 0.564
#pep: 18698 49%
Cscore: 0.559
#pep: 18697 49%
0.2
0.4
0.6
0.8

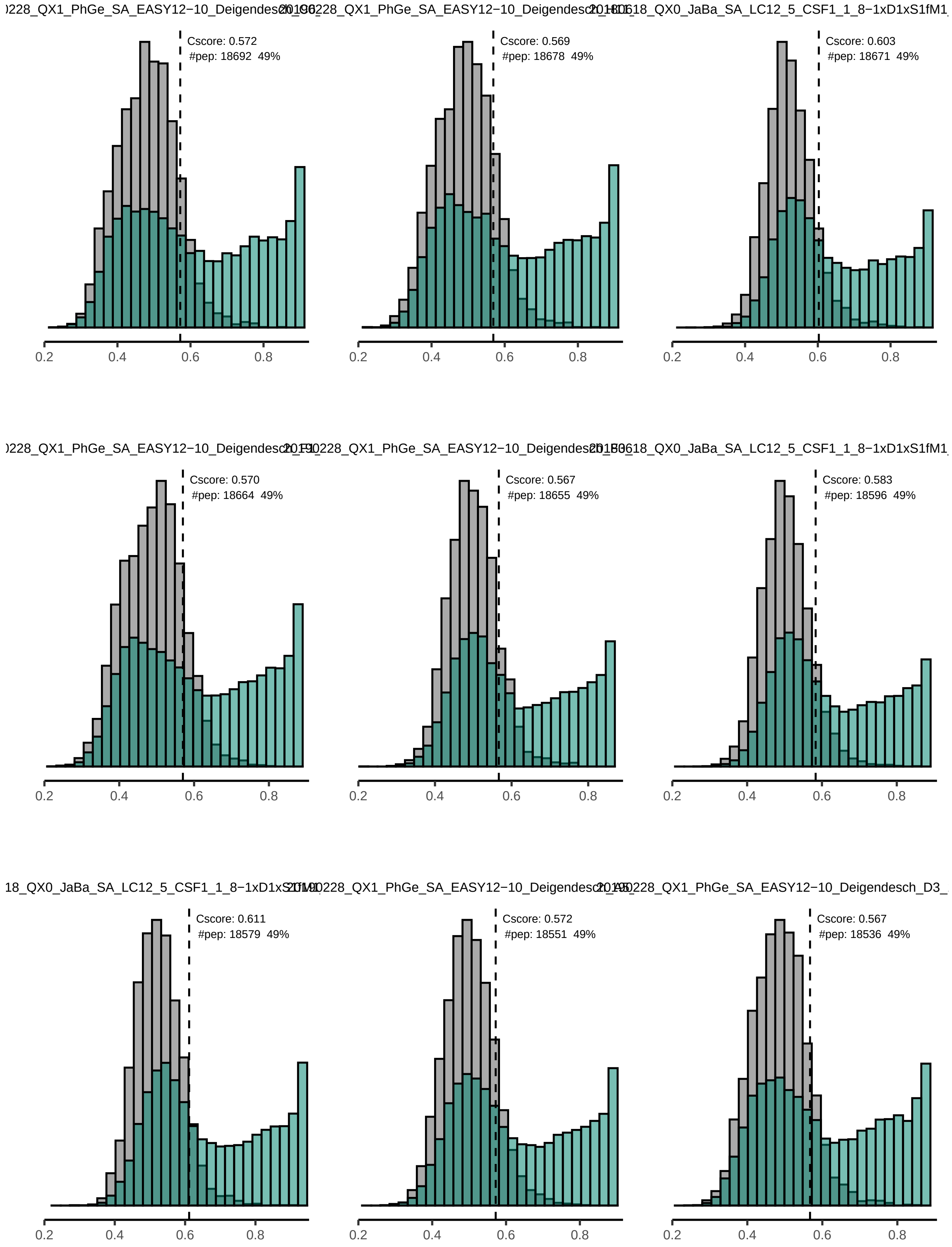
Cscore: 0.572
#pep: 18692 49%
Cscore: 0.569
#pep: 18678 49%
Cscore: 0.603
#pep: 18671 49%
Cscore: 0.570
#pep: 18664 49%
Cscore: 0.567
#pep: 18655 49%
Cscore: 0.583
#pep: 18596 49%
Cscore: 0.611
#pep: 18579 49%
Cscore: 0.572
#pep: 18551 49%
Cscore: 0.567
#pep: 18536 49%
0.2
0.4
0.6
0.8

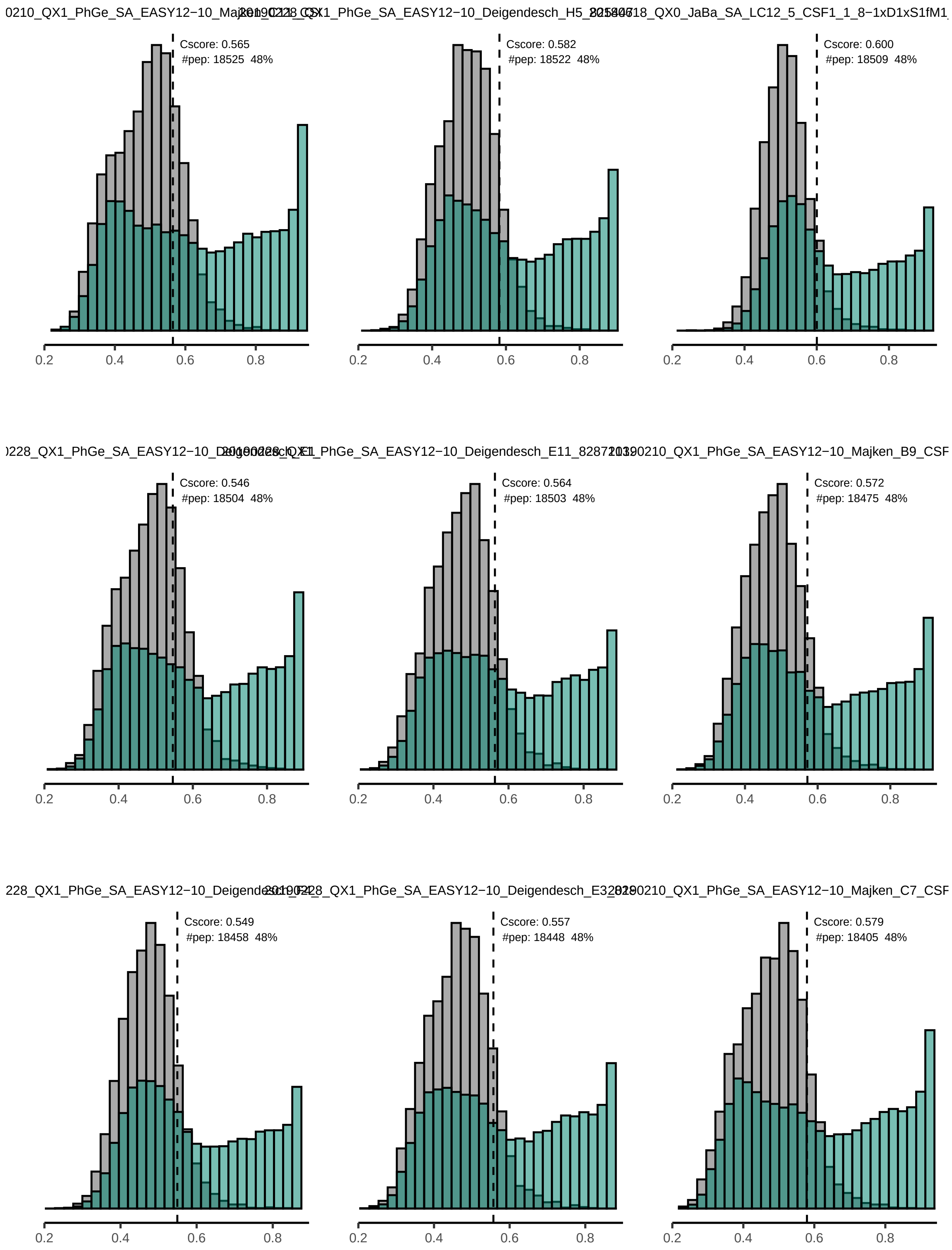

Cscore: 0.565
#pep: 18525 48%
Cscore: 0.582
#pep: 18522 48%
Cscore: 0.600
#pep: 18509 48%
Cscore: 0.546
#pep: 18504 48%
Cscore: 0.564
#pep: 18503 48%
Cscore: 0.572
#pep: 18475 48%
Cscore: 0.549
#pep: 18458 48%
Cscore: 0.557
#pep: 18448 48%
Cscore: 0.579
#pep: 18405 48%
0.2
0.4
0.6
0.8

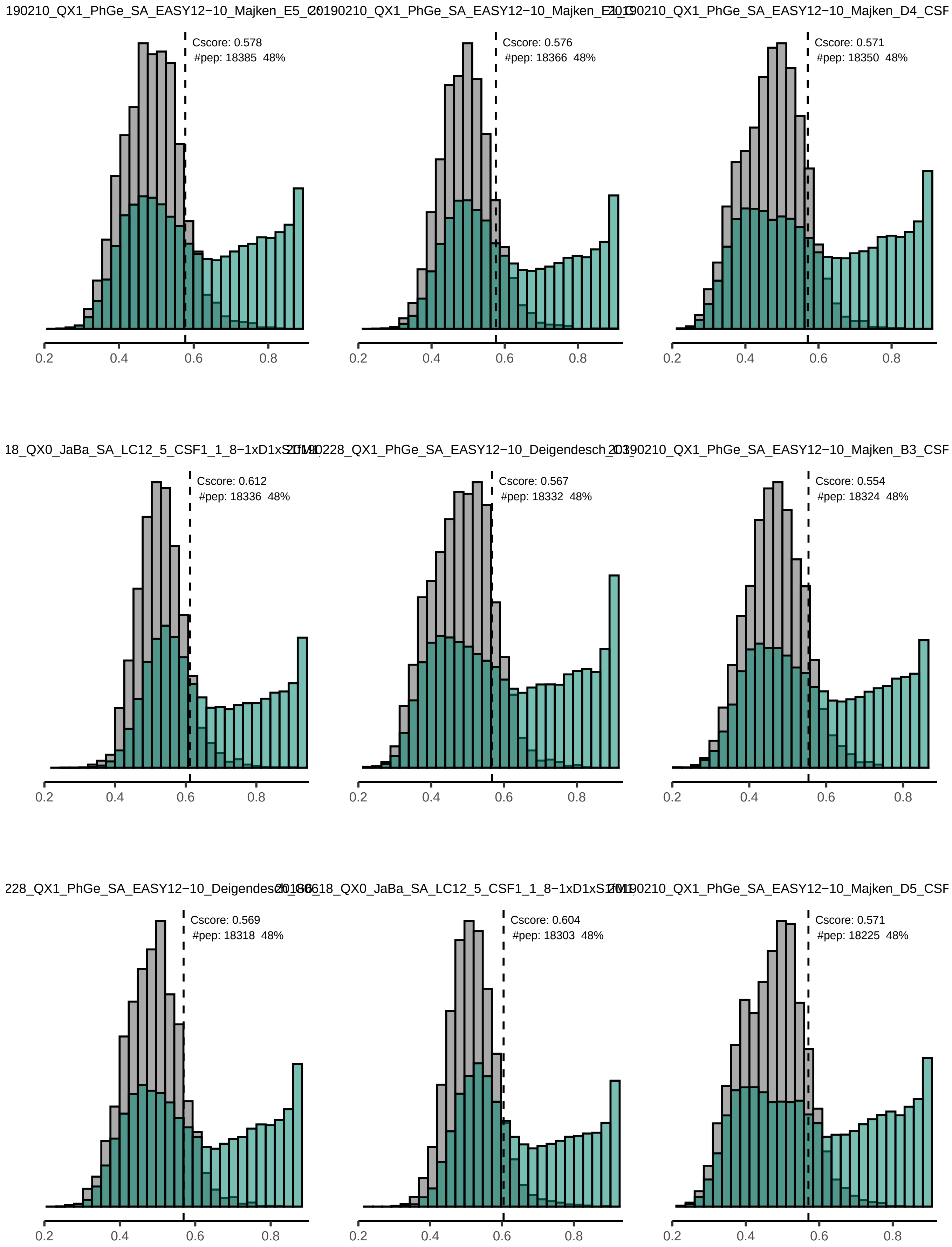
190210_QX1_PhGe_SA_EASY12−10_Majken_E5_C
20190210_QX1_PhGe_SA_EASY12−10_Majken_E1_C
20190210_QX1_PhGe_SA_EASY12−10_Majken_D4_CSF
Cscore: 0.578
#pep: 18385 48%
Cscore: 0.576
#pep: 18366 48%
Cscore: 0.571
#pep: 18350 48%
18_QX0_JaBa_SA_LC12_5_CSF1_1_8−1xD1xS1fM1
20190228_QX1_PhGe_SA_EASY12−10_Deigendesch_03
20190210_QX1_PhGe_SA_EASY12−10_Majken_B3_CSF
Cscore: 0.612
#pep: 18336 48%
Cscore: 0.567
#pep: 18332 48%
Cscore: 0.554
#pep: 18324 48%
228_QX1_PhGe_SA_EASY12−10_Deigendesch_06
20180618_QX0_JaBa_SA_LC12_5_CSF1_1_8−1xD1xS1fM1
20190210_QX1_PhGe_SA_EASY12−10_Majken_D5_CSF
Cscore: 0.569
#pep: 18318 48%
Cscore: 0.604
#pep: 18303 48%
Cscore: 0.571
#pep: 18225 48%
0.2
0.4
0.6
0.8

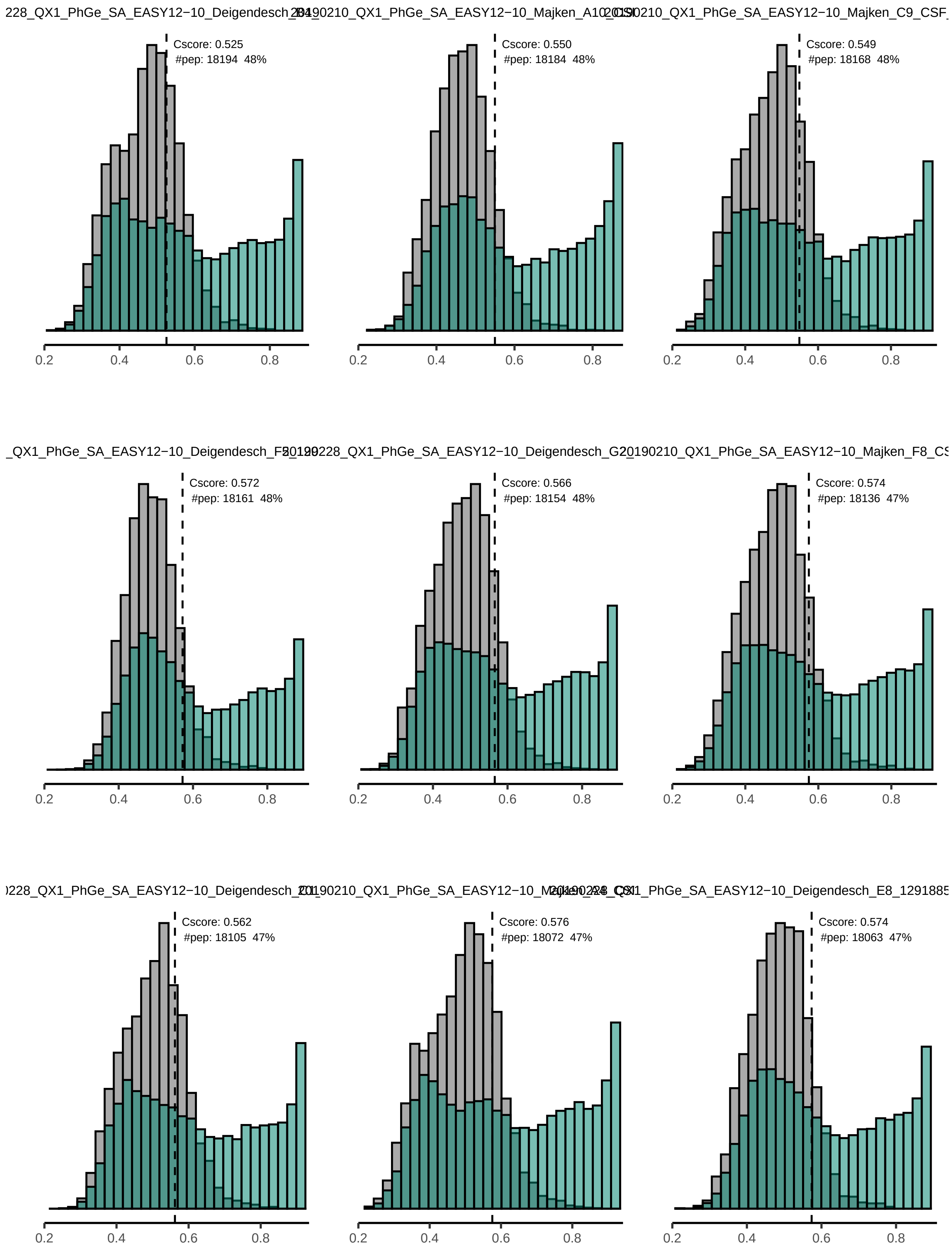
Cscore: 0.525
#pep: 18194 48%
Cscore: 0.550
#pep: 18184 48%
Cscore: 0.549
#pep: 18168 48%
Cscore: 0.572
#pep: 18161 48%
Cscore: 0.566
#pep: 18154 48%
Cscore: 0.574
#pep: 18136 47%
Cscore: 0.562
#pep: 18105 47%
Cscore: 0.576
#pep: 18072 47%
Cscore: 0.574
#pep: 18063 47%
0.2
0.4
0.6
0.8

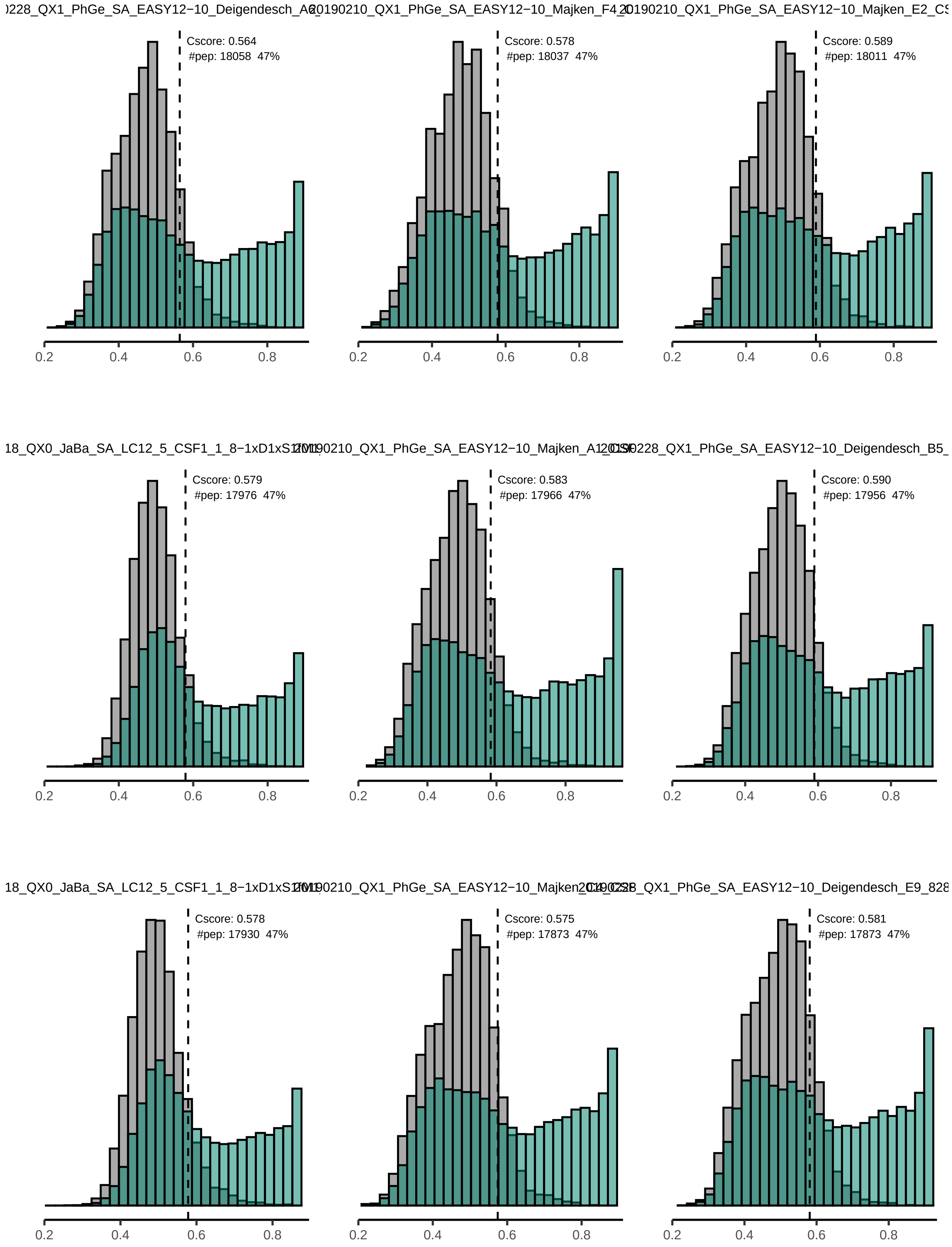
)228_QX1_PhGe_SA_EASY12−10_Deigendesch_A6
20190210_QX1_PhGe_SA_EASY12−10_Majken_F4
20190210_QX1_PhGe_SA_EASY12−10_Majken_E2_CS
Cscore: 0.564
#pep: 18058 47%
Cscore: 0.578
#pep: 18037 47%
Cscore: 0.589
#pep: 18011 47%
0.2
0.4
0.6
0.8
18_QX0_JaBa_SA_LC12_5_CSF1_1_8−1xD1xS1
20190210_QX1_PhGe_SA_EASY12−10_Majken_A1_CSF
20190228_QX1_PhGe_SA_EASY12−10_Deigendesch_B5_
Cscore: 0.579
#pep: 17976 47%
Cscore: 0.583
#pep: 17966 47%
Cscore: 0.590
#pep: 17956 47%
18_QX0_JaBa_SA_LC12_5_CSF1_1_8−1xD1xS1
20190210_QX1_PhGe_SA_EASY12−10_Majken_C1
20190228_QX1_PhGe_SA_EASY12−10_Deigendesch_E9_828
Cscore: 0.578
#pep: 17930 47%
Cscore: 0.575
#pep: 17873 47%
Cscore: 0.581
#pep: 17873 47%

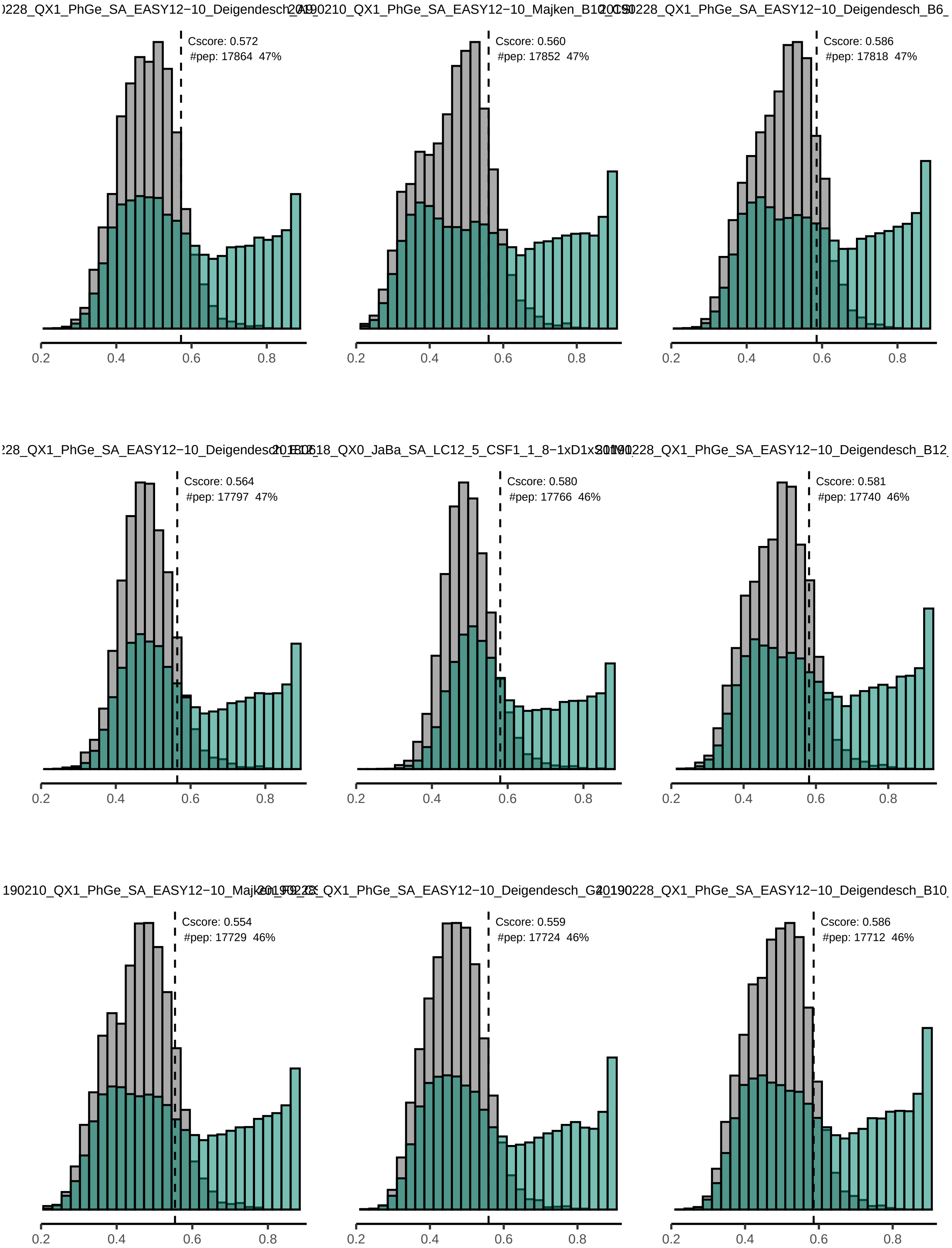
)228_QX1_PhGe_SA_EASY12−10_Deigendesch_A9
20190210_QX1_PhGe_SA_EASY12−10_Majken_B12_C9
20190228_QX1_PhGe_SA_EASY12−10_Deigendesch_B6_
Cscore: 0.572
#pep: 17864 47%
Cscore: 0.560
#pep: 17852 47%
Cscore: 0.586
#pep: 17818 47%
Cscore: 0.564
#pep: 17797 47%
Cscore: 0.580
#pep: 17766 46%
Cscore: 0.581
#pep: 17740 46%
Cscore: 0.554
#pep: 17729 46%
Cscore: 0.559
#pep: 17724 46%
Cscore: 0.586
#pep: 17712 46%
0.2
0.4
0.6
0.8

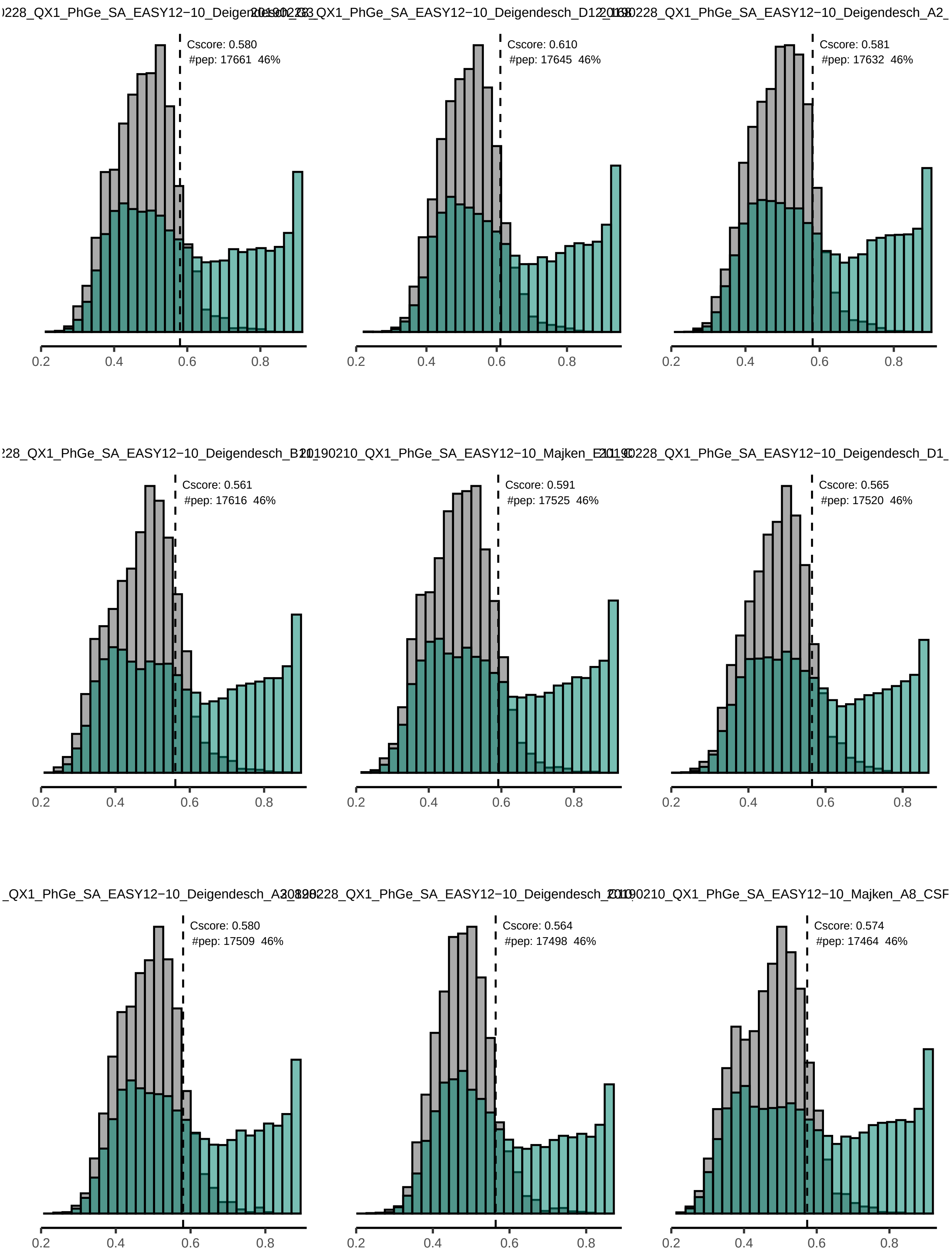
Cscore: 0.580
#pep: 17661 46%
Cscore: 0.610
#pep: 17645 46%
Cscore: 0.581
#pep: 17632 46%
Cscore: 0.561
#pep: 17616 46%
Cscore: 0.591
#pep: 17525 46%
Cscore: 0.565
#pep: 17520 46%
Cscore: 0.580
#pep: 17509 46%
Cscore: 0.564
#pep: 17498 46%
Cscore: 0.574
#pep: 17464 46%
0.2
0.4
0.6
0.8

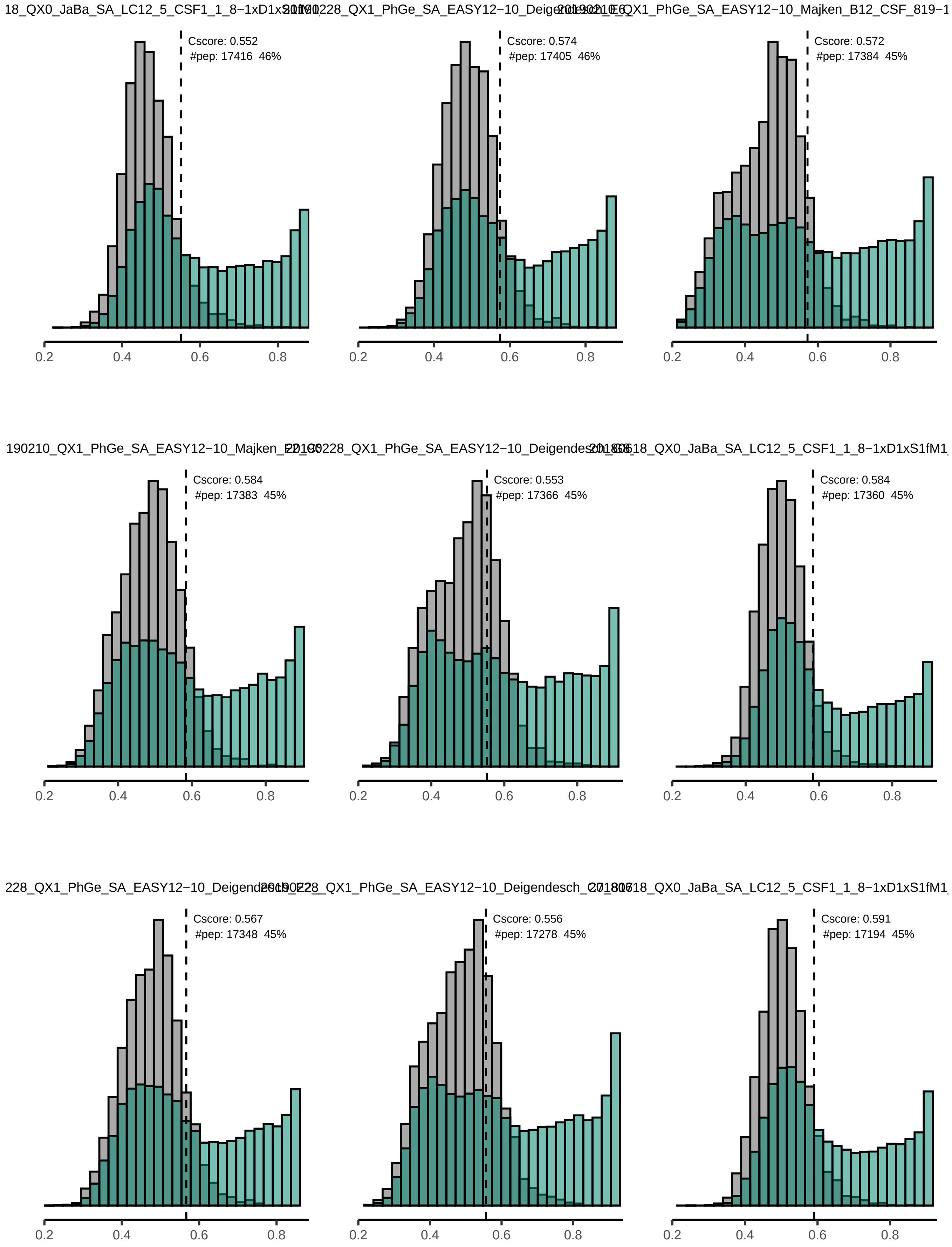
18_QX0_JaBa_SA_LC12_5_CSF1_1_8−1xD1xS1fM1
20190228_QX1_PhGe_SA_EASY12−10_Deigendesch_E6
20190210_QX1_PhGe_SA_EASY12−10_Majken_B12_CSF_819−1
Cscore: 0.552
#pep: 17416 46%
Cscore: 0.574
#pep: 17405 46%
Cscore: 0.572
#pep: 17384 45%
190210_QX1_PhGe_SA_EASY12−10_Majken_E2
20190228_QX1_PhGe_SA_EASY12−10_Deigendesch_E6
20180618_QX0_JaBa_SA_LC12_5_CSF1_1_8−1xD1xS1fM1
Cscore: 0.584
#pep: 17383 45%
Cscore: 0.553
#pep: 17366 45%
Cscore: 0.584
#pep: 17360 45%
228_QX1_PhGe_SA_EASY12−10_Deigendesch_E2
20190228_QX1_PhGe_SA_EASY12−10_Deigendesch_C7
20180618_QX0_JaBa_SA_LC12_5_CSF1_1_8−1xD1xS1fM1
Cscore: 0.567
#pep: 17348 45%
Cscore: 0.556
#pep: 17278 45%
Cscore: 0.591
#pep: 17194 45%
0.2
0.4
0.6
0.8

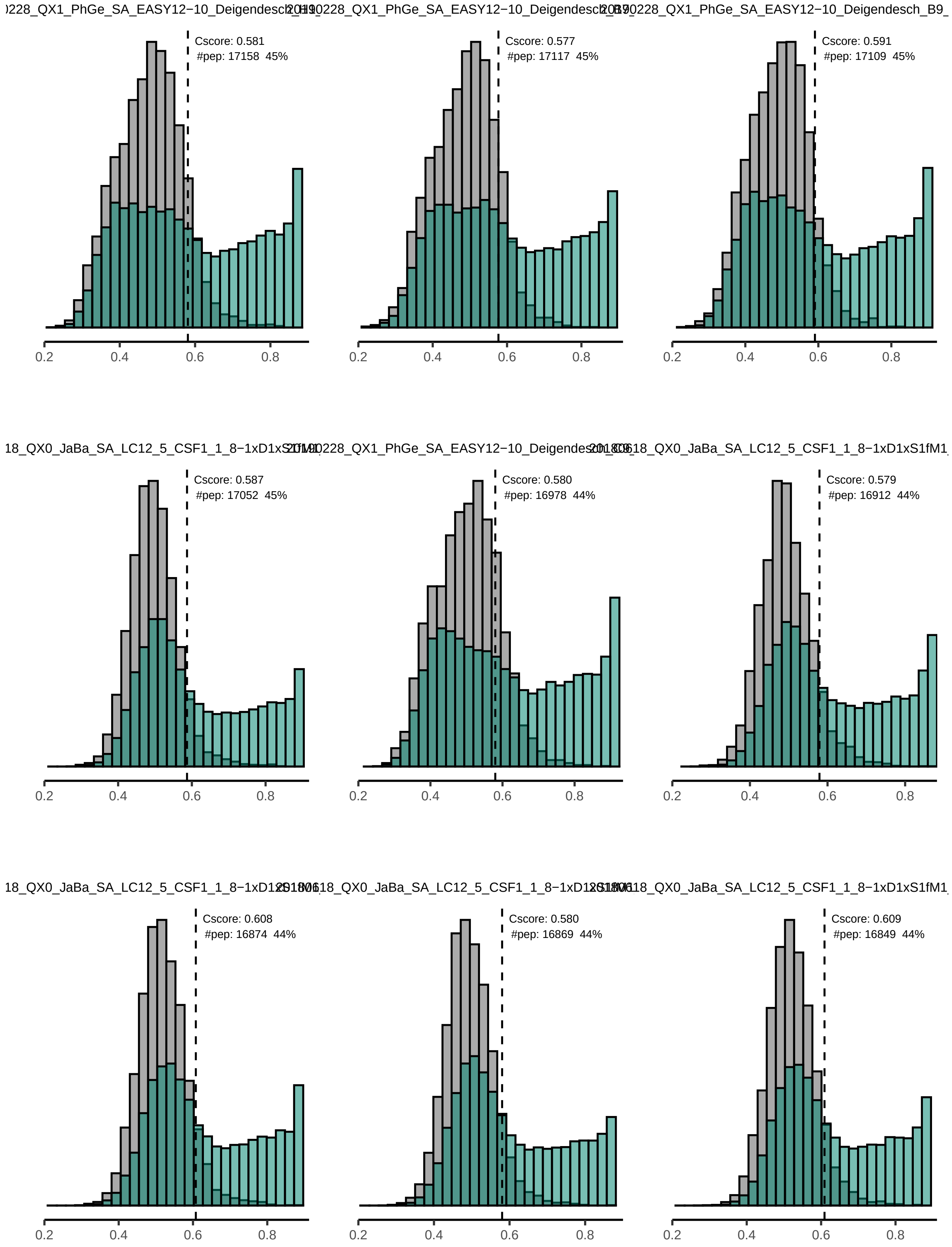
Cscore: 0.581
#pep: 17158 45%
Cscore: 0.577
#pep: 17117 45%
Cscore: 0.591
#pep: 17109 45%
Cscore: 0.587
#pep: 17052 45%
Cscore: 0.580
#pep: 16978 44%
Cscore: 0.579
#pep: 16912 44%
Cscore: 0.608
#pep: 16874 44%
Cscore: 0.580
#pep: 16869 44%
Cscore: 0.609
#pep: 16849 44%
0.2
0.4
0.6
0.8

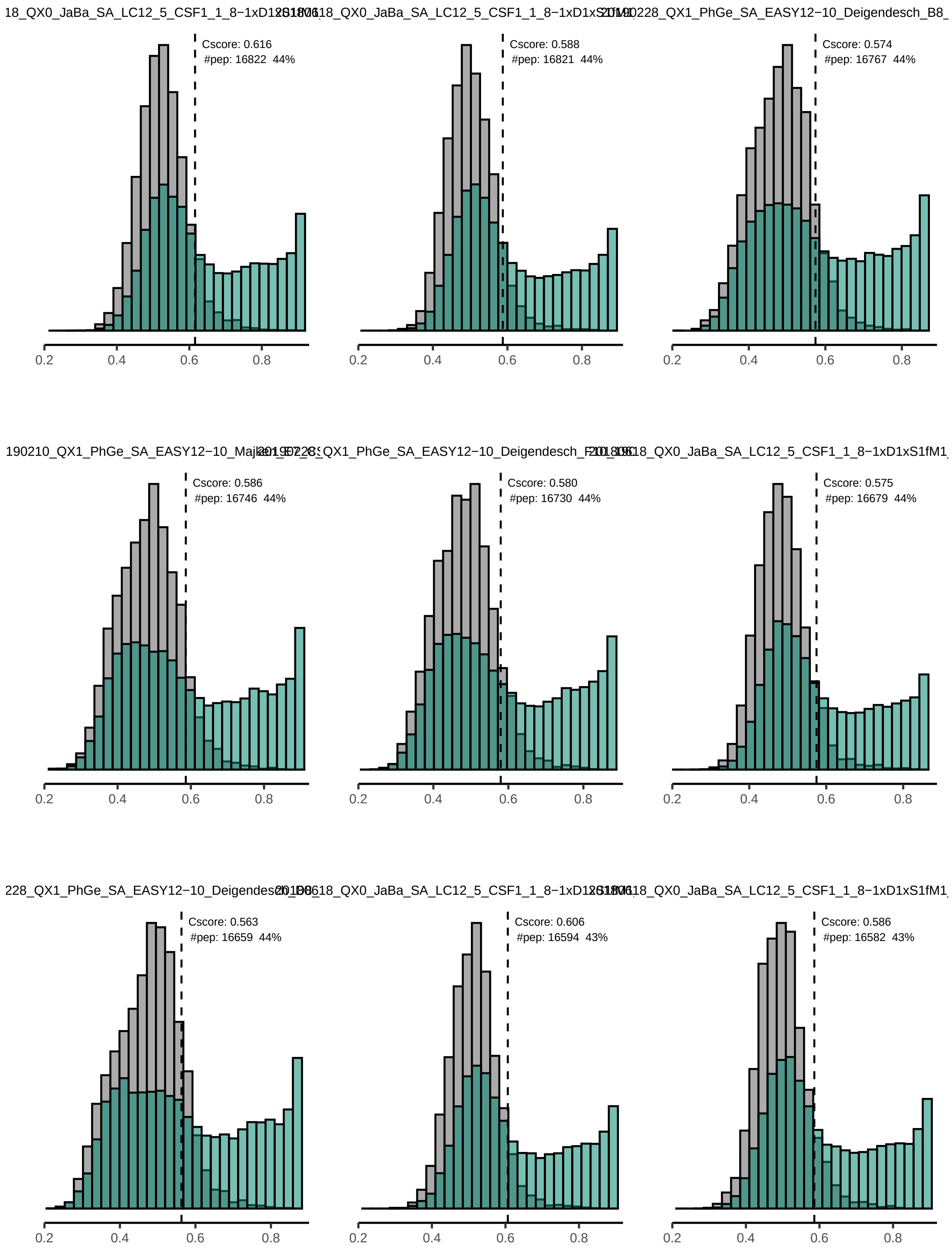
Cscore: 0.616
#pep: 16822 44%
Cscore: 0.588
#pep: 16821 44%
Cscore: 0.574
#pep: 16767 44%
Cscore: 0.586
#pep: 16746 44%
Cscore: 0.580
#pep: 16730 44%
Cscore: 0.575
#pep: 16679 44%
Cscore: 0.563
#pep: 16659 44%
Cscore: 0.606
#pep: 16594 43%
Cscore: 0.586
#pep: 16582 43%
0.2
0.4
0.6
0.8

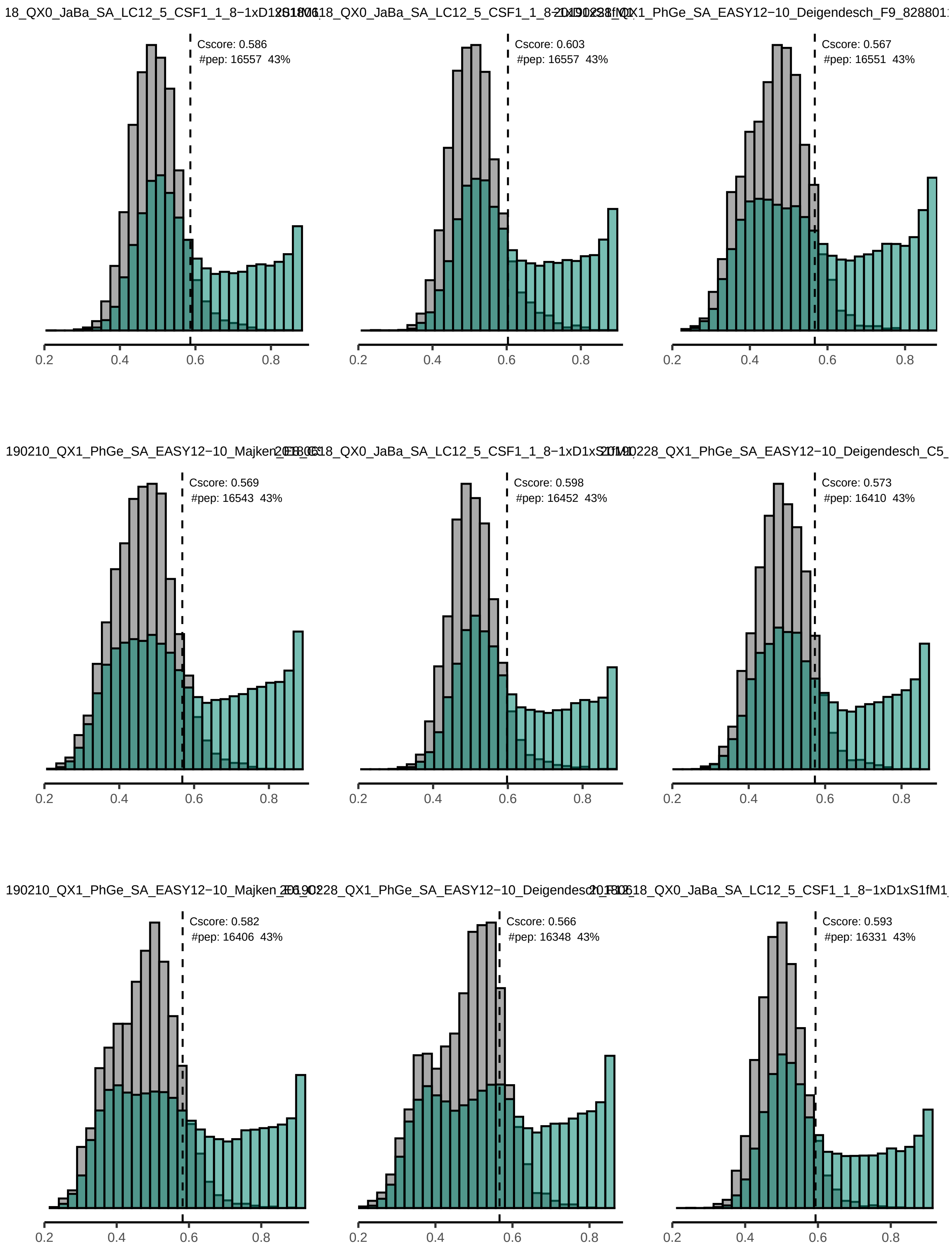
Cscore: 0.586
#pep: 16557 43%
Cscore: 0.603
#pep: 16557 43%
Cscore: 0.567
#pep: 16551 43%
Cscore: 0.569
#pep: 16543 43%
Cscore: 0.598
#pep: 16452 43%
Cscore: 0.573
#pep: 16410 43%
Cscore: 0.582
#pep: 16406 43%
Cscore: 0.566
#pep: 16348 43%
Cscore: 0.593
#pep: 16331 43%

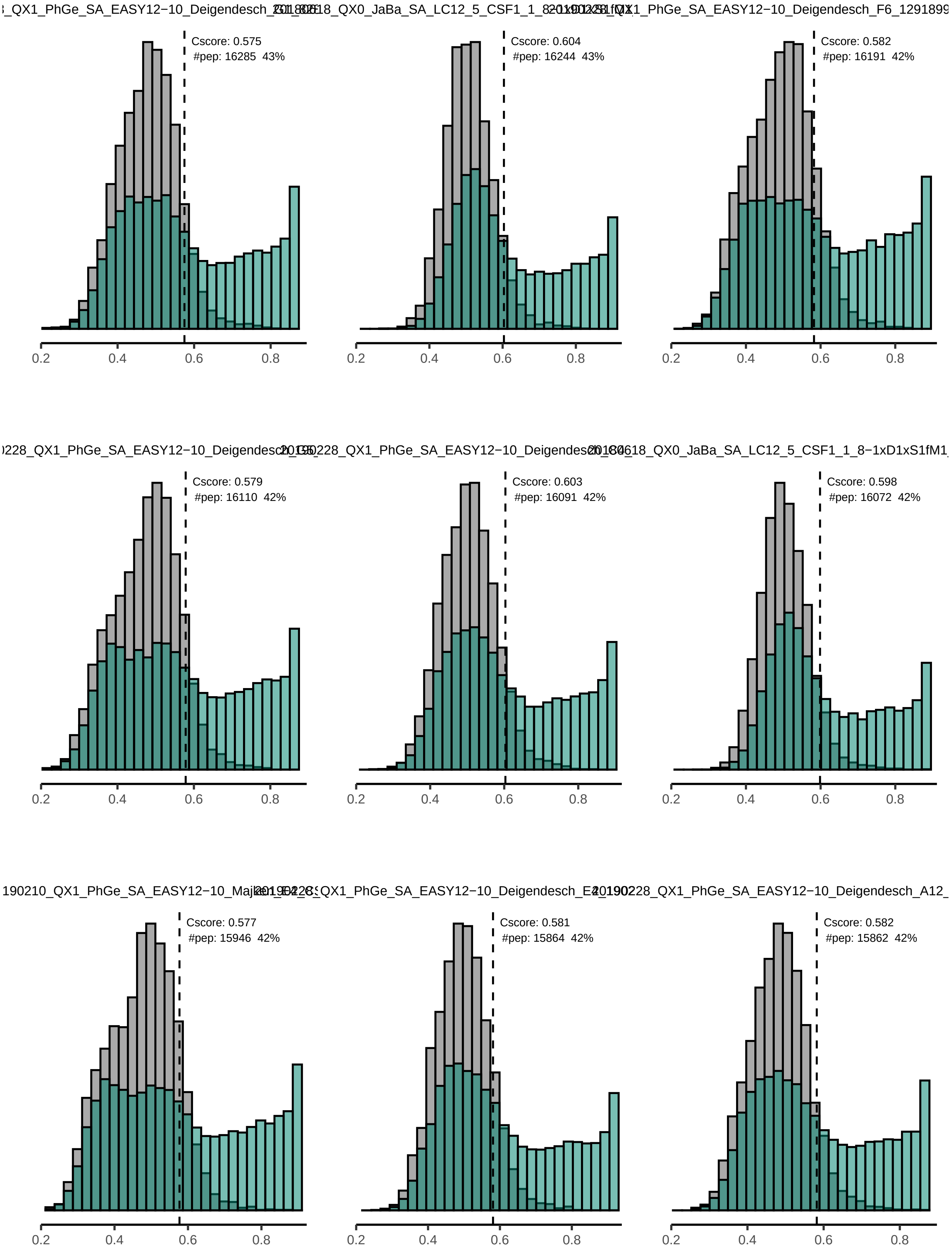
Cscore: 0.575
#pep: 16285 43%
Cscore: 0.604
#pep: 16244 43%
Cscore: 0.582
#pep: 16191 42%
Cscore: 0.579
#pep: 16110 42%
Cscore: 0.603
#pep: 16091 42%
Cscore: 0.598
#pep: 16072 42%
Cscore: 0.577
#pep: 15946 42%
Cscore: 0.581
#pep: 15864 42%
Cscore: 0.582
#pep: 15862 42%
0.2
0.4
0.6
0.8

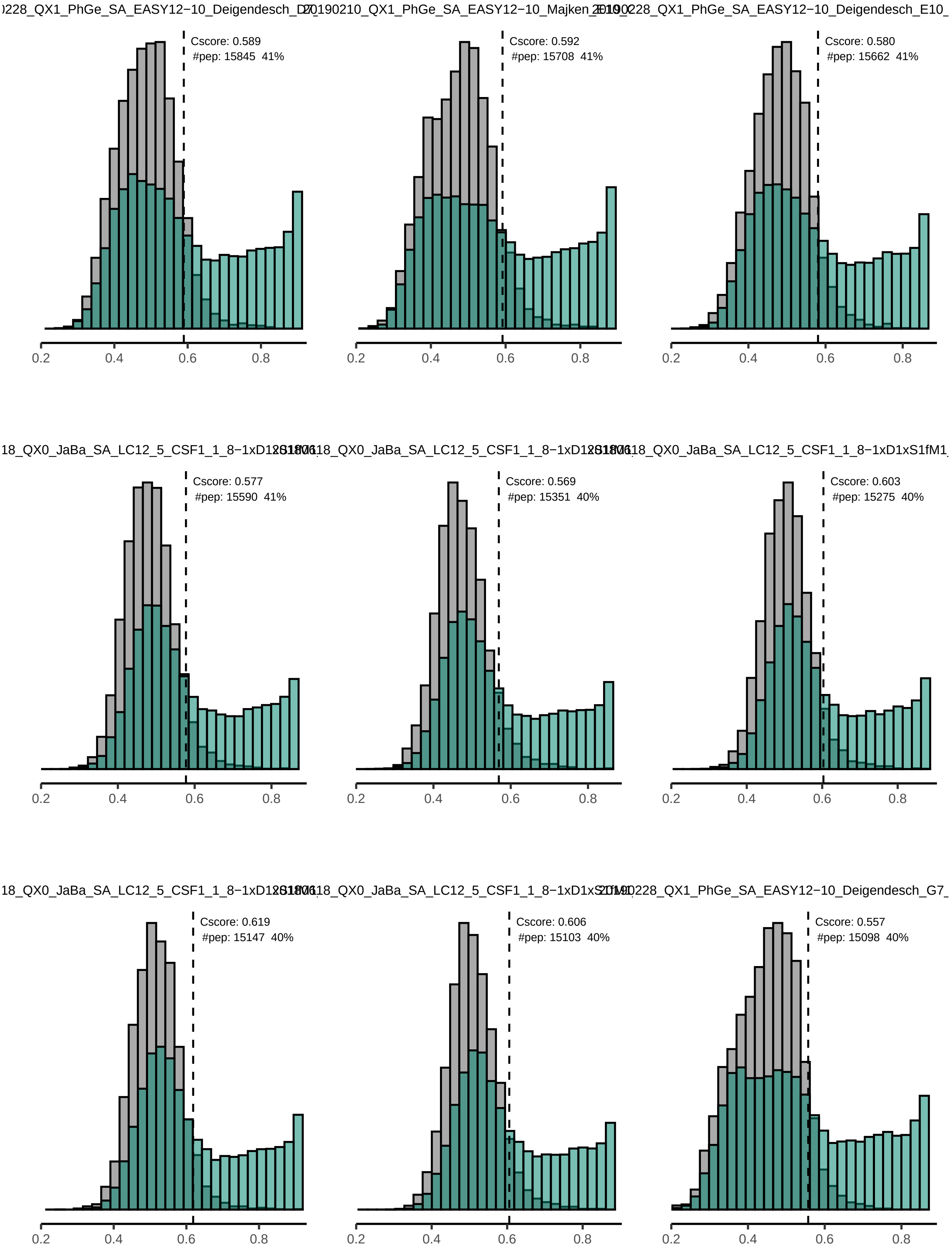
Cscore: 0.589
#pep: 15845 41%
Cscore: 0.592
#pep: 15708 41%
Cscore: 0.580
#pep: 15662 41%
Cscore: 0.577
#pep: 15590 41%
Cscore: 0.569
#pep: 15351 40%
Cscore: 0.603
#pep: 15275 40%
Cscore: 0.619
#pep: 15147 40%
Cscore: 0.606
#pep: 15103 40%
Cscore: 0.557
#pep: 15098 40%
0.2
0.4
0.6
0.8

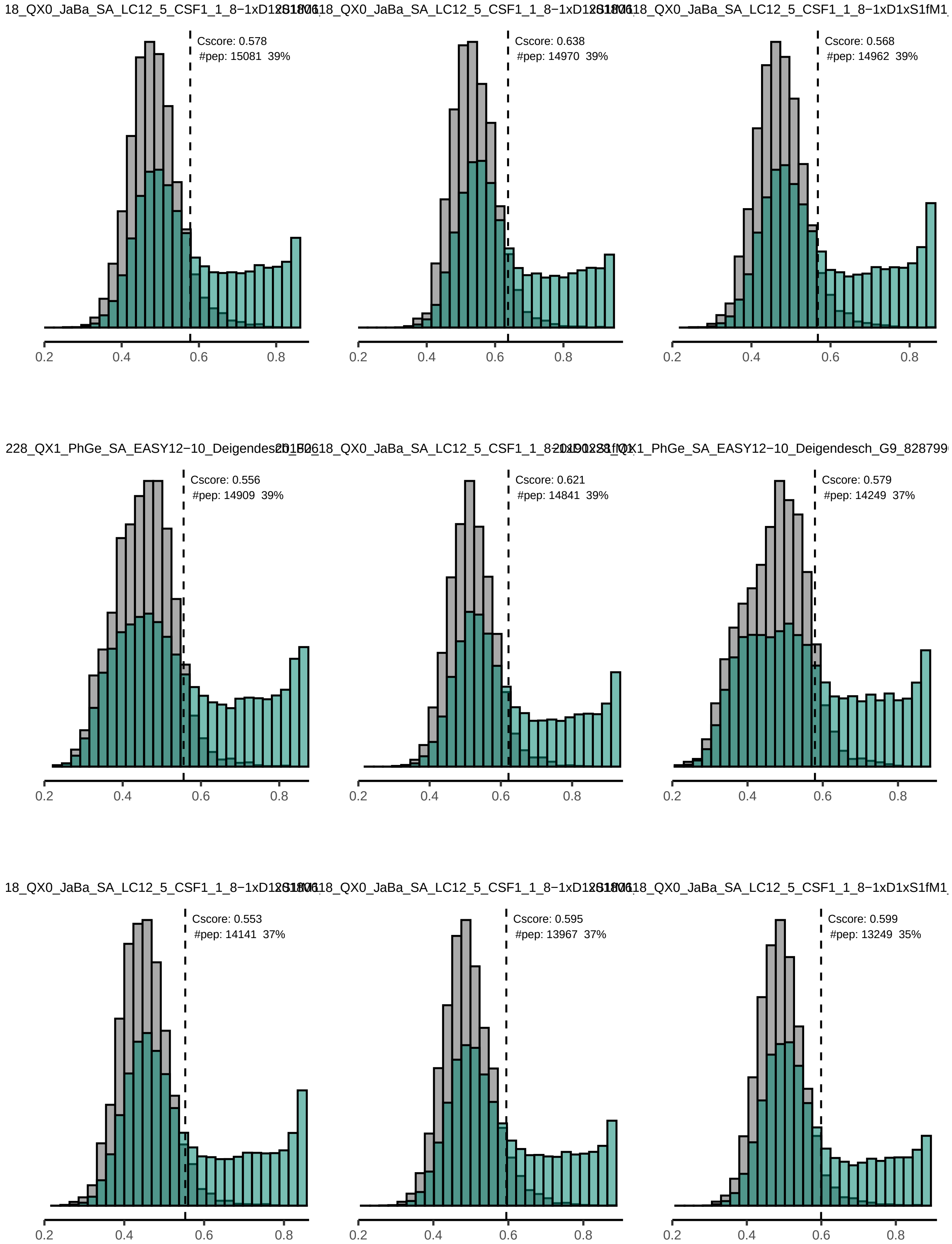

Cscore: 0.578
#pep: 15081 39%
Cscore: 0.638
#pep: 14970 39%
Cscore: 0.568
#pep: 14962 39%
Cscore: 0.556
#pep: 14909 39%
Cscore: 0.621
#pep: 14841 39%
Cscore: 0.579
#pep: 14249 37%
Cscore: 0.553
#pep: 14141 37%
Cscore: 0.595
#pep: 13967 37%
Cscore: 0.599
#pep: 13249 35%
0.2
0.4
0.6
0.8

1.2 number of peptides and proteins

These plots show the number of (target) peptides that are 'detected' per sample. For DDA, 'detected' implies the peptide has a MS/MS identification. Peptides quantified through match-between-runs (MBR) are quantified but not detected/identified. In case of DDA, we also show the number of peptides quantified through MBR. For DIA, we refer to a peptide as 'detected' if the confidence score (for identification) is <= 0.01.

Samples in this plot are sorted by their experimental group, and then ordered and by their name within each group. This data is also available in the output table 'samples.xlsx'.

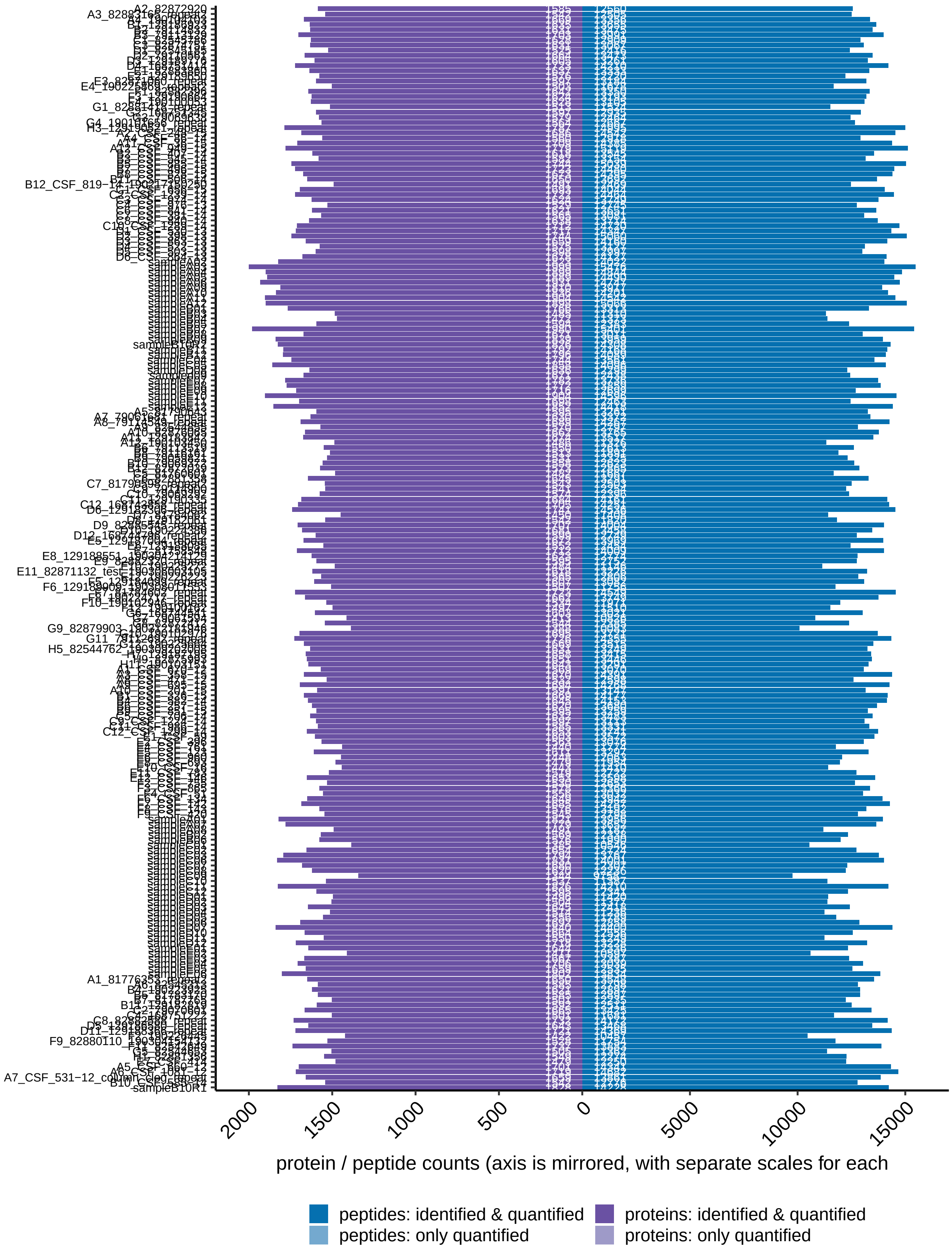
2000
1500
1000
500
0
5000
10000
15000
protein / peptide counts (axis is mirrored, with separate scales for each
peptides: identified & quantified
peptides: only quantified
proteins: identified & quantified
proteins: only quantified

1.2.1 color-coding sample metadata

The number of detected peptides in a sample, as compared to other samples within a dataset, can be used as a measure for sample quality. Color-coding individual samples for metadata that you provided as input (e.g. experiment batch, sample handling order, gel lanes, etc.) allows visual inspection as to whether these relate to the rate of successful peptide detection.

The figure below provides an overview of all sample metadata at a first glance. On each row all samples in the dataset are shown as a data point, each color-coded by the respective property shown on the y-axis (with minor vertical jitter for visual clarity). If any of these metadata coincide with a major effect on the number of detected peptides, this should become apparent by a clustering of samples by color-code. Hereafter, an additional set of figures will further expand this overview into detailed figures for each sample property.

Note that the visualization of sample metadata in this report depends on user-provided input; each column in the metadata input table (besides sample names) that contains more than 1 unique value is automatically used as a factor for color-coding all figures in this section. All information shown in these figures is also available in the output table 'samples.xlsx'.

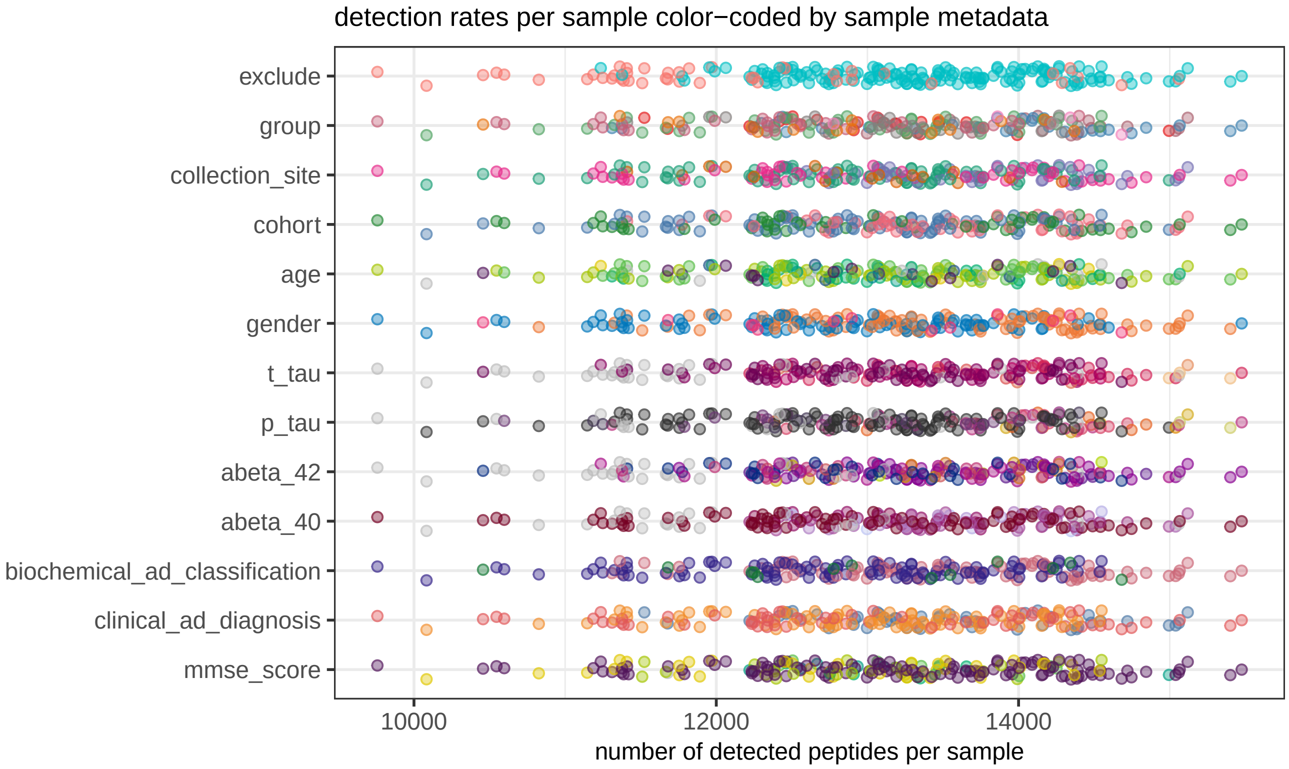

color-coding sample metadata, expanded

To further detail each sample property, each row in the above figure is now split into separate plots. Thus, a figure is generated for each property in the user-provided metadata (column in the samples table, its name shown in the plot title).

For categorical variables, a scatterplot shows on the y-axis all unique variables while the x-axis depicts the number of detected peptides. Colors are consistent with the above plot. *exclude* samples, if any, are depicted as squares. The median value is shown as a vertical line (thin line = median over all samples, wider line = median while discarding *exclude* samples). For continuous variables, a scatterplot without (left panel) and with Loess fit is shown (right panel, visualized as blue line if data was successfully fitted).

Note that samples flagged as *exclude* are user-provided in the sample metadata table. These are included in data visualizations but excluded from downstream statistical analysis (later part of the report).

For example: the first plot shows color-coding by the ‘group’ property, so each row represents a sample group. If samples in a particular group systematically yield fewer peptides than another group, a clear pattern will be visible.

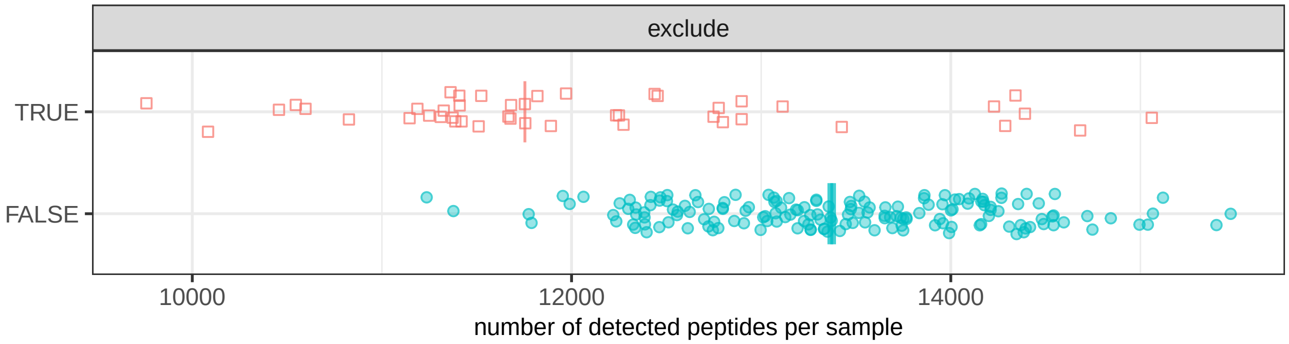

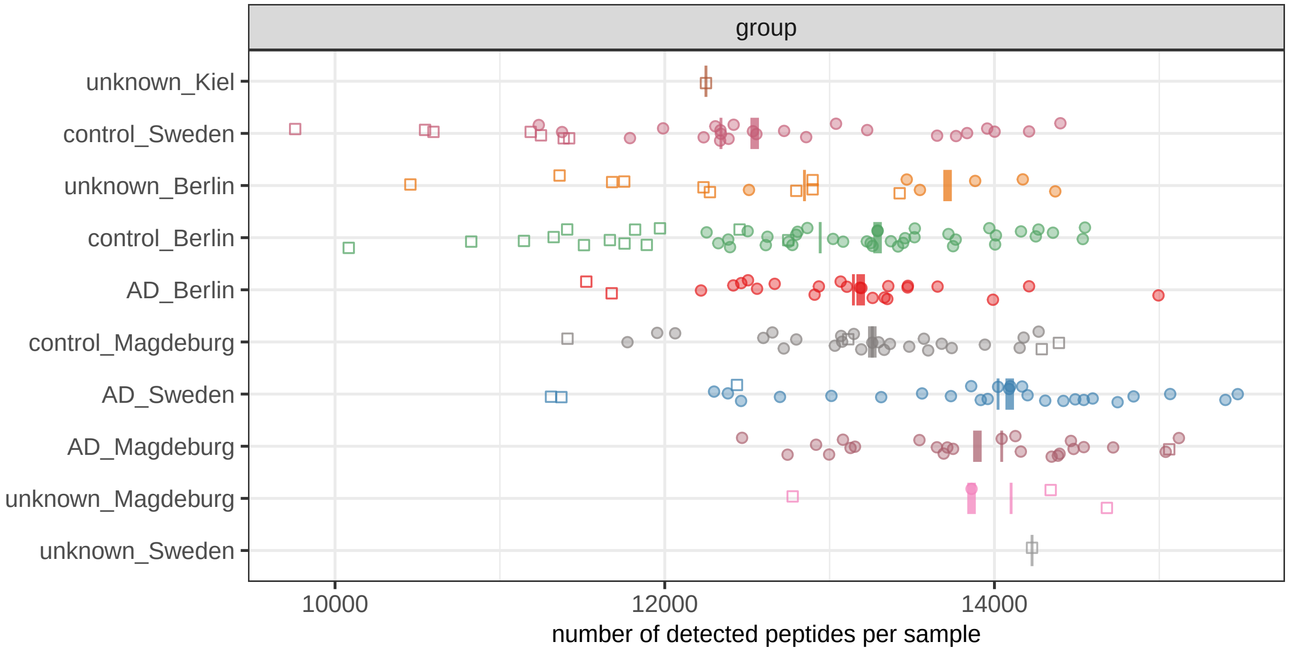

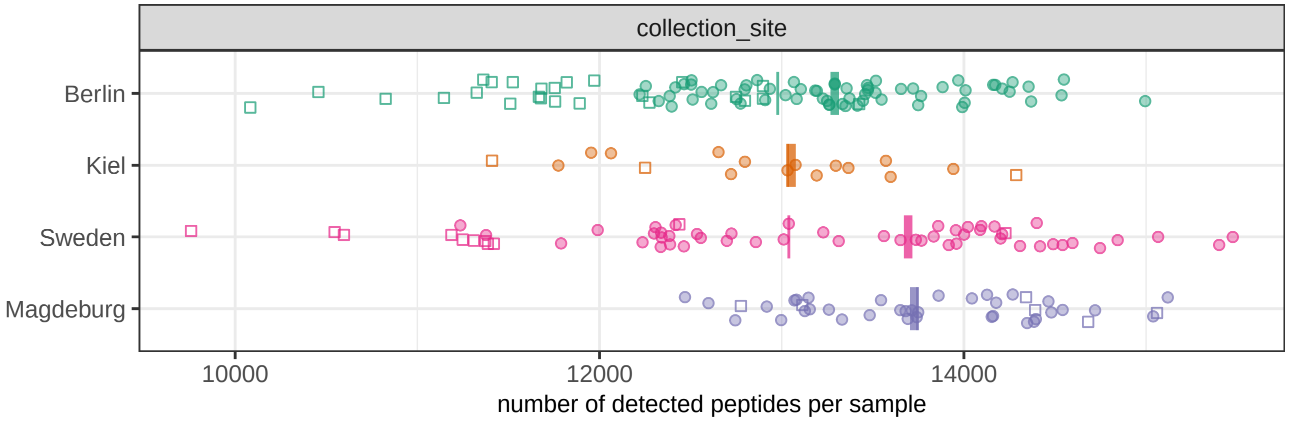

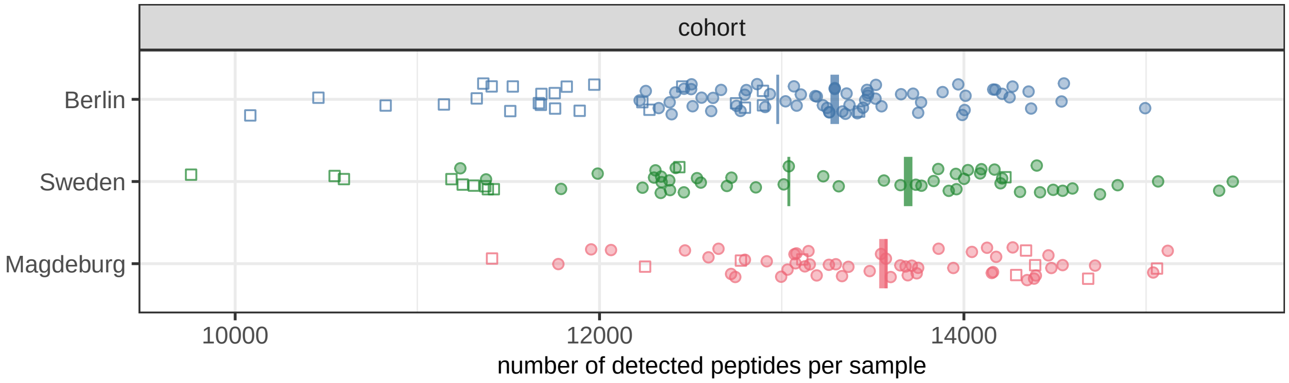
cohort
Berlin
Sweden
Magdeburg
10000
12000
14000
number of detected peptides per sample

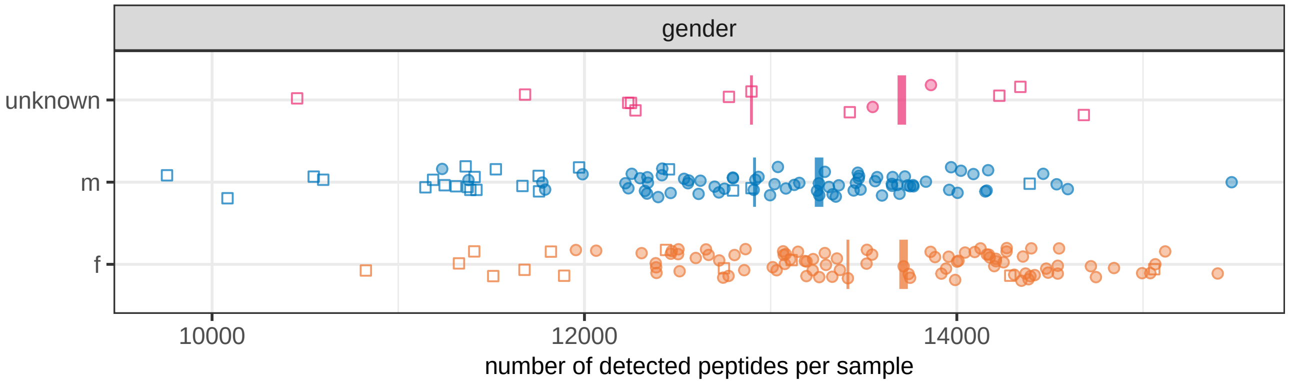
gender
unknown
m
f
10000
12000
14000
number of detected peptides per sample

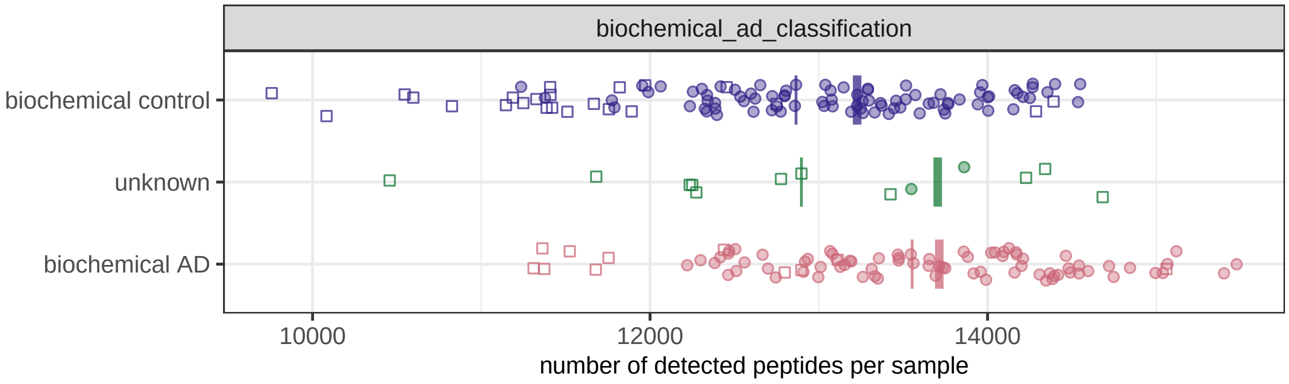
biochemical_ad_classification
biochemical control
unknown
biochemical AD
10000
12000
14000
number of detected peptides per sample

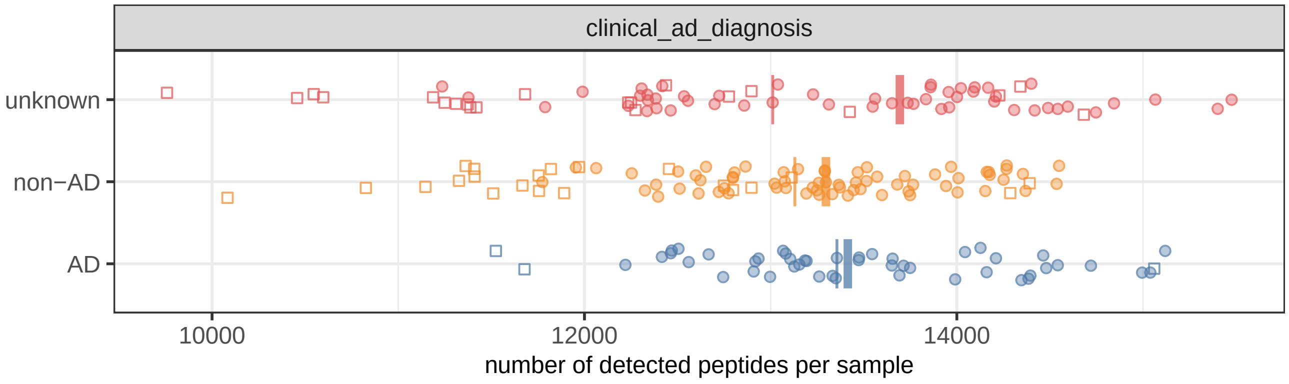
clinical_ad_diagnosis
unknown
non−AD
AD
10000
12000
14000
number of detected peptides per sample

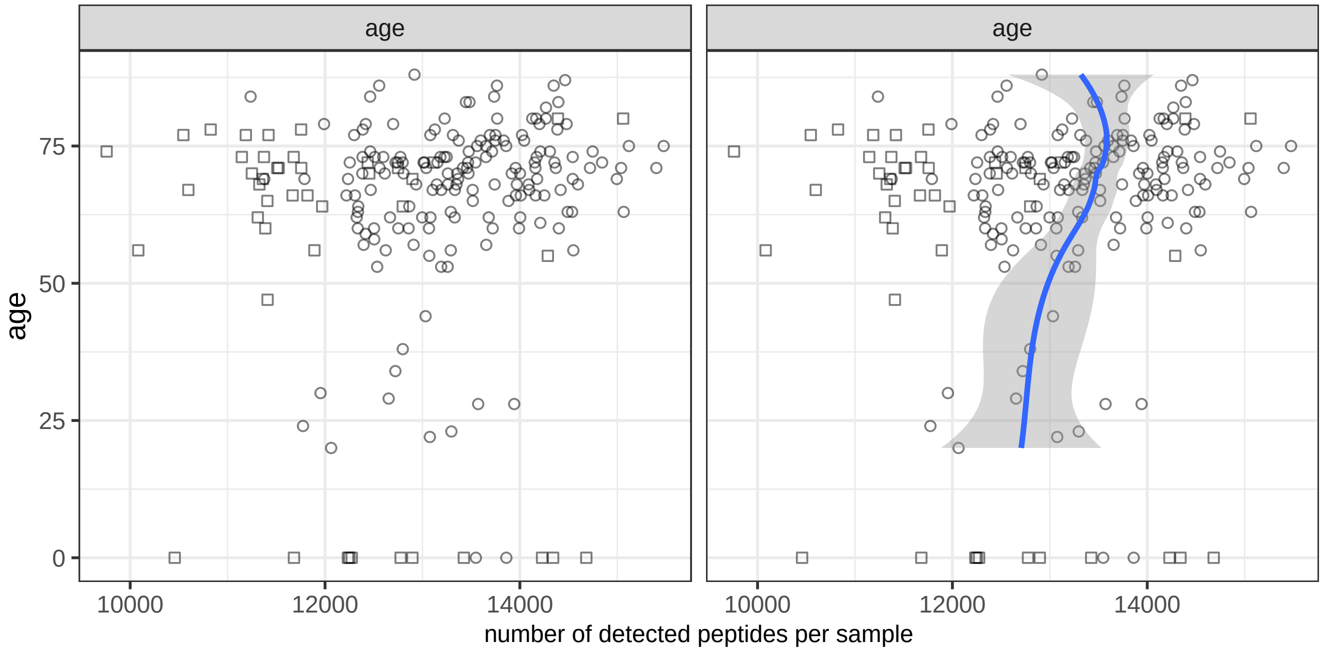
age
age
age
75
50
25
0
10000
12000
14000
number of detected peptides per sample

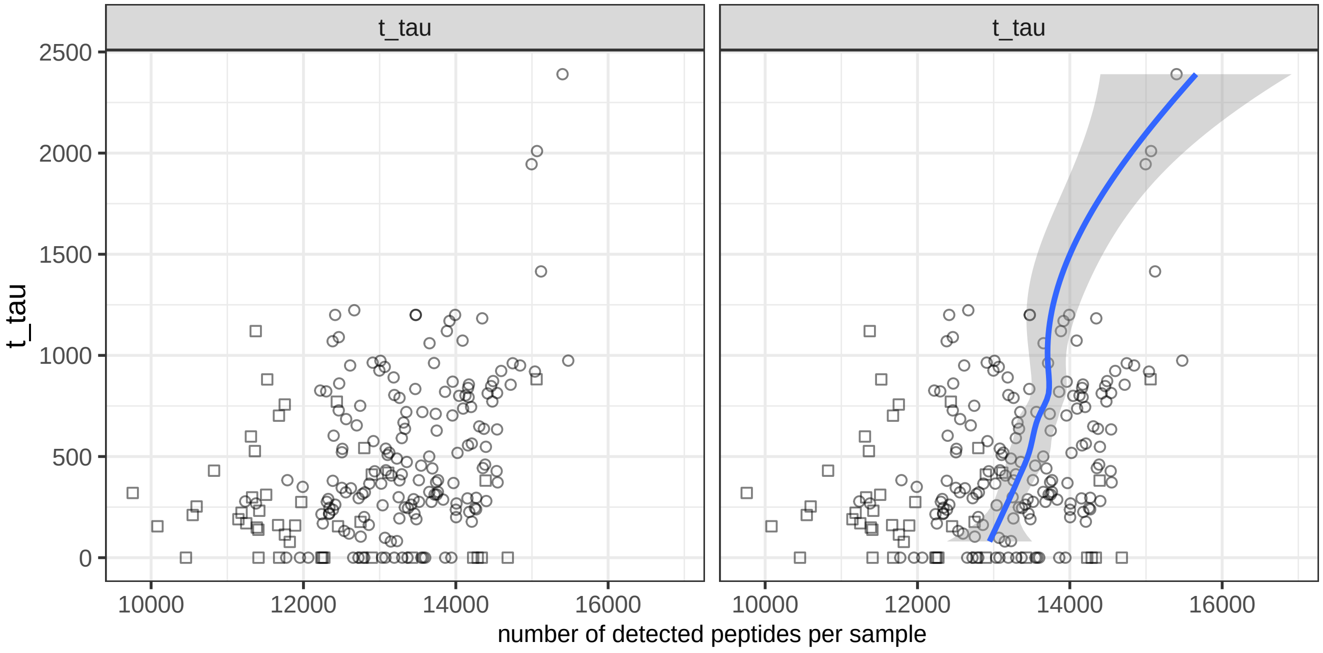
t_tau
t_tau
t_tau
2500
2000
1500
1000
500
0
10000
12000
14000
16000
number of detected peptides per sample

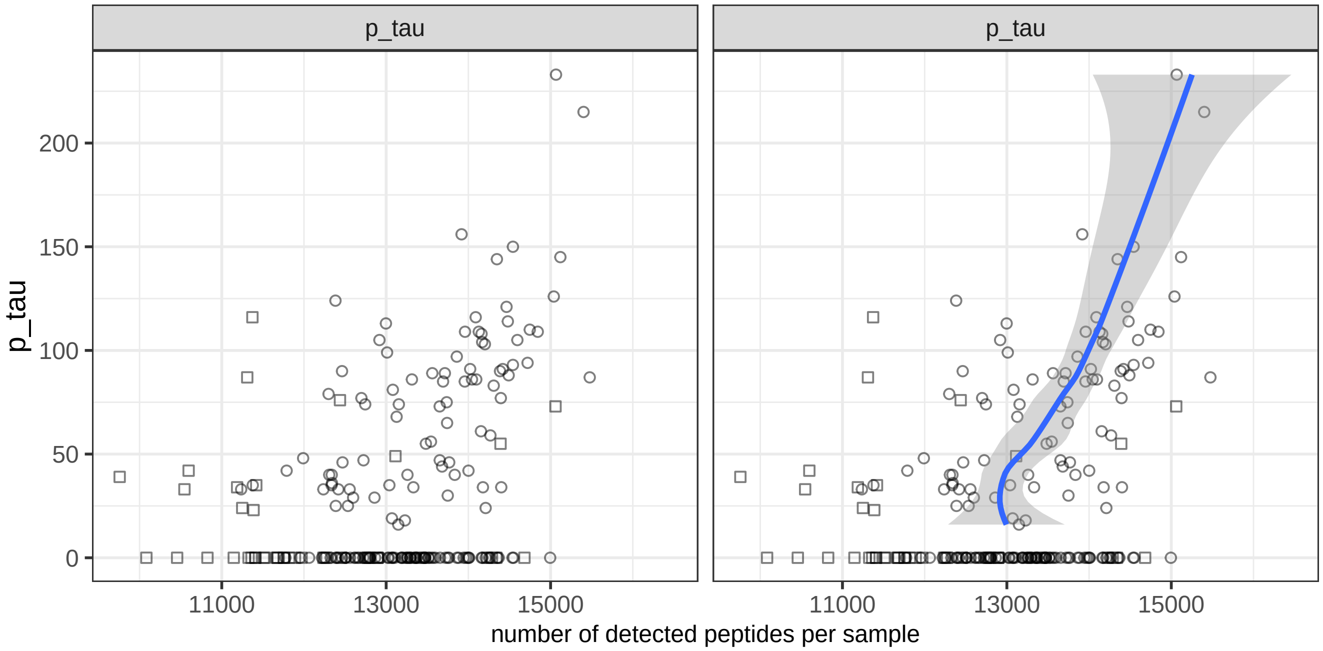
p_tau
p_tau
p_tau
200
150
100
50
0
11000
13000
15000
number of detected peptides per sample

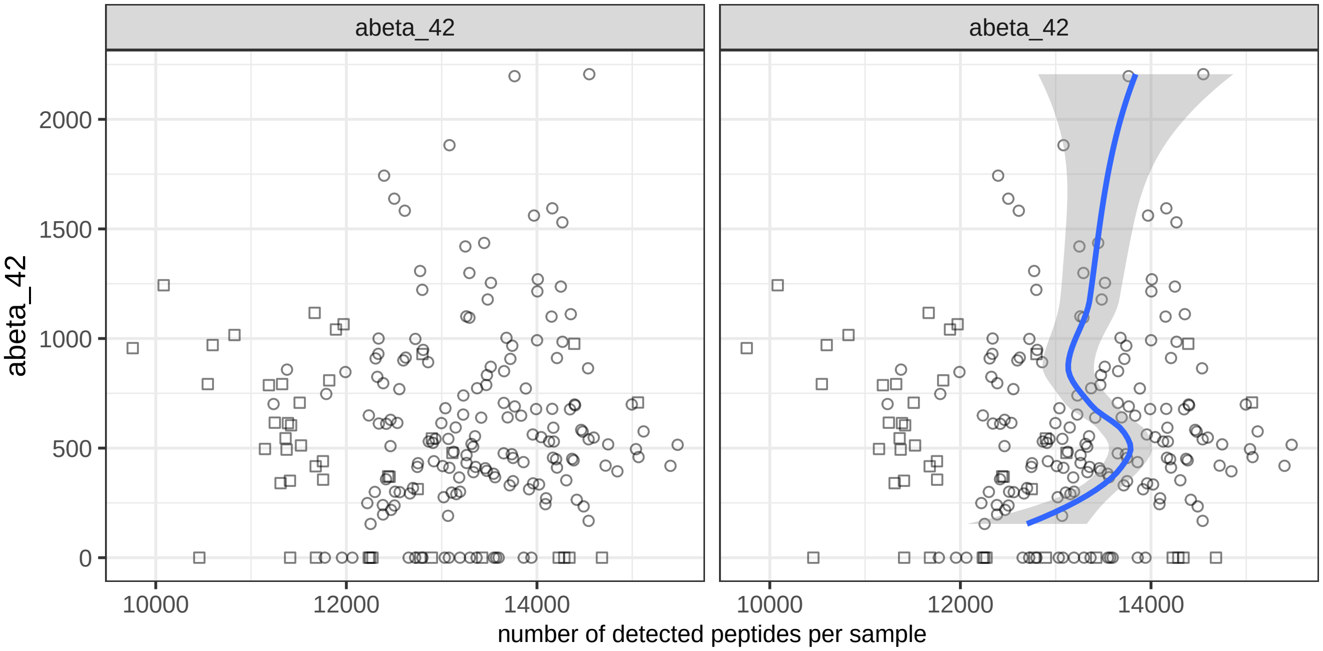
abeta_42
abeta_42
abeta_42
2000
1500
1000
500
0
10000
12000
14000
number of detected peptides per sample

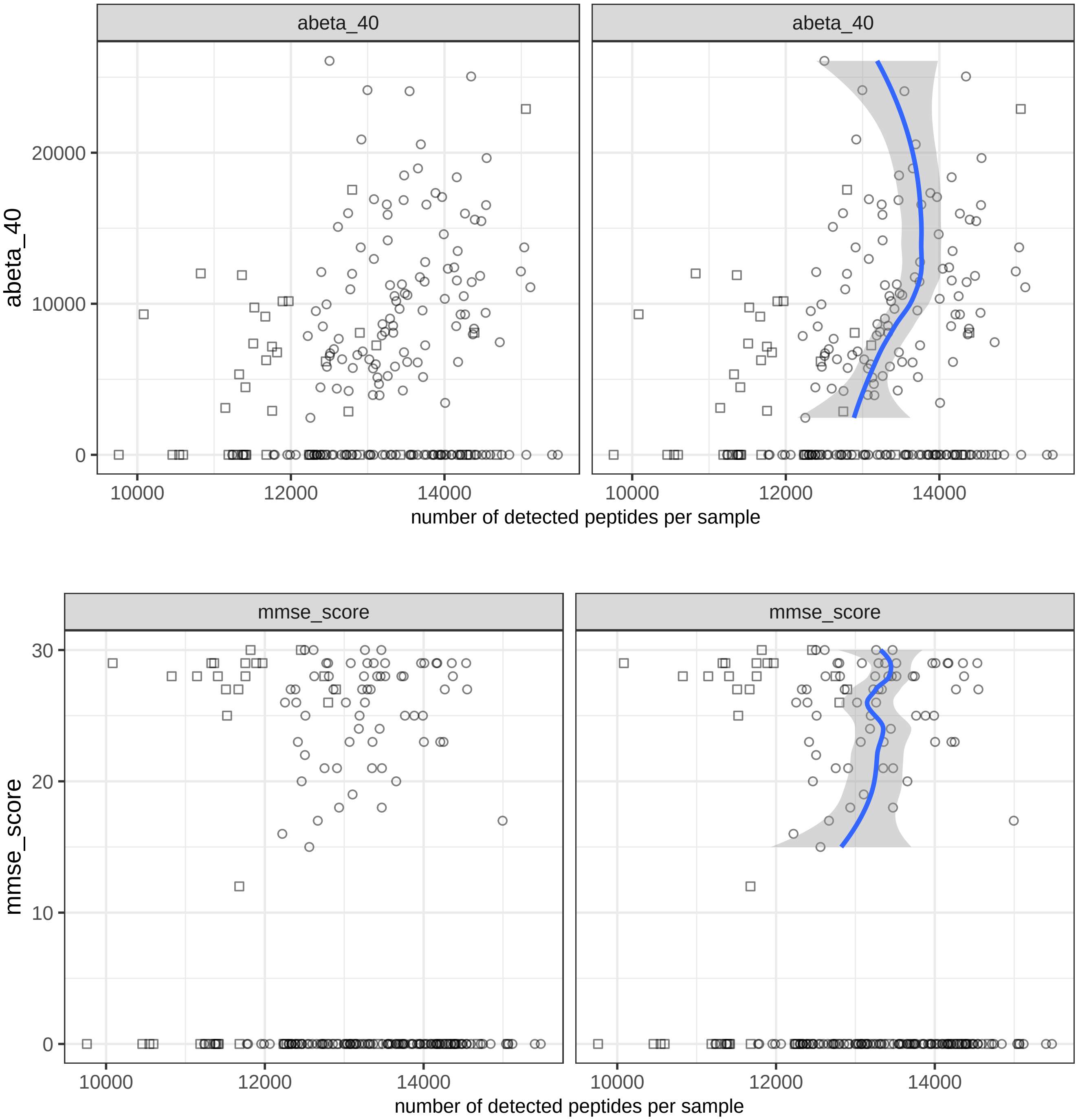
abeta_40
abeta_40
abeta_40
20000
10000
0
10000
12000
14000
10000
12000
14000
number of detected peptides per sample
mmse_score
mmse_score
mmse_score
30
20
10
0
10000
12000
14000
10000
12000
14000
number of detected peptides per sample

1.3 data completeness

To visualize how many peptides are consistently identified in multiple samples, the first figure summarizes how common missing values are in the entire dataset. Optimally, most peptides are identified in 100% of samples and this curve slowly falls of. The following figure shows for each sample whether its peptides are also present in other samples in the dataset or whether these are unique to a (minor) subset of samples. You can use this mark of experimental consistency to compare datasets generated by similar protocols and mass-spec acquisition.

1.3.1 cumulative distribution

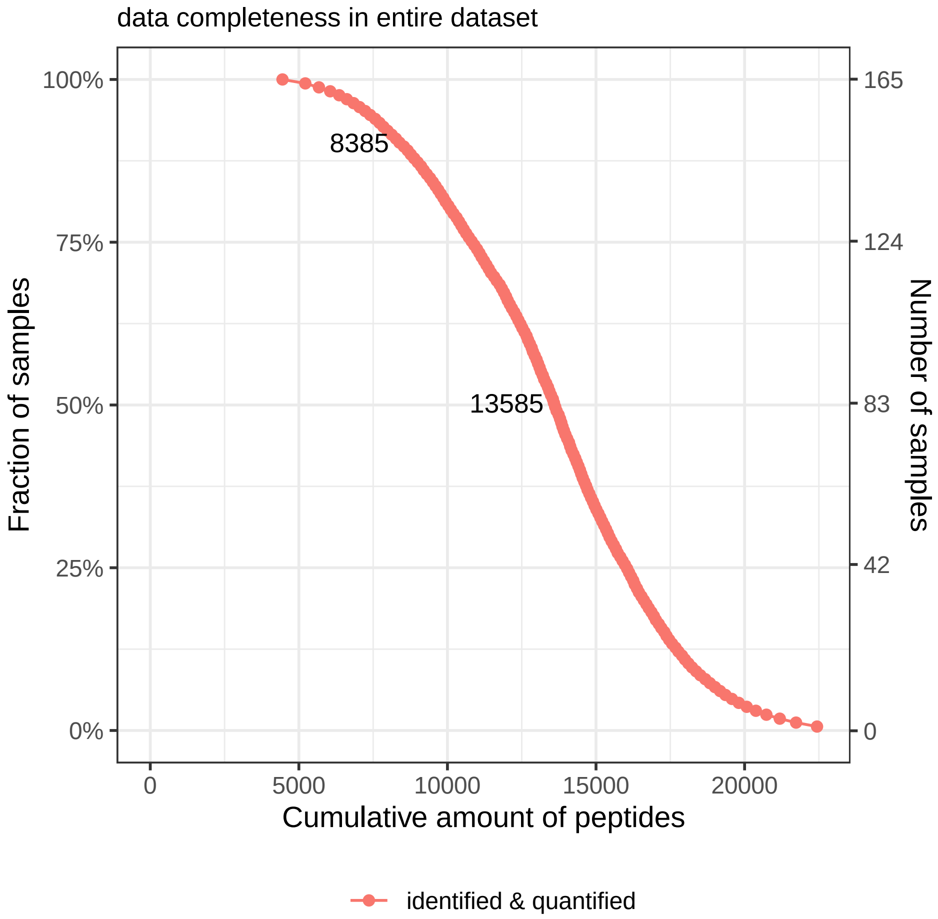

Samples flagged as 'exclude' (by user) are not taken into account in this figure. Exact values are shown for data points matching 90% and 50% of samples to convenience comparison between analyses (e.g. before/after configuring 'exclude' samples, or comparing between experiments of similar protocol).

1.3.2 peptide detection frequency

Each identified peptide in a sample is classified and color-coded by the number of other samples where the same peptide is present. Visualization of the amount of peptides that overlap with other samples in the dataset, from peptides identified in most samples (red) to one-hit-wonders (blue), helps identify uncommon samples (more blue/green than other samples).

Optimally, the majority of peptides in each sample are red~orange with relatively few uniquely identified peptides (blue~green). Samples are sorted by the total amount of detected peptides.

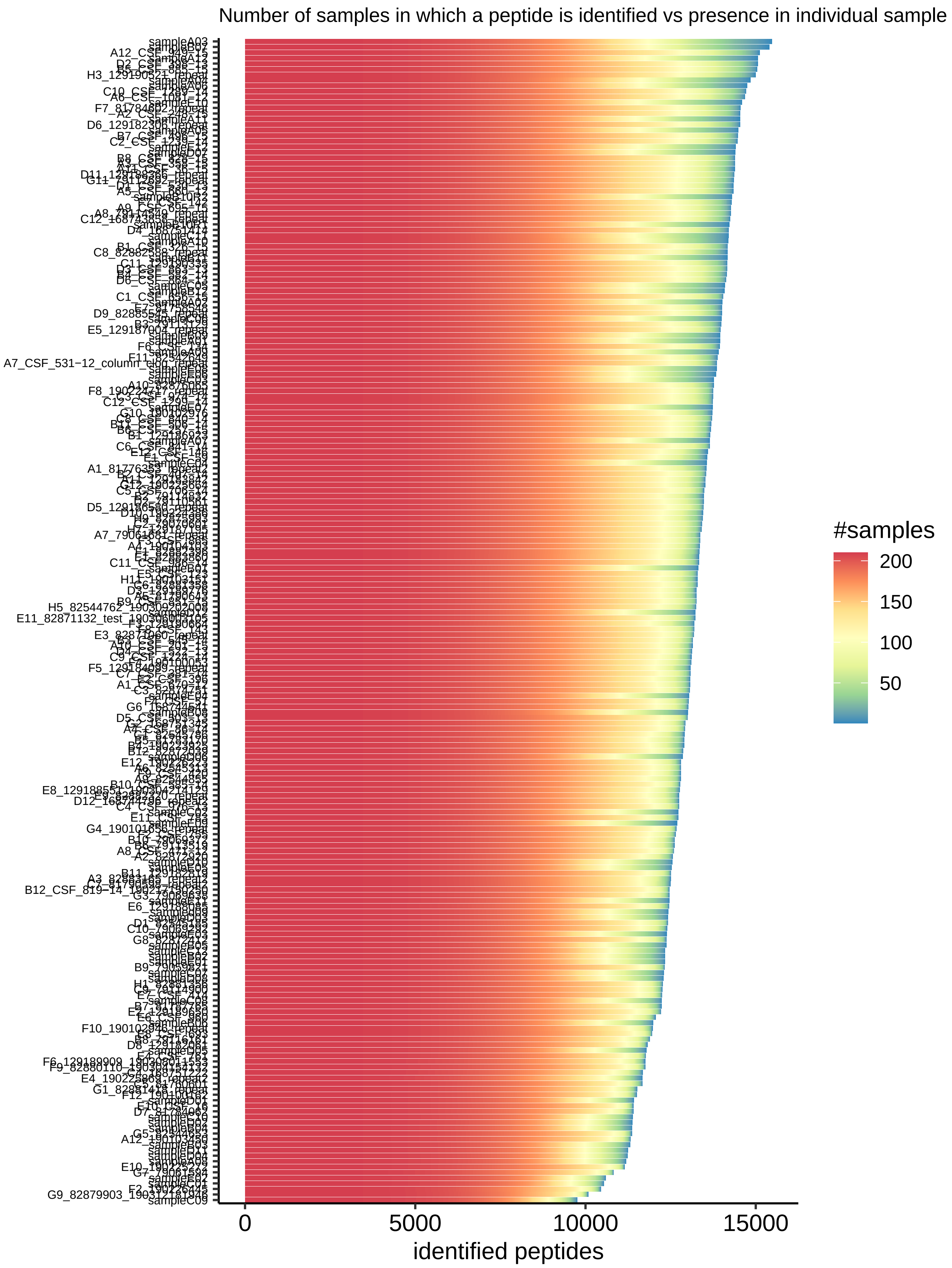
Number of samples in which a peptide is identified vs presence in individual sample
identified peptides
0
5000
10000
15000
#samples
200
150
100
50

1.4 abundance distributions

The figures in this subsection are used to identify unexpected mass-spec sensitivity or sample loading differences. Peptide data is shown as provided in input files, so peptide filtering nor intensity normalization has been applied yet (for proper QC, make sure the software that generated the input data did not apply normalization prior). If the dataset is DDA, match-between-runs (MBR) peptides are included in these distributions whereas for DIA only 'detected' peptides (based on confidence score threshold) are included.

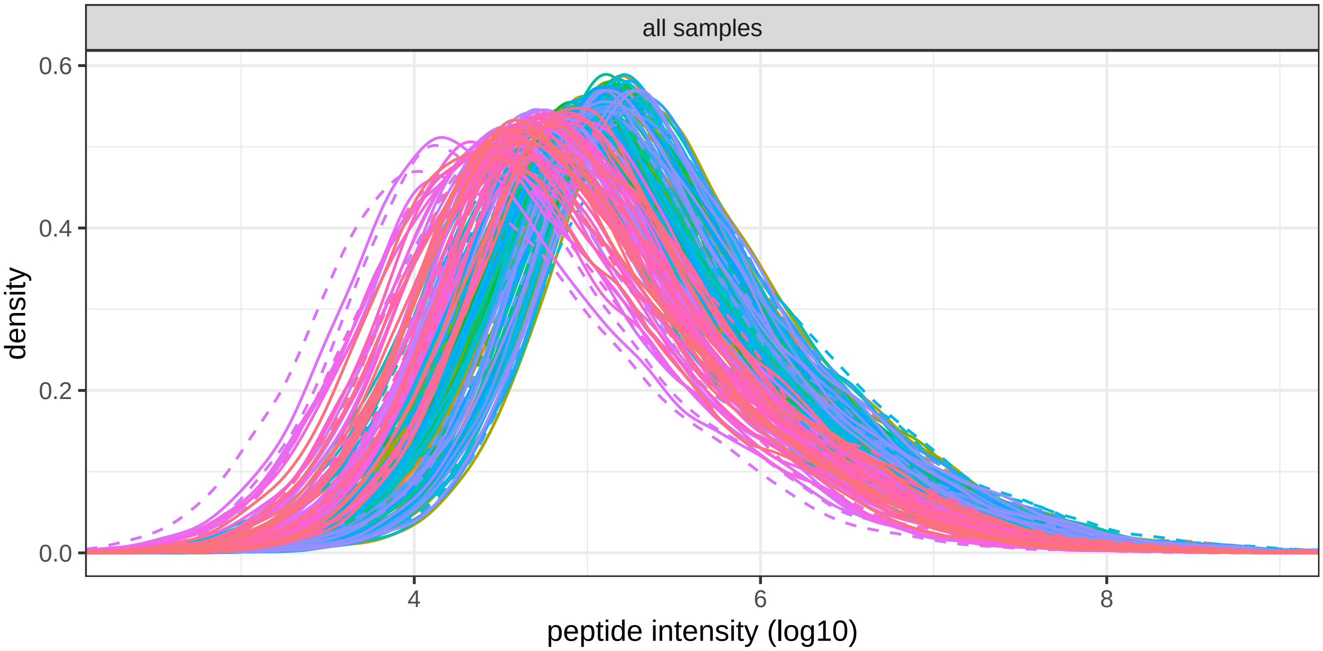

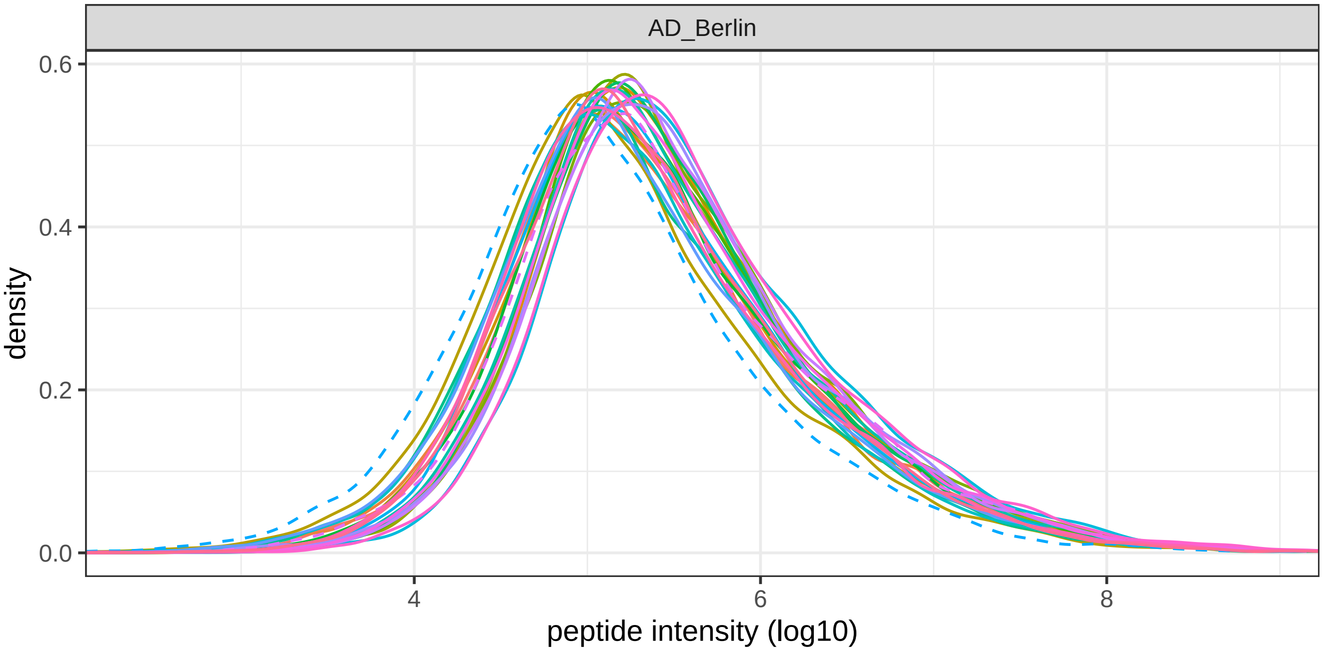

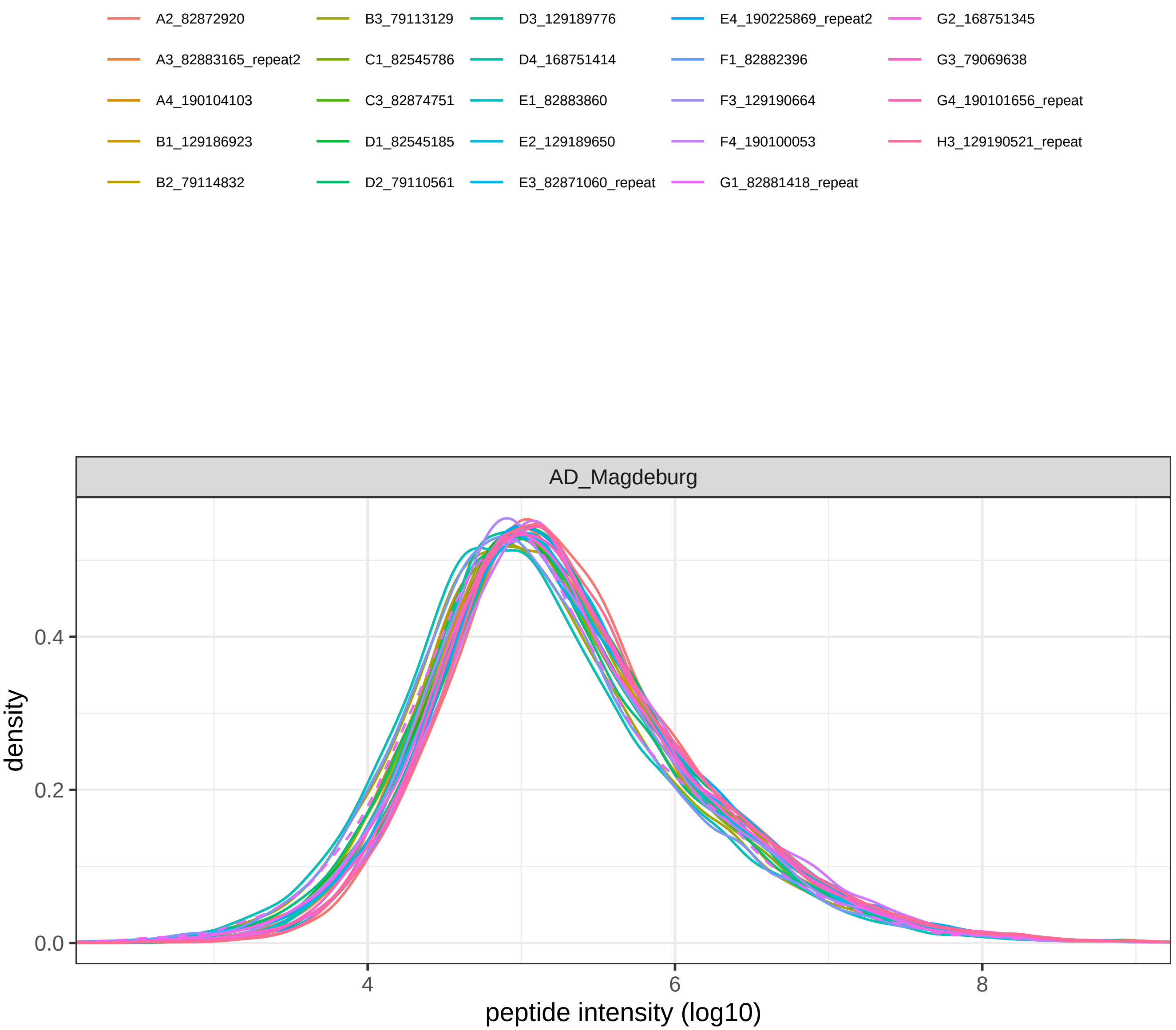
A2_82872920
A3_82883165_repeat2
A4_190104103
B1_129186923
B2_79114832
B3_79113129
C1_82545786
C3_82874751
D1_82545185
D2_79110561
D3_129189776
D4_168751414
E1_82883860
E2_129189650
E3_82871060_repeat
E4_190225869_repeat2
F1_82882396
F3_129190664
F4_190100053
G1_82881418_repeat
G2_168751345
G3_79069638
G4_190101656_repeat
H3_129190521_repeat
AD_Magdeburg
density
0.0
0.2
0.4
4
6
8
peptide intensity (log10)

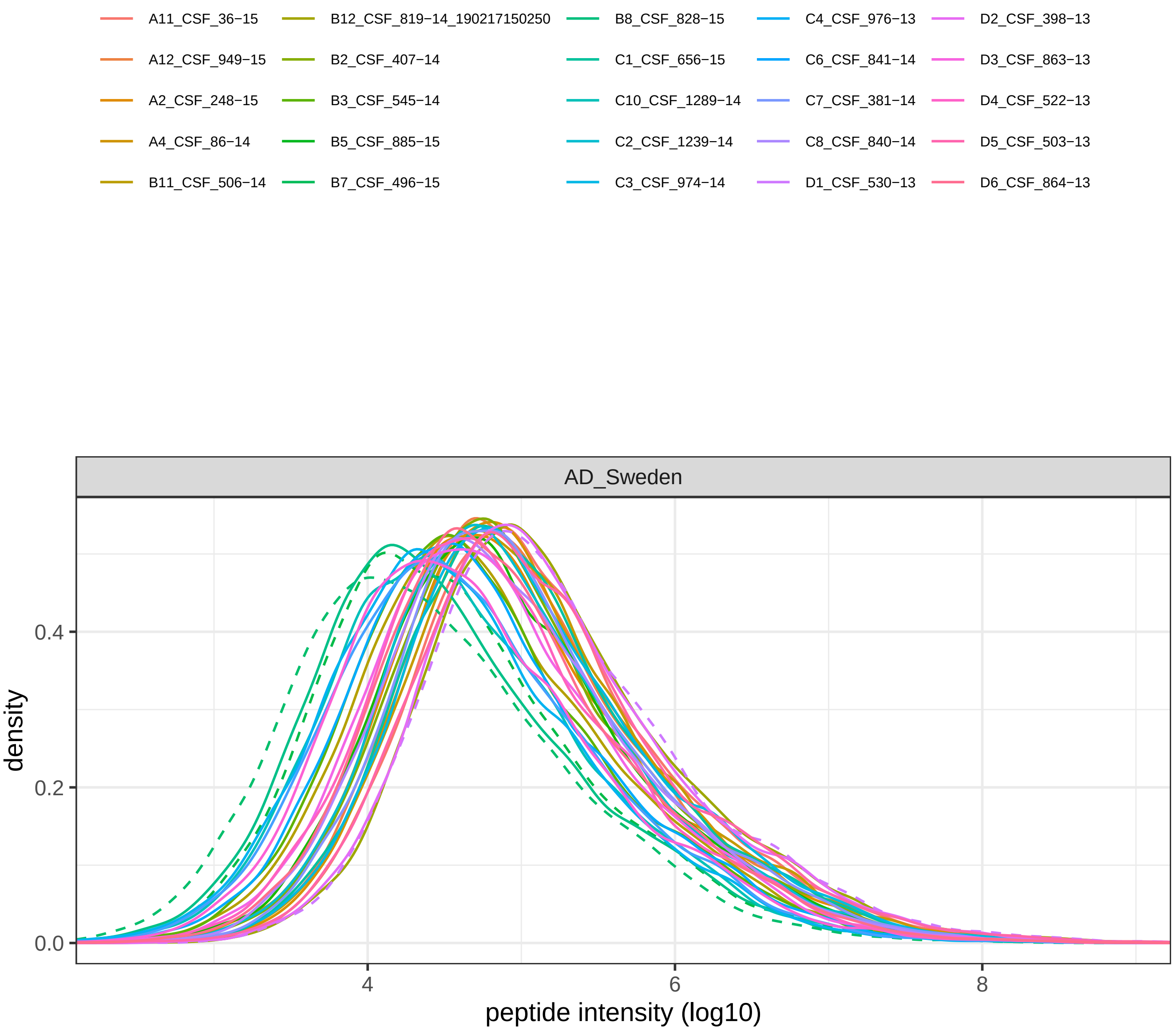
A11_CSF_36−15
A12_CSF_949−15
A2_CSF_248−15
A4_CSF_86−14
B11_CSF_506−14
B12_CSF_819−14_190217150250
B2_CSF_407−14
B3_CSF_545−14
B5_CSF_885−15
B7_CSF_496−15
B8_CSF_828−15
C1_CSF_656−15
C10_CSF_1289−14
C2_CSF_1239−14
C3_CSF_974−14
C4_CSF_976−13
C6_CSF_841−14
C7_CSF_381−14
C8_CSF_840−14
D1_CSF_530−13
D2_CSF_398−13
D3_CSF_863−13
D4_CSF_522−13
D5_CSF_503−13
D6_CSF_864−13
AD_Sweden
density
0.0
0.2
0.4
4
6
8
peptide intensity (log10)

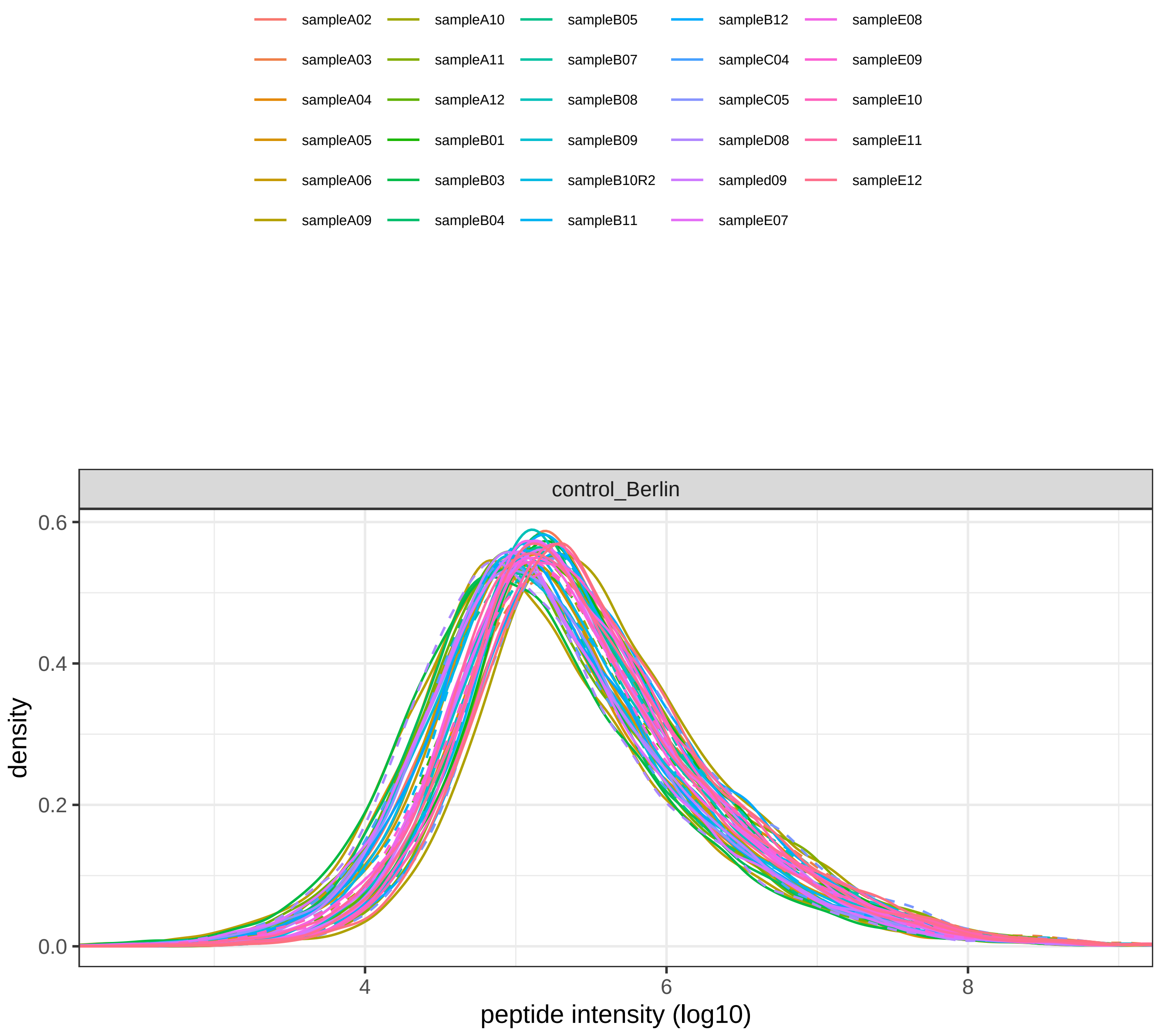
sampleA02
sampleA03
sampleA04
sampleA05
sampleA06
sampleA09
sampleA10
sampleA11
sampleA12
sampleB01
sampleB03
sampleB04
sampleB05
sampleB07
sampleB08
sampleB09
sampleB10R2
sampleB11
sampleB12
sampleC04
sampleC05
sampleD08
sampled09
sampleE07
sampleE08
sampleE09
sampleE10
sampleE11
sampleE12
control_Berlin
0.6
0.4
0.2
0.0
density
4
6
8
peptide intensity (log10)

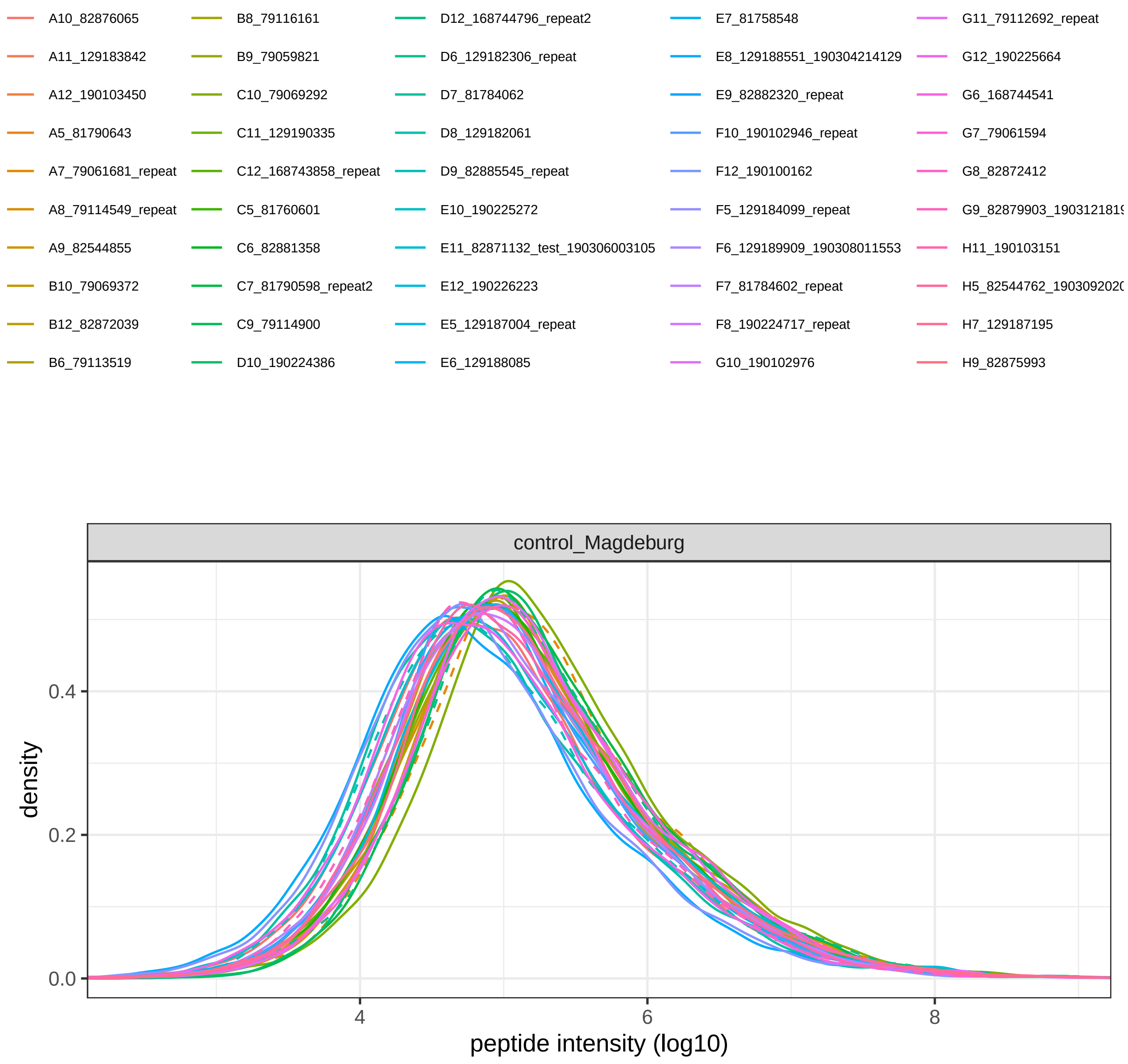
A10_82876065
A11_129183842
A12_190103450
A5_81790643
A7_79061681_repeat
A8_79114549_repeat
A9_82544855
B10_79069372
B12_82872039
B6_79113519
B8_79116161
B9_79059821
C10_79069292
C11_129190335
C12_168743858_repeat
C5_81760601
C6_82881358
C7_81790598_repeat2
C9_79114900
D10_190224386
D12_168744796_repeat2
D6_129182306_repeat
D7_81784062
D8_129182061
D9_82885545_repeat
E10_190225272
E11_82871132_test_190306003105
E12_190226223
E5_129187004_repeat
E6_129188085
E7_81758548
E8_129188551_190304214129
E9_82882320_repeat
F10_190102946_repeat
F12_190100162
F5_129184099_repeat
F6_129189909_190308011553
F7_81784602_repeat
F8_190224717_repeat
G10_190102976
G11_79112692_repeat
G12_190225664
G6_168744541
G7_79061594
G8_82872412
H11_190103151
H7_129187195
H9_82875993
control_Magdeburg
density
0.0
0.2
0.4
4
6
8
peptide intensity (log10)

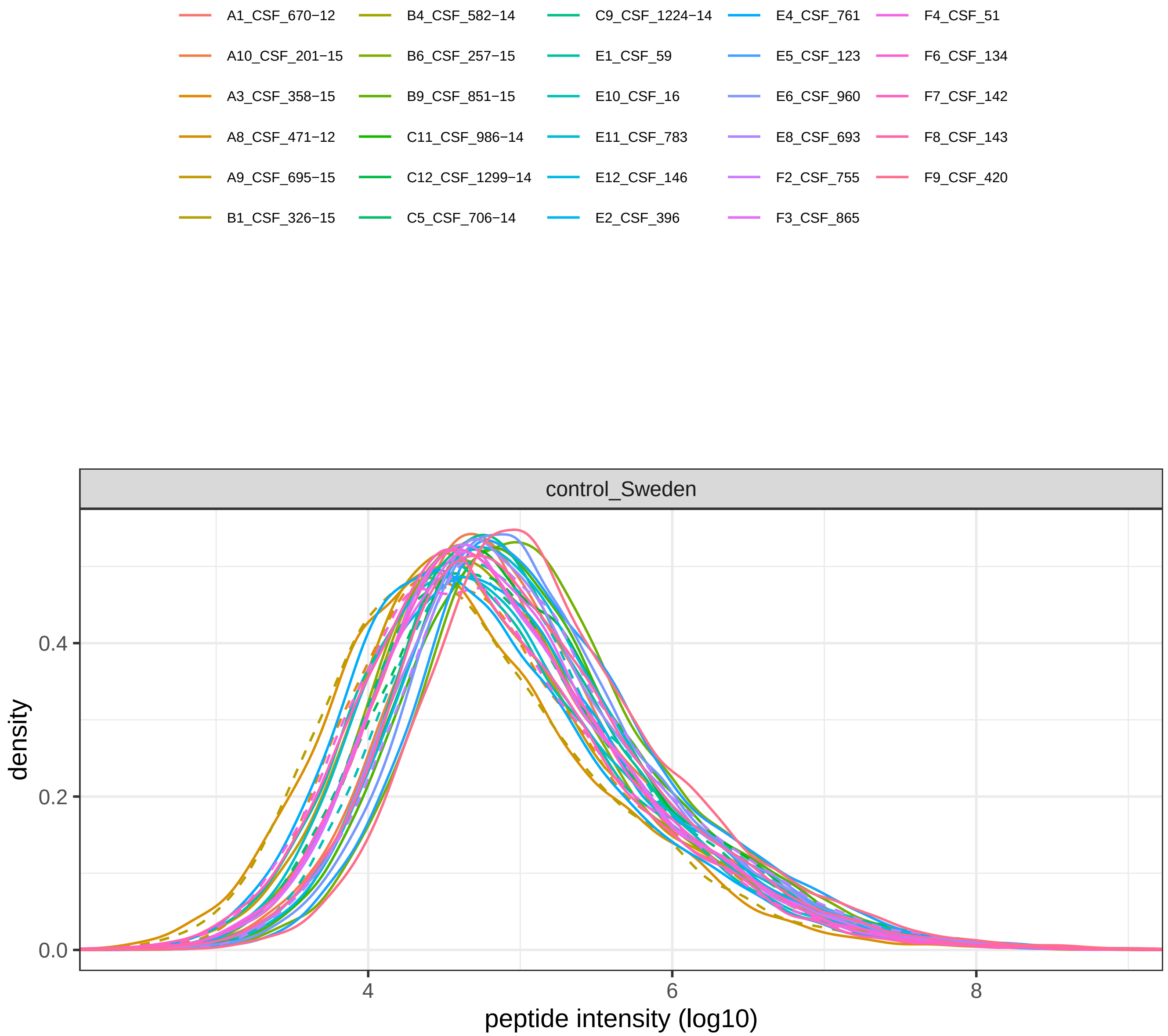
A1_CSF_670−12
A10_CSF_201−15
A3_CSF_358−15
A8_CSF_471−12
A9_CSF_695−15
B1_CSF_326−15
B4_CSF_582−14
B6_CSF_257−15
B9_CSF_851−15
C11_CSF_986−14
C12_CSF_1299−14
C5_CSF_706−14
C9_CSF_1224−14
E1_CSF_59
E10_CSF_16
E11_CSF_783
E12_CSF_146
E2_CSF_396
E4_CSF_761
E5_CSF_123
E6_CSF_960
E8_CSF_693
F2_CSF_755
F3_CSF_865
F4_CSF_51
F6_CSF_134
F7_CSF_142
F8_CSF_143
F9_CSF_420
control_Sweden
density
0.0
0.2
0.4
4
6
8
peptide intensity (log10)

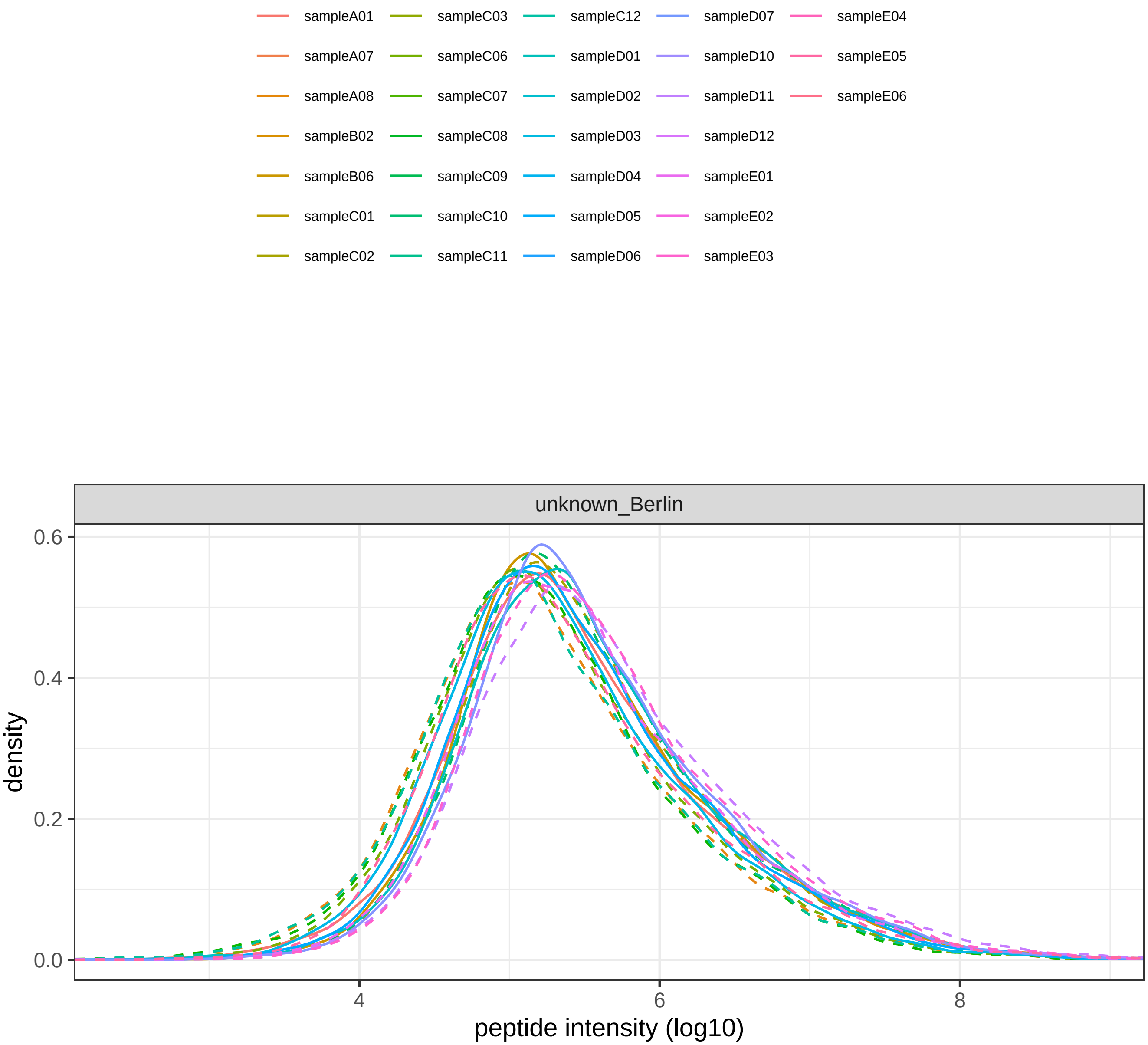
sampleA01
sampleA07
sampleA08
sampleB02
sampleB06
sampleC01
sampleC02
sampleC03
sampleC06
sampleC07
sampleC08
sampleC09
sampleC10
sampleC11
sampleC12
sampleD01
sampleD02
sampleD03
sampleD04
sampleD05
sampleD06
sampleD07
sampleD10
sampleD11
sampleD12
sampleE01
sampleE02
sampleE03
sampleE04
sampleE05
sampleE06
unknown_Berlin
density
0.0
0.2
0.4
0.6
4
6
8
peptide intensity (log10)

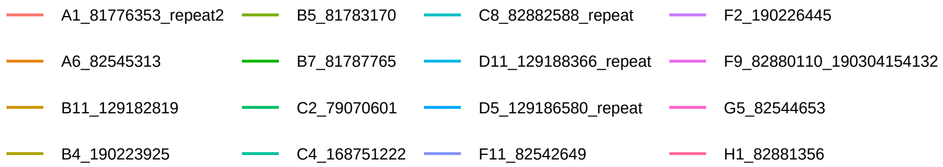
A1_81776353_repeat2
A6_82545313
B11_129182819
B4_190223925
B5_81783170
B7_81787765
C2_79070601
C4_168751222
C8_82882588_repeat
D11_129188366_repeat
D5_129186580_repeat
F11_82542649
F2_190226445
F9_82880110_190304154132
G5_82544653
H1_82881356

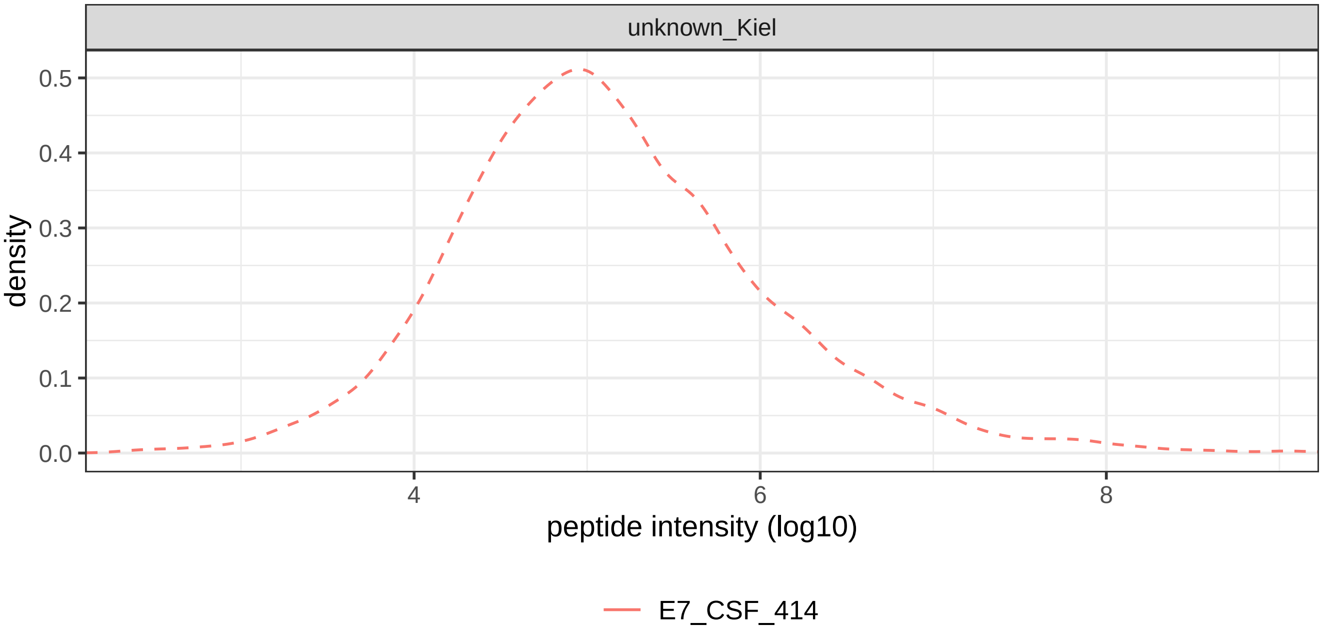
unknown_Kiel
density
0.5
0.4
0.3
0.2
0.1
0.0
4
6
8
peptide intensity (log10)
E7_CSF_414

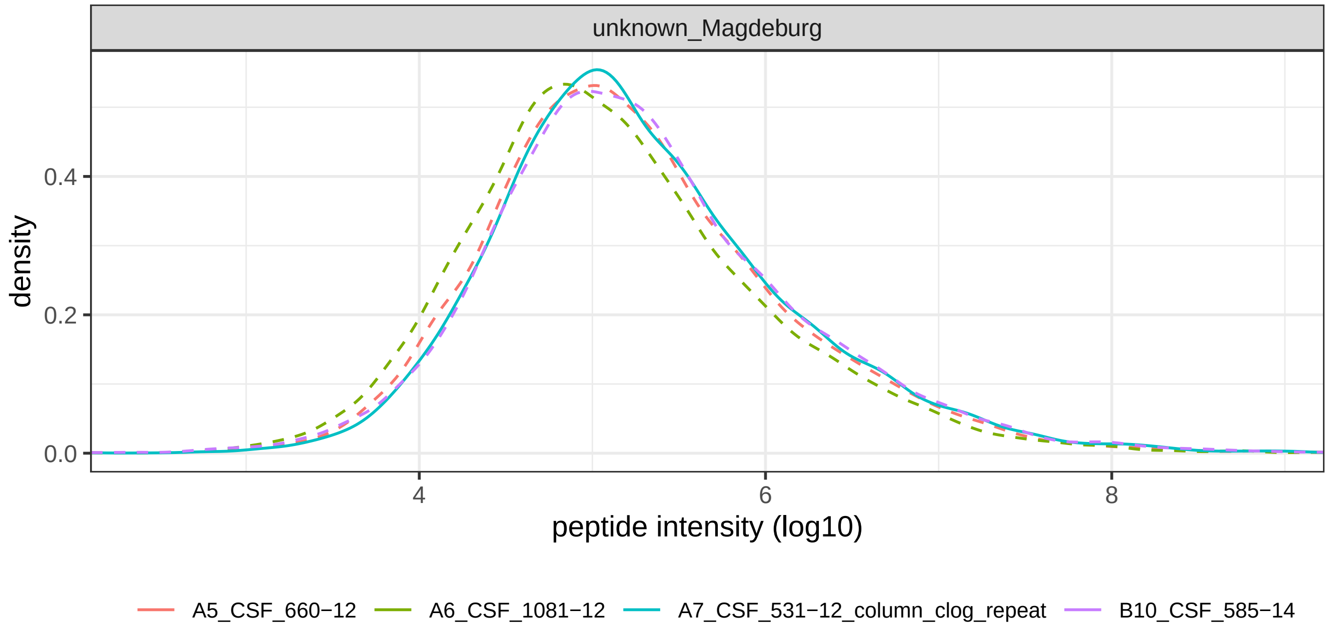
unknown_Magdeburg
density
0.0
0.2
0.4
4
6
8
peptide intensity (log10)
A5_CSF_660−12
A6_CSF_1081−12
A7_CSF_531−12_column_clog_repeat
B10_CSF_585−14

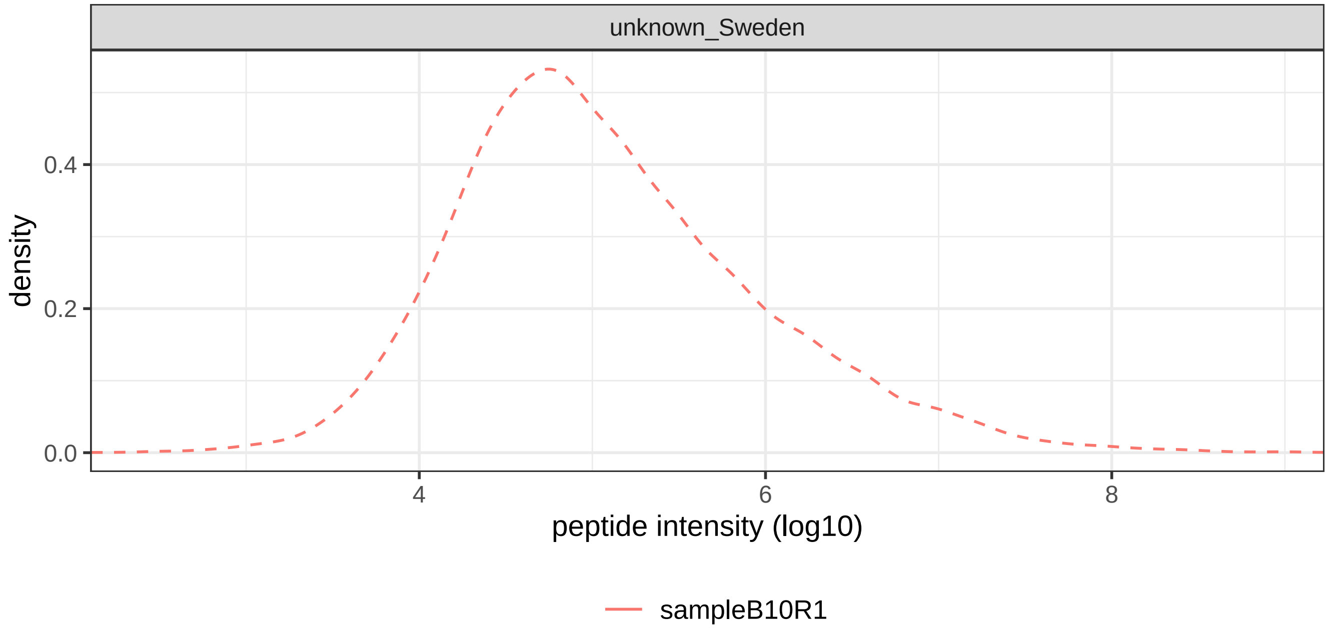
unknown_Sweden
density
0.0
0.2
0.4
4
6
8
peptide intensity (log10)
sampleB10R1

1.5 retention time

The figures in this section allow you to identify potential problems during HPLC elution, such as a temporarily blocking column, failing ionization spray or decreasing sensitivity over time. For each sample, all peptides that are also observed in a replicate (such that there is a point of reference available) are visualized.

1.5.1 retention time distributions

The density of the number of peptides eluting at each point in time. The figure below presents an overview of all samples that allows for the identification of outlier samples that follow distinct elution patterns. The following section shows details for each sample. Samples marked as 'exclude' in the provided sample metadata table are visualized as dashed lines.

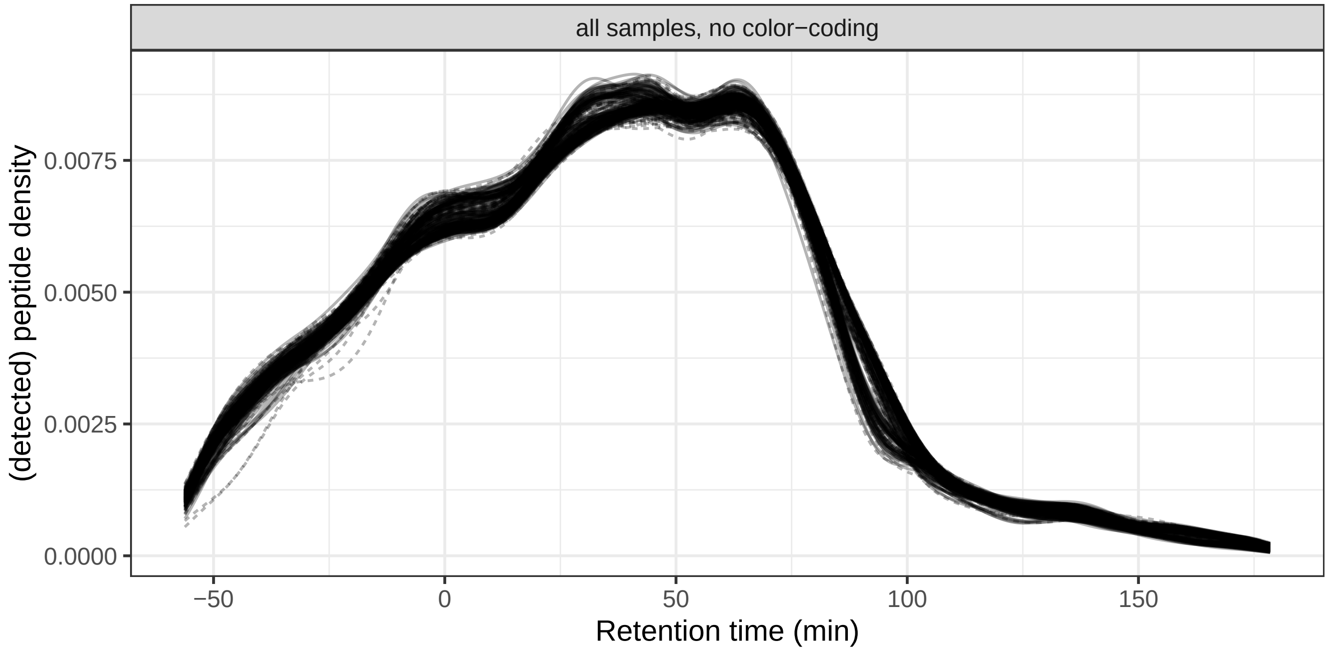

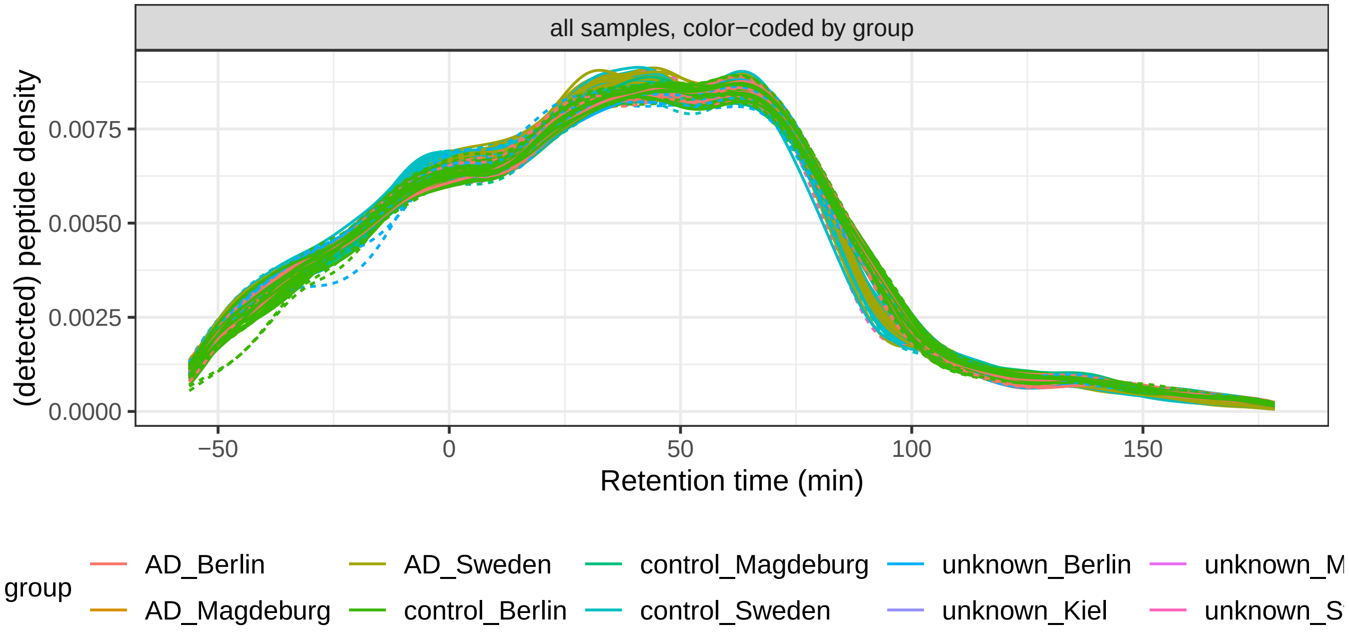

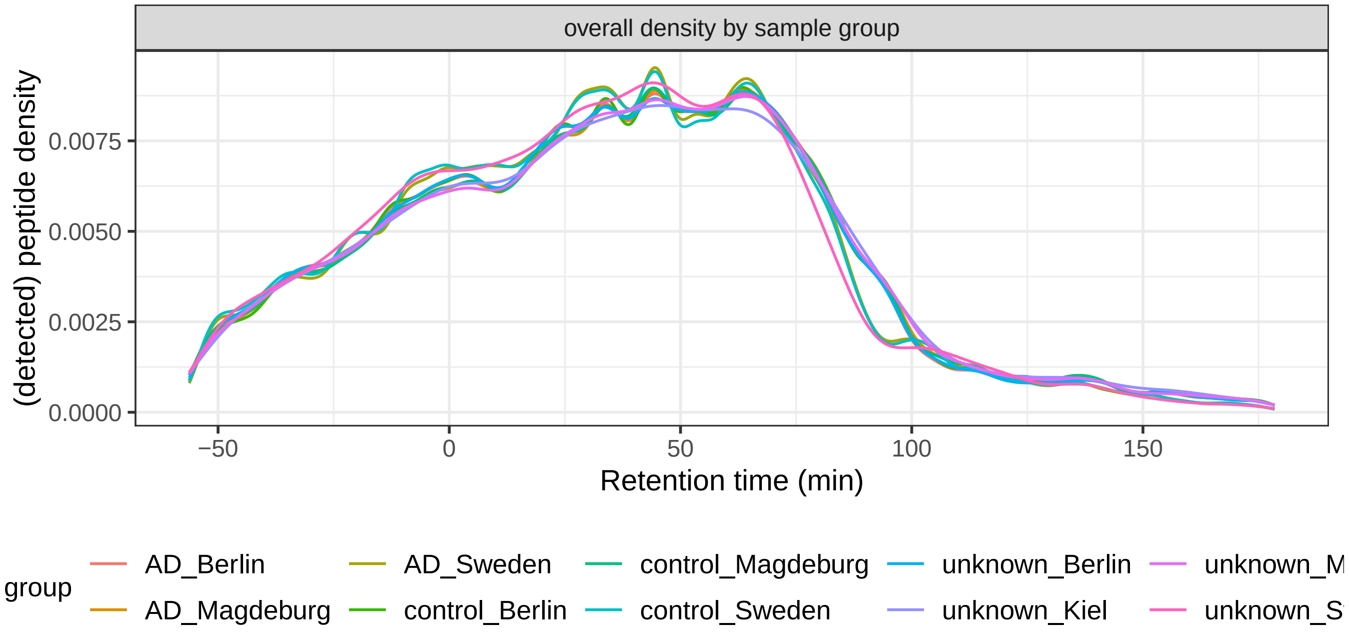

1.5.2 retention time local effects

To investigate how each measurement differs from others, we visualize each sample as a 3 panel figure. First, the data is binned across the retention time dimension (x-axis). If a samples was marked as 'exclude' in the provided sample metadata, this is indicated in the plot title.

The top panel shows the number of peptides in the input data, e.g. as recognized by the software that generated input for this pipeline, over time (black line). For reference, the grey line shows the median amount over all samples (note; if this is the exact same in all samples, the grey line may not be visible as it falls behind the black line).

The middle panel indicates whether peptide retention times deviate from their median over all samples (blue line). The grey area depicts the 5% and 95% quantiles, respectively. The line width corresponds to the number of peptides eluting at that time (data from first panel). Analogously, the bottom panel shows the deviation in peptide abundance as compared to the median over all samples (red line).

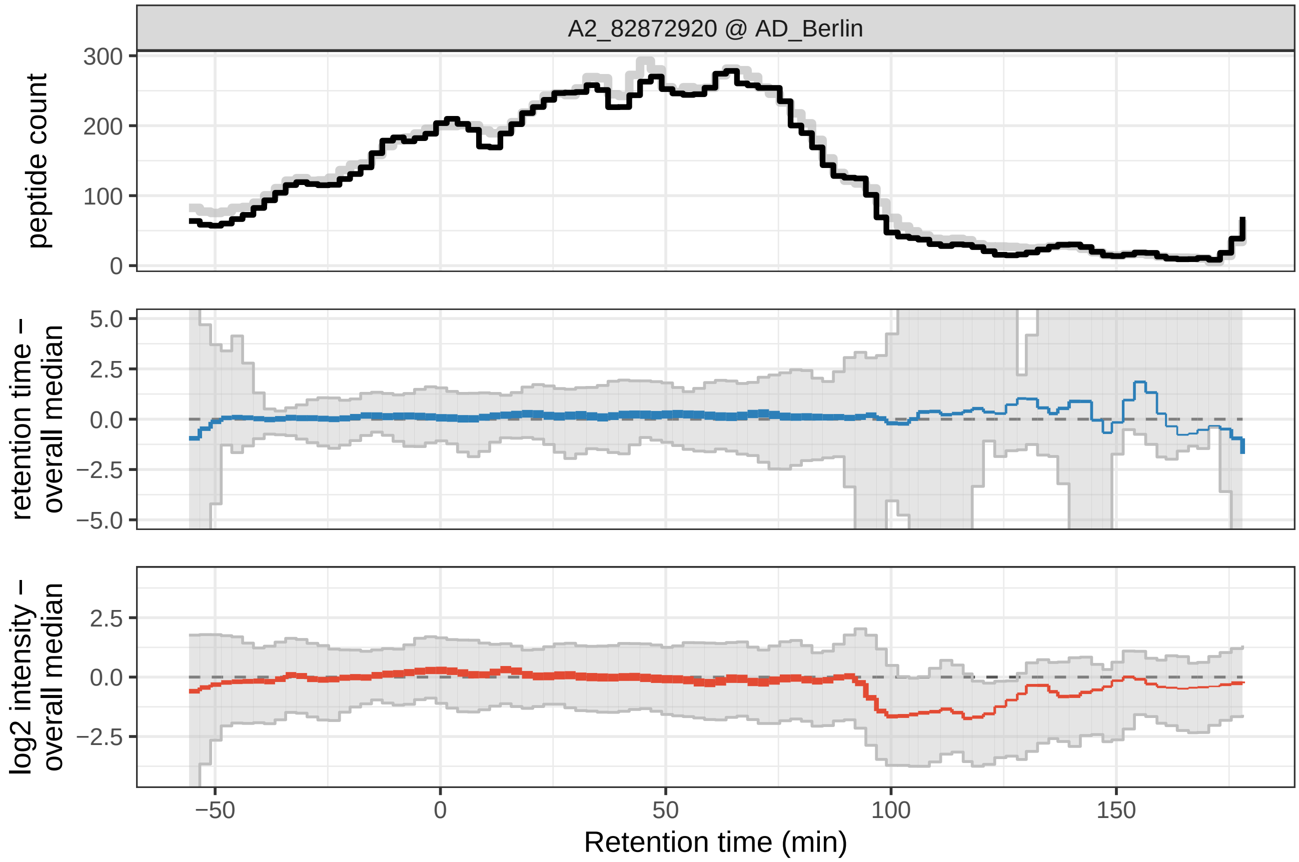
A2_82872920 @ AD_Berlin
peptide count
retention time – overall median
log2 intensity – overall median
Retention time (min)

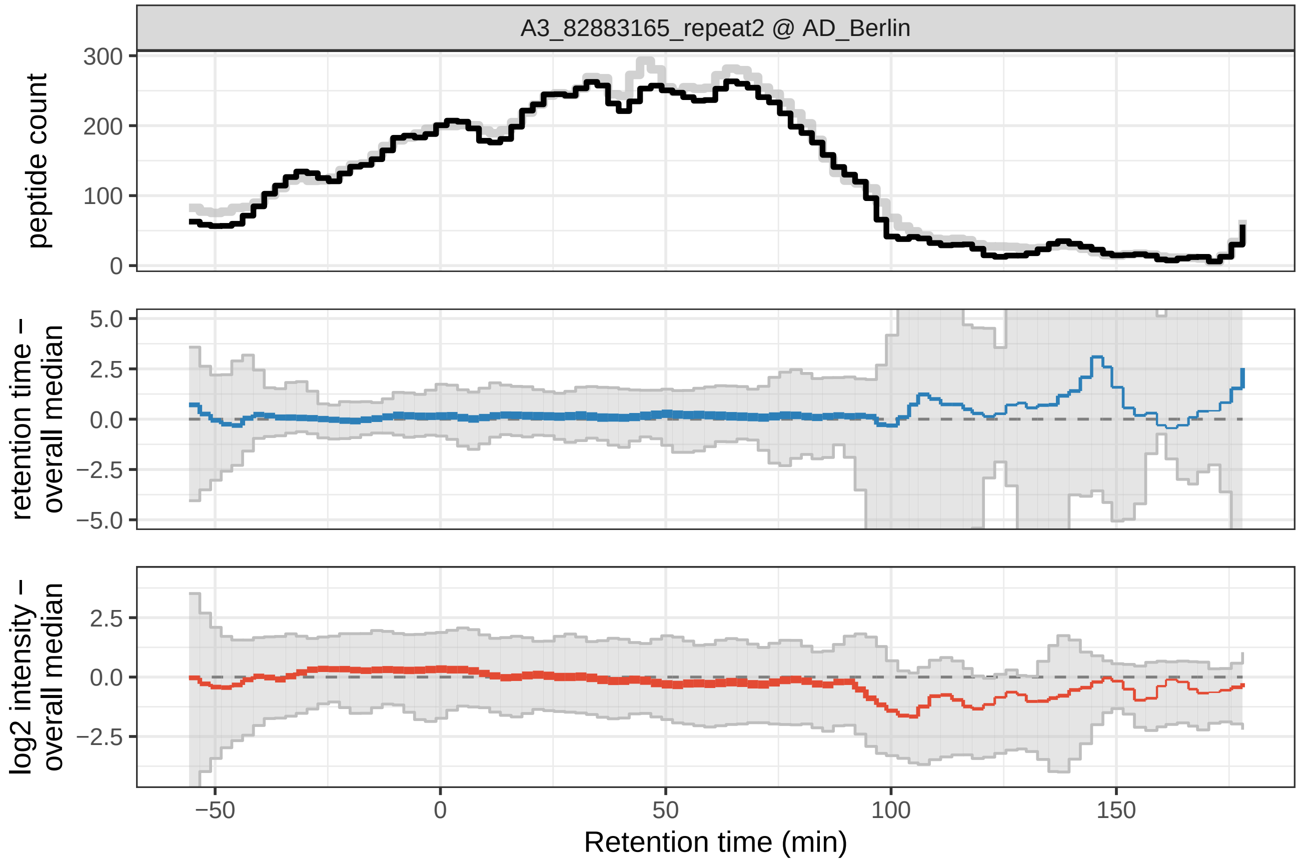
A3_82883165_repeat2 @ AD_Berlin
peptide count
retention time – overall median
log2 intensity – overall median
Retention time (min)

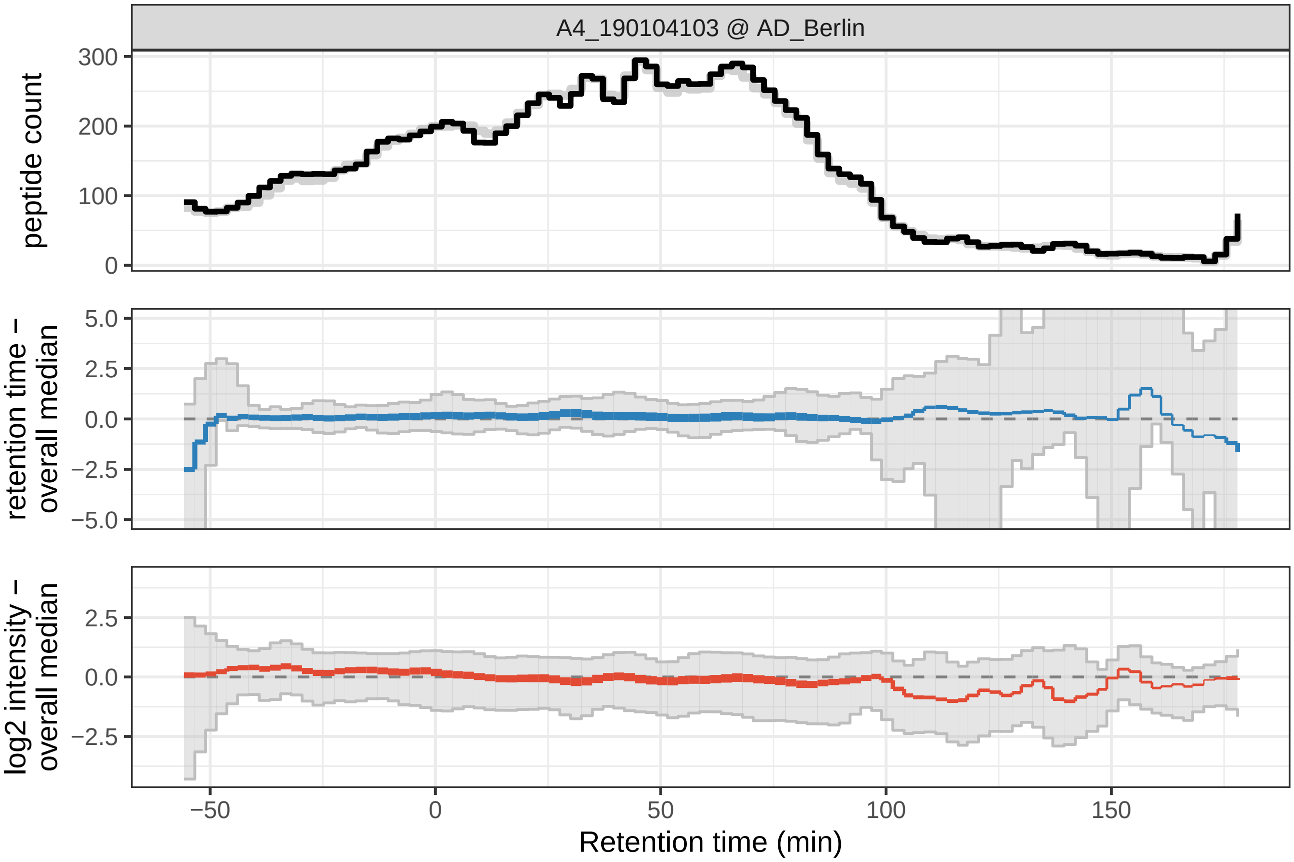
A4_190104103 @ AD_Berlin
peptide count
retention time – overall median
log2 intensity – overall median
Retention time (min)

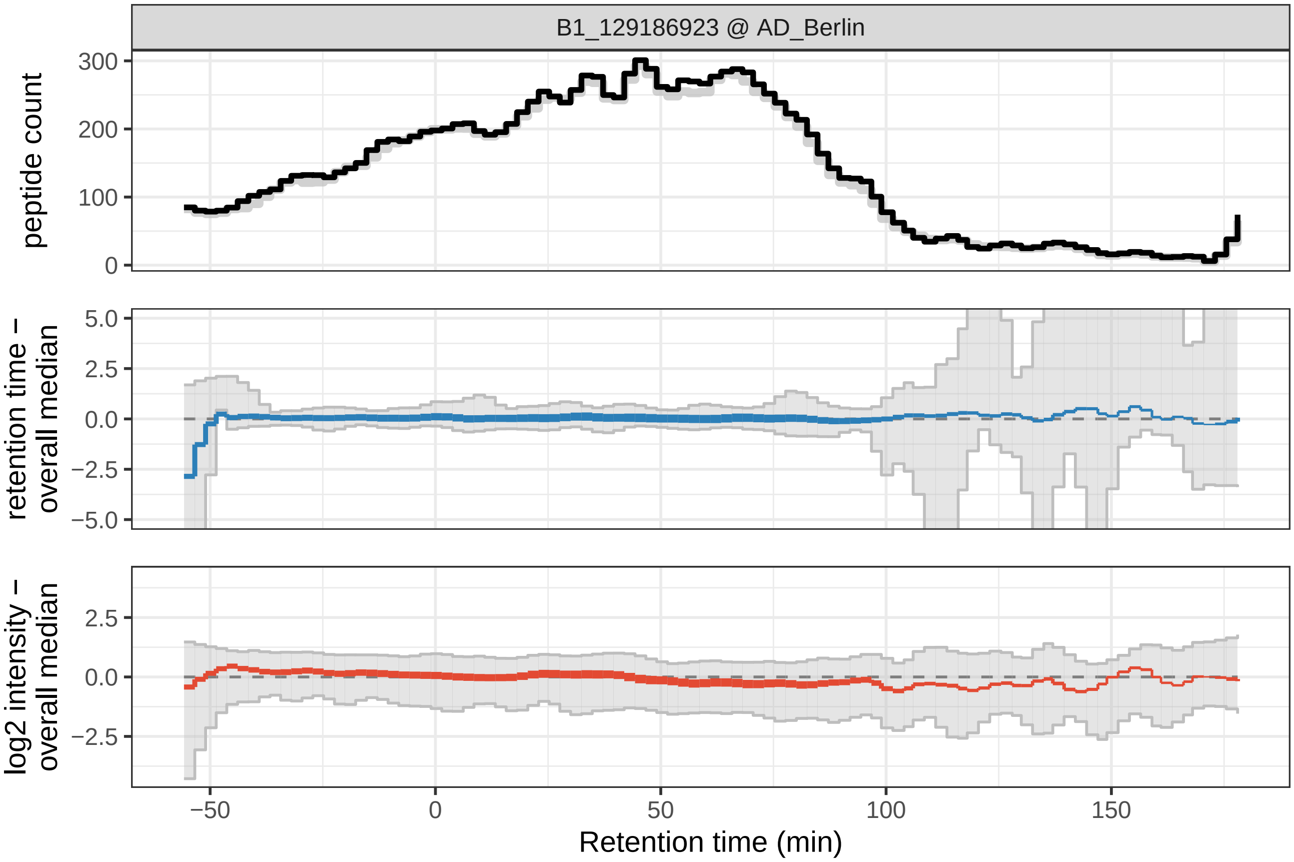
B1_129186923 @ AD_Berlin
peptide count
retention time – overall median
log2 intensity – overall median
Retention time (min)

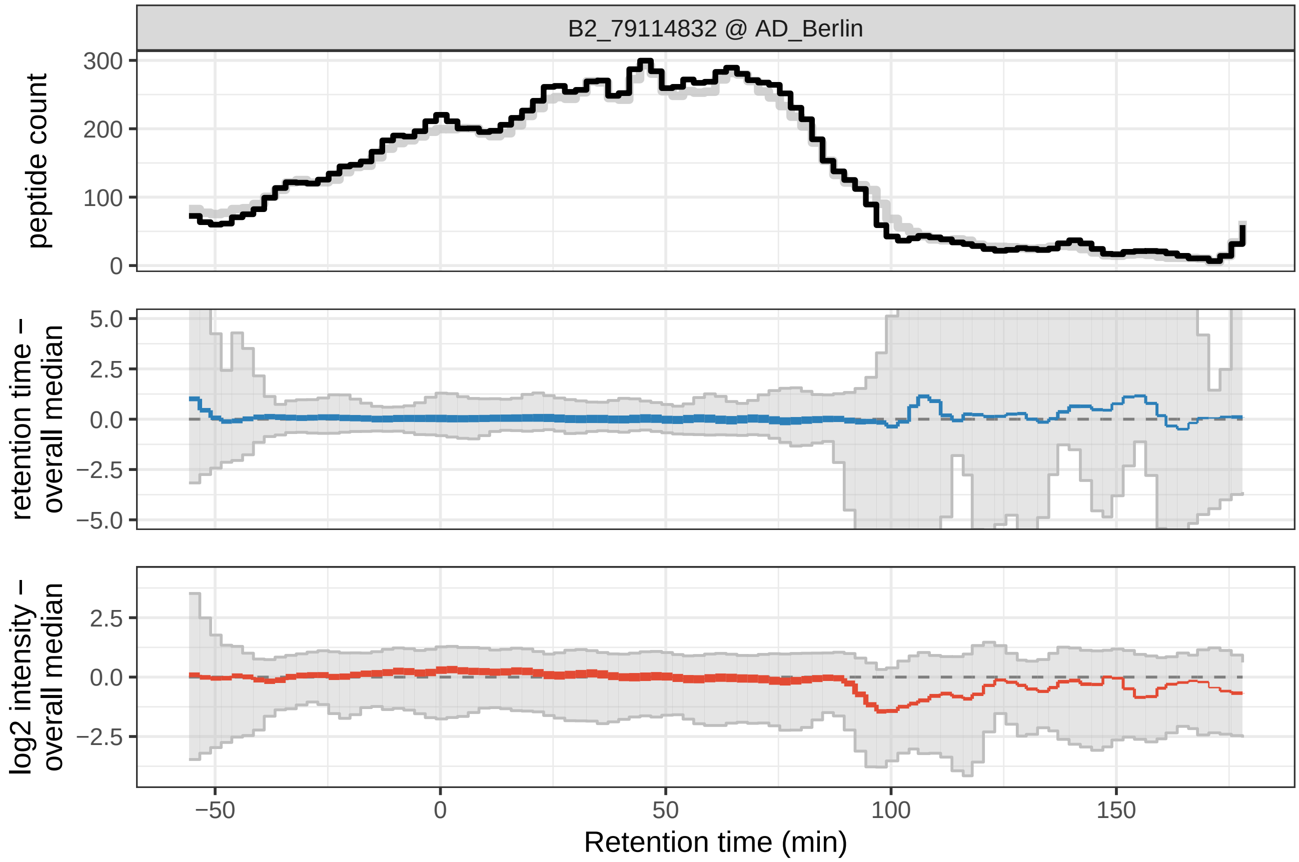
B2_79114832 @ AD_Berlin
peptide count
retention time – overall median
log2 intensity – overall median
Retention time (min)

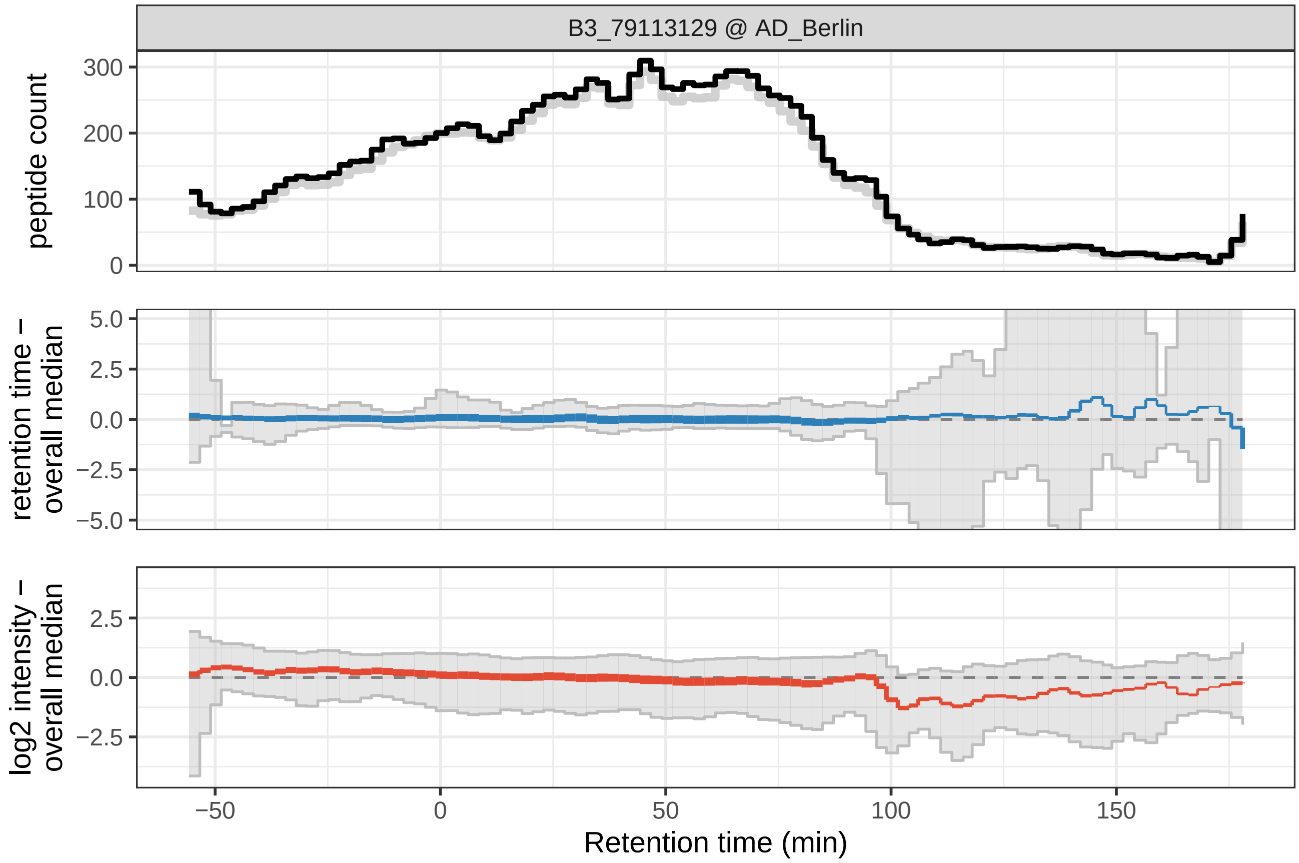
B3_79113129 @ AD_Berlin
peptide count
retention time – overall median
log2 intensity – overall median
Retention time (min)

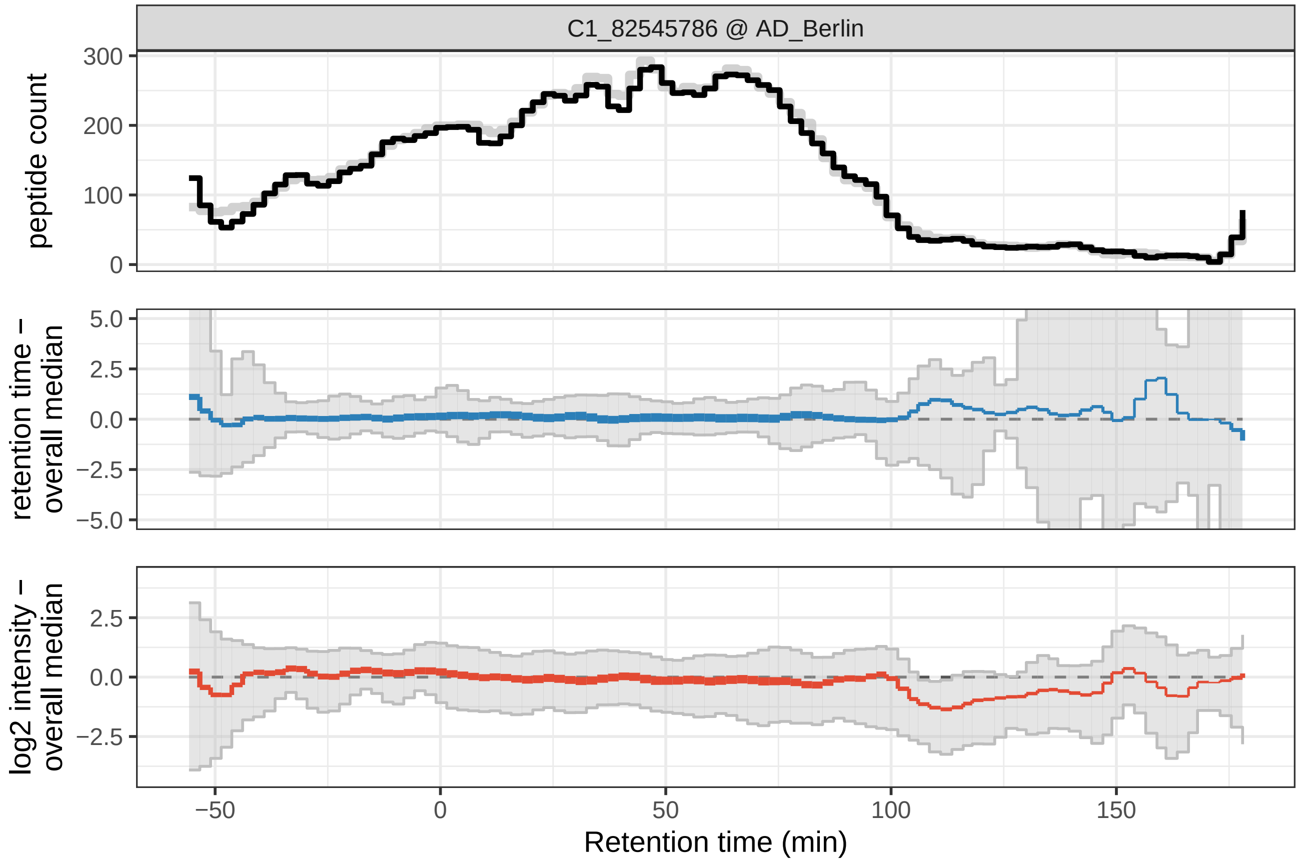
C1_82545786 @ AD_Berlin
peptide count
retention time – overall median
log2 intensity – overall median
Retention time (min)

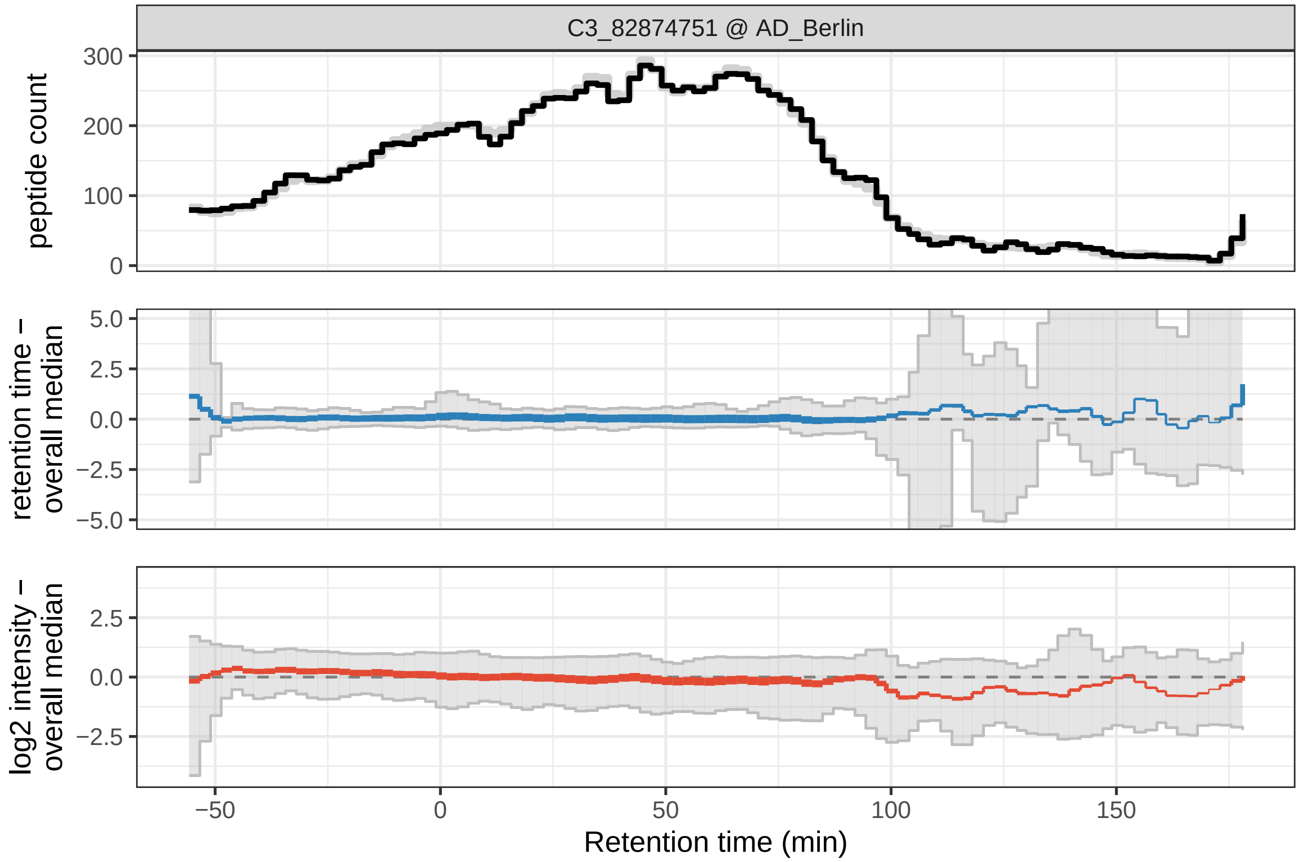
C3_82874751 @ AD_Berlin
peptide count
retention time – overall median
log2 intensity – overall median
Retention time (min)

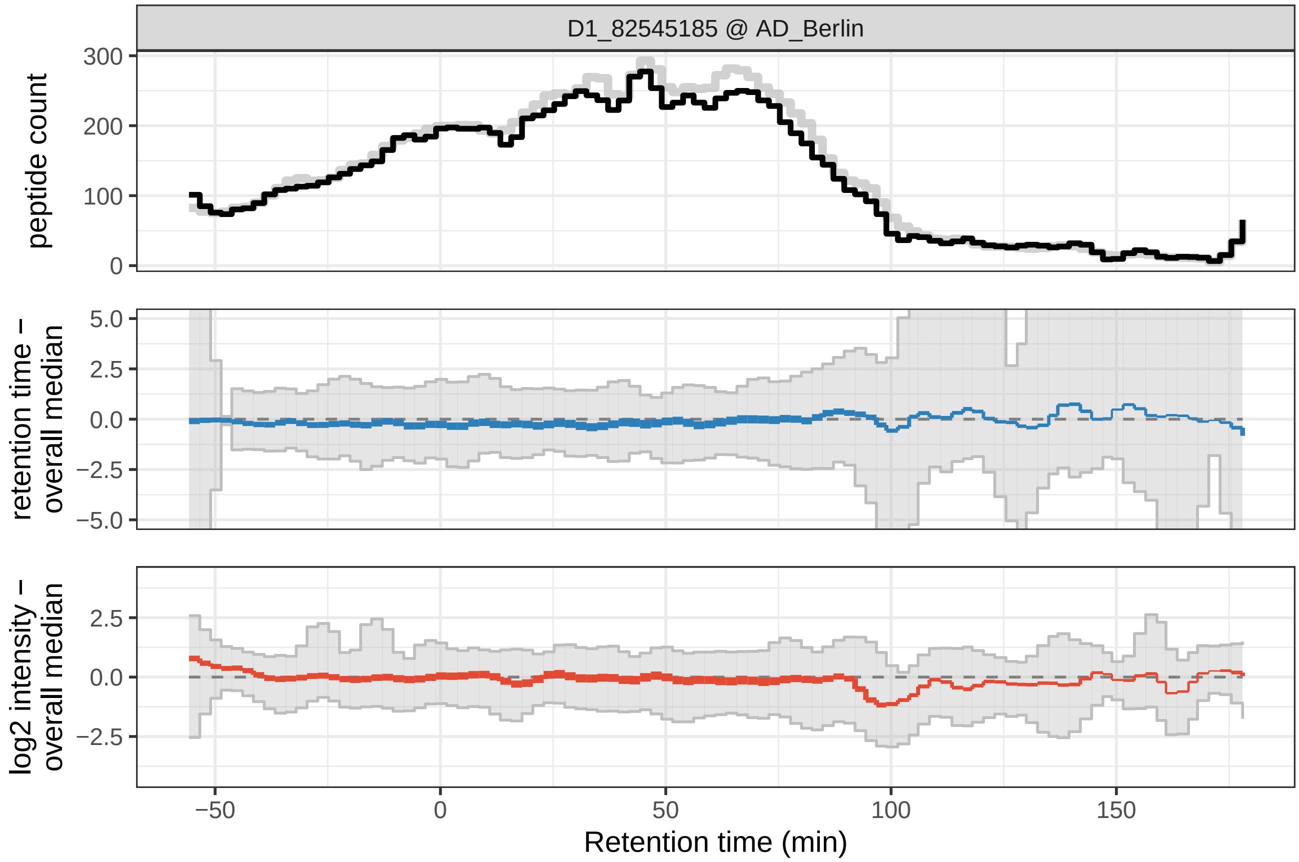
D1_82545185 @ AD_Berlin
peptide count
300
200
100
0
retention time –
overall median
5.0
2.5
0.0
−2.5
−5.0
log2 intensity –
overall median
2.5
0.0
−2.5
−50
0
50
100
150
Retention time (min)

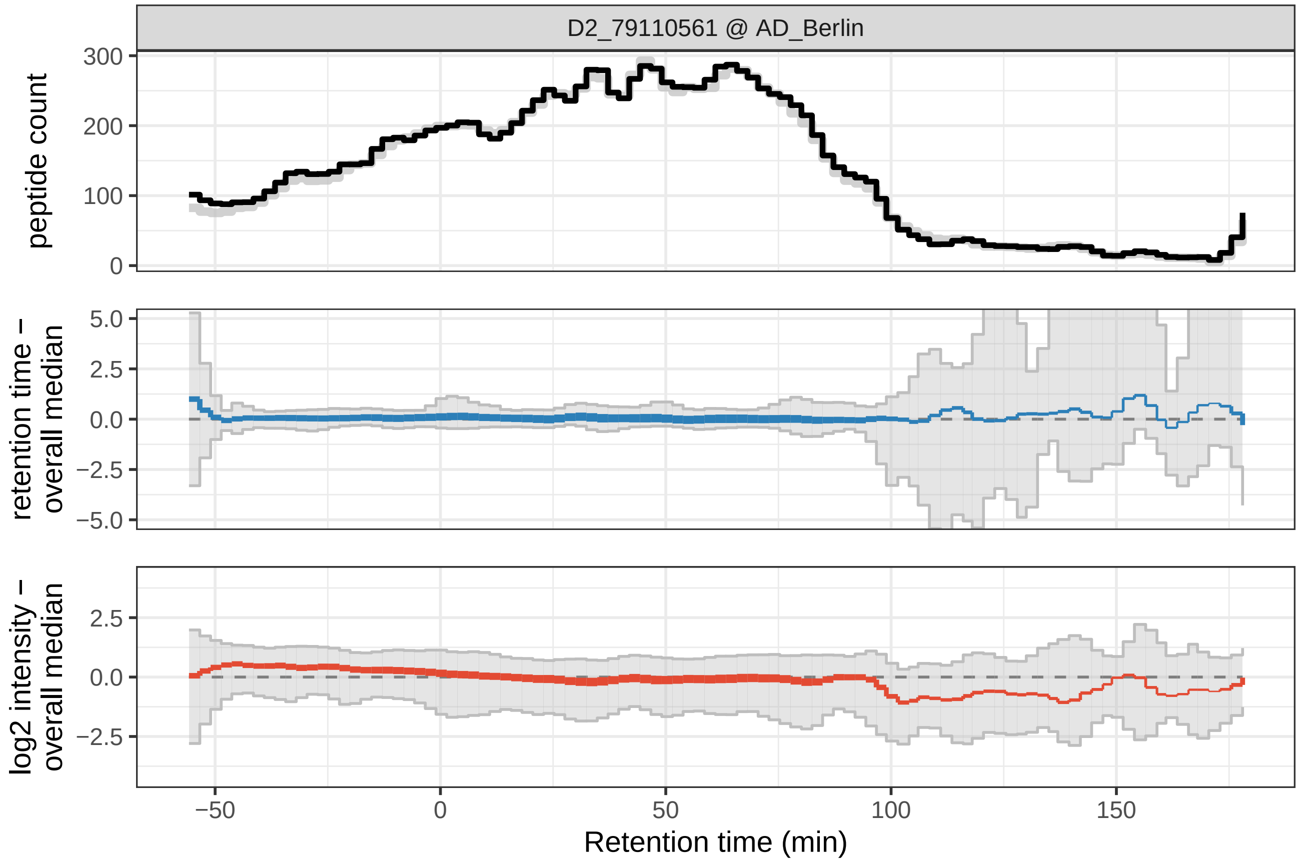
D2_79110561 @ AD_Berlin
peptide count
300
200
100
0
retention time –
overall median
5.0
2.5
0.0
−2.5
−5.0
log2 intensity –
overall median
2.5
0.0
−2.5
−50
0
50
100
150
Retention time (min)

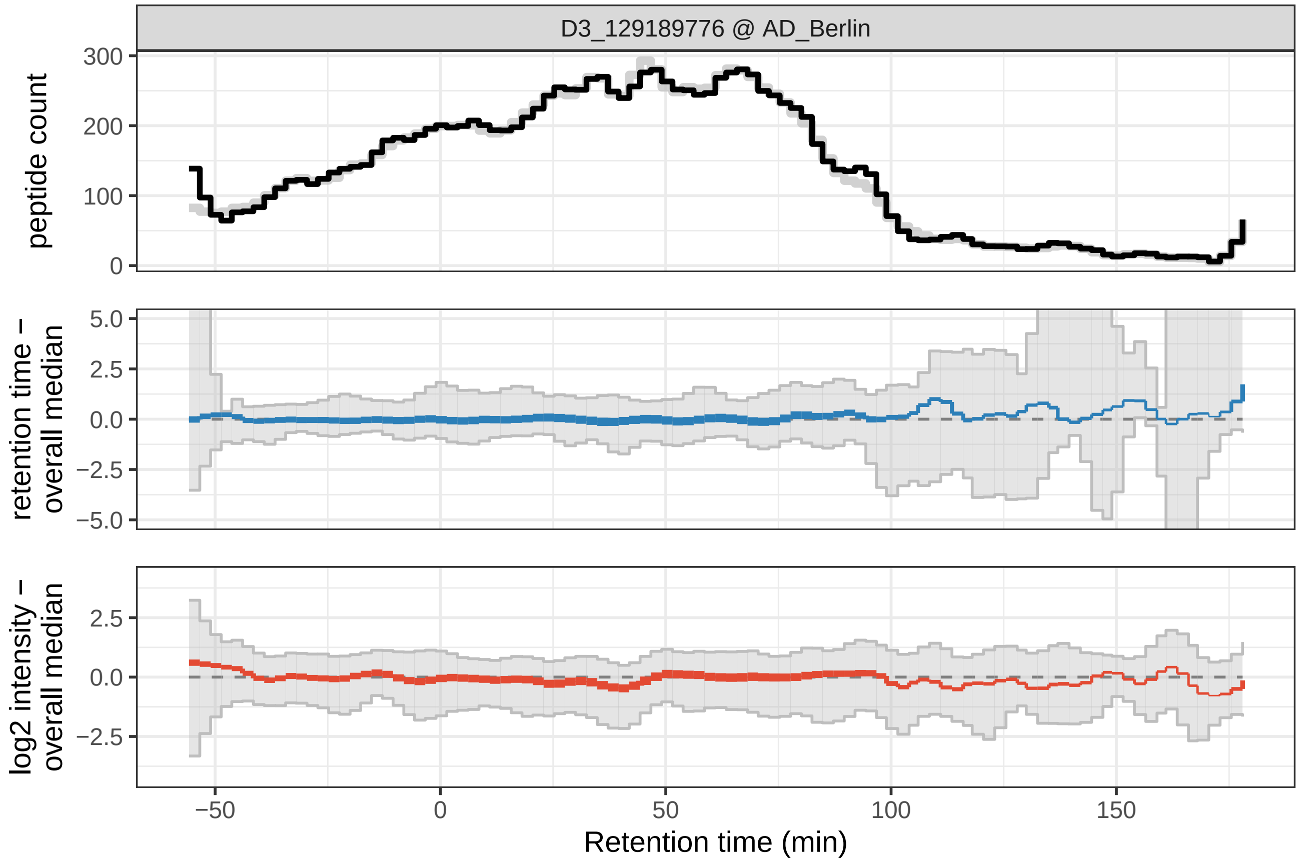
D3_129189776 @ AD_Berlin
peptide count
retention time – overall median
log2 intensity – overall median
Retention time (min)

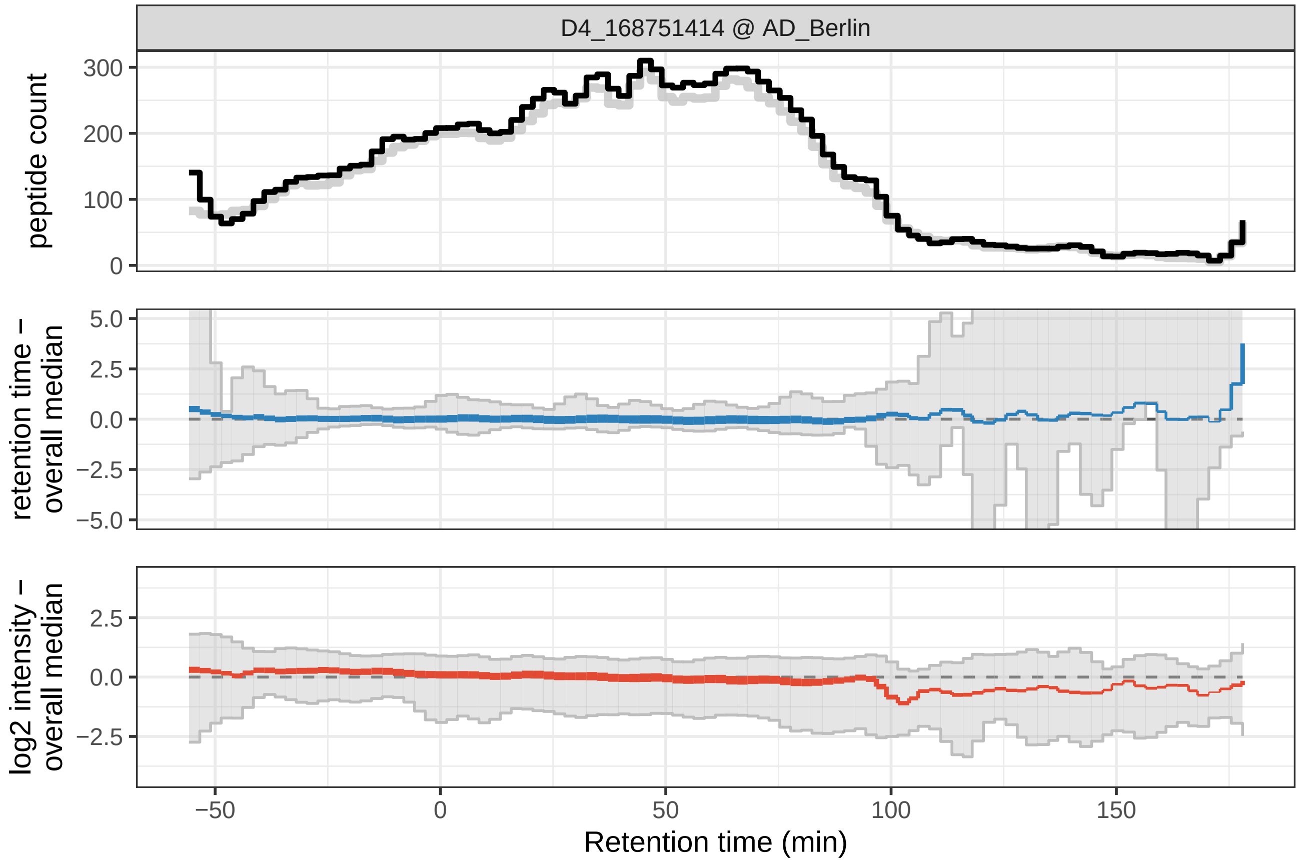
D4_168751414 @ AD_Berlin
peptide count
retention time – overall median
log2 intensity – overall median
Retention time (min)

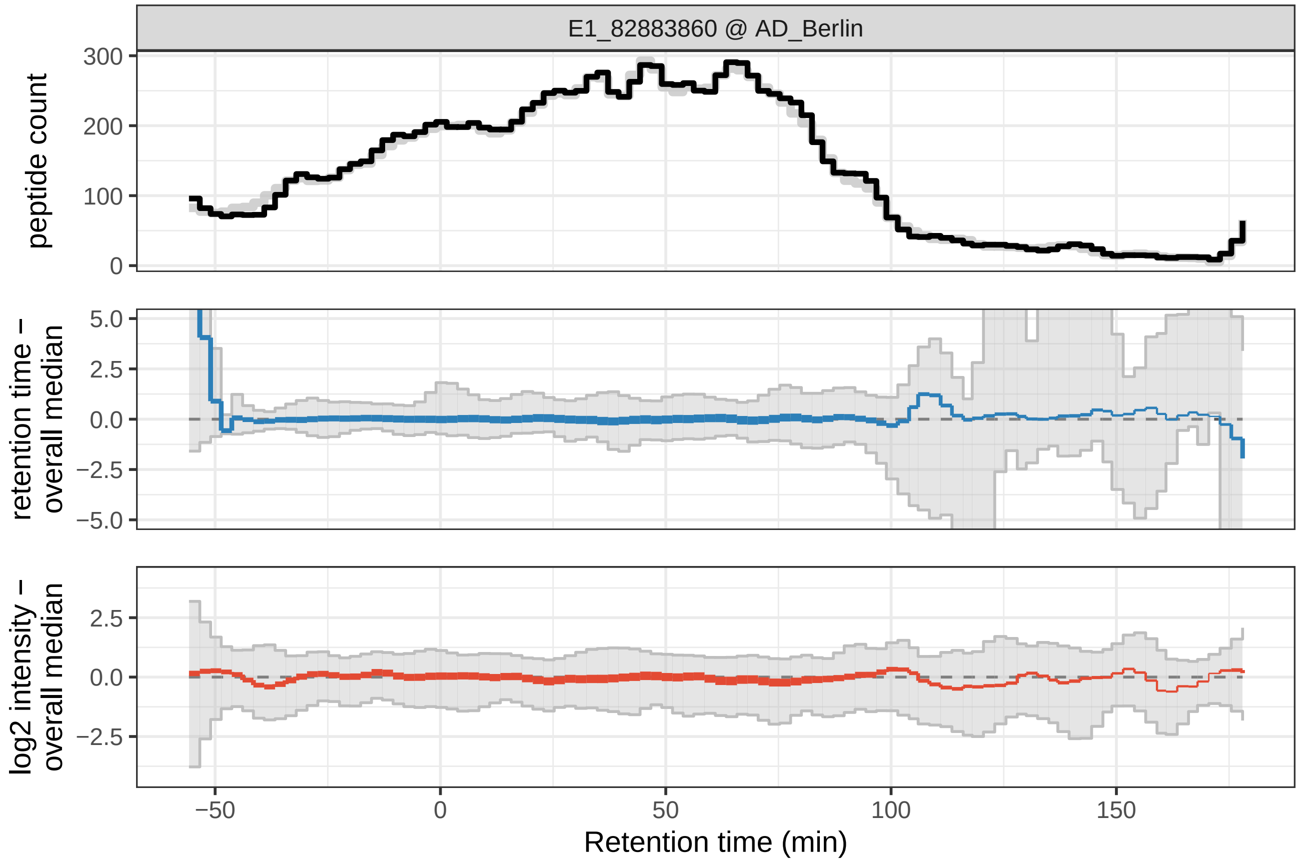
E1_82883860 @ AD_Berlin
peptide count
retention time – overall median
log2 intensity – overall median
Retention time (min)

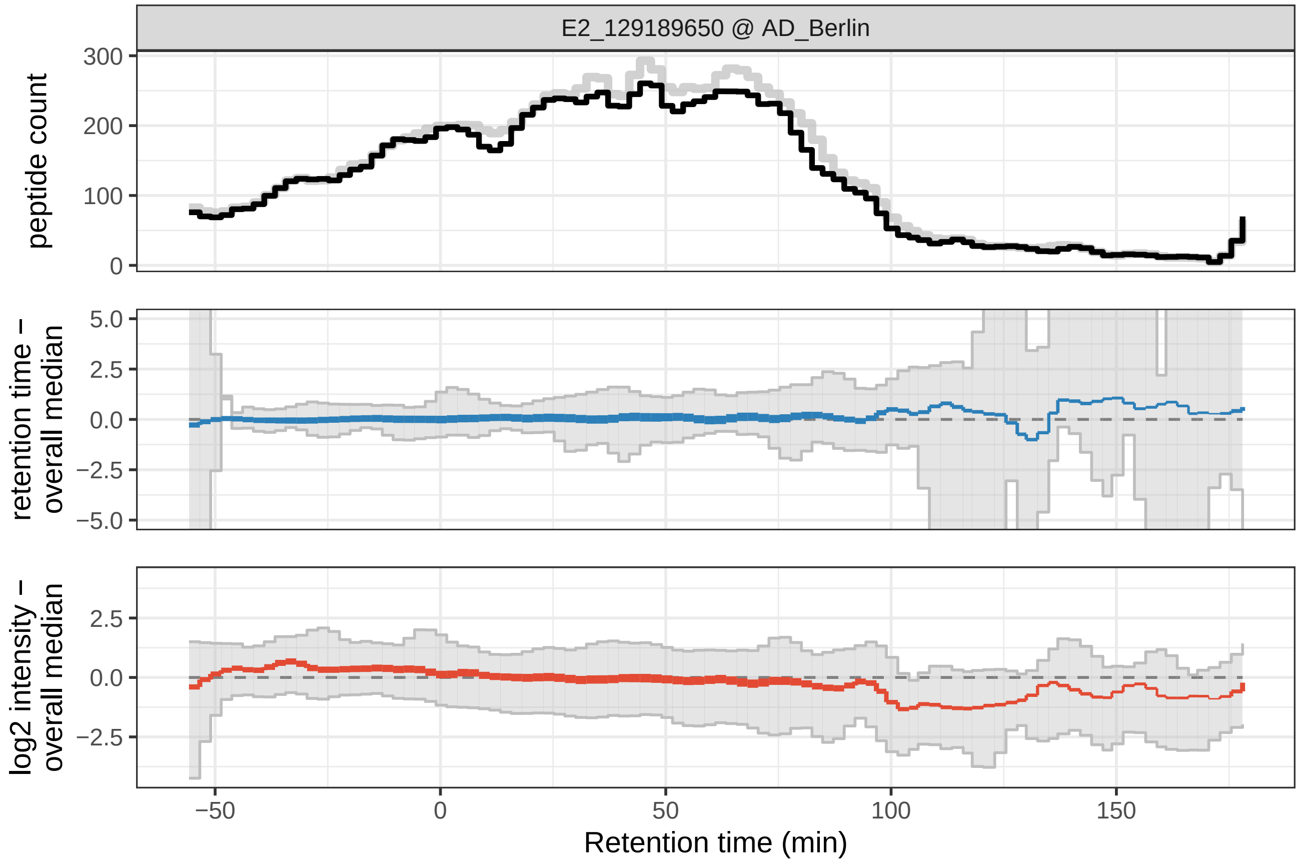
E2_129189650 @ AD_Berlin
peptide count
retention time – overall median
log2 intensity – overall median
Retention time (min)

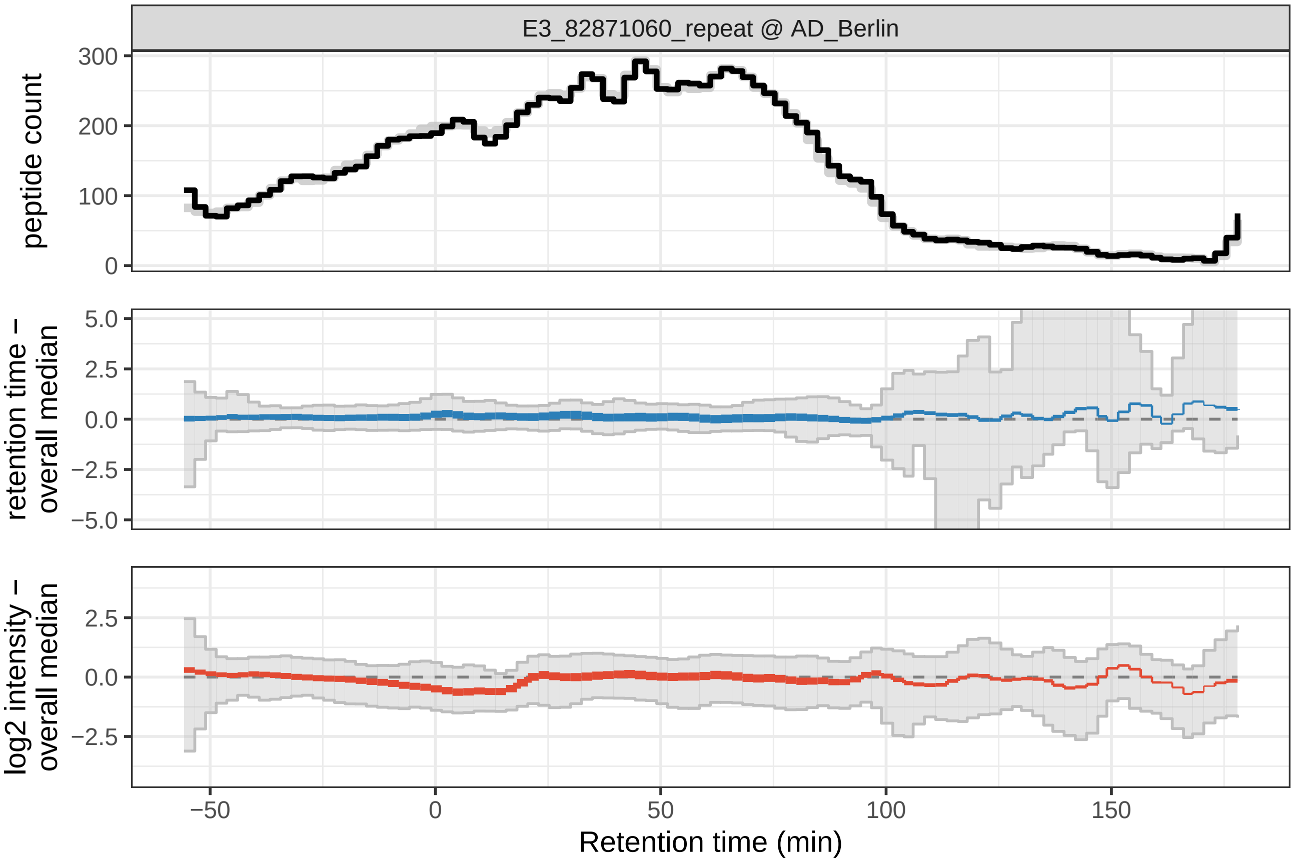
E3_82871060_repeat @ AD_Berlin
peptide count
retention time – overall median
log2 intensity – overall median
Retention time (min)

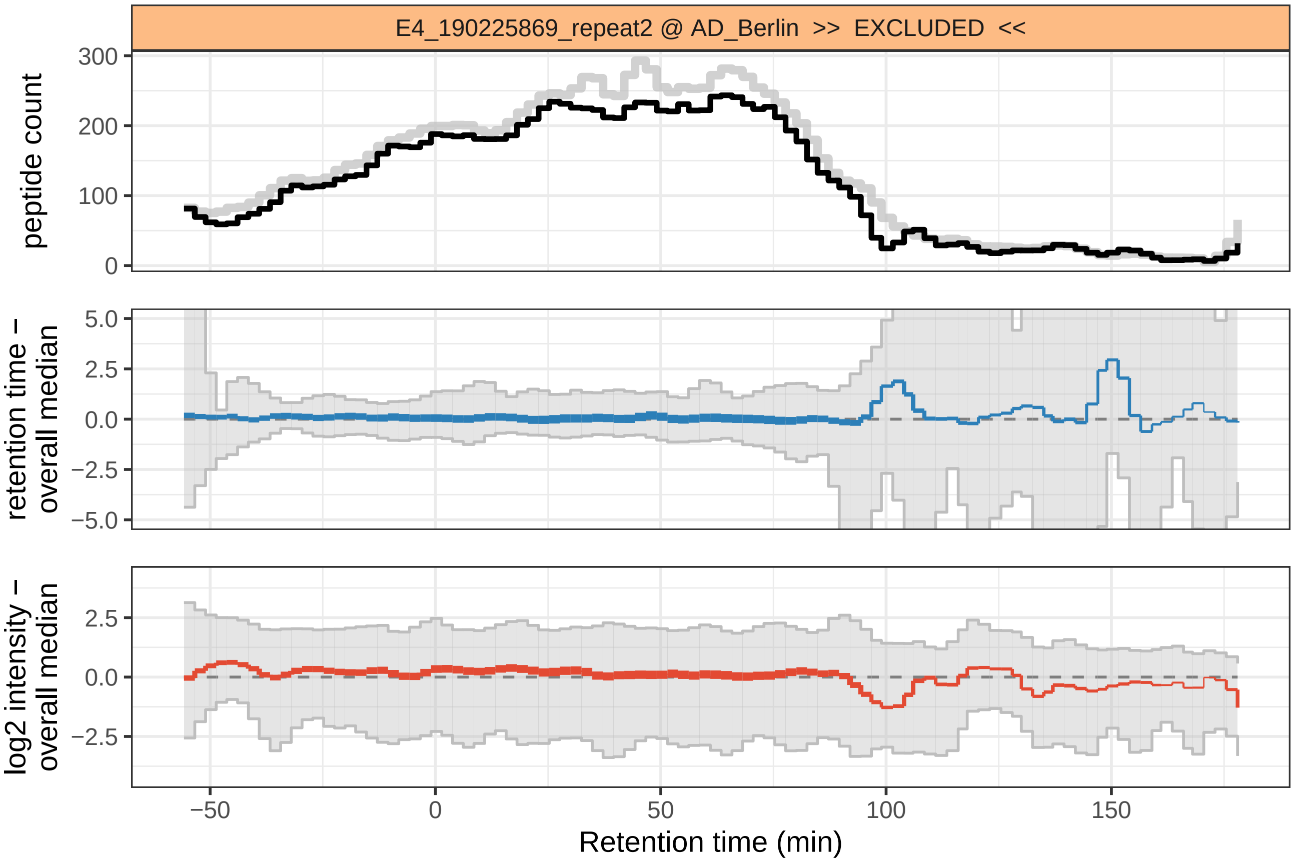
E4_190225869_repeat2 @ AD_Berlin >> EXCLUDED <<
peptide count
retention time – overall median
log2 intensity – overall median
Retention time (min)

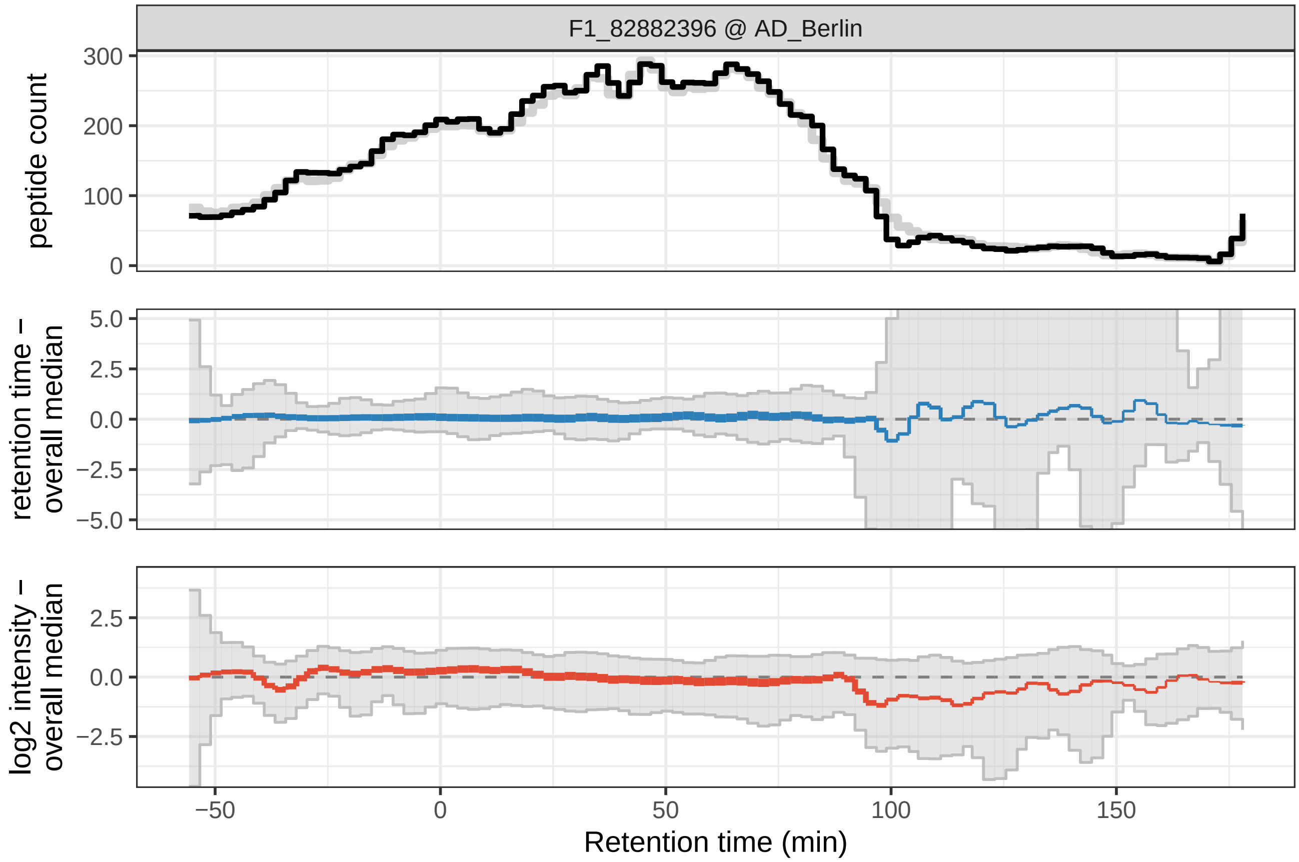
F1_82882396 @ AD_Berlin
peptide count
retention time – overall median
log2 intensity – overall median
Retention time (min)

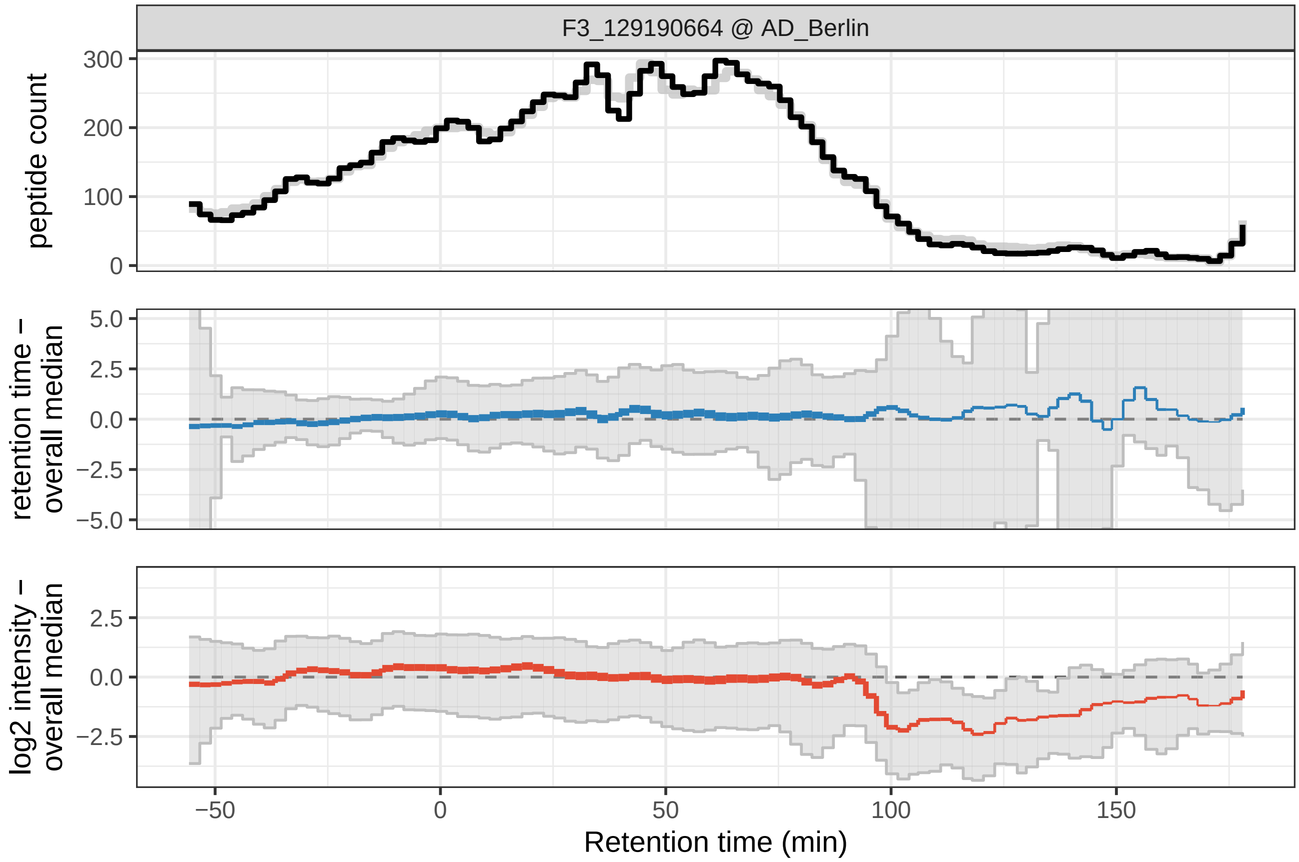
F3_129190664 @ AD_Berlin
peptide count
retention time – overall median
log2 intensity – overall median
Retention time (min)

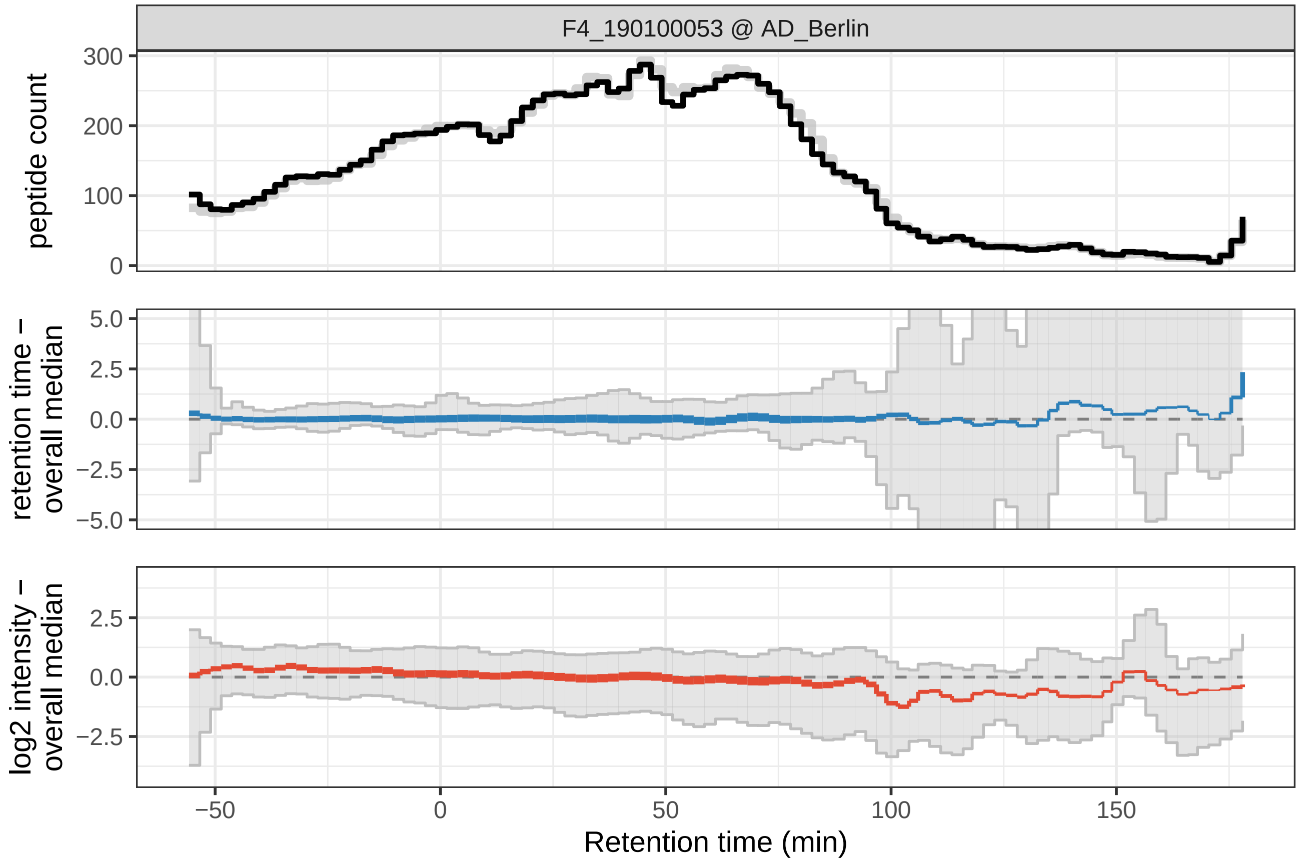
F4_190100053 @ AD_Berlin
peptide count
retention time – overall median
log2 intensity – overall median
Retention time (min)

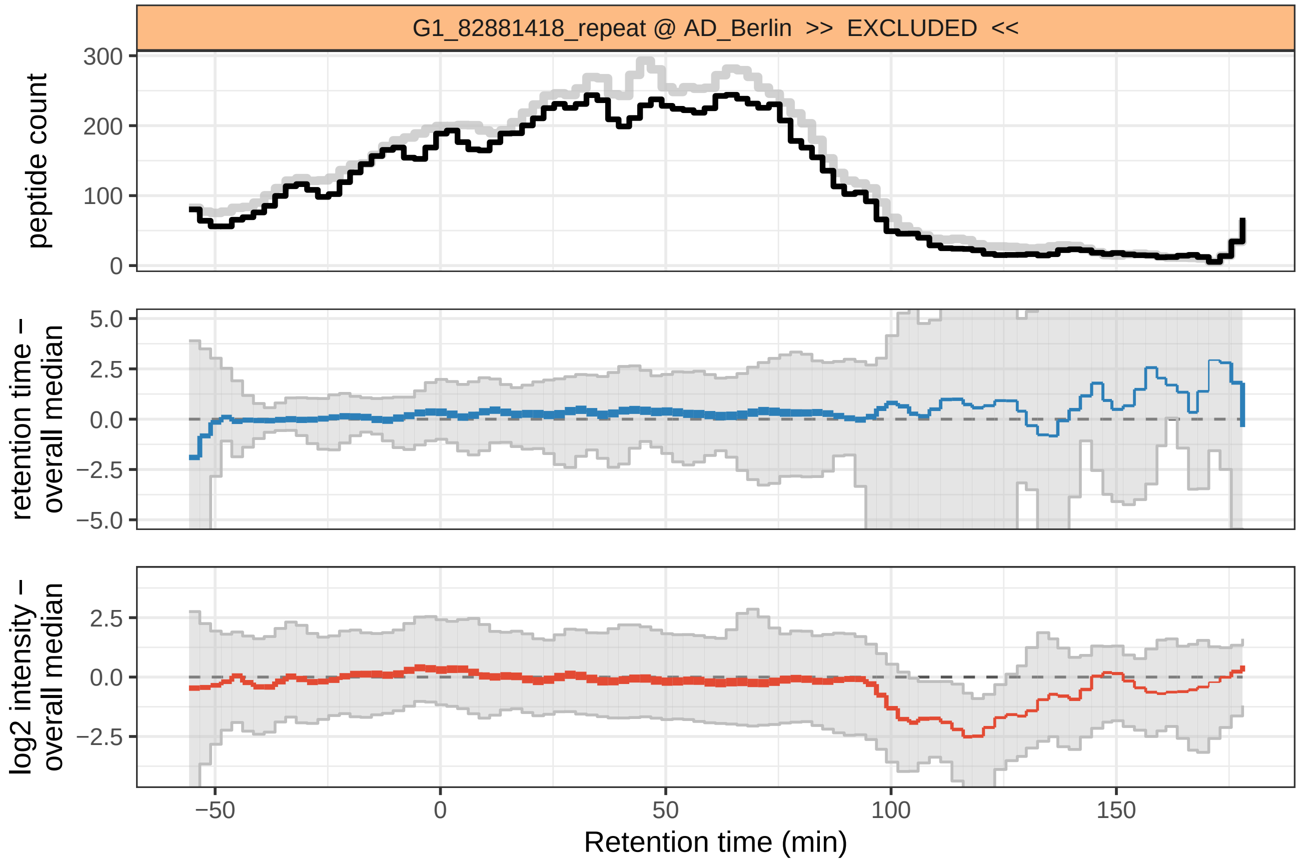
G1_82881418_repeat @ AD_Berlin >> EXCLUDED <<
peptide count
retention time – overall median
log2 intensity – overall median
Retention time (min)

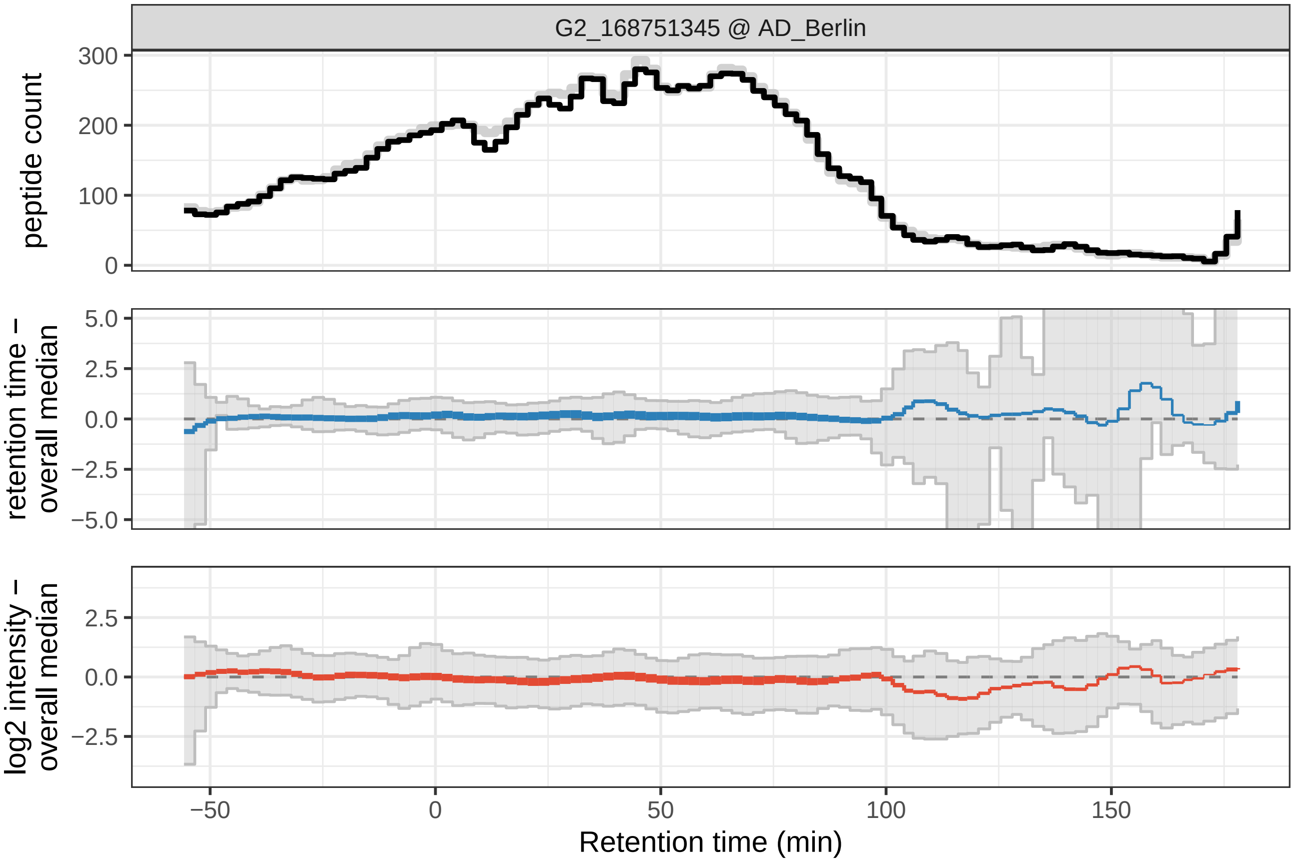

G2_168751345 @ AD_Berlin
peptide count
retention time – overall median
log2 intensity – overall median
Retention time (min)

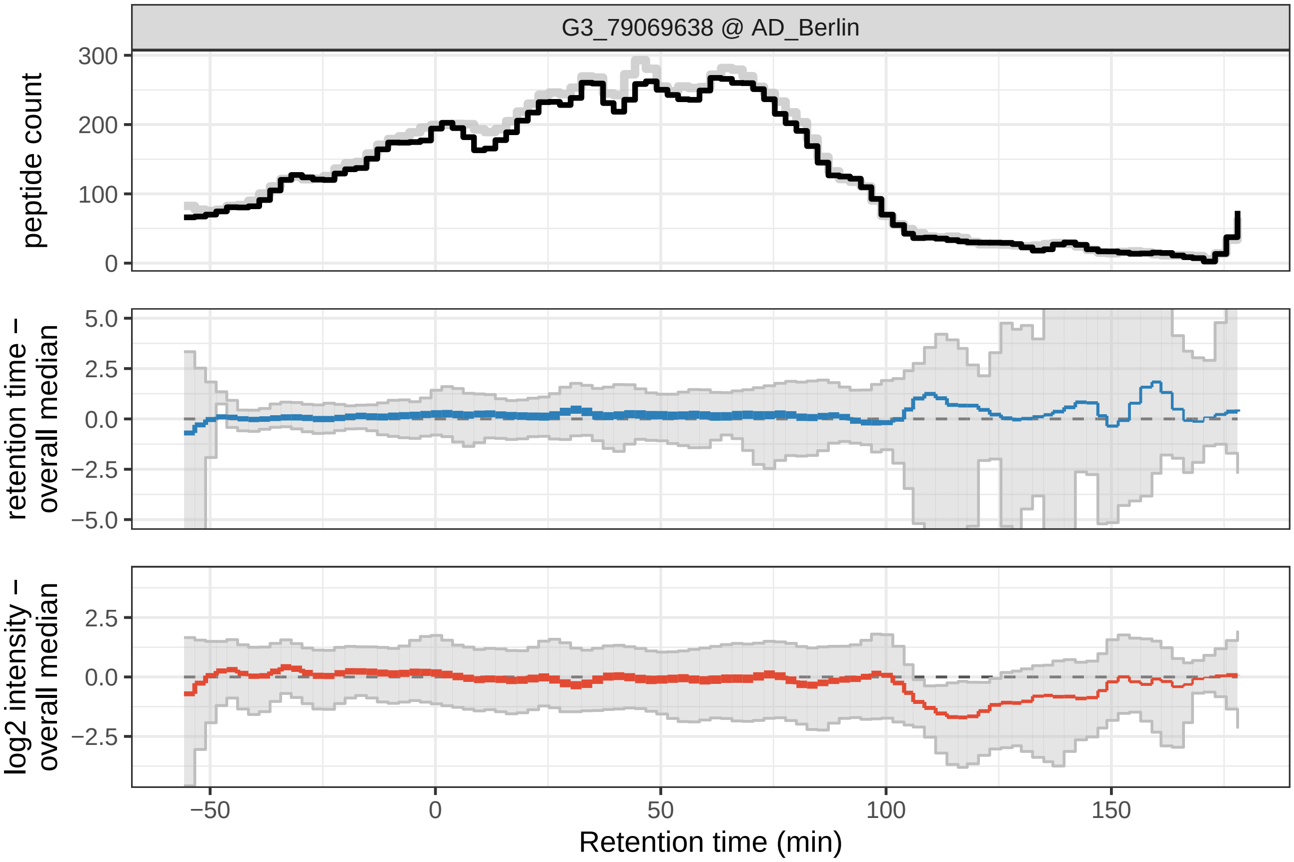

G3_79069638 @ AD_Berlin
peptide count
retention time – overall median
log2 intensity – overall median
Retention time (min)

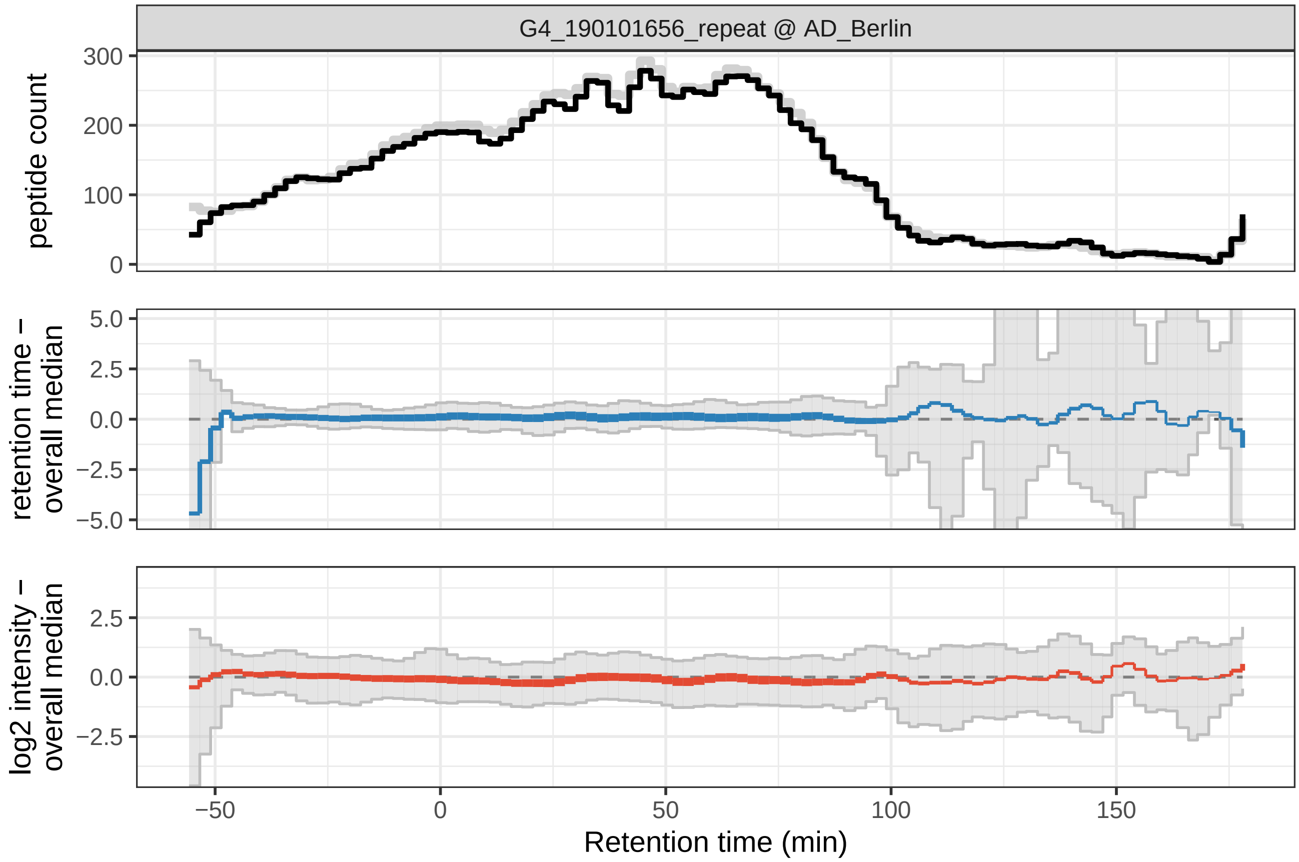
G4_190101656_repeat @ AD_Berlin
peptide count
300
200
100
0
retention time –
overall median
5.0
2.5
0.0
−2.5
−5.0
log2 intensity –
overall median
2.5
0.0
−2.5
−50
0
50
100
150
Retention time (min)

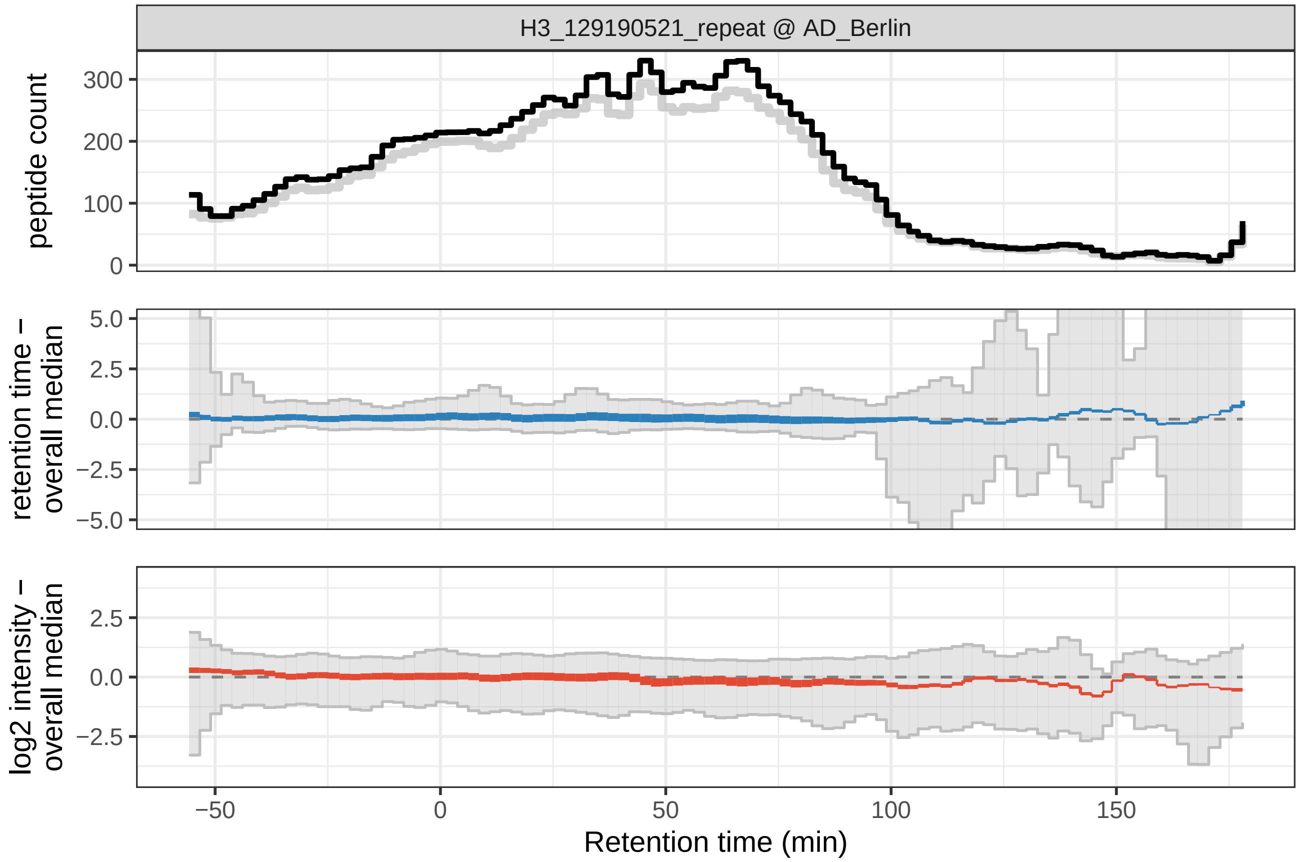
H3_129190521_repeat @ AD_Berlin
peptide count
300
200
100
0
retention time –
overall median
5.0
2.5
0.0
−2.5
−5.0
log2 intensity –
overall median
2.5
0.0
−2.5
−50
0
50
100
150
Retention time (min)

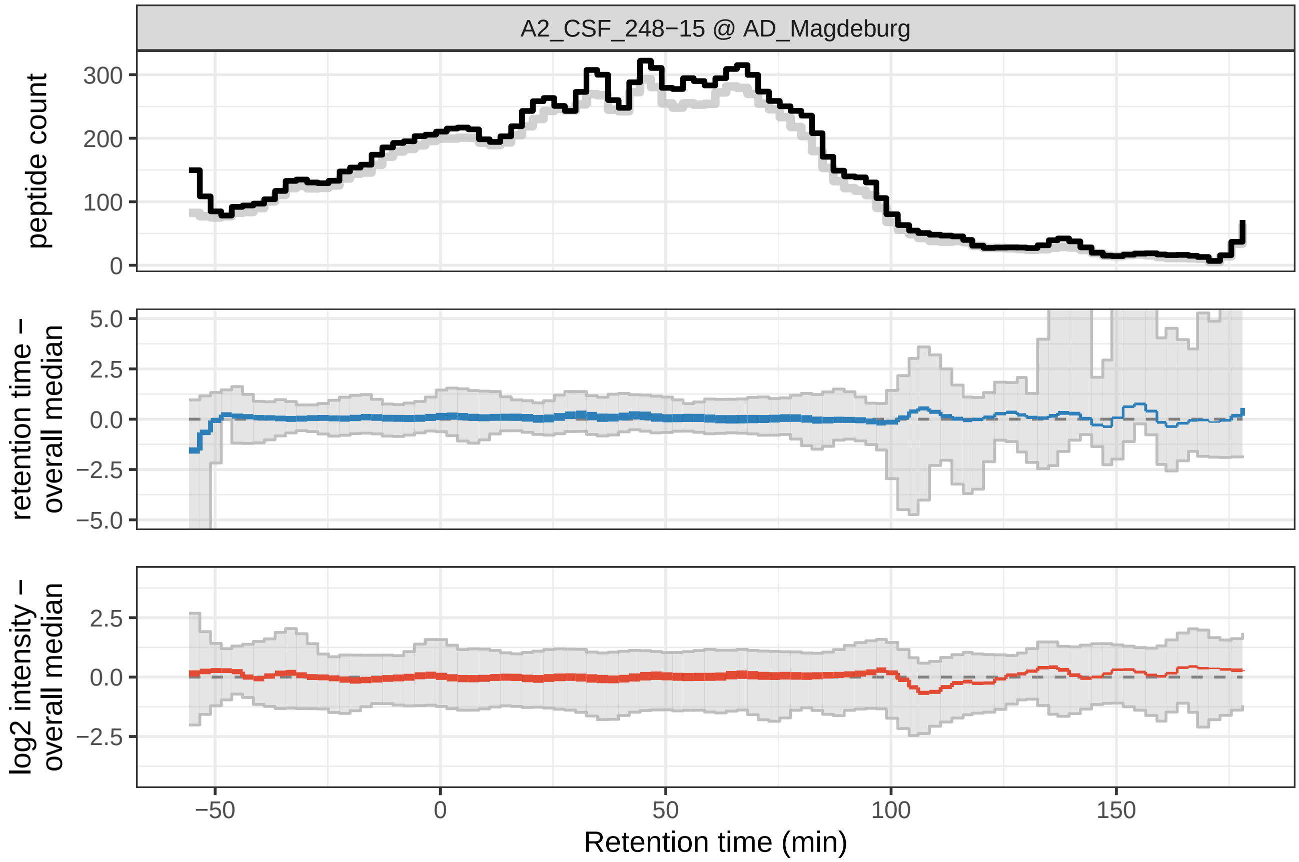

A2_CSF_248−15 @ AD_Magdeburg
peptide count
300
200
100
0
retention time –
overall median
5.0
2.5
0.0
−2.5
−5.0
log2 intensity –
overall median
2.5
0.0
−2.5
−50
0
50
100
150
Retention time (min)

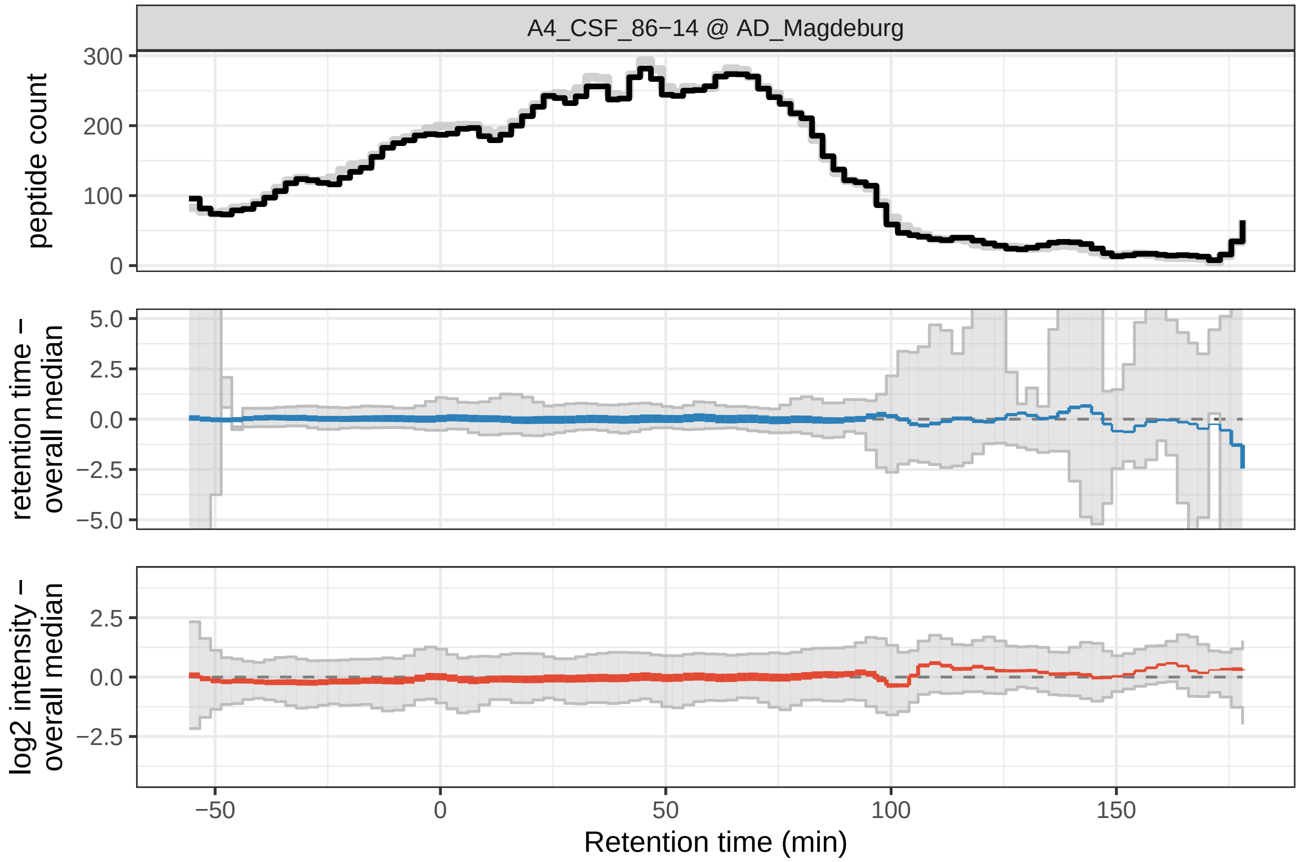

A4_CSF_86−14 @ AD_Magdeburg
peptide count
300
200
100
0
retention time –
overall median
5.0
2.5
0.0
−2.5
−5.0
log2 intensity –
overall median
2.5
0.0
−2.5
−50
0
50
100
150
Retention time (min)

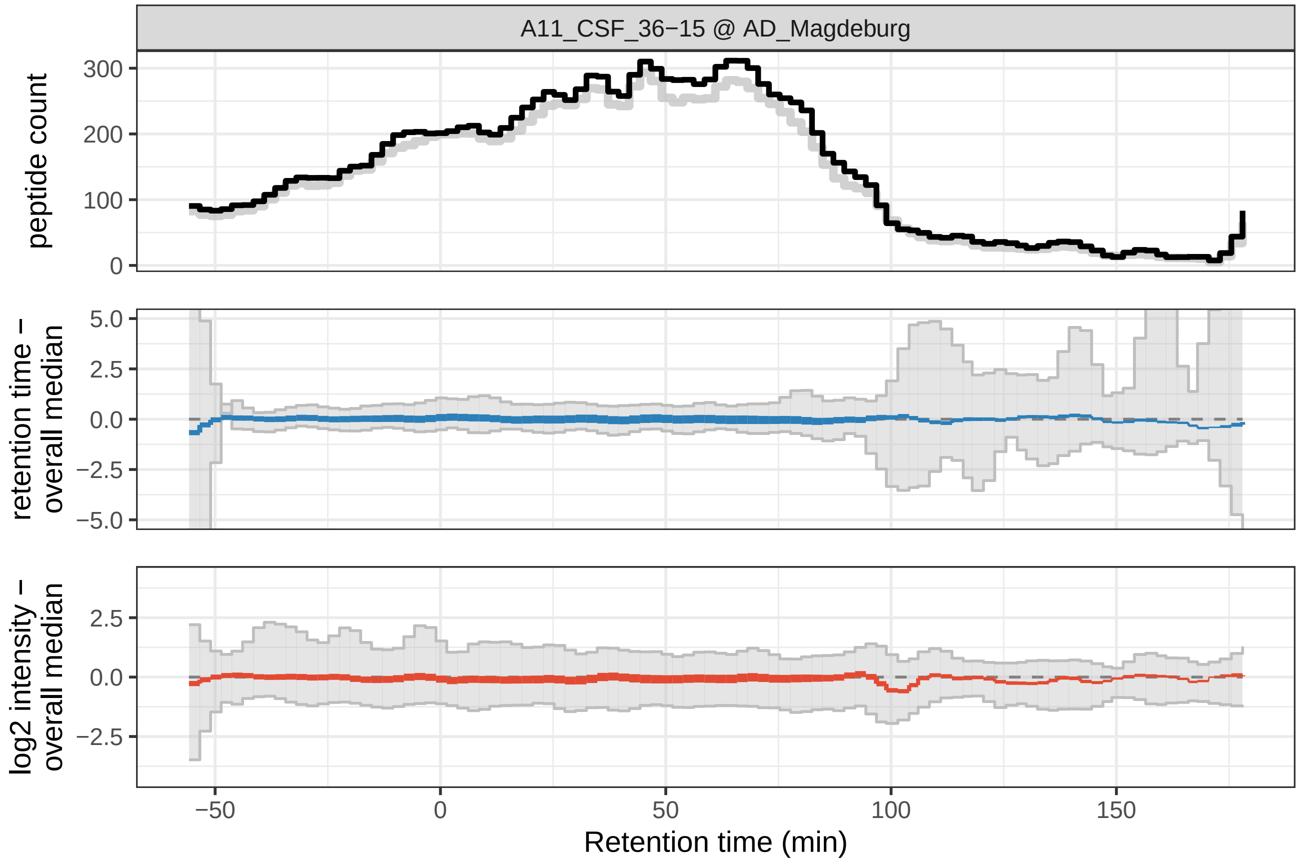

A11_CSF_36−15 @ AD_Magdeburg
peptide count
300
200
100
0
retention time –
overall median
5.0
2.5
0.0
−2.5
−5.0
log2 intensity –
overall median
2.5
0.0
−2.5
−50
0
50
100
150
Retention time (min)

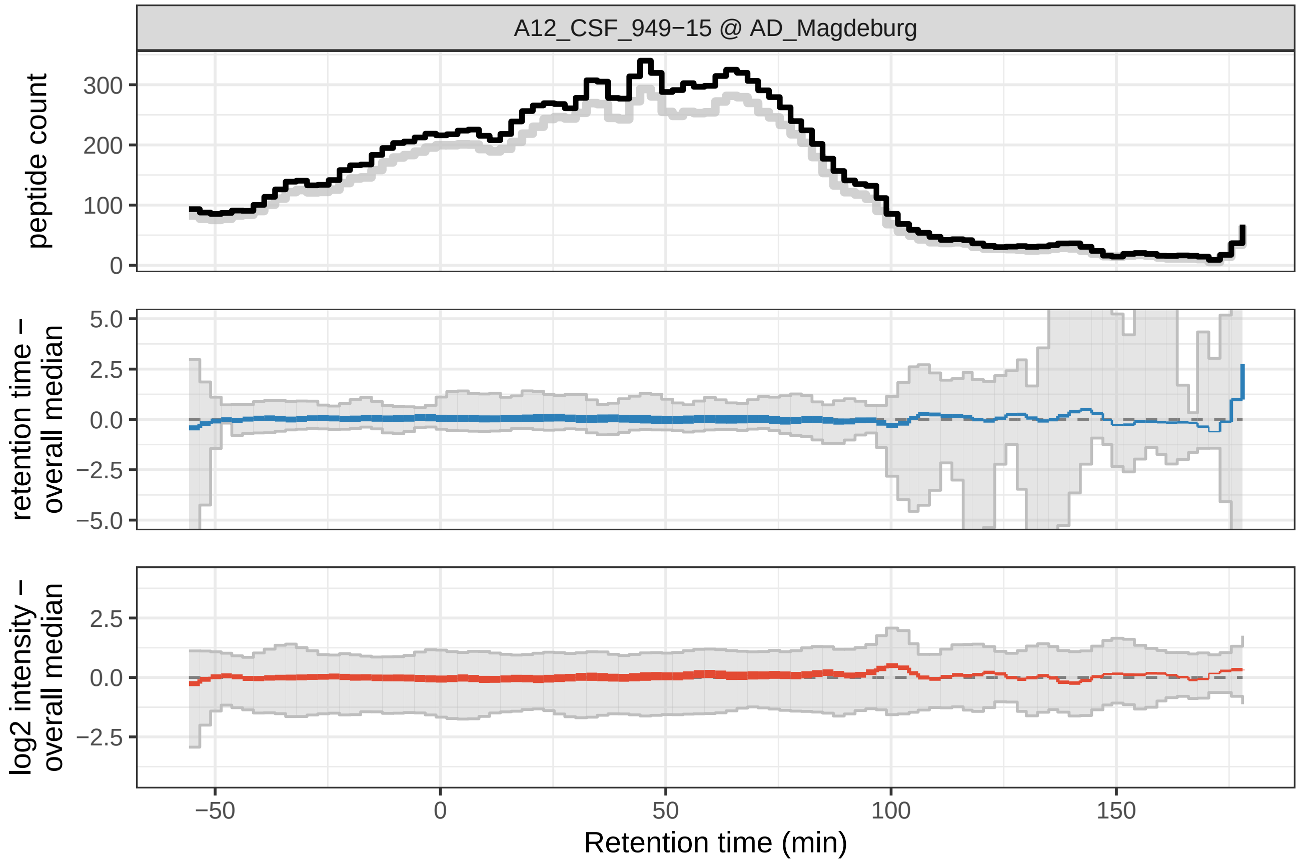

A12_CSF_949−15 @ AD_Magdeburg
peptide count
300
200
100
0
retention time –
overall median
5.0
2.5
0.0
−2.5
−5.0
log2 intensity –
overall median
2.5
0.0
−2.5
−50
0
50
100
150
Retention time (min)

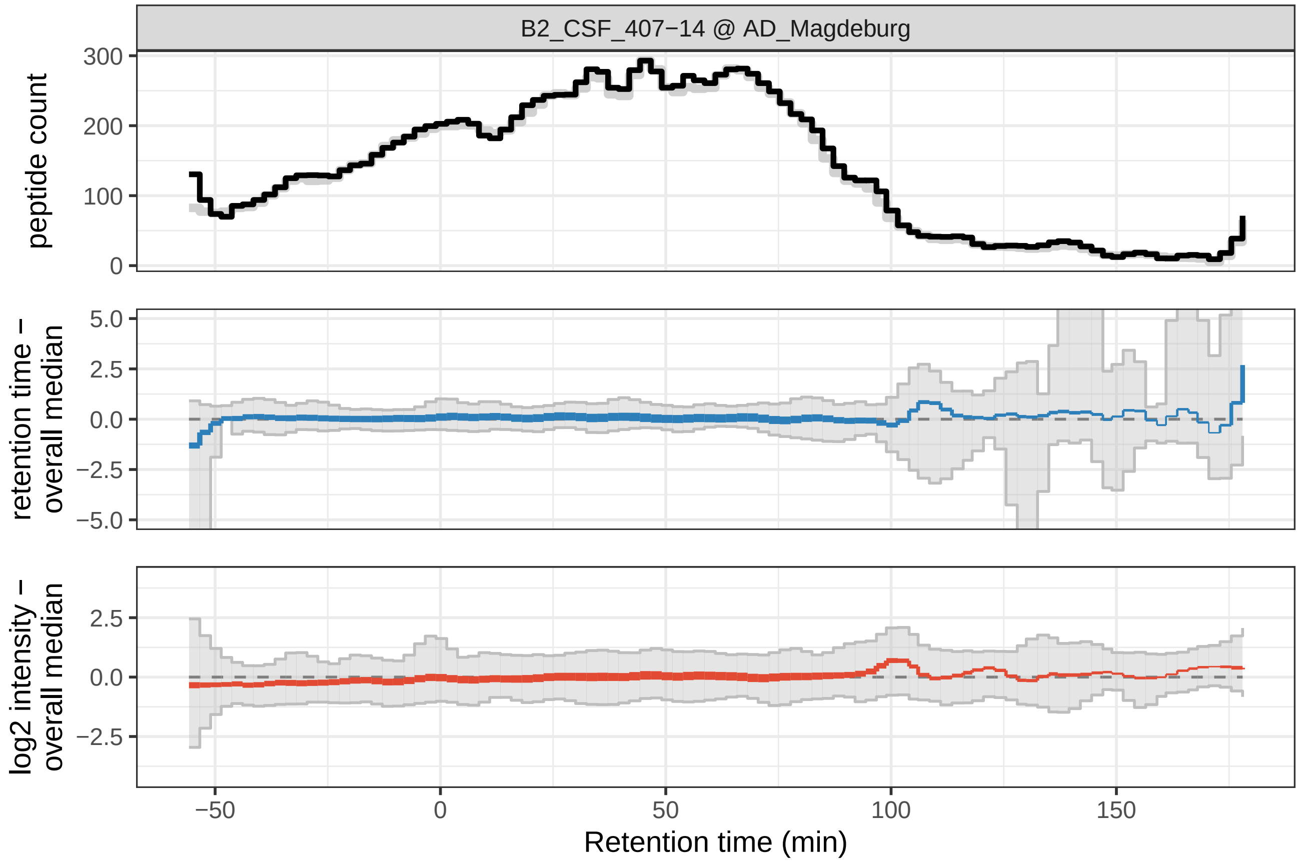
B2_CSF_407−14 @ AD_Magdeburg
peptide count
retention time –
overall median
log2 intensity –
overall median
Retention time (min)

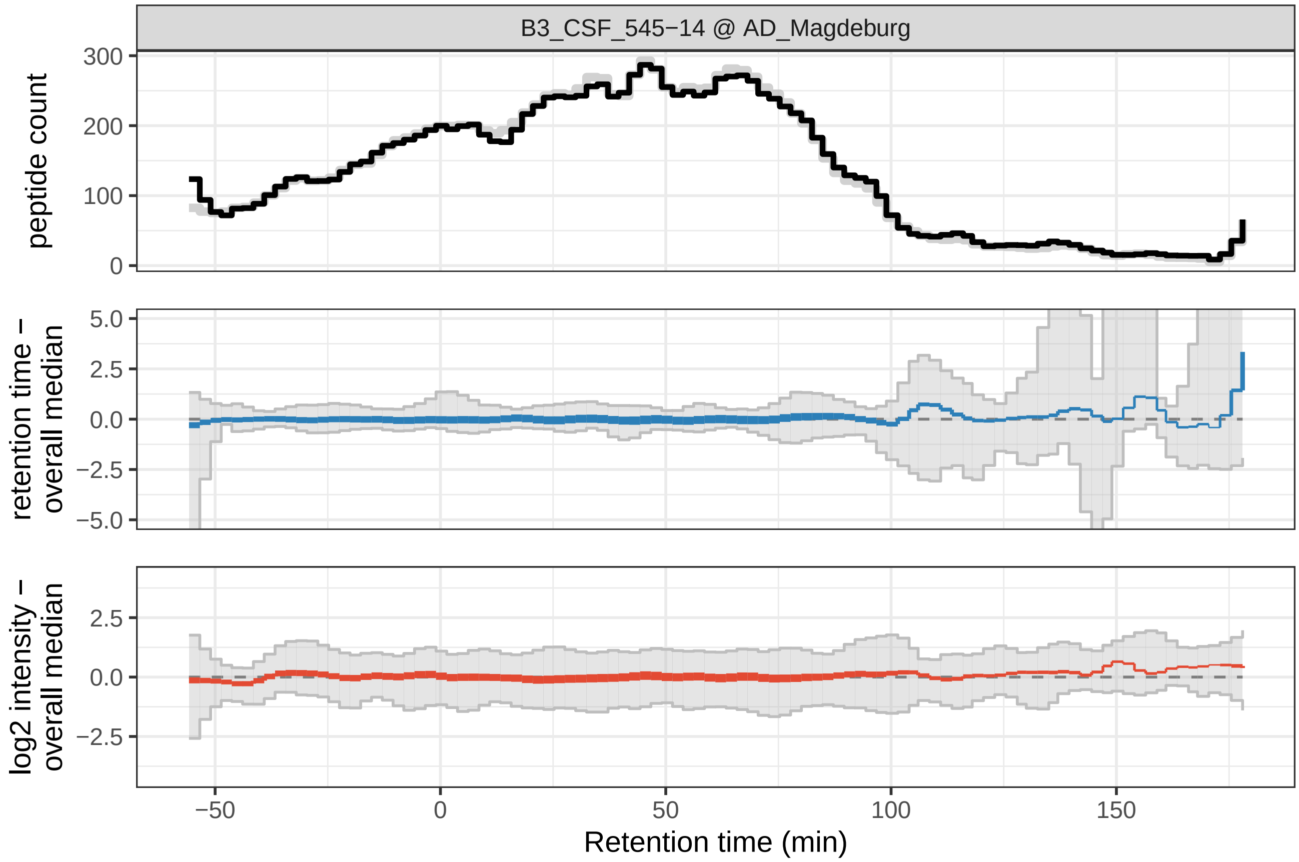
B3_CSF_545−14 @ AD_Magdeburg
peptide count
retention time –
overall median
log2 intensity –
overall median
Retention time (min)

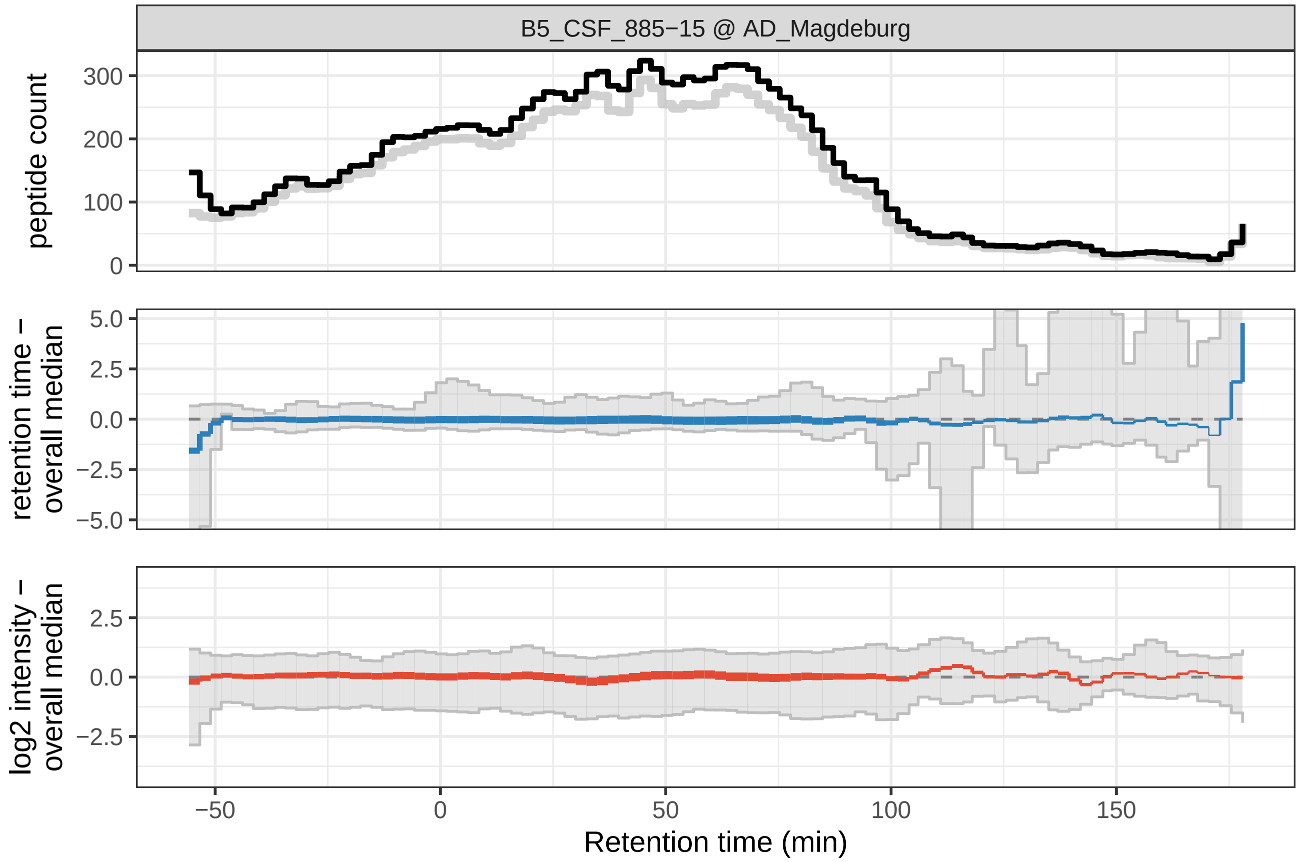
B5_CSF_885−15 @ AD_Magdeburg
peptide count
300
200
100
0
retention time –
overall median
5.0
2.5
0.0
−2.5
−5.0
log2 intensity –
overall median
2.5
0.0
−2.5
−50
0
50
100
150
Retention time (min)

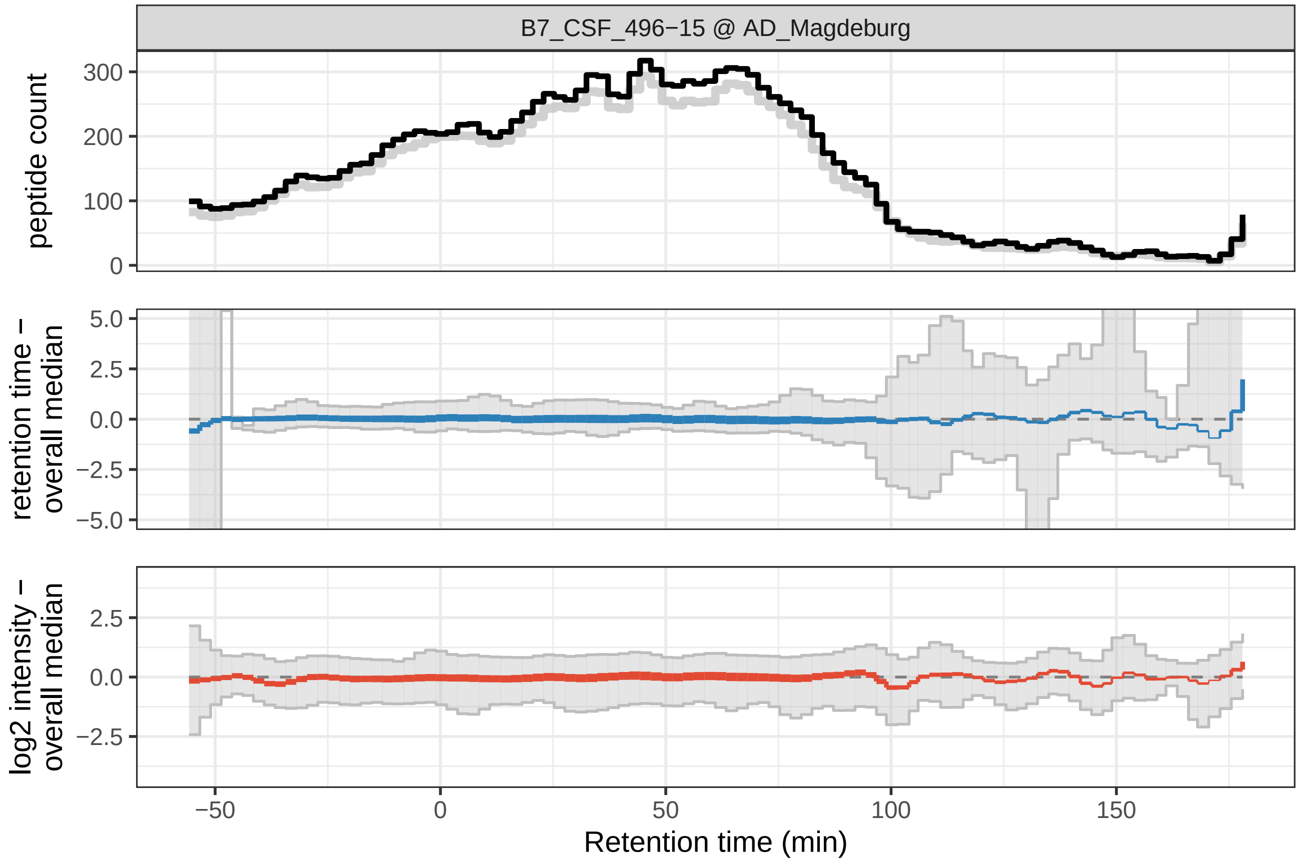
B7_CSF_496−15 @ AD_Magdeburg
peptide count
300
200
100
0
retention time –
overall median
5.0
2.5
0.0
−2.5
−5.0
log2 intensity –
overall median
2.5
0.0
−2.5
−50
0
50
100
150
Retention time (min)

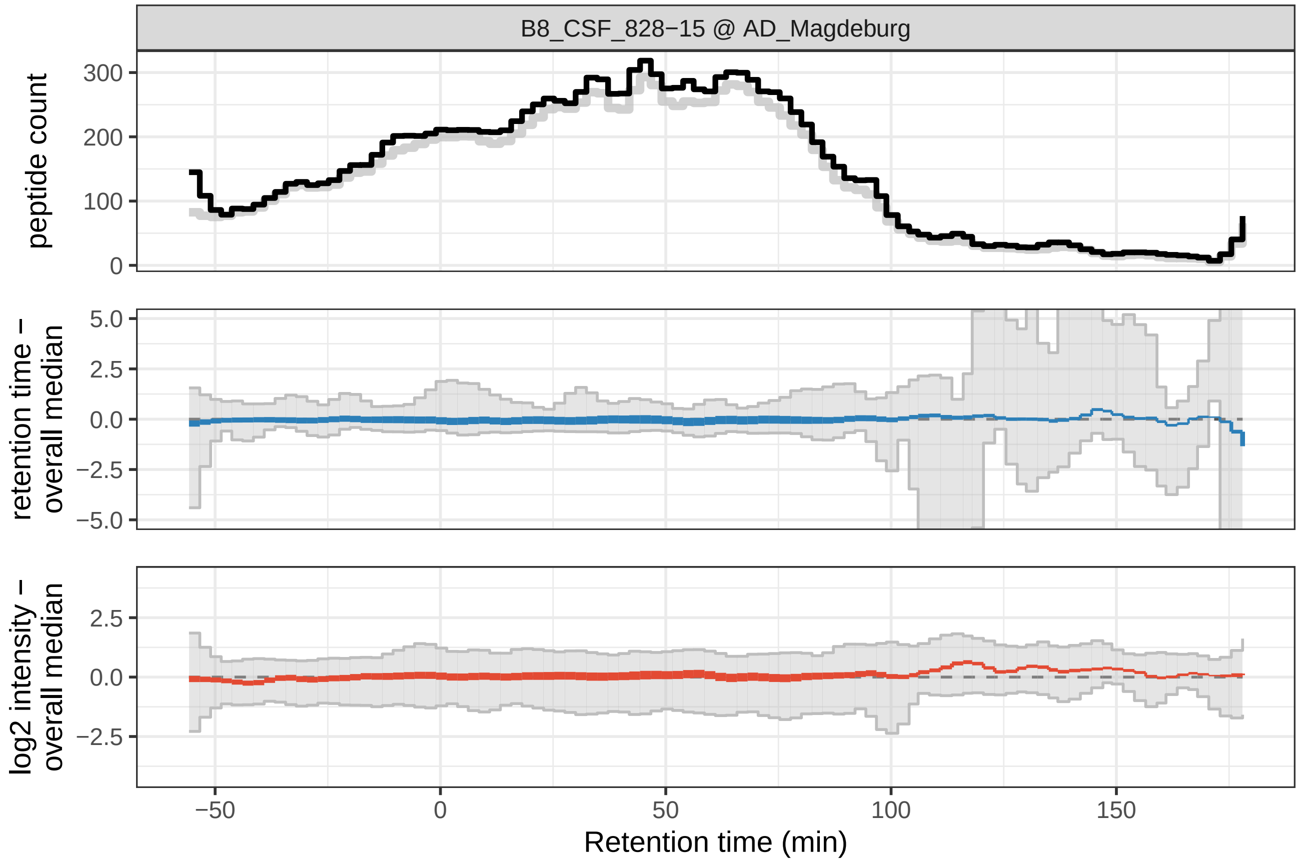
B8_CSF_828−15 @ AD_Magdeburg
peptide count
retention time − overall median
log2 intensity − overall median
Retention time (min)

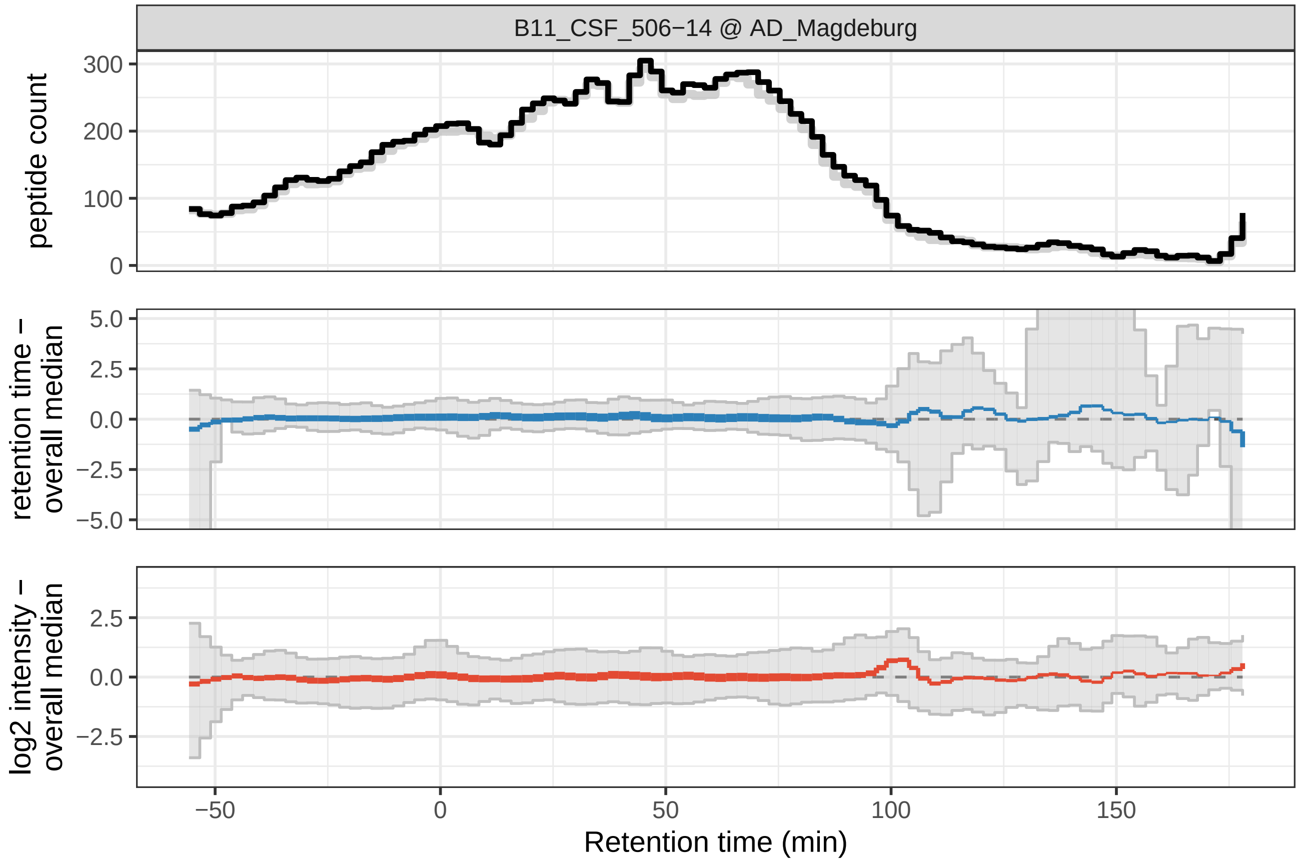
B11_CSF_506−14 @ AD_Magdeburg
peptide count
retention time − overall median
log2 intensity − overall median
Retention time (min)

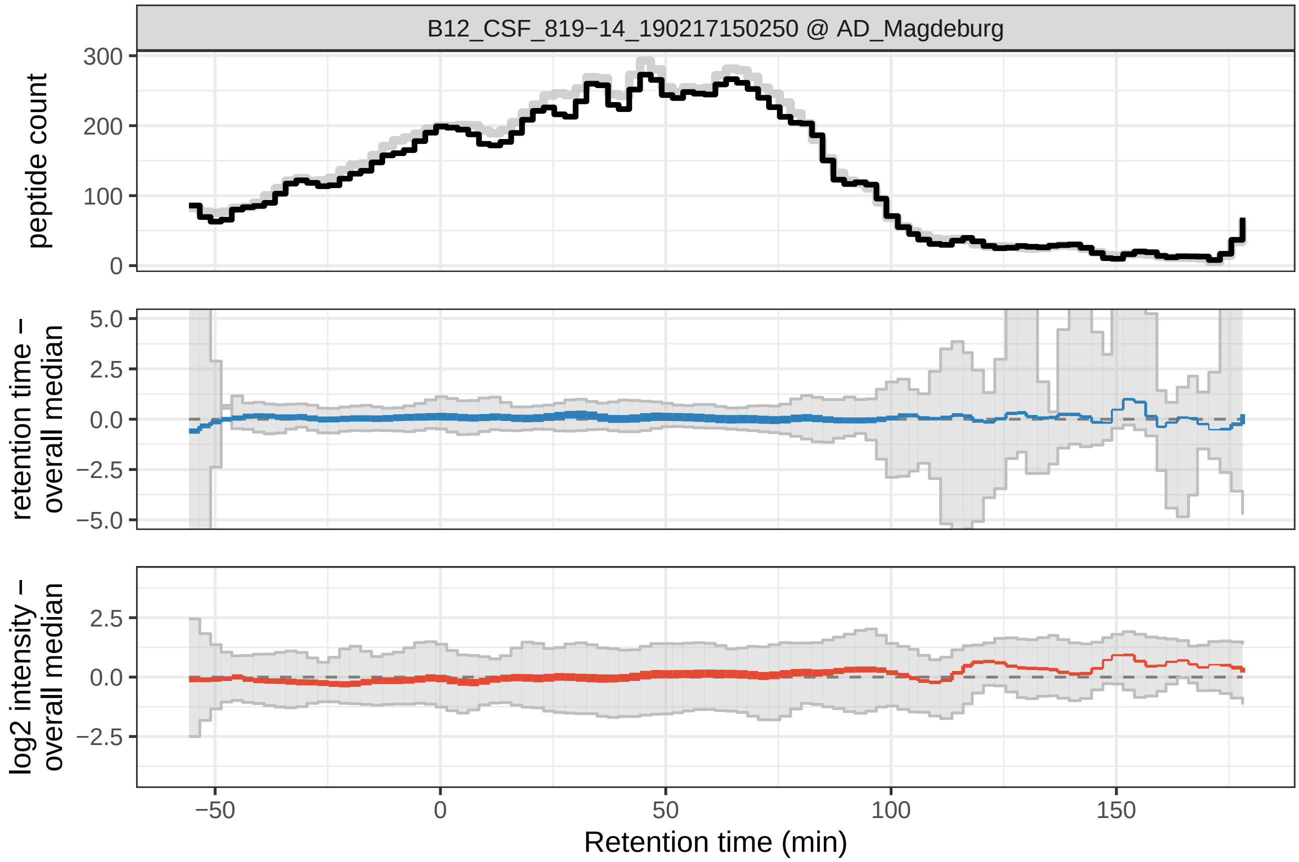

B12_CSF_819−14_190217150250 @ AD_Magdeburg
peptide count
retention time − overall median
log2 intensity − overall median
Retention time (min)

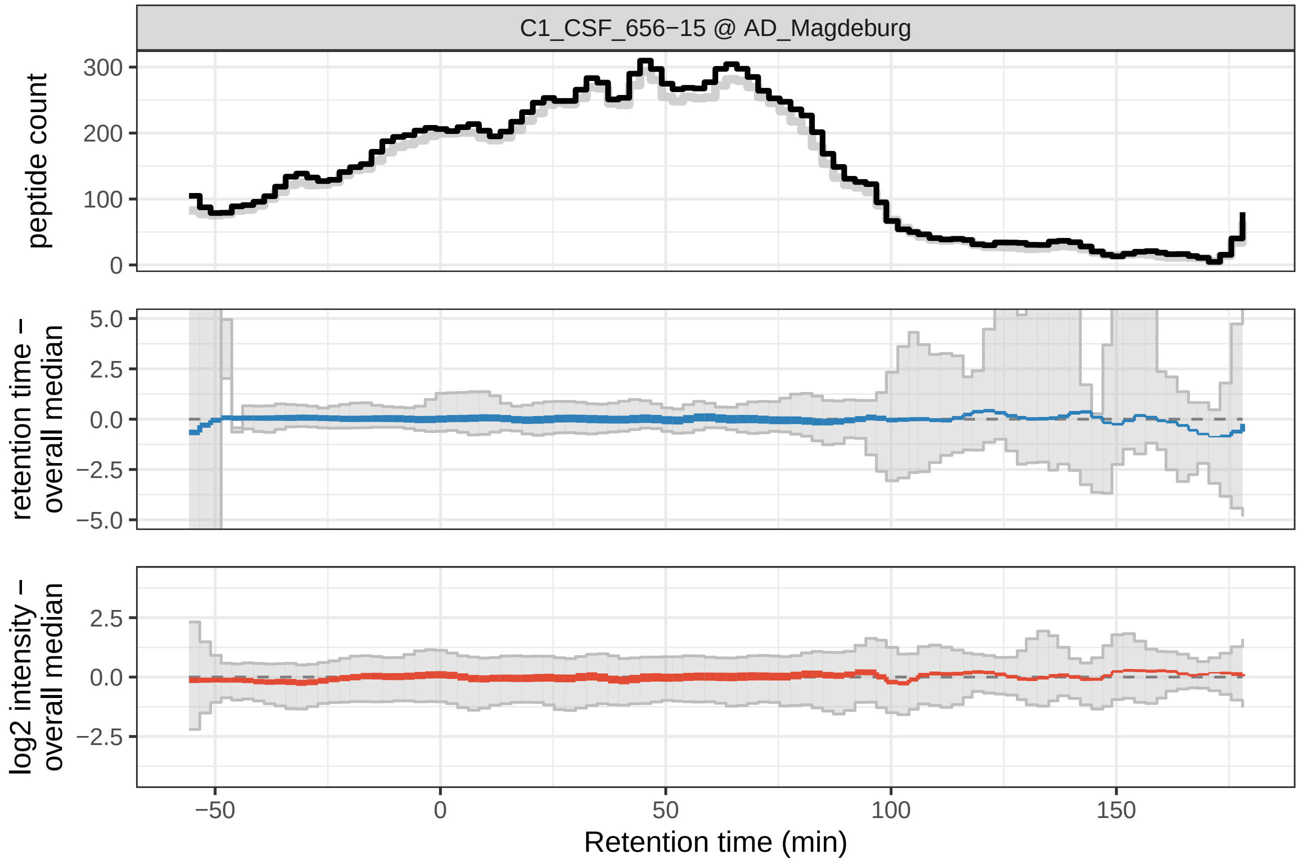

C1_CSF_656−15 @ AD_Magdeburg
peptide count
retention time − overall median
log2 intensity − overall median
Retention time (min)

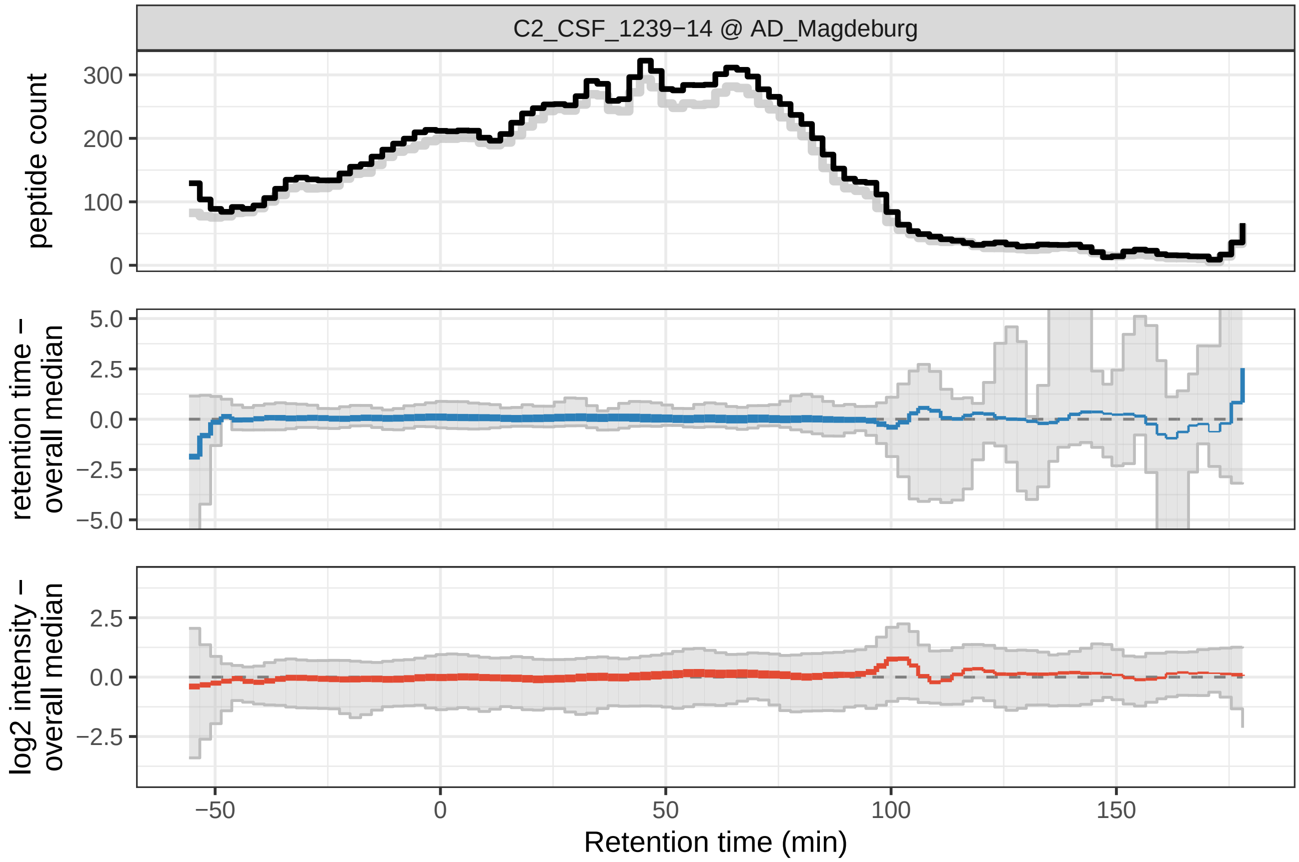
C2_CSF_1239−14 @ AD_Magdeburg
peptide count
300
200
100
0
retention time –
overall median
5.0
2.5
0.0
−2.5
−5.0
log2 intensity –
overall median
2.5
0.0
−2.5
−50
0
50
100
150
Retention time (min)

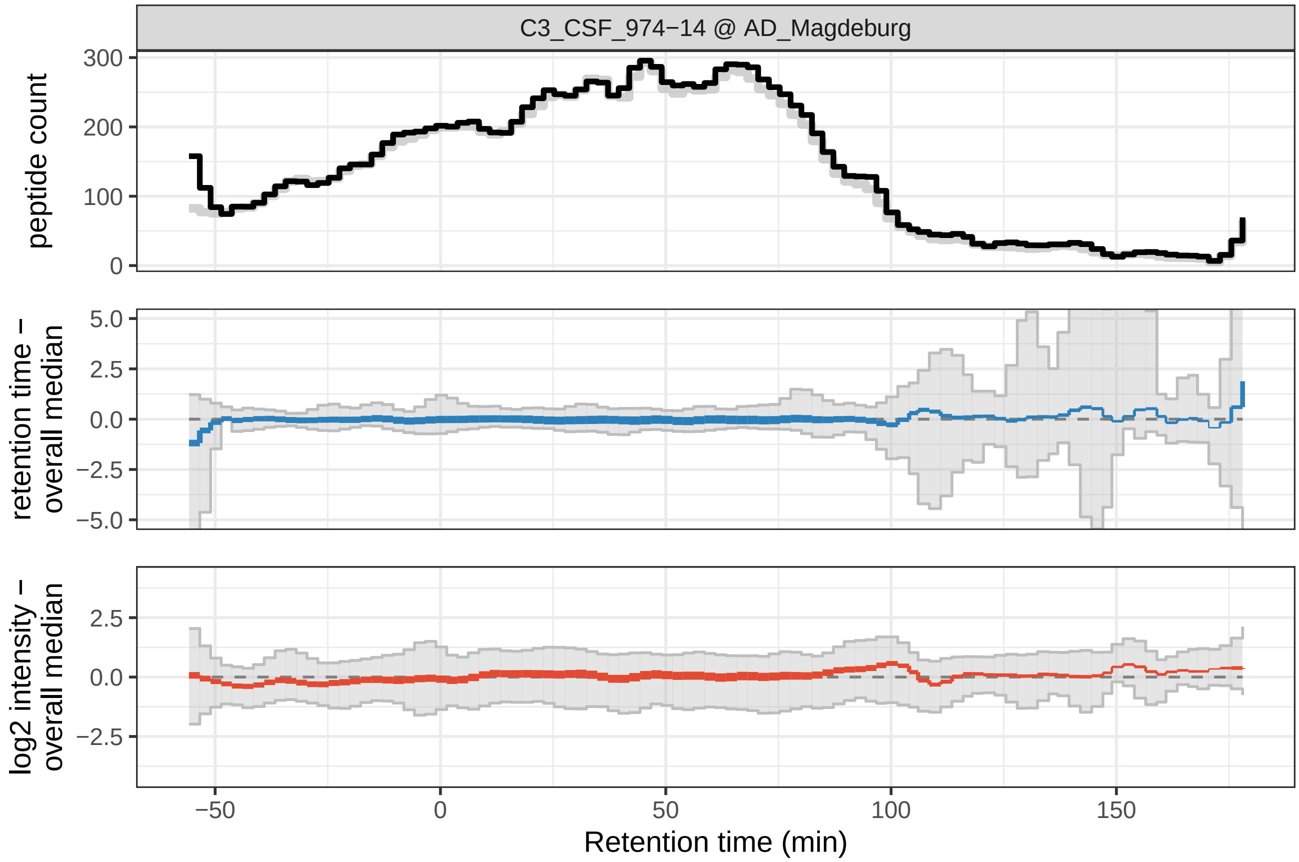
C3_CSF_974−14 @ AD_Magdeburg
peptide count
300
200
100
0
retention time –
overall median
5.0
2.5
0.0
−2.5
−5.0
log2 intensity –
overall median
2.5
0.0
−2.5
−50
0
50
100
150
Retention time (min)

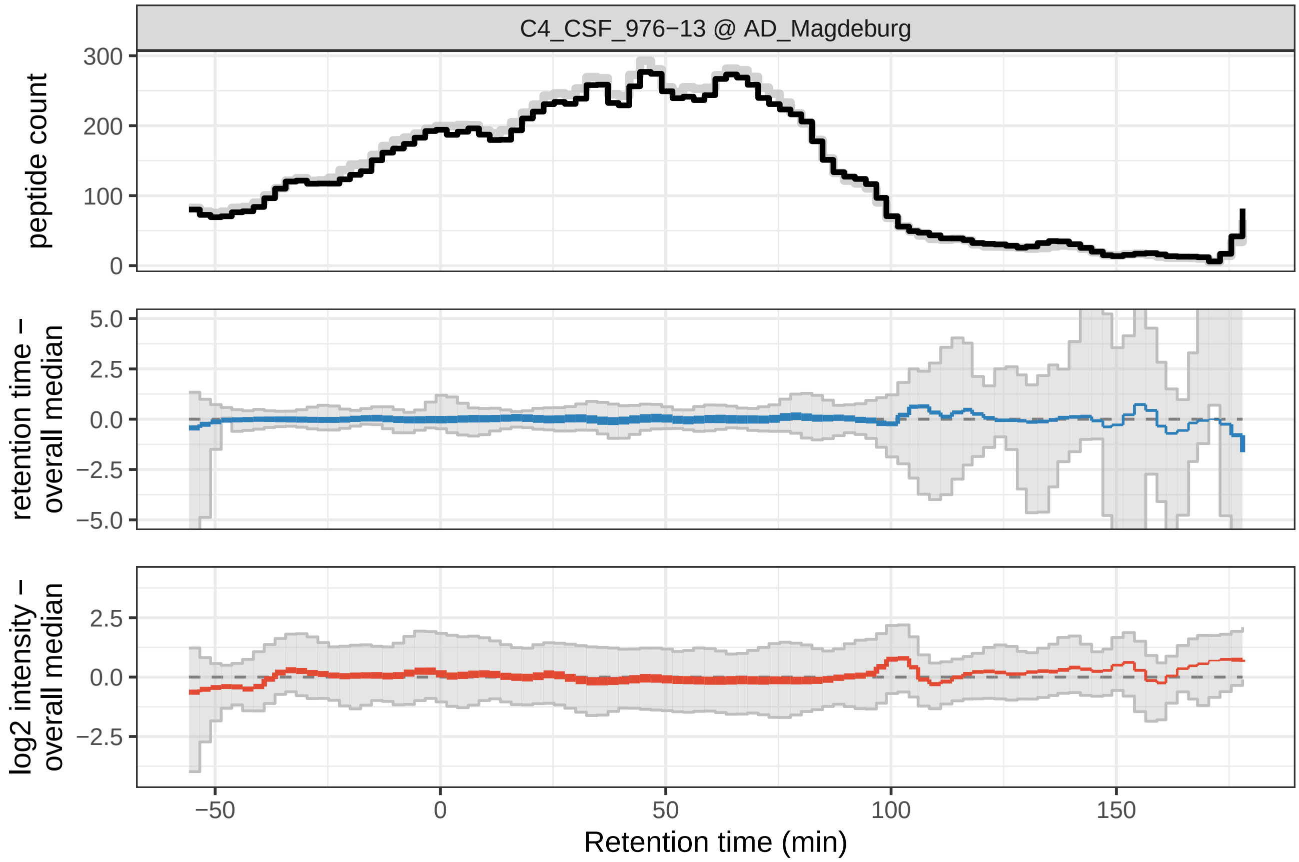
C4_CSF_976−13 @ AD_Magdeburg
peptide count
retention time – overall median
log2 intensity – overall median
Retention time (min)

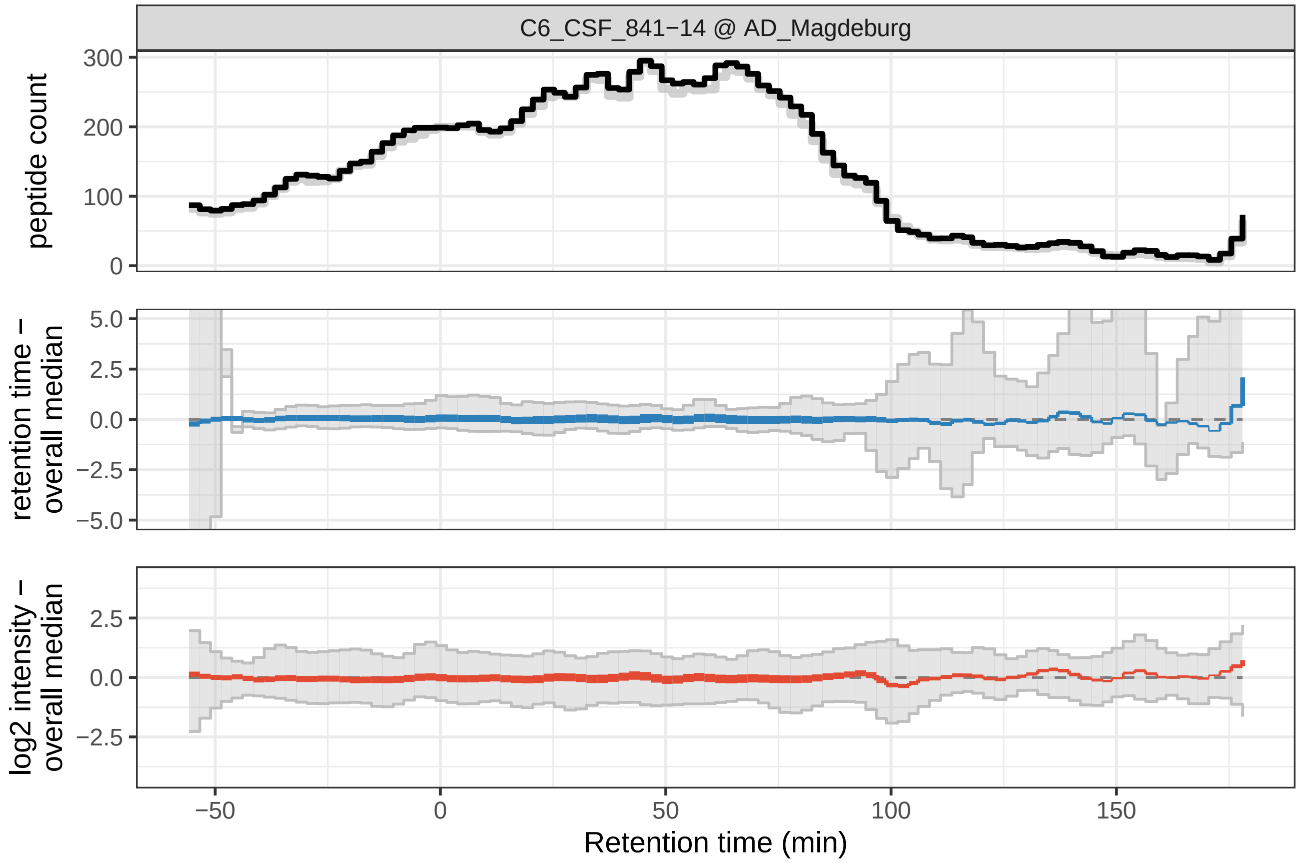
C6_CSF_841−14 @ AD_Magdeburg
peptide count
retention time – overall median
log2 intensity – overall median
Retention time (min)

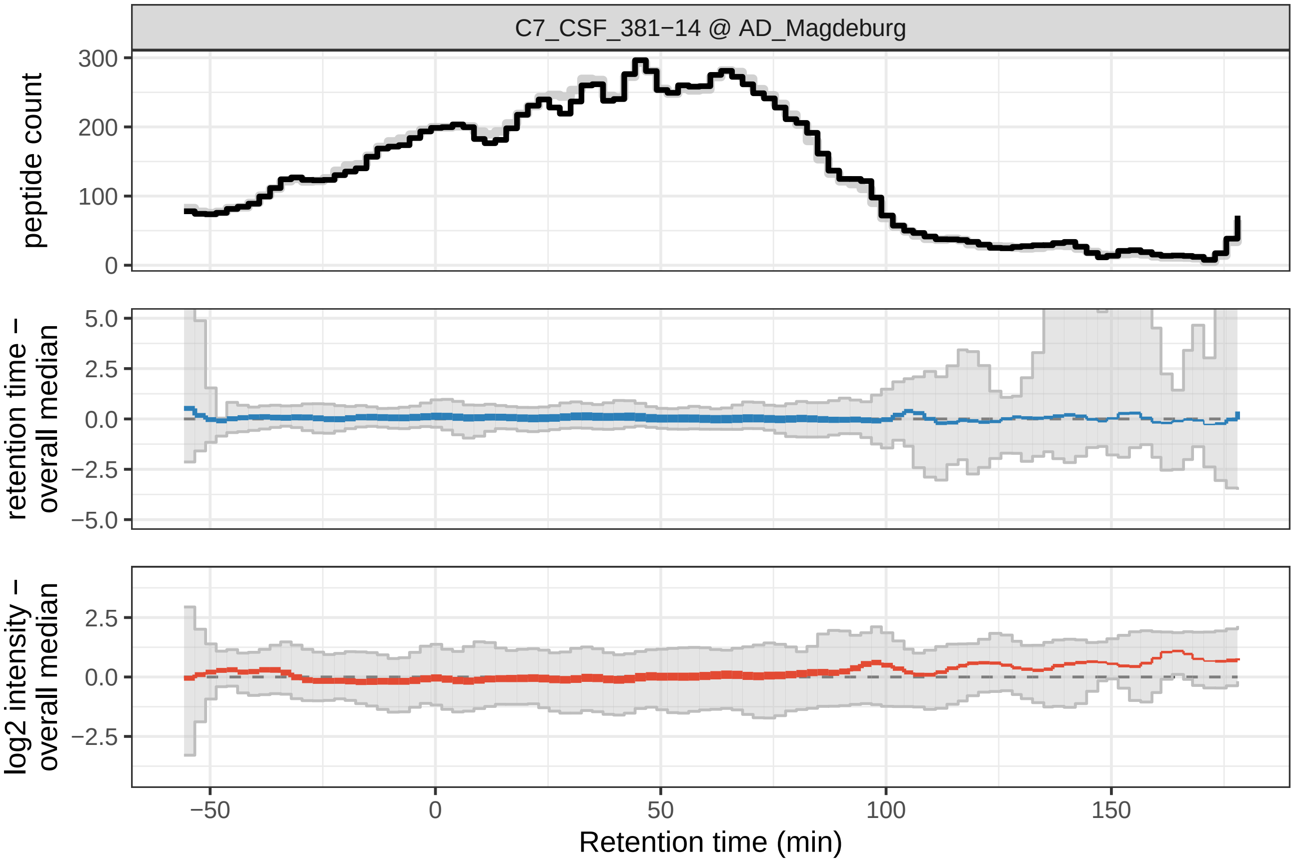
C7_CSF_381−14 @ AD_Magdeburg
peptide count
300
200
100
0
retention time –
overall median
5.0
2.5
0.0
−2.5
−5.0
log2 intensity –
overall median
2.5
0.0
−2.5
−50
0
50
100
150
Retention time (min)

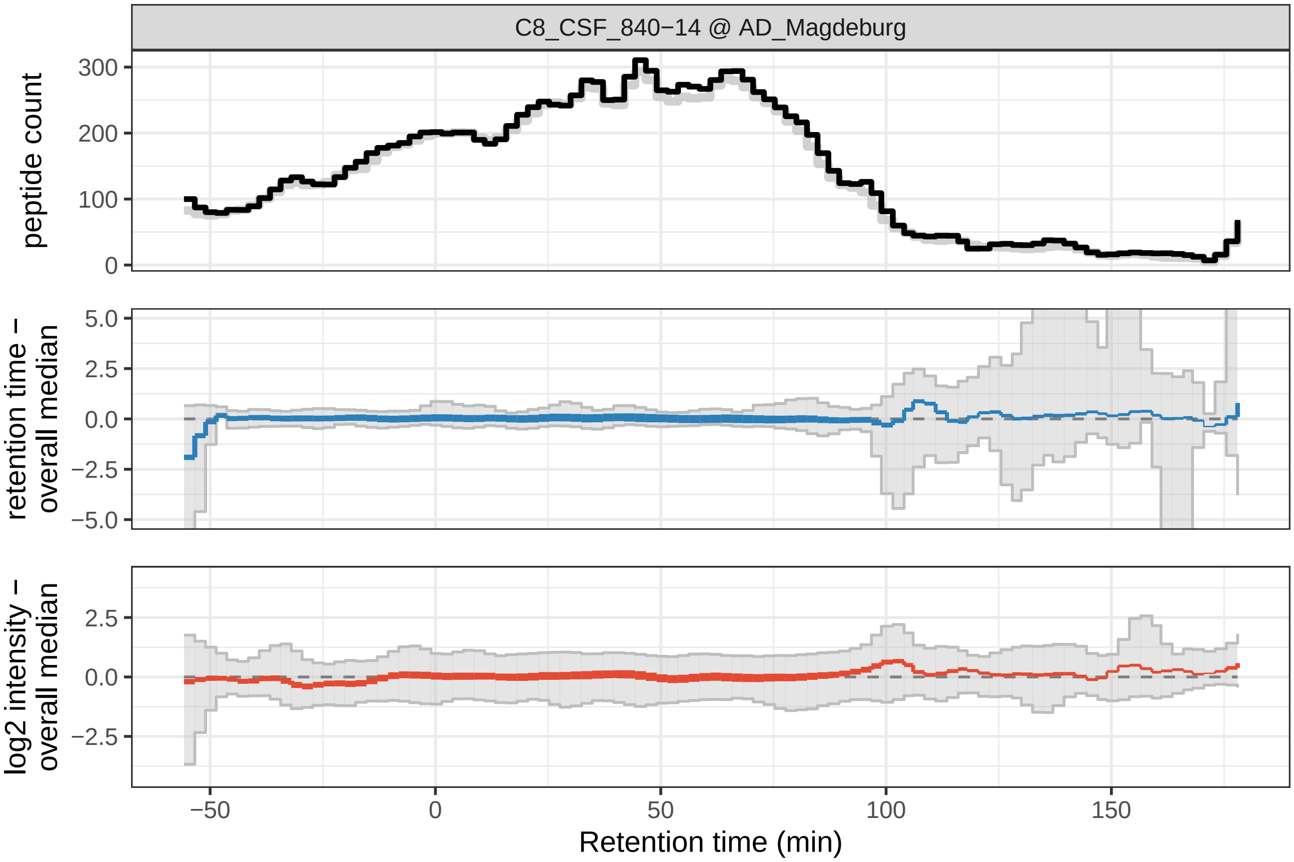
C8_CSF_840−14 @ AD_Magdeburg
peptide count
300
200
100
0
retention time –
overall median
5.0
2.5
0.0
−2.5
−5.0
log2 intensity –
overall median
2.5
0.0
−2.5
−50
0
50
100
150
Retention time (min)

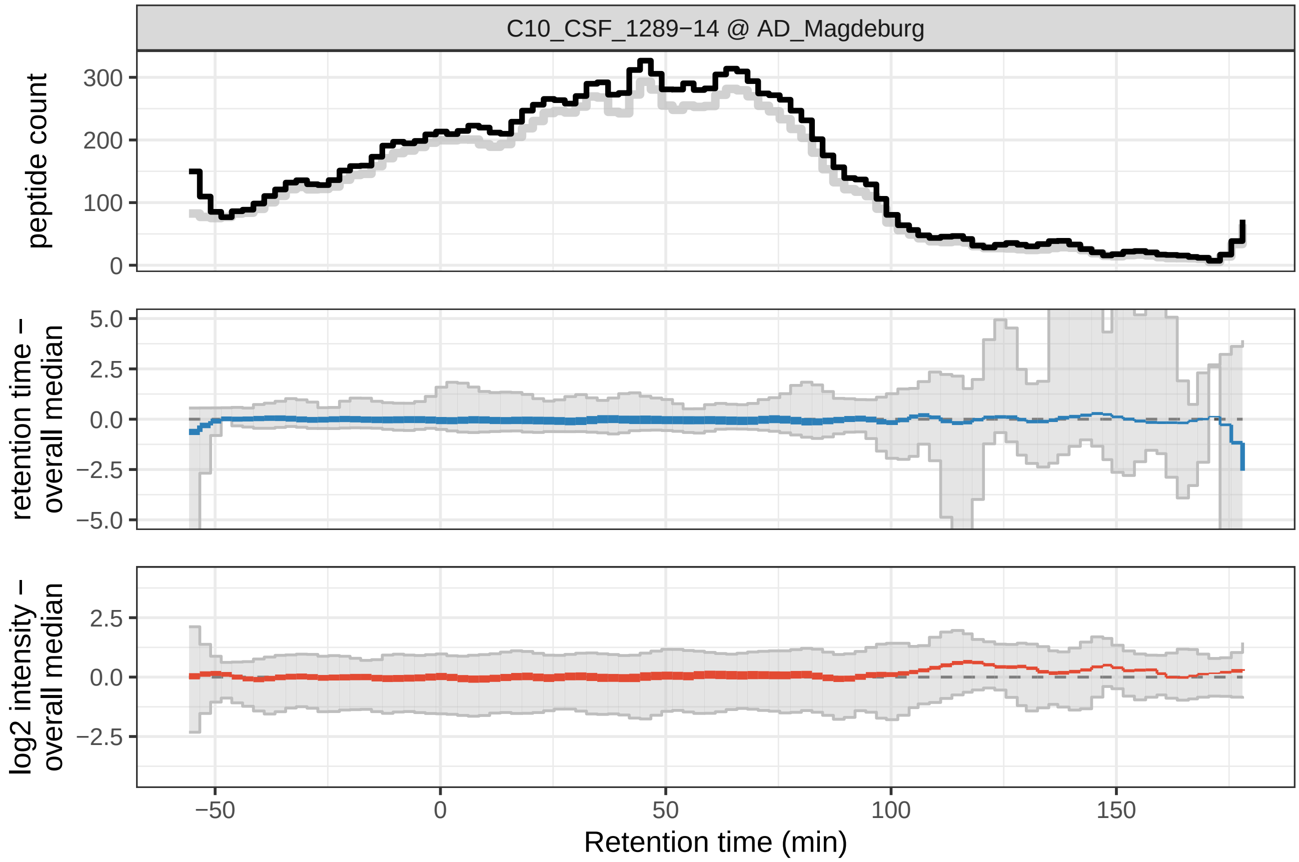
C10_CSF_1289−14 @ AD_Magdeburg
peptide count
retention time – overall median
log2 intensity – overall median
Retention time (min)

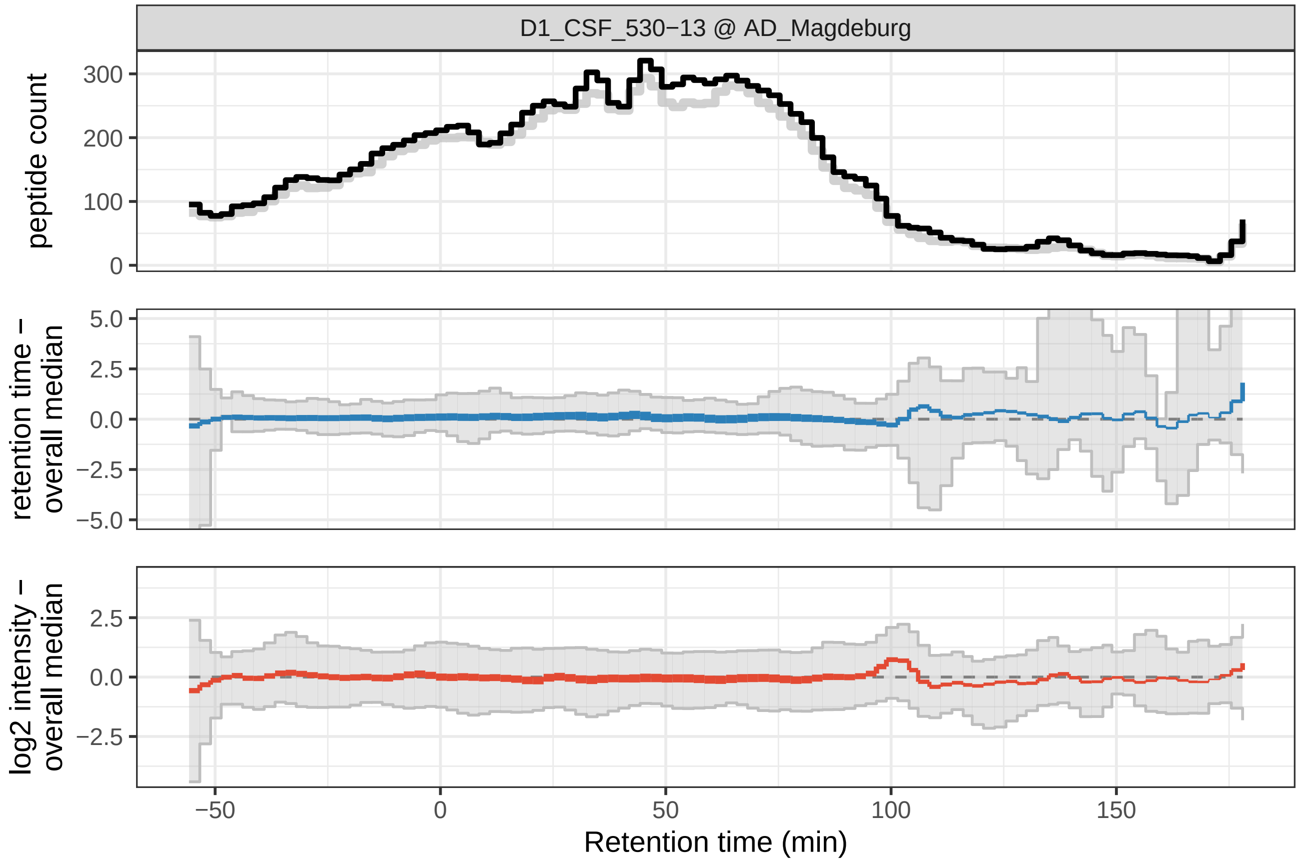
D1_CSF_530−13 @ AD_Magdeburg
peptide count
retention time – overall median
log2 intensity – overall median
Retention time (min)

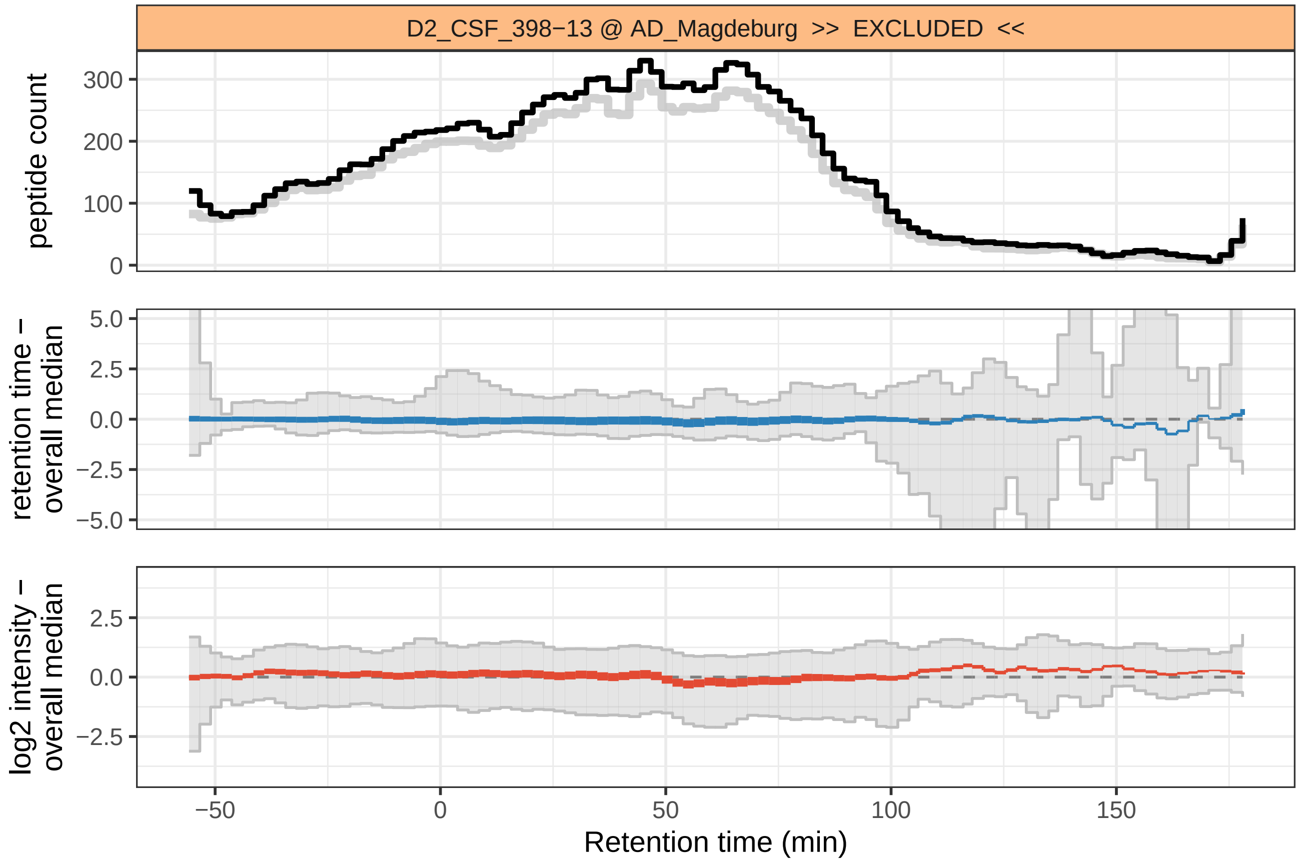
D2_CSF_398−13 @ AD_Magdeburg >> EXCLUDED <<
peptide count
retention time – overall median
log2 intensity – overall median
Retention time (min)

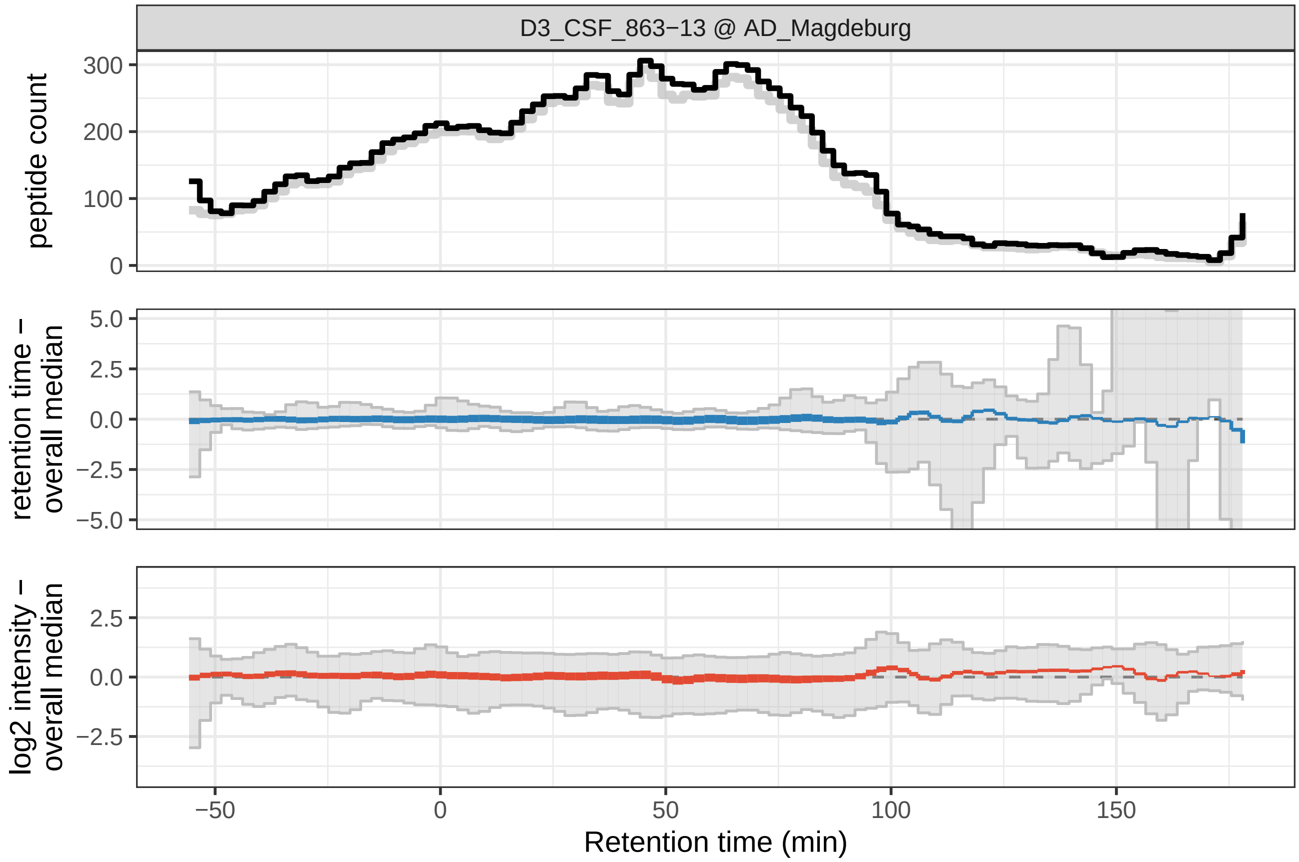
D3_CSF_863−13 @ AD_Magdeburg
peptide count
retention time – overall median
log2 intensity – overall median
Retention time (min)

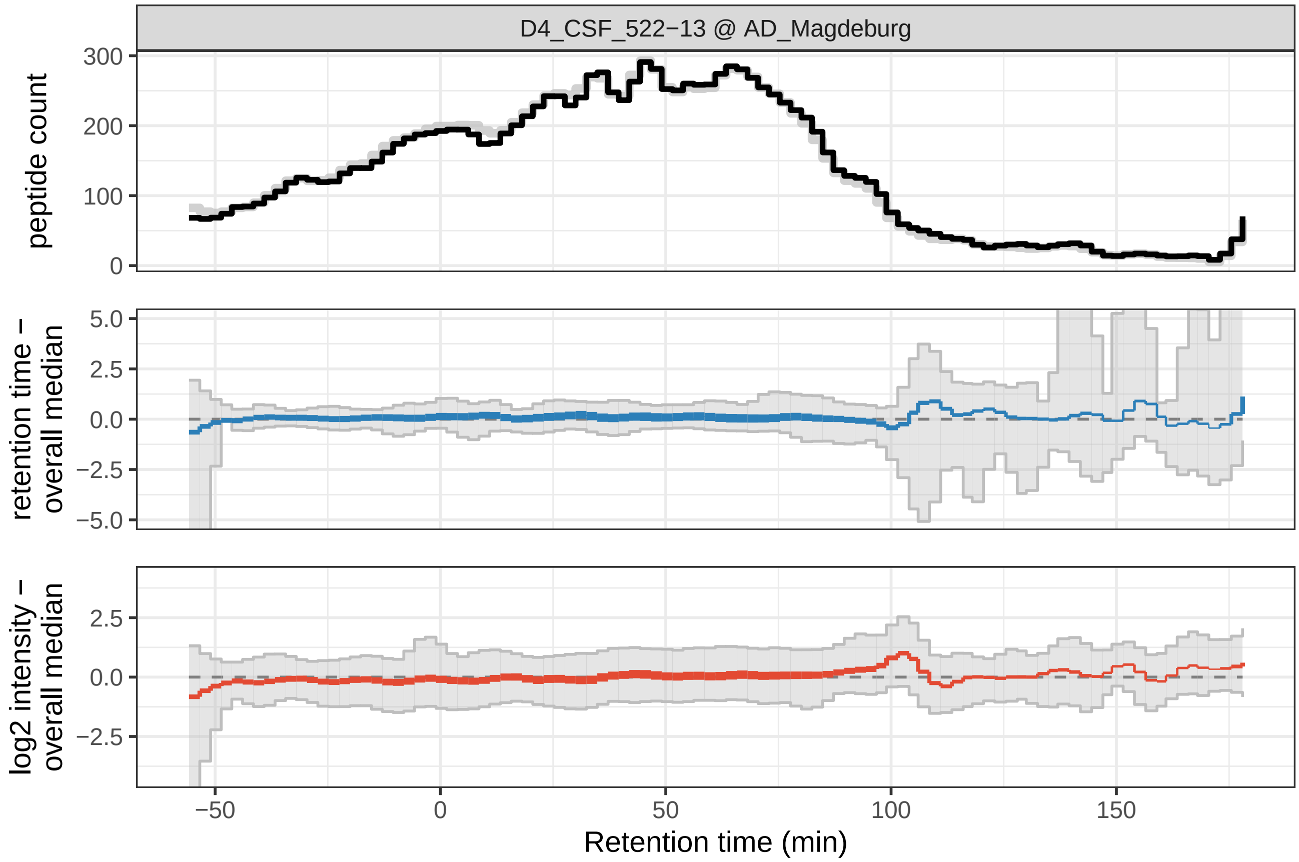
D4_CSF_522−13 @ AD_Magdeburg
peptide count
retention time – overall median
log2 intensity – overall median
Retention time (min)

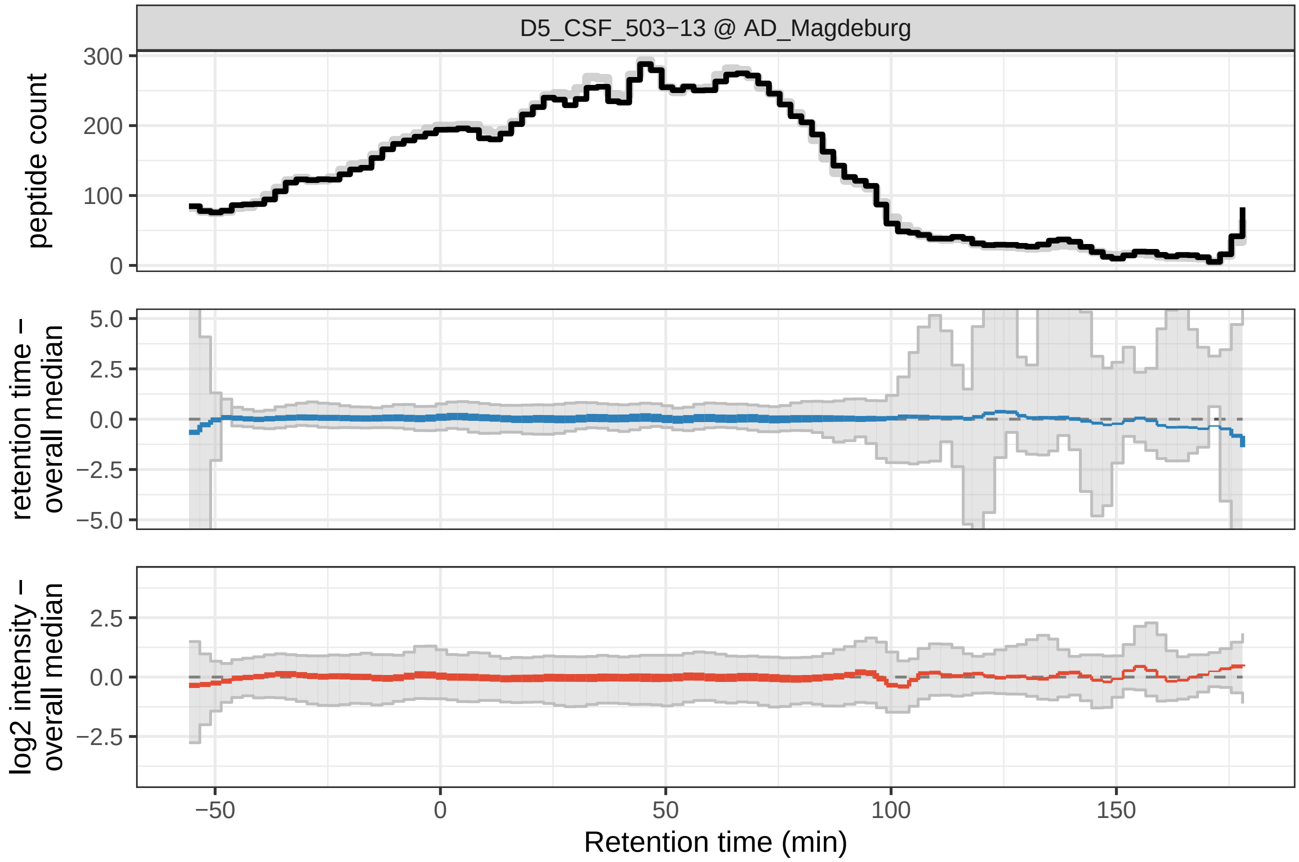
D5_CSF_503−13 @ AD_Magdeburg
peptide count
retention time – overall median
log2 intensity – overall median
Retention time (min)

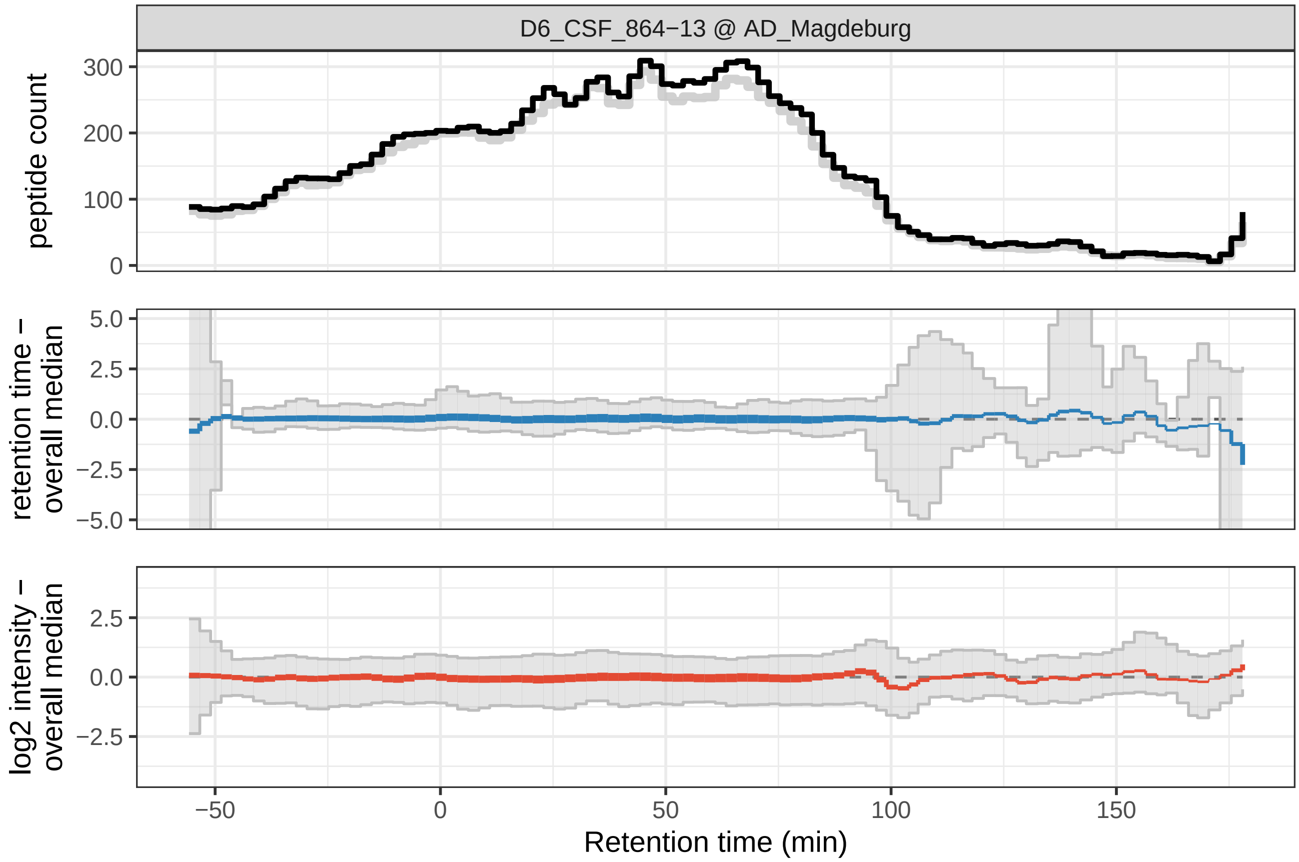
D6_CSF_864−13 @ AD_Magdeburg
peptide count
retention time – overall median
log2 intensity – overall median
Retention time (min)

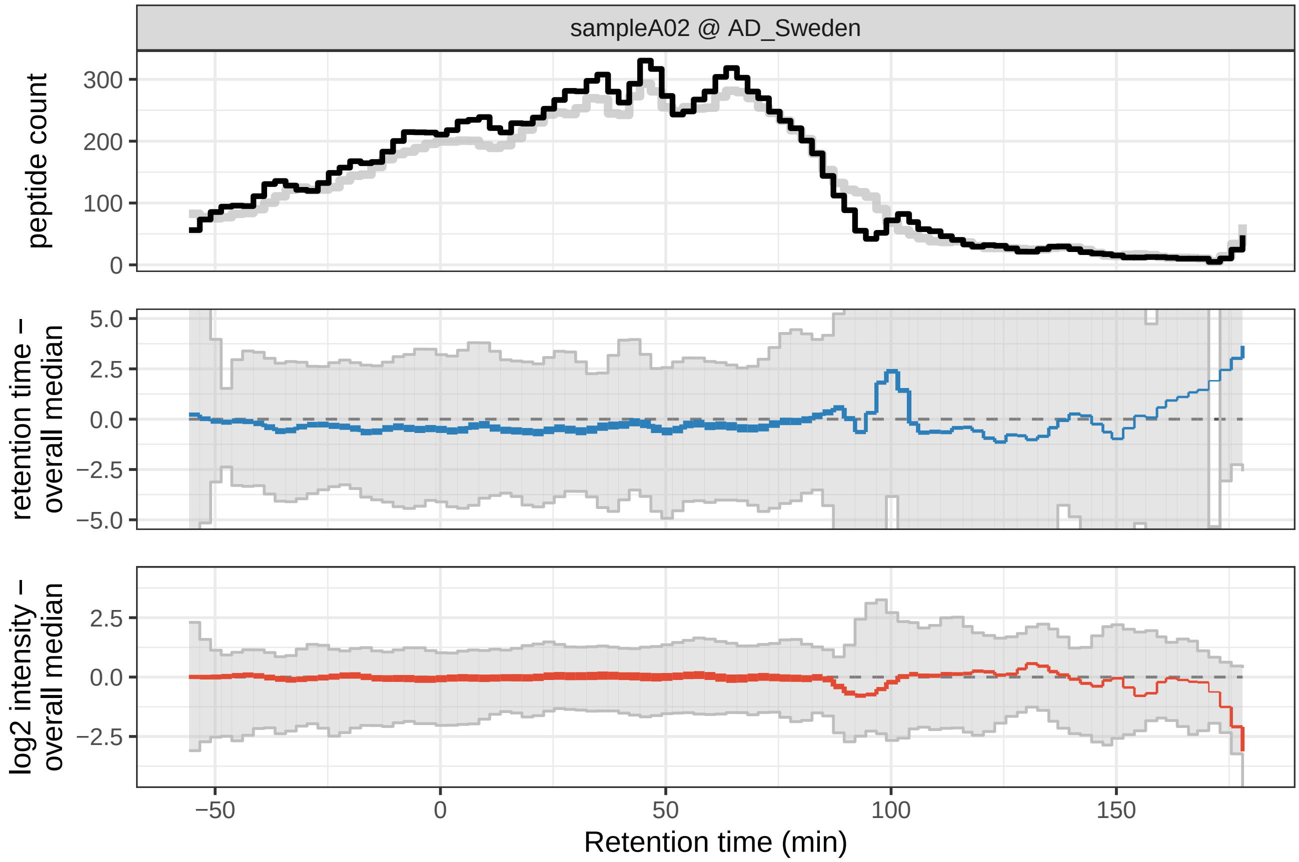
sampleA02 @ AD_Sweden
peptide count
retention time – overall median
log2 intensity – overall median
Retention time (min)

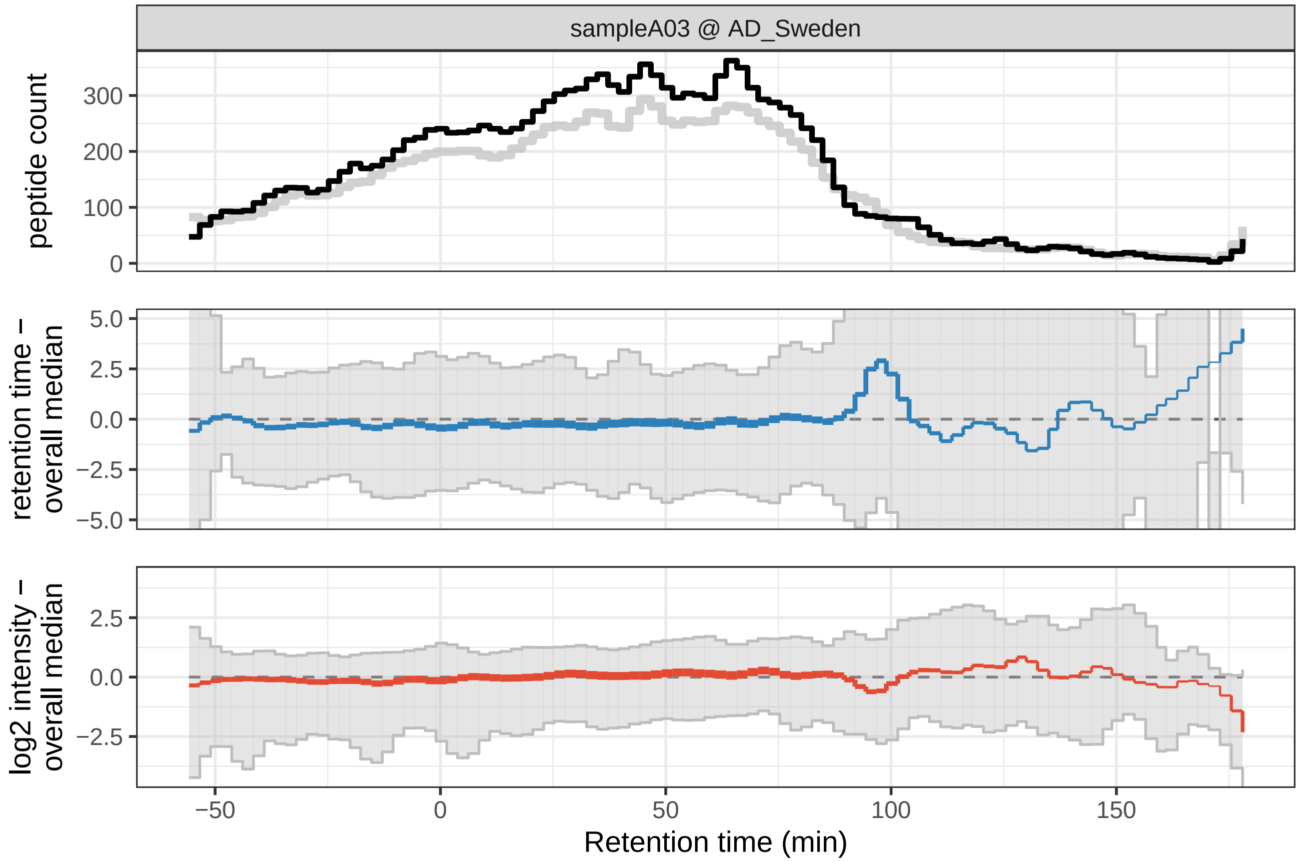
sampleA03 @ AD_Sweden
peptide count
retention time – overall median
log2 intensity – overall median
Retention time (min)

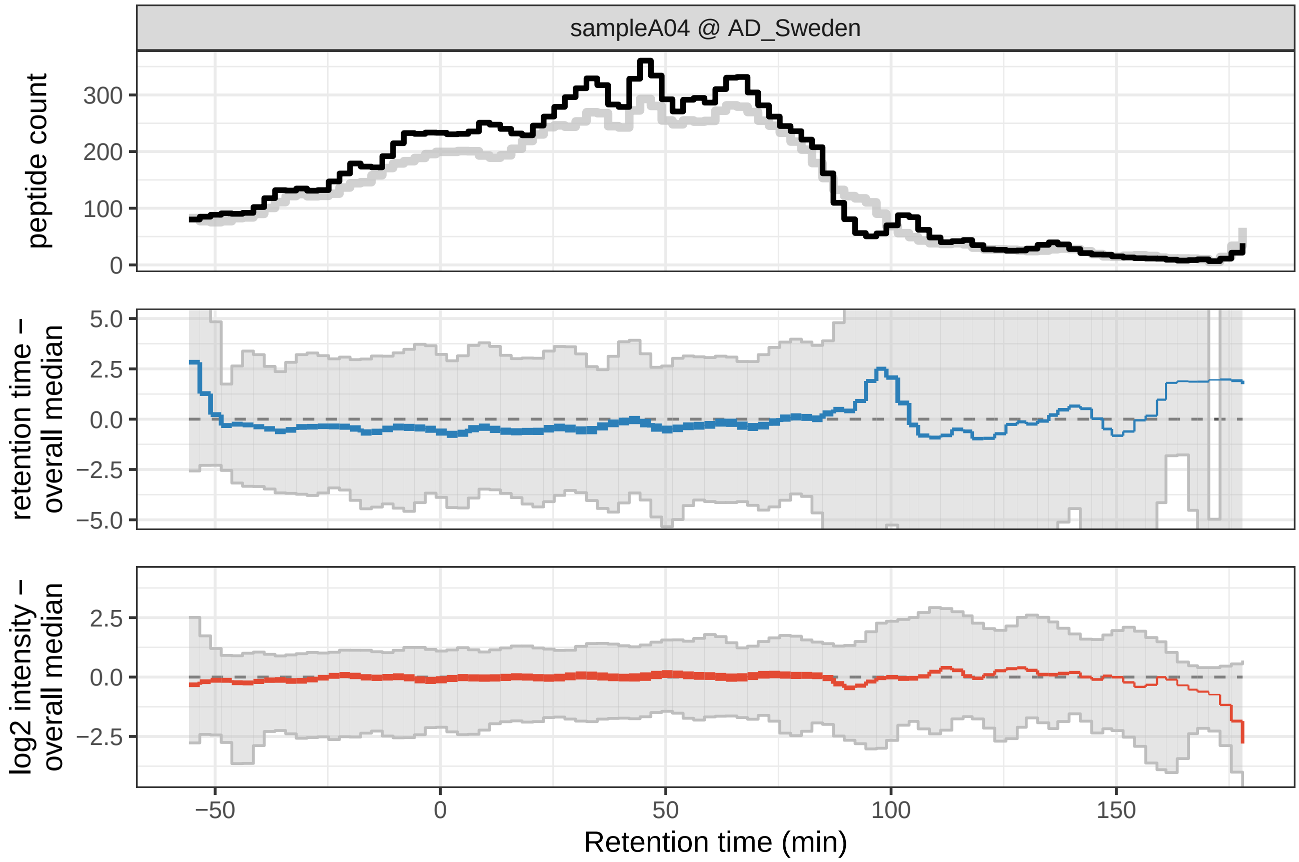
sampleA04 @ AD_Sweden
peptide count
retention time – overall median
log2 intensity – overall median
Retention time (min)

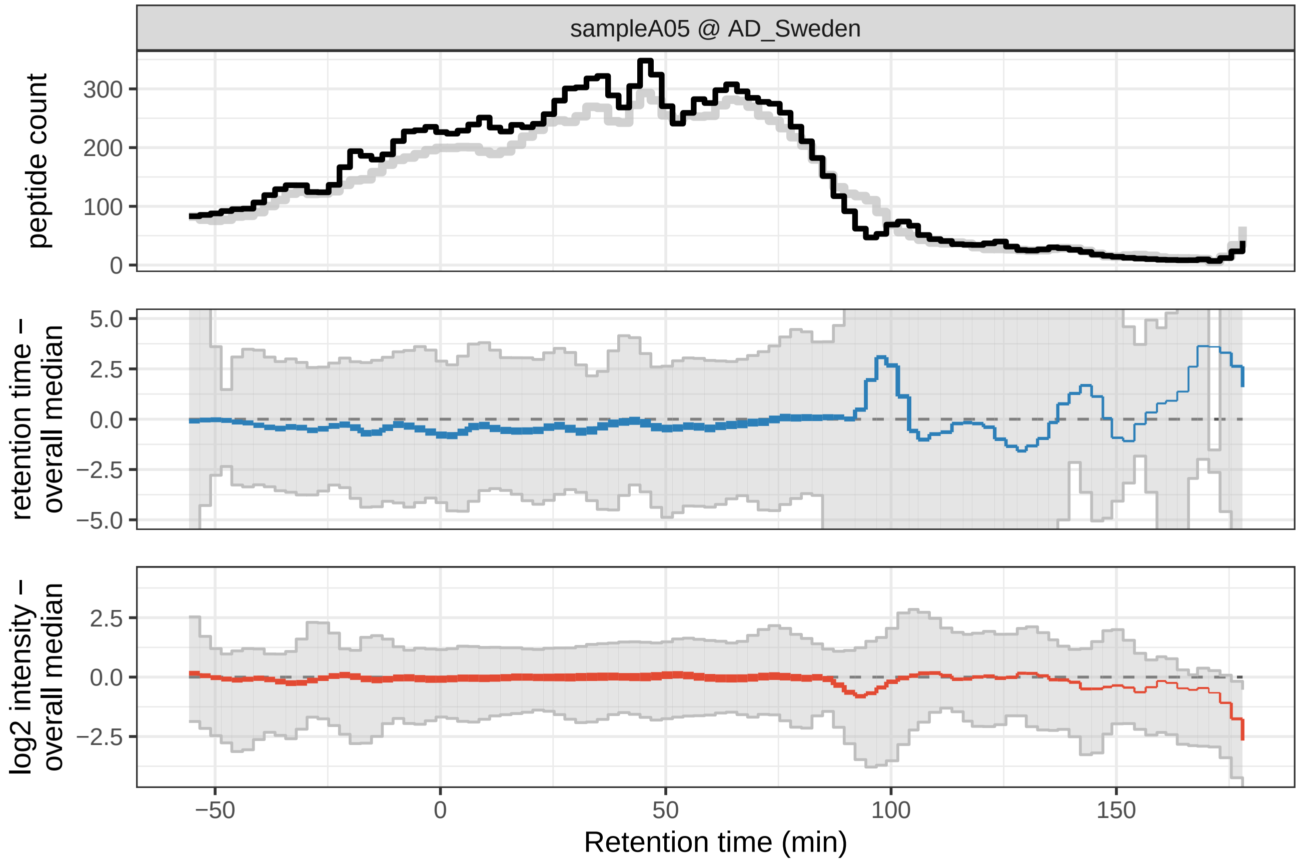
sampleA05 @ AD_Sweden
peptide count
300
200
100
0
retention time – overall median
5.0
2.5
0.0
−2.5
−5.0
log2 intensity – overall median
2.5
0.0
−2.5
−50
0
50
100
150
Retention time (min)

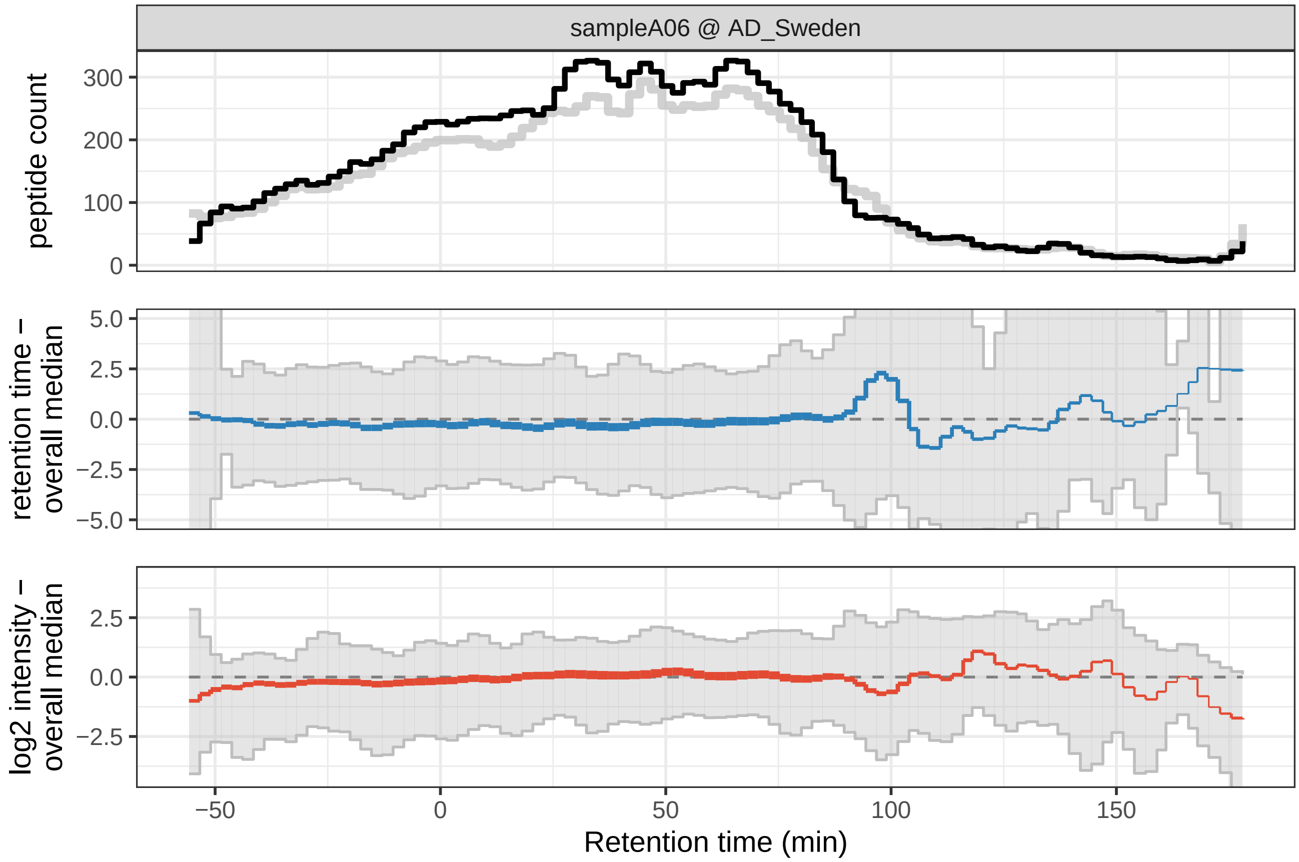
sampleA06 @ AD_Sweden
peptide count
300
200
100
0
retention time – overall median
5.0
2.5
0.0
−2.5
−5.0
log2 intensity – overall median
2.5
0.0
−2.5
−50
0
50
100
150
Retention time (min)

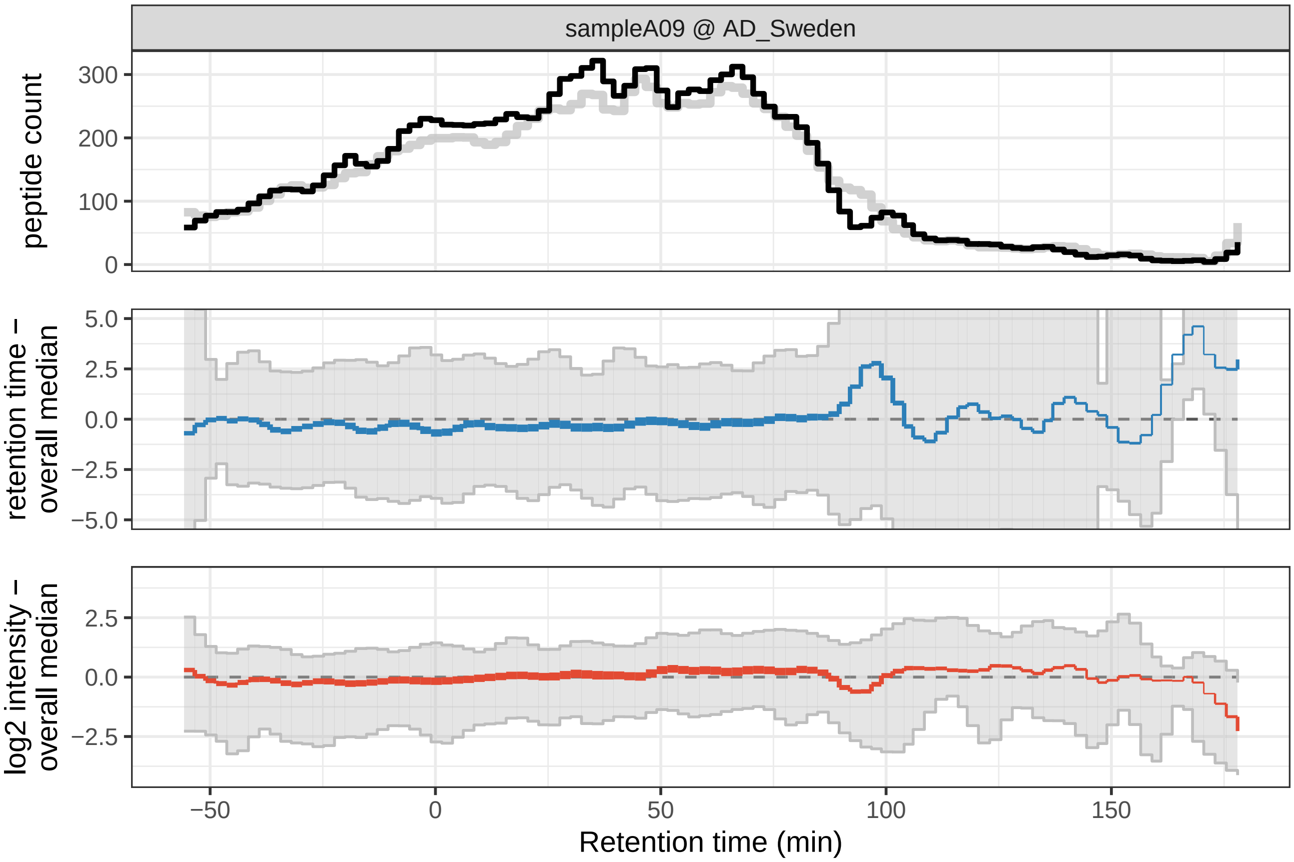

sampleA09 @ AD_Sweden
peptide count
300
200
100
0
retention time –
overall median
5.0
2.5
0.0
−2.5
−5.0
log2 intensity –
overall median
2.5
0.0
−2.5
−50
0
50
100
150
Retention time (min)

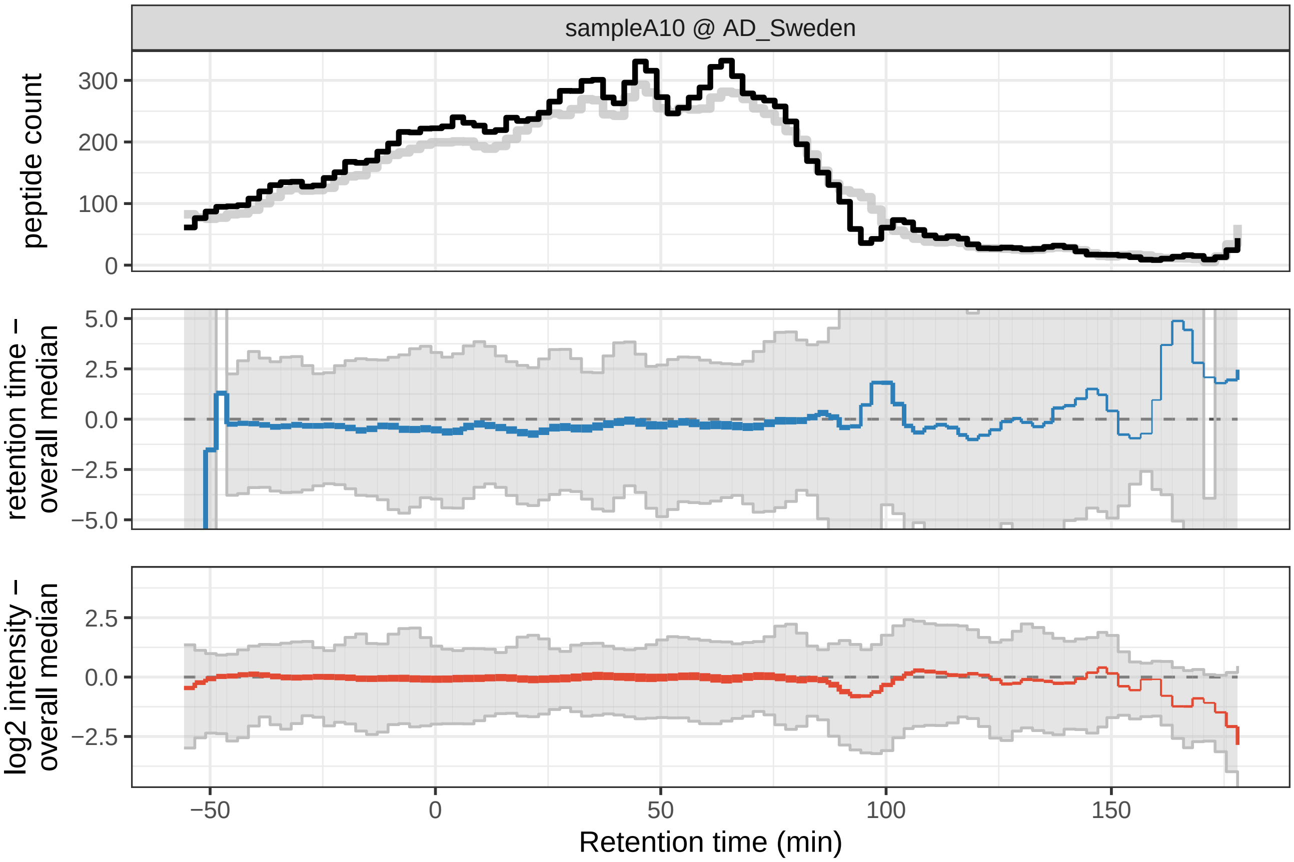

sampleA10 @ AD_Sweden
peptide count
300
200
100
0
retention time –
overall median
5.0
2.5
0.0
−2.5
−5.0
log2 intensity –
overall median
2.5
0.0
−2.5
−50
0
50
100
150
Retention time (min)

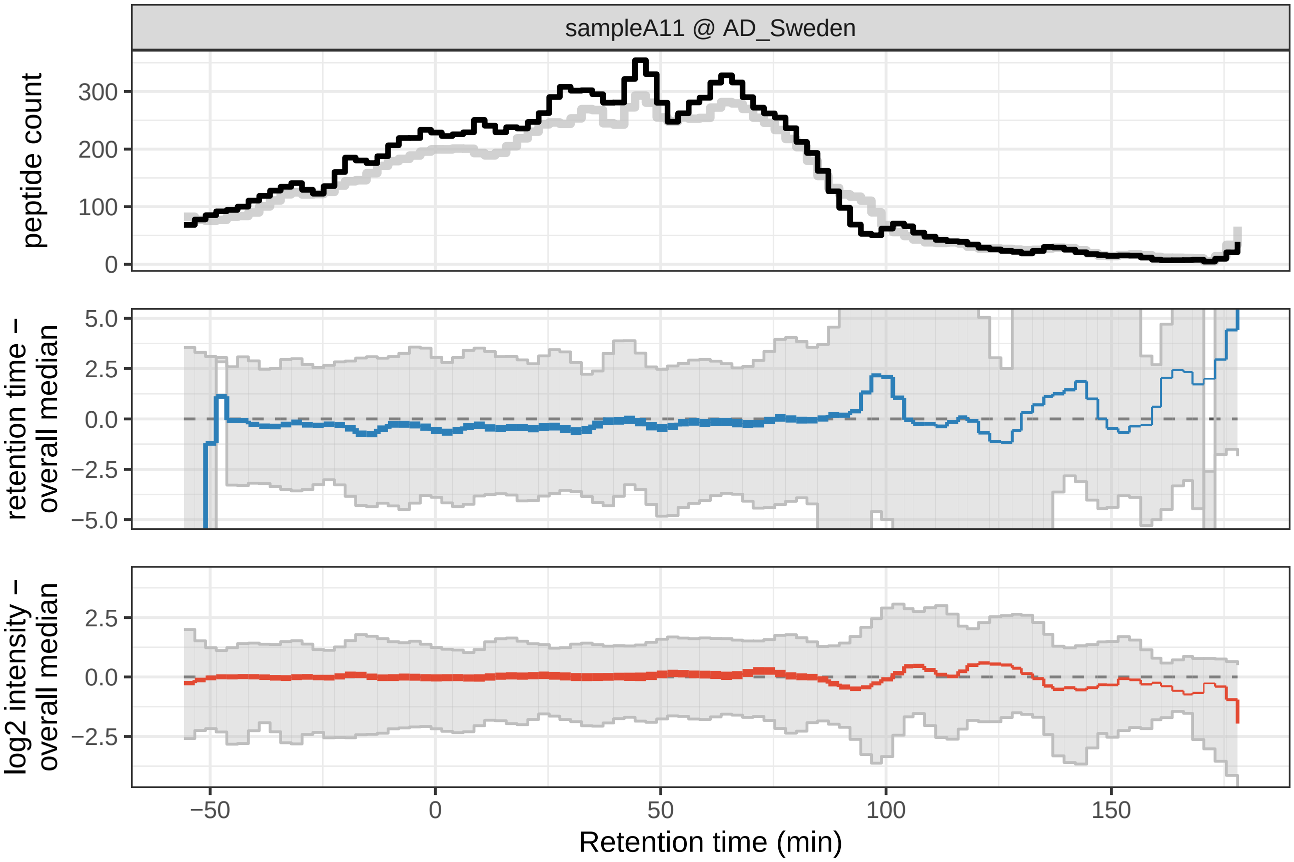
sampleA11 @ AD_Sweden
peptide count
300
200
100
0
retention time – overall median
5.0
2.5
0.0
−2.5
−5.0
log2 intensity – overall median
2.5
0.0
−2.5
−50
0
50
100
150
Retention time (min)

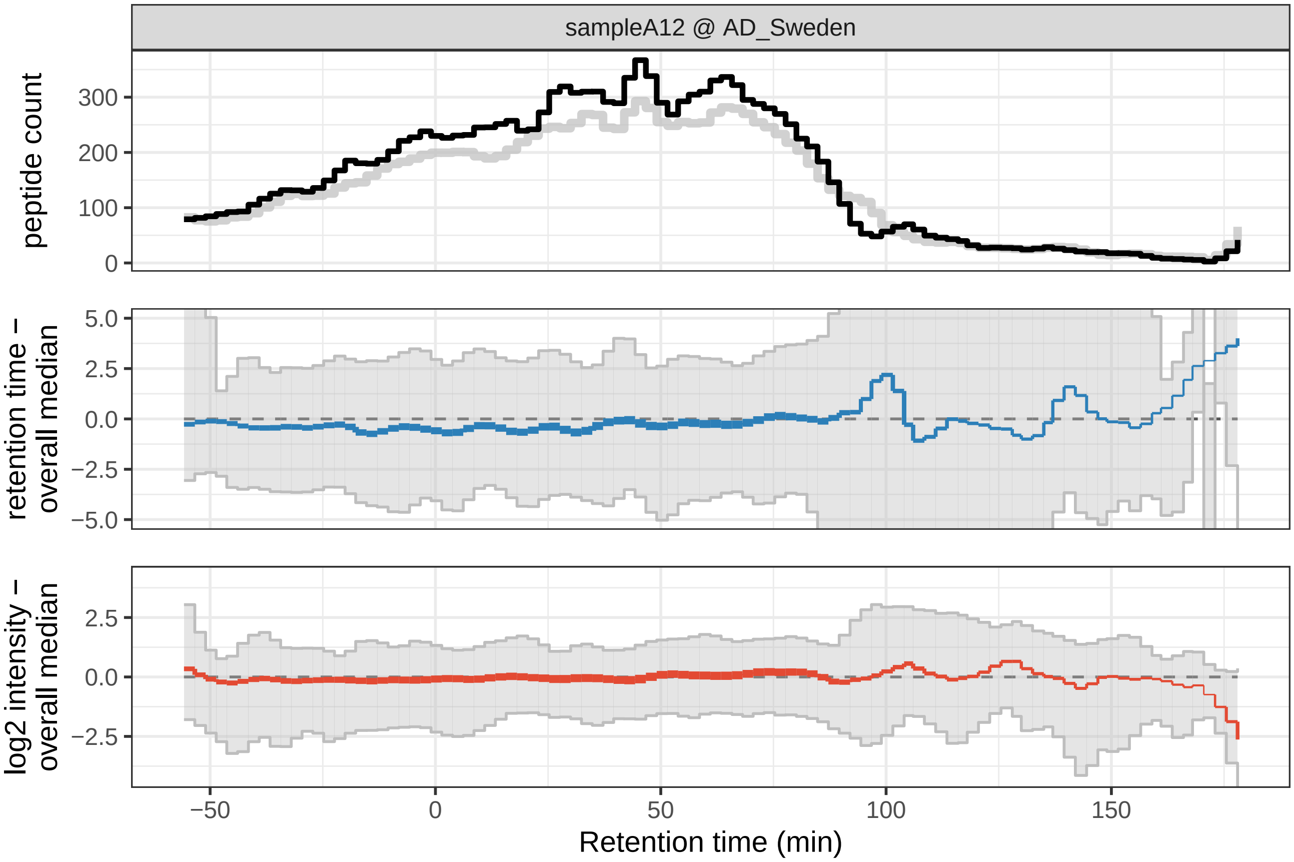
sampleA12 @ AD_Sweden
peptide count
300
200
100
0
retention time – overall median
5.0
2.5
0.0
−2.5
−5.0
log2 intensity – overall median
2.5
0.0
−2.5
−50
0
50
100
150
Retention time (min)

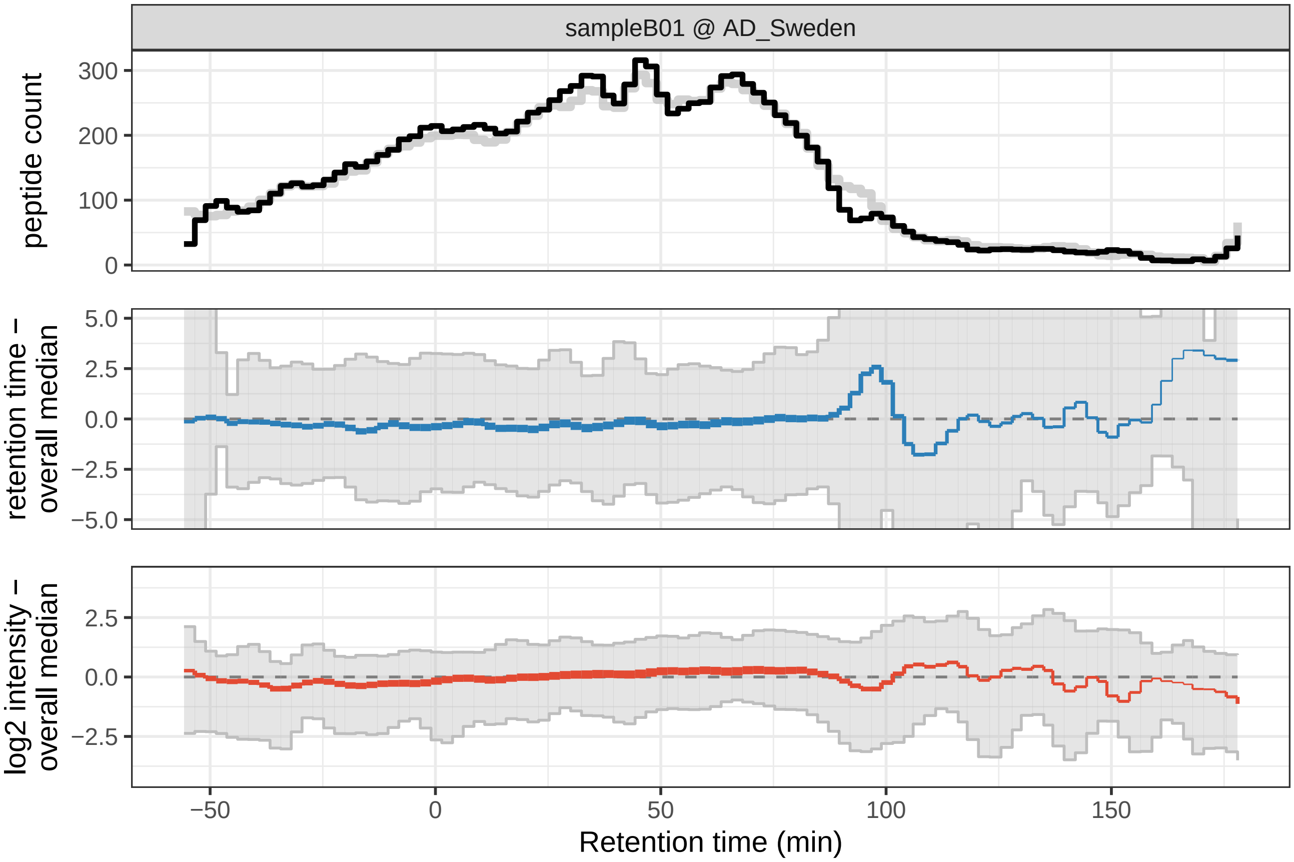
sampleB01 @ AD_Sweden
peptide count
300
200
100
0
retention time –
overall median
5.0
2.5
0.0
−2.5
−5.0
log2 intensity –
overall median
2.5
0.0
−2.5
−50
0
50
100
150
Retention time (min)

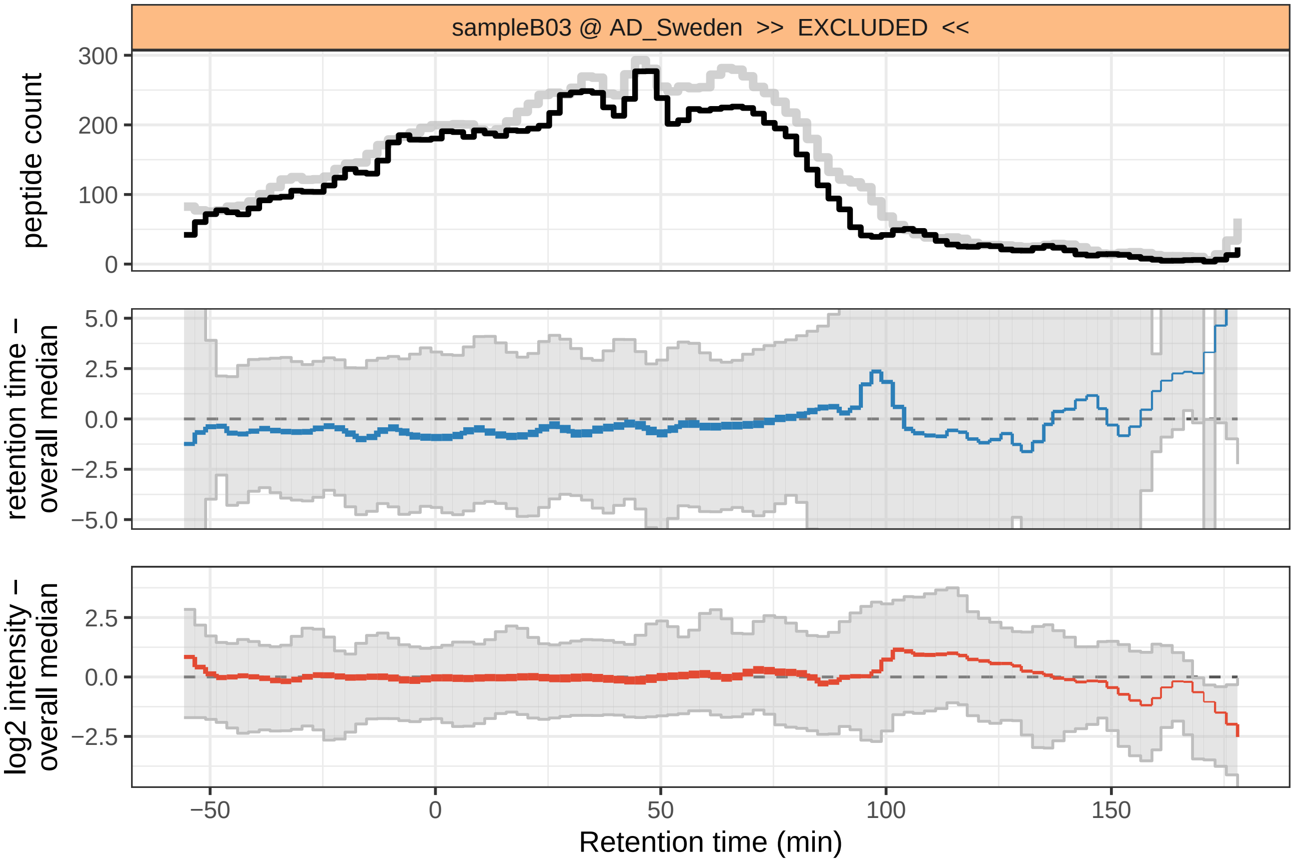
sampleB03 @ AD_Sweden >> EXCLUDED <<
peptide count
300
200
100
0
retention time –
overall median
5.0
2.5
0.0
−2.5
−5.0
log2 intensity –
overall median
2.5
0.0
−2.5
−50
0
50
100
150
Retention time (min)

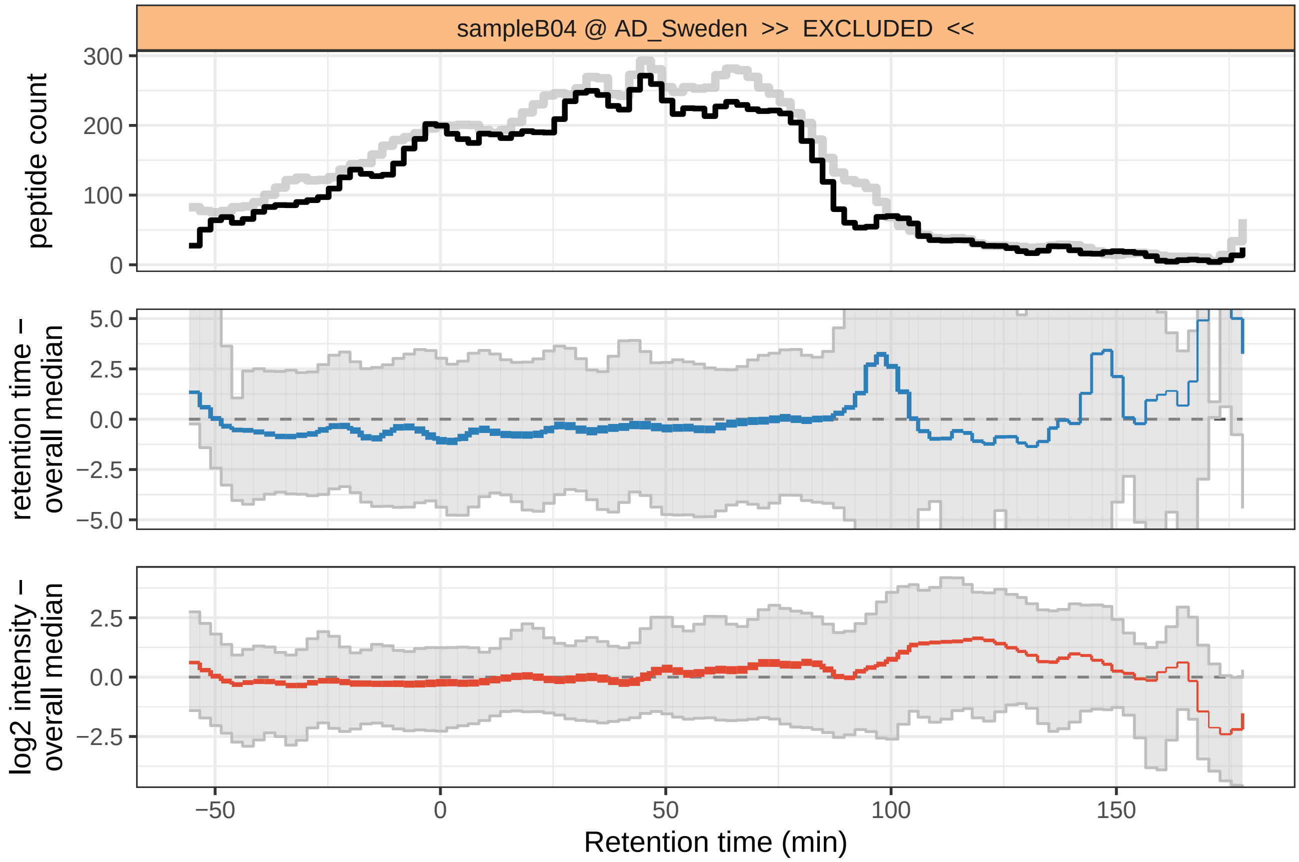
sampleB04 @ AD_Sweden >> EXCLUDED <<
peptide count
retention time – overall median
log2 intensity – overall median
Retention time (min)

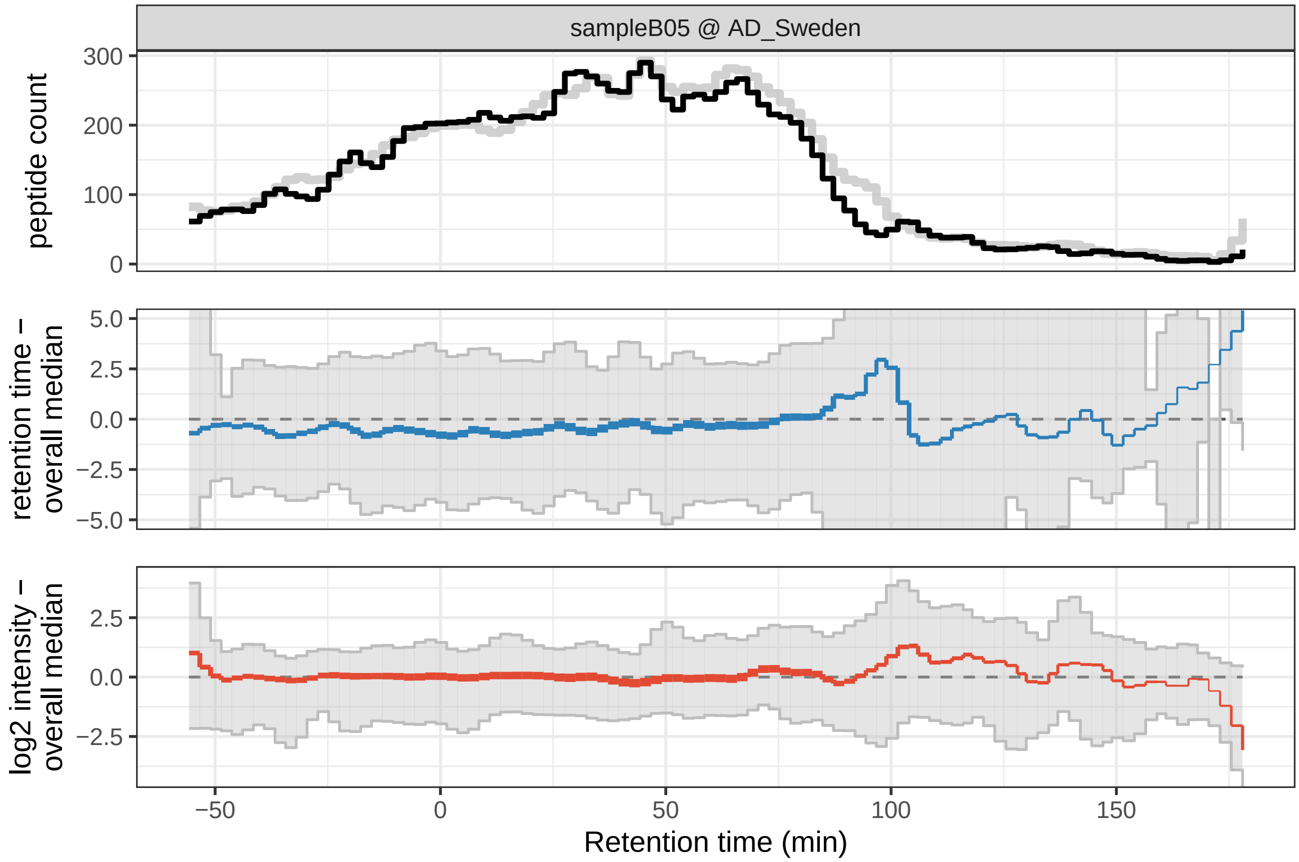
sampleB05 @ AD_Sweden
peptide count
retention time – overall median
log2 intensity – overall median
Retention time (min)

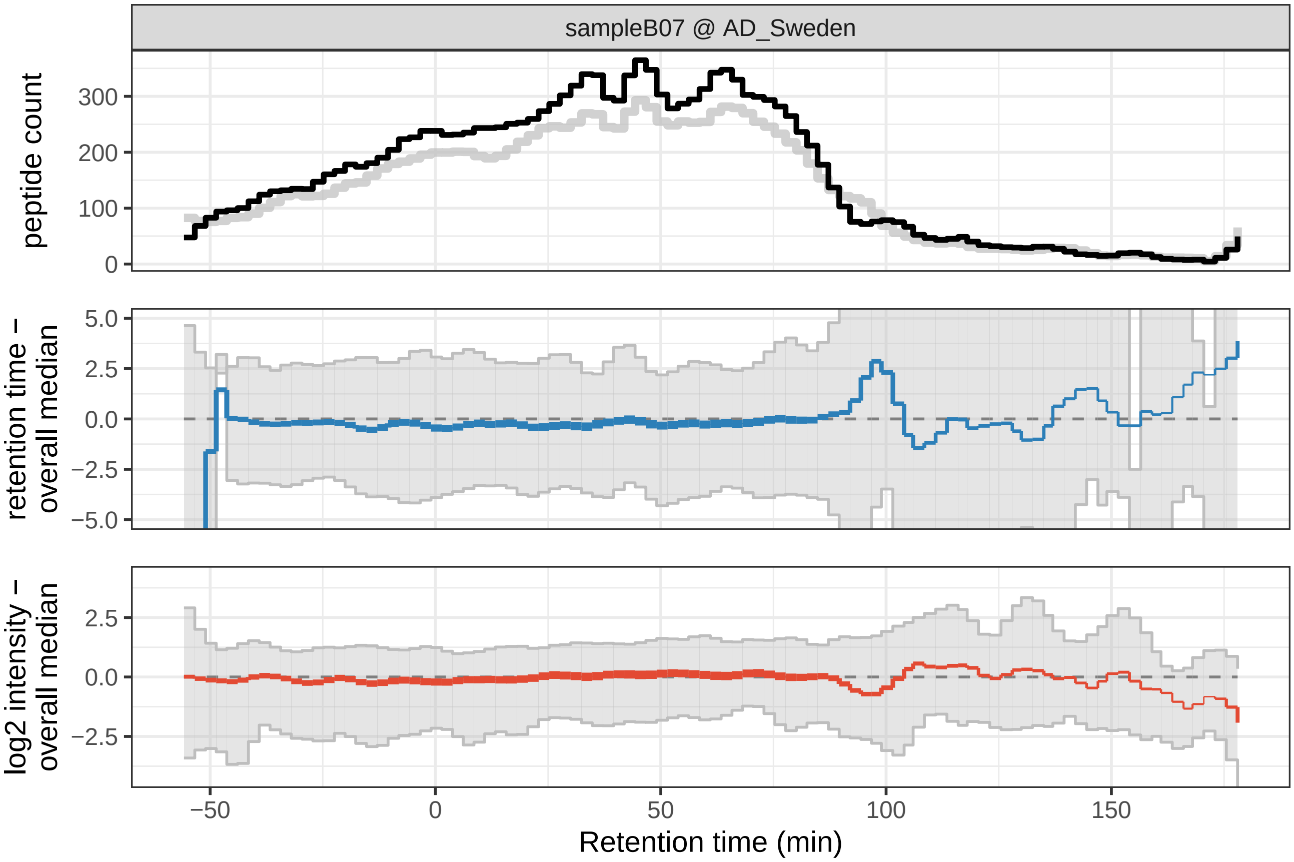
sampleB07 @ AD_Sweden
peptide count
300
200
100
0
retention time –
overall median
5.0
2.5
0.0
−2.5
−5.0
log2 intensity –
overall median
2.5
0.0
−2.5
−50
0
50
100
150
Retention time (min)

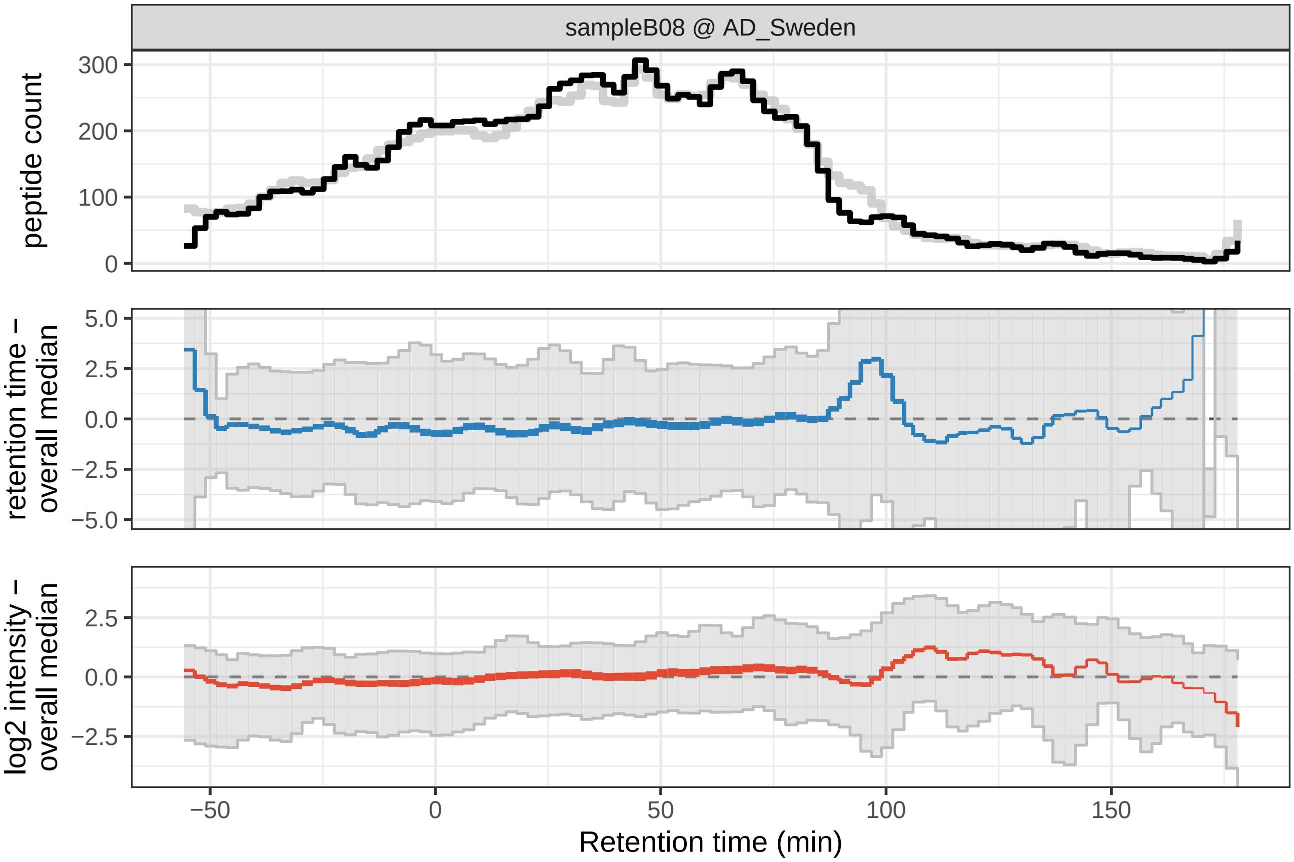
sampleB08 @ AD_Sweden
peptide count
300
200
100
0
retention time –
overall median
5.0
2.5
0.0
−2.5
−5.0
log2 intensity –
overall median
2.5
0.0
−2.5
−50
0
50
100
150
Retention time (min)

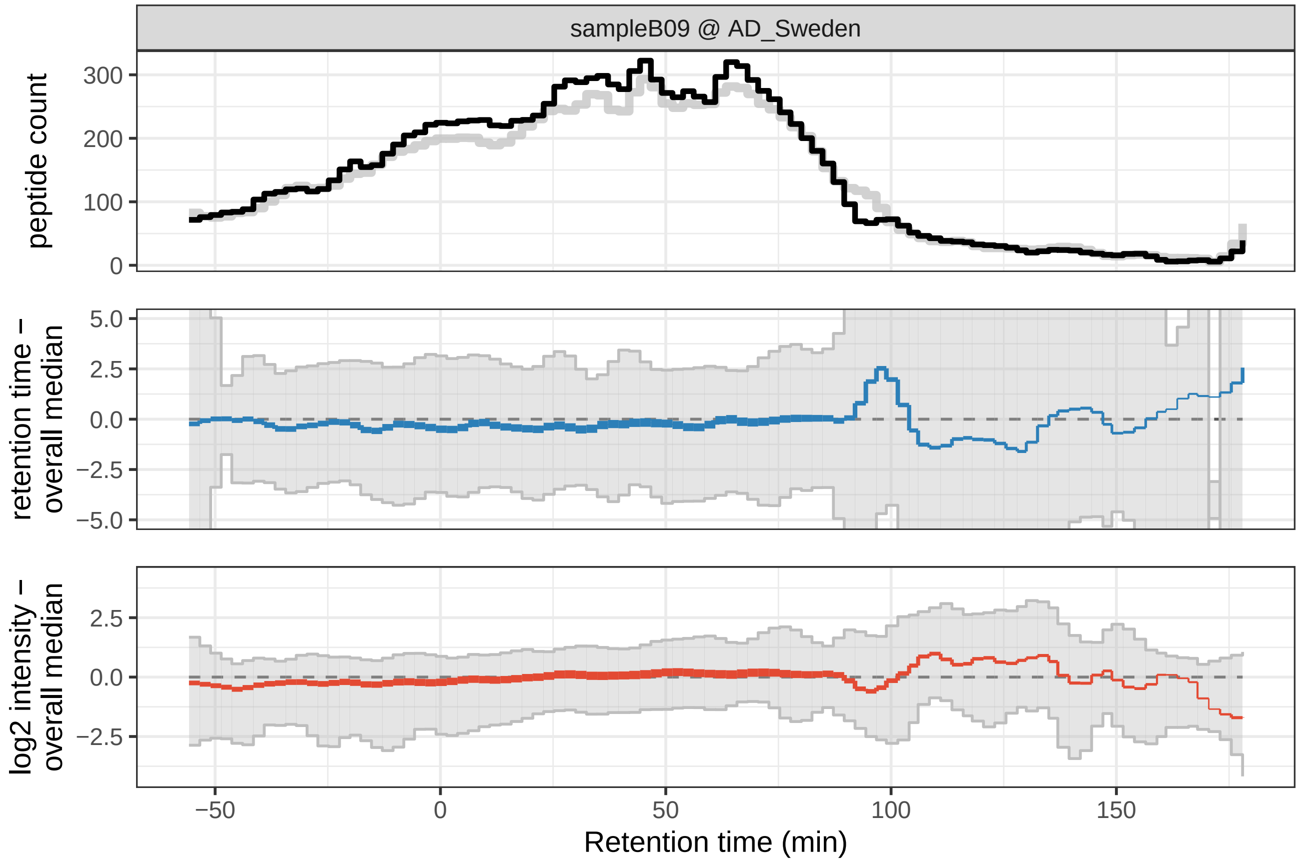
sampleB09 @ AD_Sweden
peptide count
300
200
100
0
retention time – overall median
5.0
2.5
0.0
−2.5
−5.0
log2 intensity – overall median
2.5
0.0
−2.5
−50
0
50
100
150
Retention time (min)

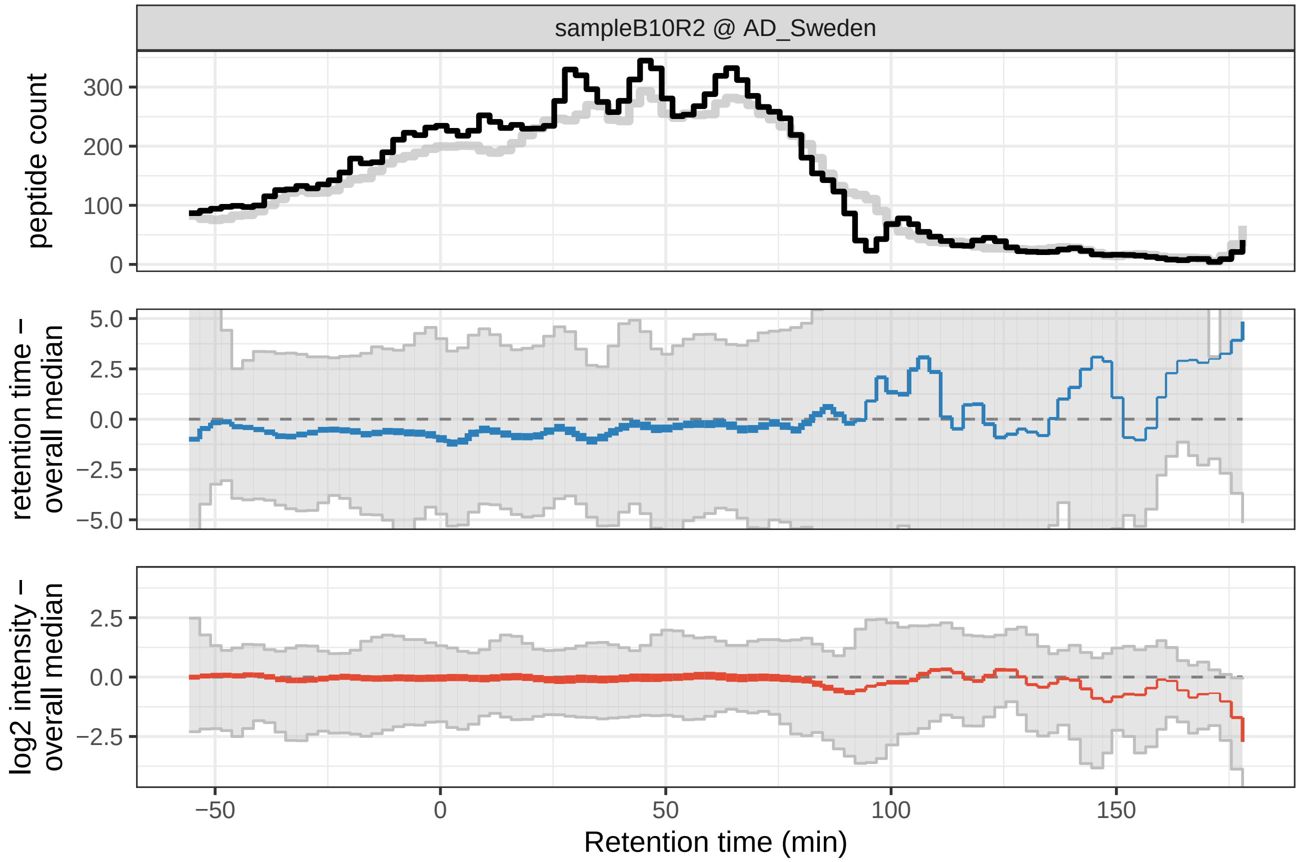
sampleB10R2 @ AD_Sweden
peptide count
300
200
100
0
retention time – overall median
5.0
2.5
0.0
−2.5
−5.0
log2 intensity – overall median
2.5
0.0
−2.5
−50
0
50
100
150
Retention time (min)

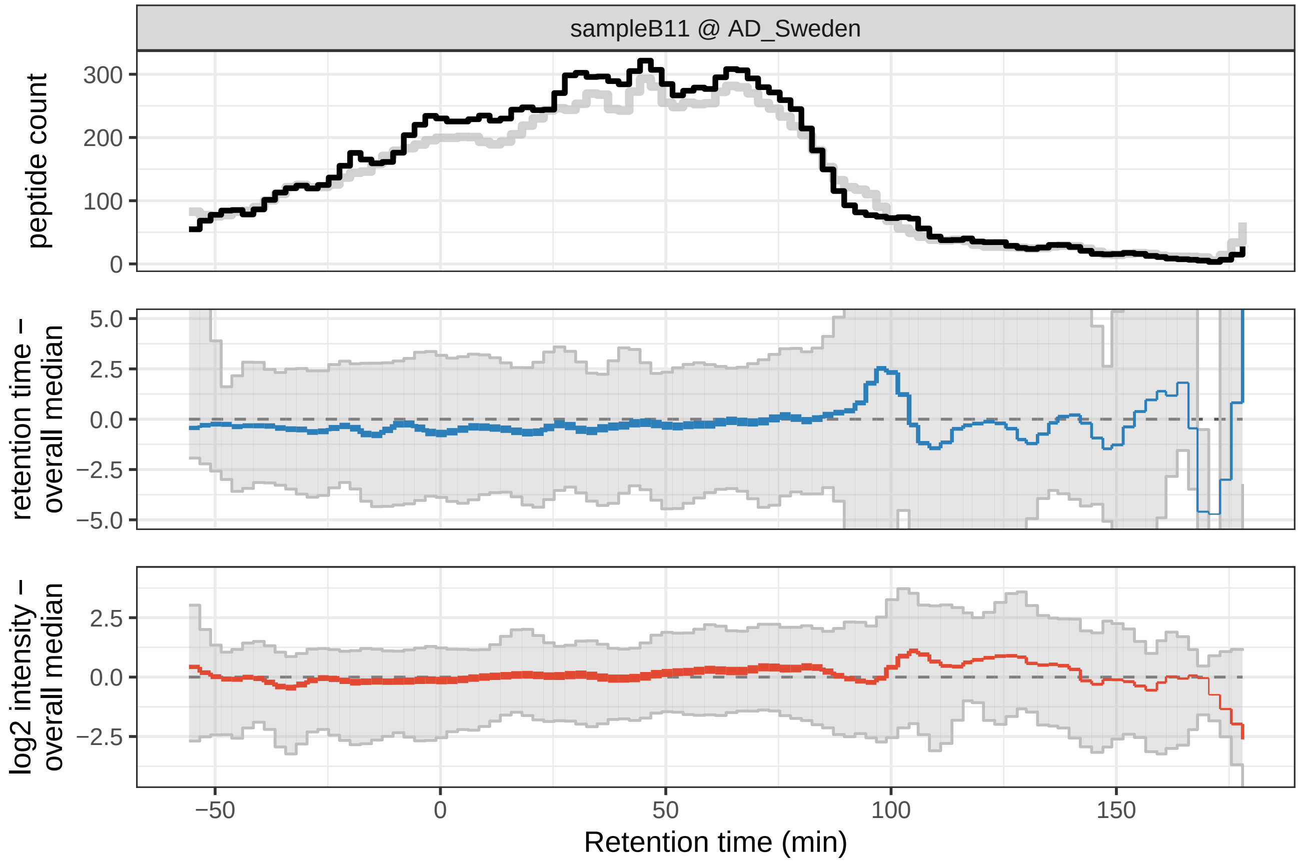
sampleB11 @ AD_Sweden
peptide count
300
200
100
0
retention time – overall median
5.0
2.5
0.0
−2.5
−5.0
log2 intensity – overall median
2.5
0.0
−2.5
−50
0
50
100
150
Retention time (min)

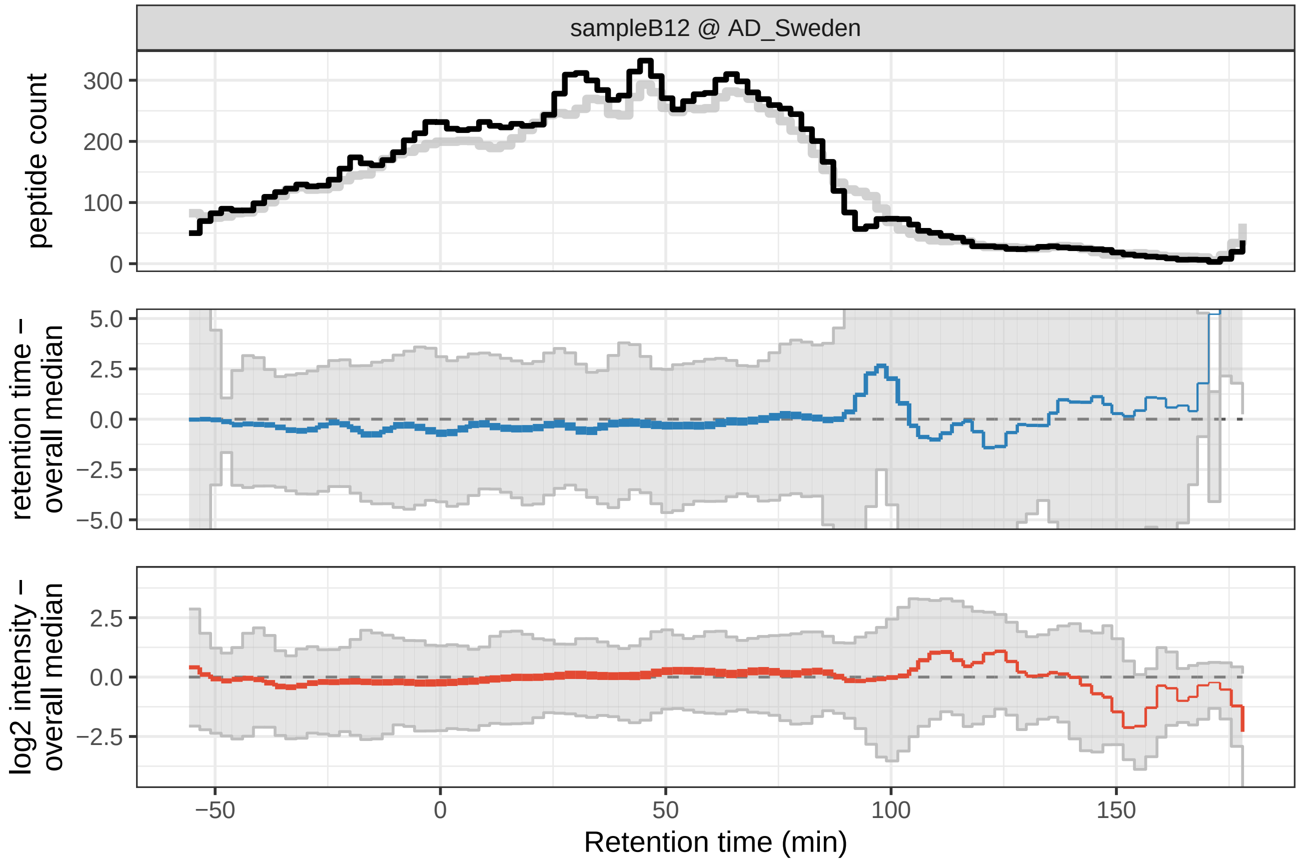
sampleB12 @ AD_Sweden
peptide count
300
200
100
0
retention time – overall median
5.0
2.5
0.0
−2.5
−5.0
log2 intensity – overall median
2.5
0.0
−2.5
−50
0
50
100
150
Retention time (min)

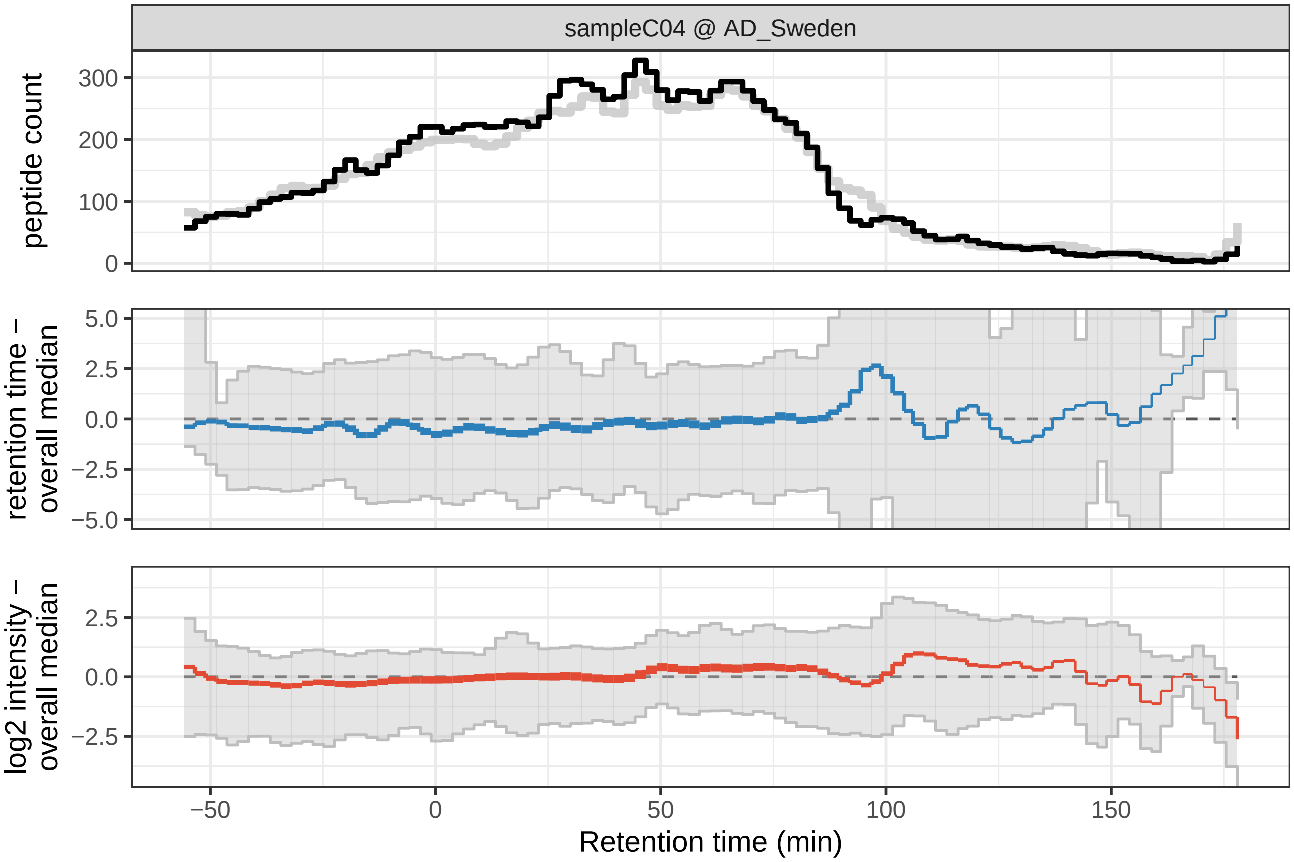

sampleC04 @ AD_Sweden
peptide count
retention time – overall median
log2 intensity – overall median
Retention time (min)

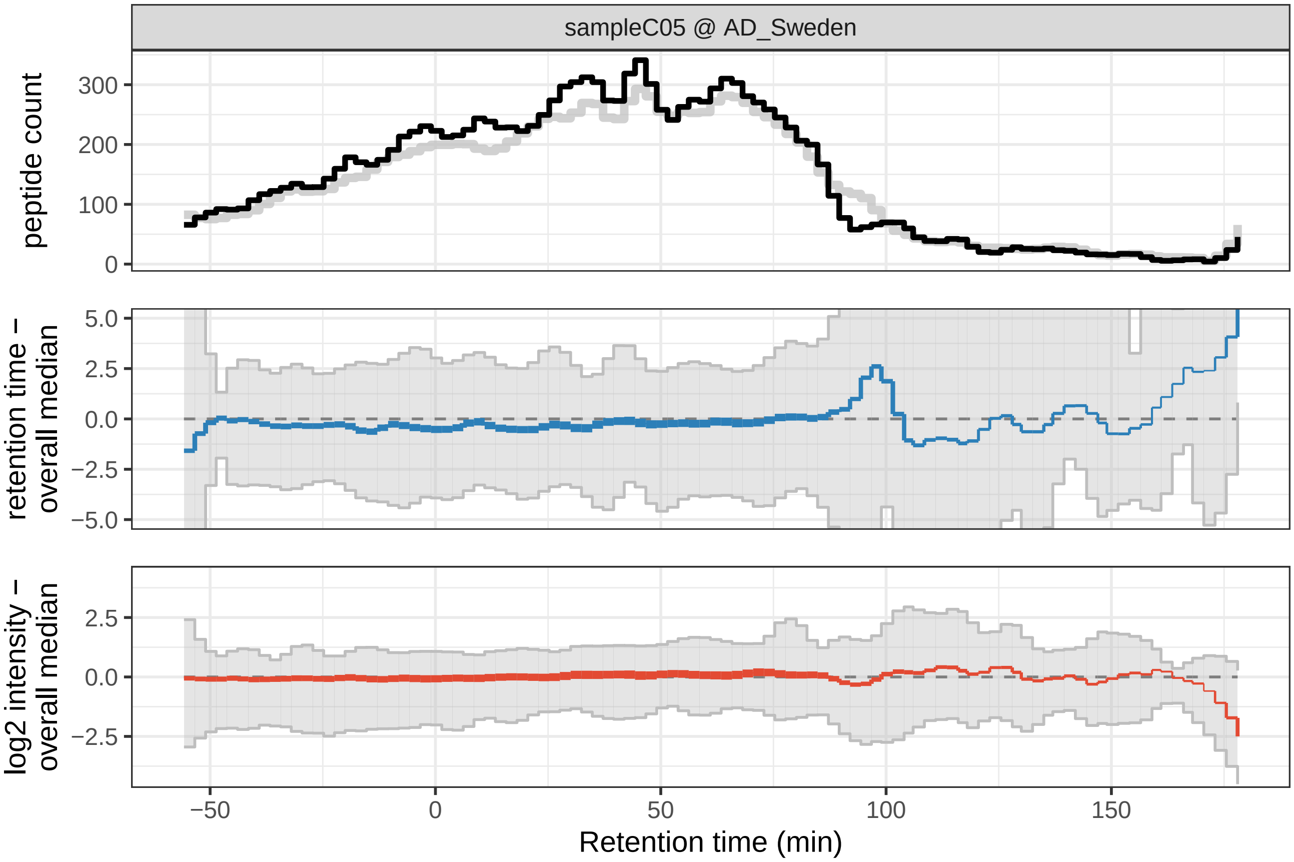

sampleC05 @ AD_Sweden
peptide count
retention time – overall median
log2 intensity – overall median
Retention time (min)

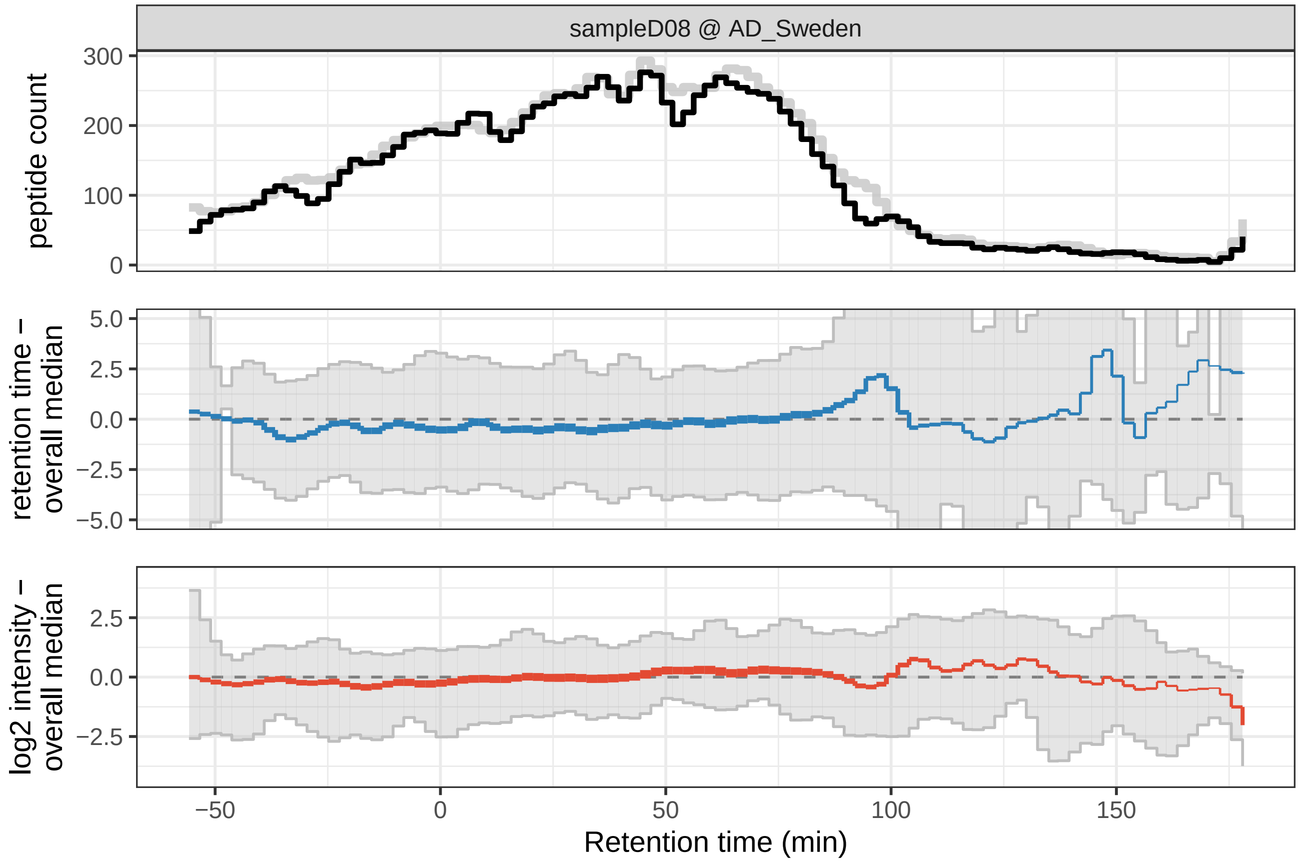

sampleD08 @ AD_Sweden
peptide count
retention time – overall median
log2 intensity – overall median
Retention time (min)

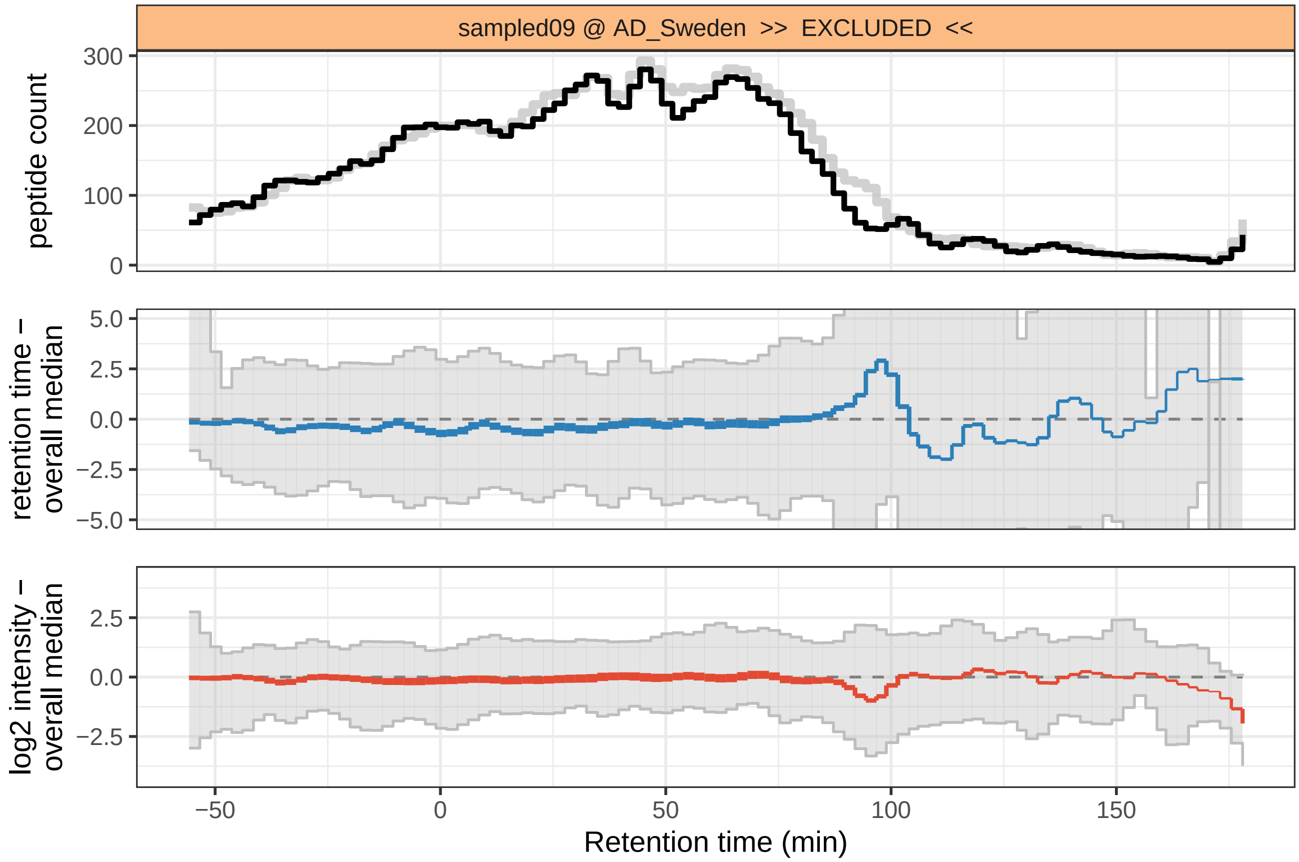

sampled09 @ AD_Sweden >> EXCLUDED <<
peptide count
retention time – overall median
log2 intensity – overall median
Retention time (min)

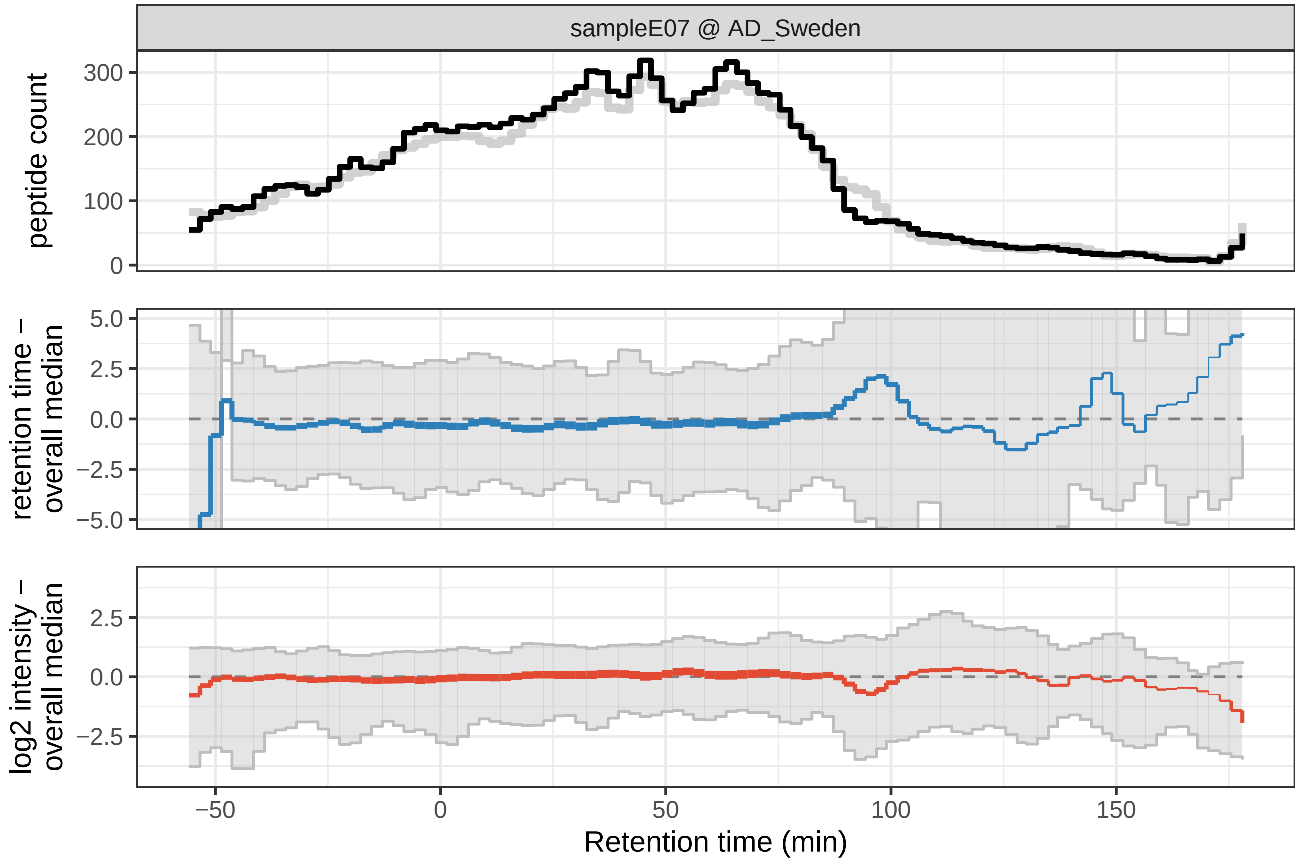
sampleE07 @ AD_Sweden
peptide count
300
200
100
0
retention time –
overall median
5.0
2.5
0.0
−2.5
−5.0
log2 intensity –
overall median
2.5
0.0
−2.5
−50
0
50
100
150
Retention time (min)

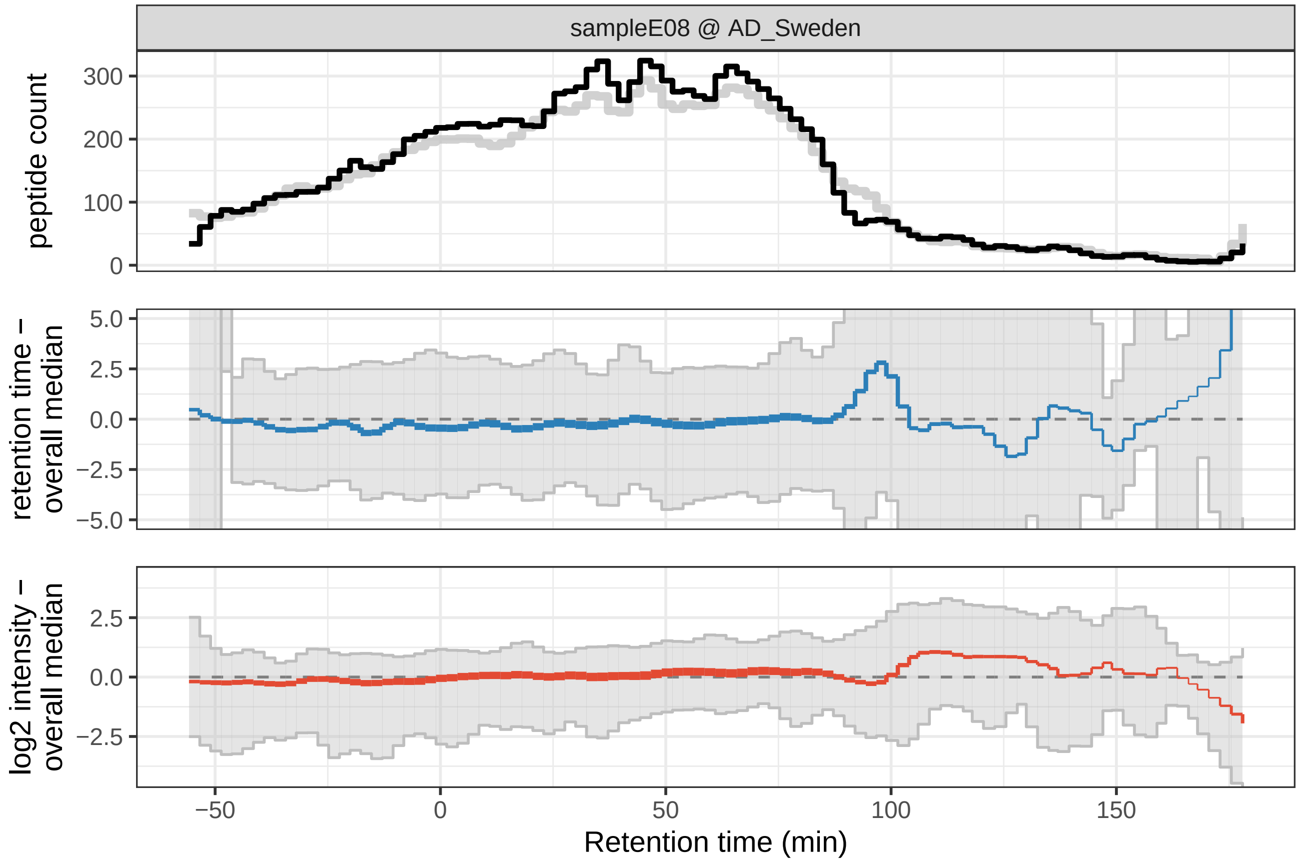
sampleE08 @ AD_Sweden
peptide count
300
200
100
0
retention time –
overall median
5.0
2.5
0.0
−2.5
−5.0
log2 intensity –
overall median
2.5
0.0
−2.5
−50
0
50
100
150
Retention time (min)

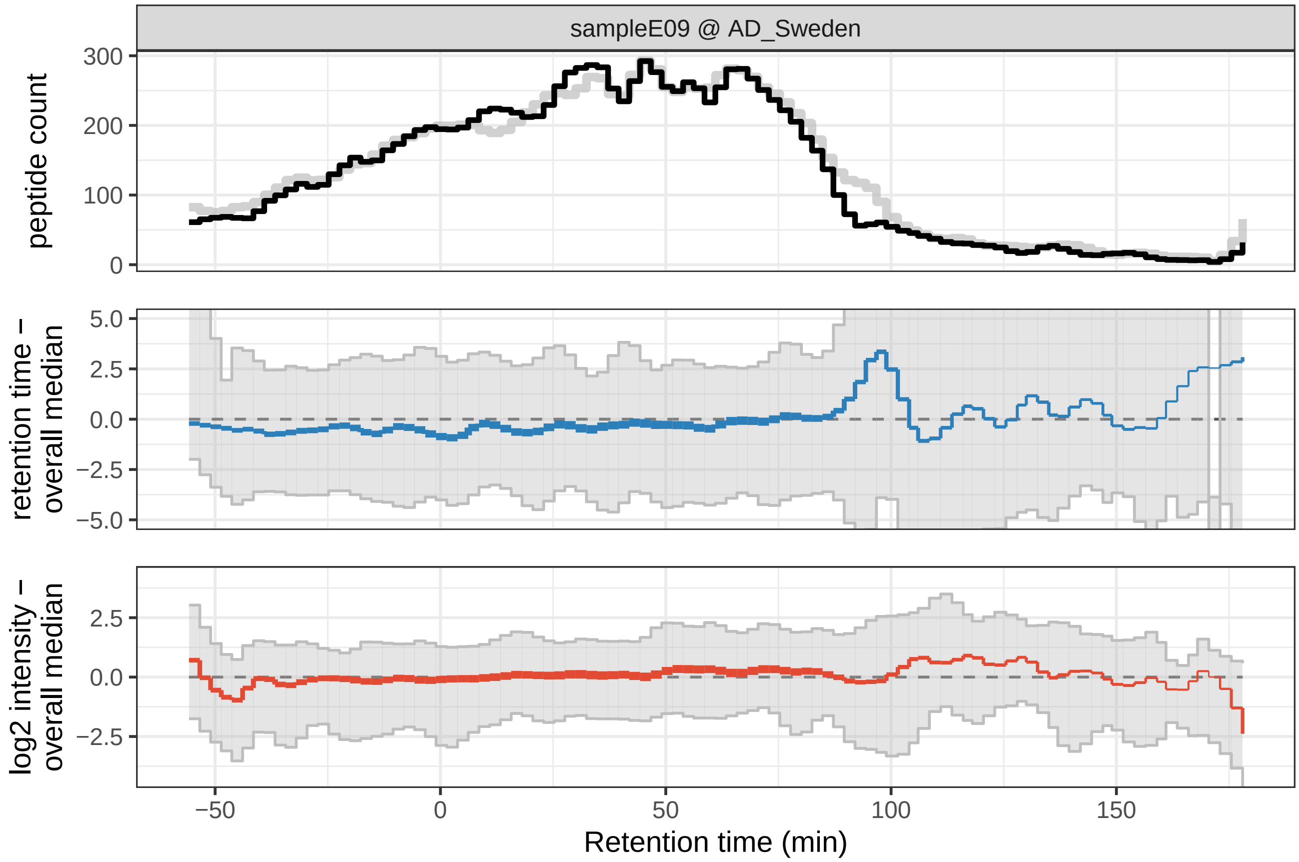
sampleE09 @ AD_Sweden
peptide count
retention time – overall median
log2 intensity – overall median
Retention time (min)

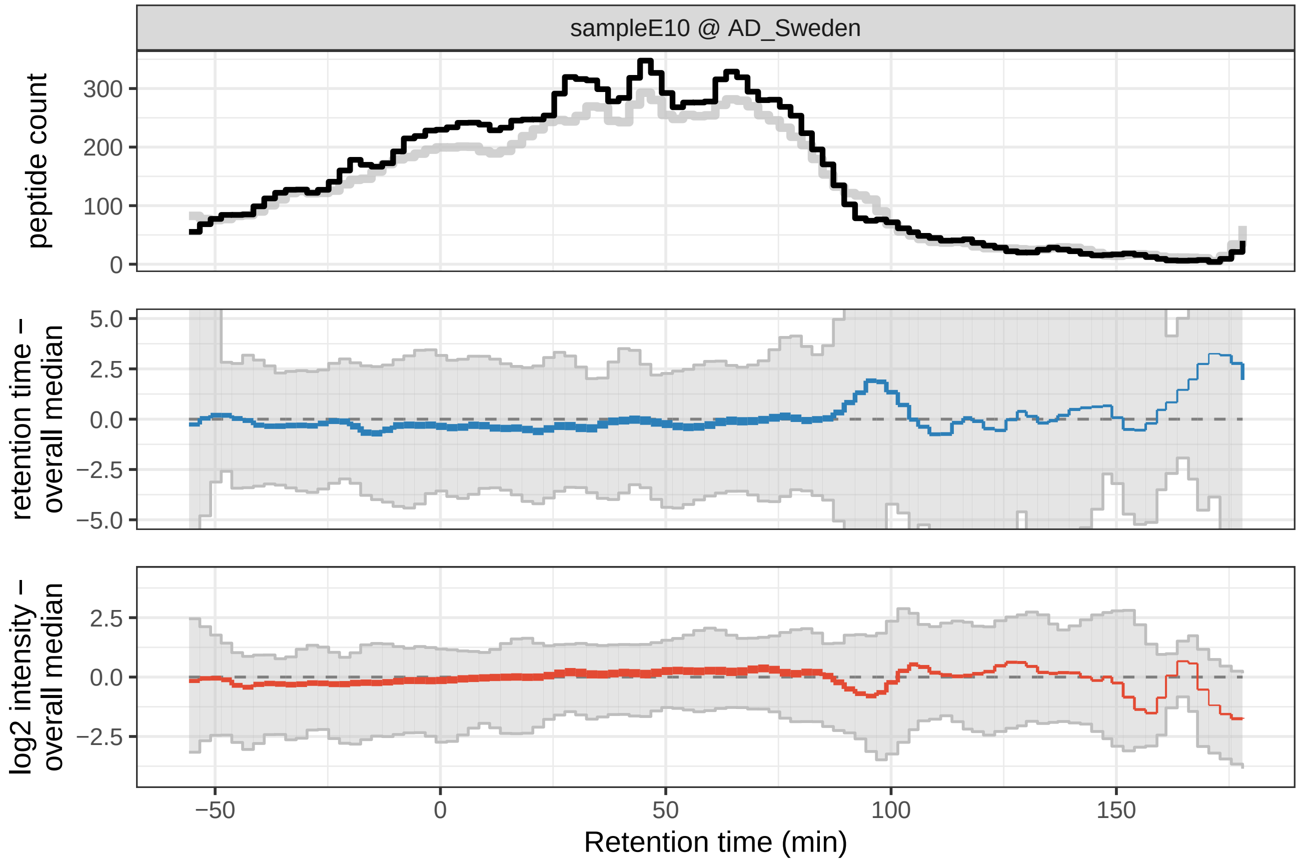
sampleE10 @ AD_Sweden
peptide count
retention time – overall median
log2 intensity – overall median
Retention time (min)

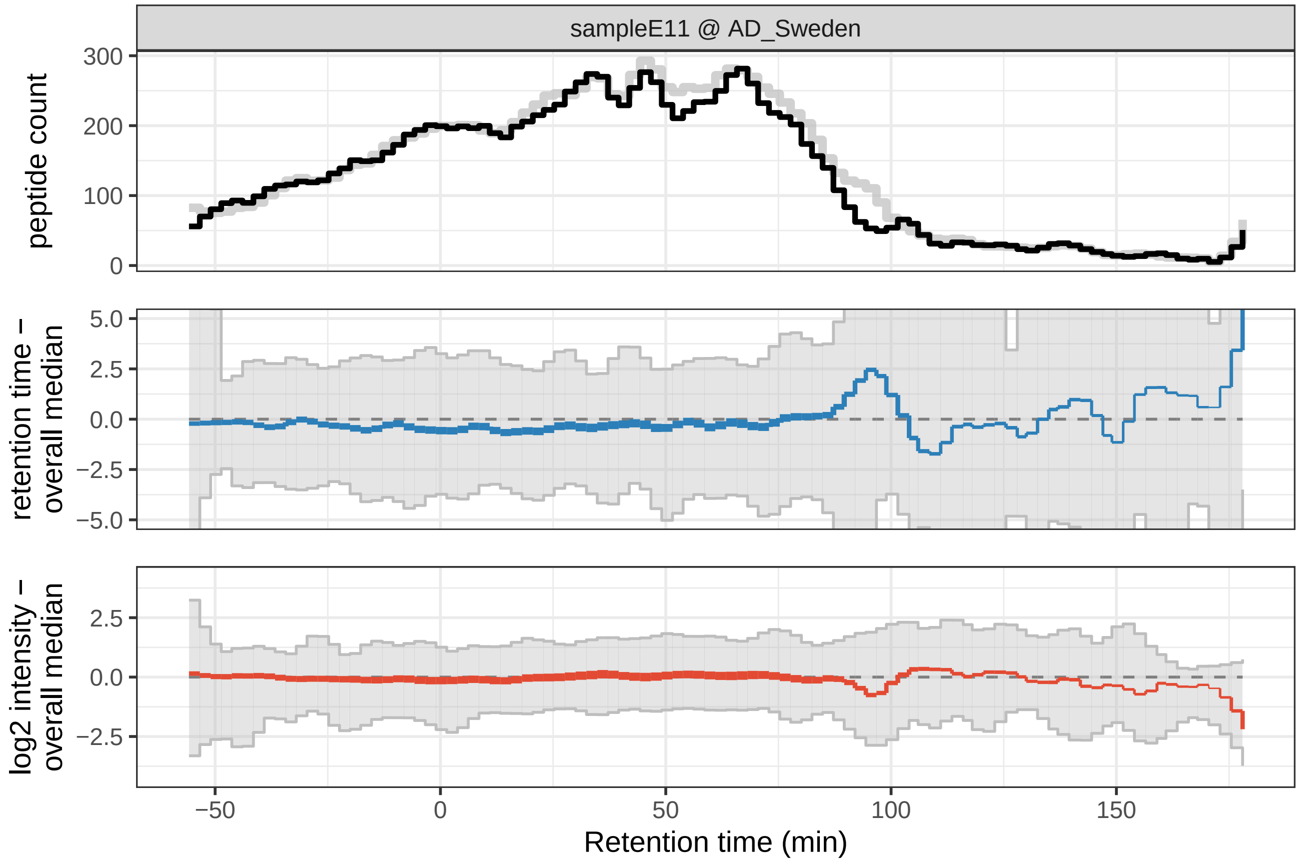
sampleE11 @ AD_Sweden
peptide count
retention time – overall median
log2 intensity – overall median
Retention time (min)

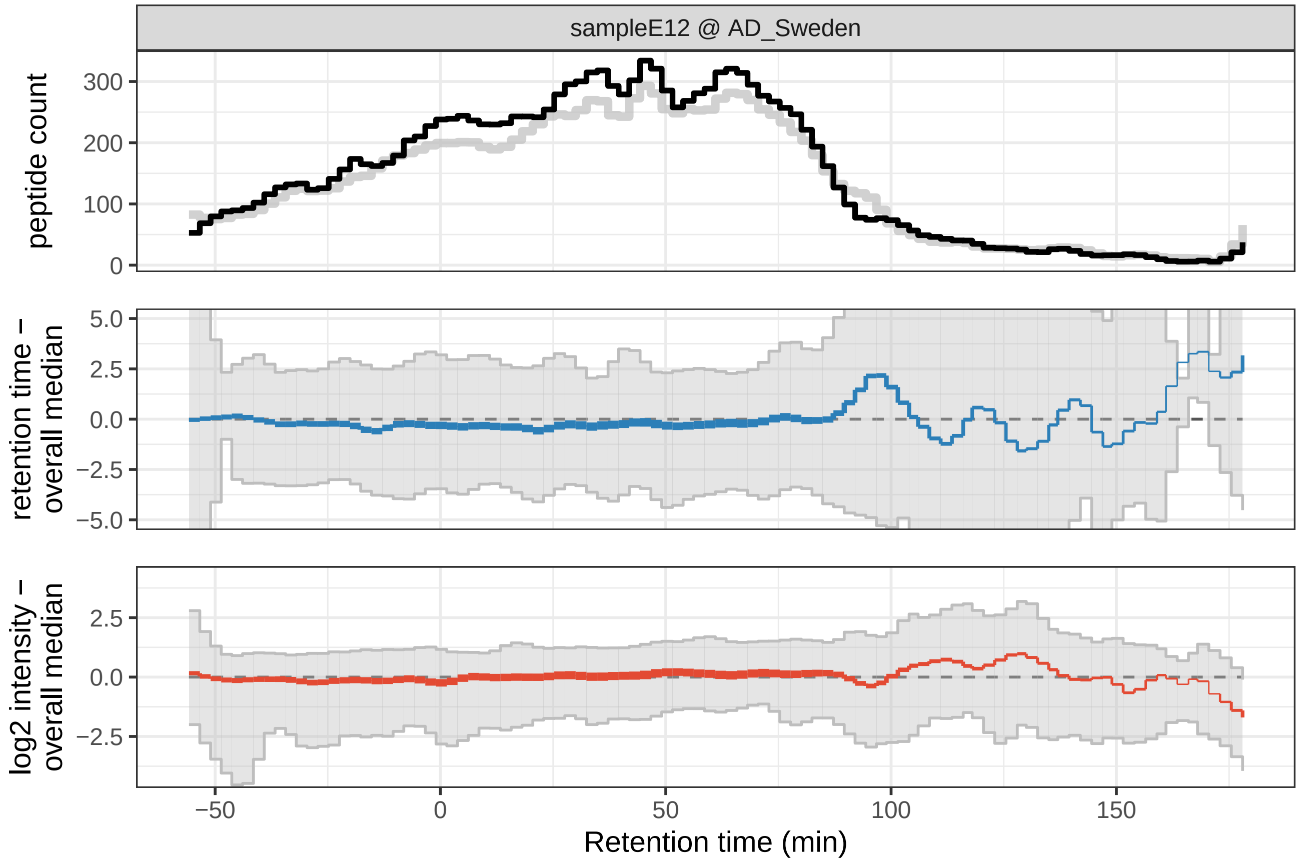
sampleE12 @ AD_Sweden
peptide count
retention time – overall median
log2 intensity – overall median
Retention time (min)

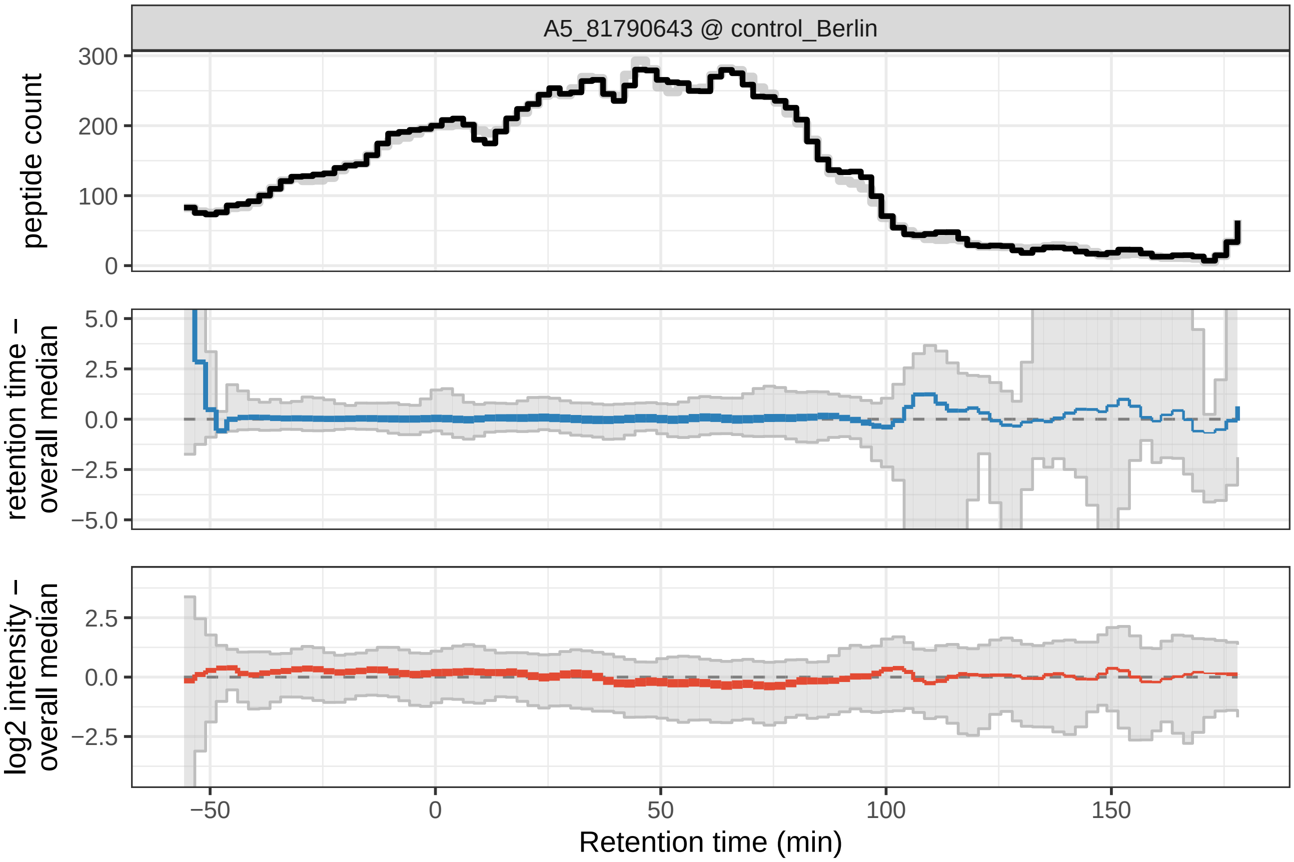
A5_81790643 @ control_Berlin
peptide count
retention time – overall median
log2 intensity – overall median
Retention time (min)

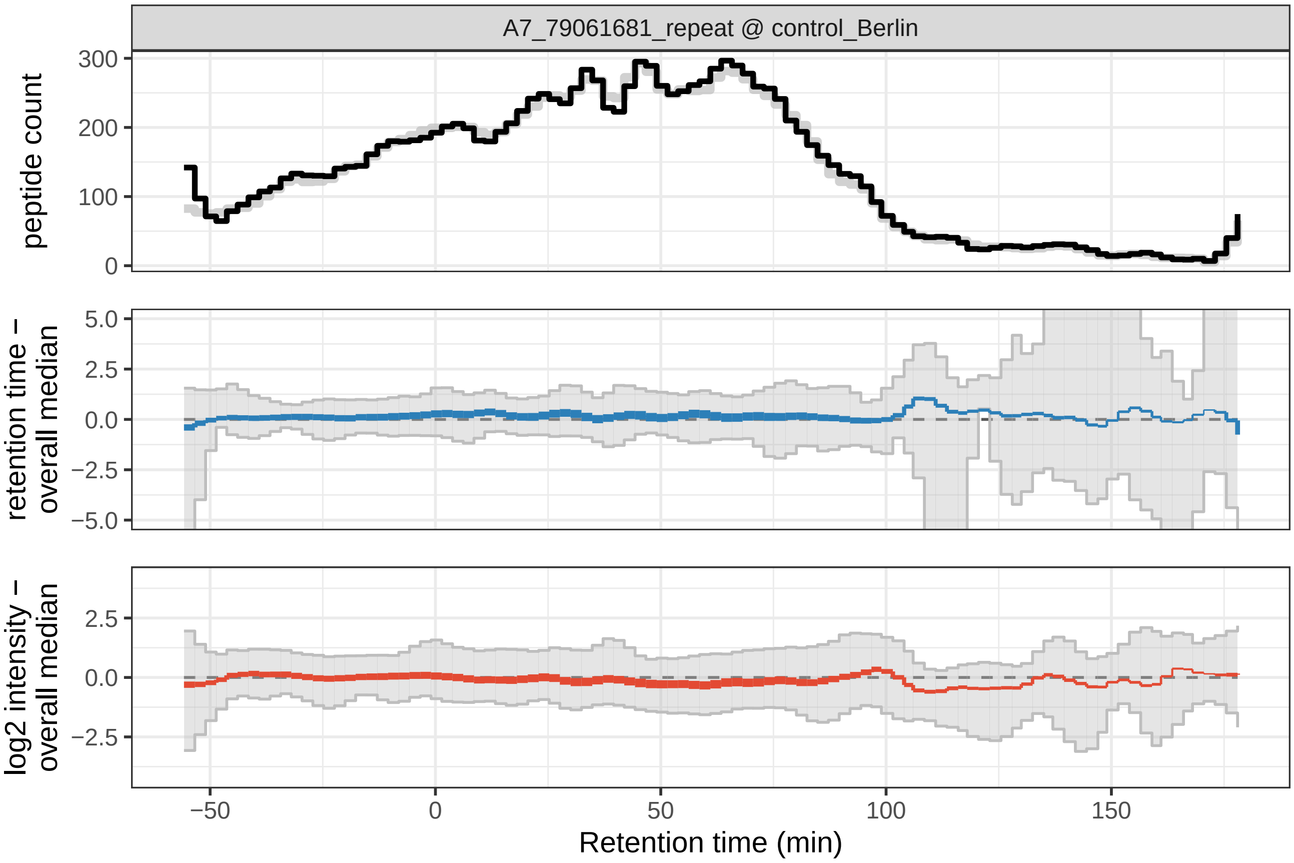
A7_79061681_repeat @ control_Berlin
peptide count
retention time – overall median
log2 intensity – overall median
Retention time (min)

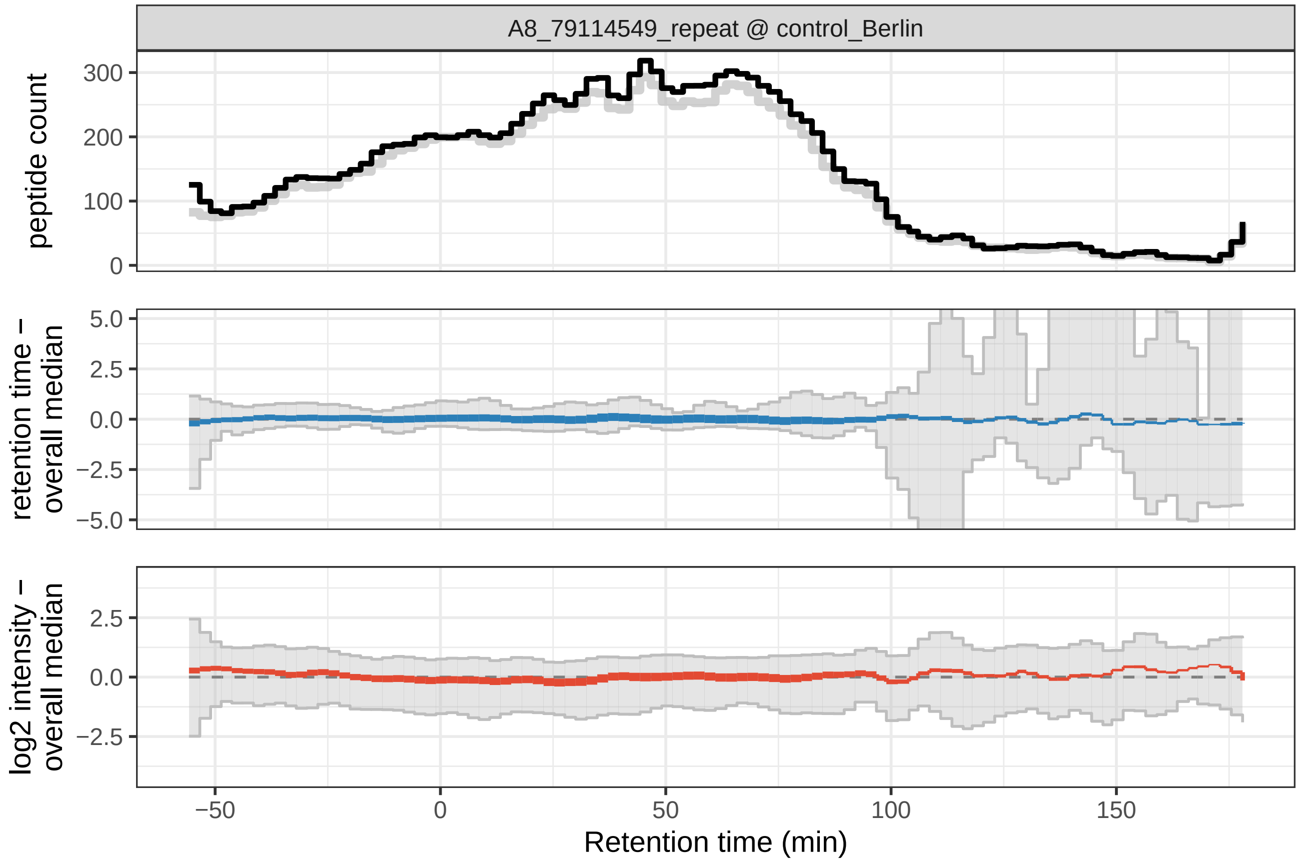
A8_79114549_repeat @ control_Berlin
peptide count
300
200
100
0
retention time –
overall median
5.0
2.5
0.0
−2.5
−5.0
log2 intensity –
overall median
2.5
0.0
−2.5
−50
0
50
100
150
Retention time (min)

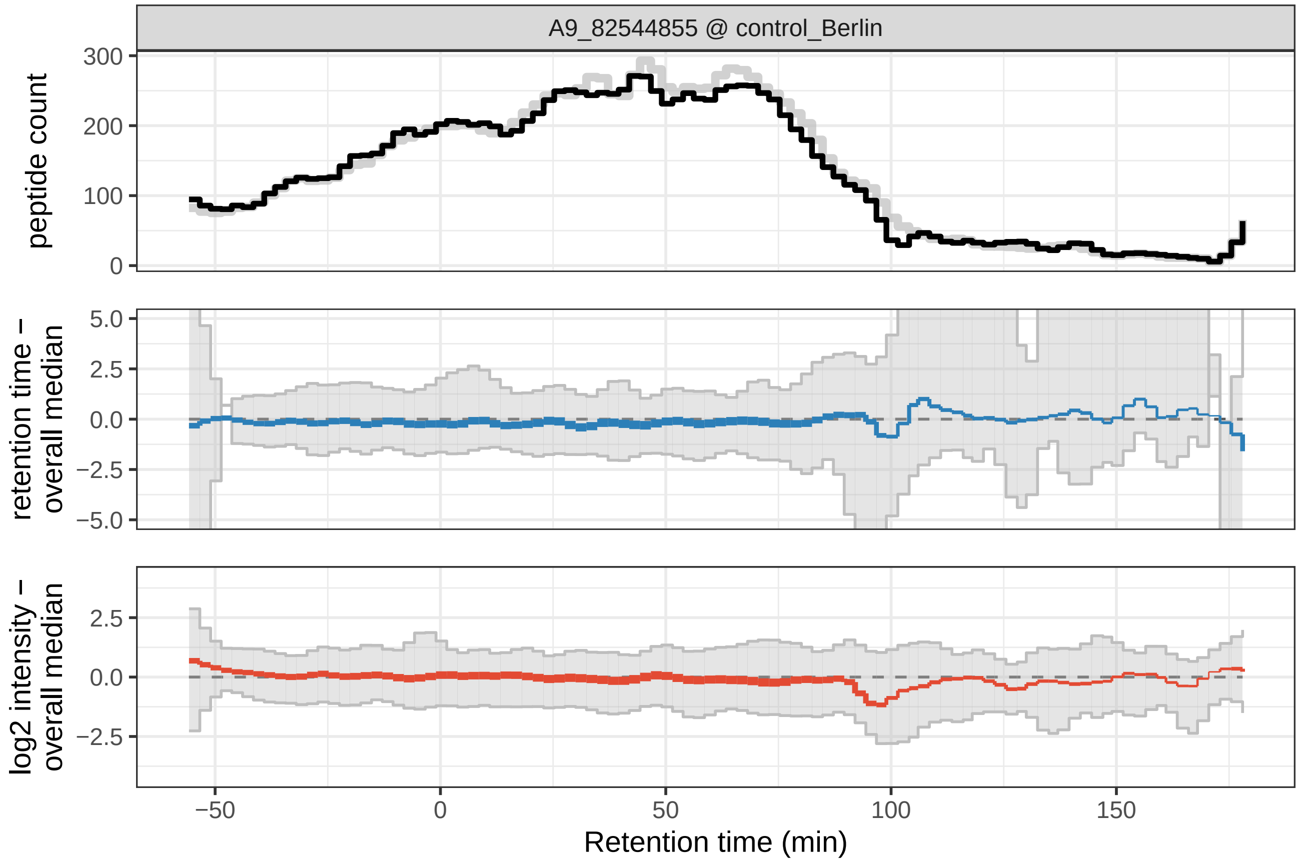
A9_82544855 @ control_Berlin
peptide count
300
200
100
0
retention time –
overall median
5.0
2.5
0.0
−2.5
−5.0
log2 intensity –
overall median
2.5
0.0
−2.5
−50
0
50
100
150
Retention time (min)

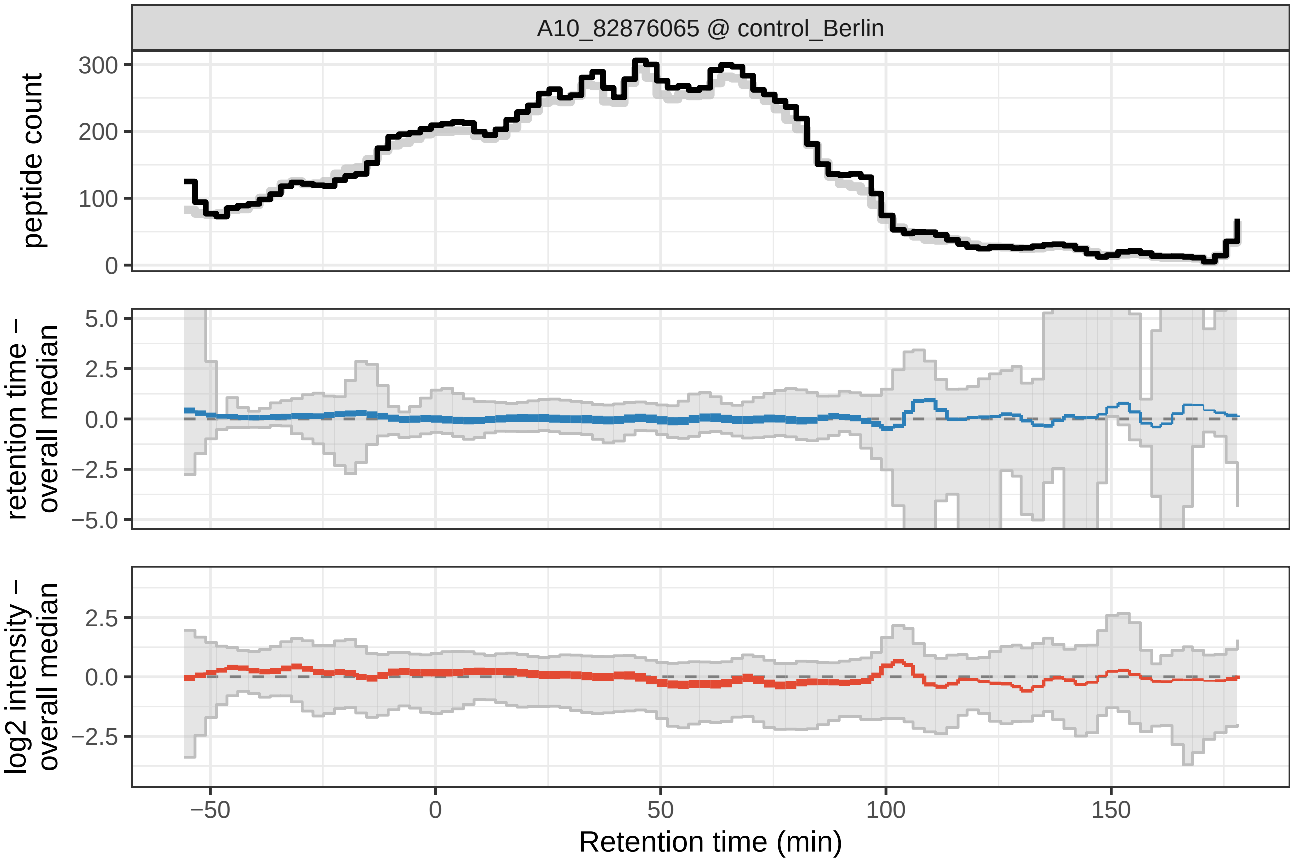
A10_82876065 @ control_Berlin
peptide count
retention time – overall median
log2 intensity – overall median
Retention time (min)

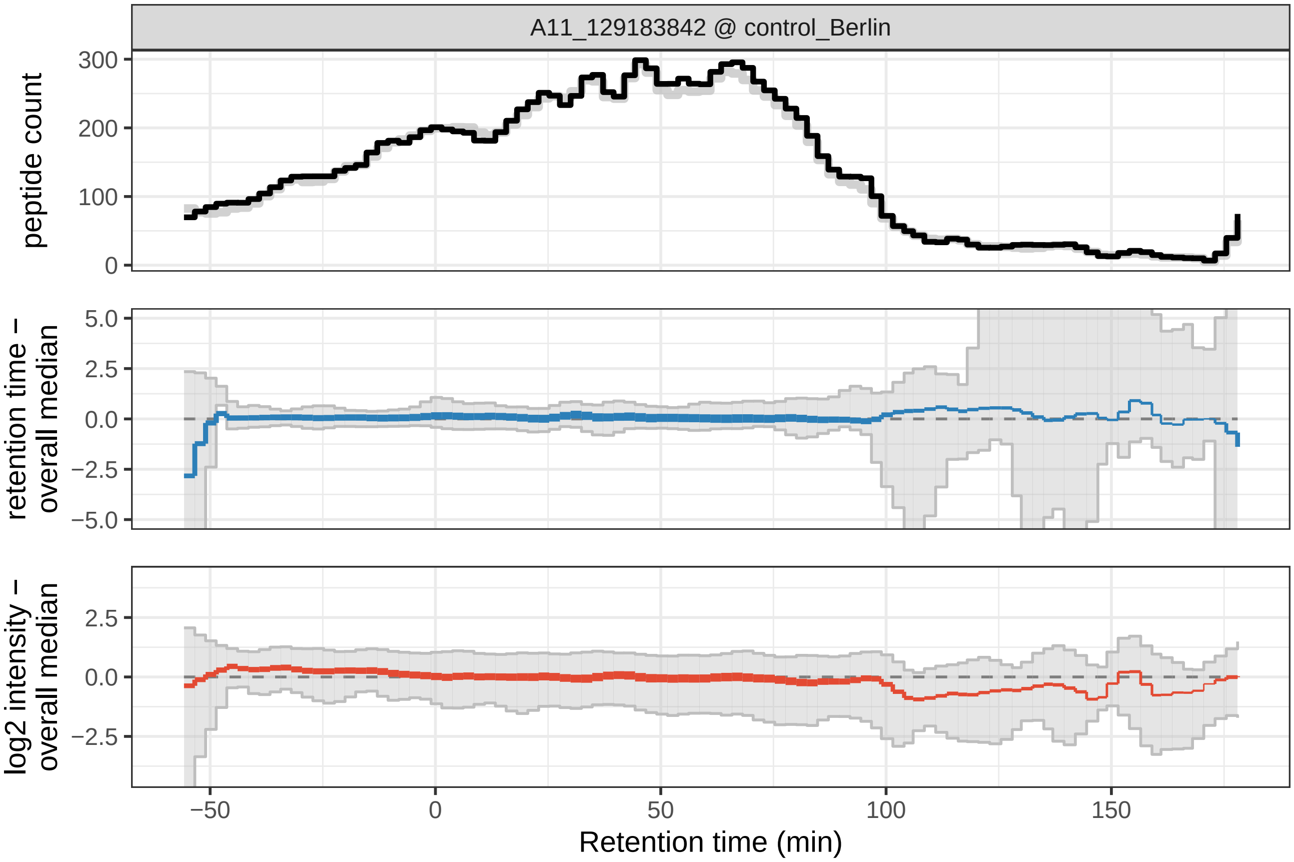
A11_129183842 @ control_Berlin
peptide count
retention time – overall median
log2 intensity – overall median
Retention time (min)

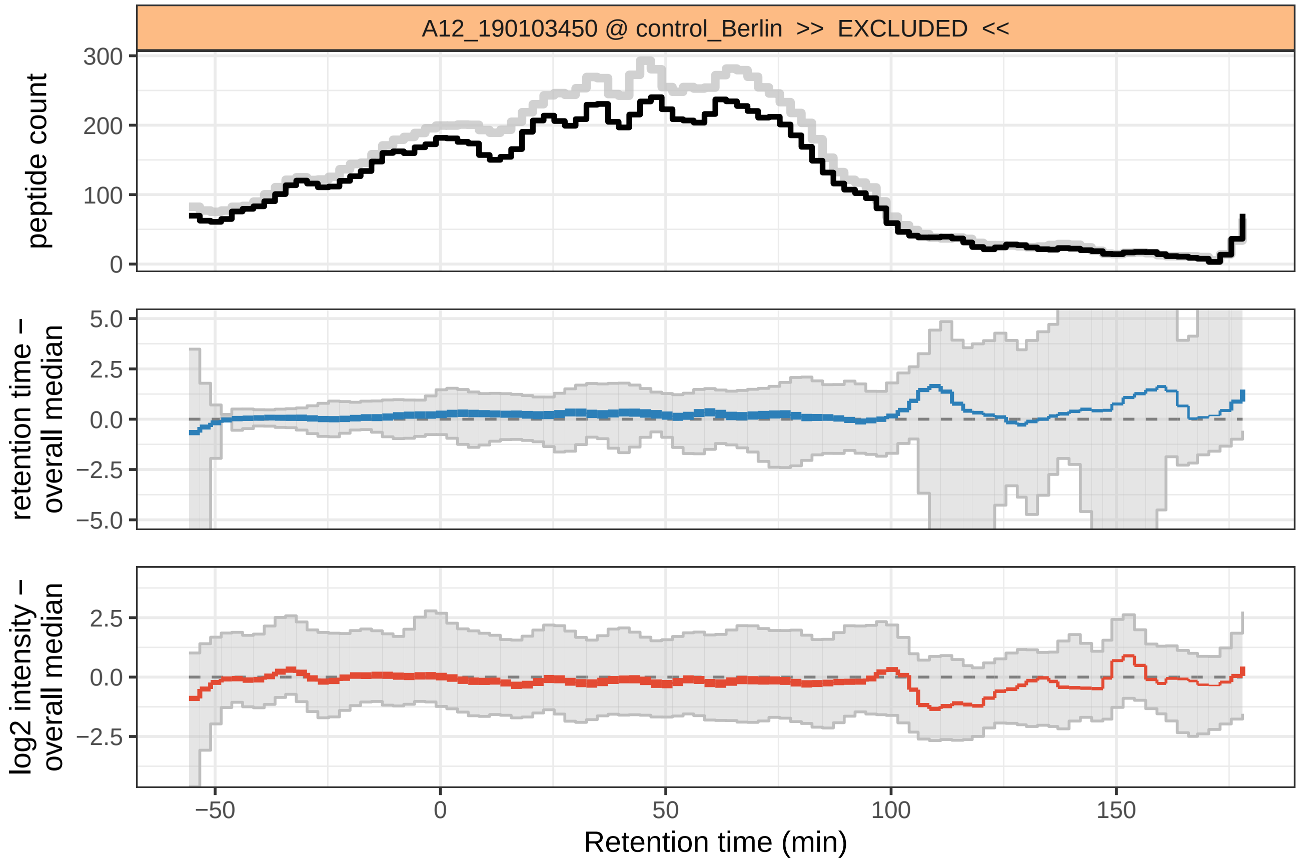
A12_190103450 @ control_Berlin >> EXCLUDED <<
peptide count
retention time – overall median
log2 intensity – overall median
Retention time (min)

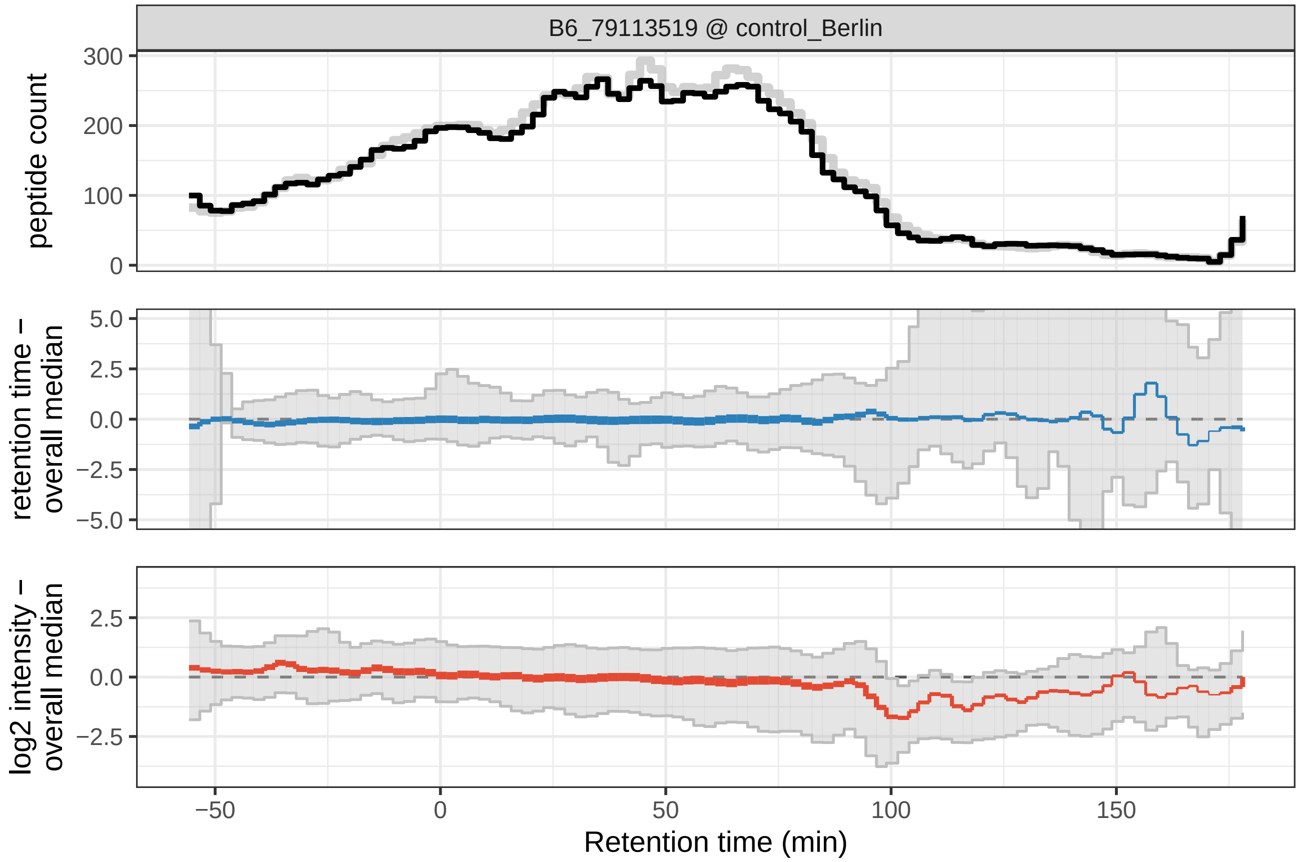
B6_79113519 @ control_Berlin
peptide count
retention time – overall median
log2 intensity – overall median
Retention time (min)

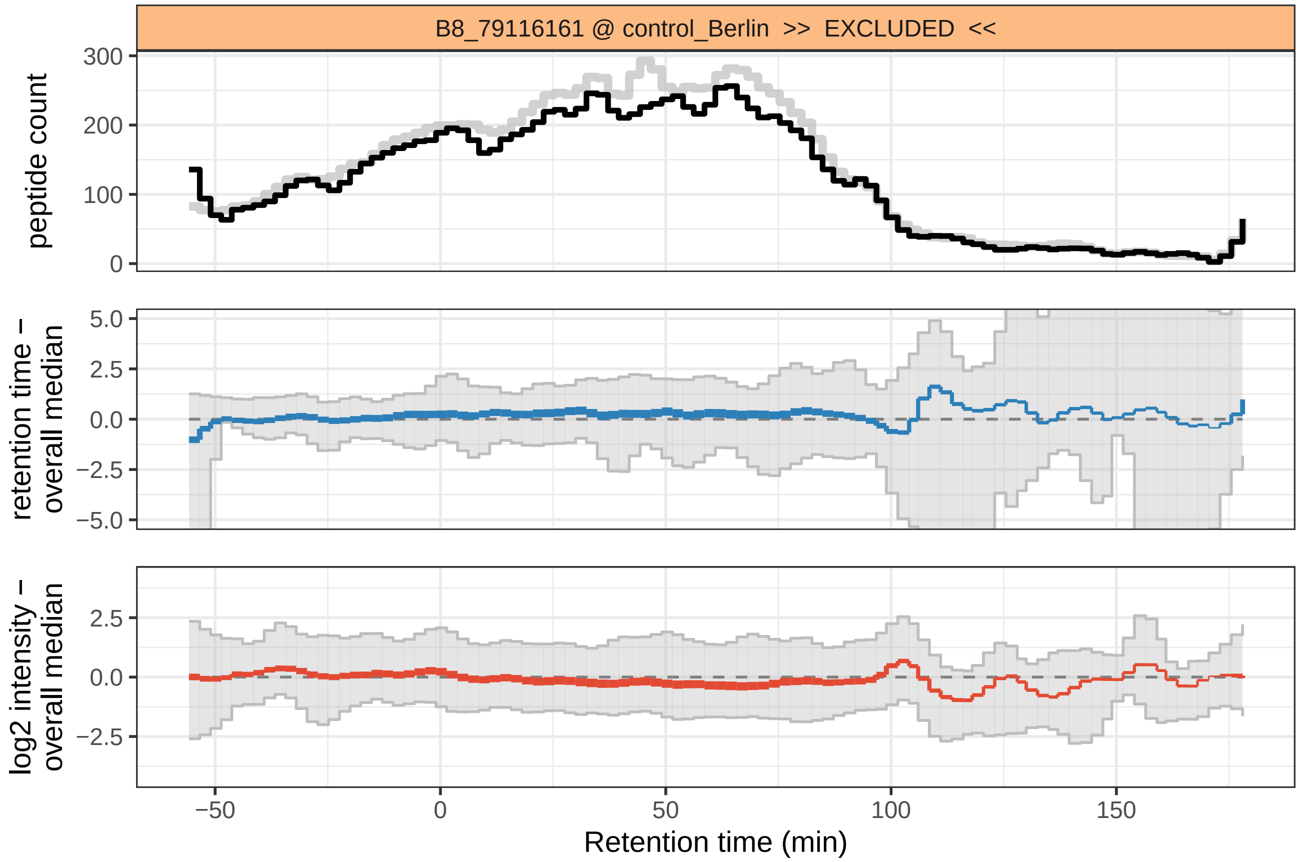
B8_79116161 @ control_Berlin >> EXCLUDED <<
peptide count
retention time – overall median
log2 intensity – overall median
Retention time (min)

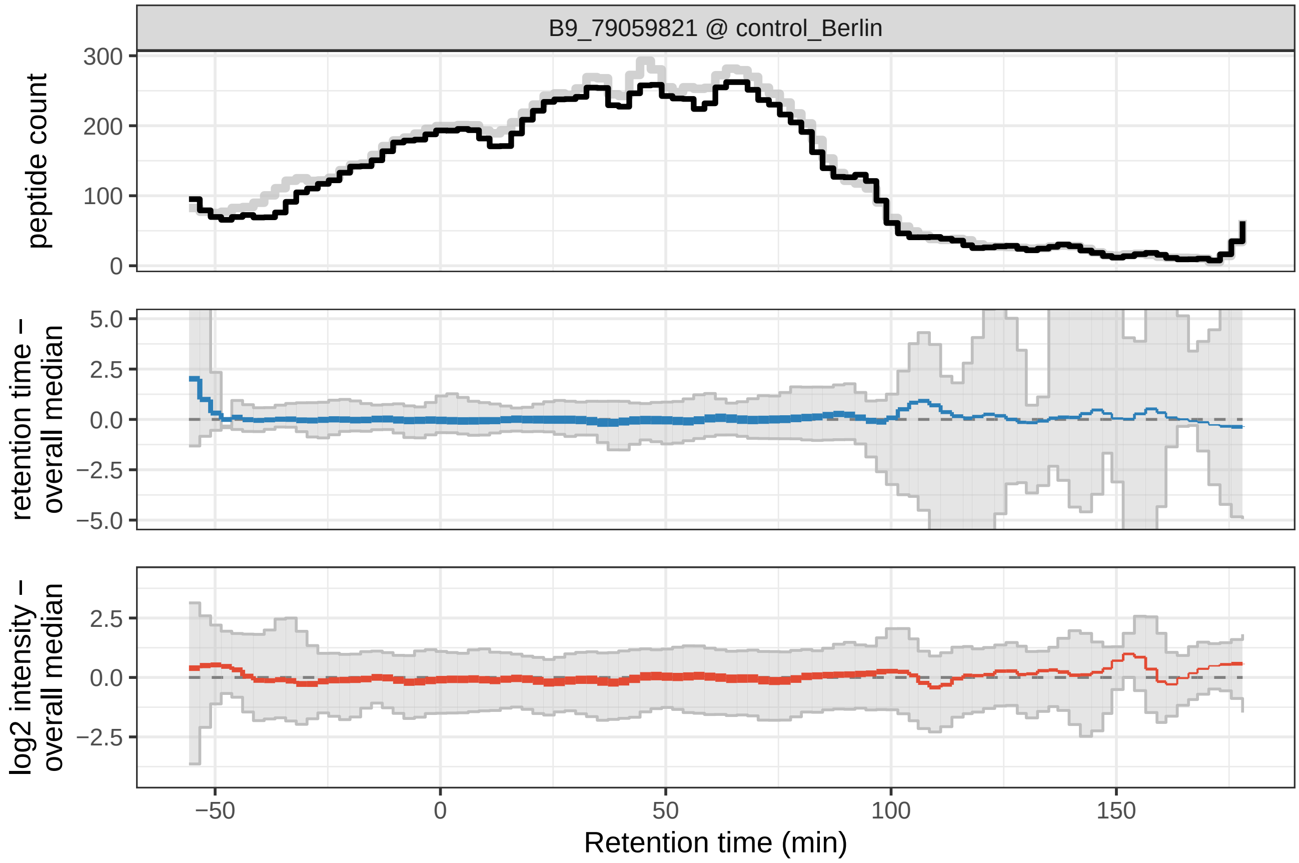
B9_79059821 @ control_Berlin
peptide count
retention time – overall median
log2 intensity – overall median
Retention time (min)

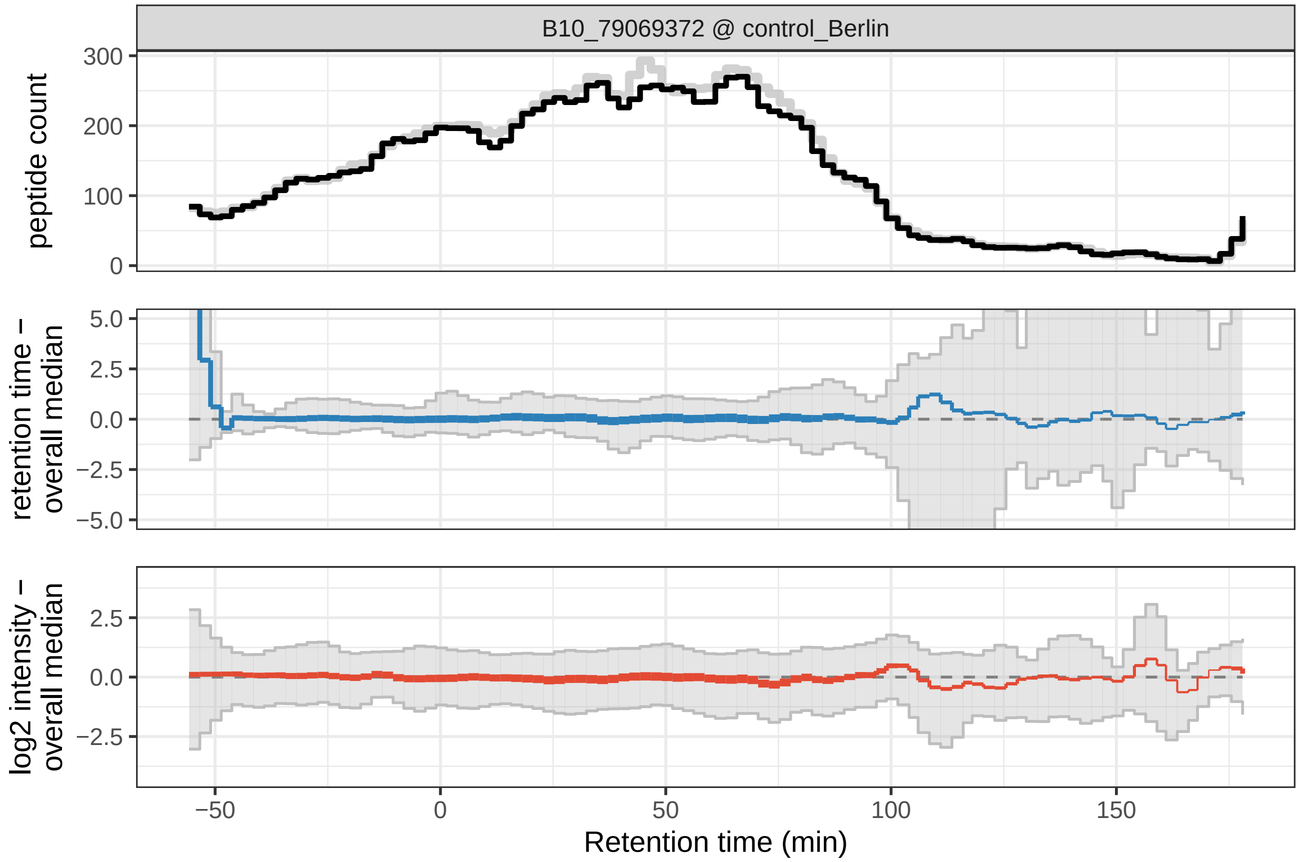

B10_79069372 @ control_Berlin
peptide count
retention time – overall median
log2 intensity – overall median
Retention time (min)

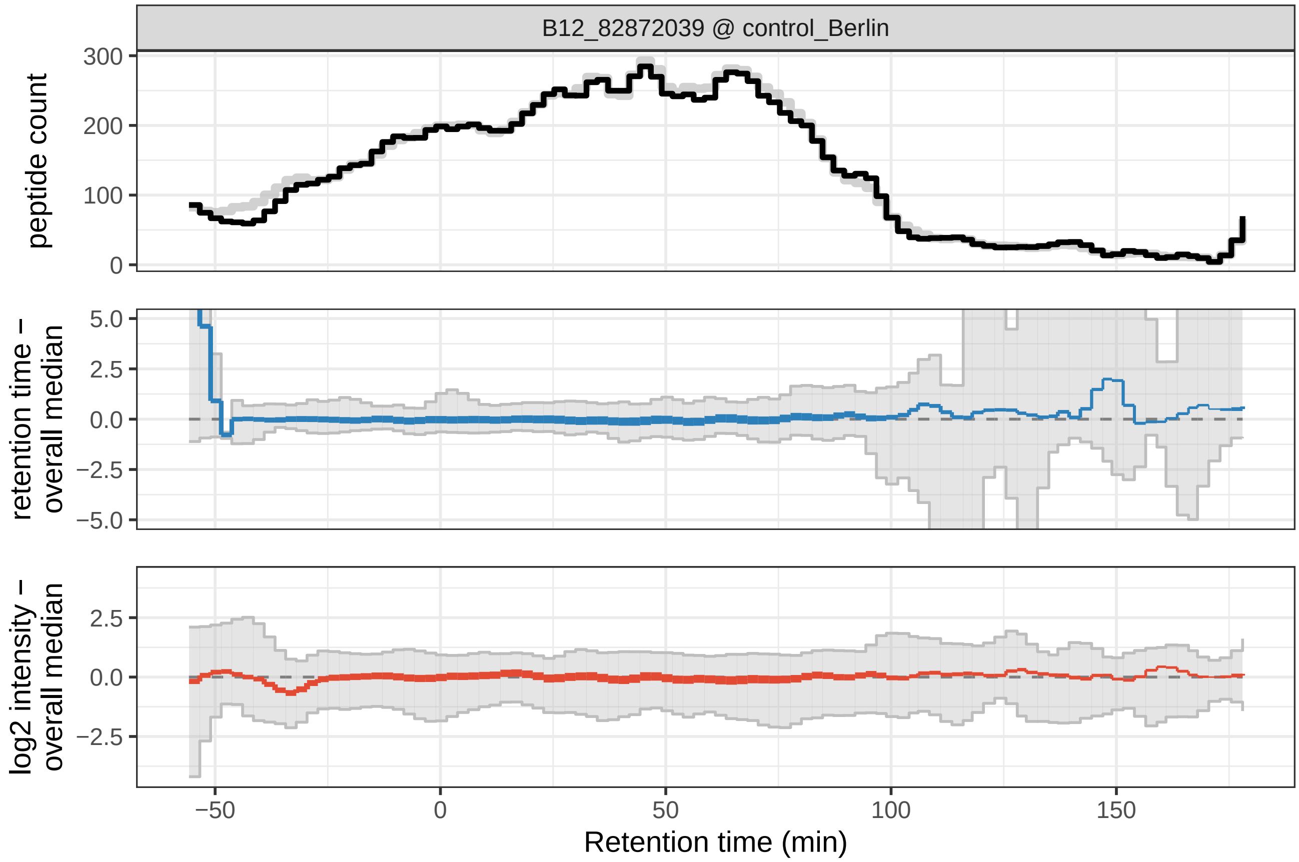

B12_82872039 @ control_Berlin
peptide count
retention time – overall median
log2 intensity – overall median
Retention time (min)

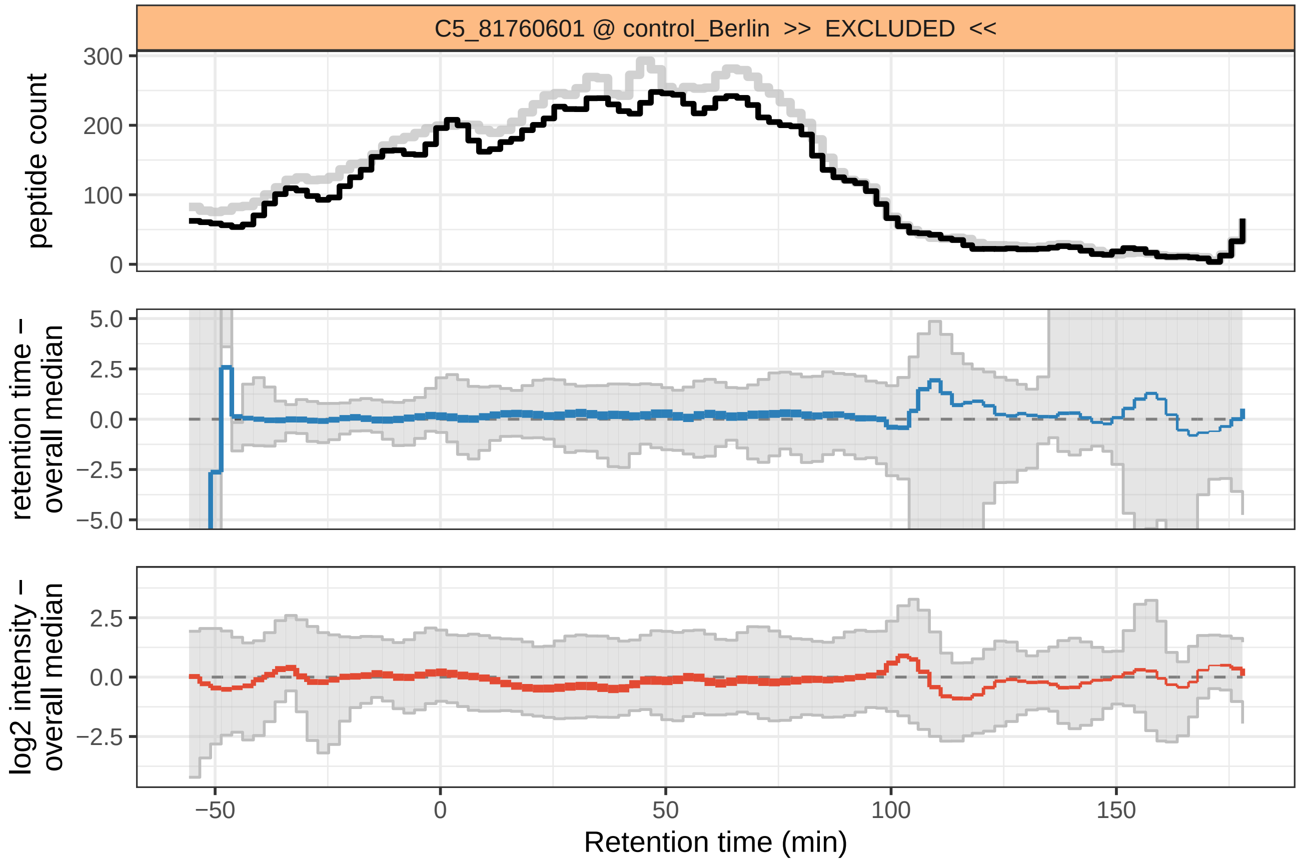

C5_81760601 @ control_Berlin >> EXCLUDED <<
peptide count
retention time – overall median
log2 intensity – overall median
Retention time (min)

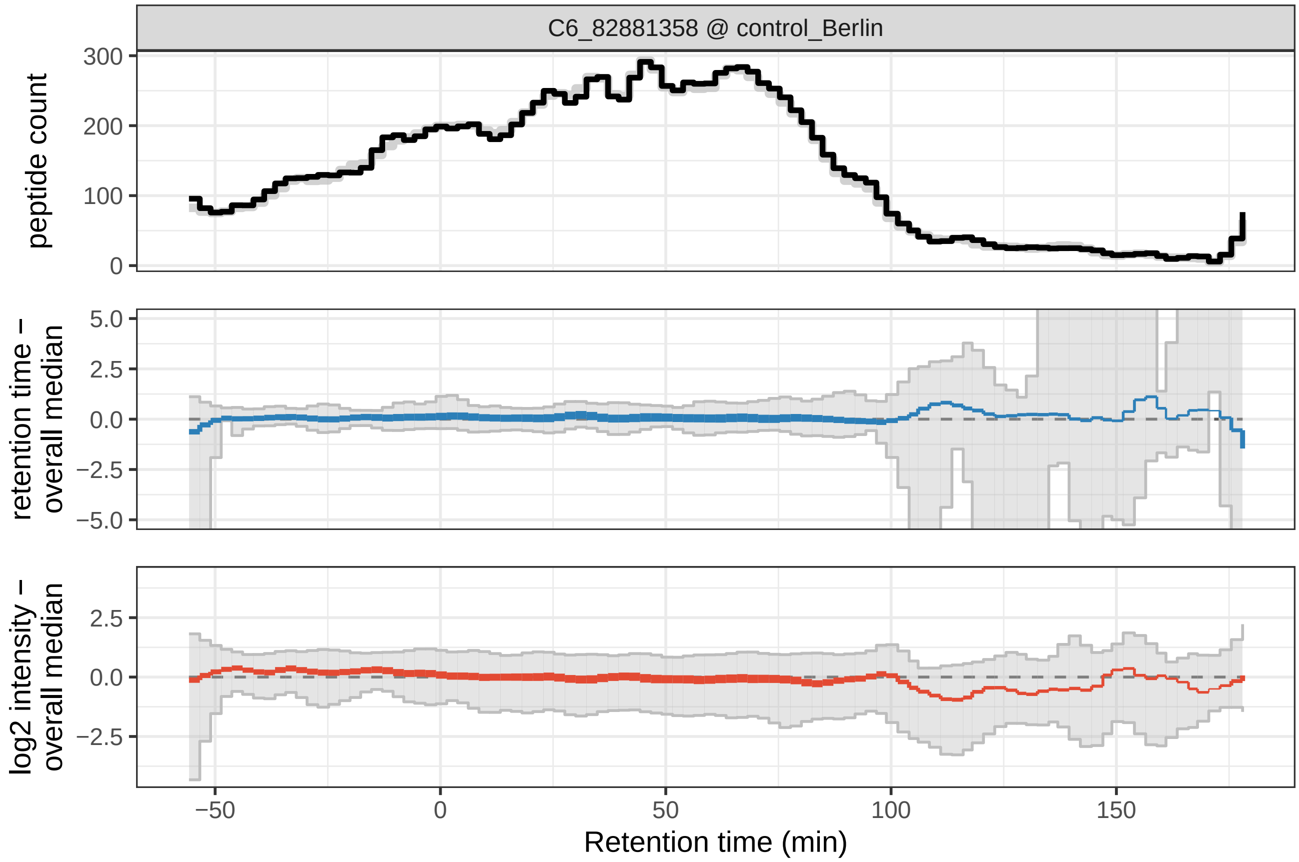

C6_82881358 @ control_Berlin
peptide count
retention time – overall median
log2 intensity – overall median
Retention time (min)

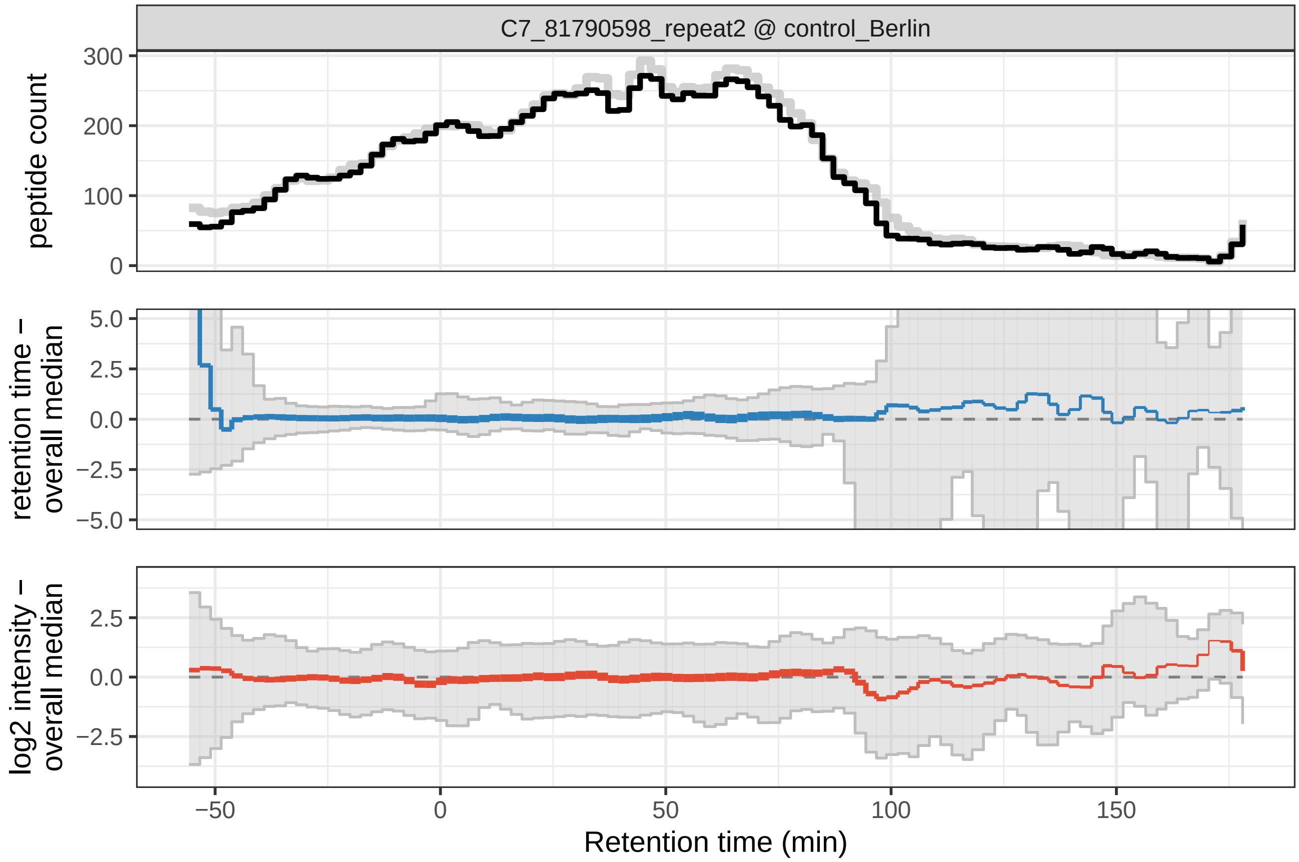
C7_81790598_repeat2 @ control_Berlin
peptide count
300
200
100
0
retention time –
overall median
5.0
2.5
0.0
−2.5
−5.0
log2 intensity –
overall median
2.5
0.0
−2.5
−50
0
50
100
150
Retention time (min)

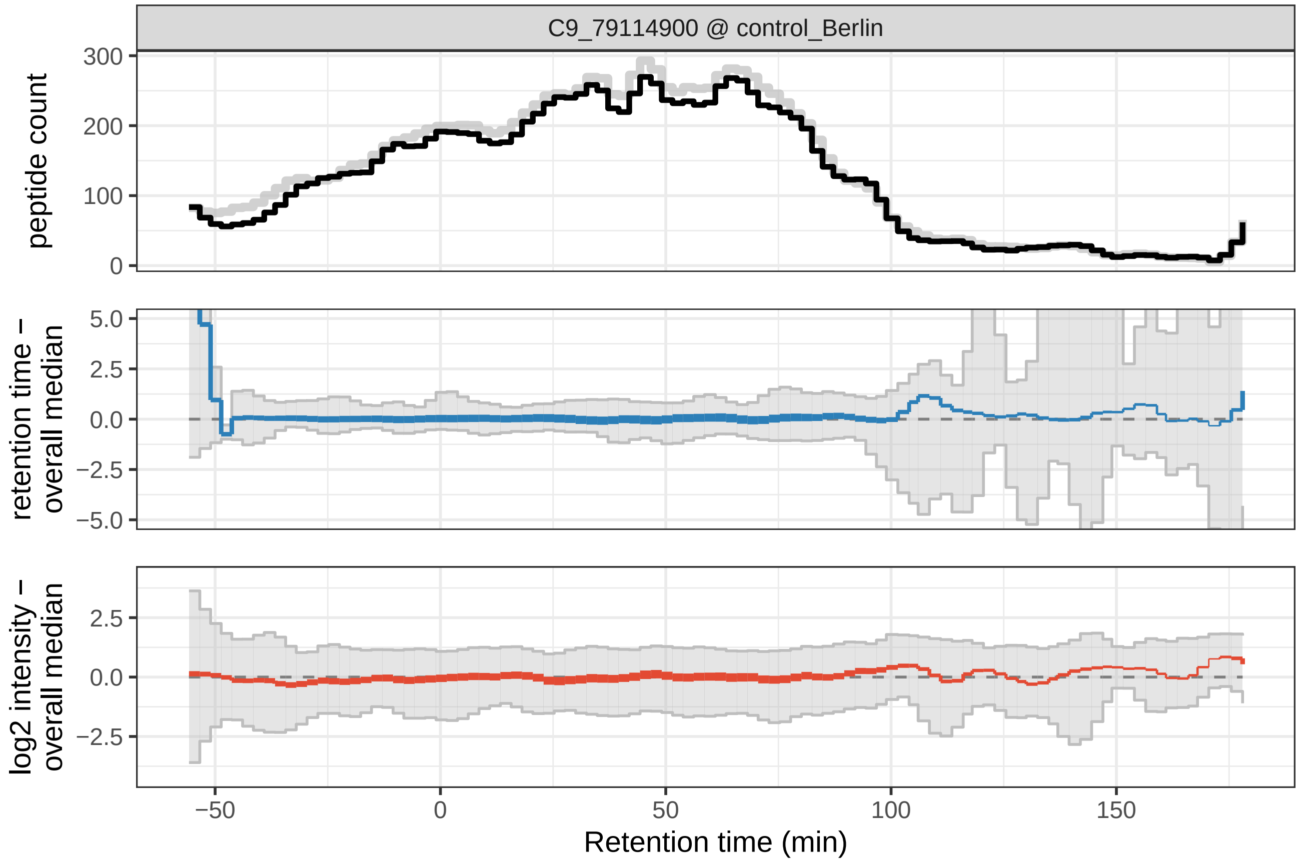
C9_79114900 @ control_Berlin
peptide count
300
200
100
0
retention time –
overall median
5.0
2.5
0.0
−2.5
−5.0
log2 intensity –
overall median
2.5
0.0
−2.5
−50
0
50
100
150
Retention time (min)

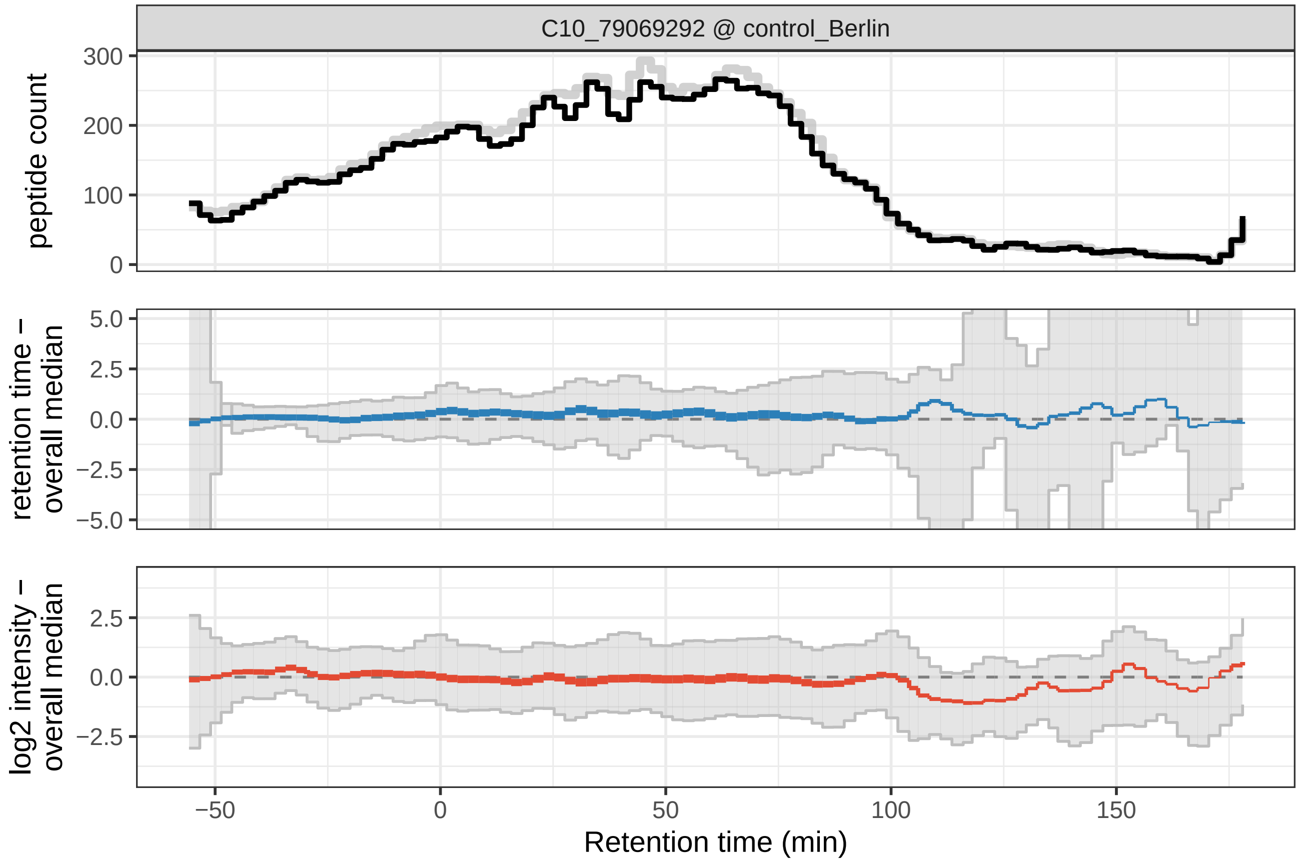
C10_79069292 @ control_Berlin
peptide count
300
200
100
0
retention time –
overall median
5.0
2.5
0.0
−2.5
−5.0
log2 intensity –
overall median
2.5
0.0
−2.5
−50
0
50
100
150
Retention time (min)

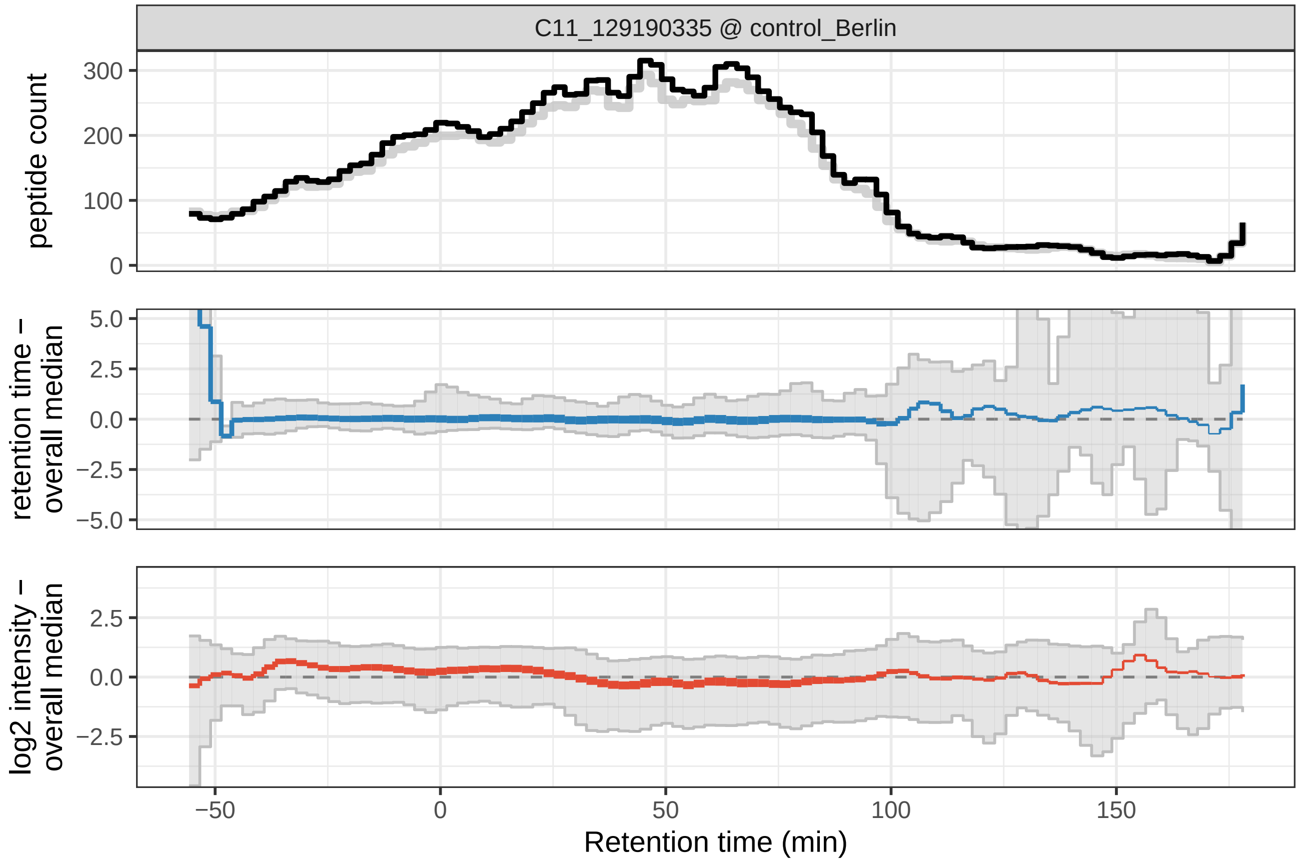
C11_129190335 @ control_Berlin
peptide count
300
200
100
0
retention time –
overall median
5.0
2.5
0.0
−2.5
−5.0
log2 intensity –
overall median
2.5
0.0
−2.5
−50
0
50
100
150
Retention time (min)

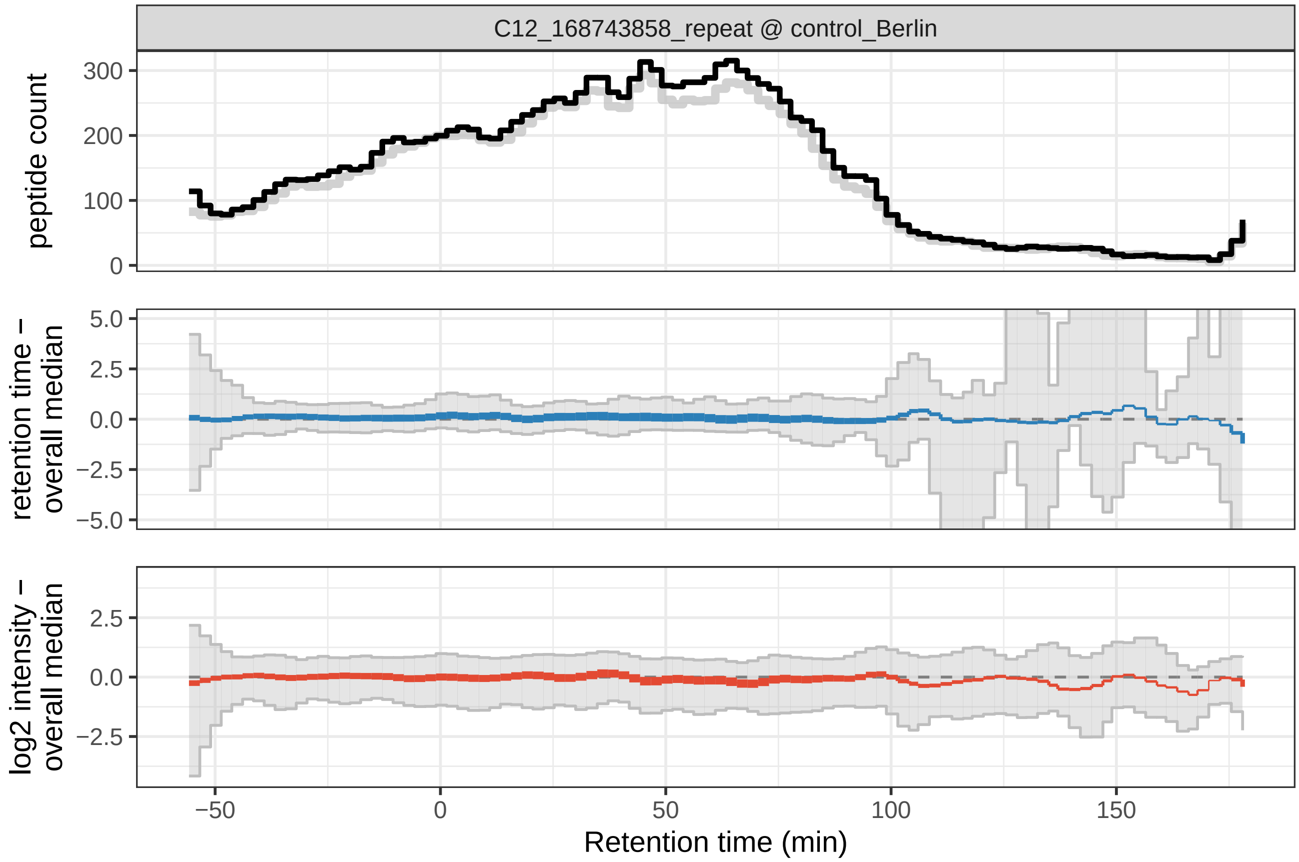
C12_168743858_repeat @ control_Berlin
peptide count
retention time – overall median
log2 intensity – overall median
Retention time (min)

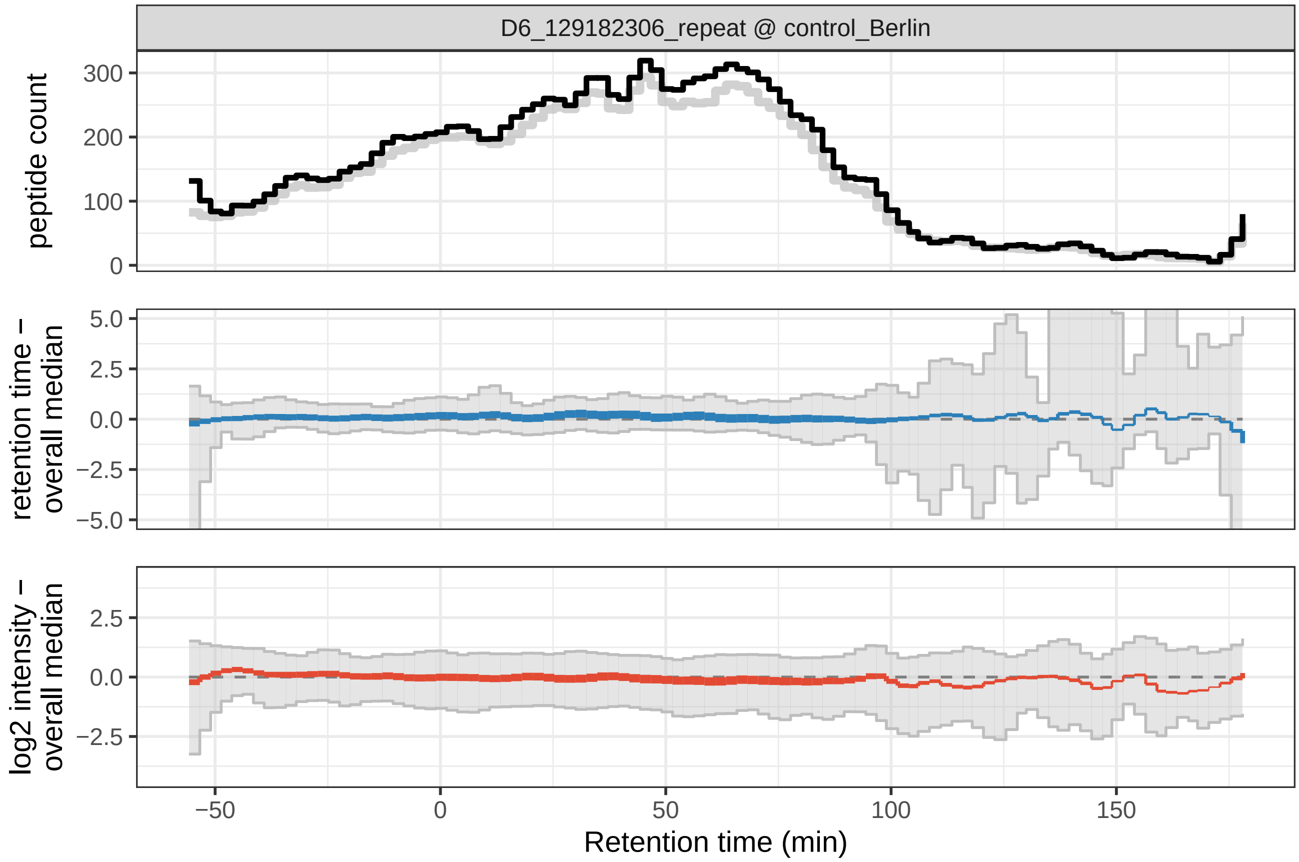
D6_129182306_repeat @ control_Berlin
peptide count
retention time – overall median
log2 intensity – overall median
Retention time (min)

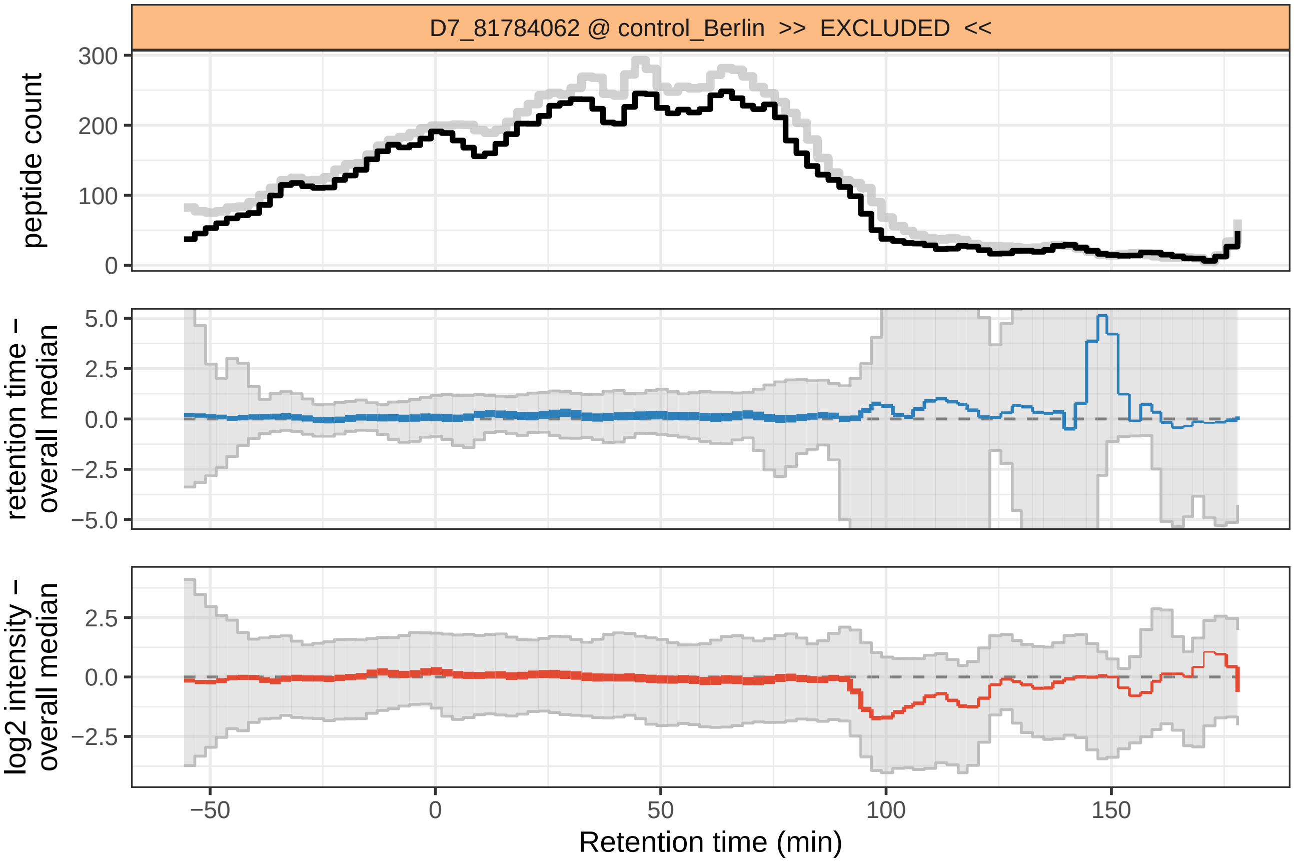

D7_81784062 @ control_Berlin >> EXCLUDED <<
peptide count
retention time – overall median
log2 intensity – overall median
Retention time (min)

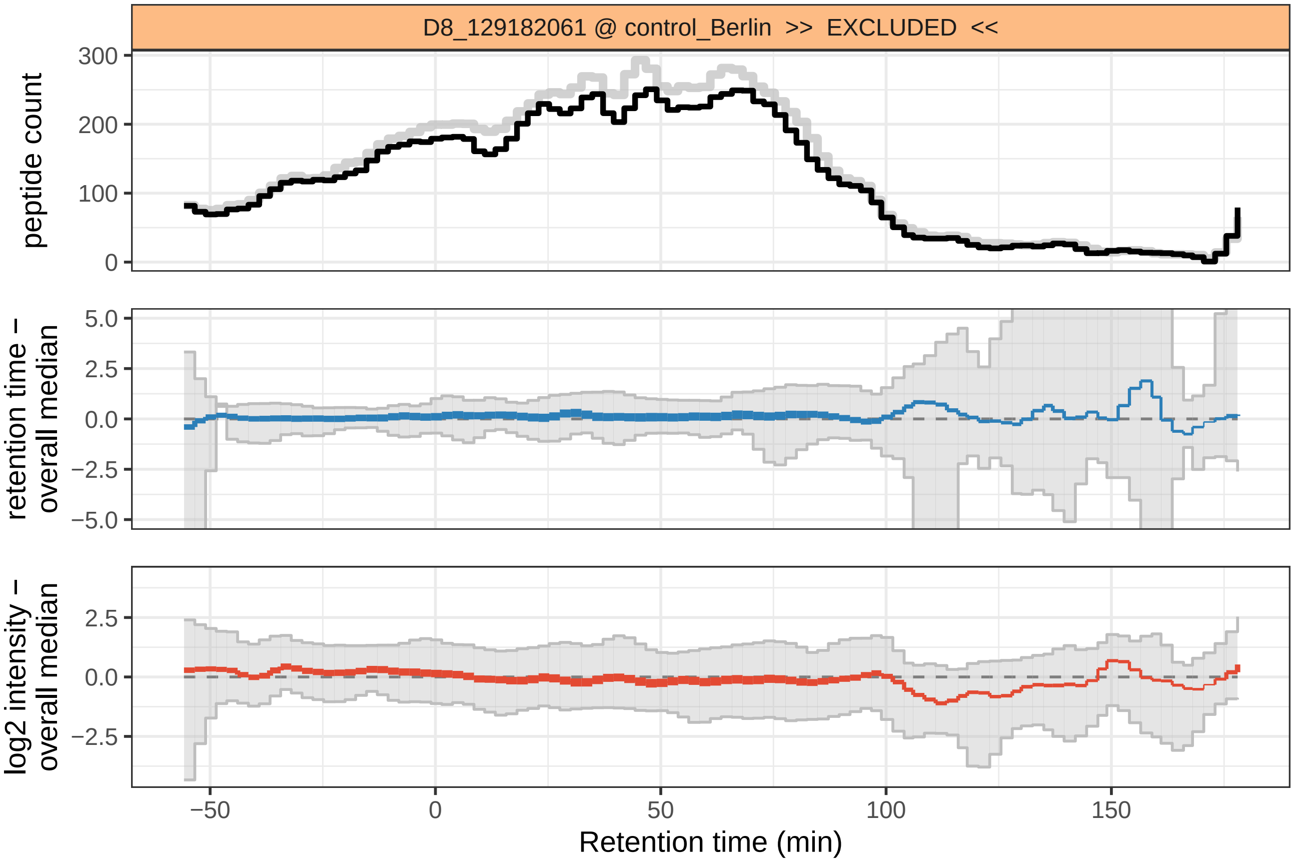

D8_129182061 @ control_Berlin >> EXCLUDED <<
peptide count
retention time – overall median
log2 intensity – overall median
Retention time (min)

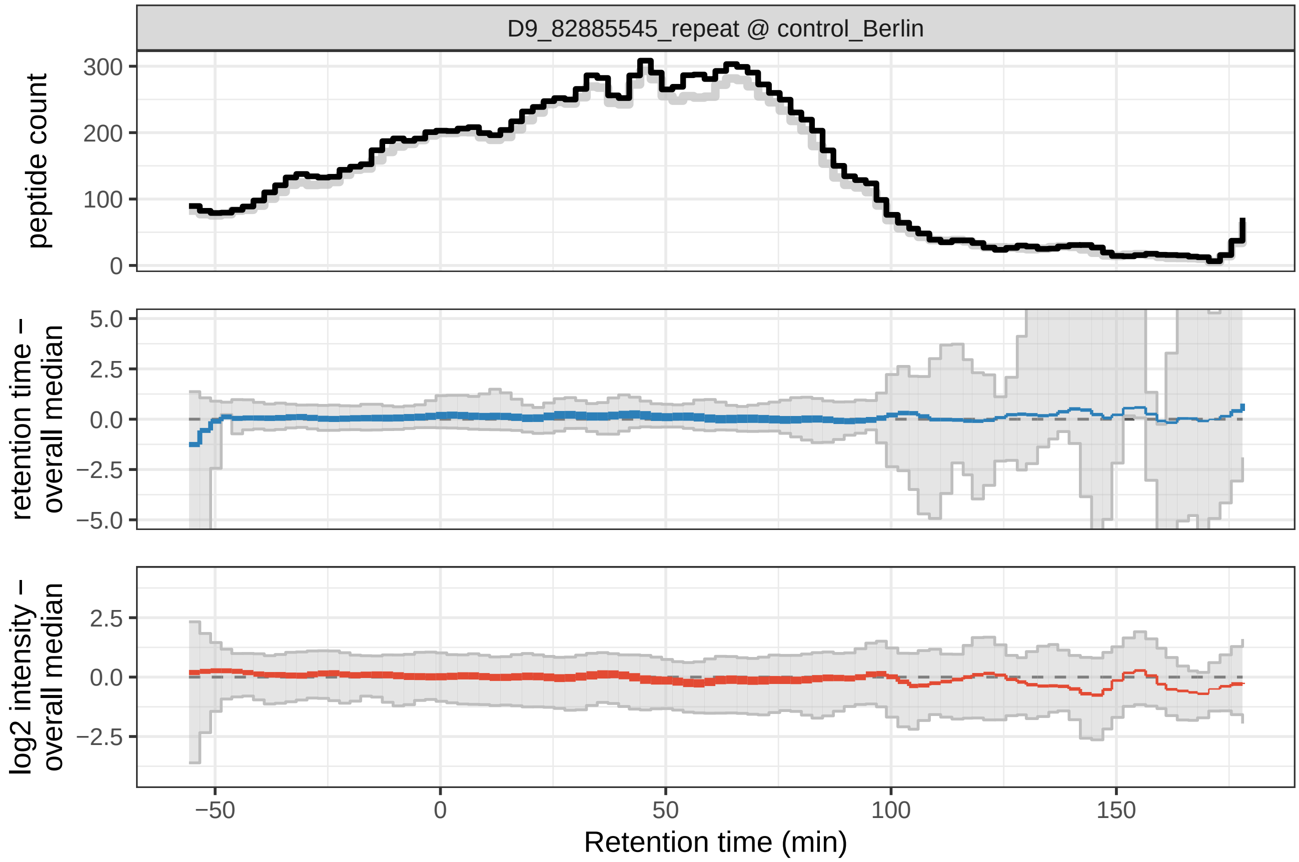
D9_82885545_repeat @ control_Berlin
peptide count
300
200
100
0
retention time –
overall median
5.0
2.5
0.0
−2.5
−5.0
log2 intensity –
overall median
2.5
0.0
−2.5
−50
0
50
100
150
Retention time (min)

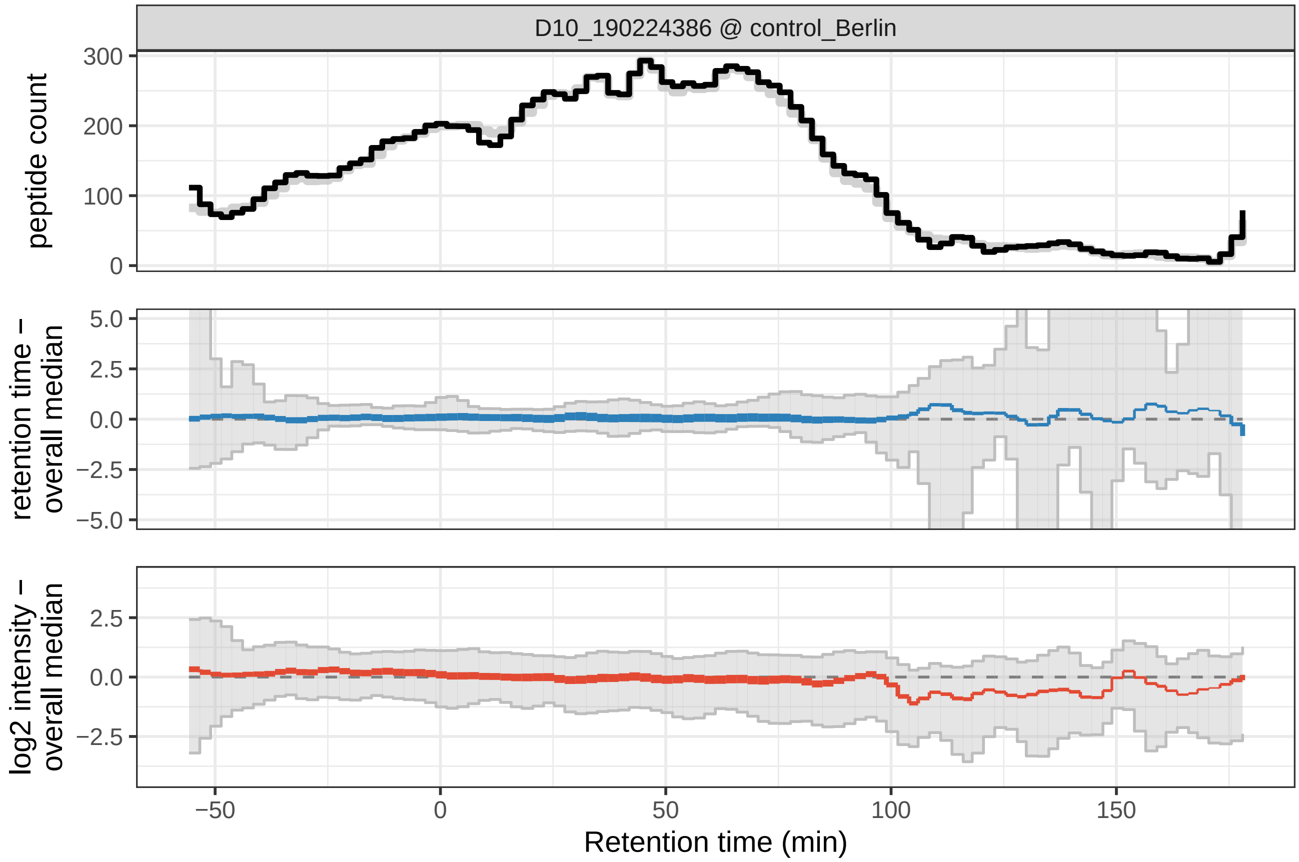
D10_190224386 @ control_Berlin
peptide count
300
200
100
0
retention time –
overall median
5.0
2.5
0.0
−2.5
−5.0
log2 intensity –
overall median
2.5
0.0
−2.5
−50
0
50
100
150
Retention time (min)

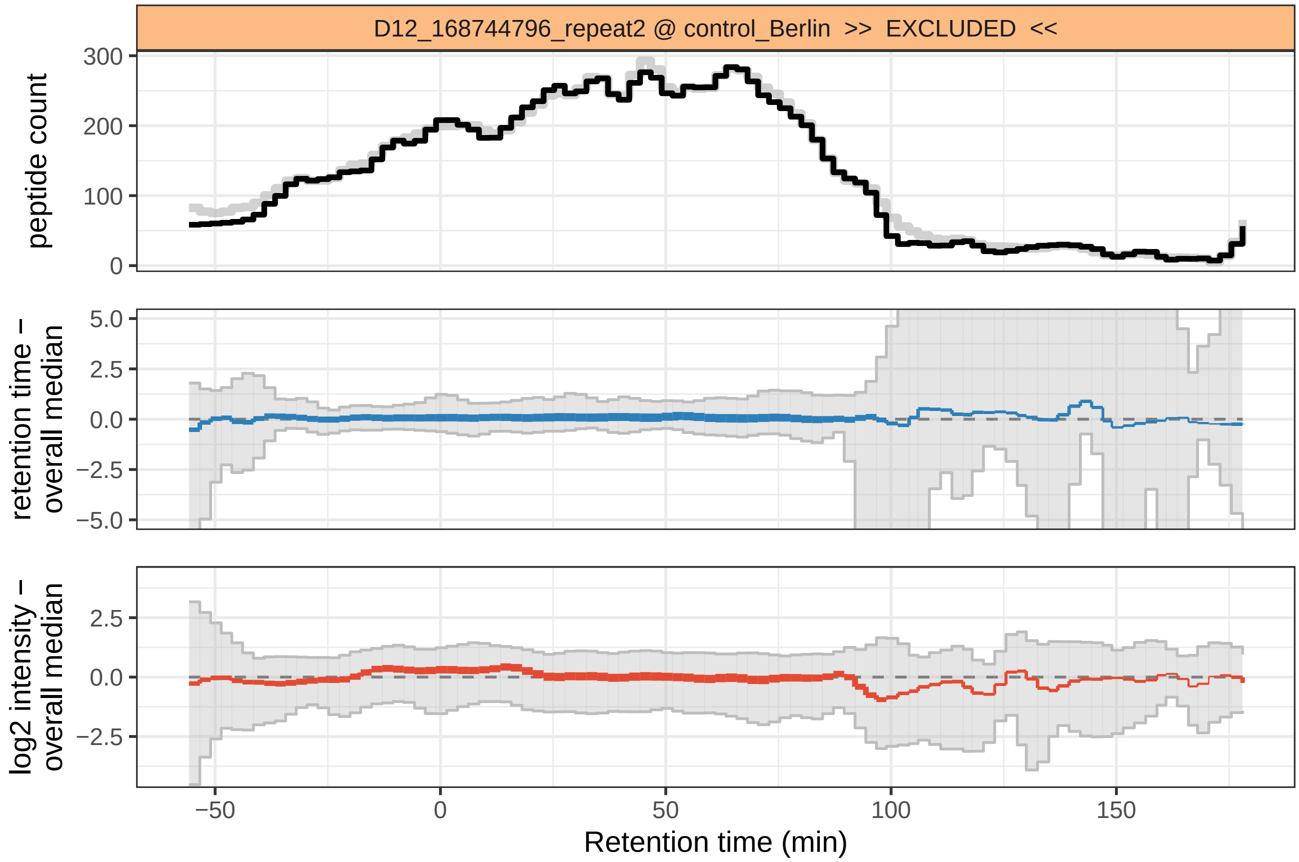
D12_168744796_repeat2 @ control_Berlin >> EXCLUDED <<
peptide count
retention time – overall median
log2 intensity – overall median
Retention time (min)

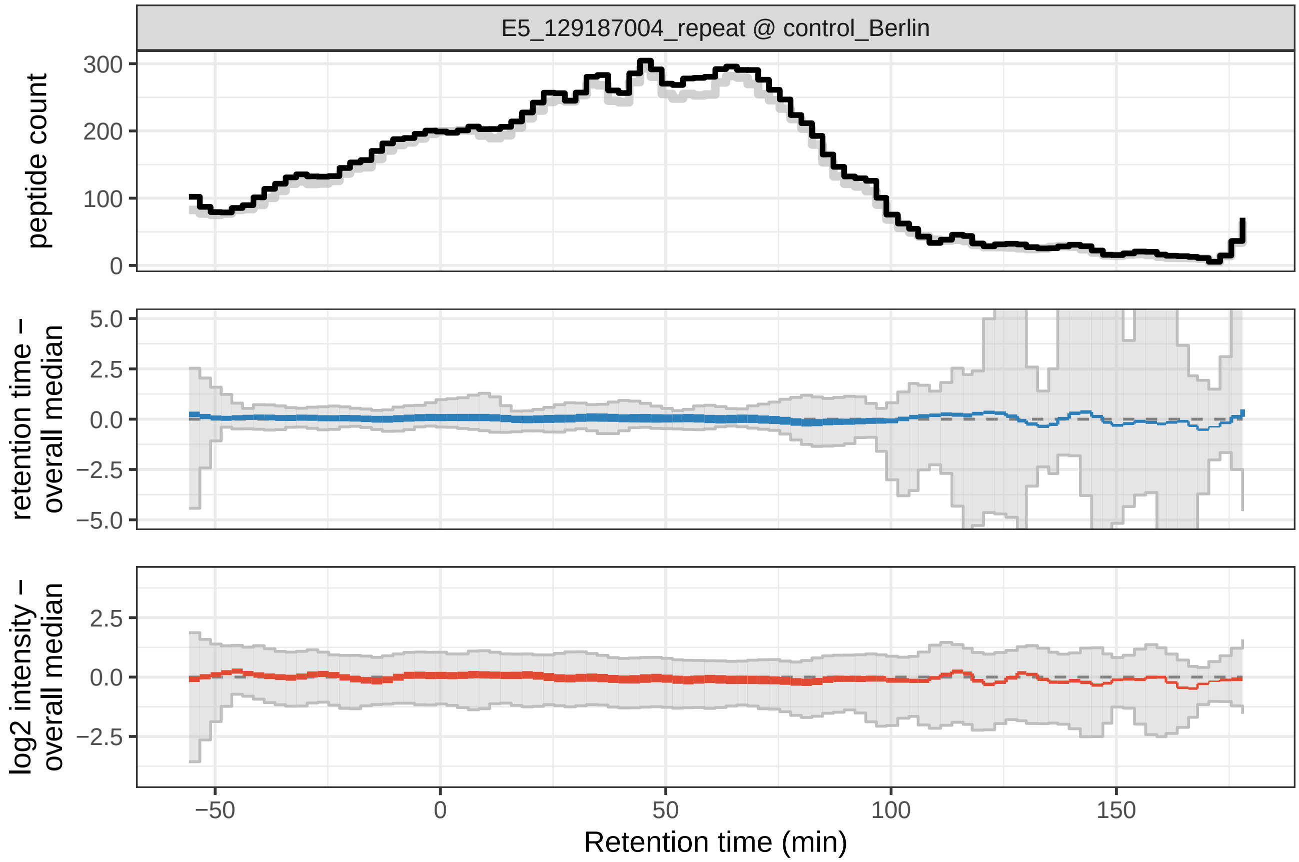
E5_129187004_repeat @ control_Berlin
peptide count
retention time – overall median
log2 intensity – overall median
Retention time (min)

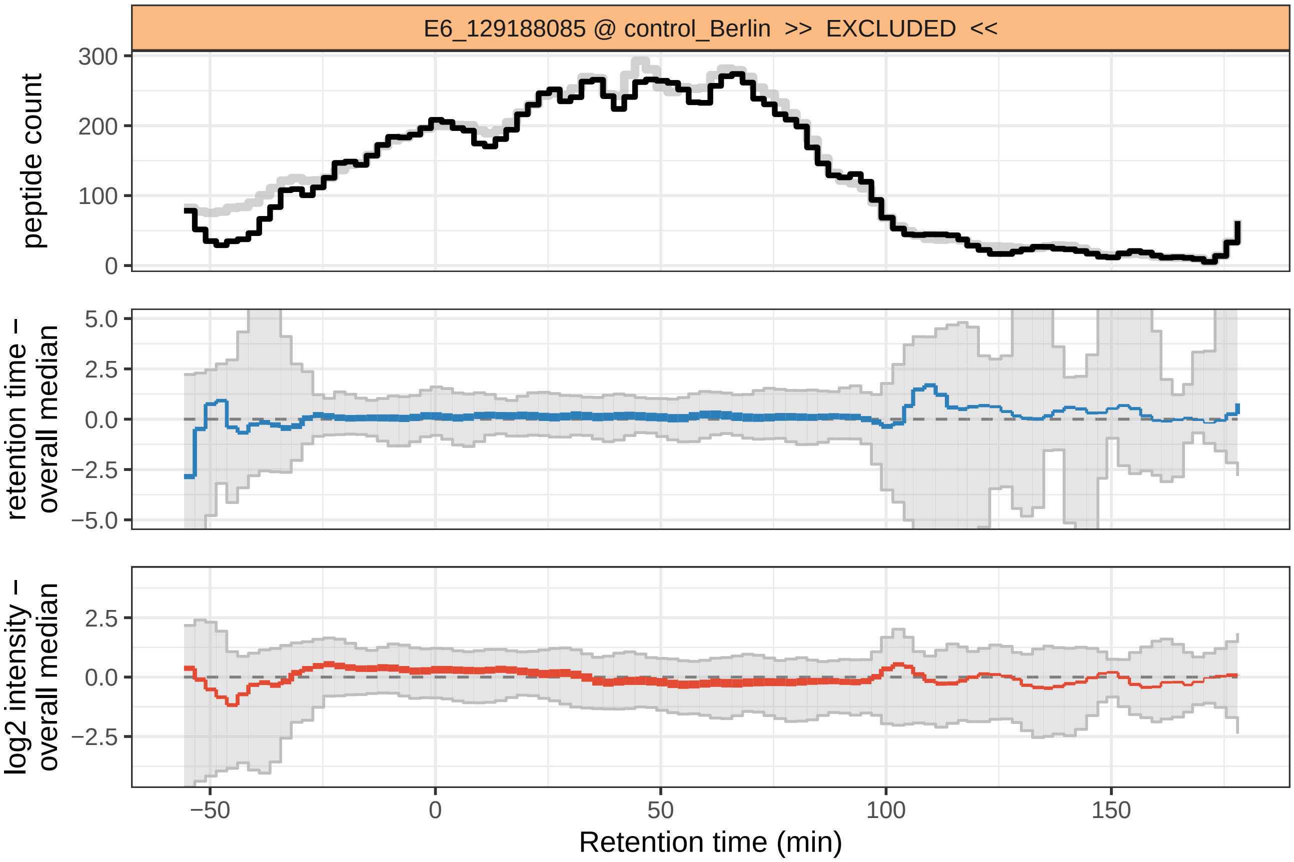
E6_129188085 @ control_Berlin >> EXCLUDED <<
peptide count
retention time – overall median
log2 intensity – overall median
Retention time (min)

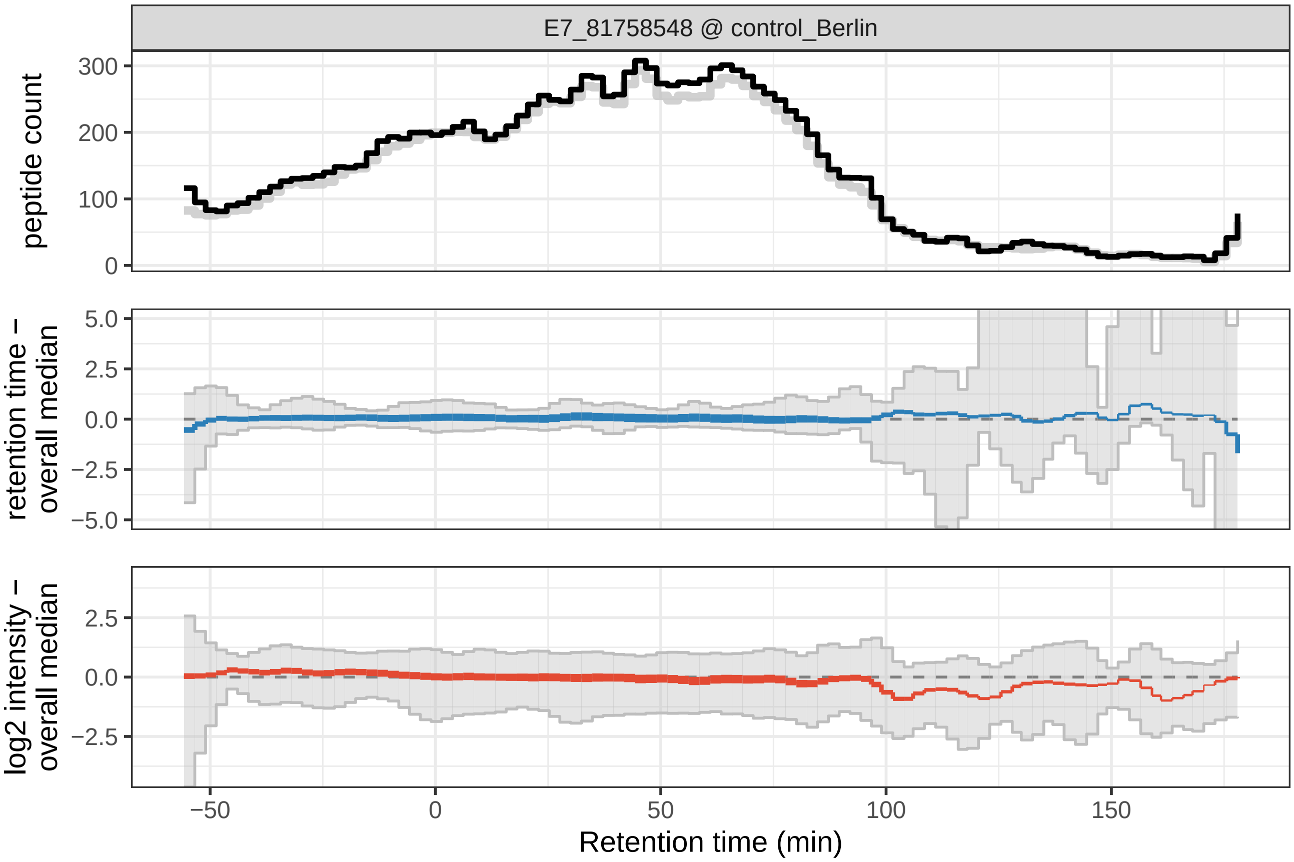
E7_81758548 @ control_Berlin
peptide count
retention time – overall median
log2 intensity – overall median
Retention time (min)

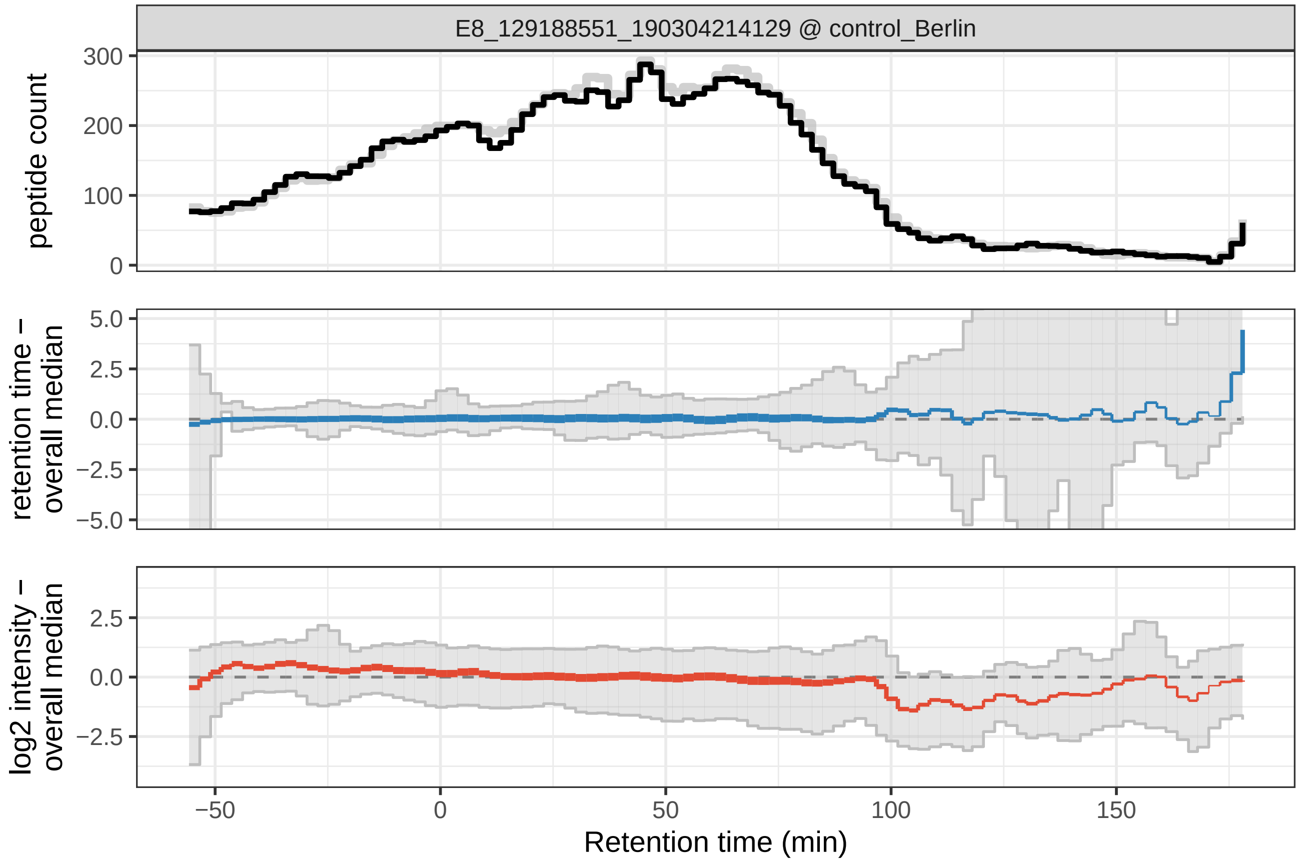

E8_129188551_190304214129 @ control_Berlin
peptide count
300
200
100
0
retention time –
overall median
5.0
2.5
0.0
−2.5
−5.0
log2 intensity –
overall median
2.5
0.0
−2.5
−50
0
50
100
150
Retention time (min)

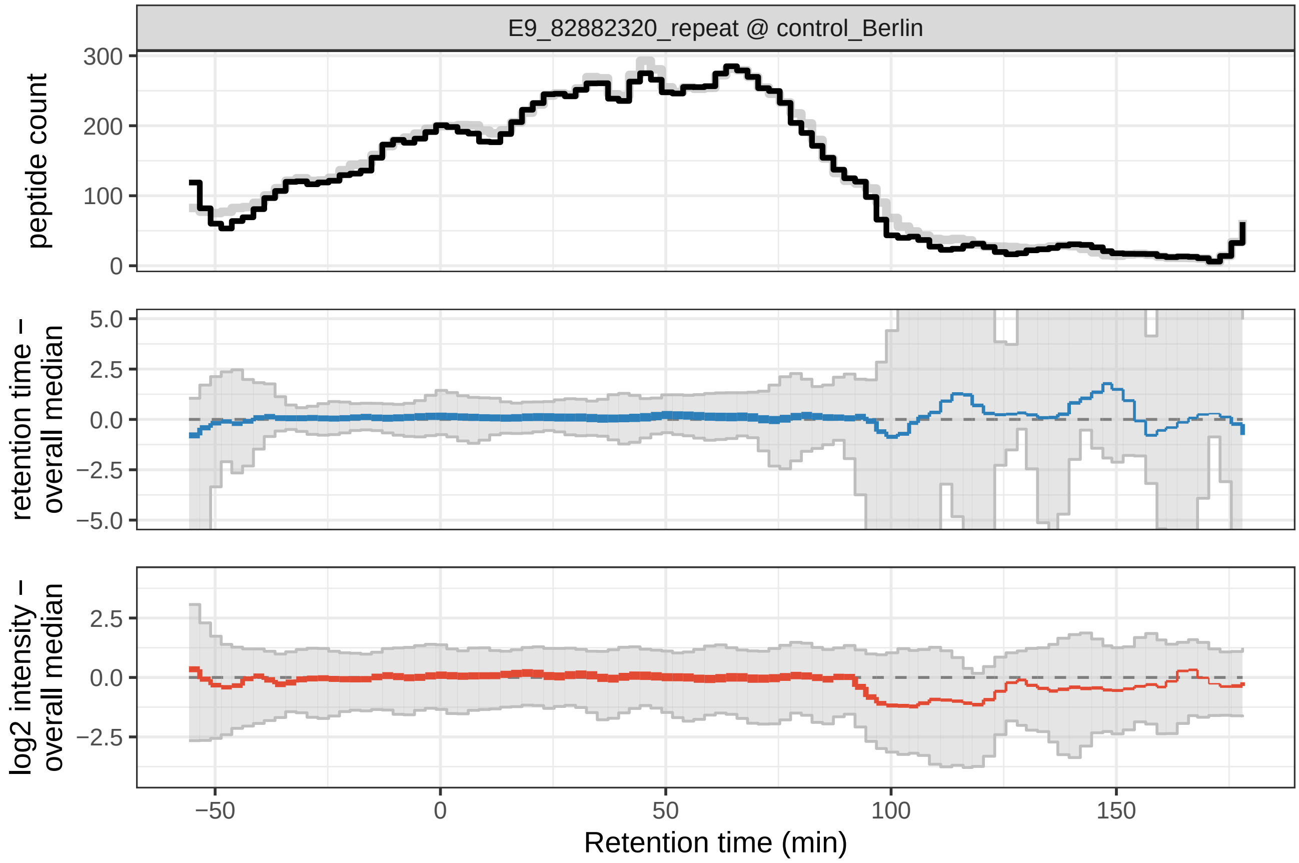

E9_82882320_repeat @ control_Berlin
peptide count
300
200
100
0
retention time –
overall median
5.0
2.5
0.0
−2.5
−5.0
log2 intensity –
overall median
2.5
0.0
−2.5
−50
0
50
100
150
Retention time (min)

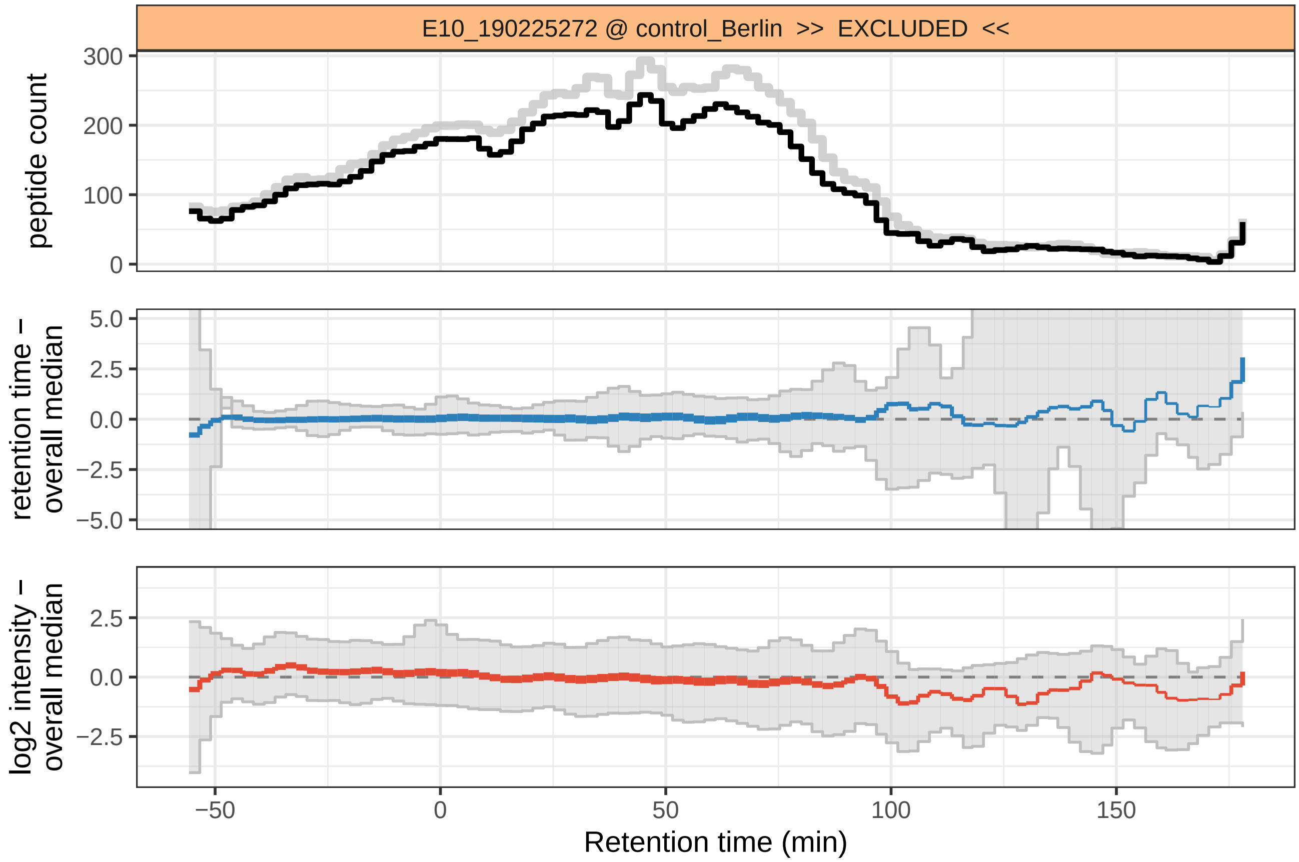

E10_190225272 @ control_Berlin >> EXCLUDED <<
peptide count
300
200
100
0
retention time –
overall median
5.0
2.5
0.0
−2.5
−5.0
log2 intensity –
overall median
2.5
0.0
−2.5
−50
0
50
100
150
Retention time (min)

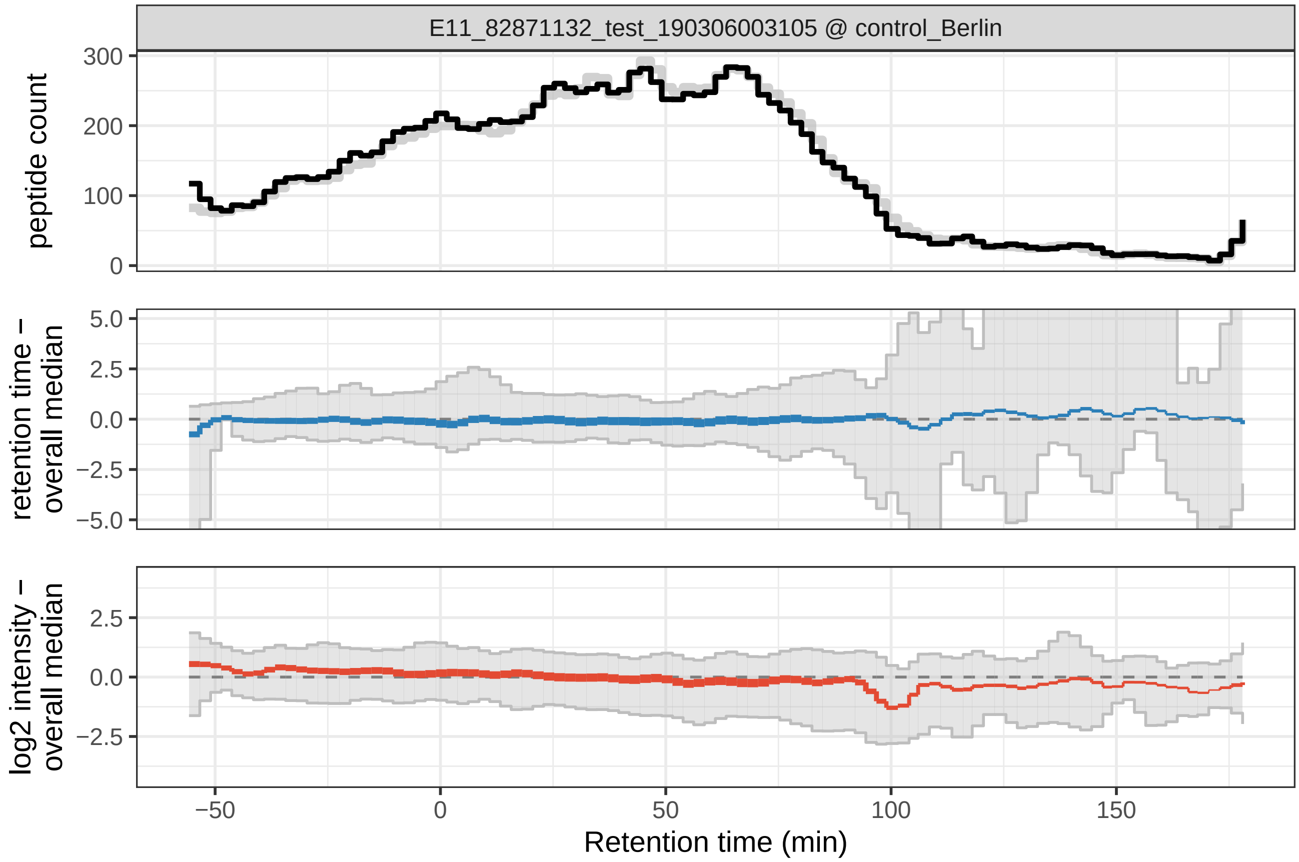

E11_82871132_test_190306003105 @ control_Berlin
peptide count
300
200
100
0
retention time –
overall median
5.0
2.5
0.0
−2.5
−5.0
log2 intensity –
overall median
2.5
0.0
−2.5
−50
0
50
100
150
Retention time (min)

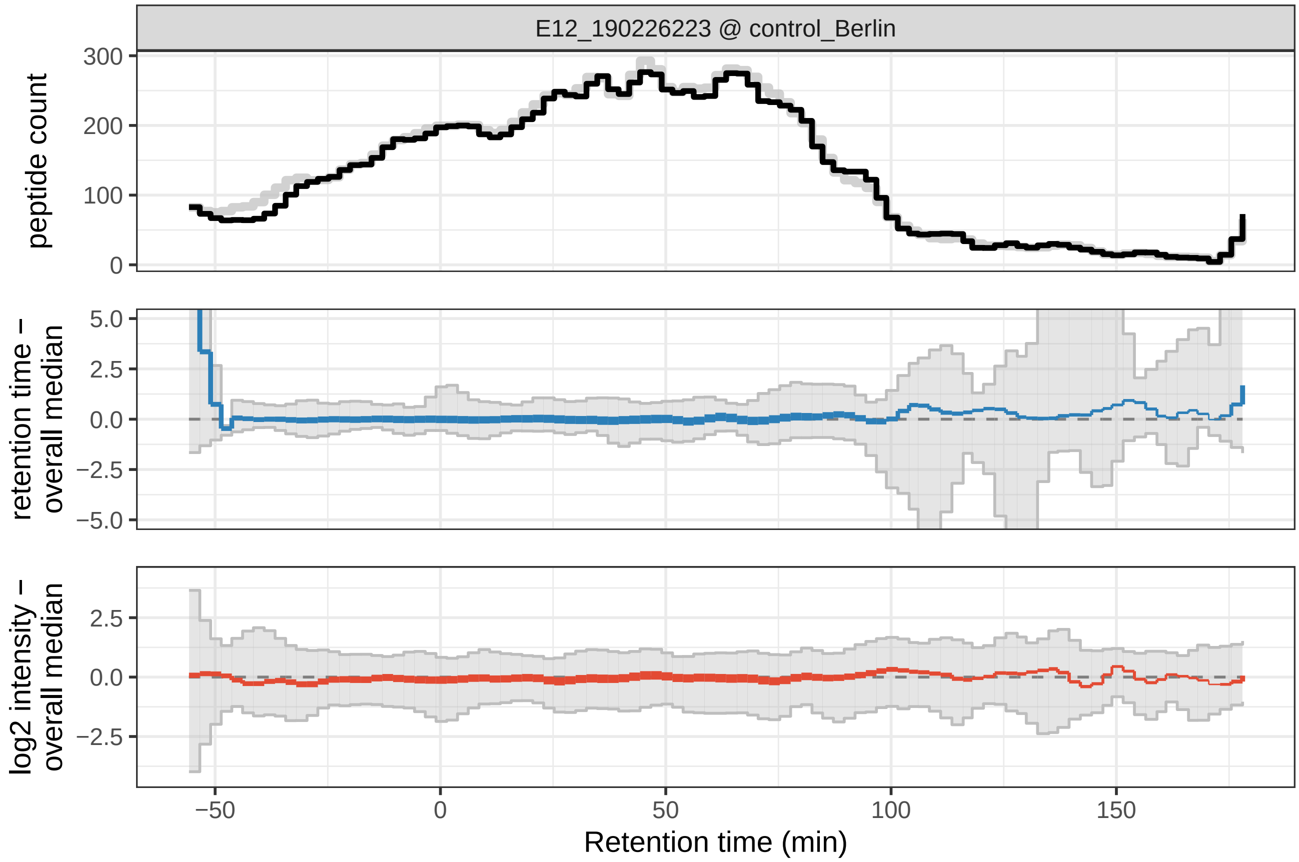
E12_190226223 @ control_Berlin
peptide count
retention time – overall median
log2 intensity – overall median
Retention time (min)

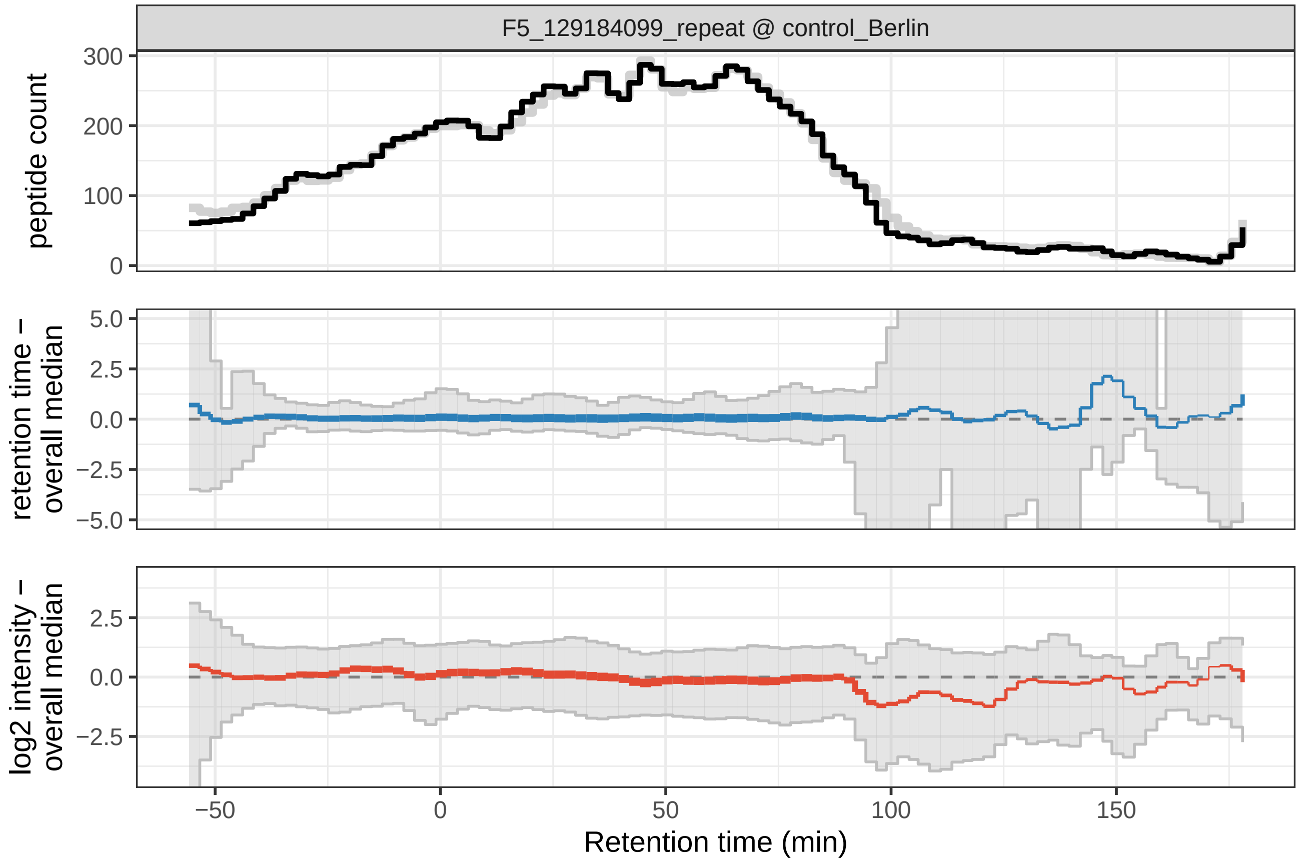
F5_129184099_repeat @ control_Berlin
peptide count
retention time – overall median
log2 intensity – overall median
Retention time (min)

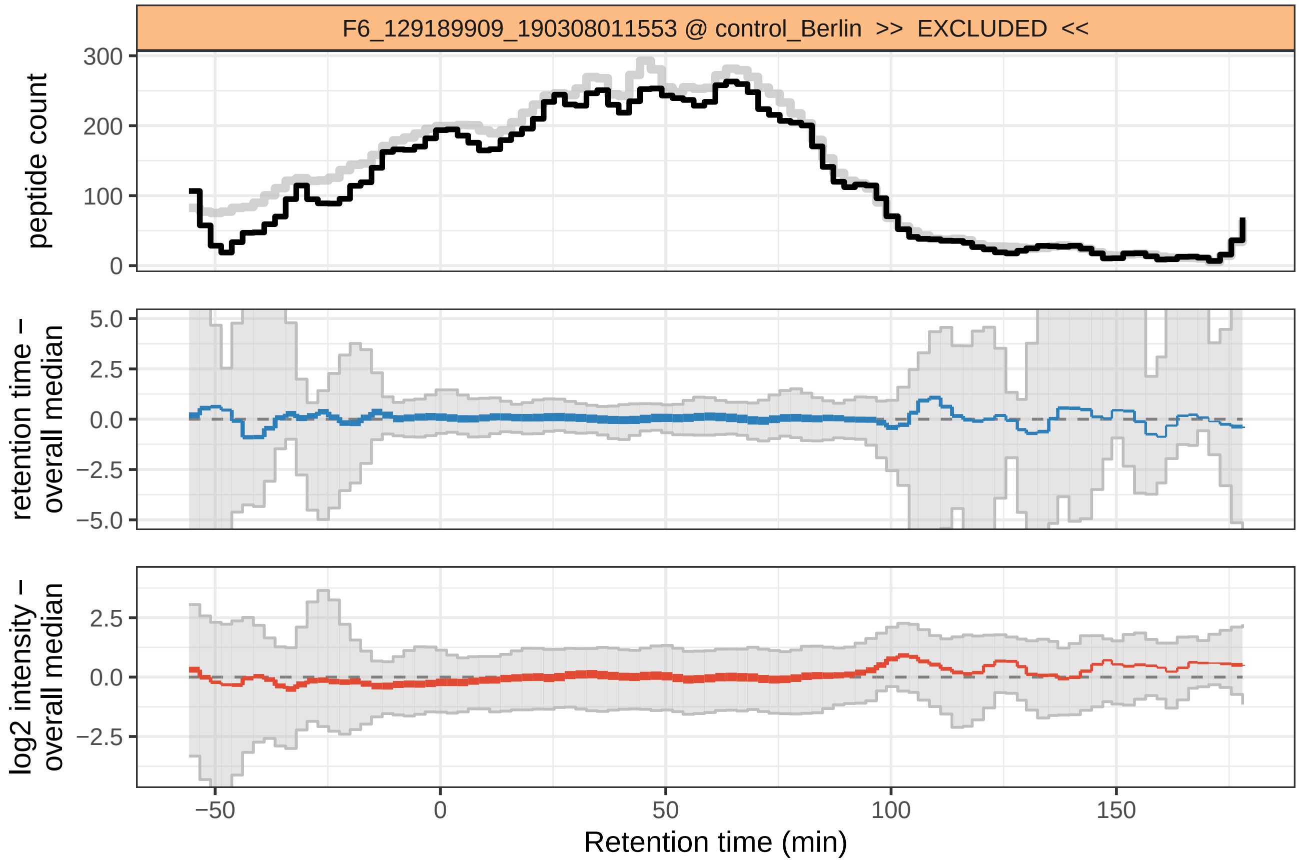

F6_129189909_190308011553 @ control_Berlin >> EXCLUDED <<
peptide count
retention time – overall median
log2 intensity – overall median
Retention time (min)

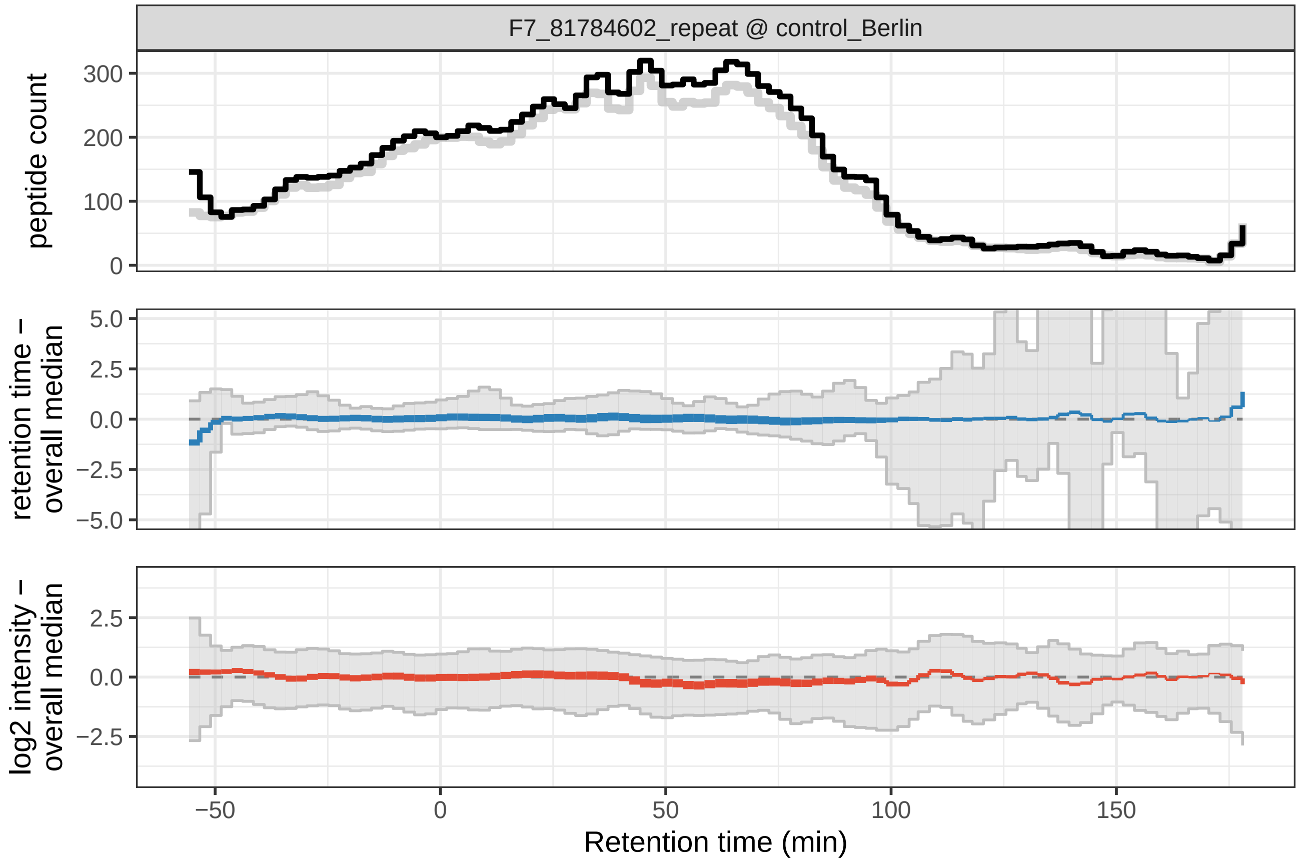

F7_81784602_repeat @ control_Berlin
peptide count
retention time – overall median
log2 intensity – overall median
Retention time (min)

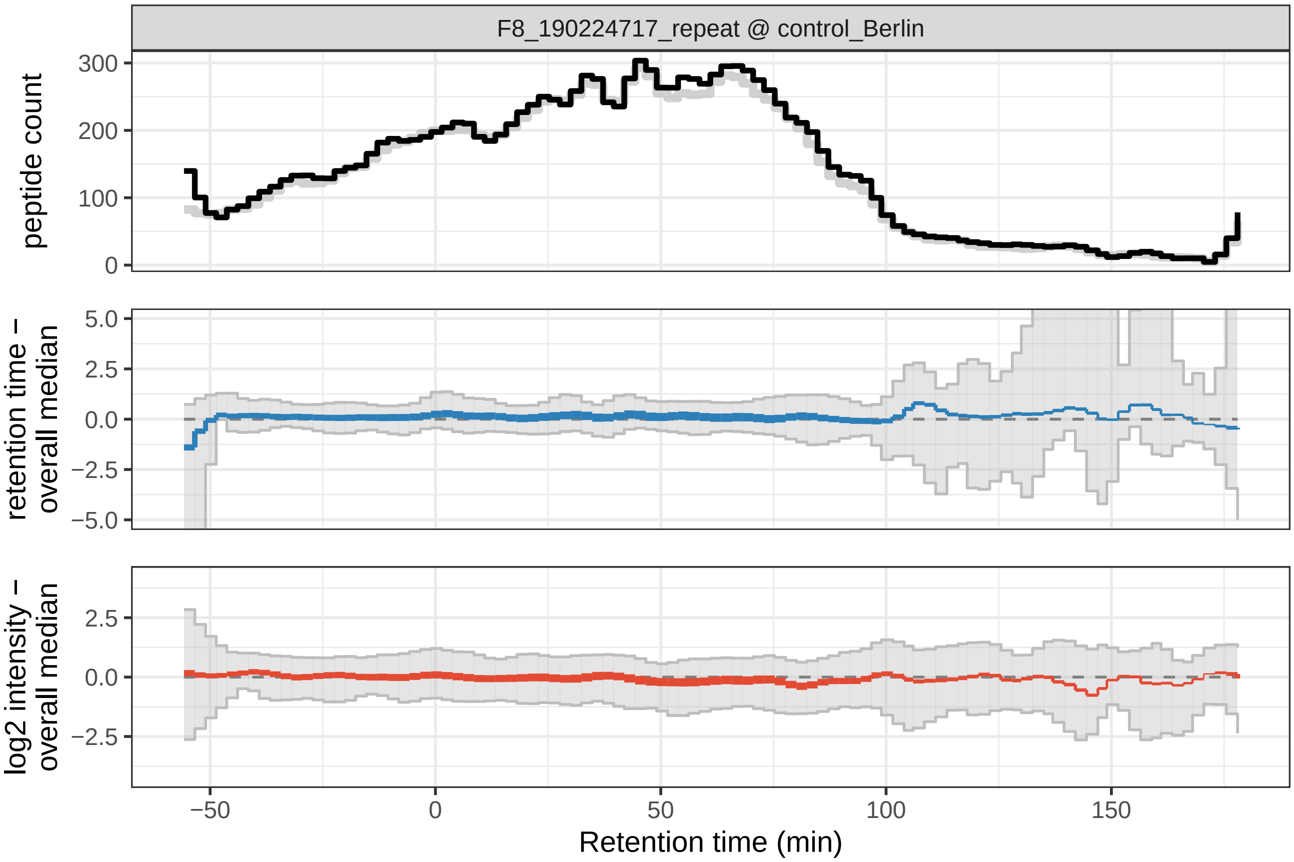
F8_190224717_repeat @ control_Berlin
peptide count
300
200
100
0
retention time –
overall median
5.0
2.5
0.0
−2.5
−5.0
log2 intensity –
overall median
2.5
0.0
−2.5
−50
0
50
100
150
Retention time (min)

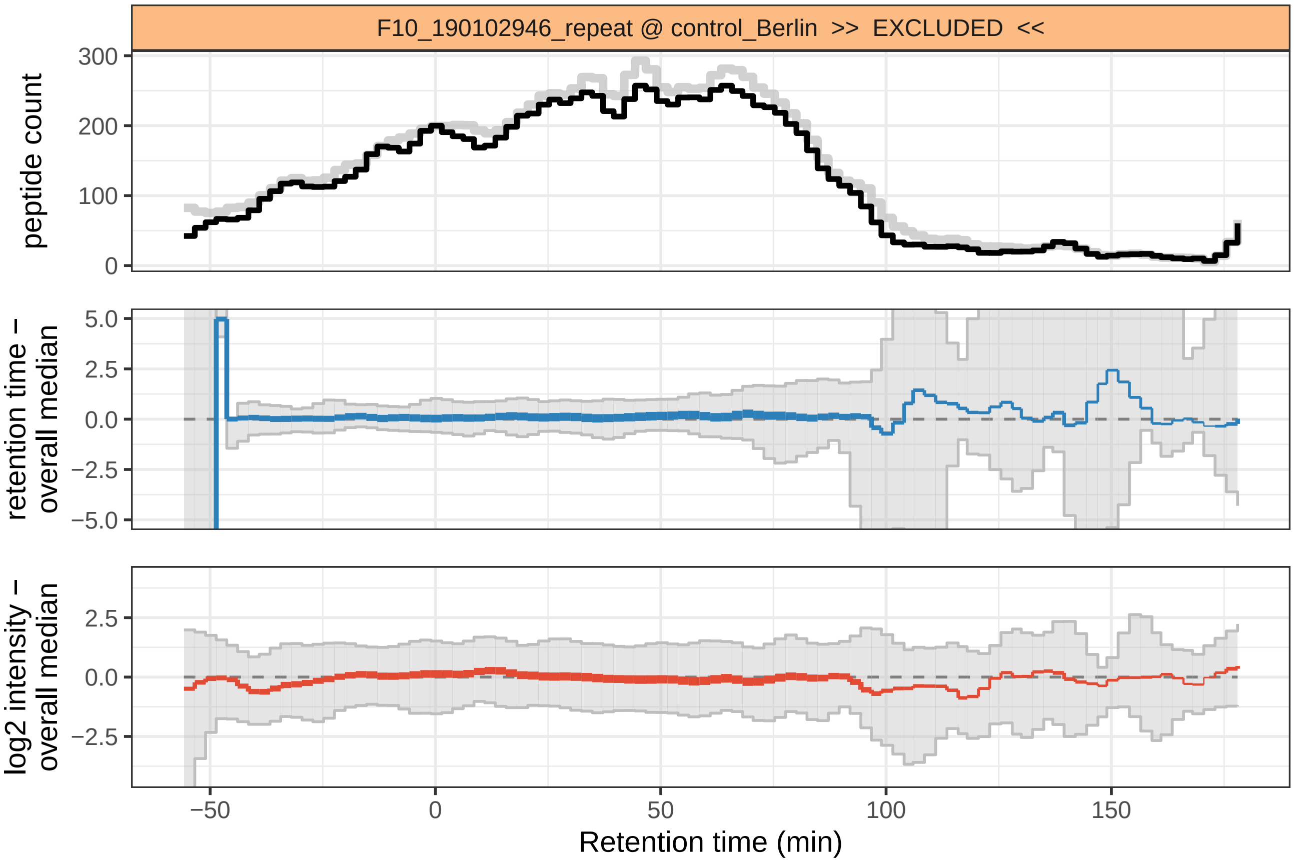
F10_190102946_repeat @ control_Berlin >> EXCLUDED <<
peptide count
300
200
100
0
retention time –
overall median
5.0
2.5
0.0
−2.5
−5.0
log2 intensity –
overall median
2.5
0.0
−2.5
−50
0
50
100
150
Retention time (min)

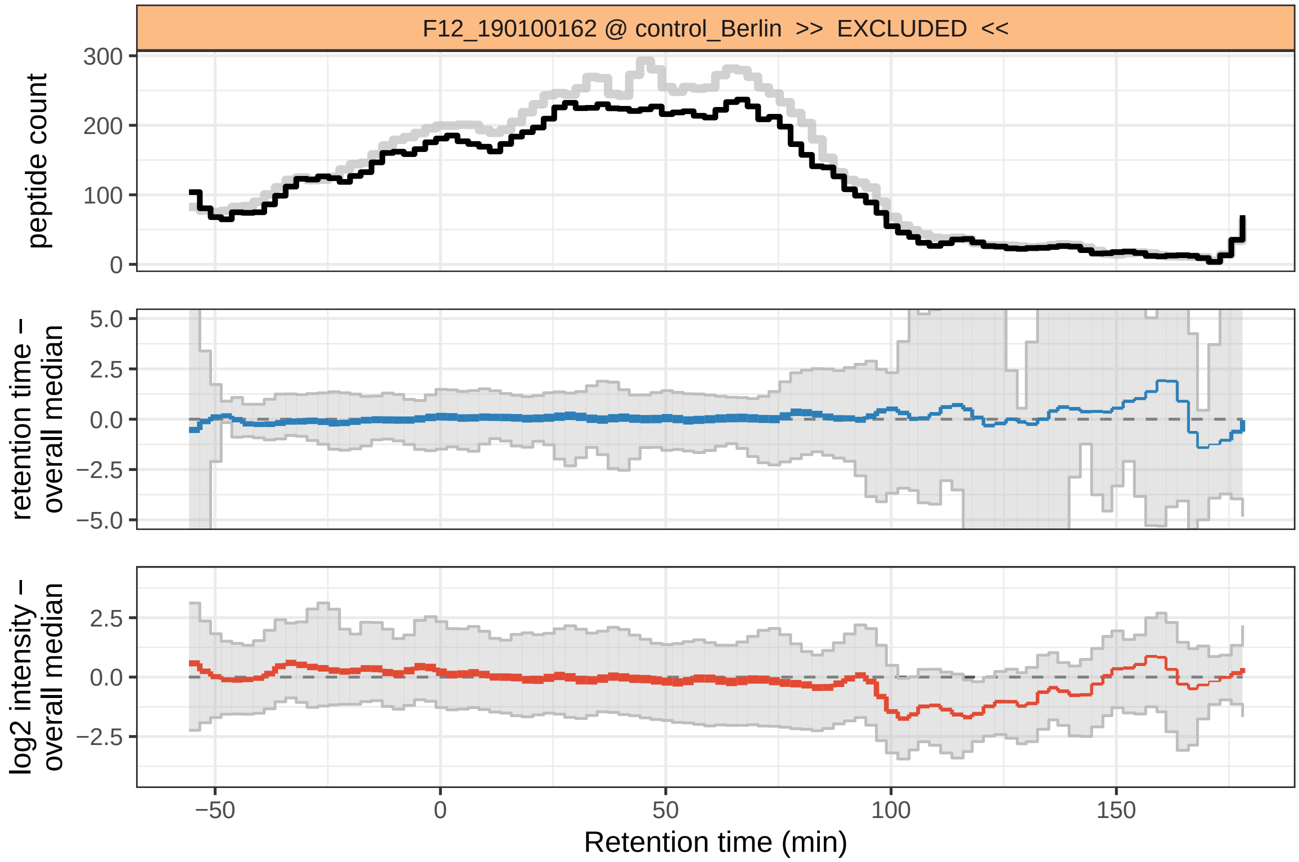
F12_190100162 @ control_Berlin >> EXCLUDED <<
peptide count
retention time – overall median
log2 intensity – overall median
Retention time (min)

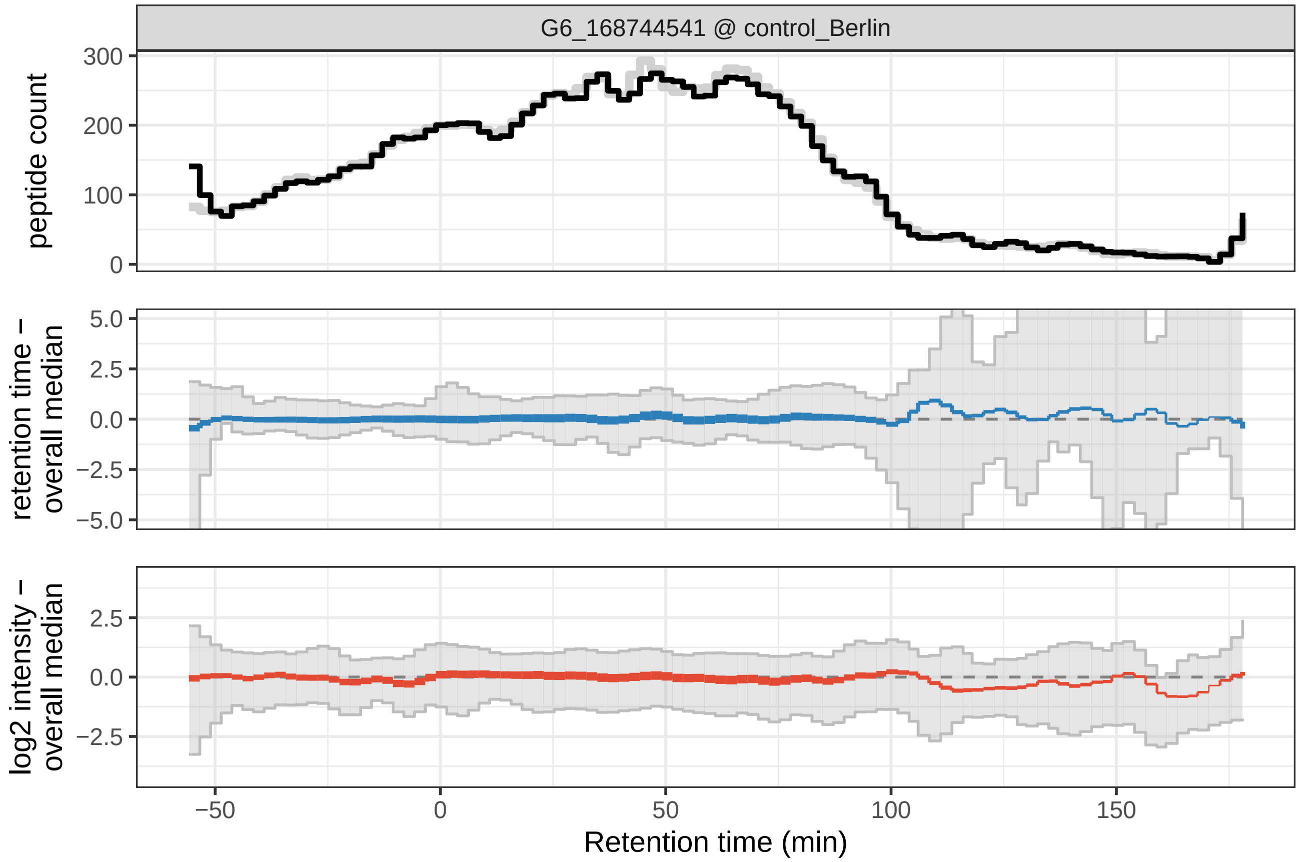
G6_168744541 @ control_Berlin
peptide count
retention time – overall median
log2 intensity – overall median
Retention time (min)

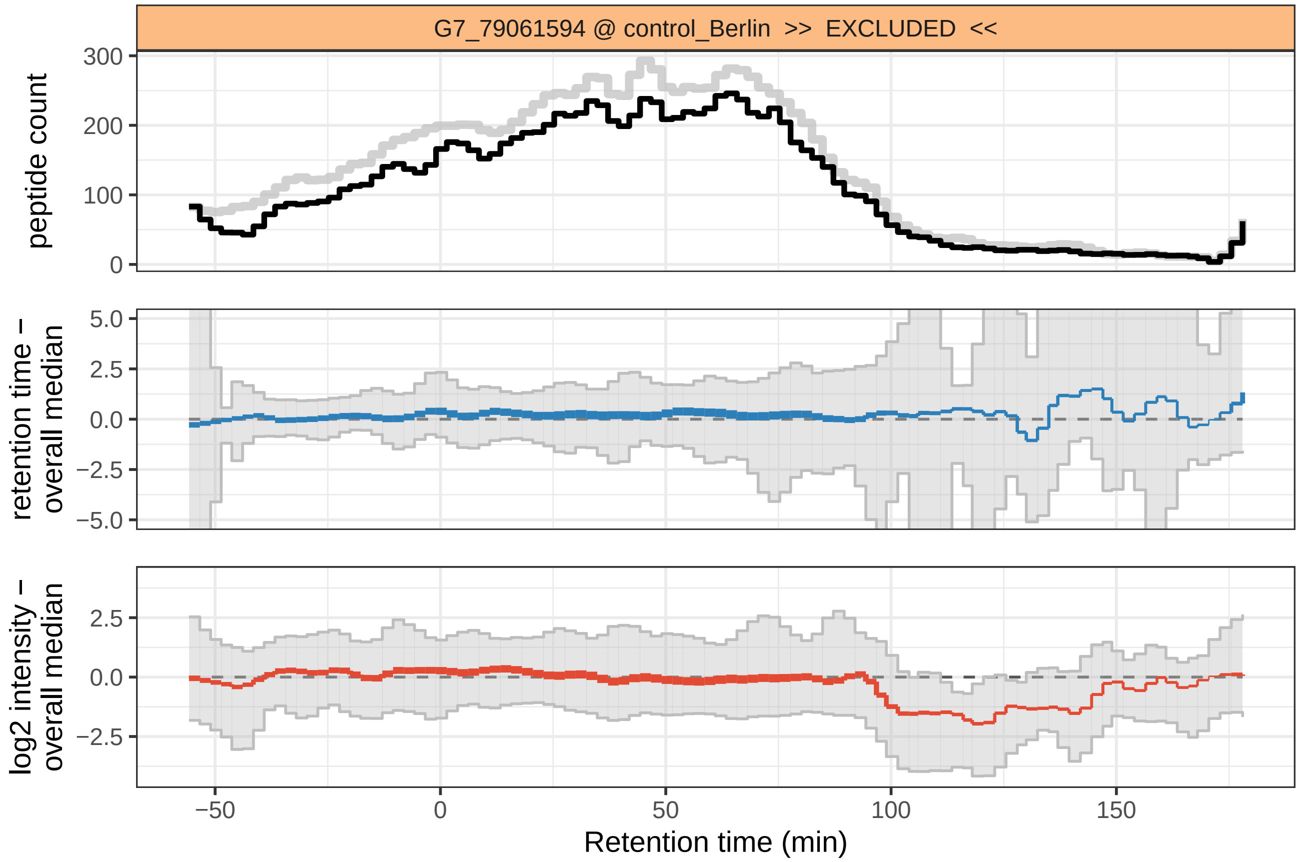
G7_79061594 @ control_Berlin >> EXCLUDED <<
peptide count
retention time – overall median
log2 intensity – overall median
Retention time (min)

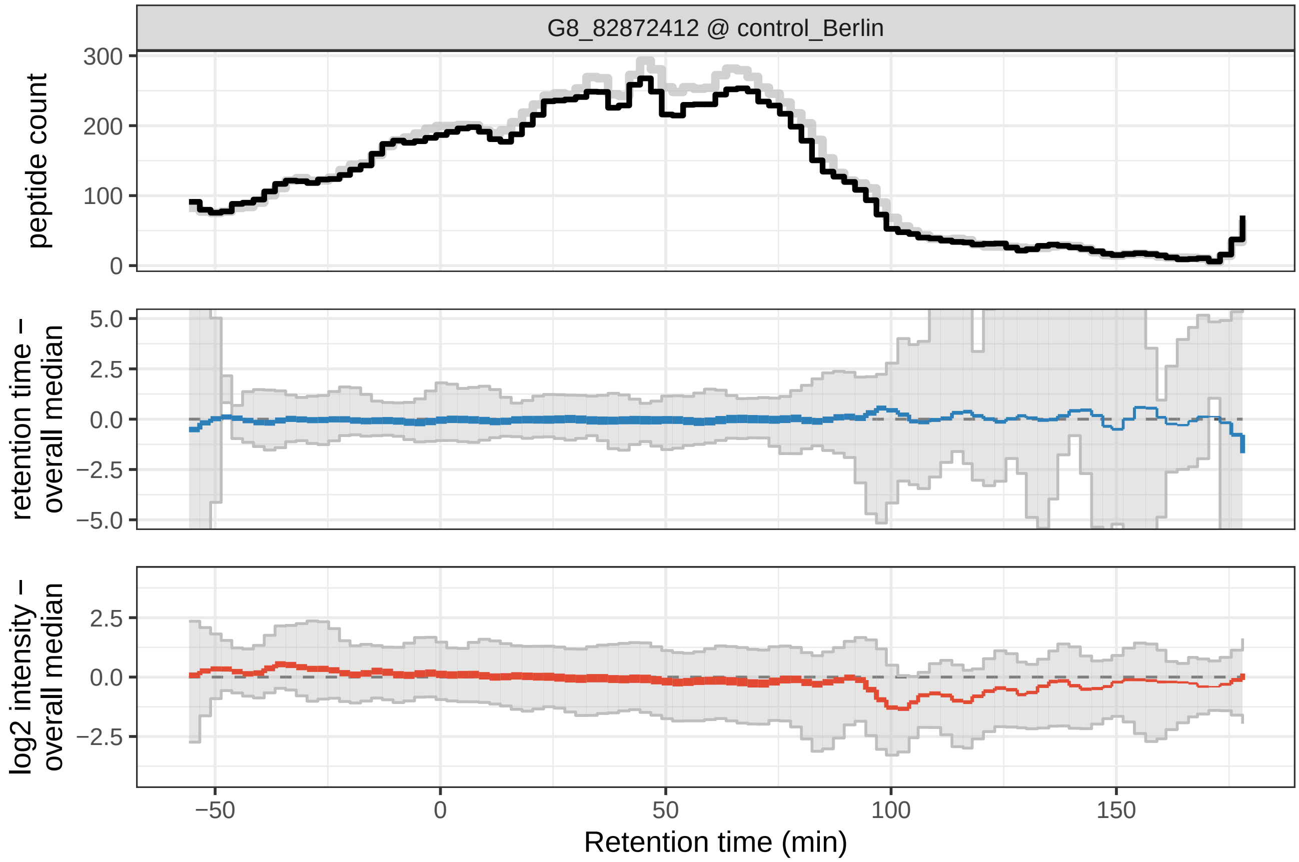
G8_82872412 @ control_Berlin
peptide count
retention time – overall median
log2 intensity – overall median
Retention time (min)

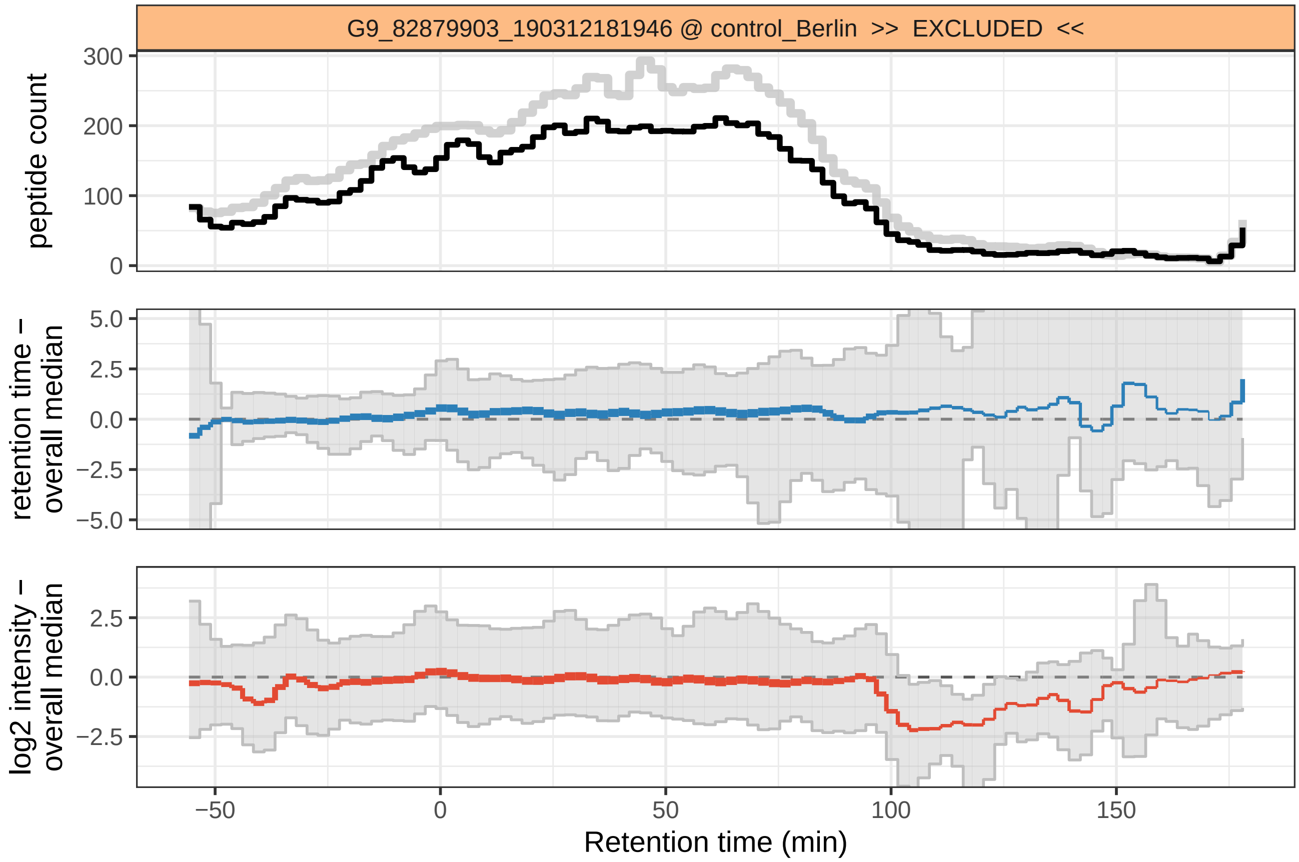
G9_82879903_190312181946 @ control_Berlin >> EXCLUDED <<
peptide count
300
200
100
0
retention time –
overall median
5.0
2.5
0.0
−2.5
−5.0
log2 intensity –
overall median
2.5
0.0
−2.5
−50
0
50
100
150
Retention time (min)

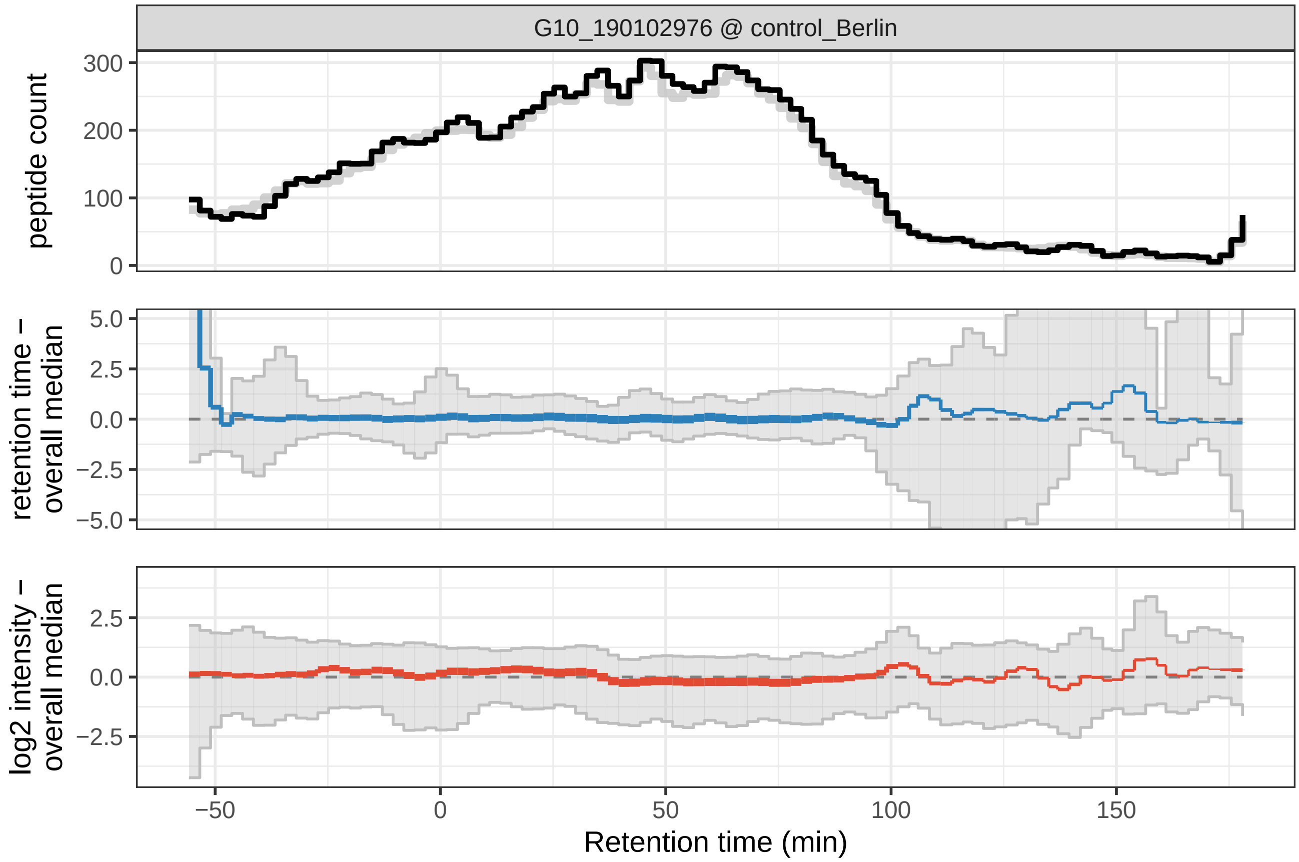
G10_190102976 @ control_Berlin
peptide count
300
200
100
0
retention time –
overall median
5.0
2.5
0.0
−2.5
−5.0
log2 intensity –
overall median
2.5
0.0
−2.5
−50
0
50
100
150
Retention time (min)

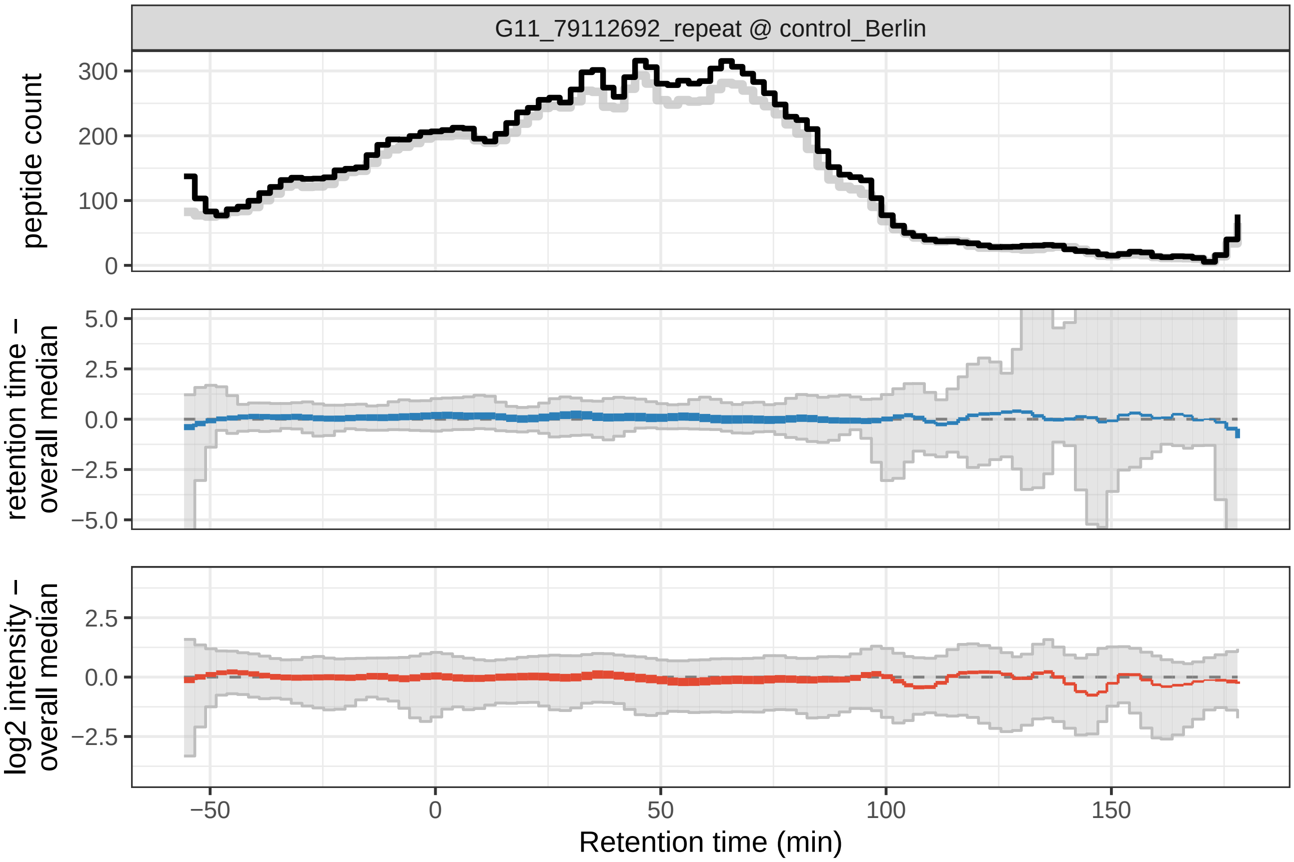
G11_79112692_repeat @ control_Berlin
peptide count
300
200
100
0
retention time – overall median
5.0
2.5
0.0
−2.5
−5.0
log2 intensity – overall median
2.5
0.0
−2.5
−50
0
50
100
150
Retention time (min)

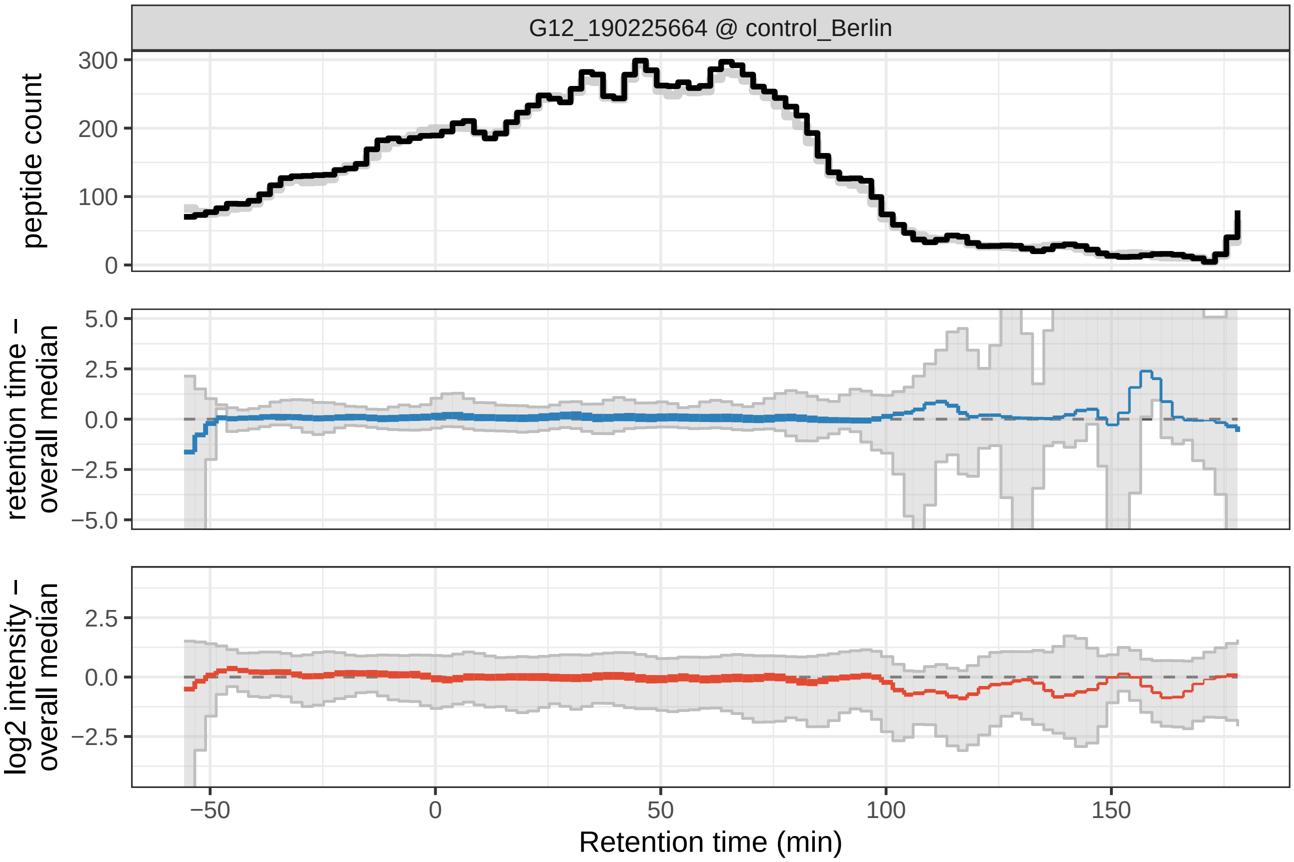
G12_190225664 @ control_Berlin
peptide count
300
200
100
0
retention time – overall median
5.0
2.5
0.0
−2.5
−5.0
log2 intensity – overall median
2.5
0.0
−2.5
−50
0
50
100
150
Retention time (min)

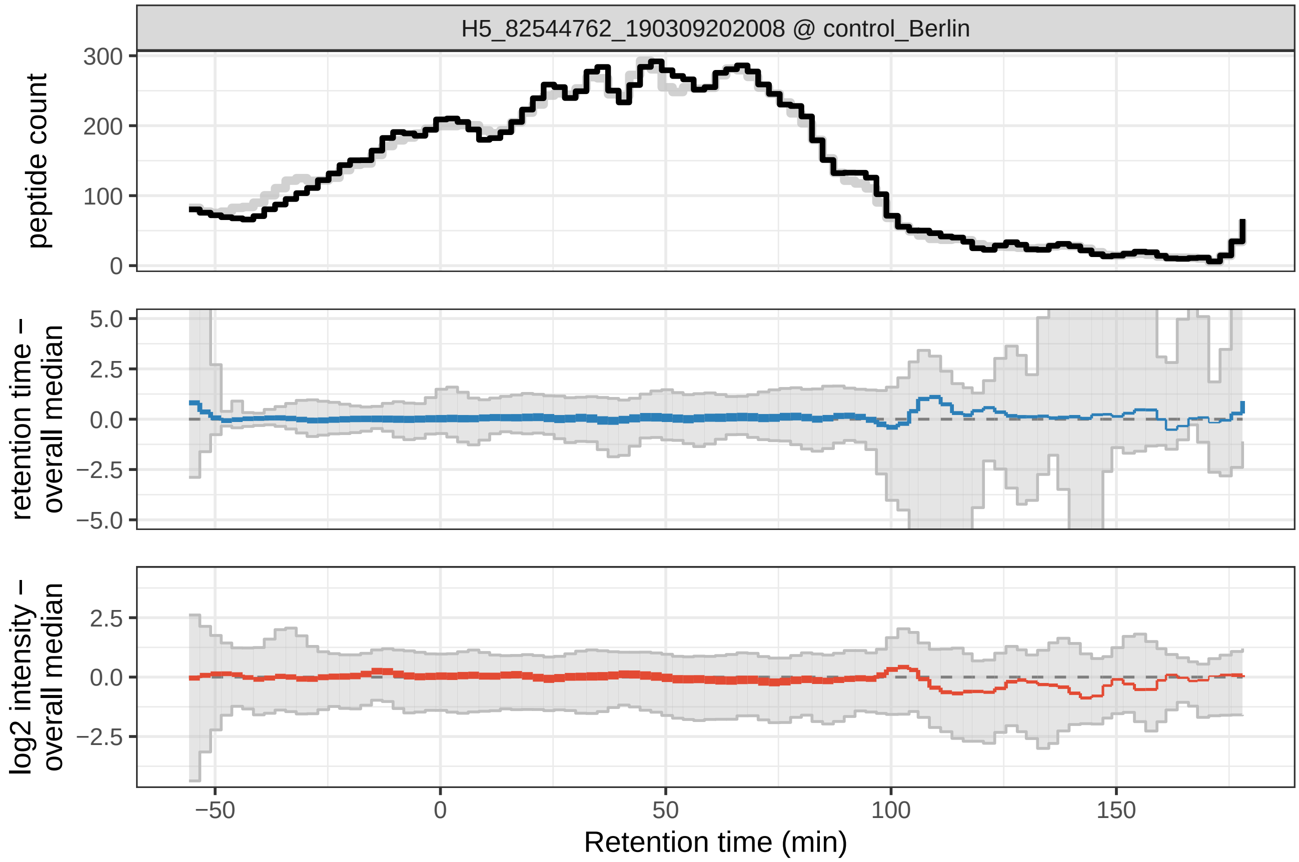
H5_82544762_190309202008 @ control_Berlin
peptide count
retention time – overall median
log2 intensity – overall median
Retention time (min)

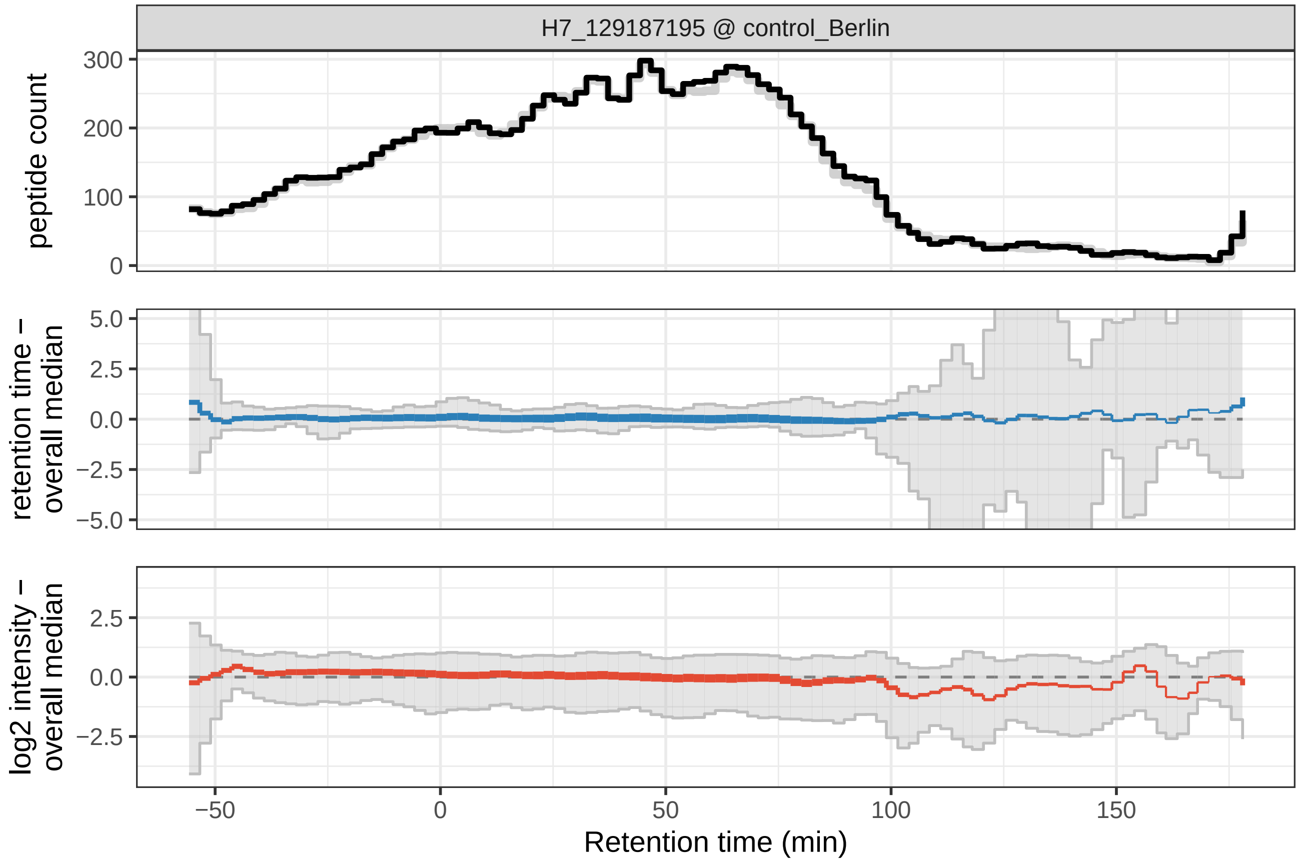
H7_129187195 @ control_Berlin
peptide count
retention time – overall median
log2 intensity – overall median
Retention time (min)

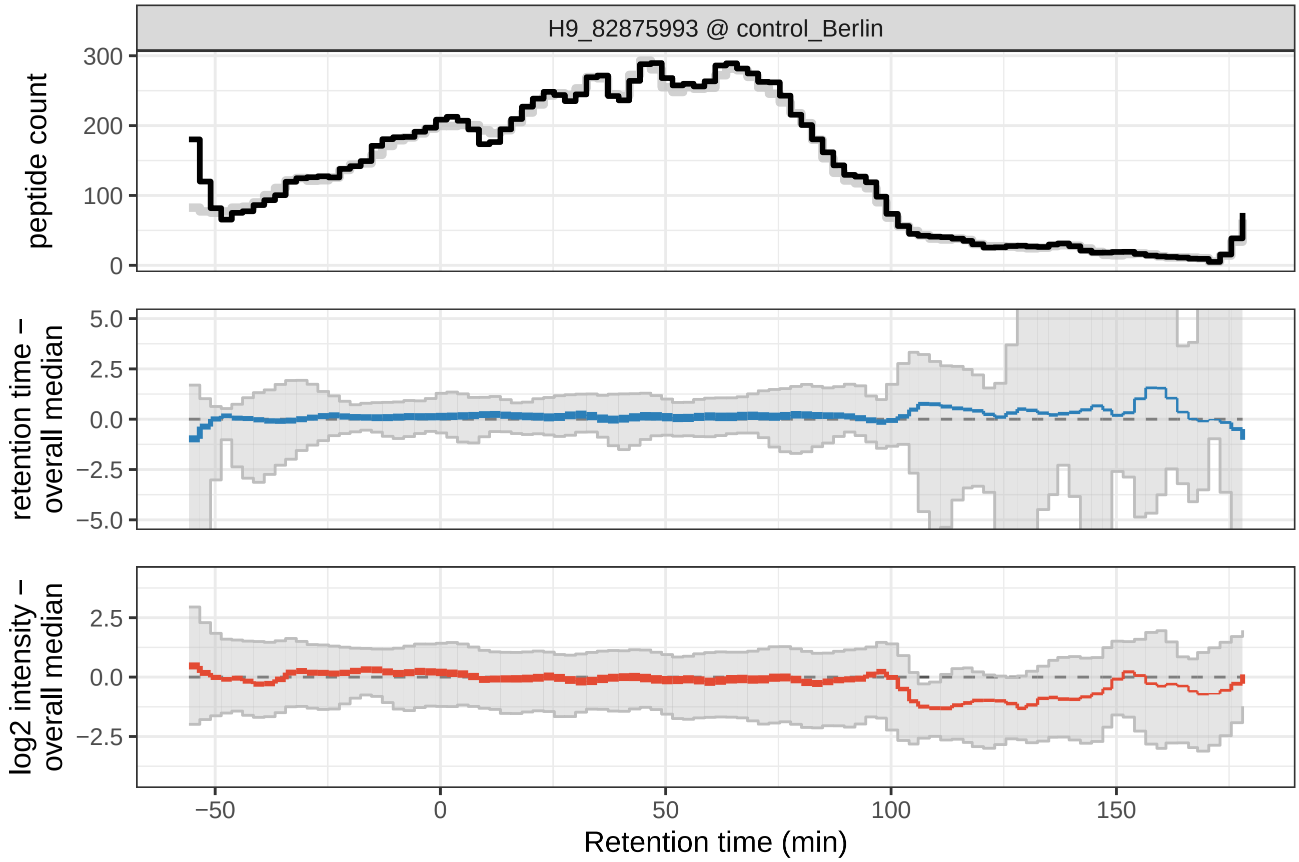

H9_82875993 @ control_Berlin
peptide count
retention time – overall median
log2 intensity – overall median
Retention time (min)

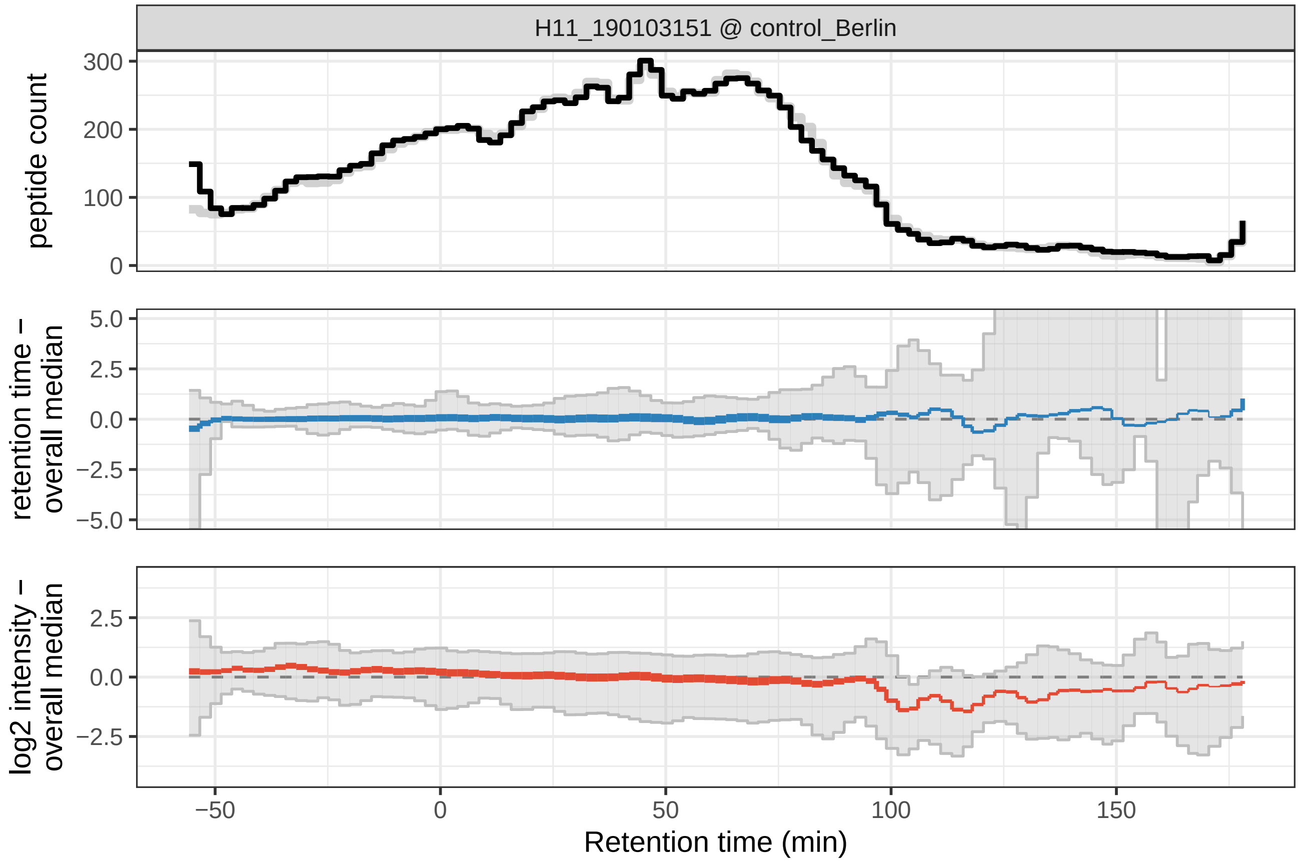

H11_190103151 @ control_Berlin
peptide count
retention time – overall median
log2 intensity – overall median
Retention time (min)

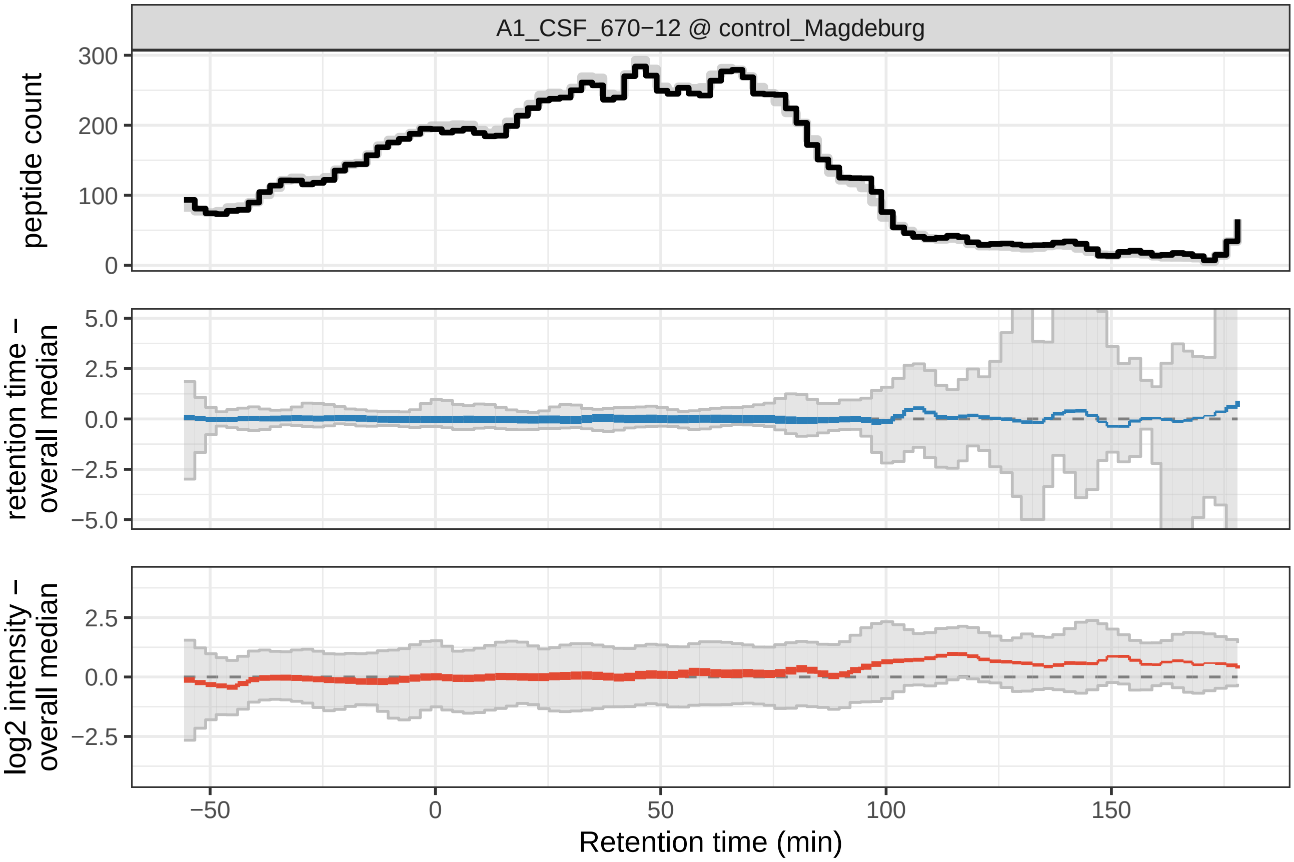
A1_CSF_670−12 @ control_Magdeburg
peptide count
retention time – overall median
log2 intensity – overall median
Retention time (min)

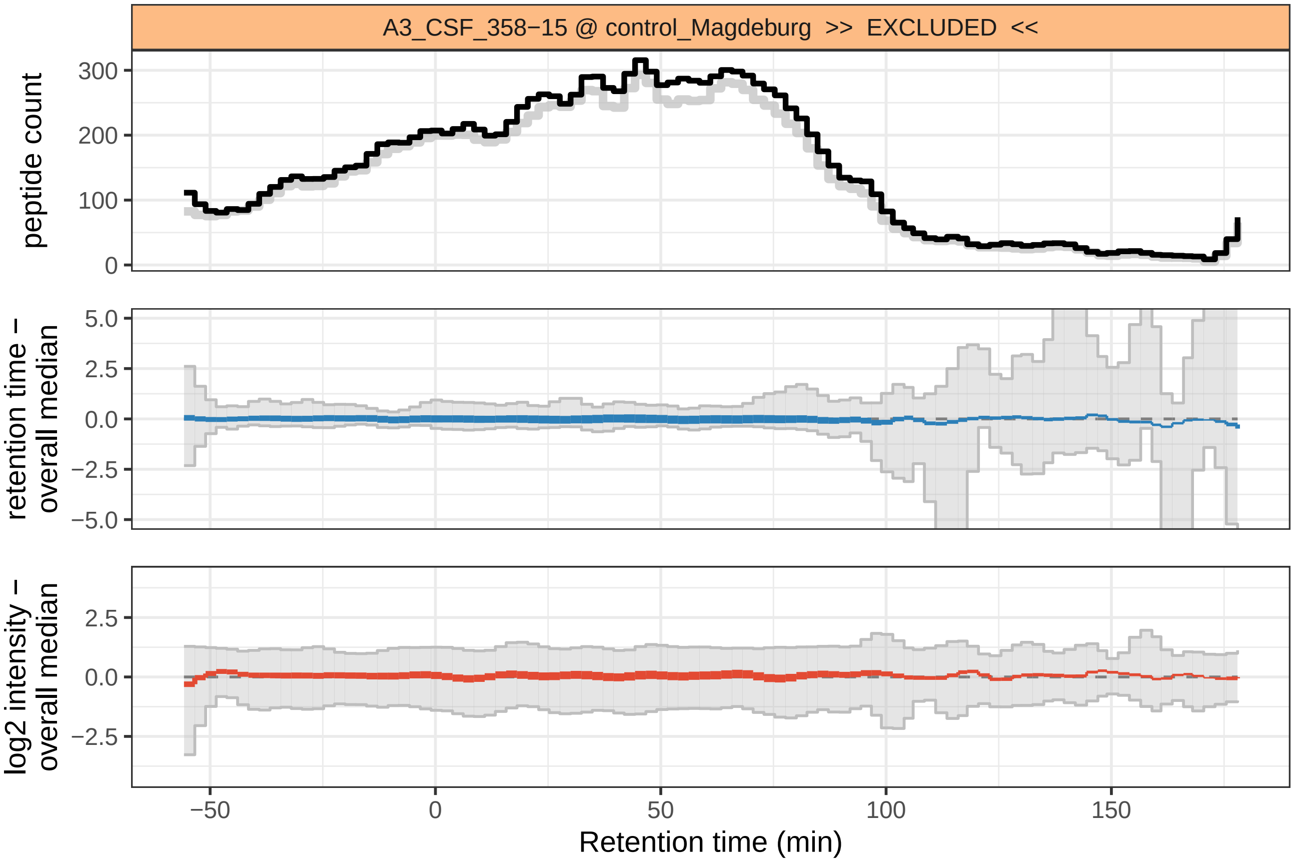
A3_CSF_358−15 @ control_Magdeburg >> EXCLUDED <<
peptide count
retention time – overall median
log2 intensity – overall median
Retention time (min)

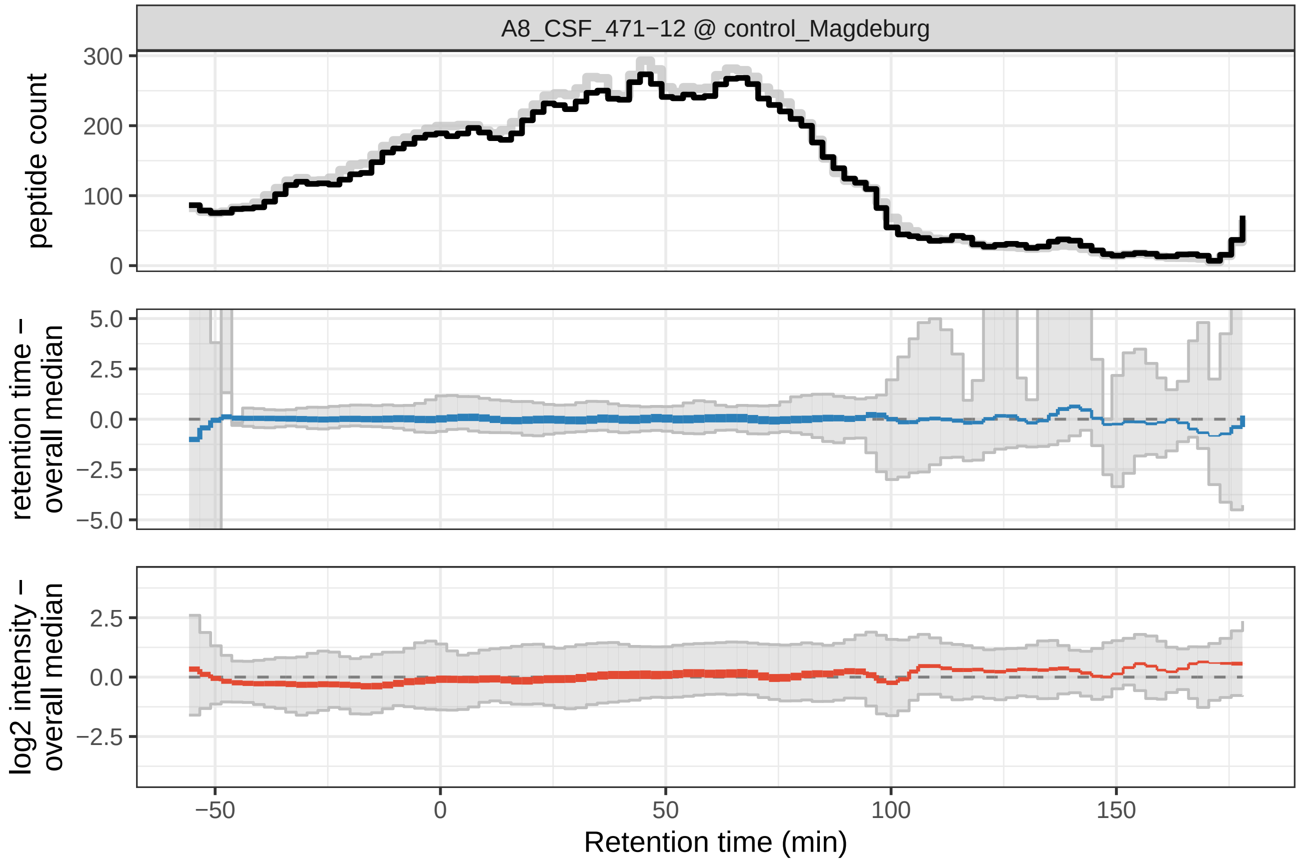
A8_CSF_471−12 @ control_Magdeburg
peptide count
retention time – overall median
log2 intensity – overall median
Retention time (min)

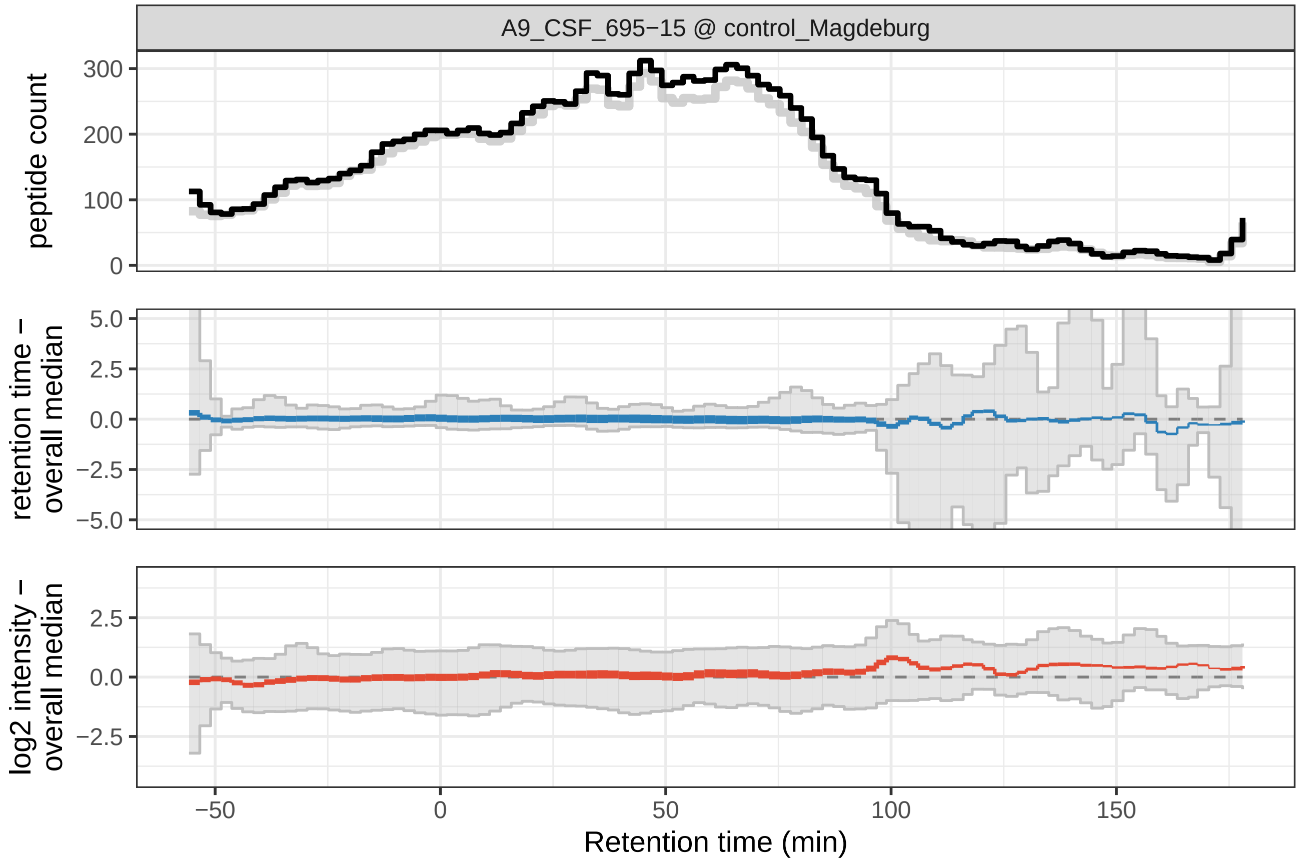
A9_CSF_695−15 @ control_Magdeburg
peptide count
retention time – overall median
log2 intensity – overall median
Retention time (min)

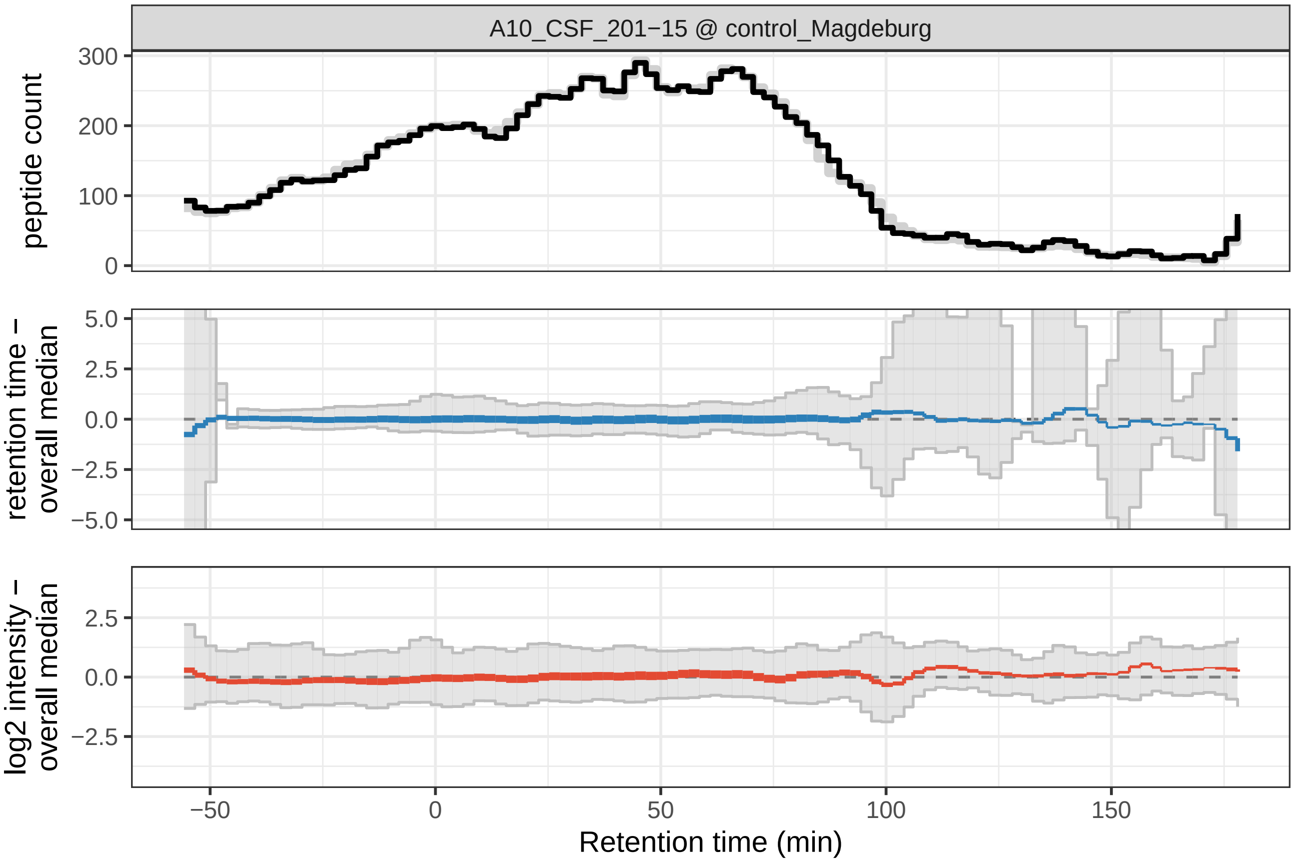
A10_CSF_201−15 @ control_Magdeburg
peptide count
retention time – overall median
log2 intensity – overall median
Retention time (min)

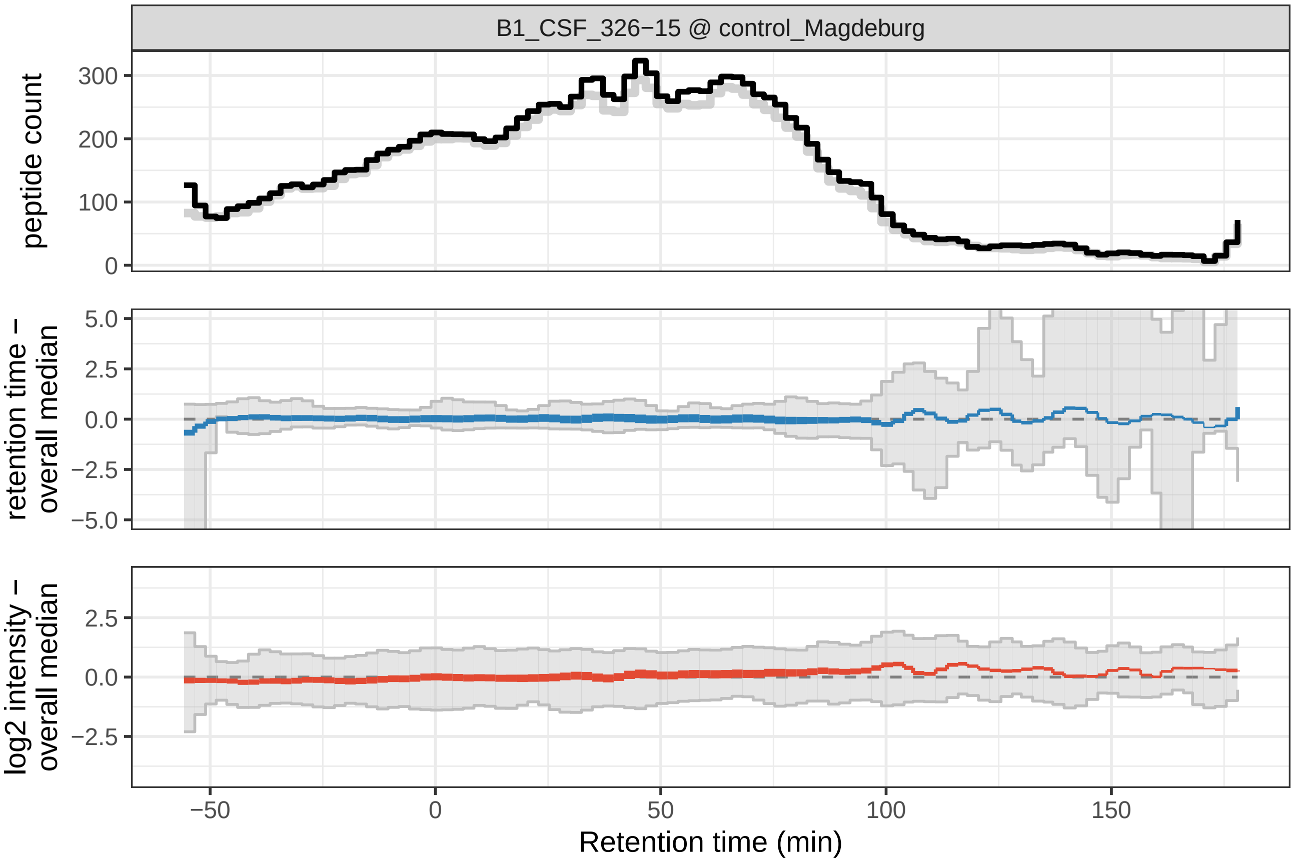
B1_CSF_326−15 @ control_Magdeburg
peptide count
retention time – overall median
log2 intensity – overall median
Retention time (min)

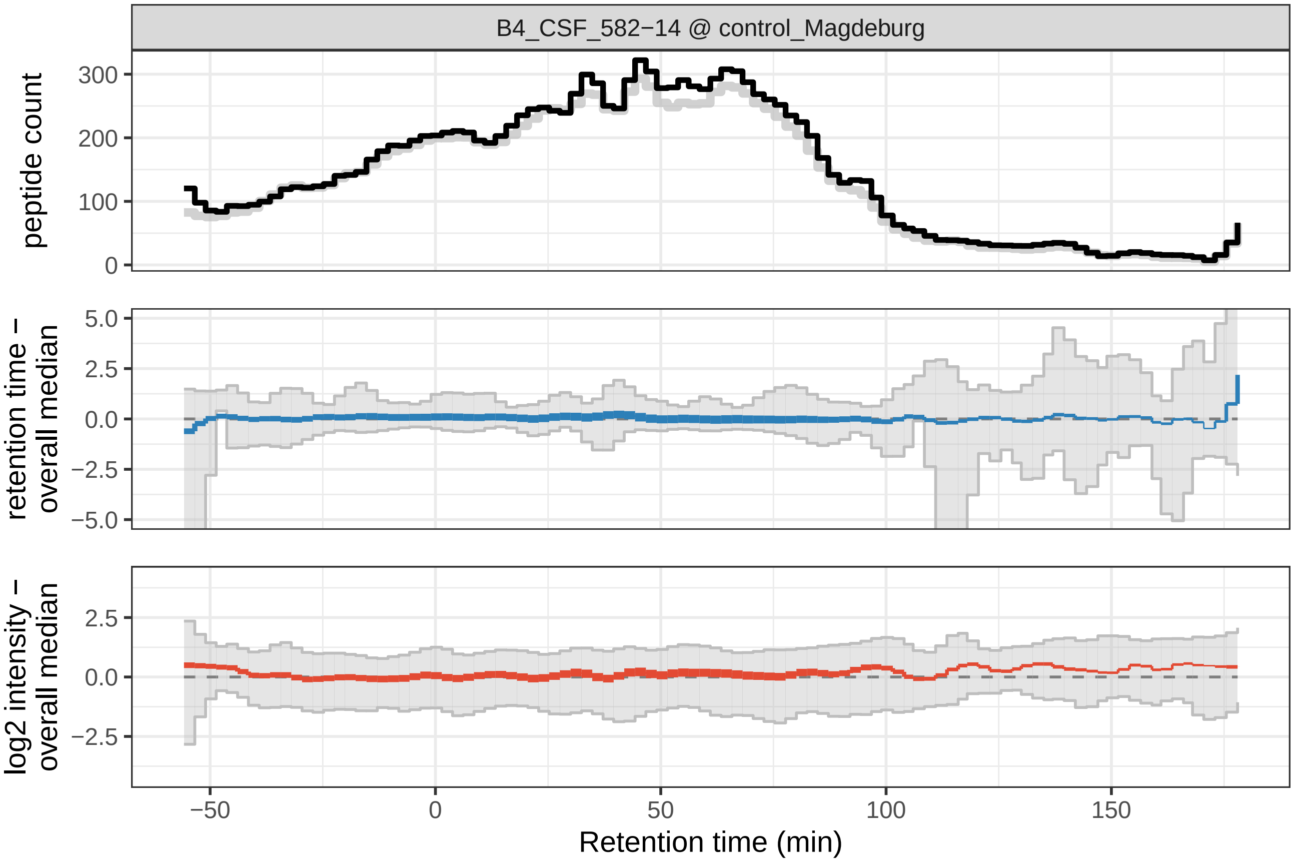
B4_CSF_582−14 @ control_Magdeburg
peptide count
300
200
100
0
retention time –
overall median
5.0
2.5
0.0
−2.5
−5.0
log2 intensity –
overall median
2.5
0.0
−2.5
−50
0
50
100
150
Retention time (min)

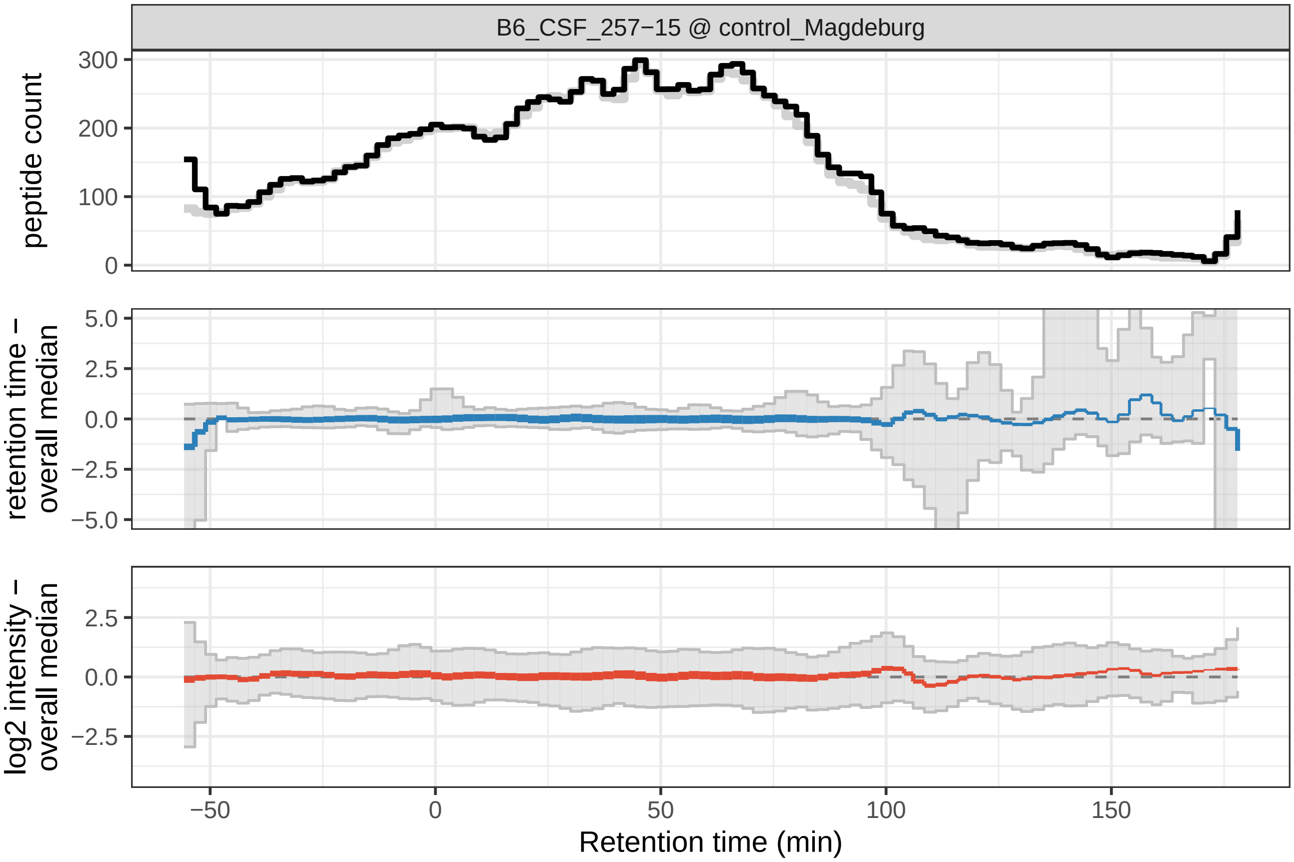
B6_CSF_257−15 @ control_Magdeburg
peptide count
300
200
100
0
retention time –
overall median
5.0
2.5
0.0
−2.5
−5.0
log2 intensity –
overall median
2.5
0.0
−2.5
−50
0
50
100
150
Retention time (min)

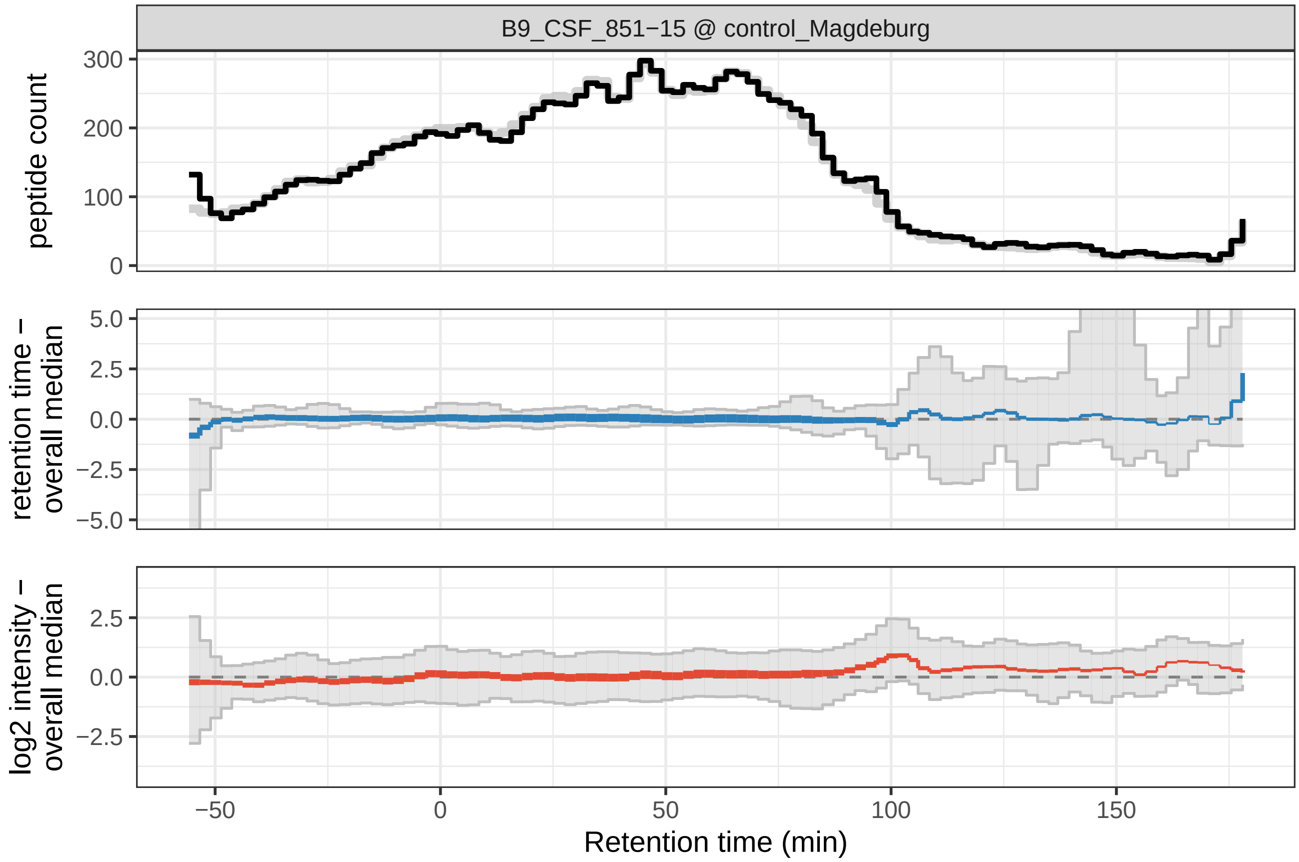
B9_CSF_851−15 @ control_Magdeburg
peptide count
retention time – overall median
log2 intensity – overall median
Retention time (min)

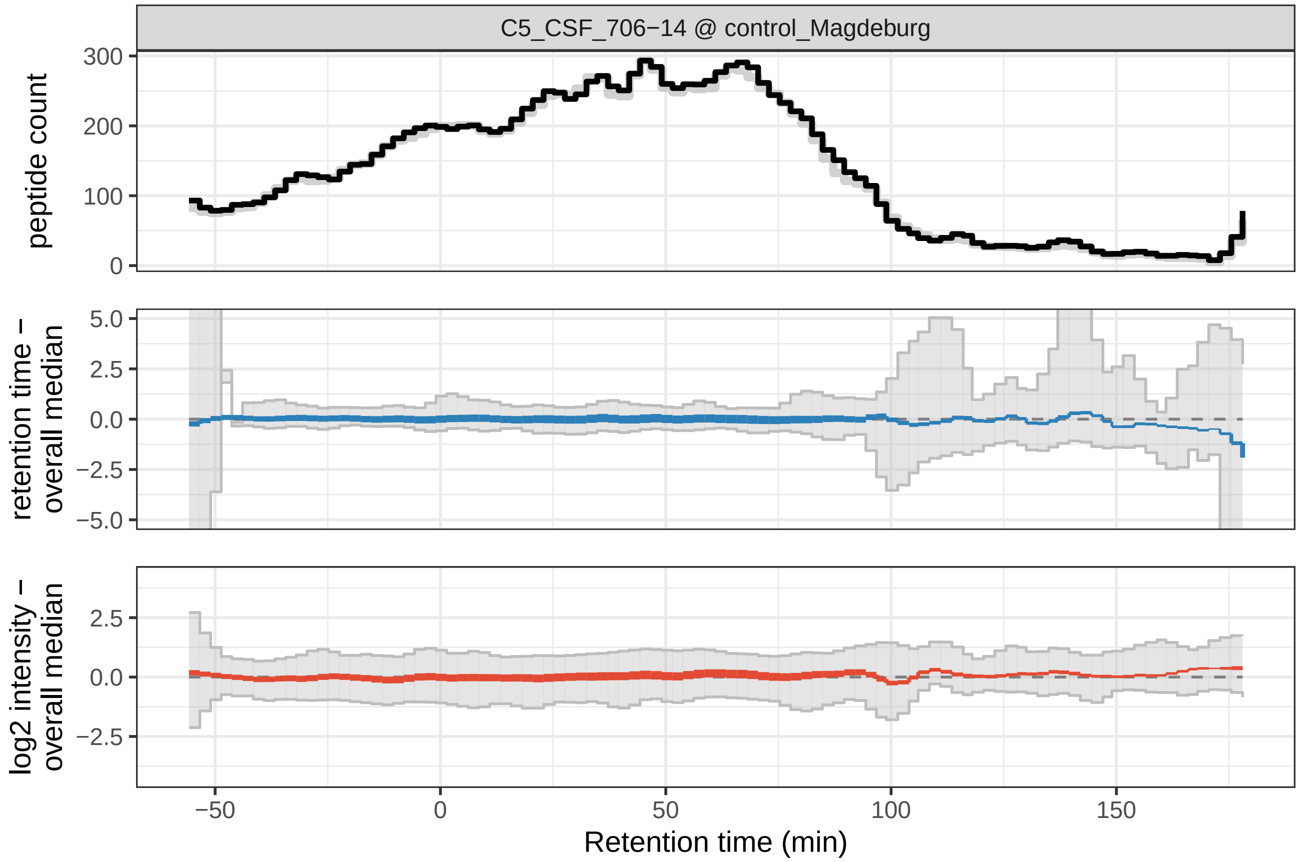
C5_CSF_706−14 @ control_Magdeburg
peptide count
retention time – overall median
log2 intensity – overall median
Retention time (min)

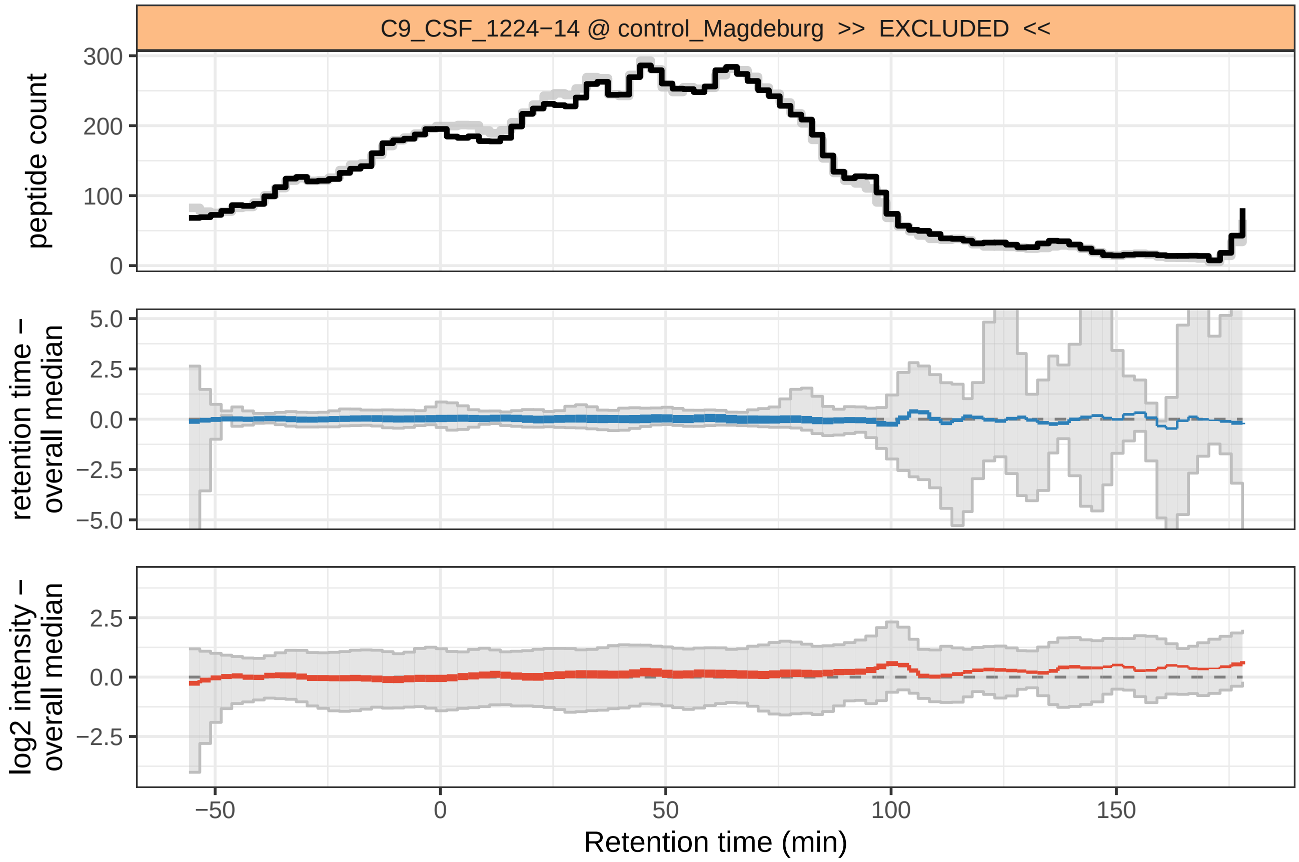
C9_CSF_1224−14 @ control_Magdeburg >> EXCLUDED <<
peptide count
retention time − overall median
log2 intensity − overall median
Retention time (min)

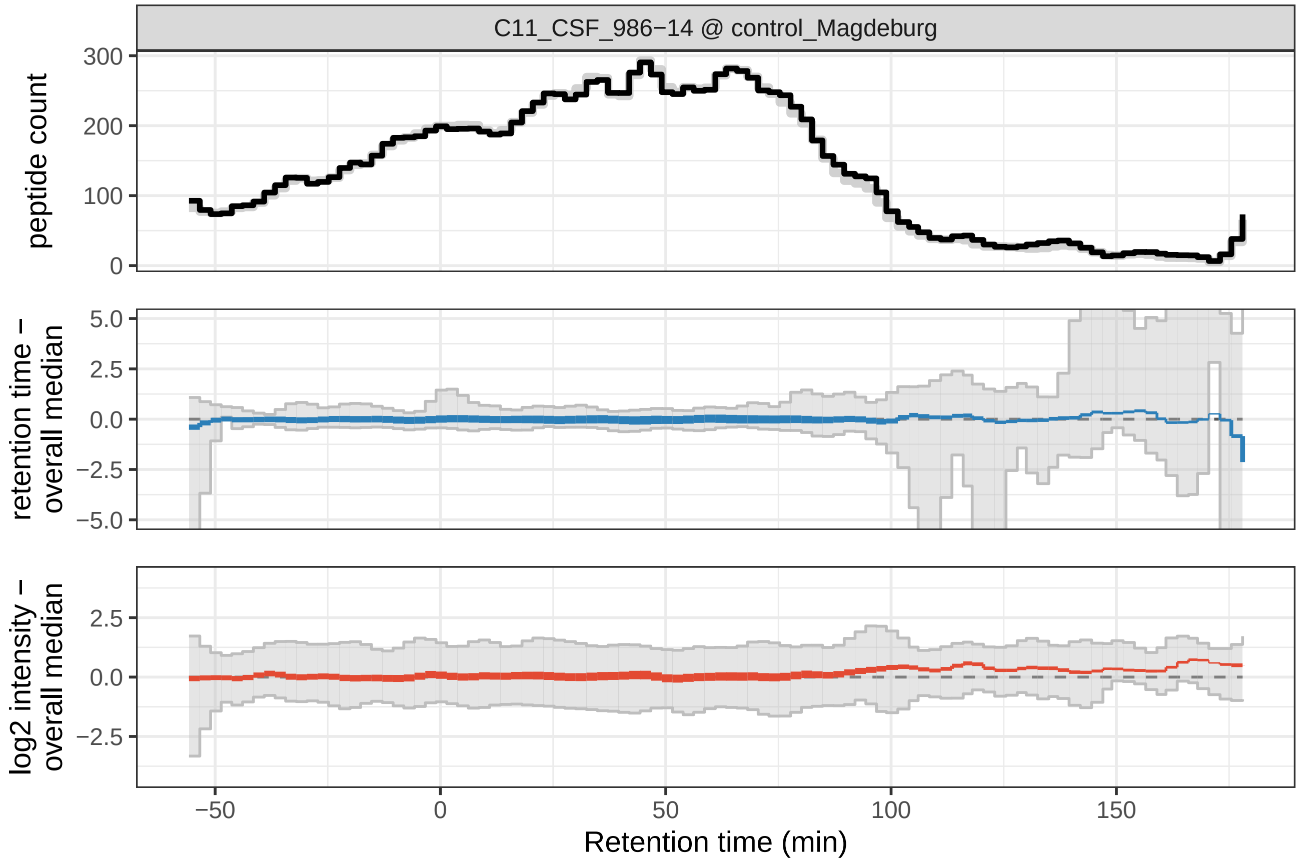
C11_CSF_986−14 @ control_Magdeburg
peptide count
retention time − overall median
log2 intensity − overall median
Retention time (min)

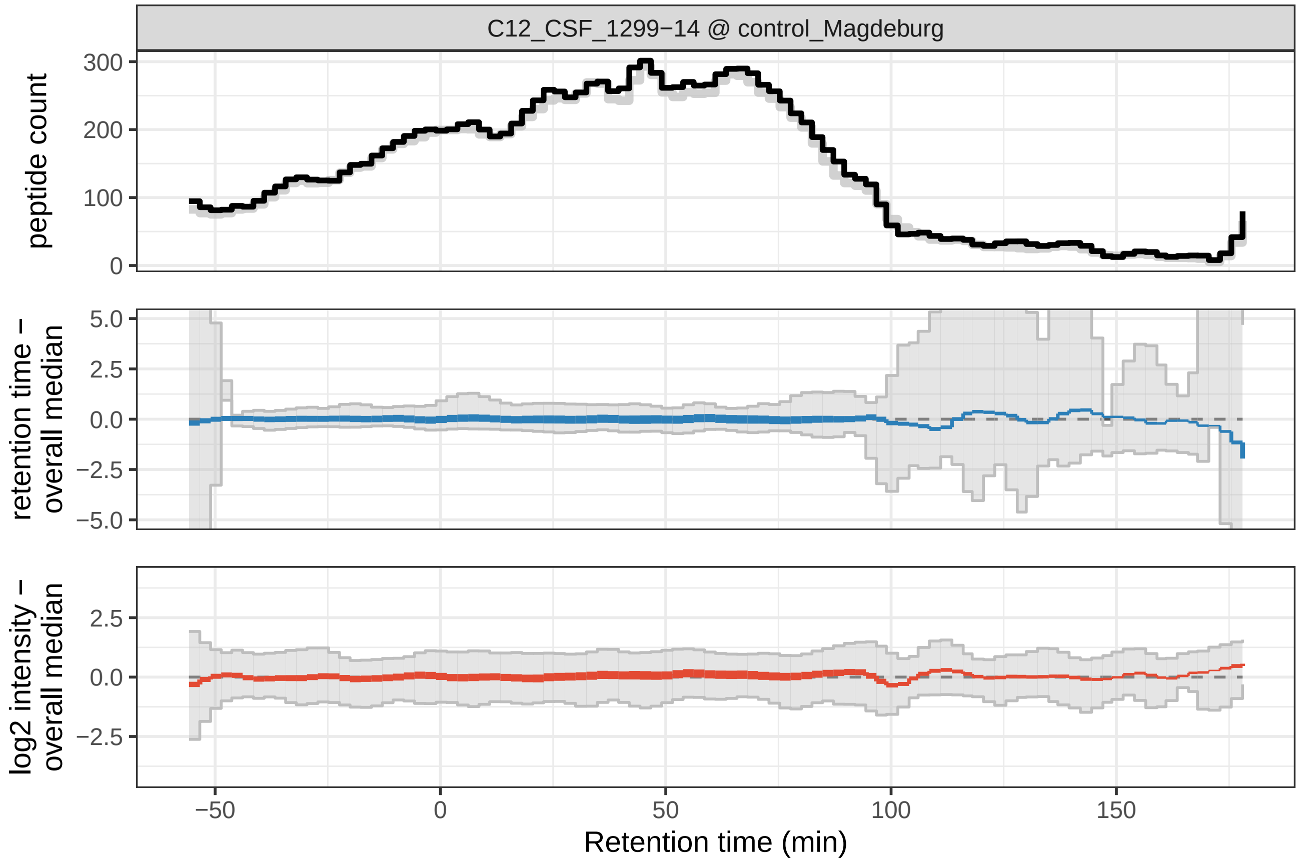
C12_CSF_1299−14 @ control_Magdeburg
peptide count
300
200
100
0
retention time –
overall median
5.0
2.5
0.0
−2.5
−5.0
log2 intensity –
overall median
2.5
0.0
−2.5
−50
0
50
100
150
Retention time (min)

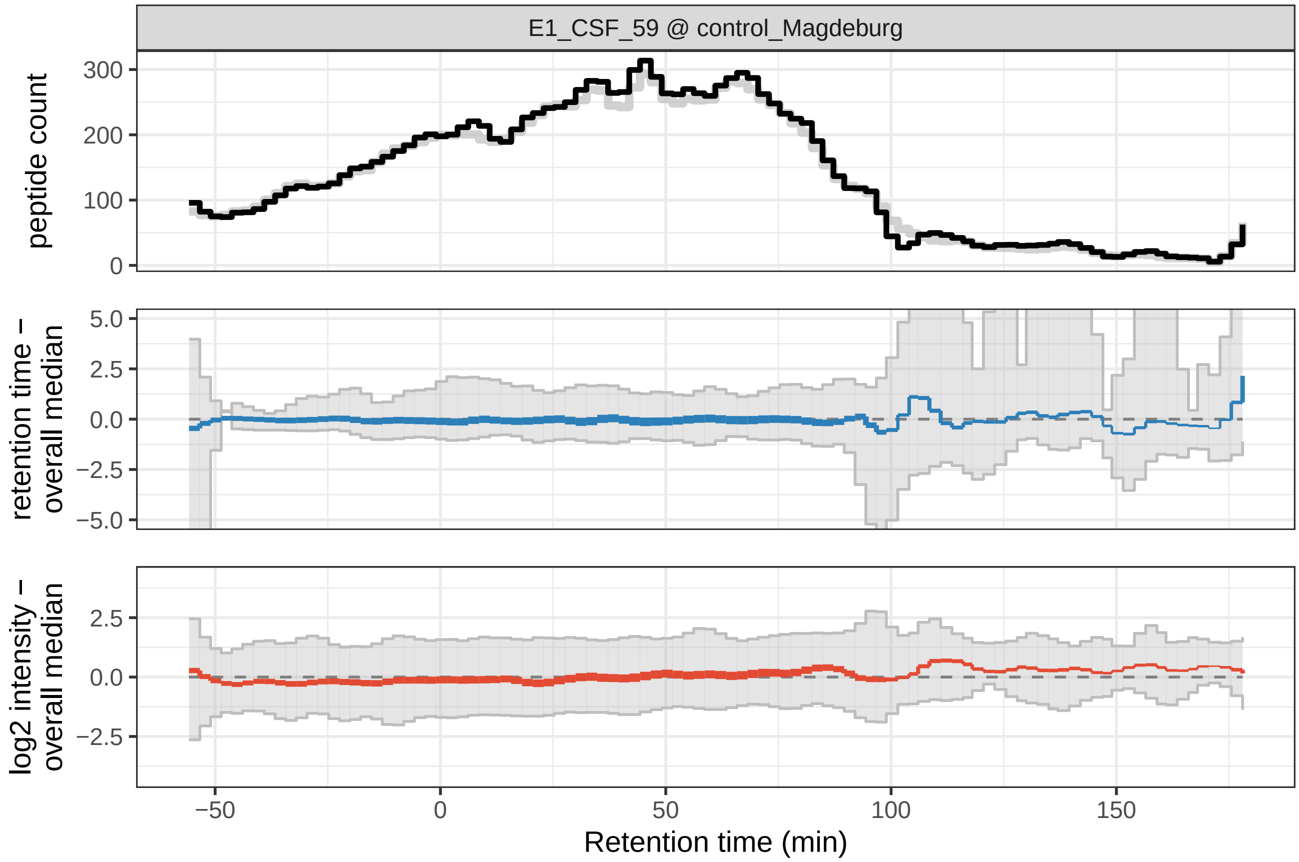
E1_CSF_59 @ control_Magdeburg
peptide count
300
200
100
0
retention time –
overall median
5.0
2.5
0.0
−2.5
−5.0
log2 intensity –
overall median
2.5
0.0
−2.5
−50
0
50
100
150
Retention time (min)

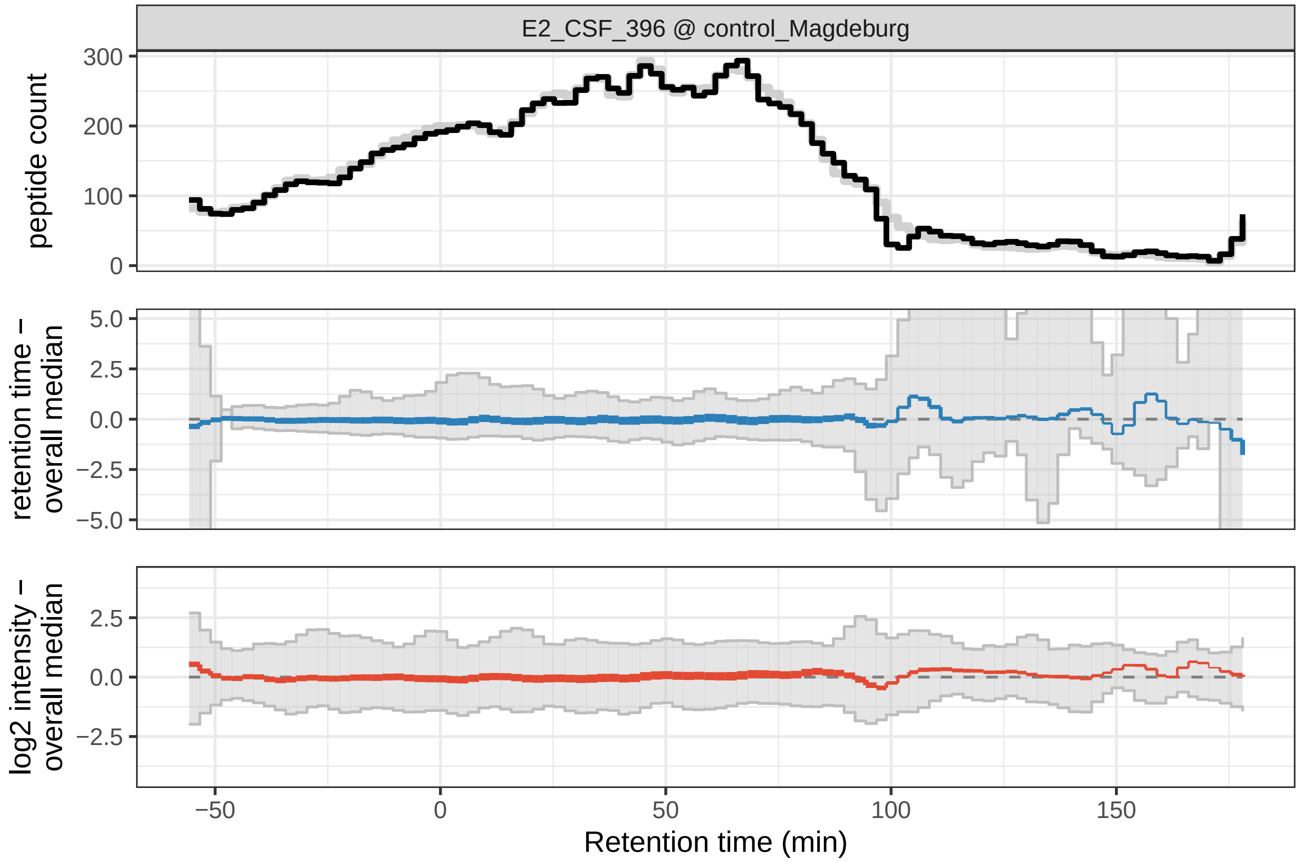
E2_CSF_396 @ control_Magdeburg
peptide count
300
200
100
0
retention time –
overall median
5.0
2.5
0.0
−2.5
−5.0
log2 intensity –
overall median
2.5
0.0
−2.5
−50
0
50
100
150
Retention time (min)

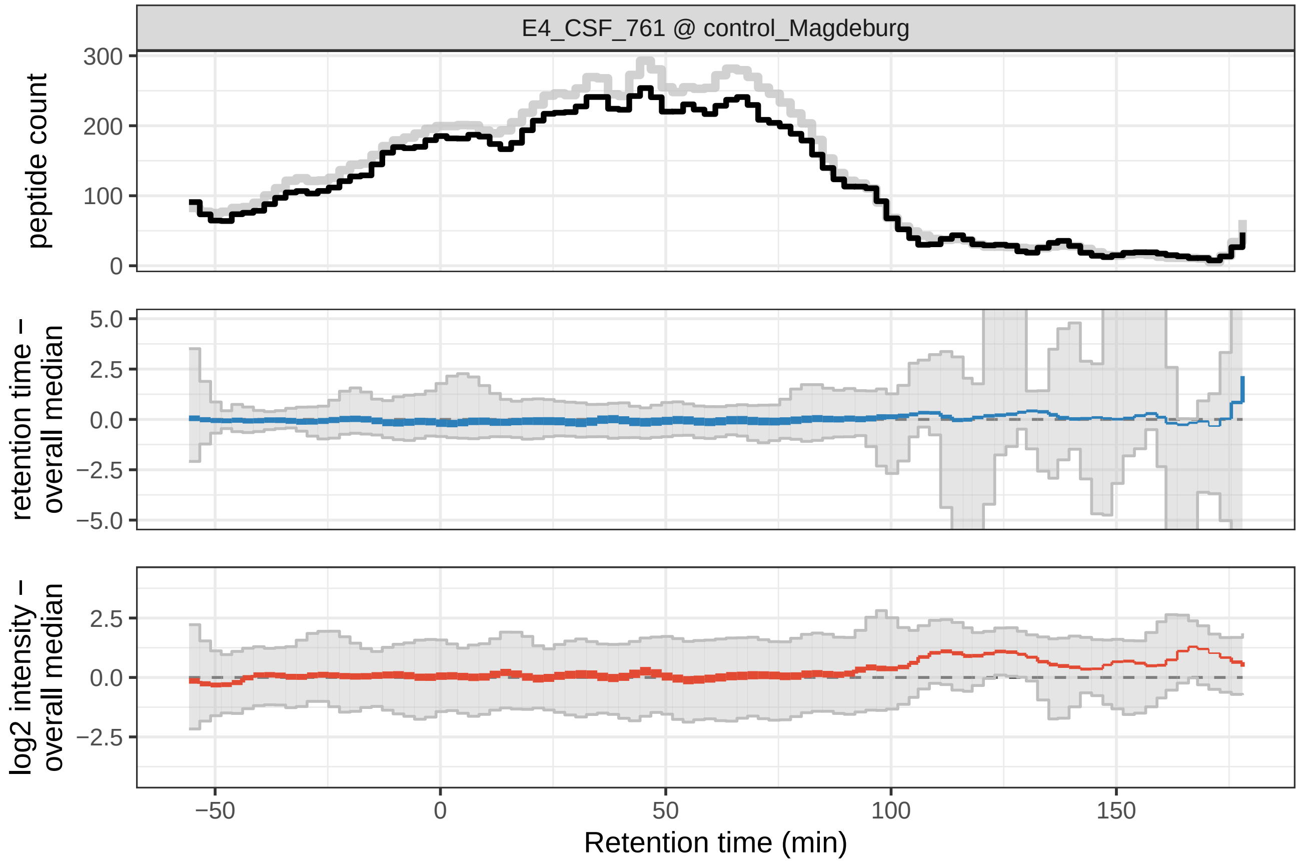
E4_CSF_761 @ control_Magdeburg
peptide count
300
200
100
0
retention time –
overall median
5.0
2.5
0.0
−2.5
−5.0
log2 intensity –
overall median
2.5
0.0
−2.5
−50
0
50
100
150
Retention time (min)

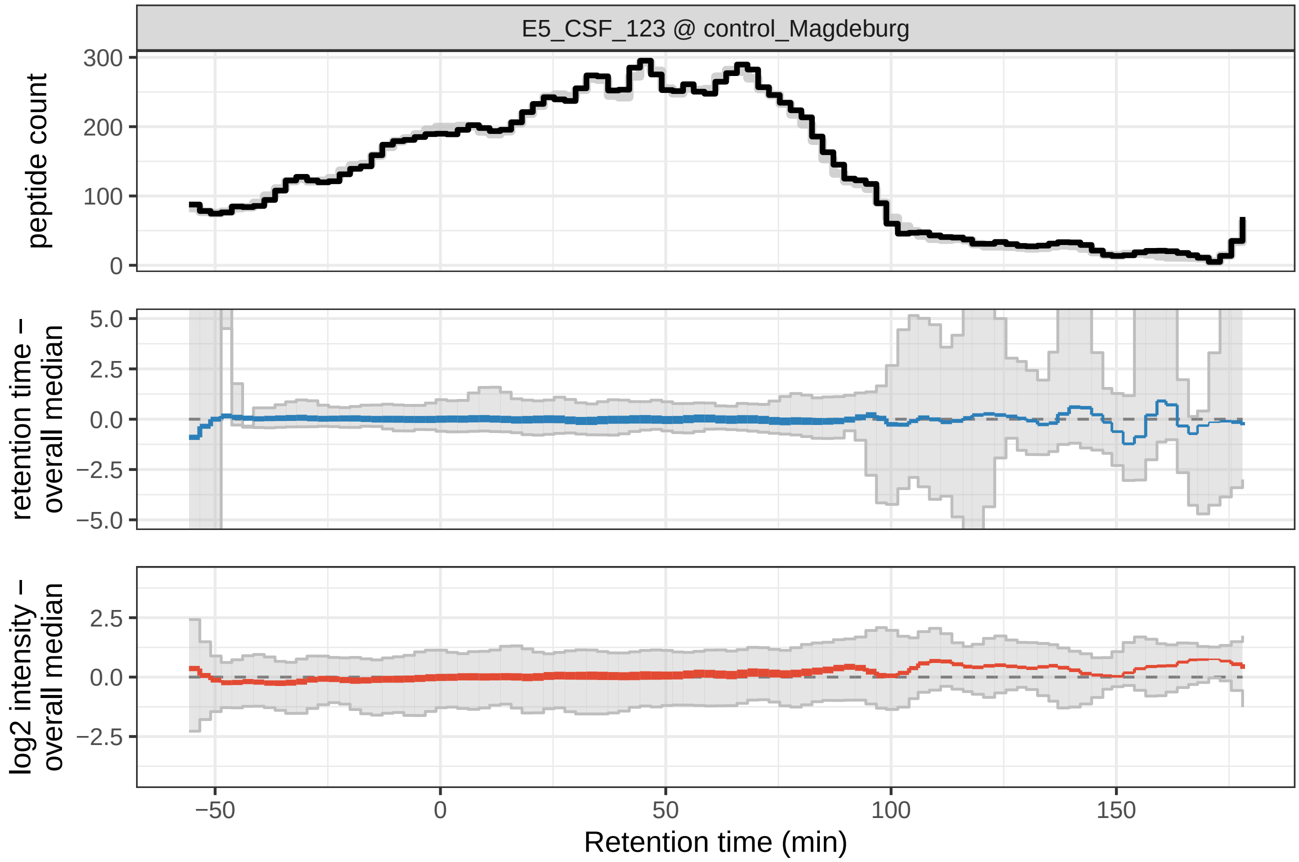

E5_CSF_123 @ control_Magdeburg
peptide count
retention time – overall median
log2 intensity – overall median
Retention time (min)

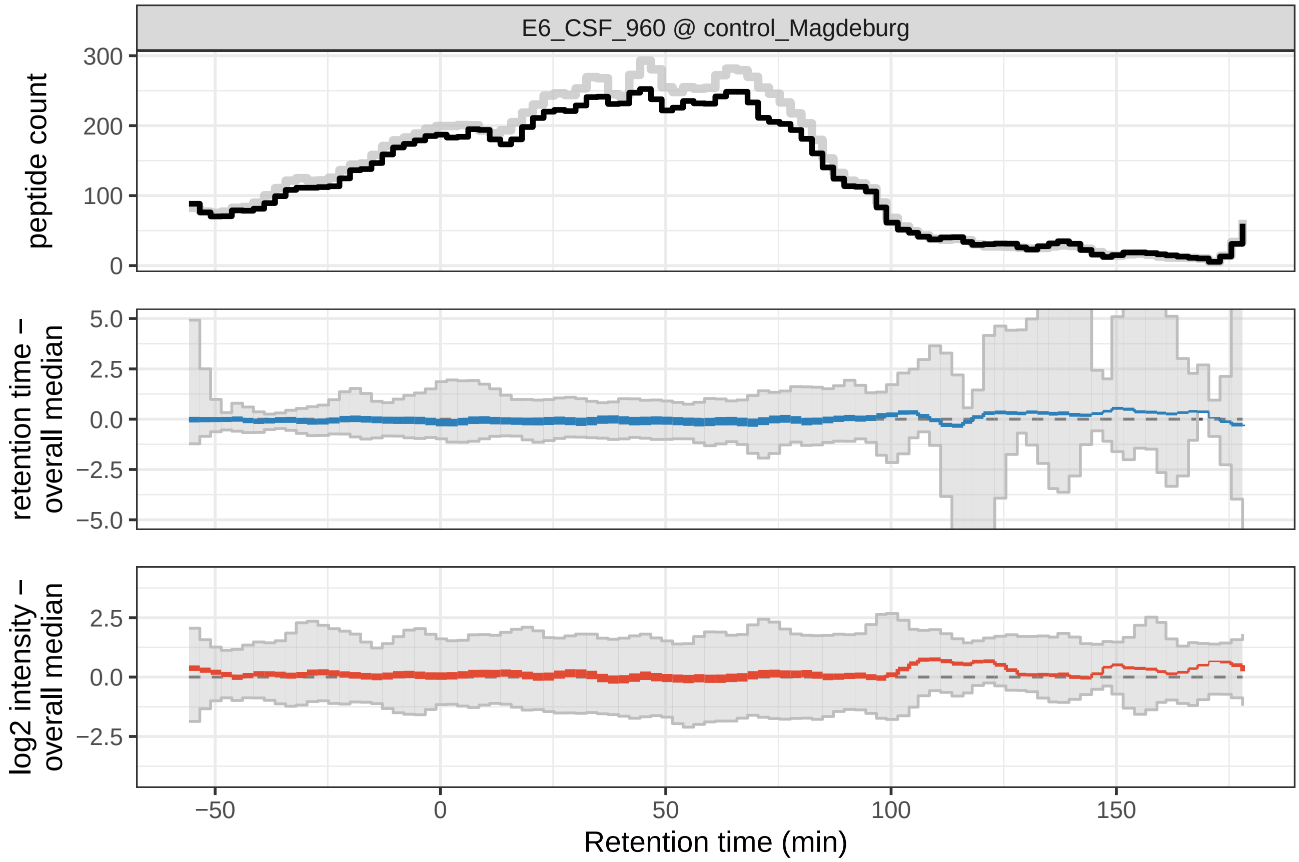

E6_CSF_960 @ control_Magdeburg
peptide count
retention time – overall median
log2 intensity – overall median
Retention time (min)

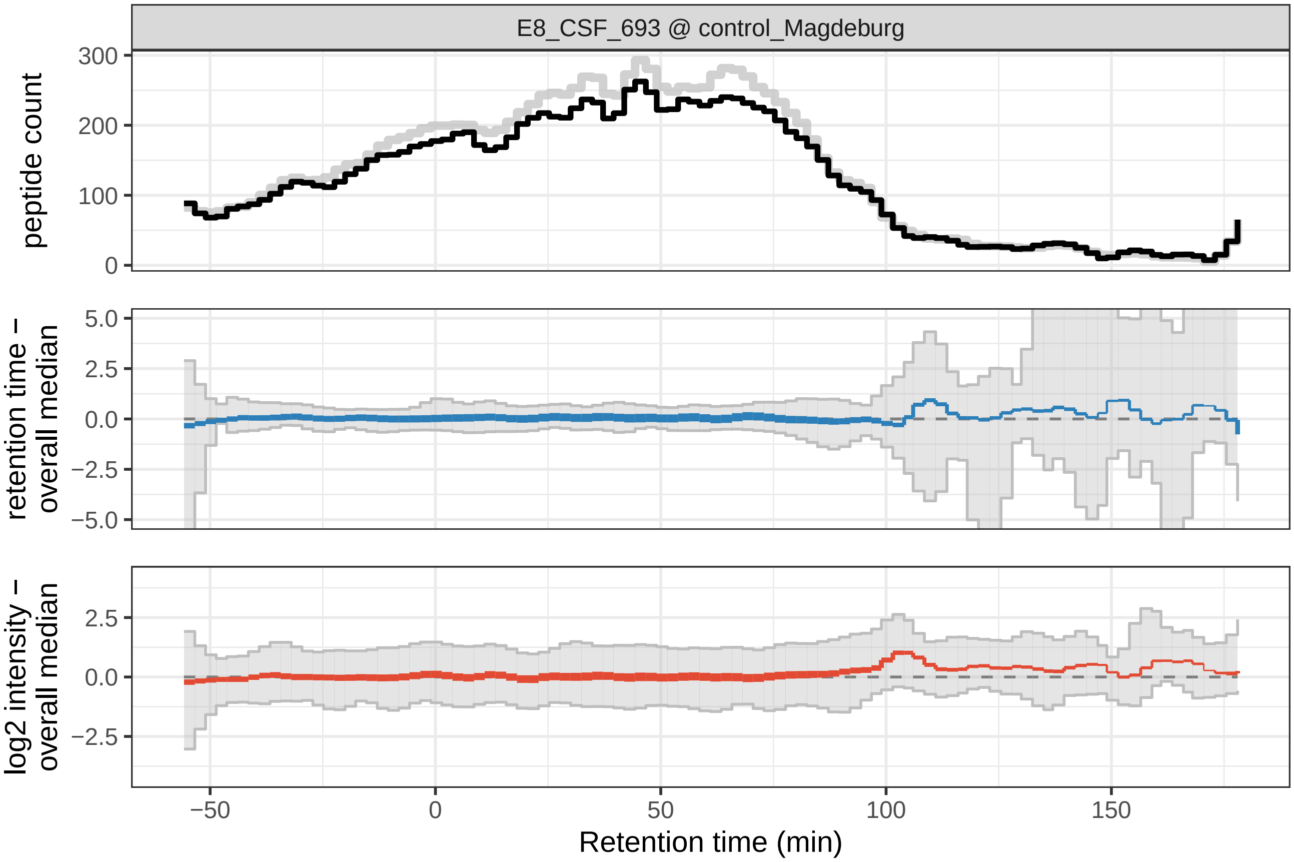
E8_CSF_693 @ control_Magdeburg
peptide count
300
200
100
0
retention time –
overall median
5.0
2.5
0.0
−2.5
−5.0
log2 intensity –
overall median
2.5
0.0
−2.5
−50
0
50
100
150
Retention time (min)

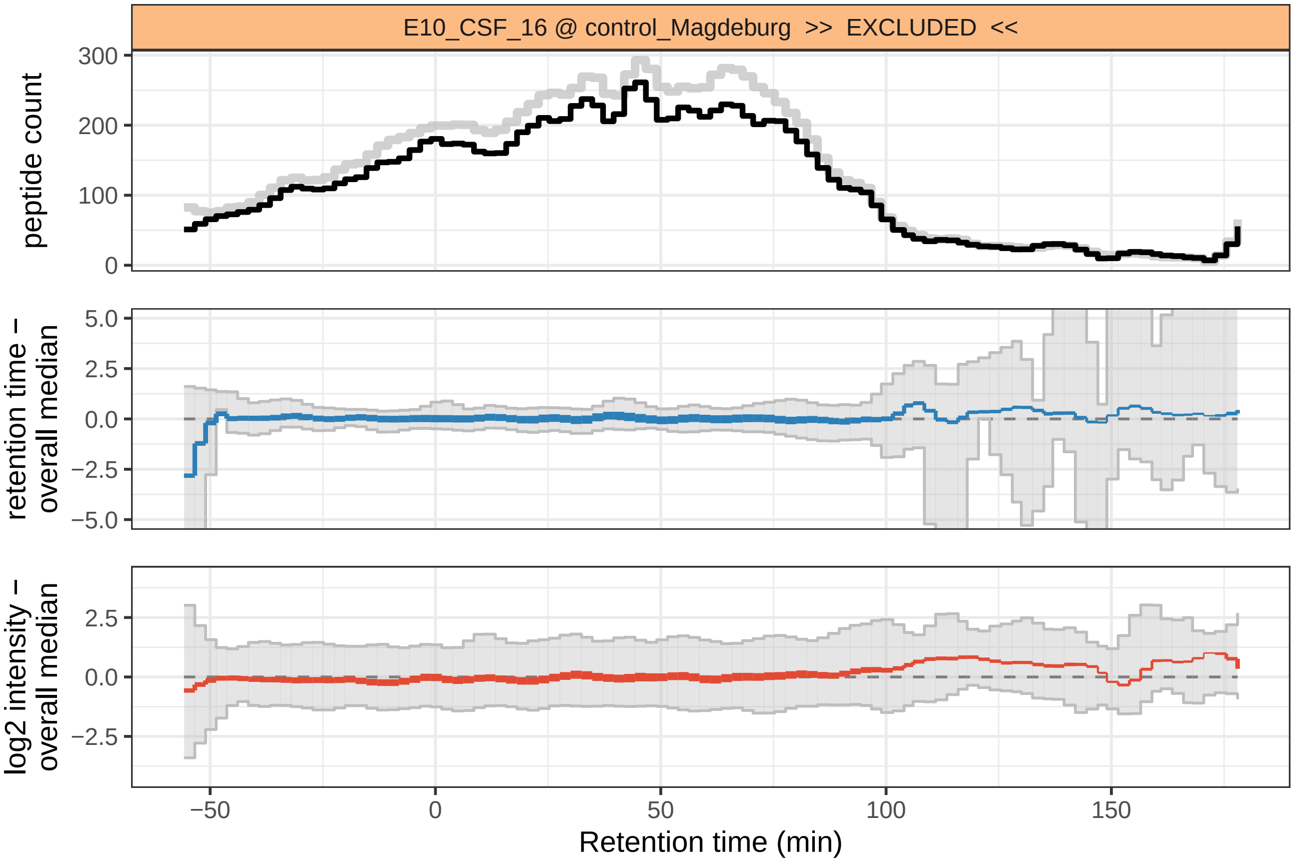
E10_CSF_16 @ control_Magdeburg >> EXCLUDED <<
peptide count
300
200
100
0
retention time –
overall median
5.0
2.5
0.0
−2.5
−5.0
log2 intensity –
overall median
2.5
0.0
−2.5
−50
0
50
100
150
Retention time (min)

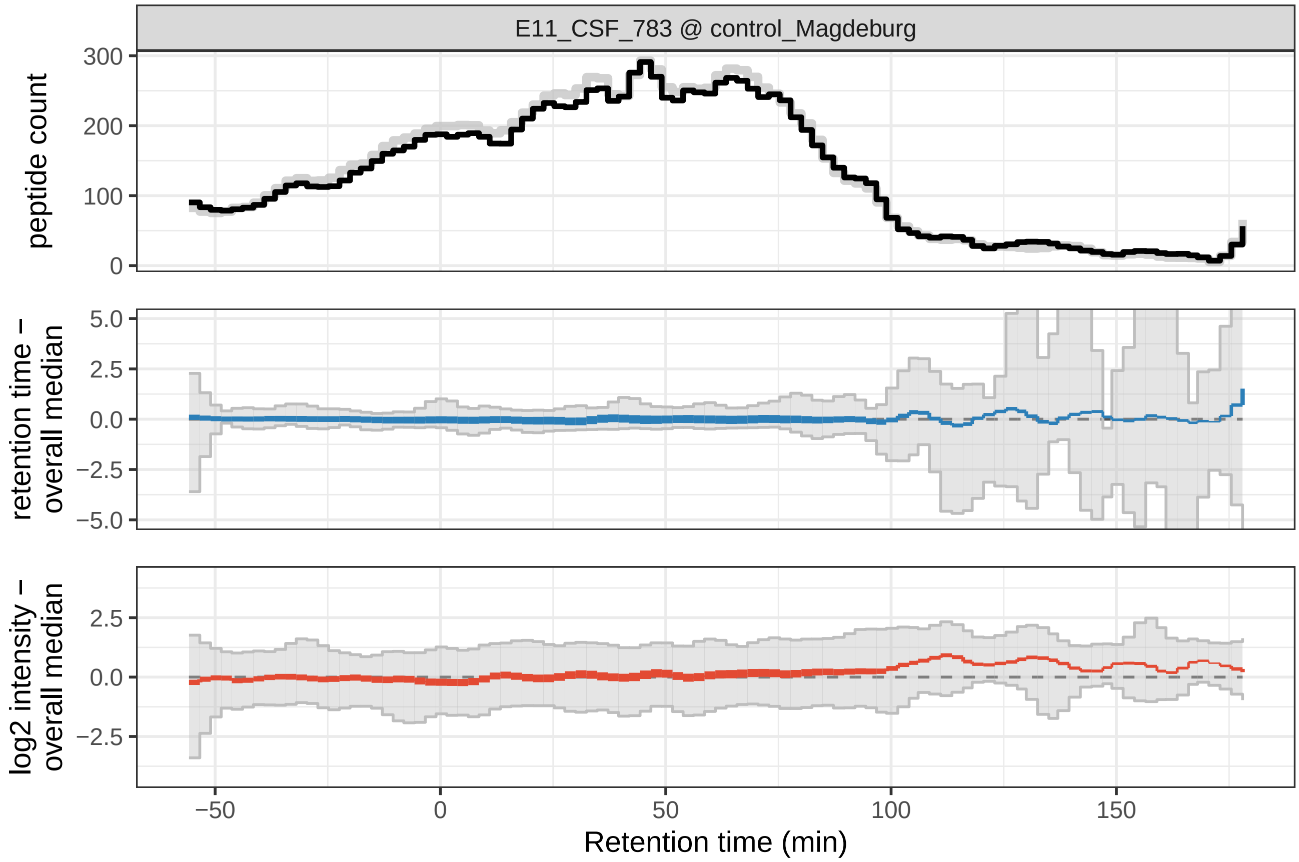
E11_CSF_783 @ control_Magdeburg
peptide count
retention time – overall median
log2 intensity – overall median
Retention time (min)

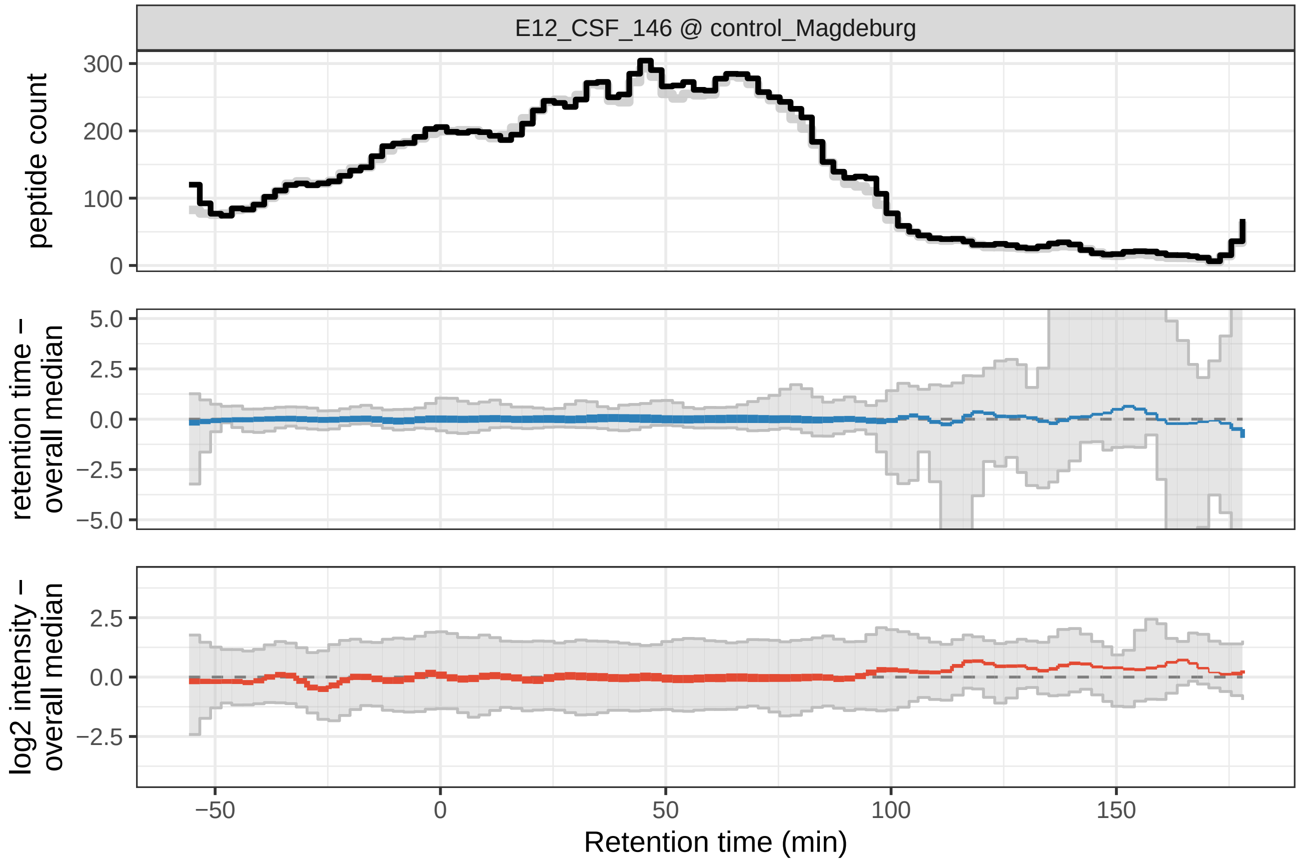
E12_CSF_146 @ control_Magdeburg
peptide count
retention time – overall median
log2 intensity – overall median
Retention time (min)

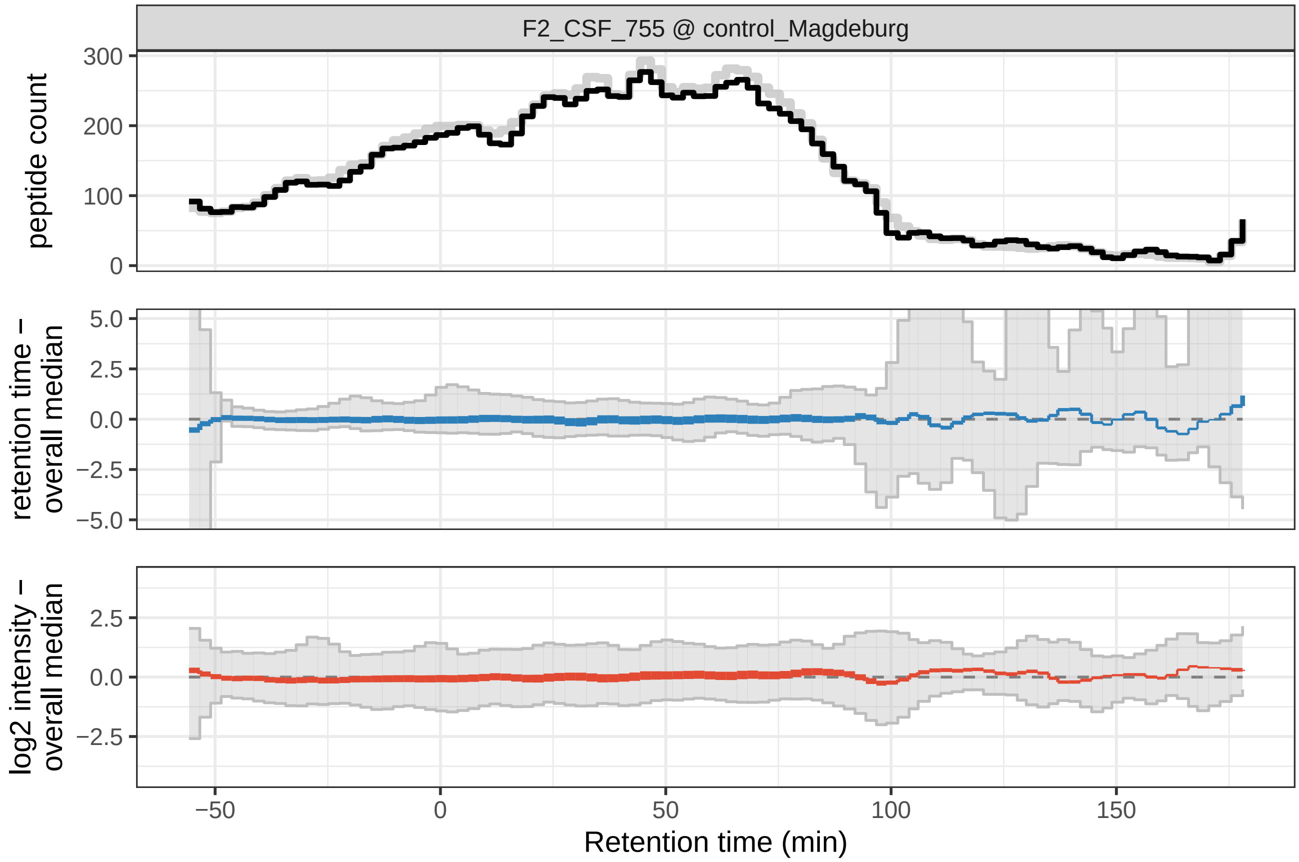
F2_CSF_755 @ control_Magdeburg
peptide count
retention time – overall median
log2 intensity – overall median
Retention time (min)

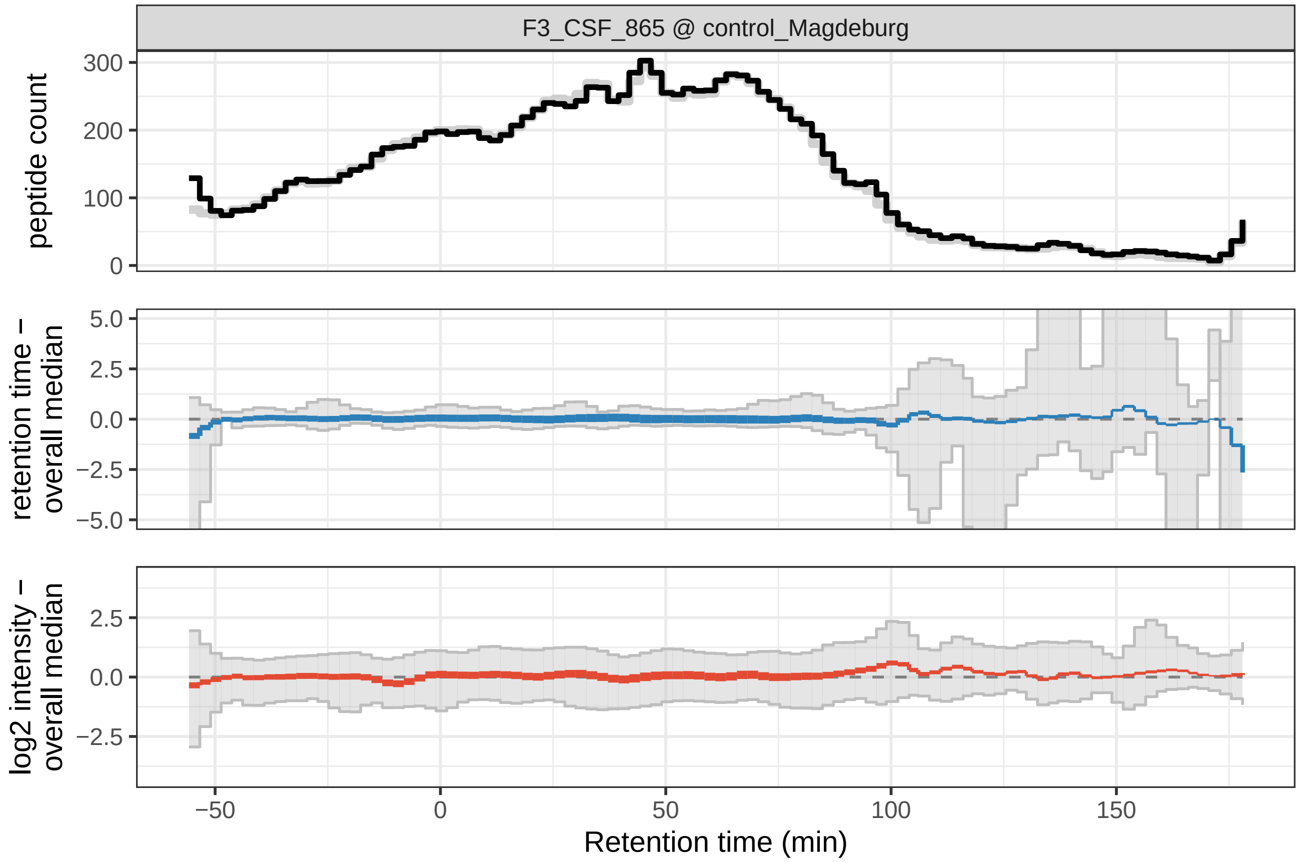
F3_CSF_865 @ control_Magdeburg
peptide count
retention time – overall median
log2 intensity – overall median
Retention time (min)

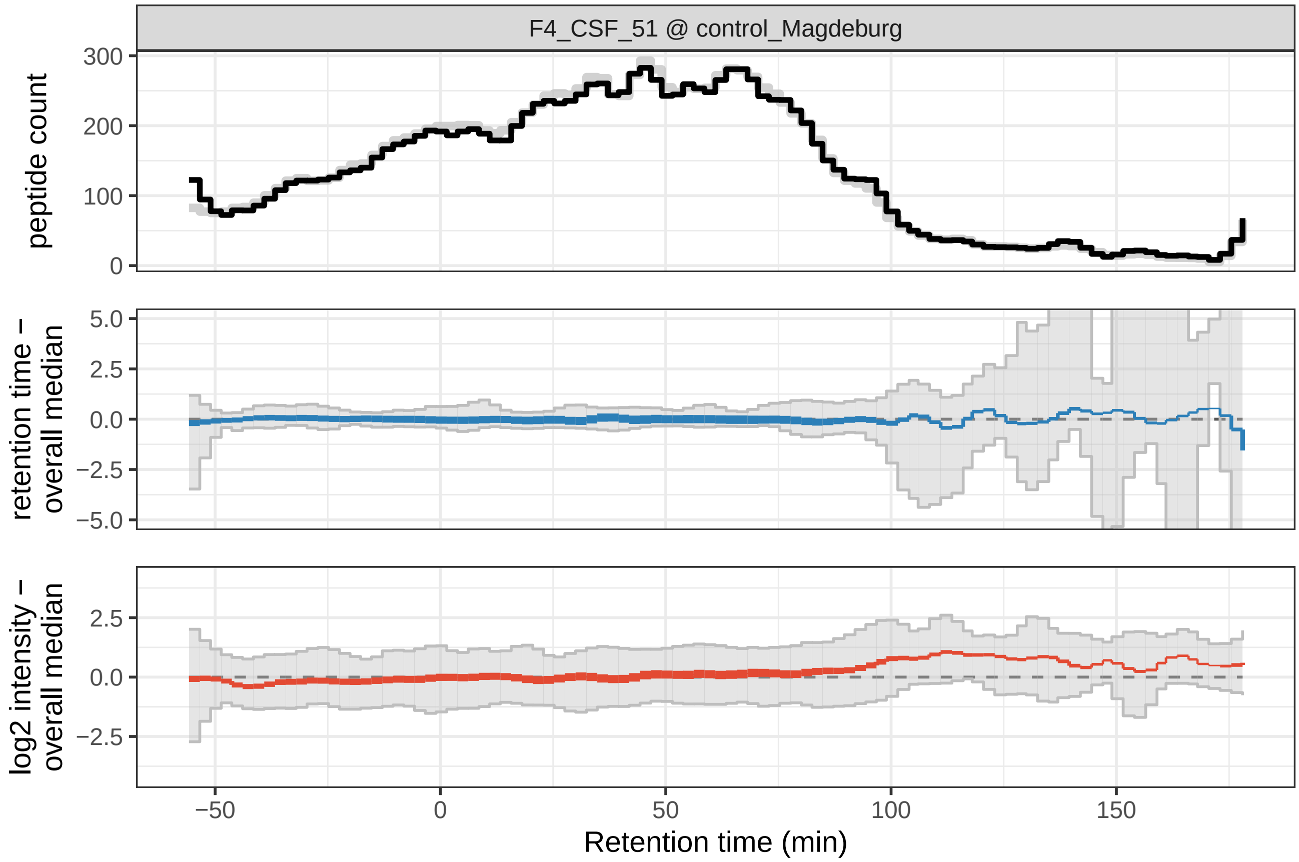
F4_CSF_51 @ control_Magdeburg
peptide count
300
200
100
0
retention time –
overall median
5.0
2.5
0.0
−2.5
−5.0
log2 intensity –
overall median
2.5
0.0
−2.5
−50
0
50
100
150
Retention time (min)

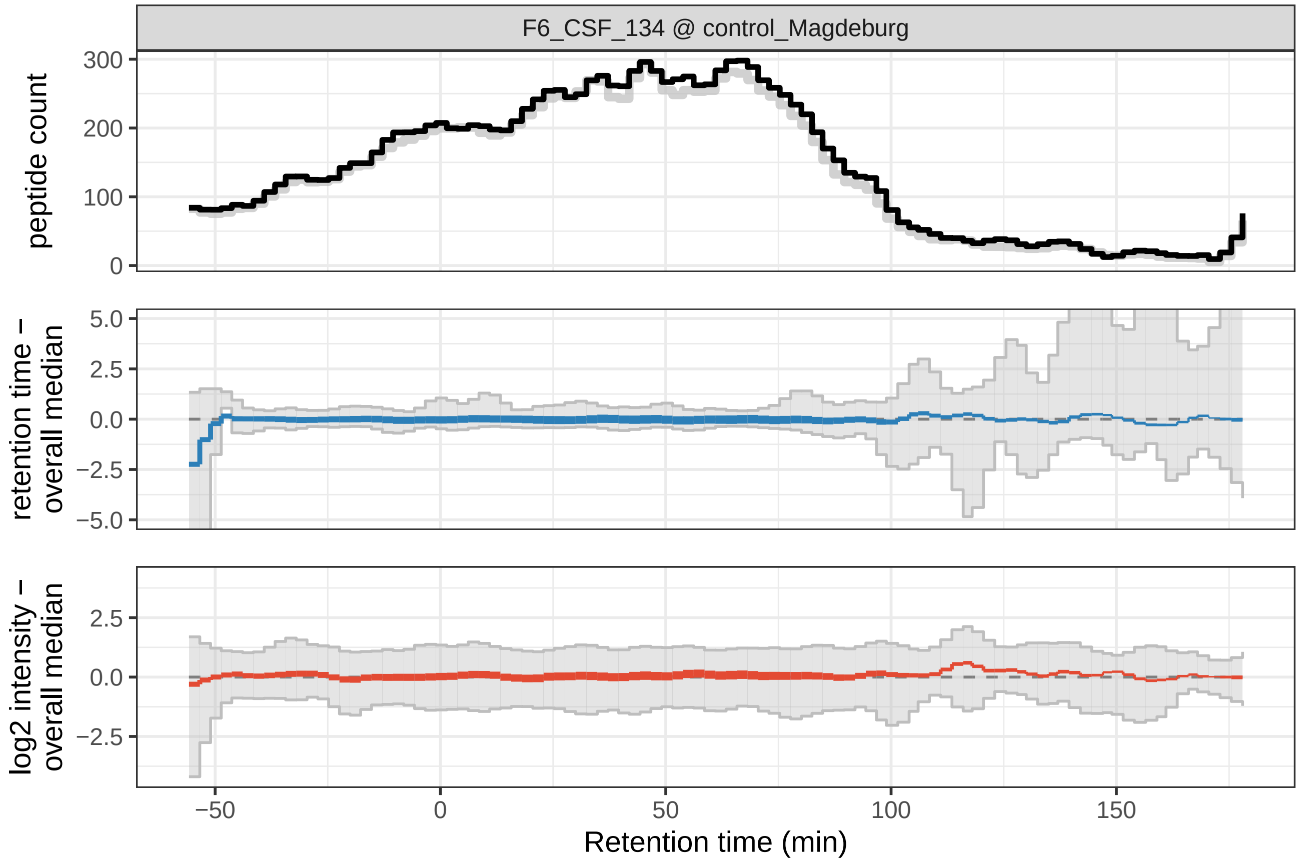
F6_CSF_134 @ control_Magdeburg
peptide count
300
200
100
0
retention time –
overall median
5.0
2.5
0.0
−2.5
−5.0
log2 intensity –
overall median
2.5
0.0
−2.5
−50
0
50
100
150
Retention time (min)

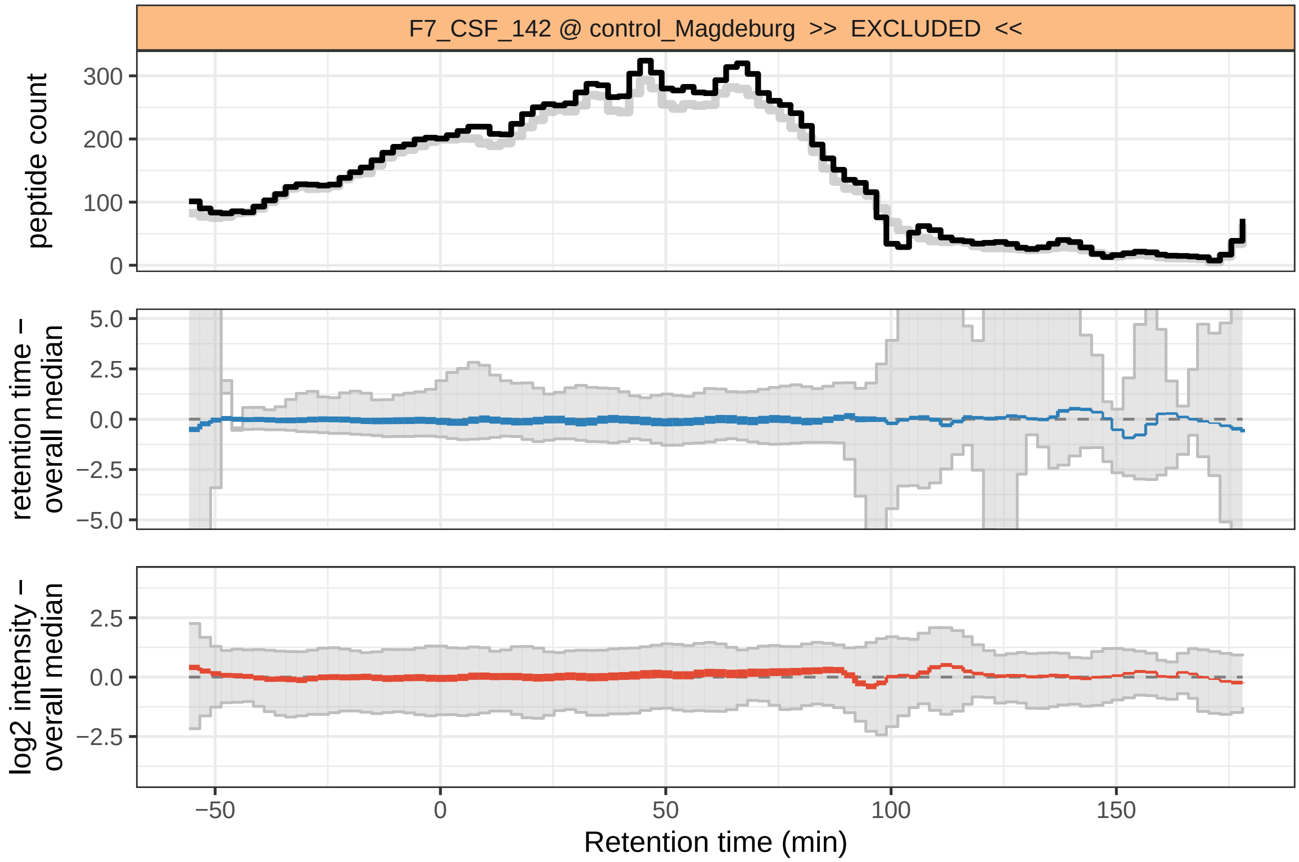
F7_CSF_142 @ control_Magdeburg >> EXCLUDED <<
peptide count
retention time – overall median
log2 intensity – overall median
Retention time (min)

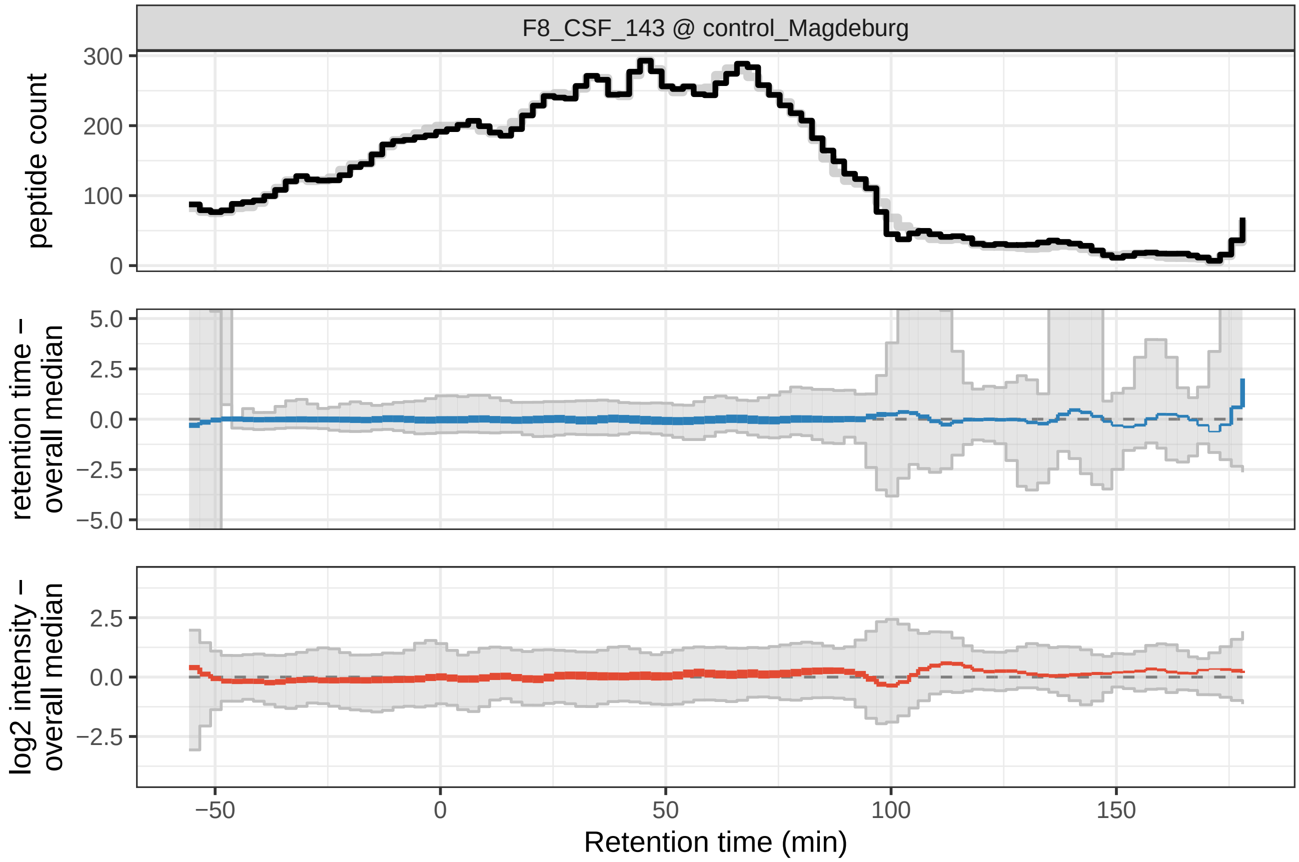
F8_CSF_143 @ control_Magdeburg
peptide count
retention time – overall median
log2 intensity – overall median
Retention time (min)

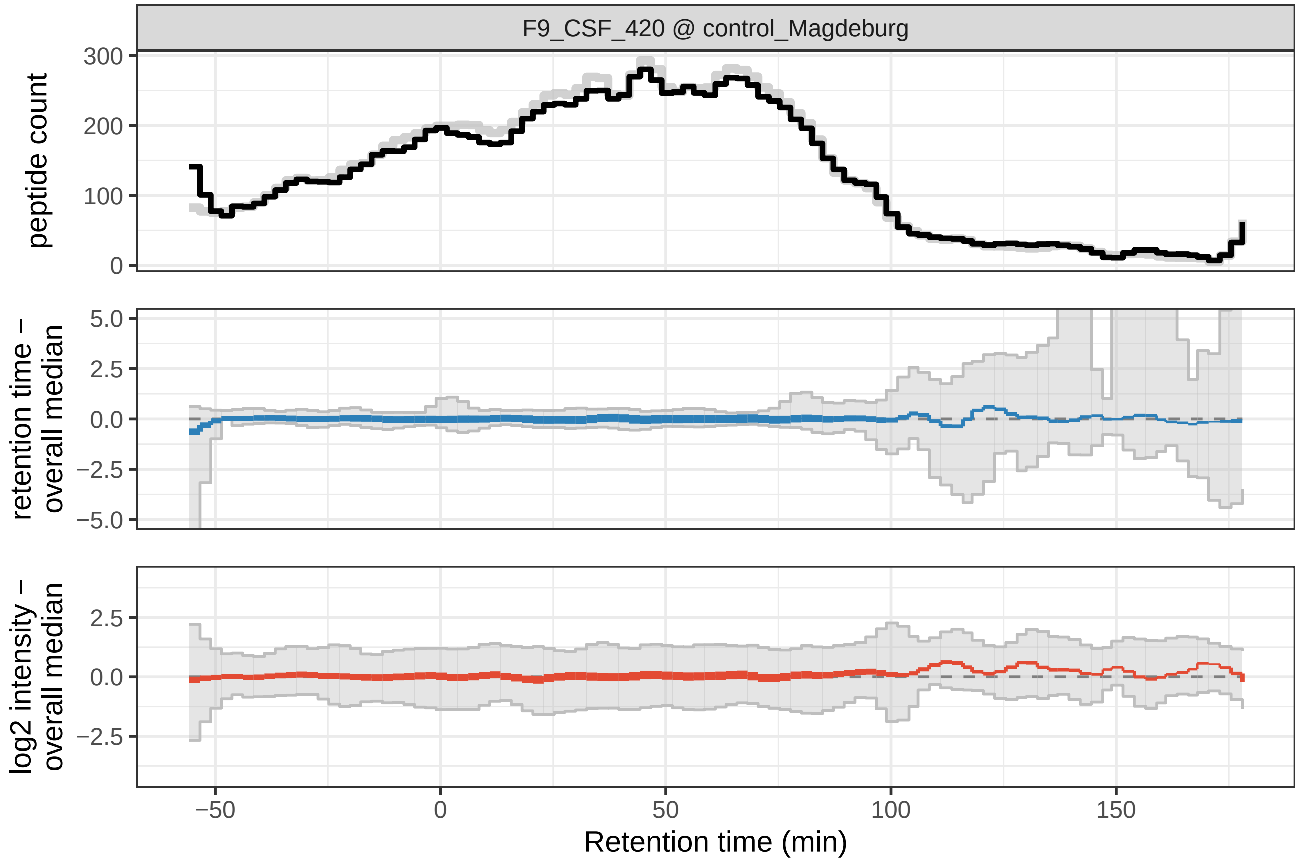
F9_CSF_420 @ control_Magdeburg
peptide count
300
200
100
0
retention time –
overall median
5.0
2.5
0.0
−2.5
−5.0
log2 intensity –
overall median
2.5
0.0
−2.5
−50
0
50
100
150
Retention time (min)

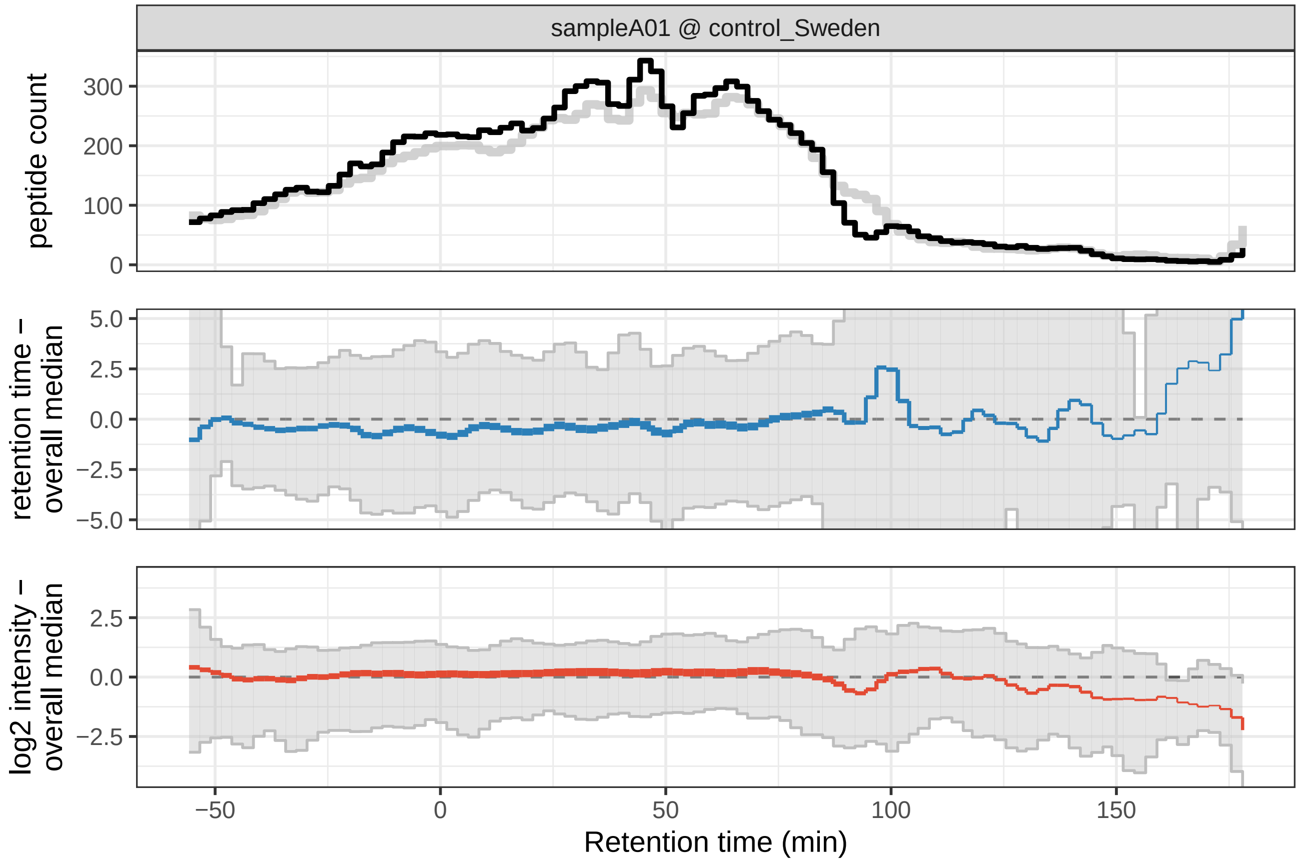
sampleA01 @ control_Sweden
peptide count
300
200
100
0
retention time –
overall median
5.0
2.5
0.0
−2.5
−5.0
log2 intensity –
overall median
2.5
0.0
−2.5
−50
0
50
100
150
Retention time (min)

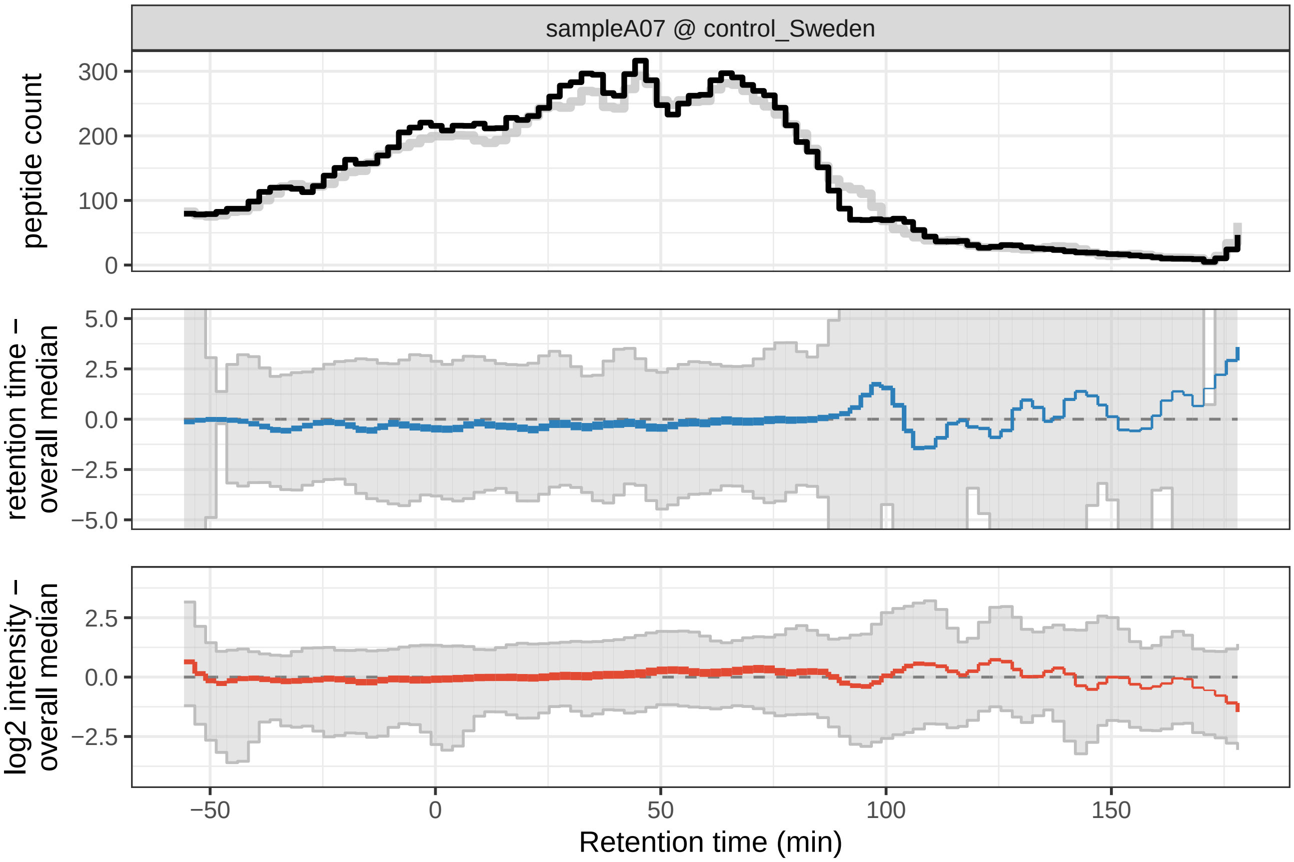
sampleA07 @ control_Sweden
peptide count
retention time –
overall median
log2 intensity –
overall median
Retention time (min)

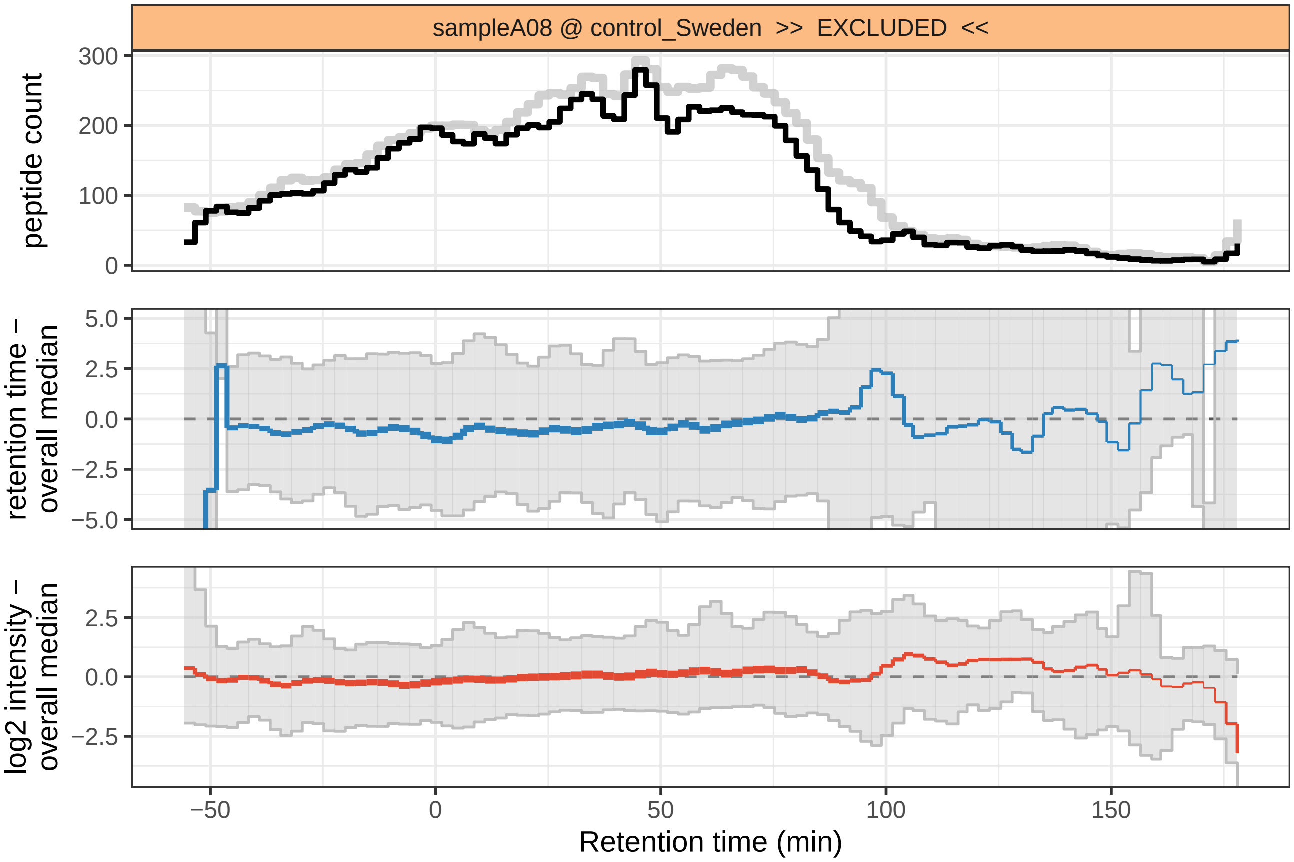
sampleA08 @ control_Sweden >> EXCLUDED <<
peptide count
retention time –
overall median
log2 intensity –
overall median
Retention time (min)

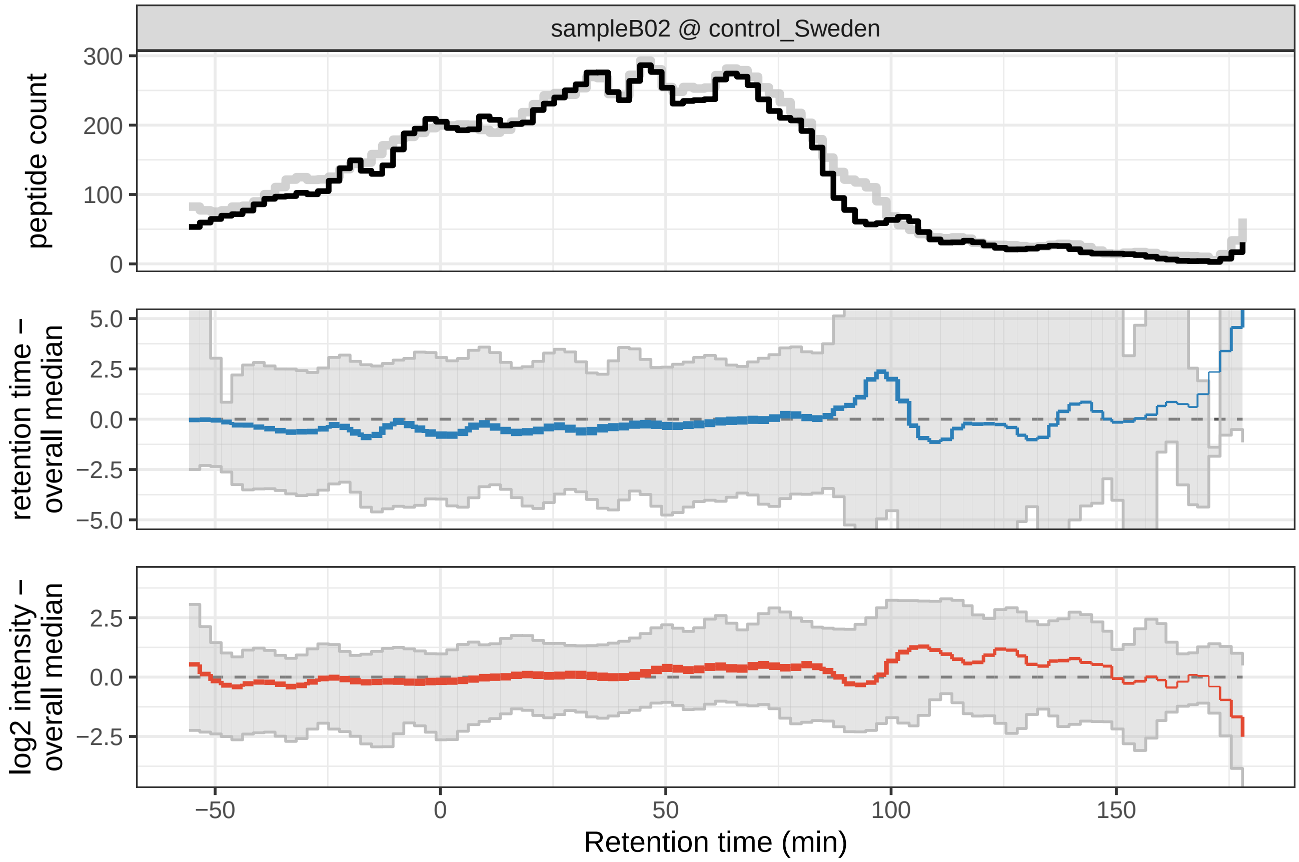
sampleB02 @ control_Sweden
peptide count
300
200
100
0
retention time –
overall median
5.0
2.5
0.0
−2.5
−5.0
log2 intensity –
overall median
2.5
0.0
−2.5
−50
0
50
100
150
Retention time (min)

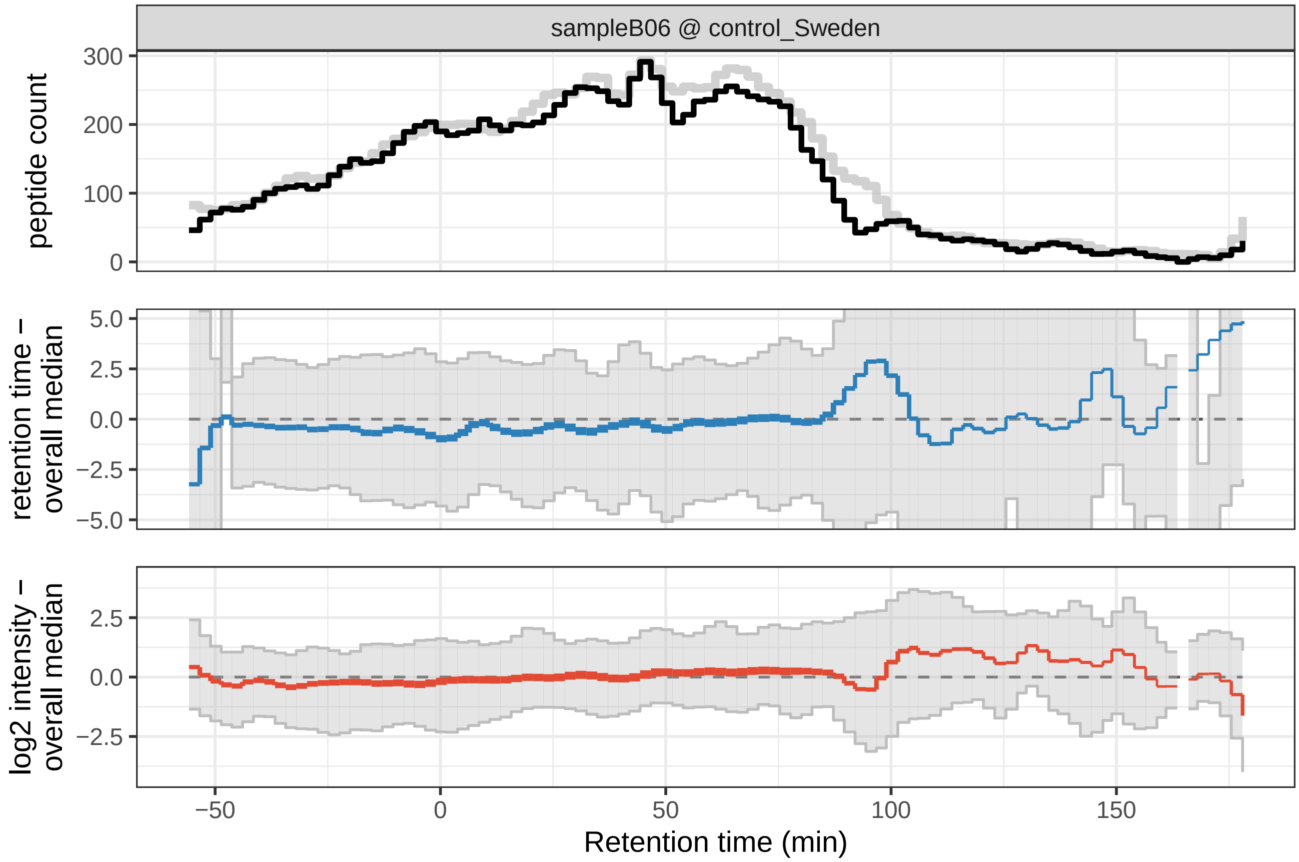
sampleB06 @ control_Sweden
peptide count
300
200
100
0
retention time –
overall median
5.0
2.5
0.0
−2.5
−5.0
log2 intensity –
overall median
2.5
0.0
−2.5
−50
0
50
100
150
Retention time (min)

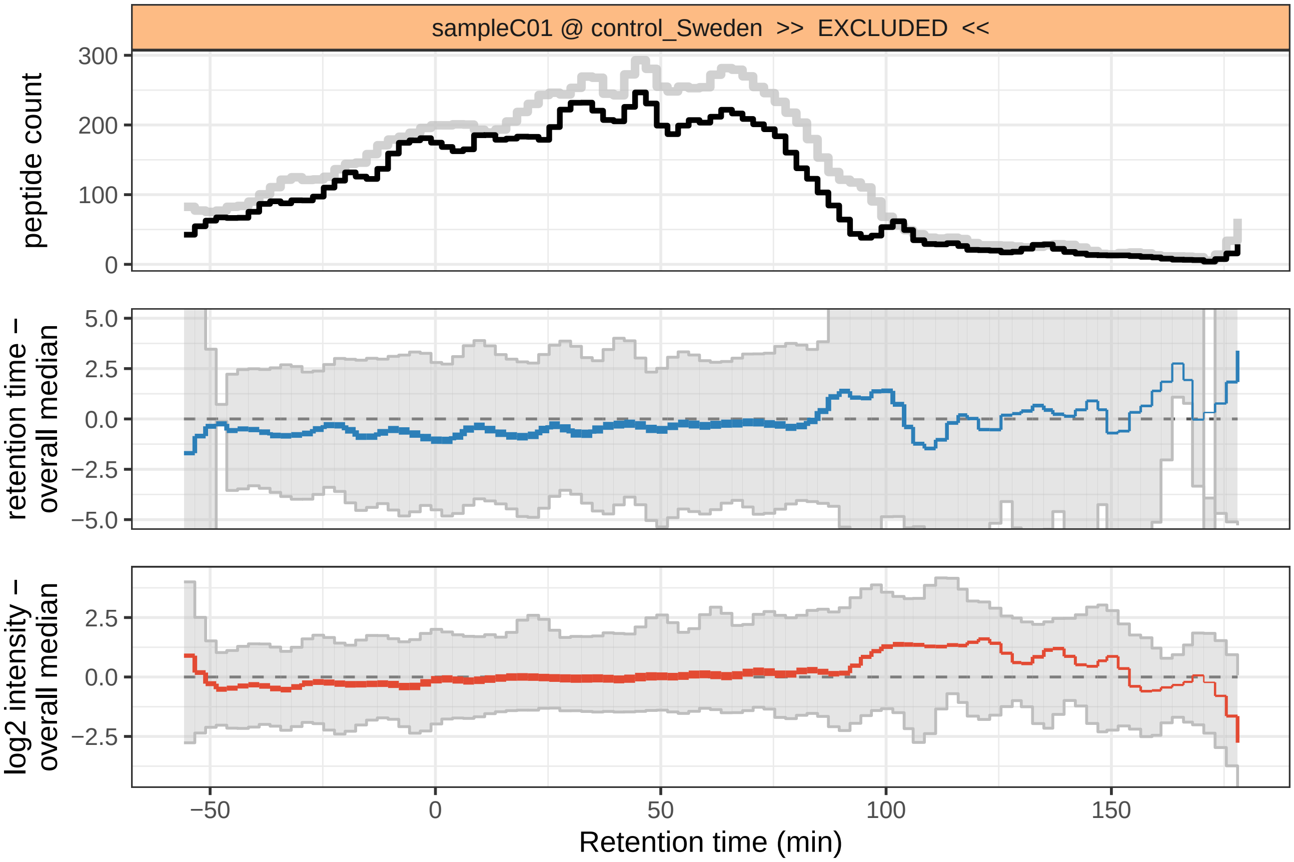
sampleC01 @ control_Sweden >> EXCLUDED <<
peptide count
retention time – overall median
log2 intensity – overall median
Retention time (min)

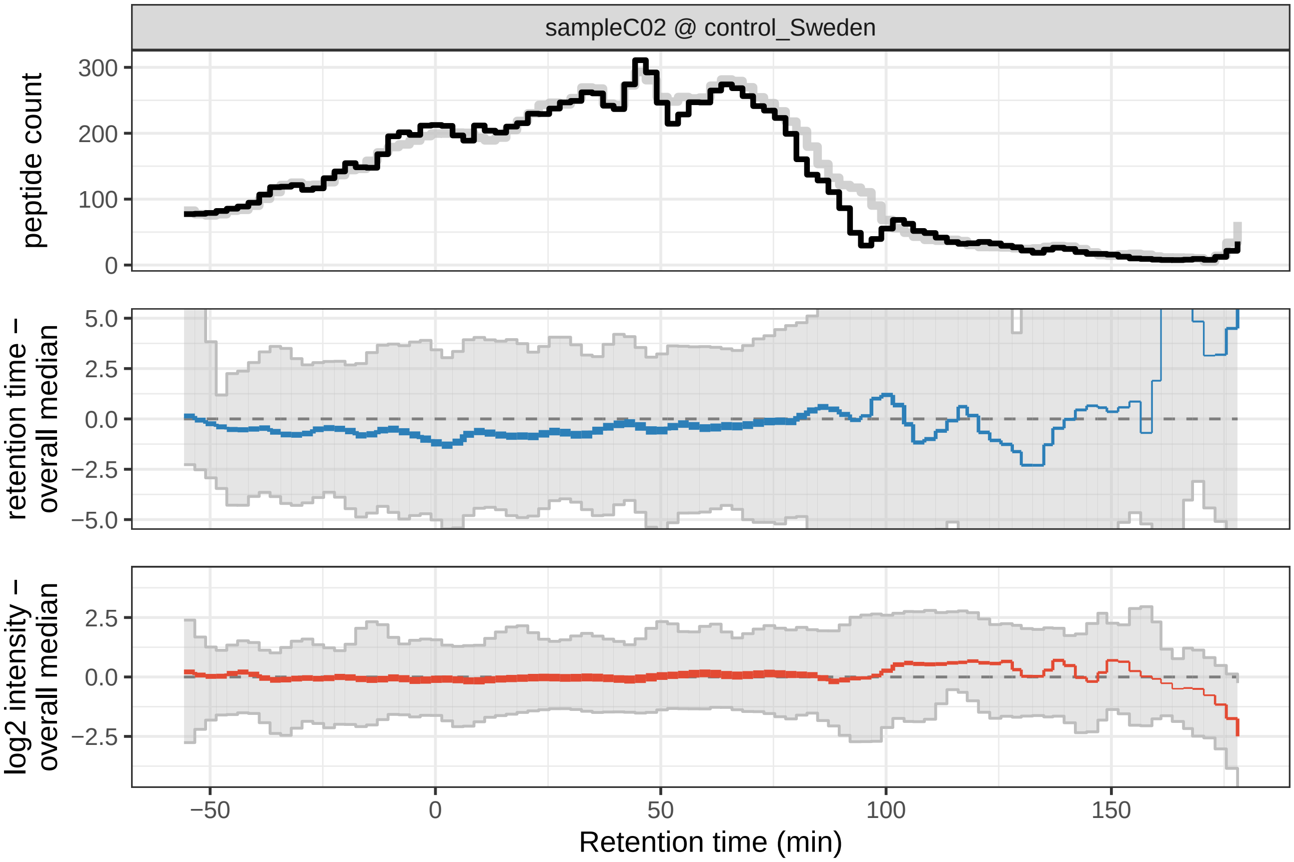
sampleC02 @ control_Sweden
peptide count
retention time – overall median
log2 intensity – overall median
Retention time (min)

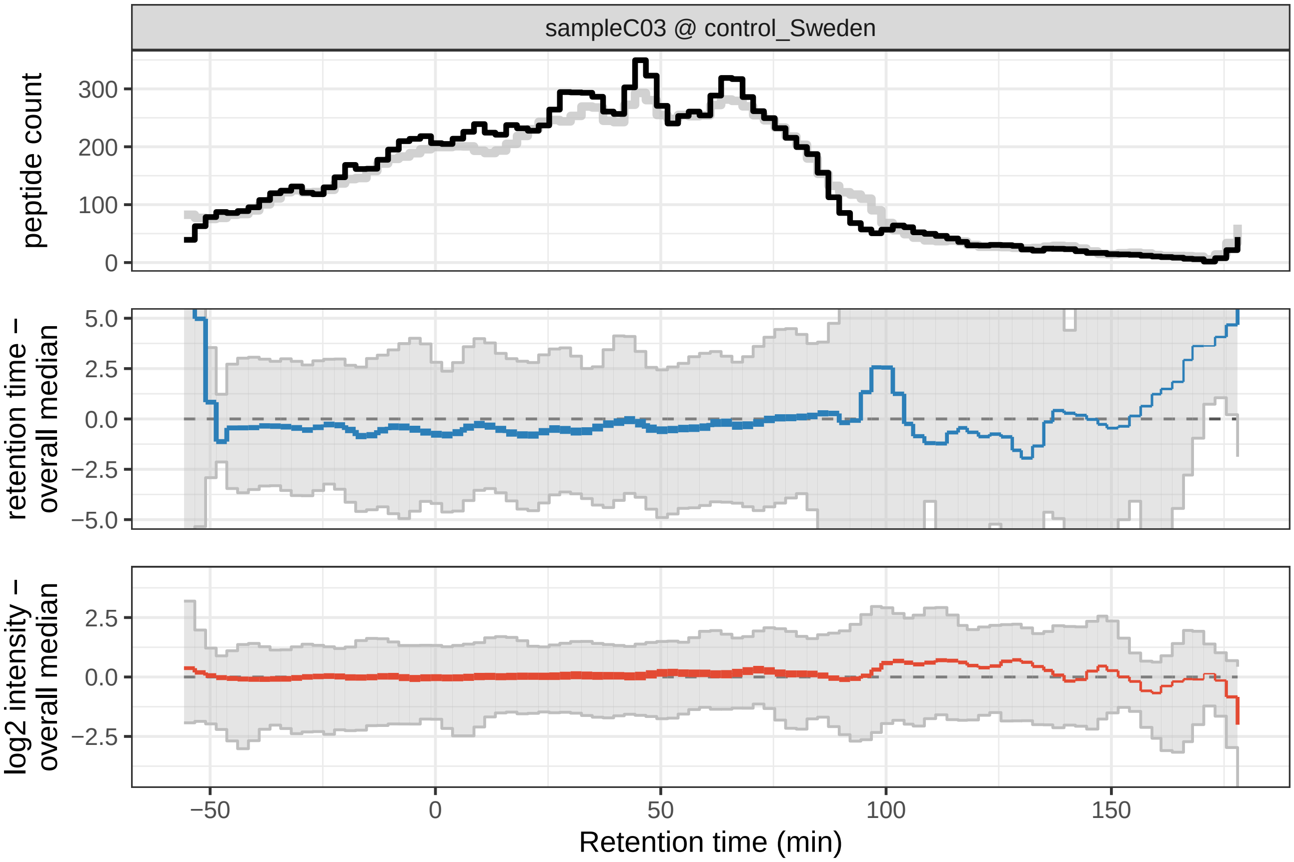
sampleC03 @ control_Sweden
peptide count
300
200
100
0
retention time –
overall median
5.0
2.5
0.0
−2.5
−5.0
log2 intensity –
overall median
2.5
0.0
−2.5
−50
0
50
100
150
Retention time (min)

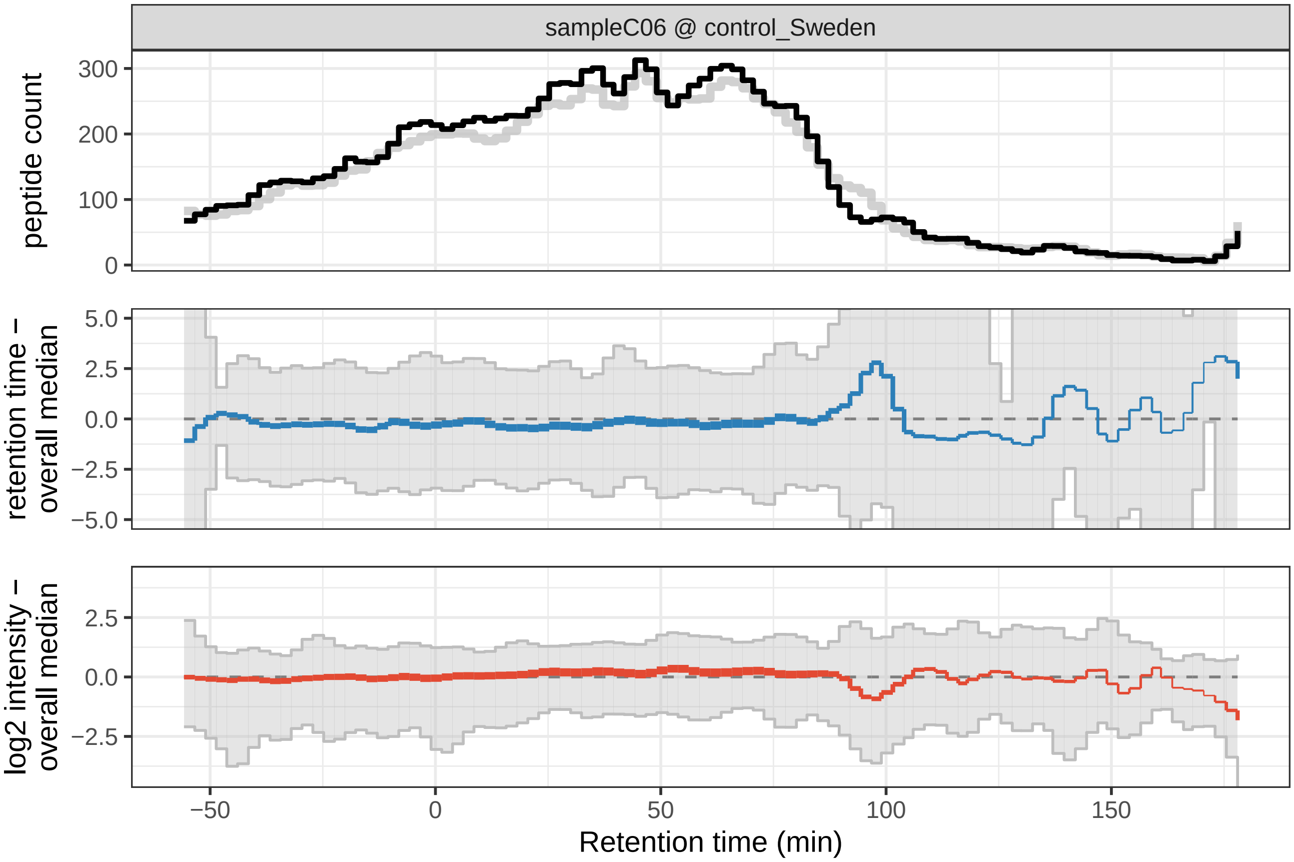
sampleC06 @ control_Sweden
peptide count
300
200
100
0
retention time –
overall median
5.0
2.5
0.0
−2.5
−5.0
log2 intensity –
overall median
2.5
0.0
−2.5
−50
0
50
100
150
Retention time (min)

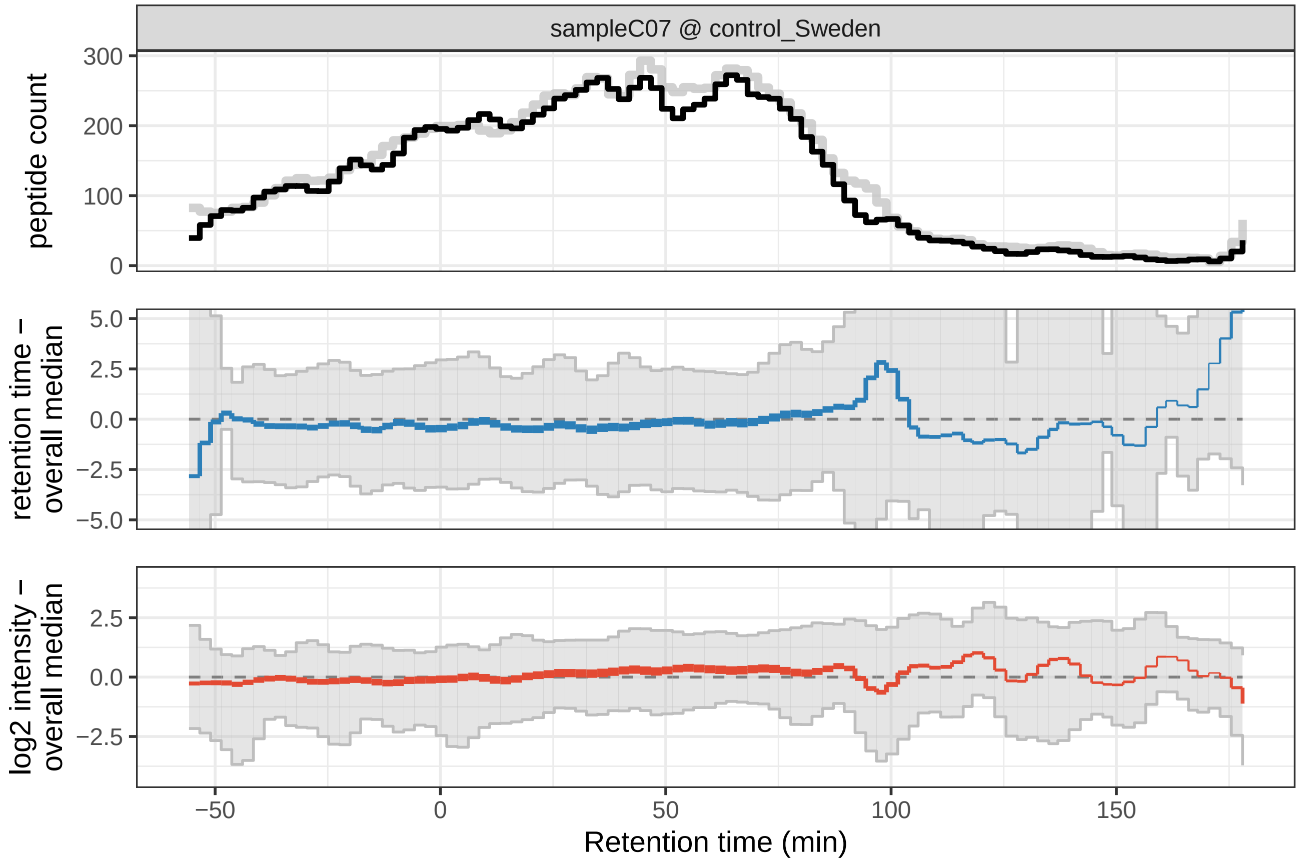
sampleC07 @ control_Sweden
peptide count
retention time – overall median
log2 intensity – overall median
Retention time (min)

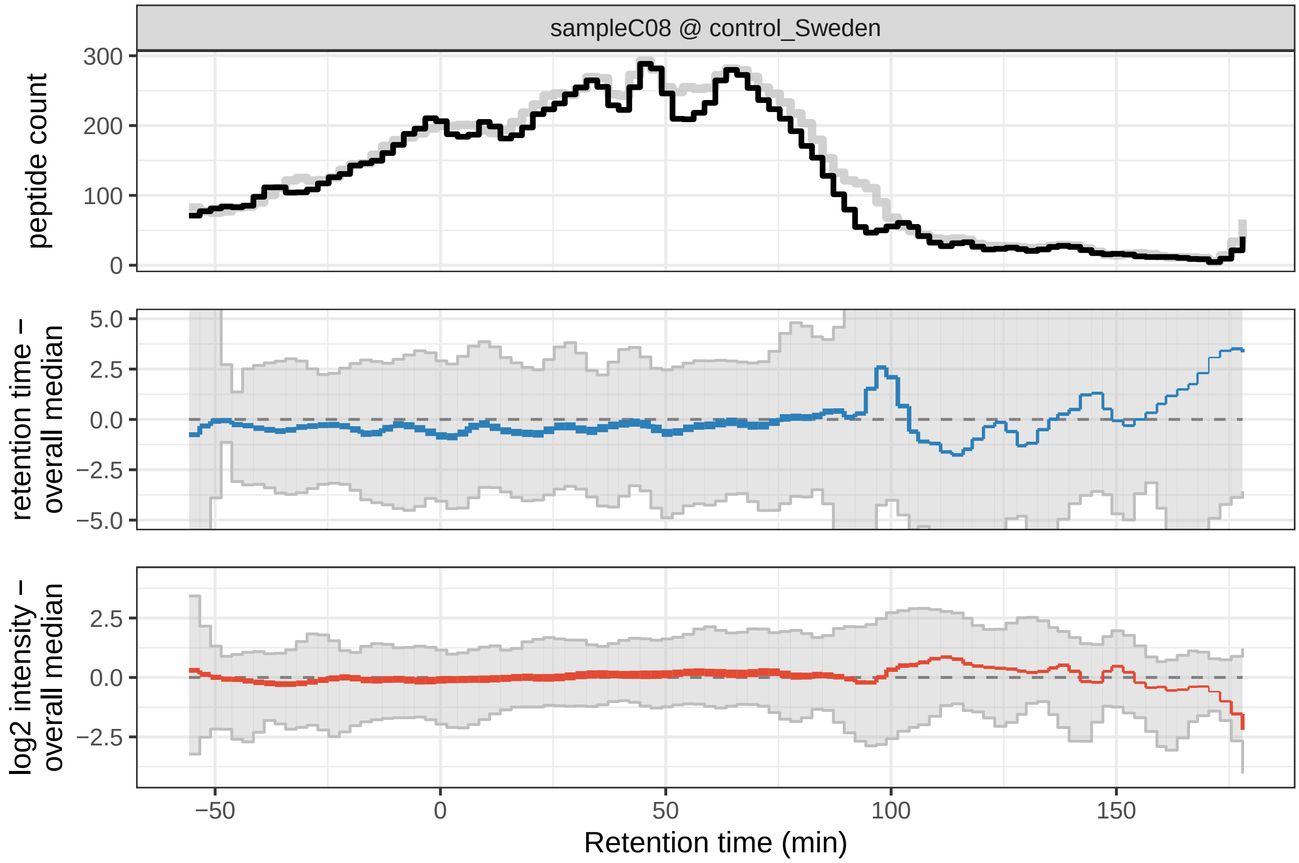
sampleC08 @ control_Sweden
peptide count
retention time – overall median
log2 intensity – overall median
Retention time (min)

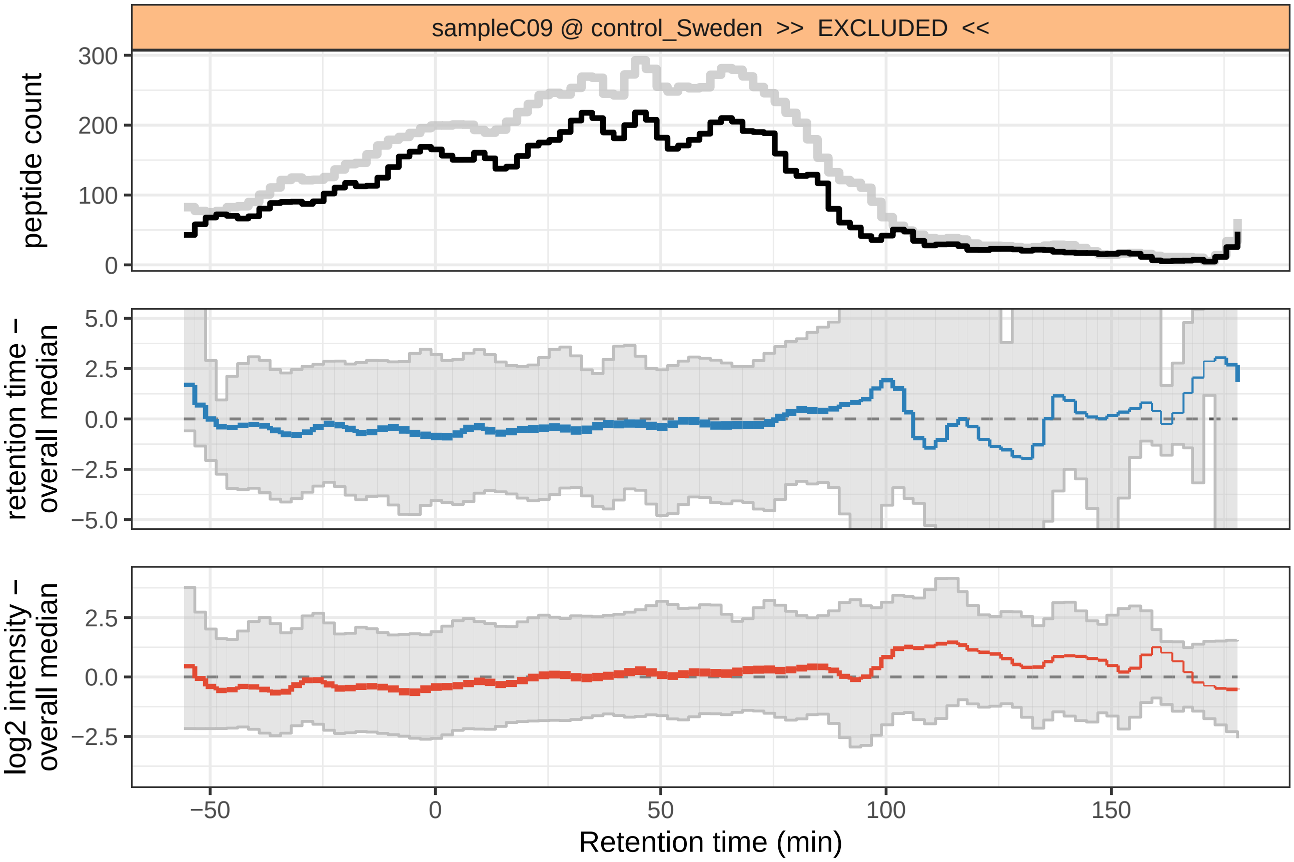
sampleC09 @ control_Sweden >> EXCLUDED <<
peptide count
retention time – overall median
log2 intensity – overall median
Retention time (min)

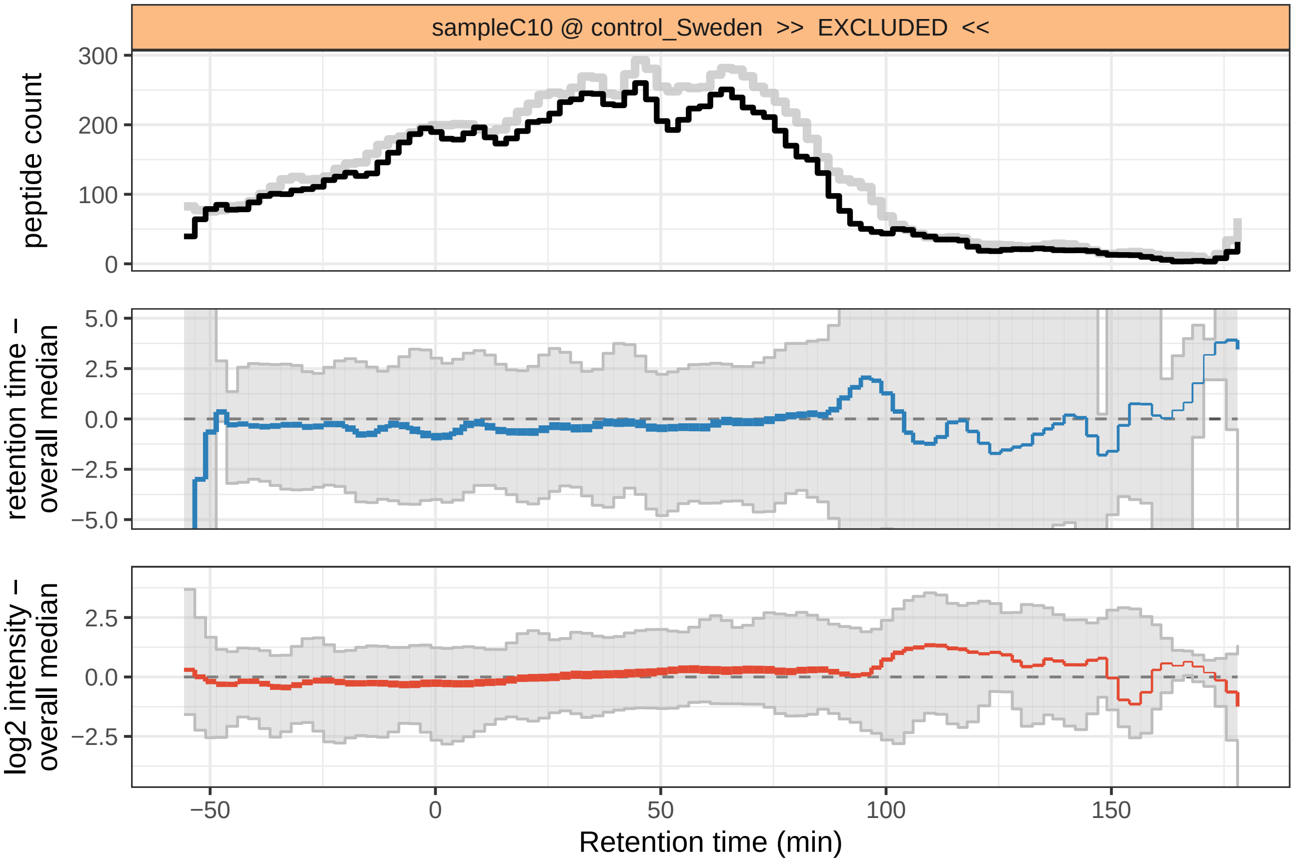
sampleC10 @ control_Sweden >> EXCLUDED <<
peptide count
retention time – overall median
log2 intensity – overall median
Retention time (min)

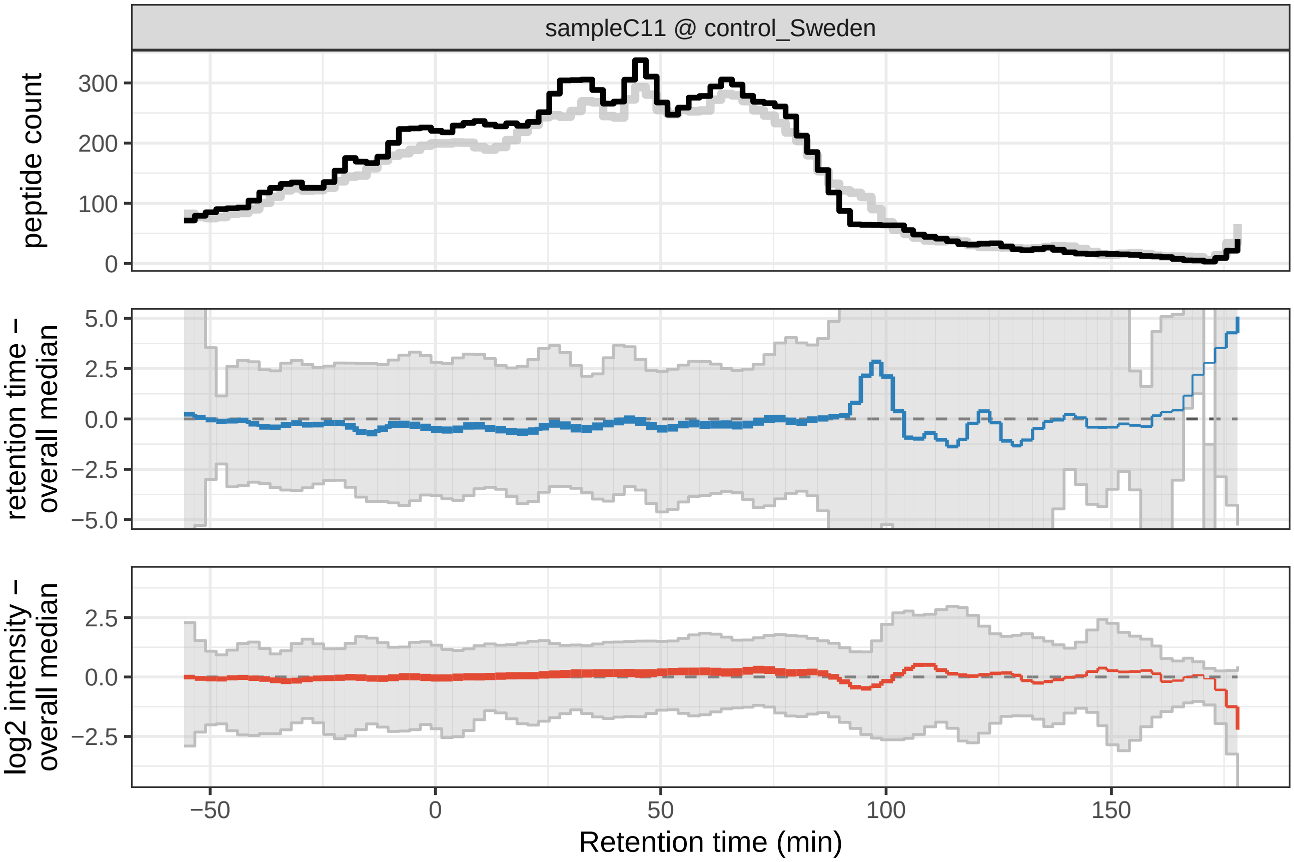
sampleC11 @ control_Sweden
peptide count
300
200
100
0
retention time –
overall median
5.0
2.5
0.0
−2.5
−5.0
log2 intensity –
overall median
2.5
0.0
−2.5
−50
0
50
100
150
Retention time (min)

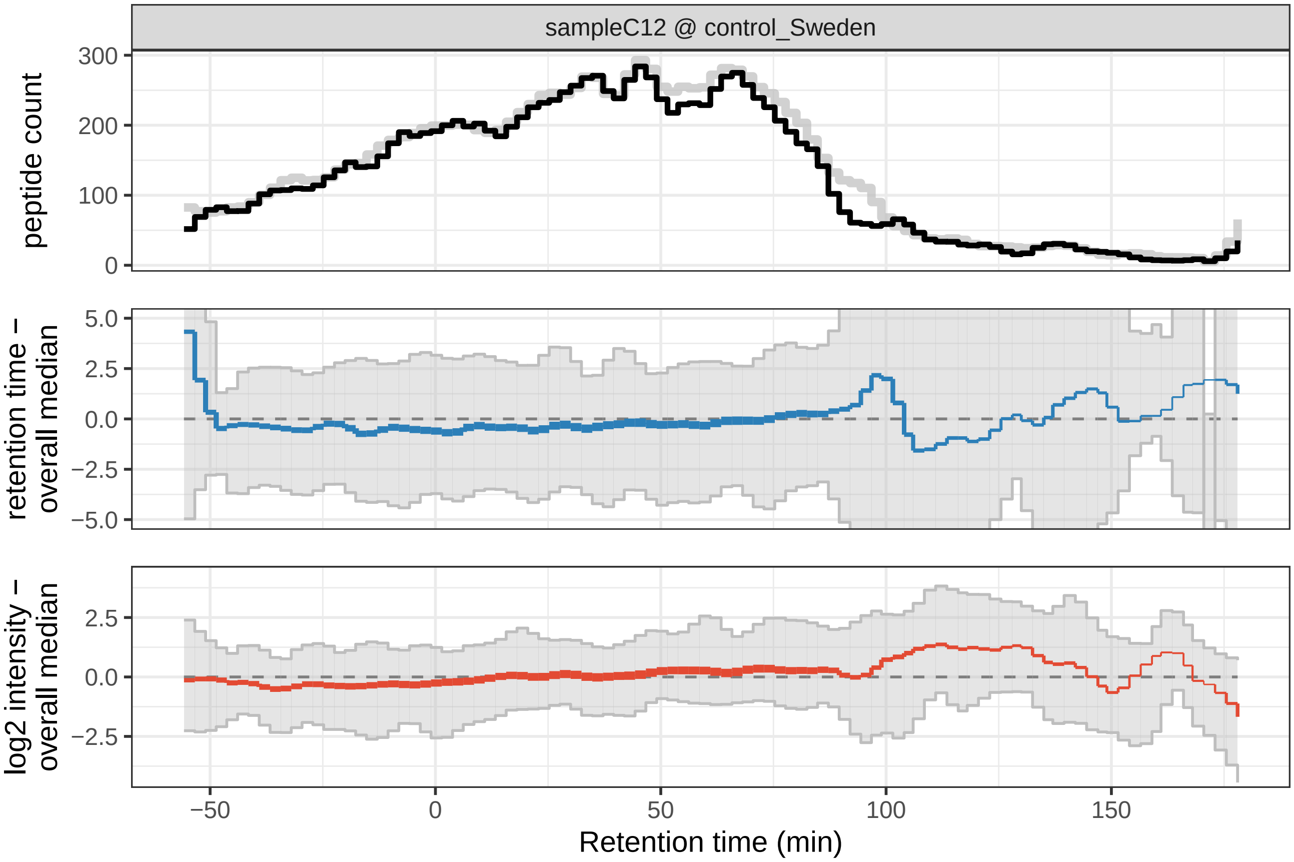
sampleC12 @ control_Sweden
peptide count
300
200
100
0
retention time –
overall median
5.0
2.5
0.0
−2.5
−5.0
log2 intensity –
overall median
2.5
0.0
−2.5
−50
0
50
100
150
Retention time (min)

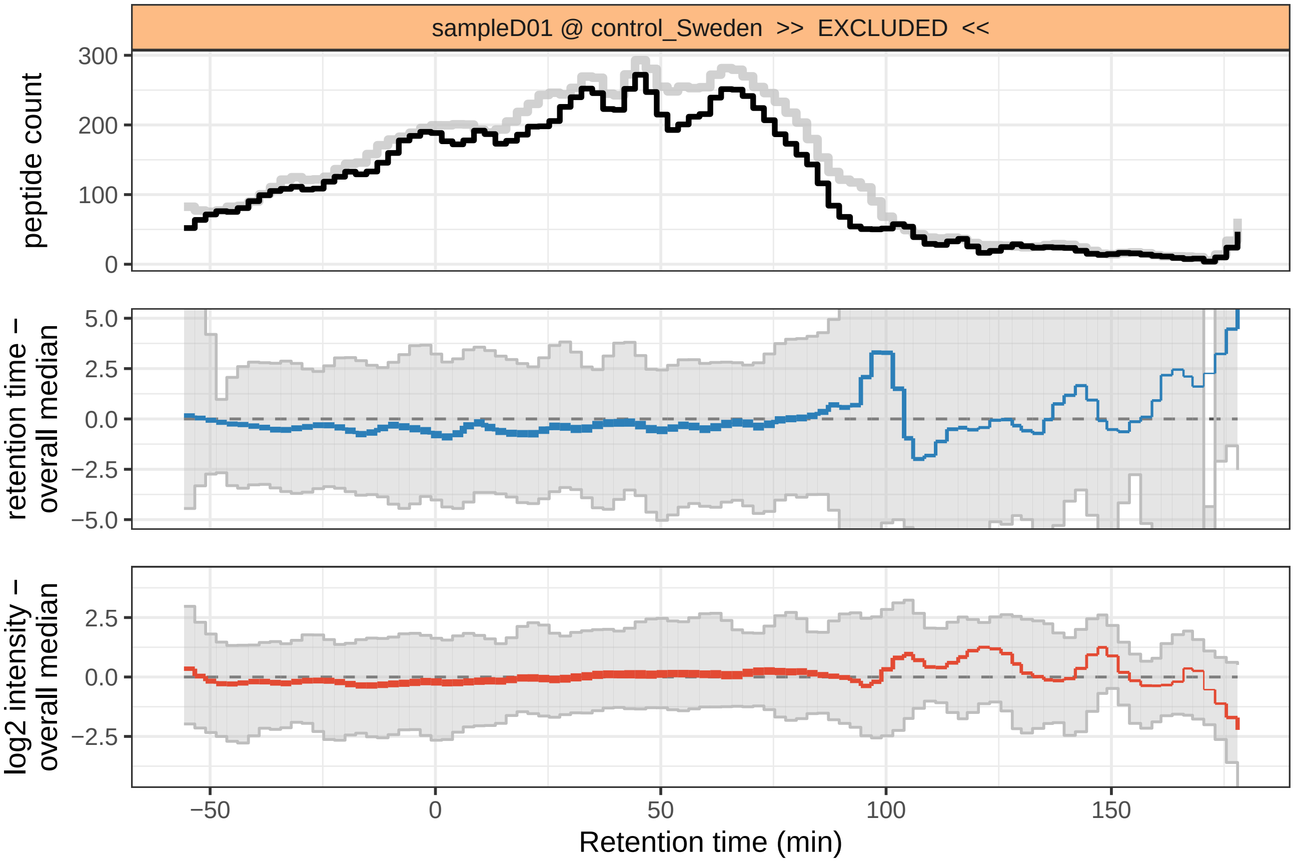
sampleD01 @ control_Sweden >> EXCLUDED <<
peptide count
retention time – overall median
log2 intensity – overall median
Retention time (min)

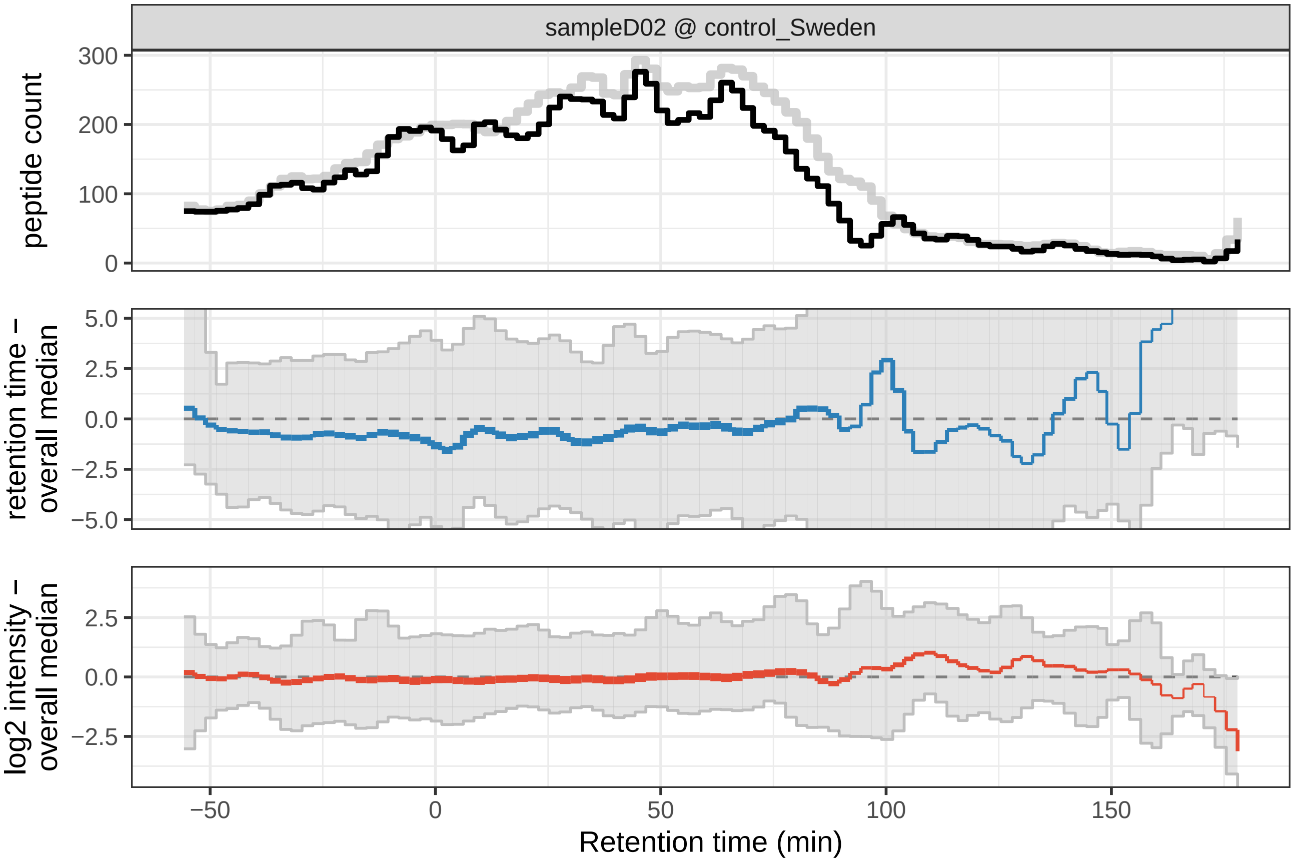
sampleD02 @ control_Sweden
peptide count
retention time – overall median
log2 intensity – overall median
Retention time (min)

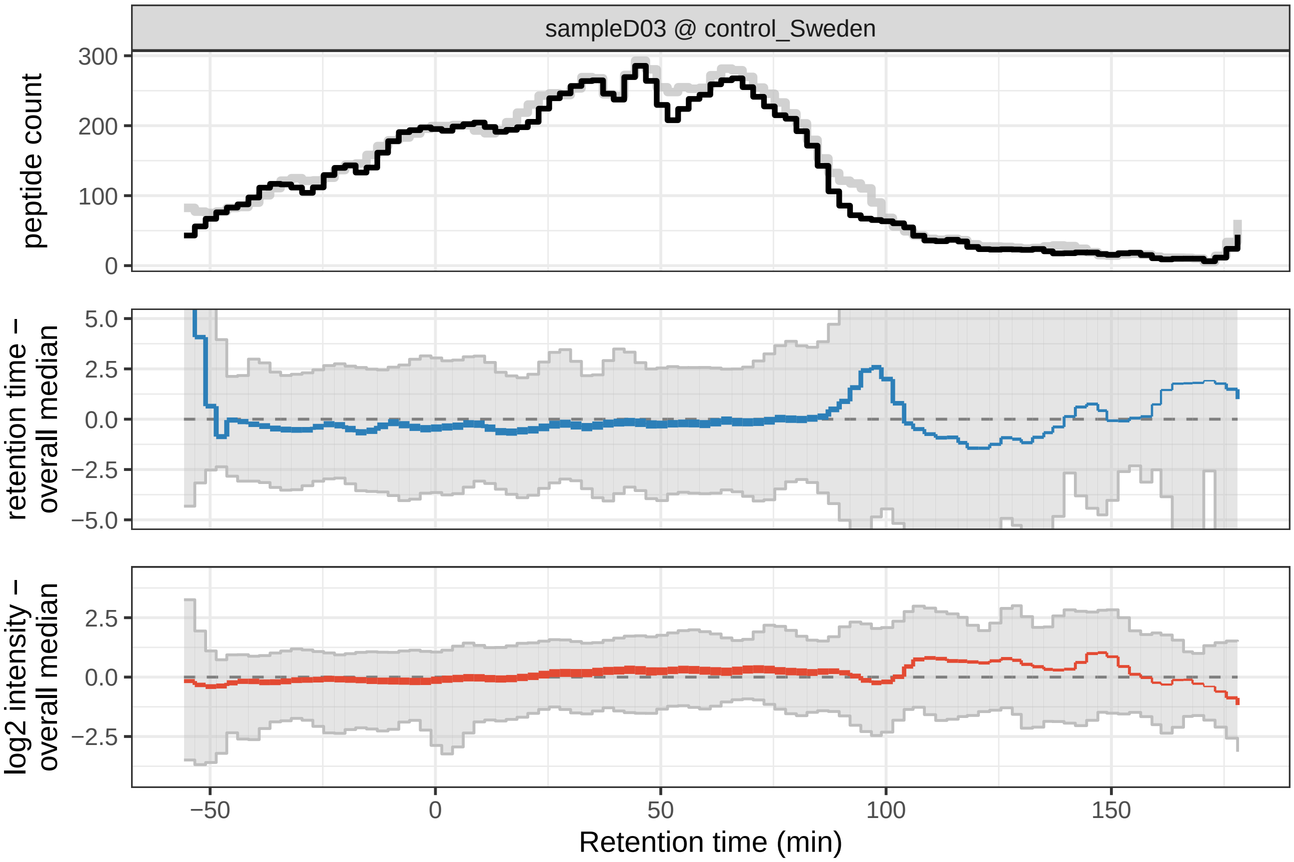
sampleD03 @ control_Sweden
peptide count
300
200
100
0
retention time –
overall median
5.0
2.5
0.0
−2.5
−5.0
log2 intensity –
overall median
2.5
0.0
−2.5
−50
0
50
100
150
Retention time (min)

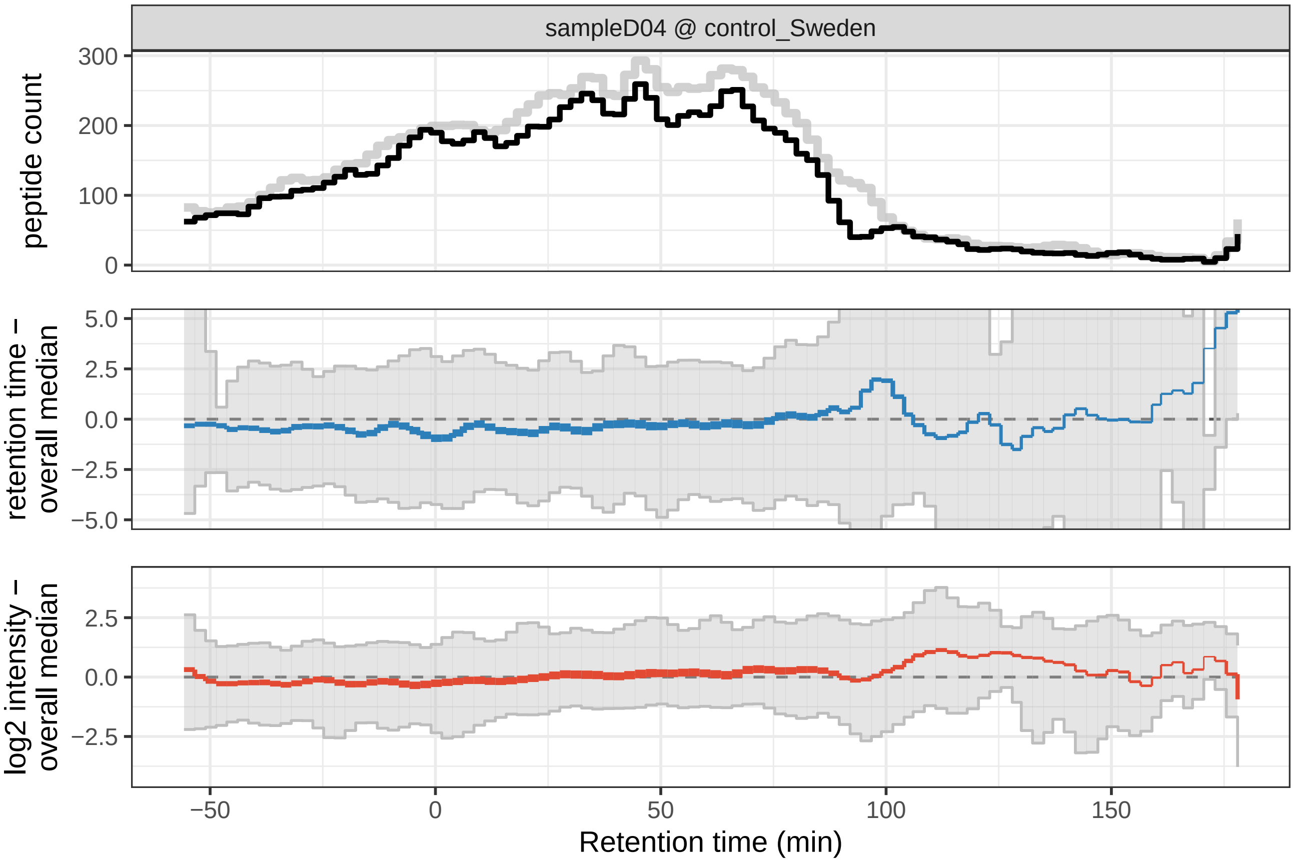
sampleD04 @ control_Sweden
peptide count
300
200
100
0
retention time –
overall median
5.0
2.5
0.0
−2.5
−5.0
log2 intensity –
overall median
2.5
0.0
−2.5
−50
0
50
100
150
Retention time (min)

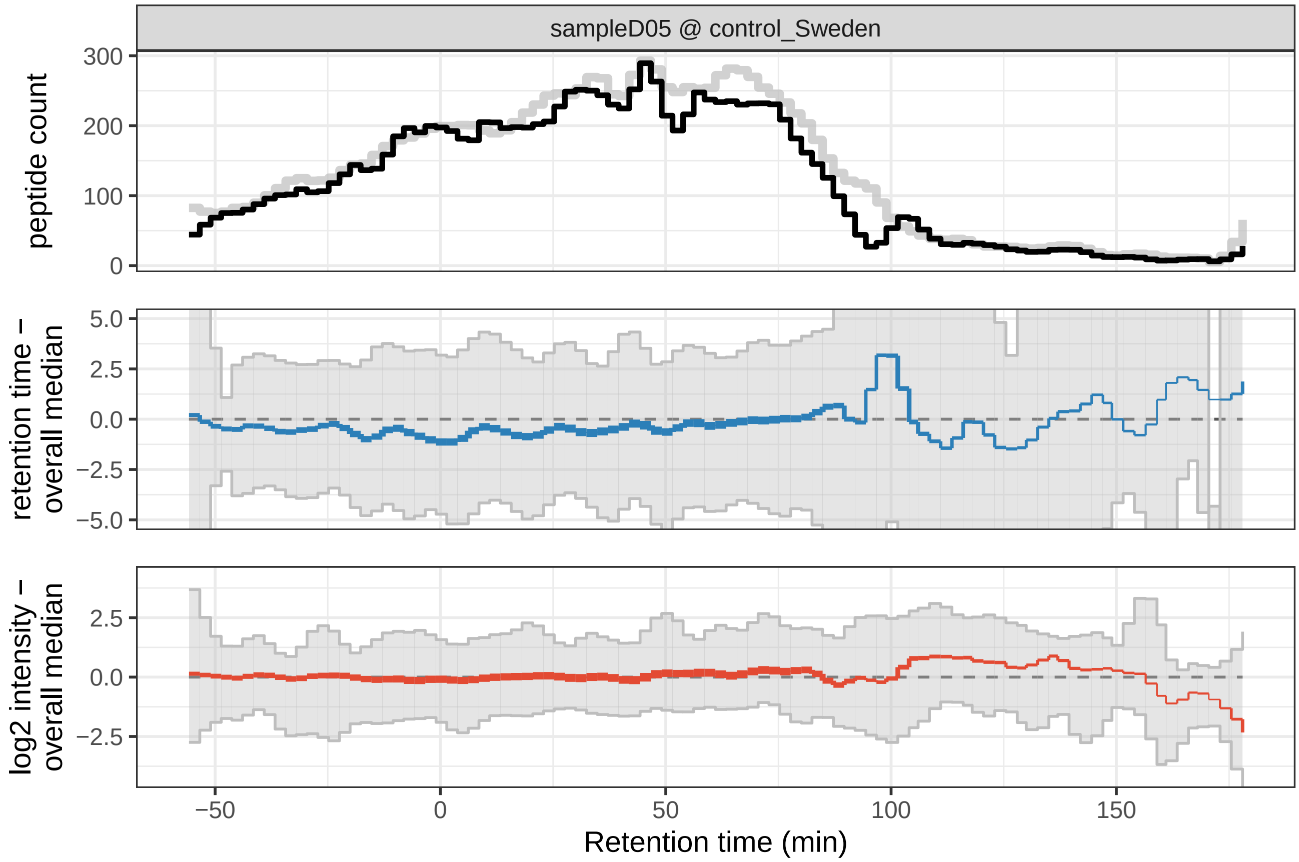
sampleD05 @ control_Sweden
peptide count
retention time –
overall median
log2 intensity –
overall median
Retention time (min)

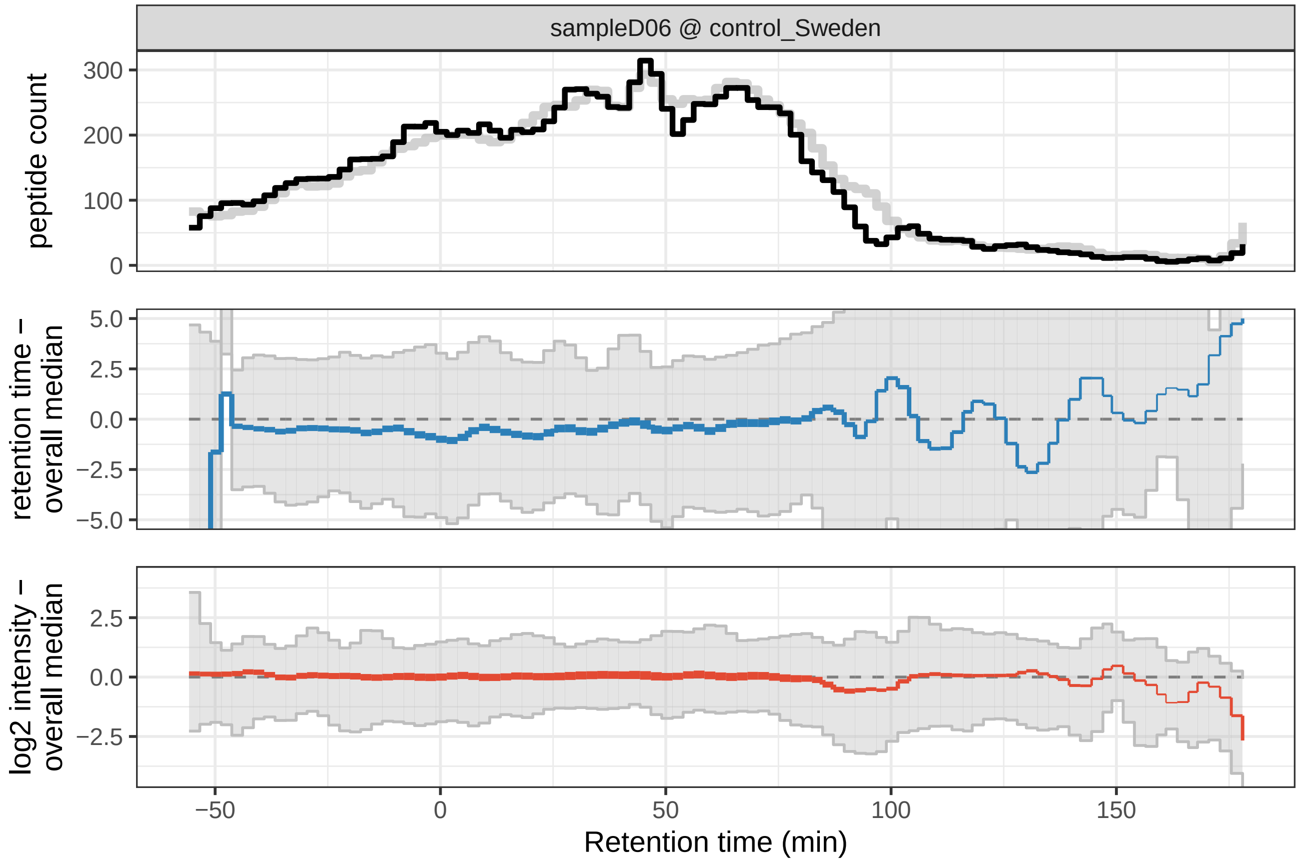
sampleD06 @ control_Sweden
peptide count
retention time –
overall median
log2 intensity –
overall median
Retention time (min)

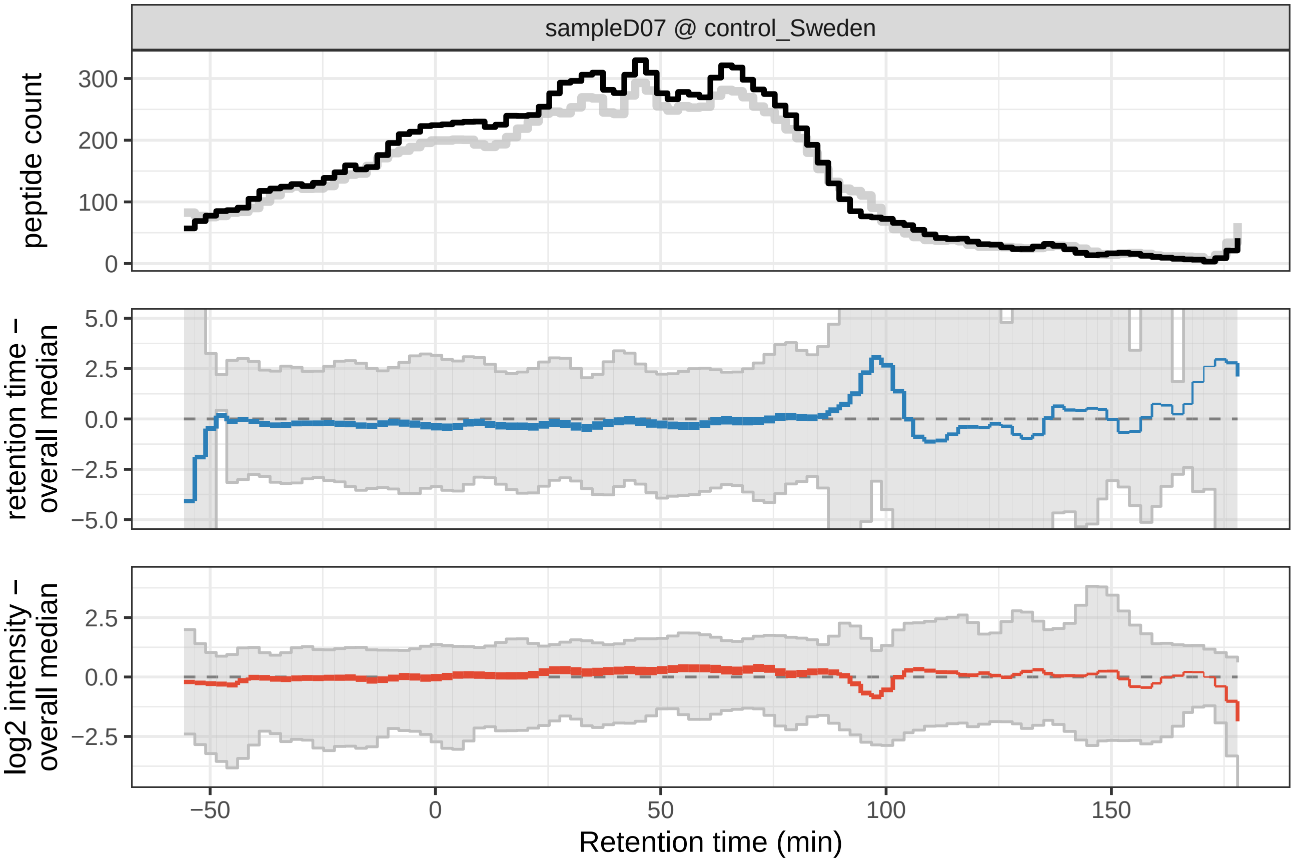
sampleD07 @ control_Sweden
peptide count
0
100
200
300
retention time – overall median
5.0
2.5
0.0
−2.5
−5.0
log2 intensity – overall median
2.5
0.0
−2.5
−50
0
50
100
150
Retention time (min)

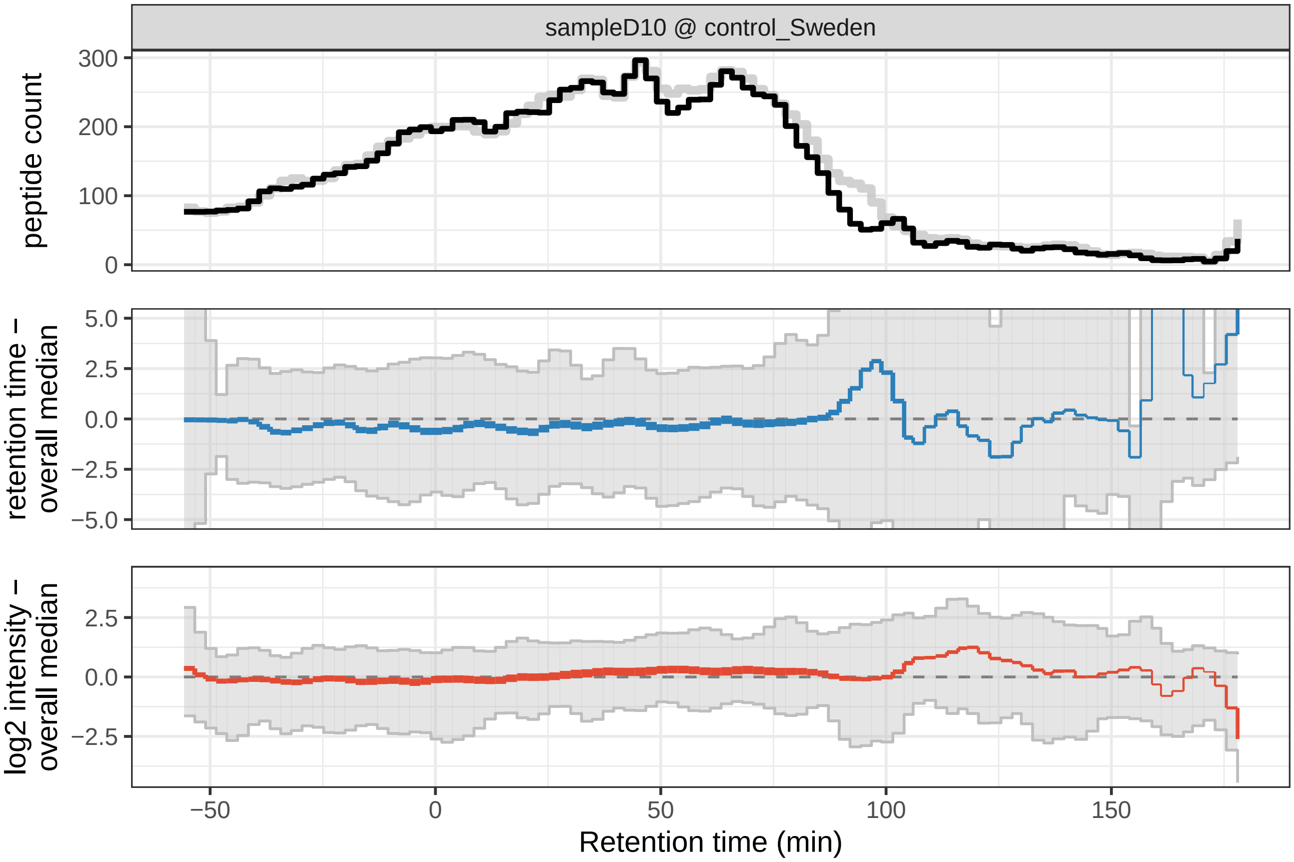
sampleD10 @ control_Sweden
peptide count
0
100
200
300
retention time – overall median
5.0
2.5
0.0
−2.5
−5.0
log2 intensity – overall median
2.5
0.0
−2.5
−50
0
50
100
150
Retention time (min)

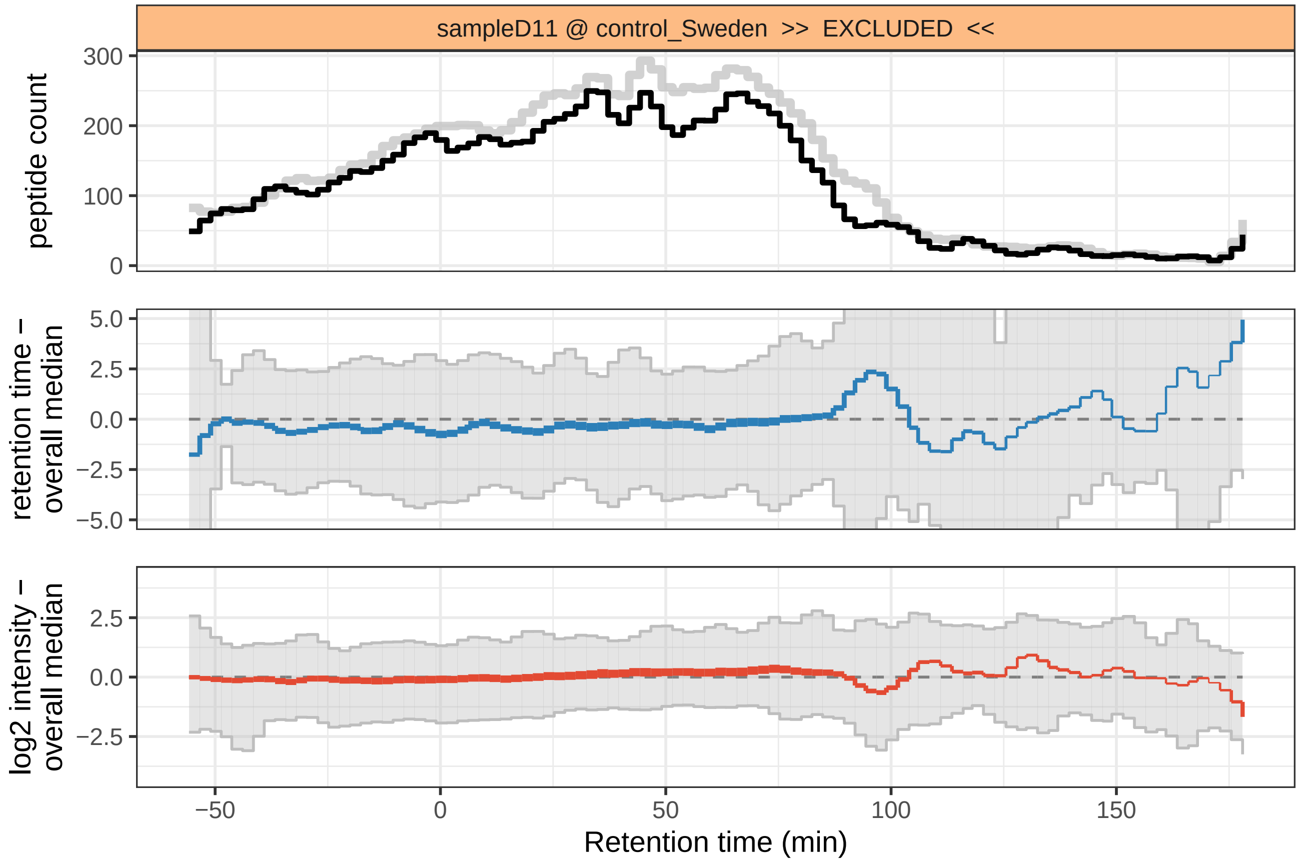
sampleD11 @ control_Sweden >> EXCLUDED <<
peptide count
300
200
100
0
retention time – overall median
5.0
2.5
0.0
−2.5
−5.0
log2 intensity – overall median
2.5
0.0
−2.5
−50
0
50
100
150
Retention time (min)

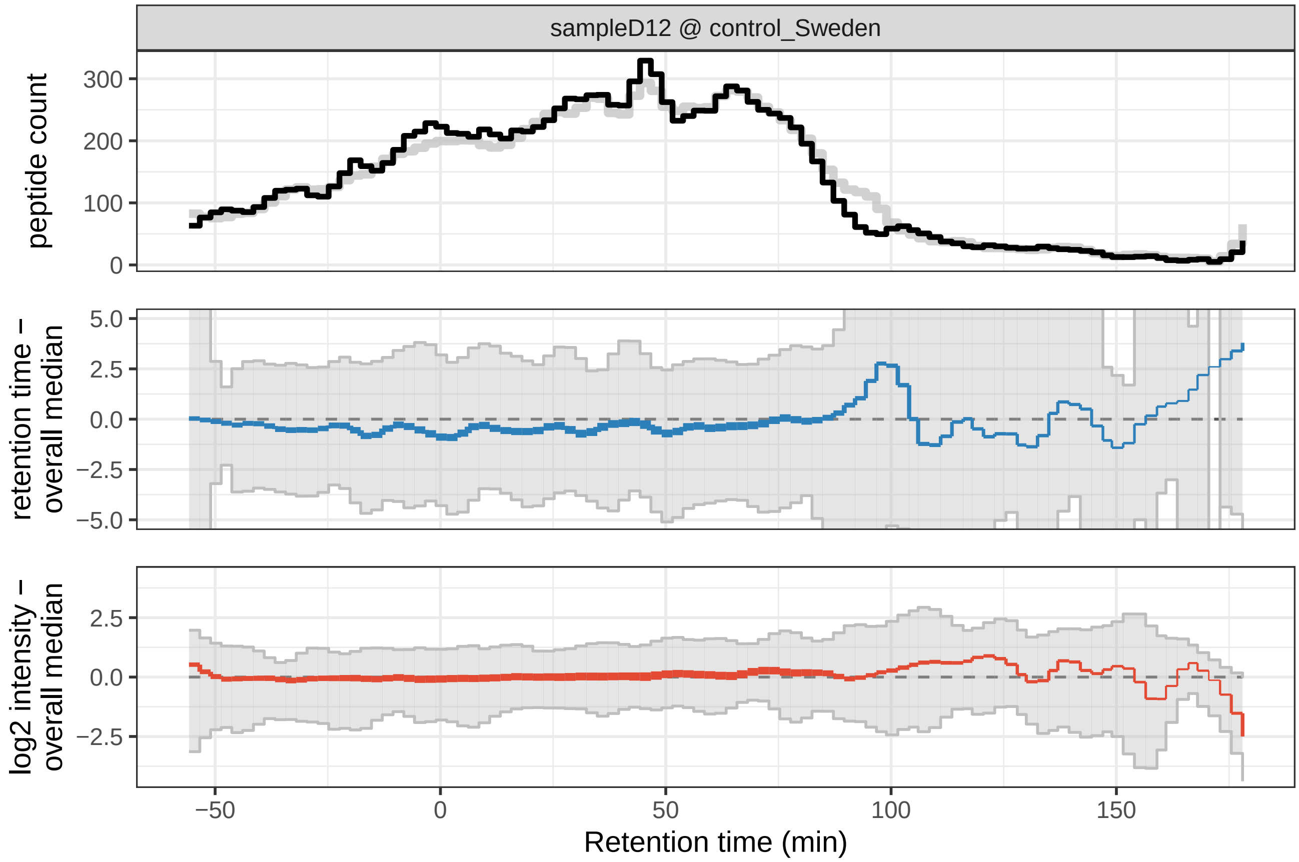
sampleD12 @ control_Sweden
peptide count
300
200
100
0
retention time – overall median
5.0
2.5
0.0
−2.5
−5.0
log2 intensity – overall median
2.5
0.0
−2.5
−50
0
50
100
150
Retention time (min)

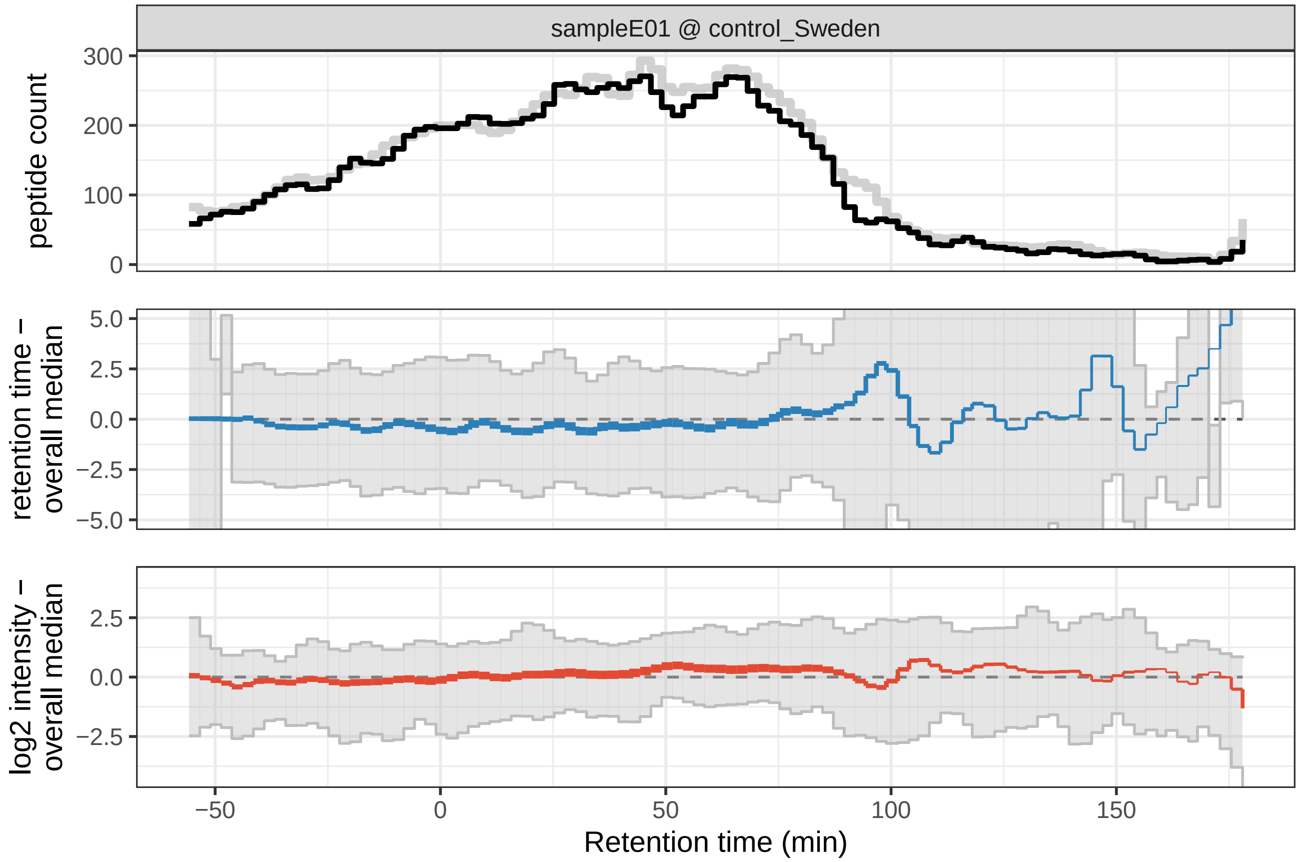
sampleE01 @ control_Sweden
peptide count
retention time – overall median
log2 intensity – overall median
Retention time (min)

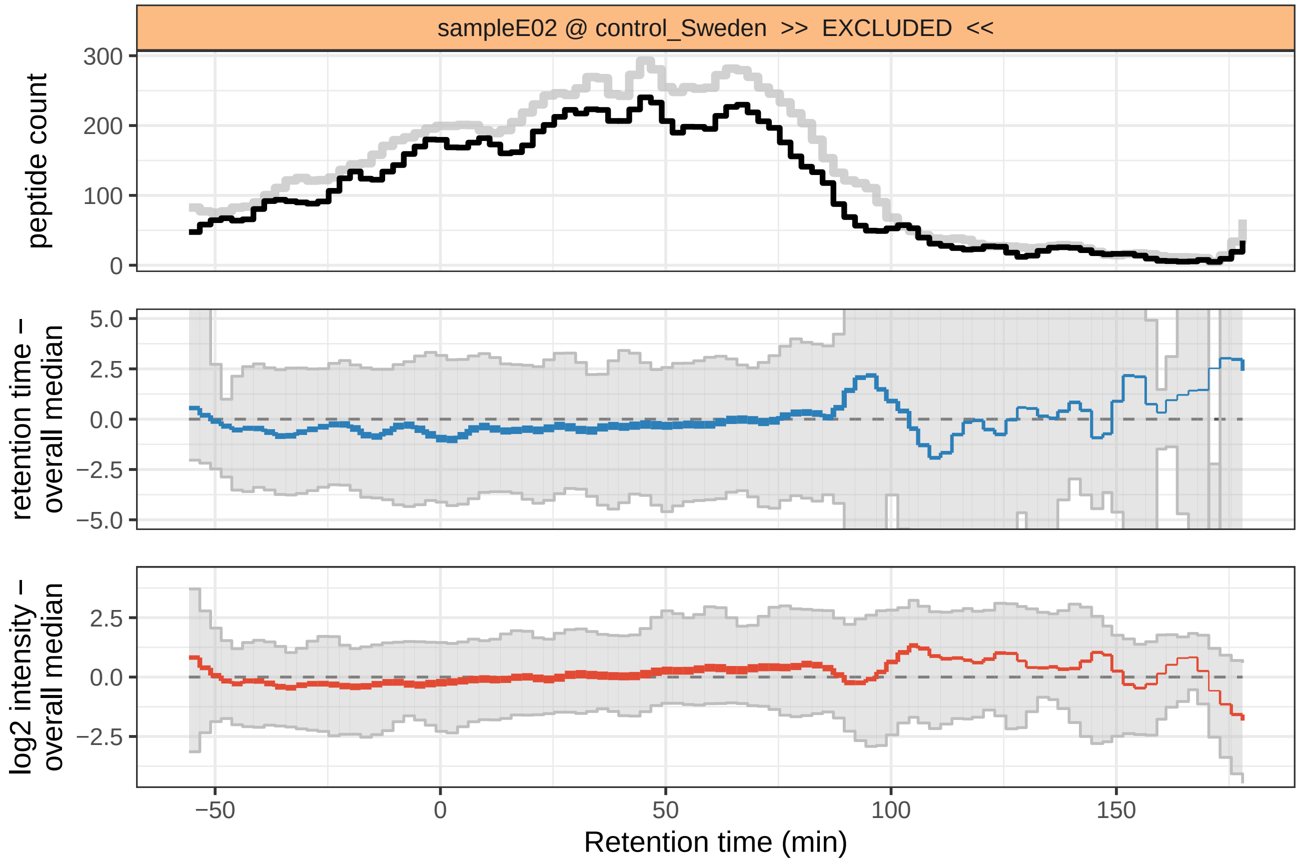
sampleE02 @ control_Sweden >> EXCLUDED <<
peptide count
retention time – overall median
log2 intensity – overall median
Retention time (min)

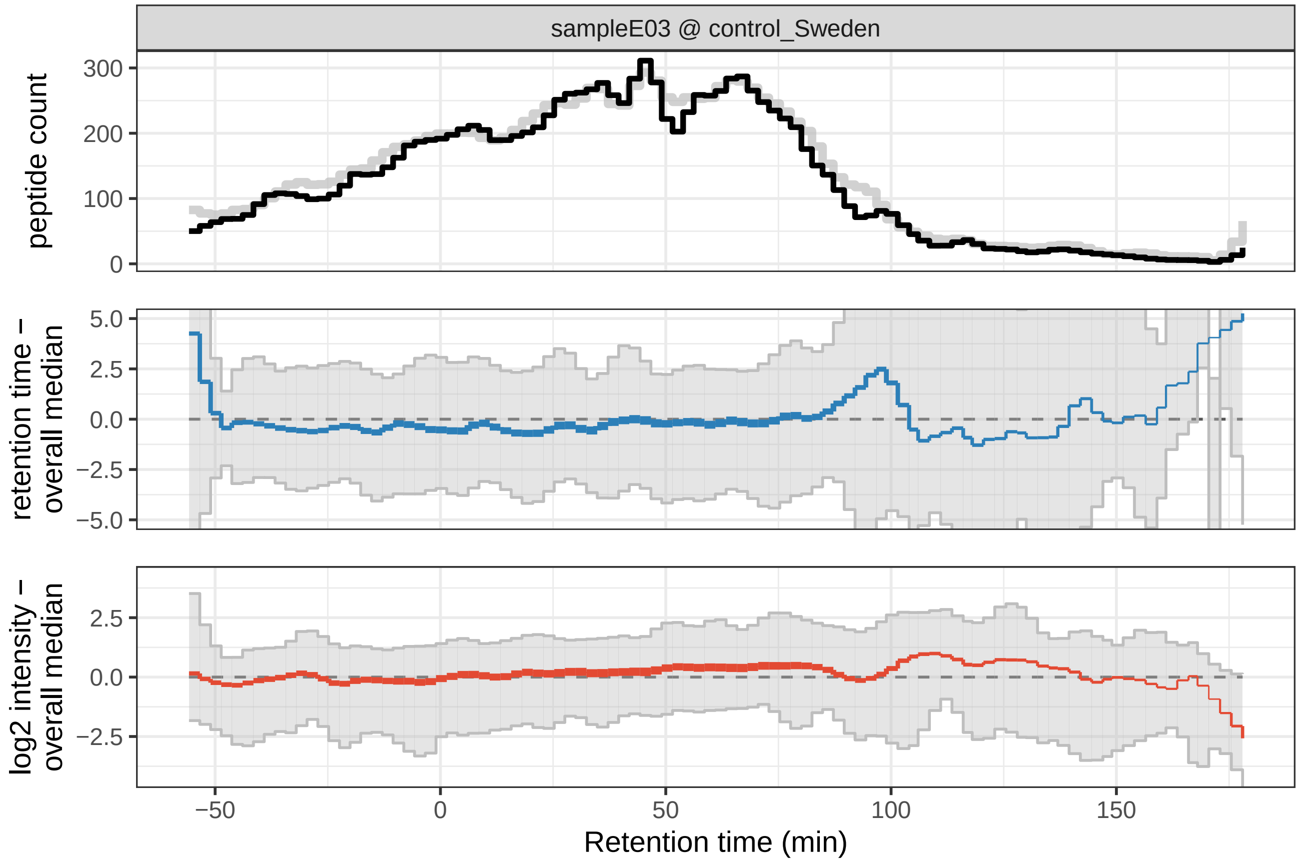
sampleE03 @ control_Sweden
peptide count
retention time – overall median
log2 intensity – overall median
Retention time (min)

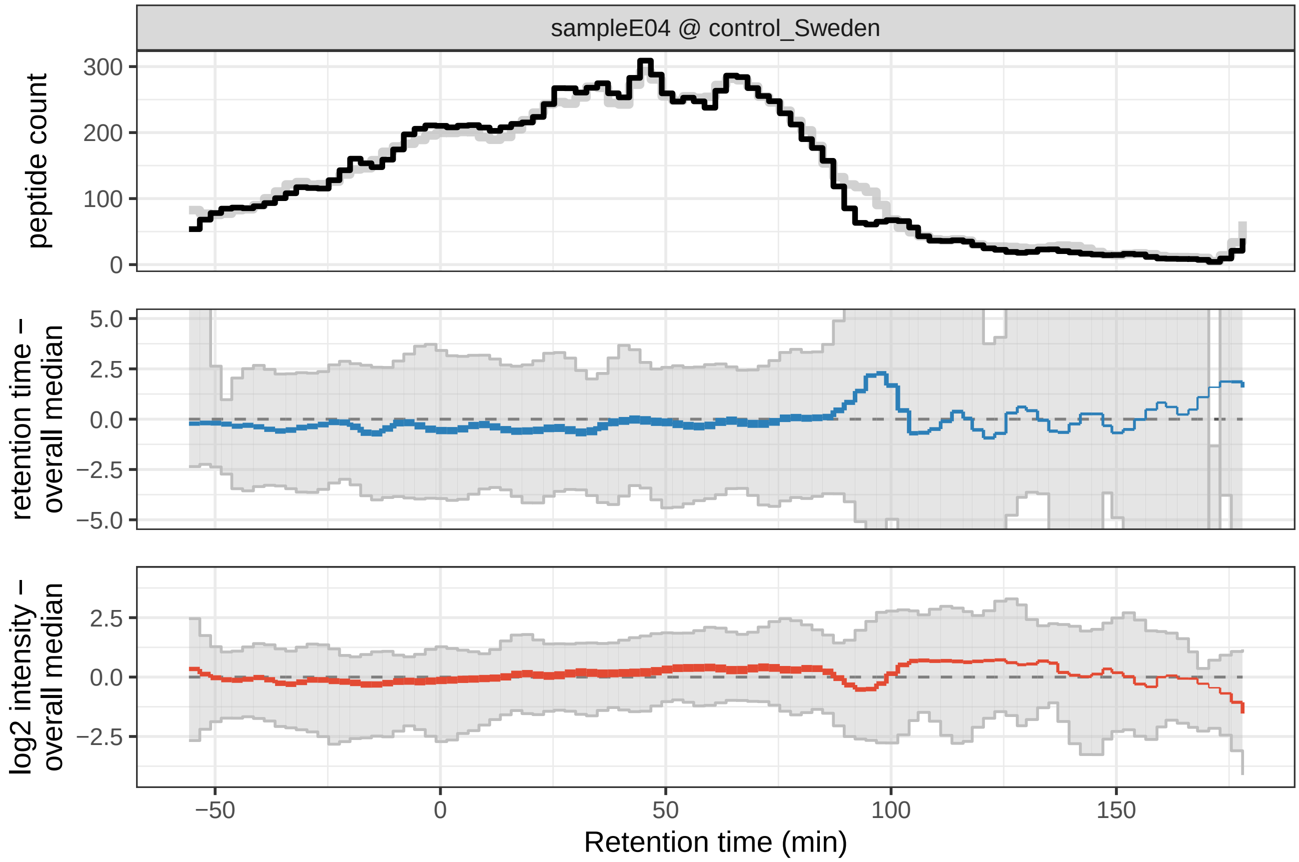
sampleE04 @ control_Sweden
peptide count
retention time – overall median
log2 intensity – overall median
Retention time (min)

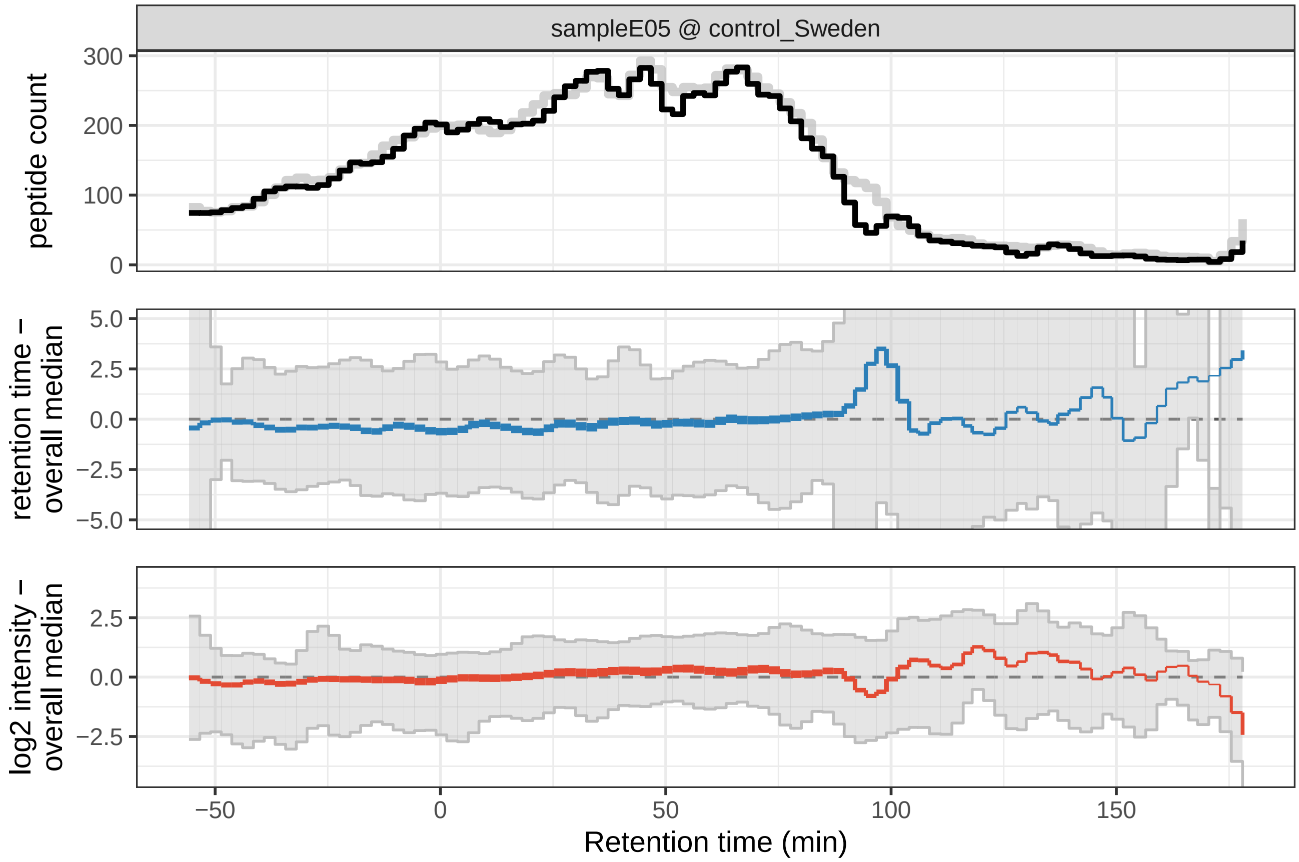
sampleE05 @ control_Sweden
peptide count
300
200
100
0
retention time –
overall median
5.0
2.5
0.0
−2.5
−5.0
log2 intensity –
overall median
2.5
0.0
−2.5
−50
0
50
100
150
Retention time (min)

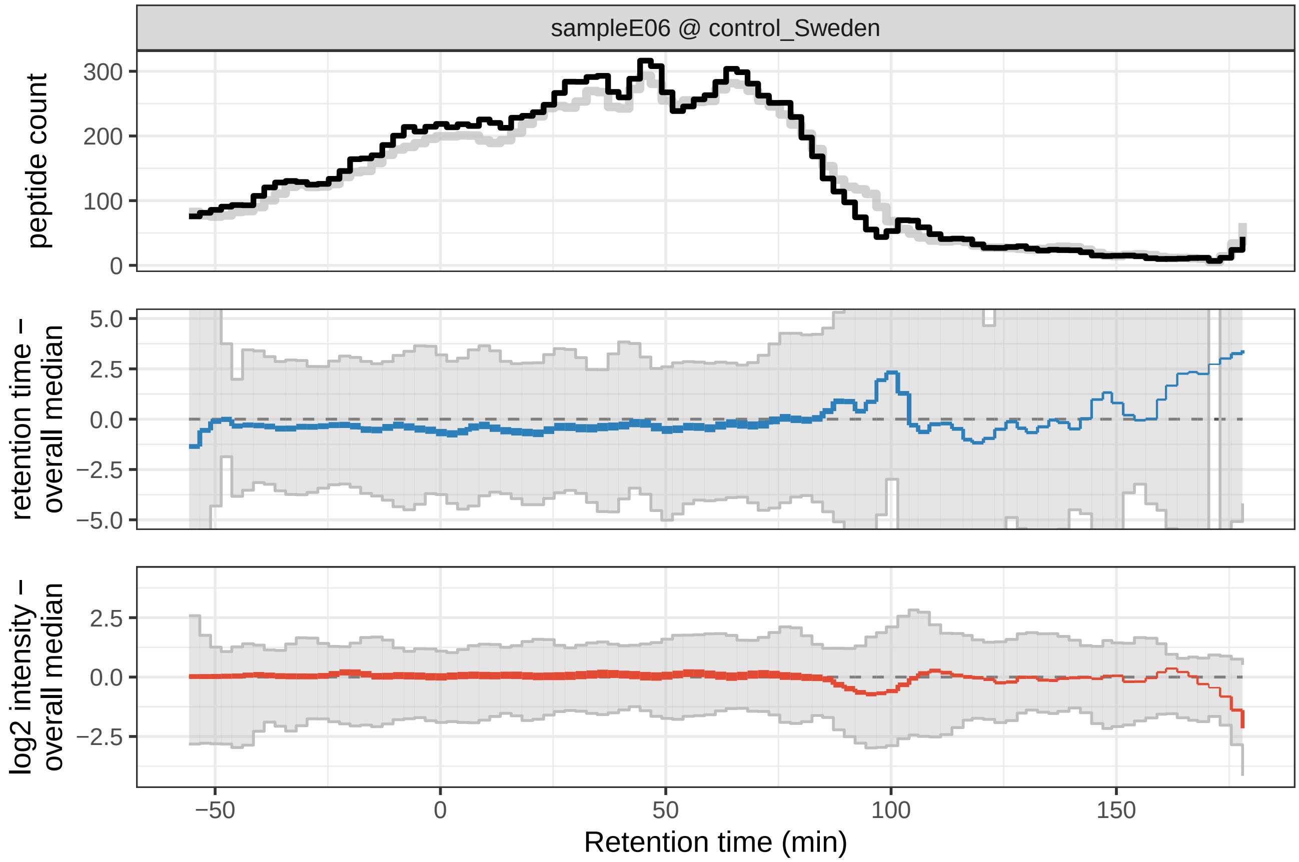
sampleE06 @ control_Sweden
peptide count
300
200
100
0
retention time –
overall median
5.0
2.5
0.0
−2.5
−5.0
log2 intensity –
overall median
2.5
0.0
−2.5
−50
0
50
100
150
Retention time (min)

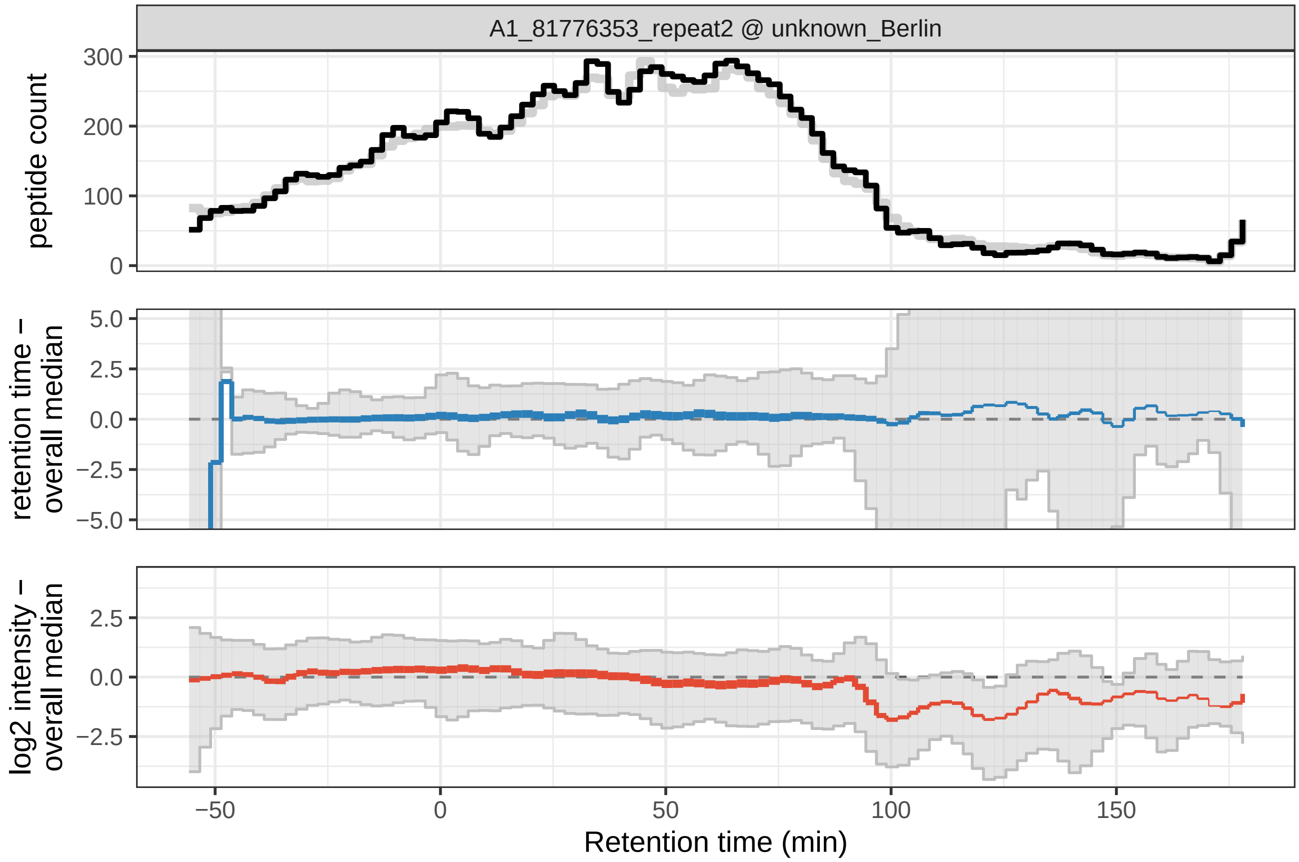
A1_81776353_repeat2 @ unknown_Berlin
peptide count
retention time – overall median
log2 intensity – overall median
Retention time (min)

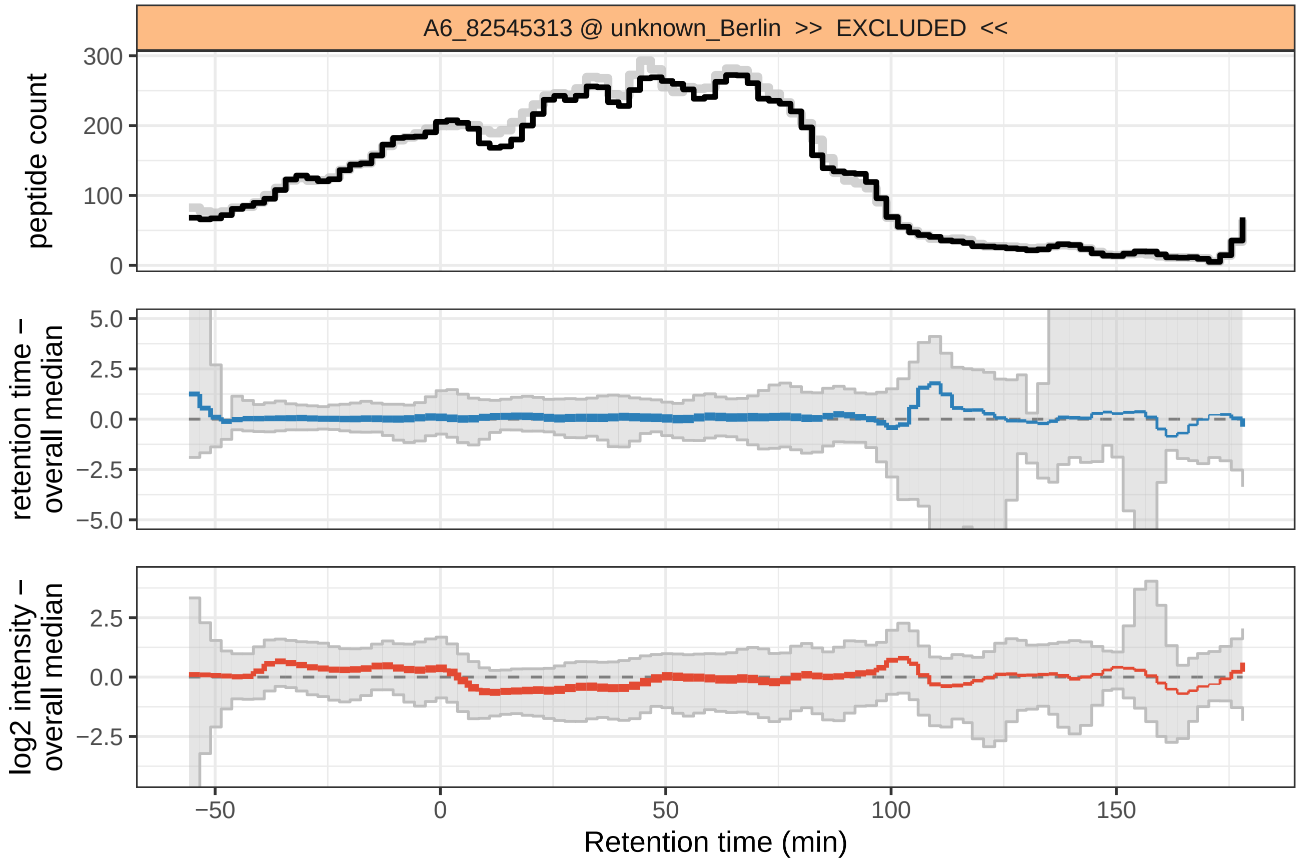
A6_82545313 @ unknown_Berlin >> EXCLUDED <<
peptide count
retention time – overall median
log2 intensity – overall median
Retention time (min)

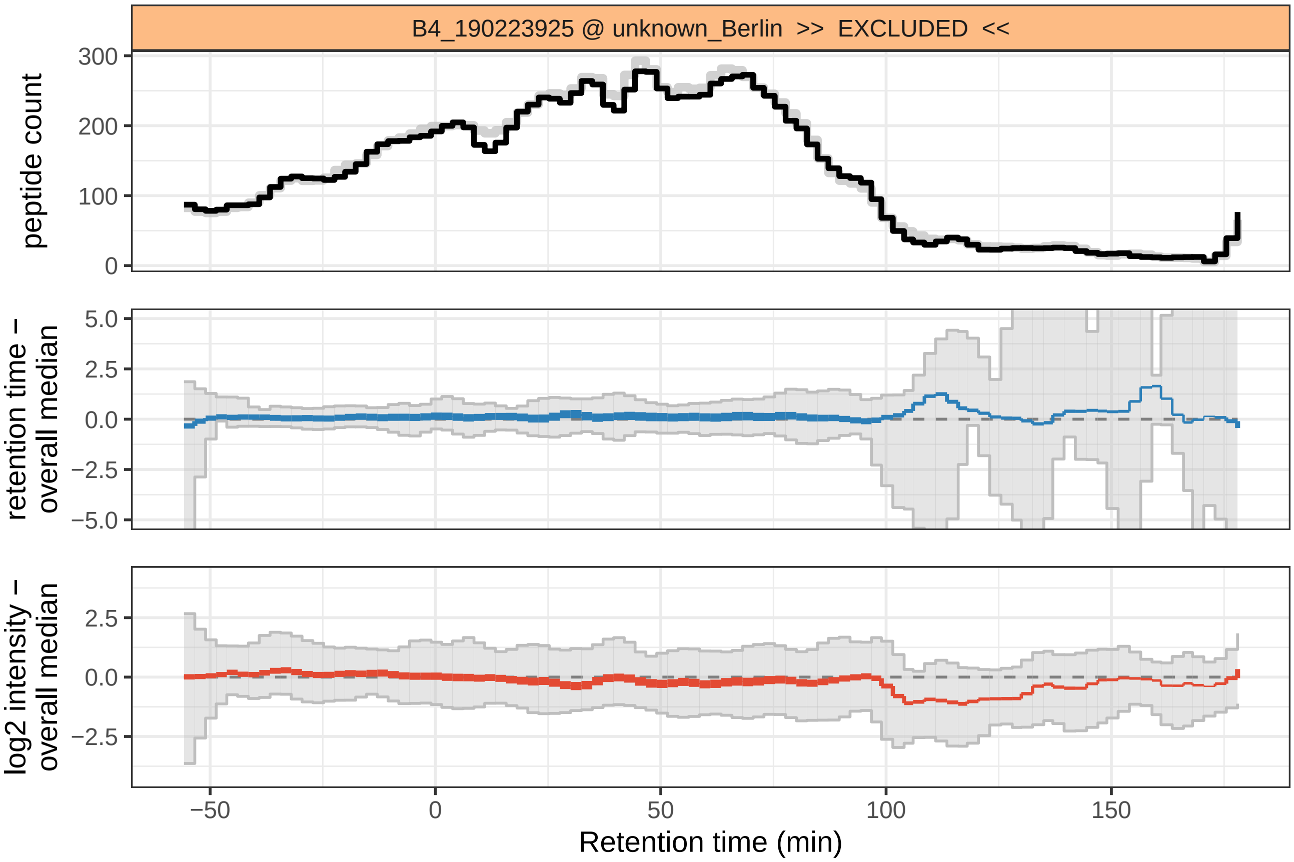
B4_190223925 @ unknown_Berlin >> EXCLUDED <<
peptide count
retention time – overall median
log2 intensity – overall median
Retention time (min)

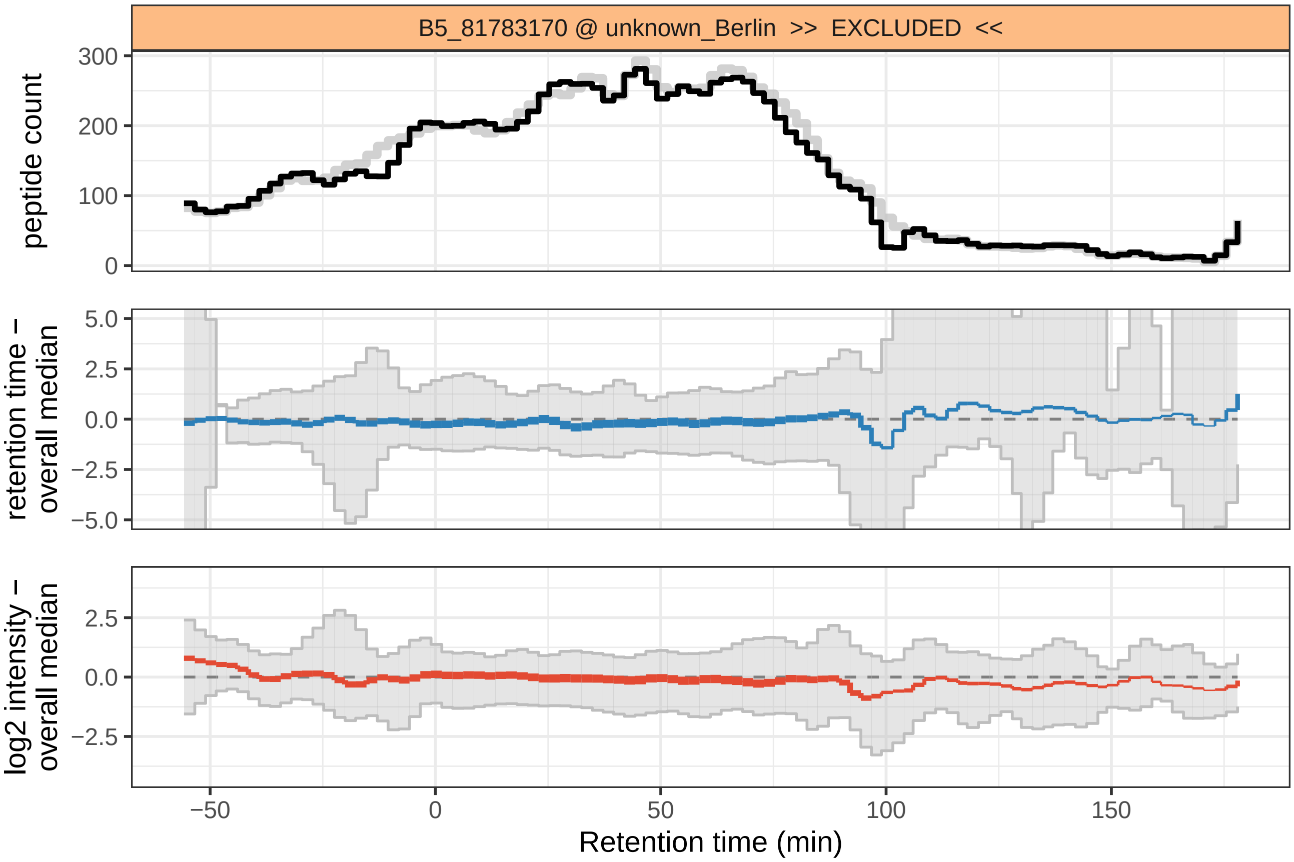
B5_81783170 @ unknown_Berlin >> EXCLUDED <<
peptide count
retention time – overall median
log2 intensity – overall median
Retention time (min)

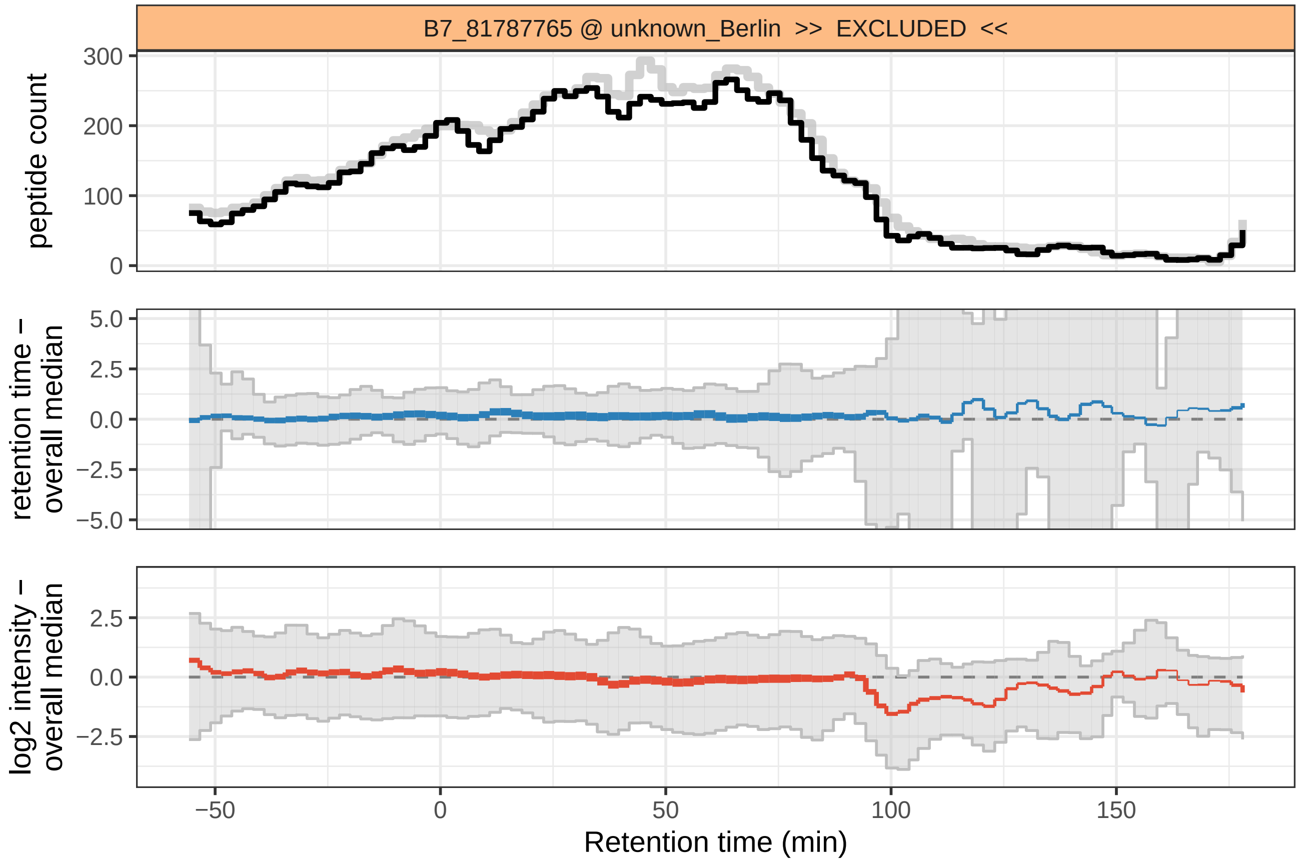

B7_81787765 @ unknown_Berlin >> EXCLUDED <<
peptide count
retention time – overall median
log2 intensity – overall median
Retention time (min)

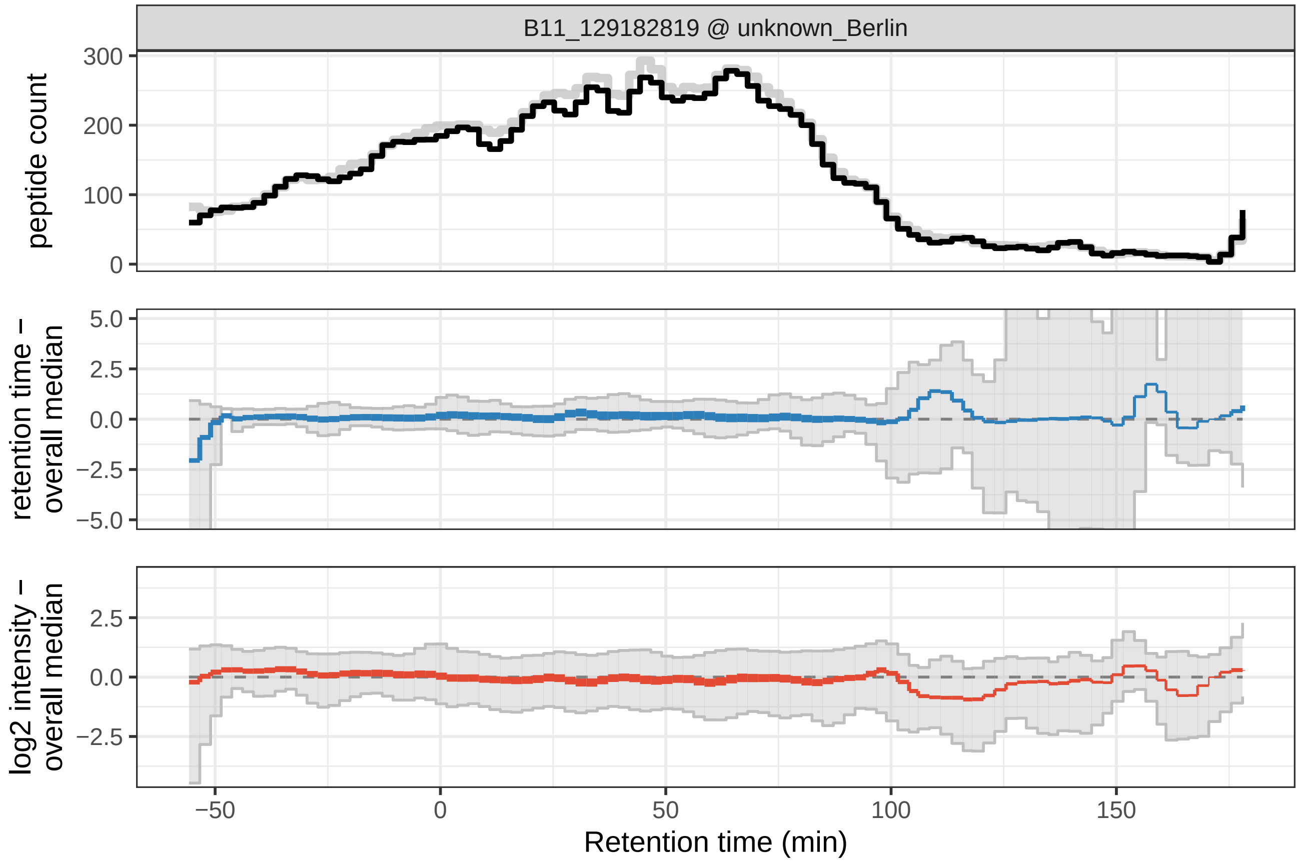

B11_129182819 @ unknown_Berlin
peptide count
retention time – overall median
log2 intensity – overall median
Retention time (min)

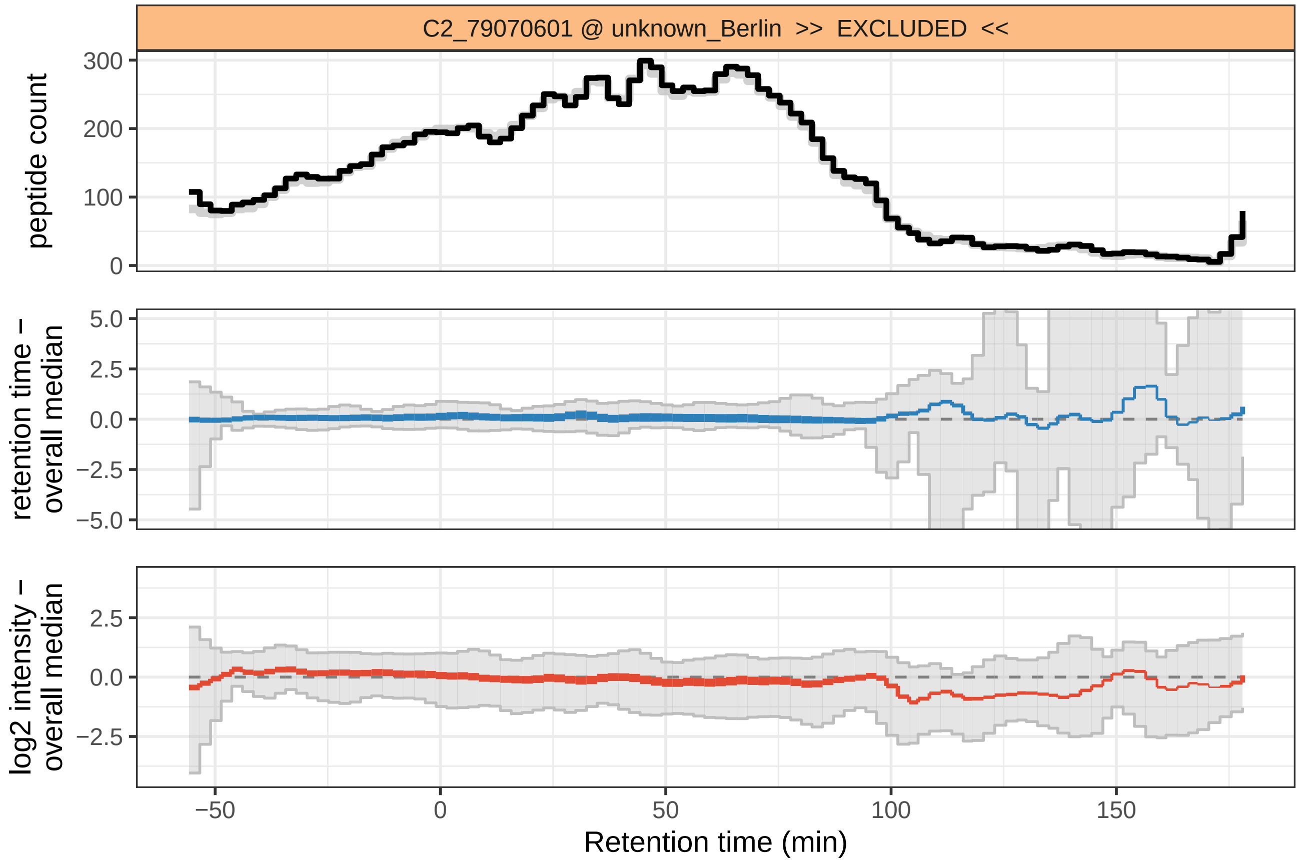
C2_79070601 @ unknown_Berlin >> EXCLUDED <<
peptide count
retention time – overall median
log2 intensity – overall median
Retention time (min)

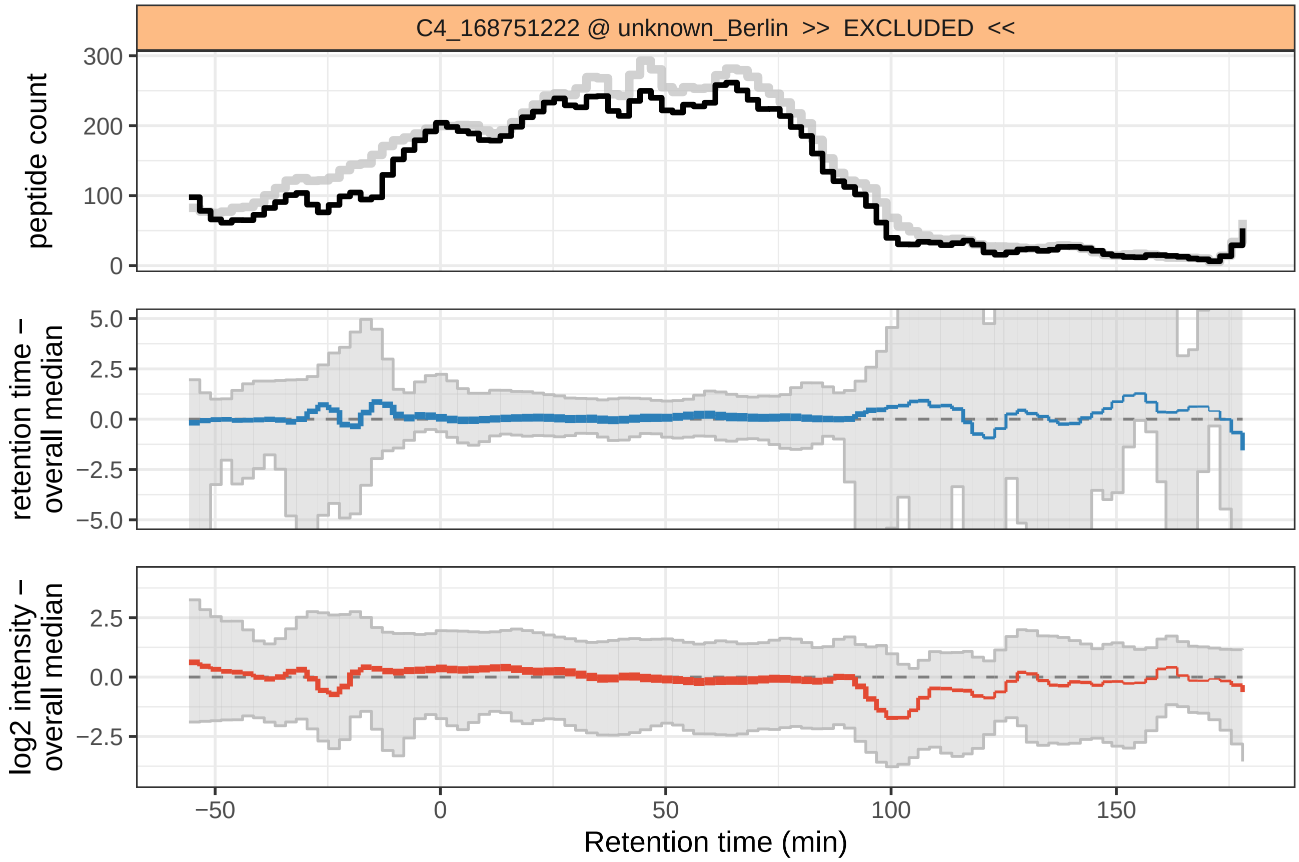
C4_168751222 @ unknown_Berlin >> EXCLUDED <<
peptide count
retention time – overall median
log2 intensity – overall median
Retention time (min)

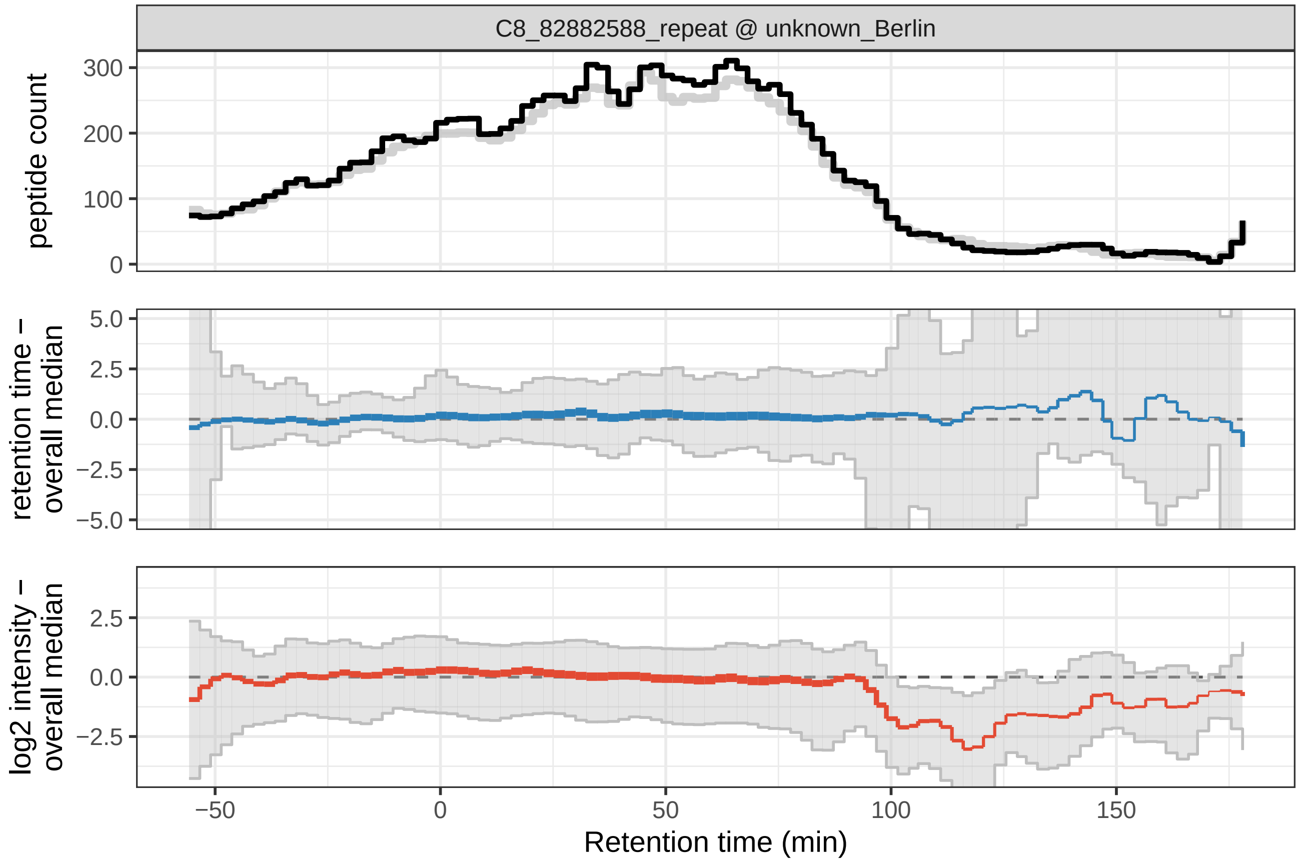
C8_82882588_repeat @ unknown_Berlin
peptide count
retention time – overall median
log2 intensity – overall median
300
200
100
0
5.0
2.5
0.0
−2.5
−5.0
−50
50
100
150
Retention time (min)

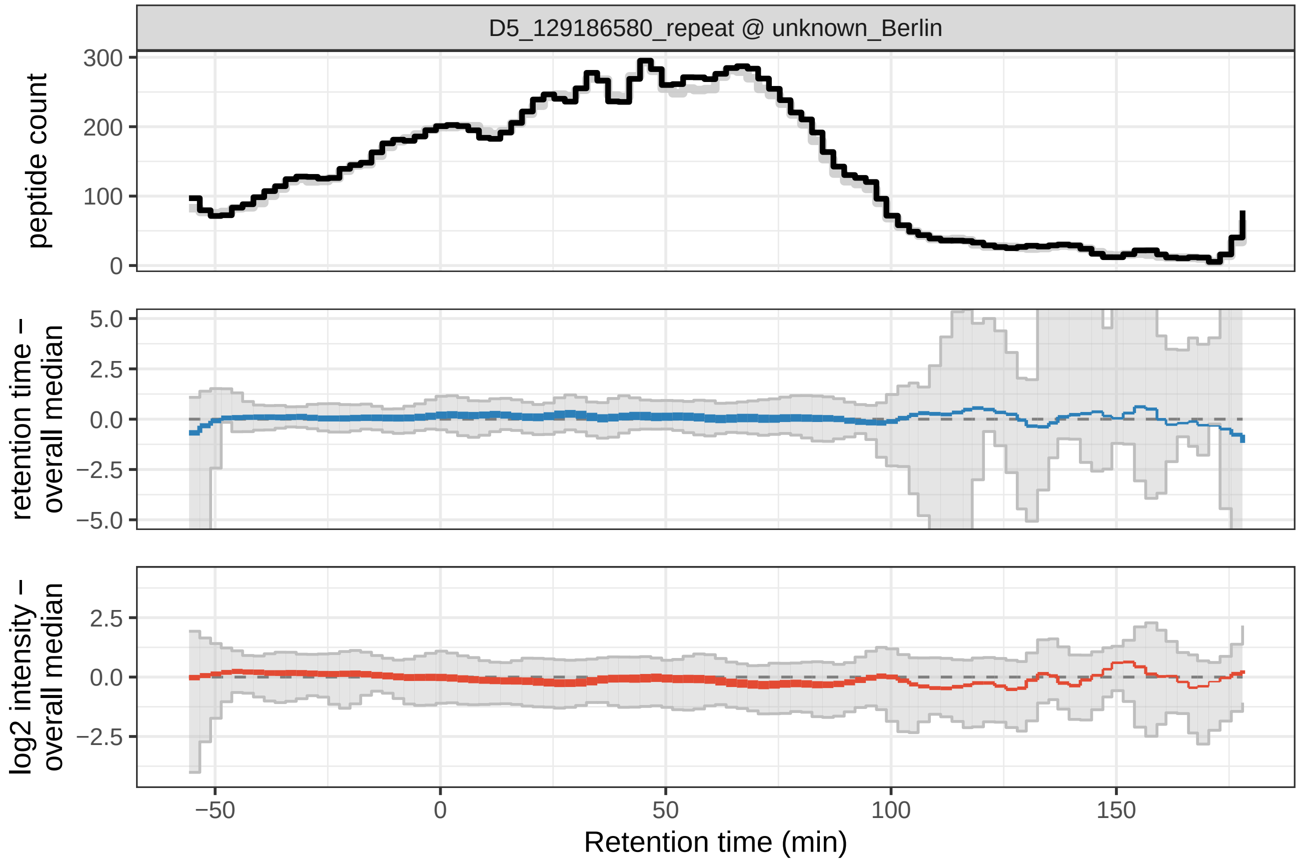
D5_129186580_repeat @ unknown_Berlin
peptide count
retention time – overall median
log2 intensity – overall median
300
200
100
0
5.0
2.5
0.0
−2.5
−5.0
−50
50
100
150
Retention time (min)

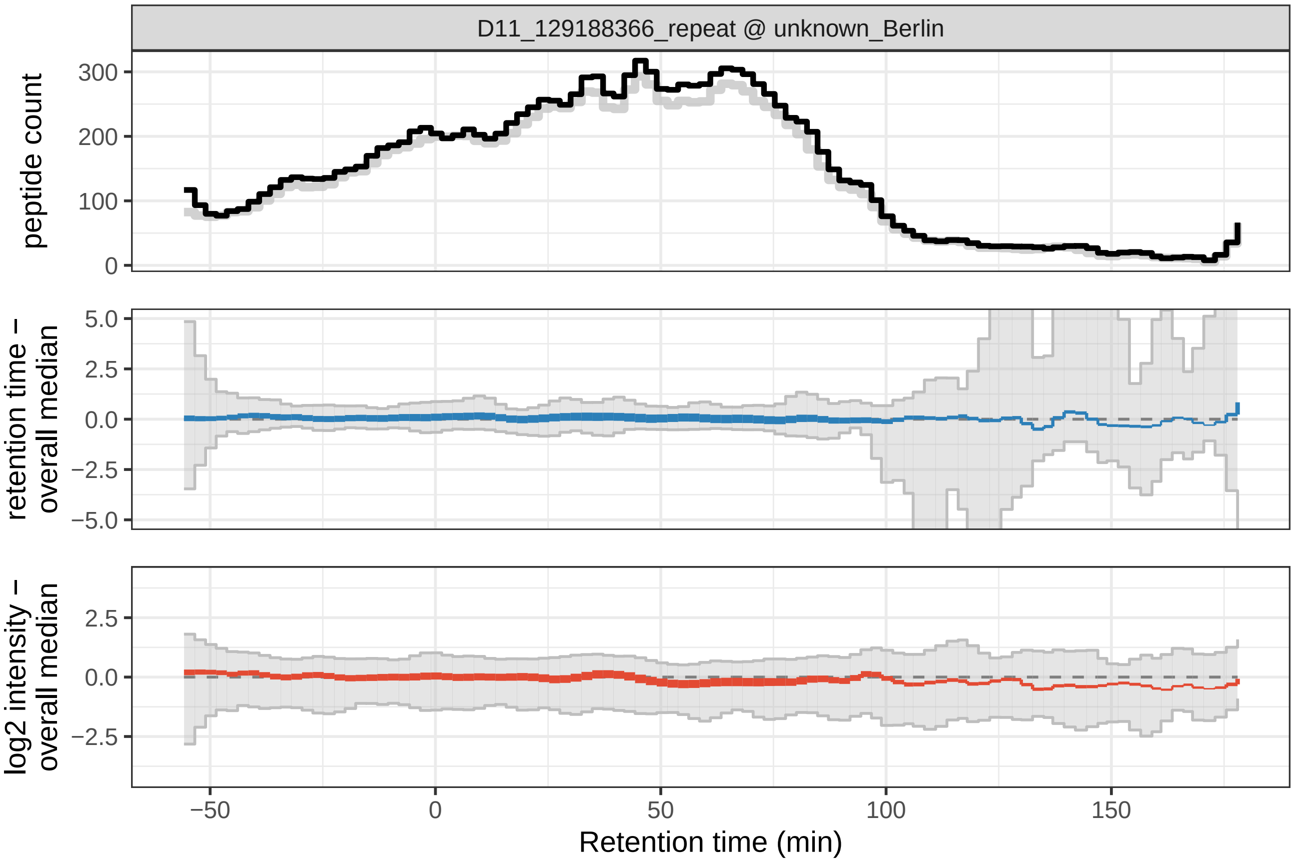
D11_129188366_repeat @ unknown_Berlin
peptide count
300
200
100
0
retention time – overall median
5.0
2.5
0.0
−2.5
−5.0
log2 intensity – overall median
2.5
0.0
−2.5
−50
0
50
100
150
Retention time (min)

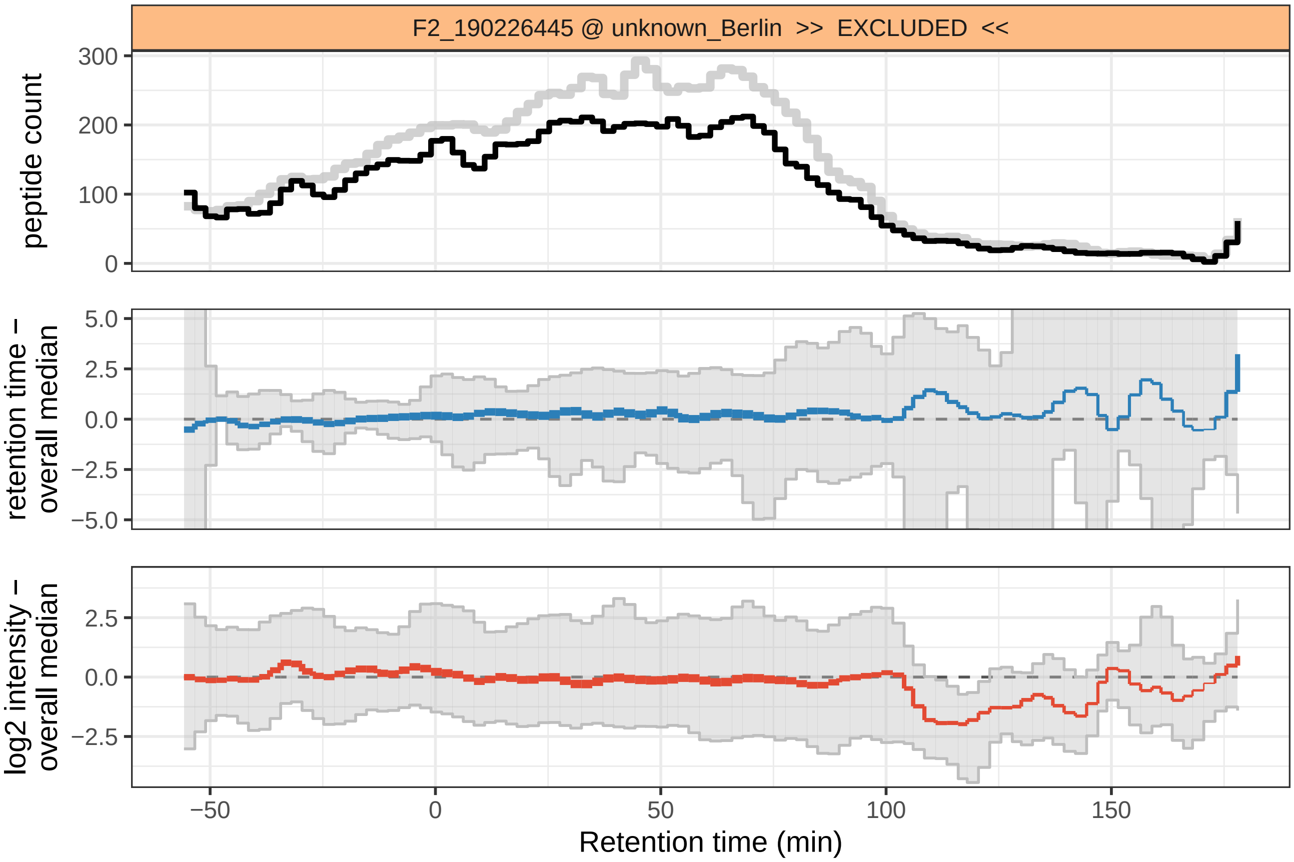
F2_190226445 @ unknown_Berlin >> EXCLUDED <<
peptide count
300
200
100
0
retention time – overall median
5.0
2.5
0.0
−2.5
−5.0
log2 intensity – overall median
2.5
0.0
−2.5
−50
0
50
100
150
Retention time (min)

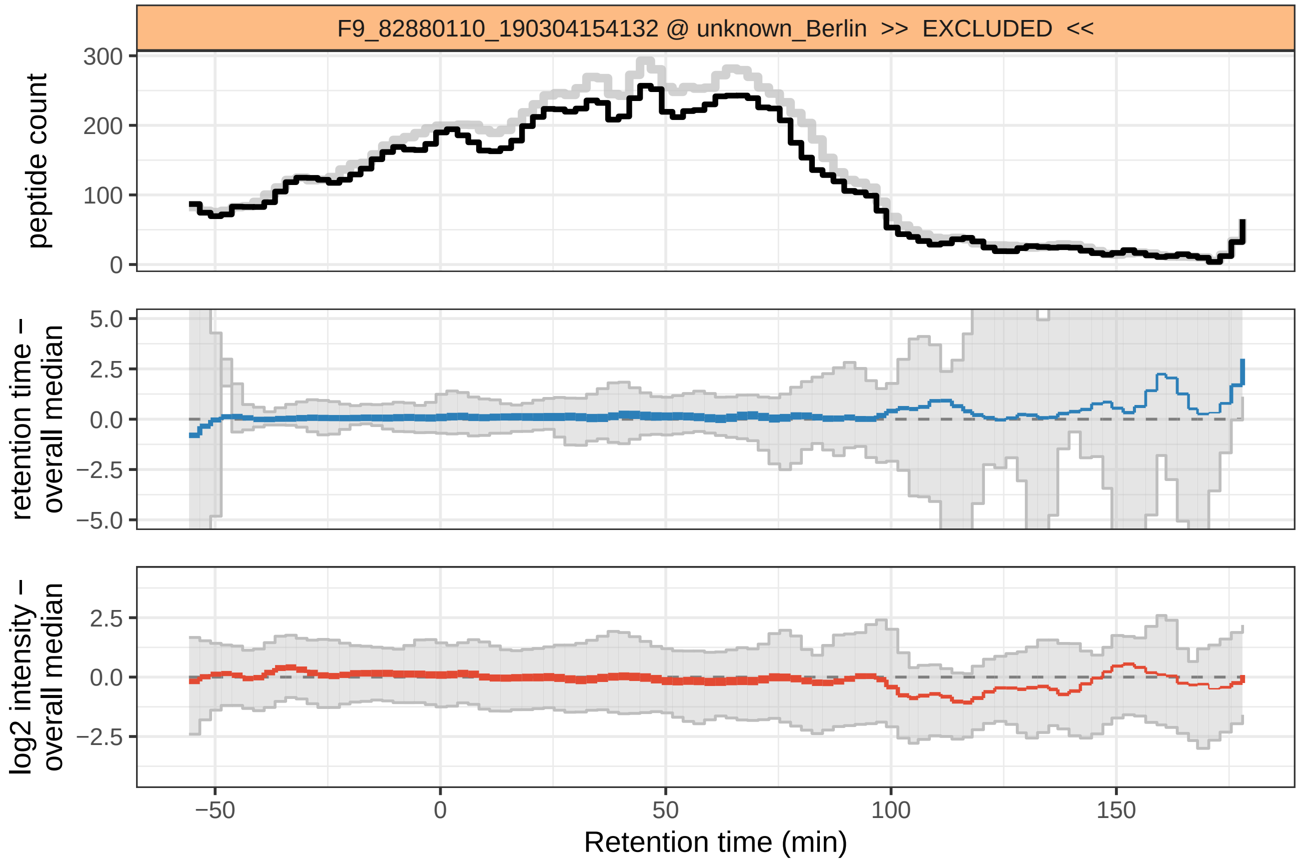
F9_82880110_190304154132 @ unknown_Berlin >> EXCLUDED <<
peptide count
300
200
100
0
retention time – overall median
5.0
2.5
0.0
−2.5
−5.0
log2 intensity – overall median
2.5
0.0
−2.5
−50
0
50
100
150
Retention time (min)

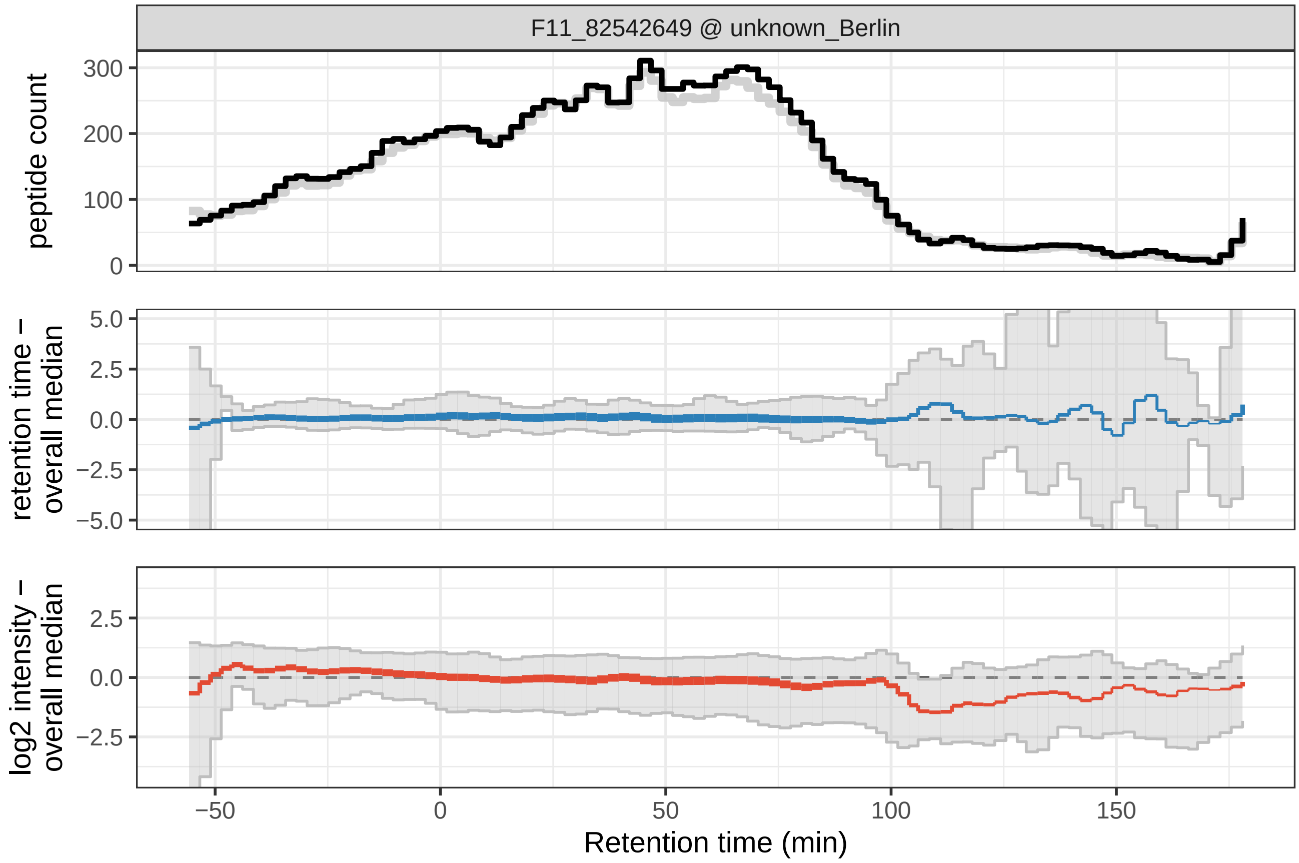
F11_82542649 @ unknown_Berlin
peptide count
300
200
100
0
retention time – overall median
5.0
2.5
0.0
−2.5
−5.0
log2 intensity – overall median
2.5
0.0
−2.5
−50
0
50
100
150
Retention time (min)

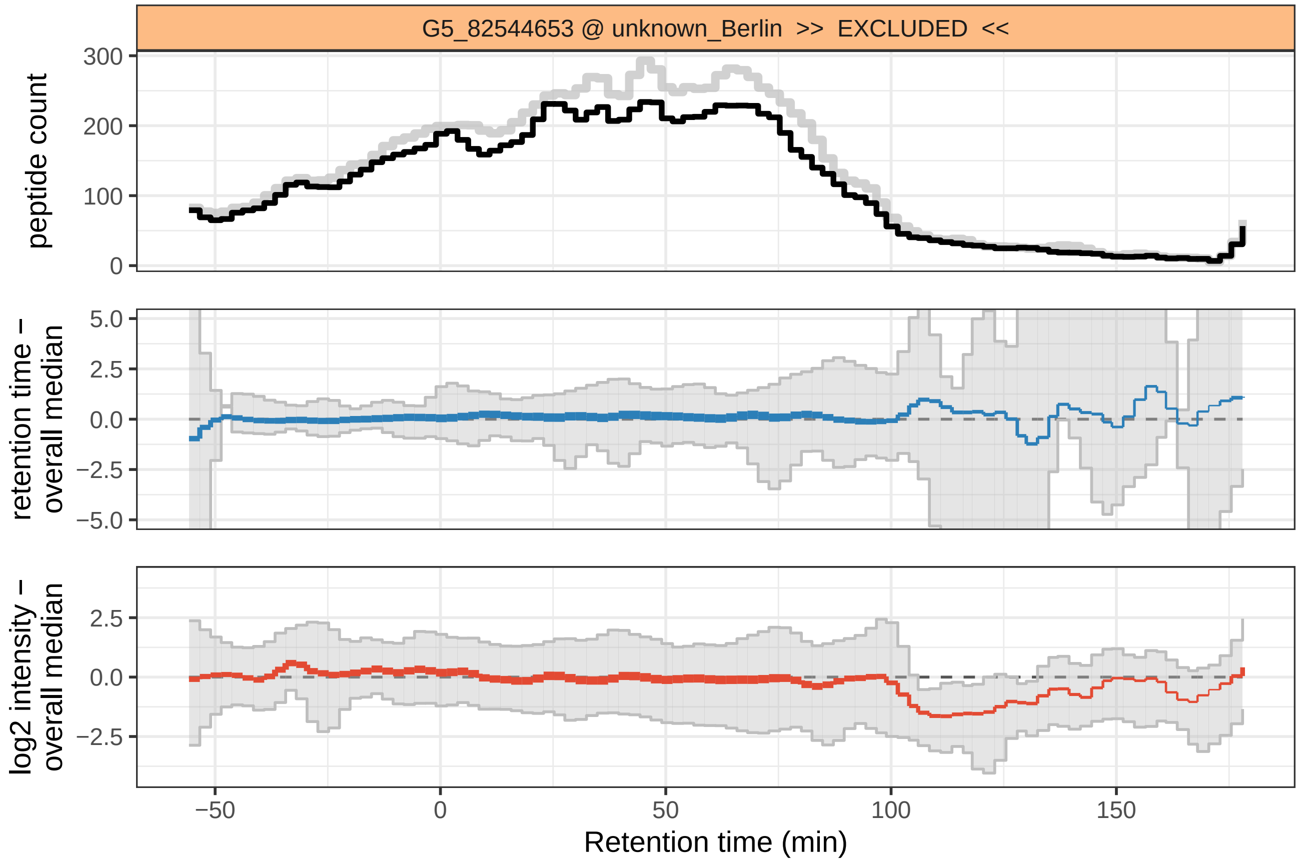
G5_82544653 @ unknown_Berlin >> EXCLUDED <<
peptide count
300
200
100
0
retention time – overall median
5.0
2.5
0.0
−2.5
−5.0
log2 intensity – overall median
2.5
0.0
−2.5
−50
0
50
100
150
Retention time (min)

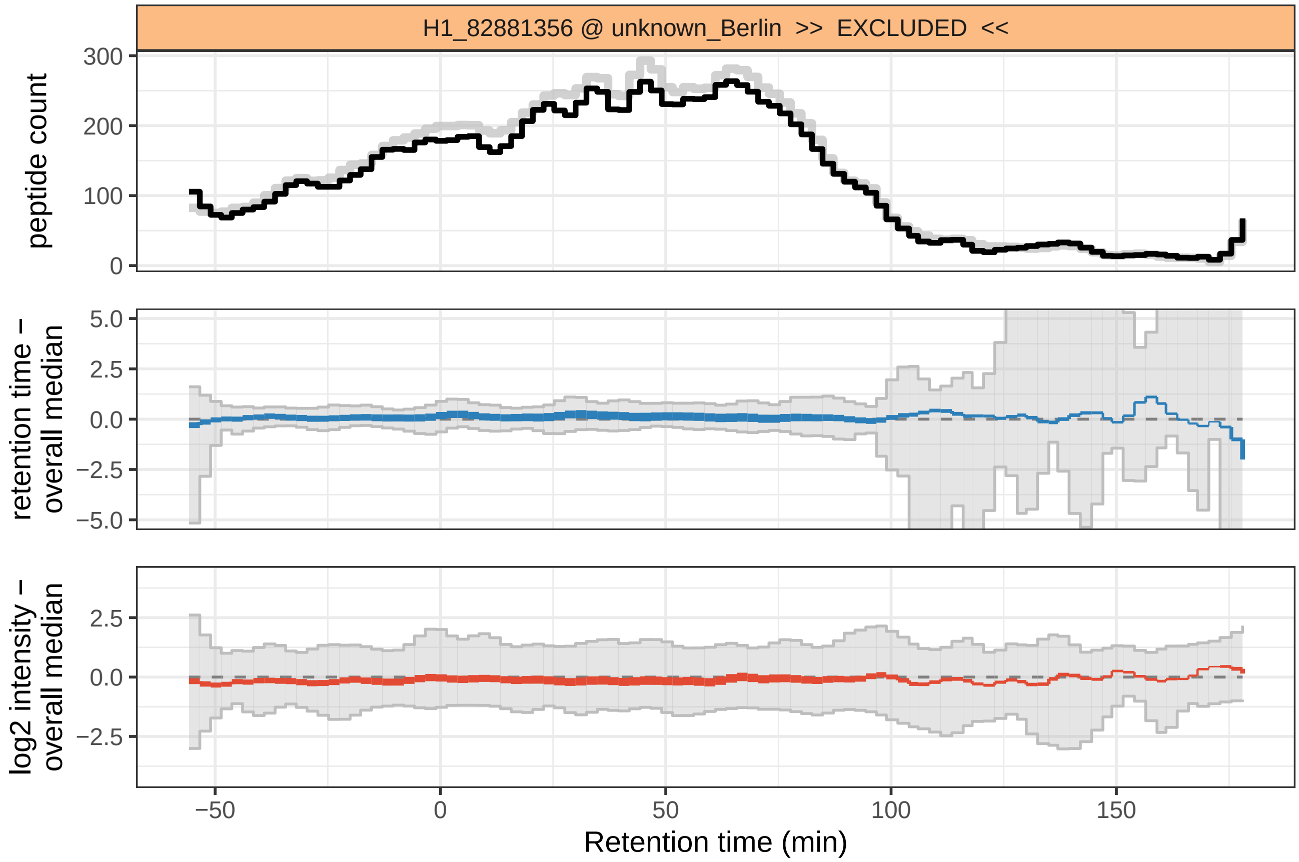
H1_82881356 @ unknown_Berlin >> EXCLUDED <<
peptide count
300
200
100
0
retention time – overall median
5.0
2.5
0.0
−2.5
−5.0
log2 intensity – overall median
2.5
0.0
−2.5
−50
0
50
100
150
Retention time (min)

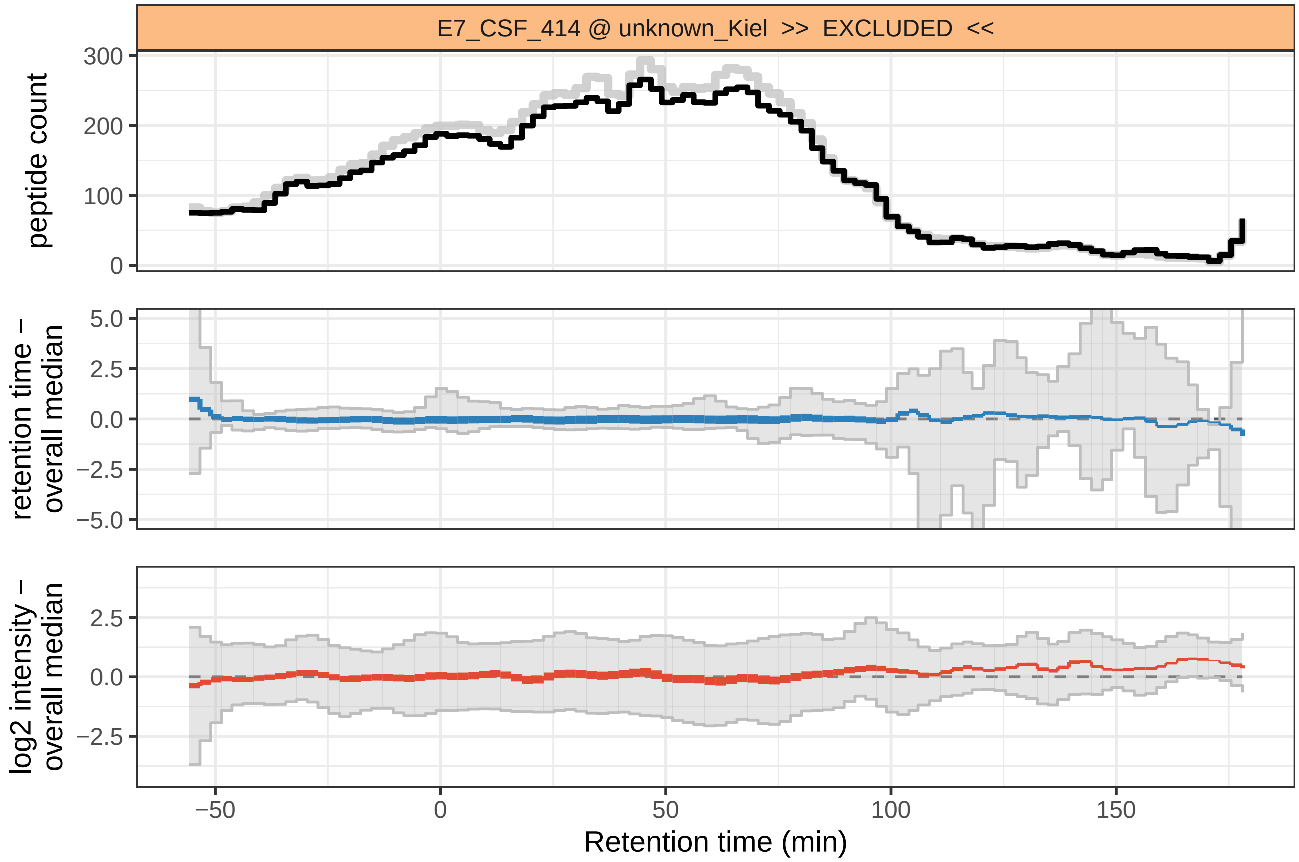
E7_CSF_414 @ unknown_Kiel >> EXCLUDED <<
peptide count
300
200
100
0
retention time −
overall median
5.0
2.5
0.0
−2.5
−5.0
log2 intensity −
overall median
2.5
0.0
−2.5
−50
0
50
100
150
Retention time (min)

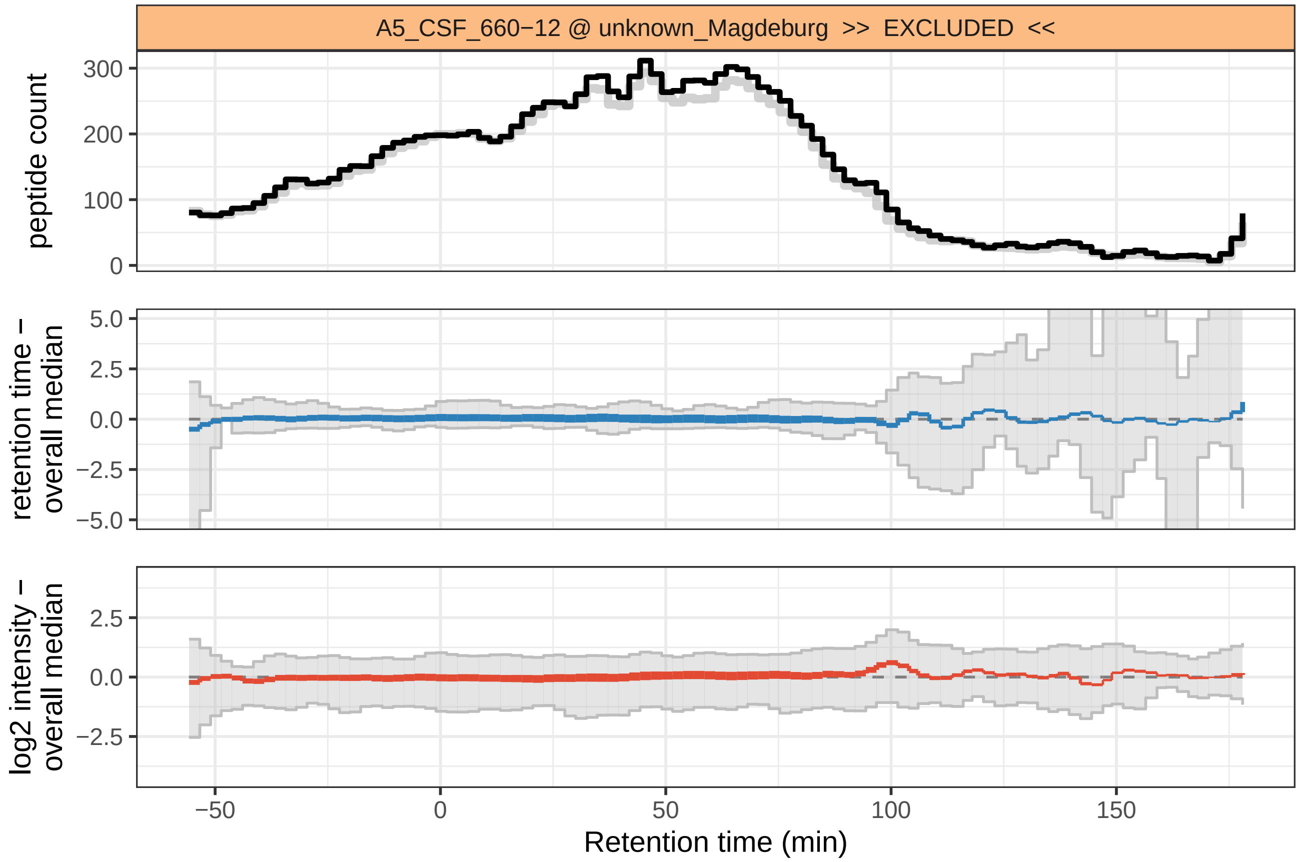
A5_CSF_660−12 @ unknown_Magdeburg >> EXCLUDED <<
peptide count
300
200
100
0
retention time −
overall median
5.0
2.5
0.0
−2.5
−5.0
log2 intensity −
overall median
2.5
0.0
−2.5
−50
0
50
100
150
Retention time (min)

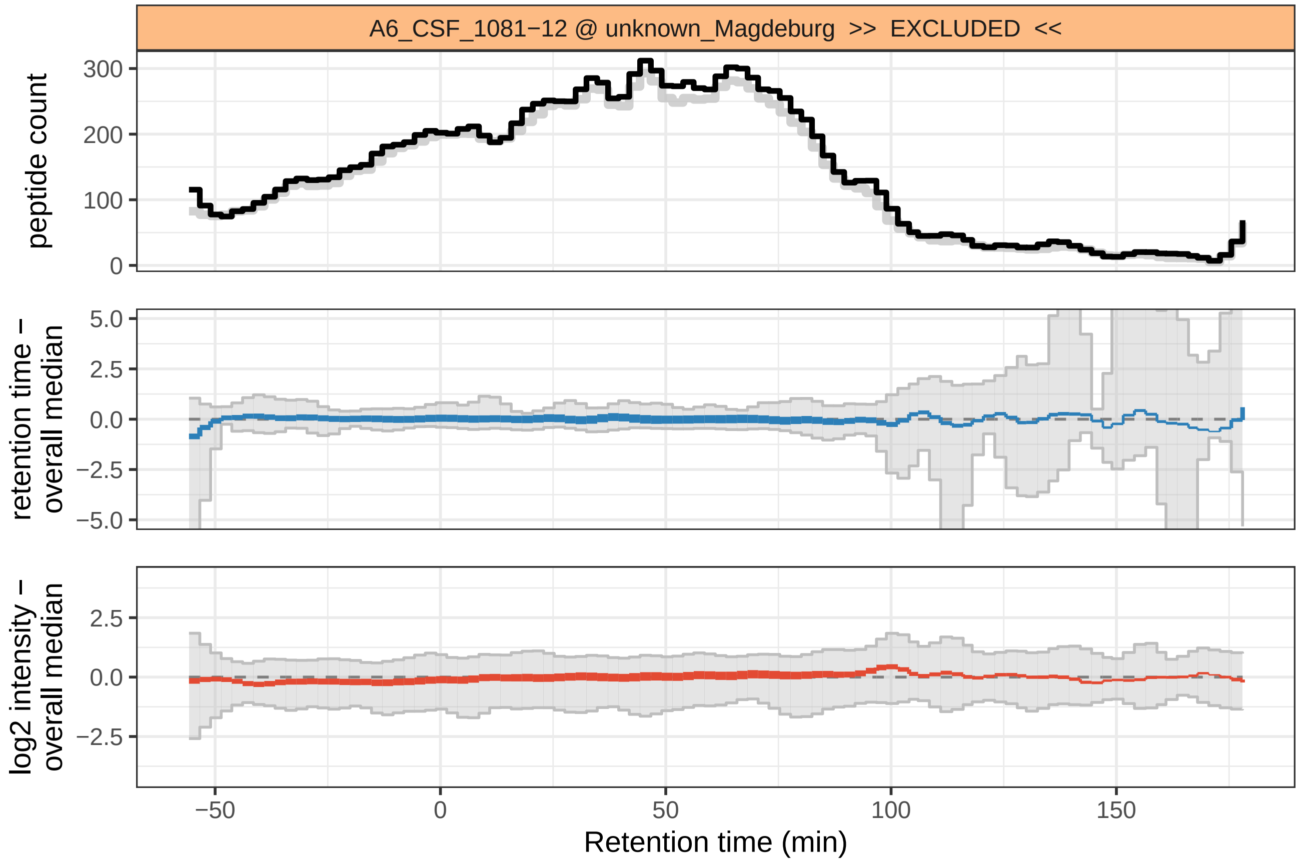
A6_CSF_1081−12 @ unknown_Magdeburg >> EXCLUDED <<
peptide count
300
200
100
0
retention time –
overall median
5.0
2.5
0.0
−2.5
−5.0
log2 intensity –
overall median
2.5
0.0
−2.5
−50
0
50
100
150
Retention time (min)

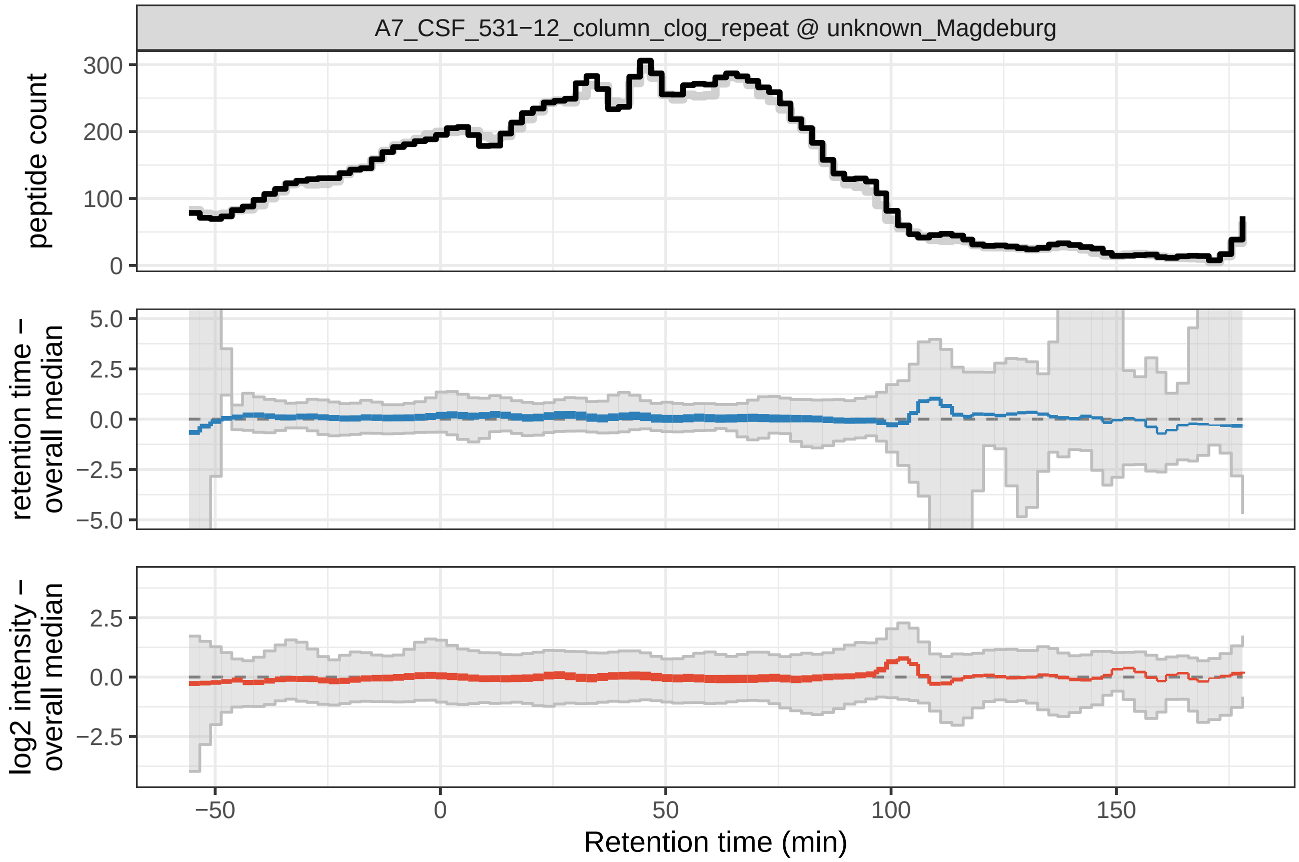
A7_CSF_531−12_column_clog_repeat @ unknown_Magdeburg
peptide count
300
200
100
0
retention time –
overall median
5.0
2.5
0.0
−2.5
−5.0
log2 intensity –
overall median
2.5
0.0
−2.5
−50
0
50
100
150
Retention time (min)

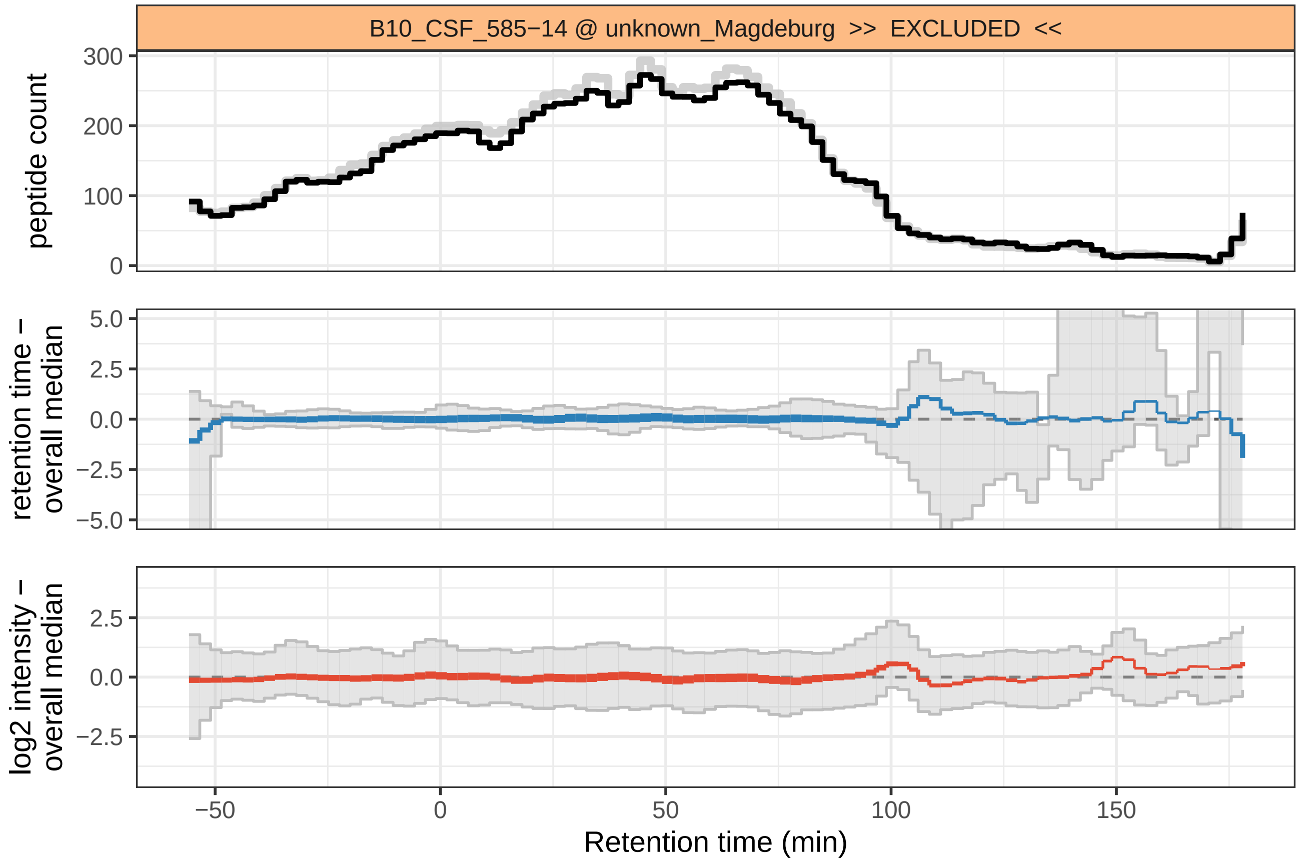

B10_CSF_585−14 @ unknown_Magdeburg >> EXCLUDED <<
peptide count
retention time – overall median
log2 intensity – overall median
Retention time (min)

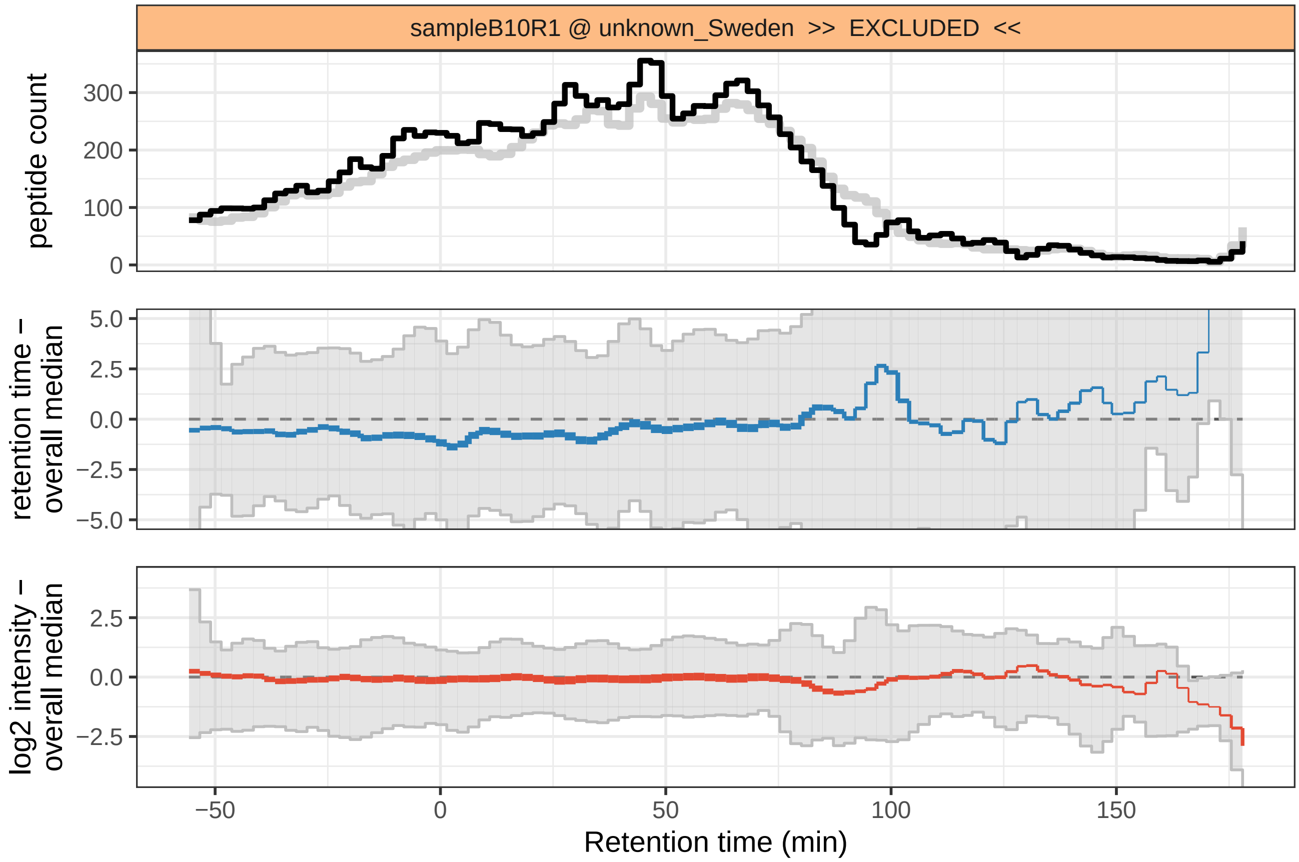

sampleB10R1 @ unknown_Sweden >> EXCLUDED <<
peptide count
retention time – overall median
log2 intensity – overall median
Retention time (min)

1.6 variation among replicates

The reproducibility of replicate measurements is expressed in three different analyses. First, the difference between peptide intensities in each sample are compared to the mean value among all replicates (foldchange distributions). Next, the Coefficient of Variation (CoV) is used as a metric for reproducibility to explore how much the CoV within a sample group can be improved by removing a single sample (eg; if CoV strongly improved after removing sample s, it could be regarded as an outlier). Finally, the CoV within each sample group is visualized as a boxplot and a violin plot, figures commonly seen in proteomics literature and useful for comparing across experiments (of similar protocol).

1.6.1 within-group foldchange distributions

The foldchange of all peptides in a sample is compared to their respective mean value over all samples in the group. This visualizes how strongly each sample deviates from other samples in the same group which helps identify outlier samples. The same data was used as detailed in the "retention time" section above.

For each sample group, two plots are shown: 1) a basic monochrome plot and 2) a variant that color-codes the top10 'worst' samples based on the standard deviation (sd) of their respective distributions. Note; per sample, the 0.5% quantiles of both top- and bottom-most outliers are disregarded in sd computation. The legend is sorted in column-first descending order (sample with highest sd in column 1 row 1, sample with second highest sd in column 1 row 2, etc.). If there are 10 or fewer samples, all are color-coded. On the pages after the plots, these sd values are shown as tables.

The 'input' panel is based on the peptide intensities as-is (i.e. the user-provided input data from upstream software), the 'normalized' panel shows the exact same samples and peptides after normalization (as specified by user). Samples marked as 'exclude' in the provided sample metadata table are visualized as dashed lines.

info: skipping within-group foldchange plots for sample group ‘unknown_Kiel’, require at least 2 replicates info: skipping within-group foldchange plots for sample group ‘unknown_Sweden’, require at least 2 replicates

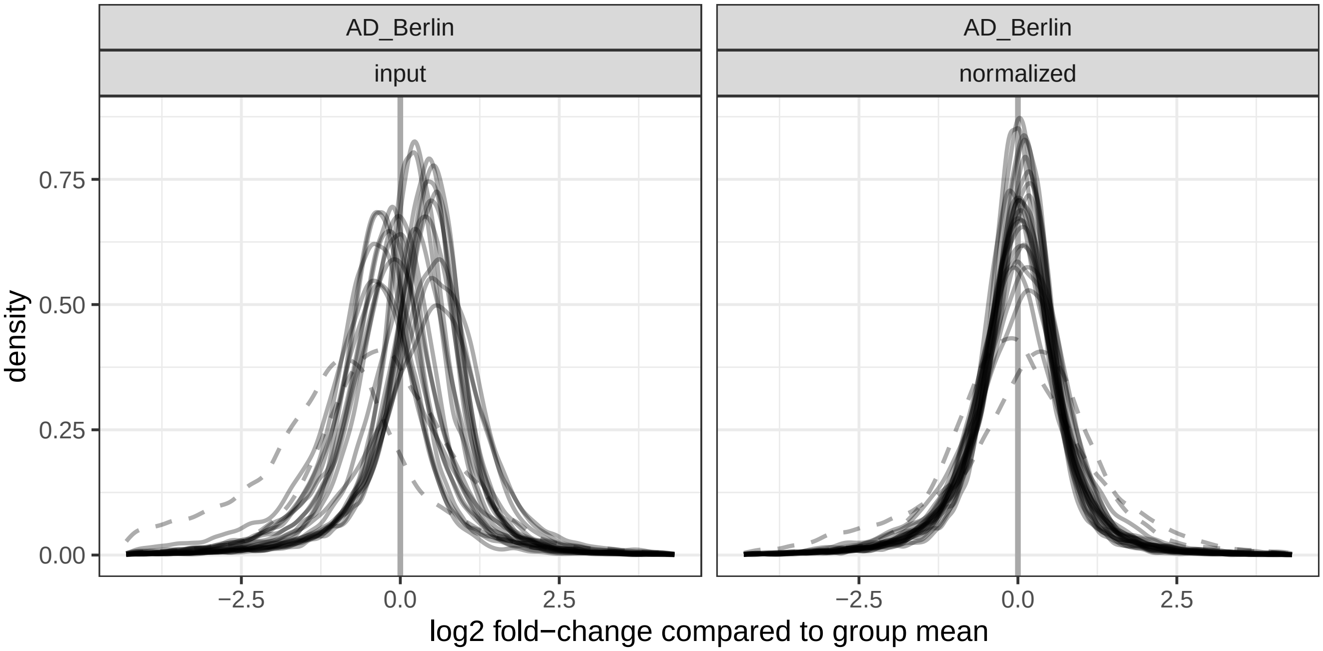

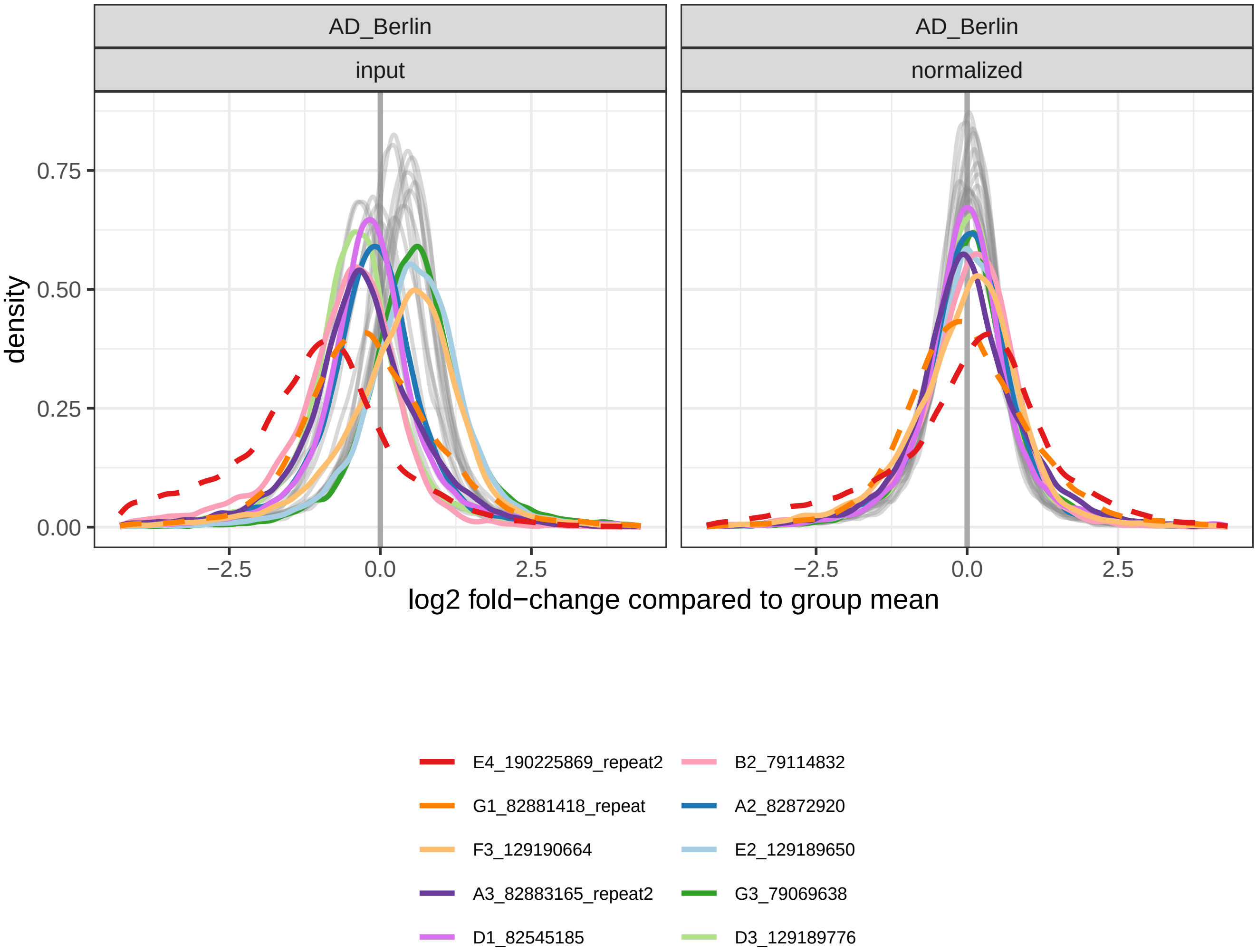

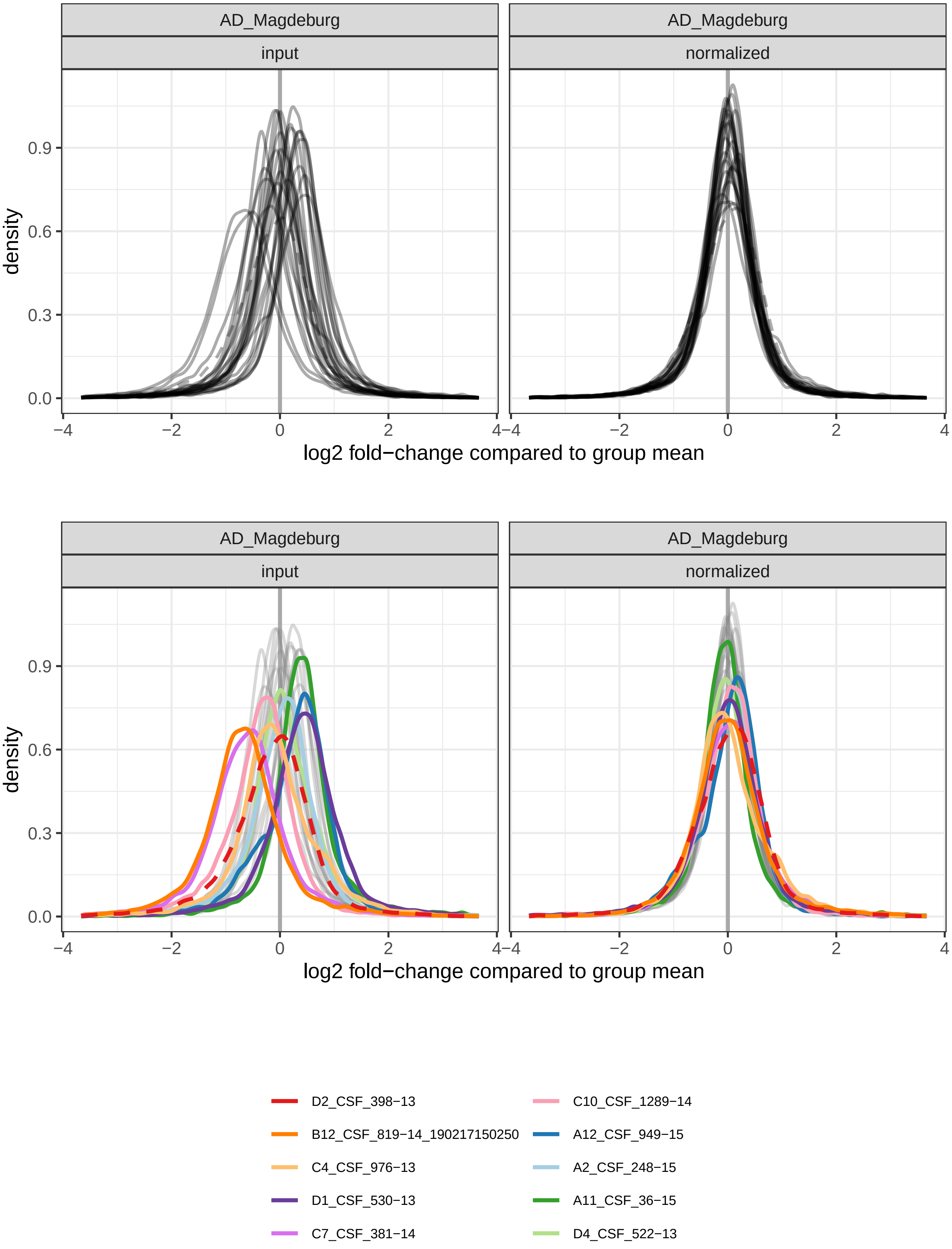
AD_Magdeburg
input
AD_Magdeburg
normalized
density
0.9
0.6
0.3
0.0
−4
−2
0
2
4
log2 fold−change compared to group mean
AD_Magdeburg
input
AD_Magdeburg
normalized
density
log2 fold−change compared to group mean
D2_CSF_398−13
B12_CSF_819−14_190217150250
C4_CSF_976−13
D1_CSF_530−13
C7_CSF_381−14
C10_CSF_1289−14
A12_CSF_949−15
A2_CSF_248−15
A11_CSF_36−15
D4_CSF_522−13

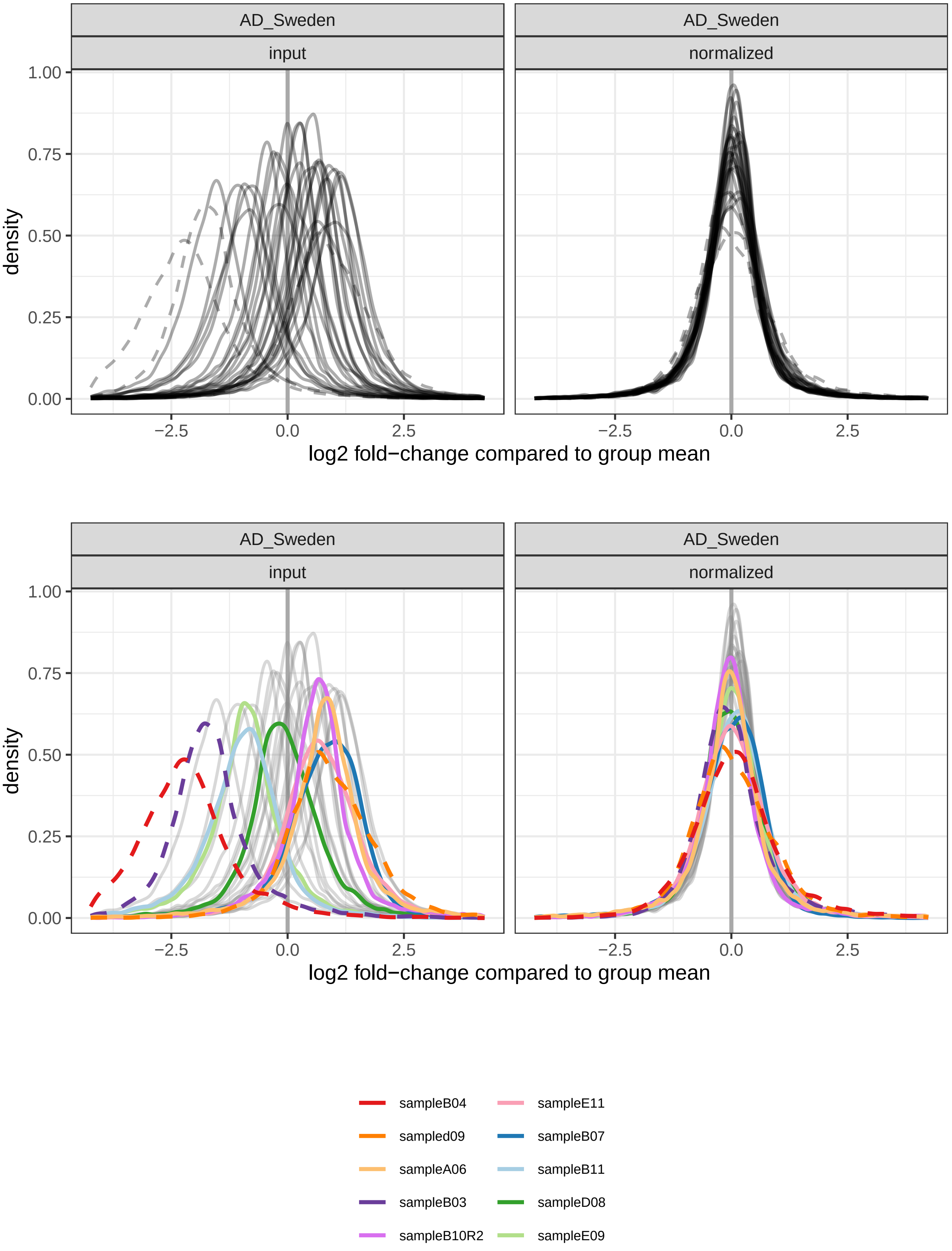
AD_Sweden
input
AD_Sweden
normalized
density
1.00
0.75
0.50
0.25
0.00
−2.5
0.0
2.5
log2 fold−change compared to group mean
sampleB04
sampled09
sampleA06
sampleB03
sampleB10R2
sampleE11
sampleB07
sampleB11
sampleD08
sampleE09

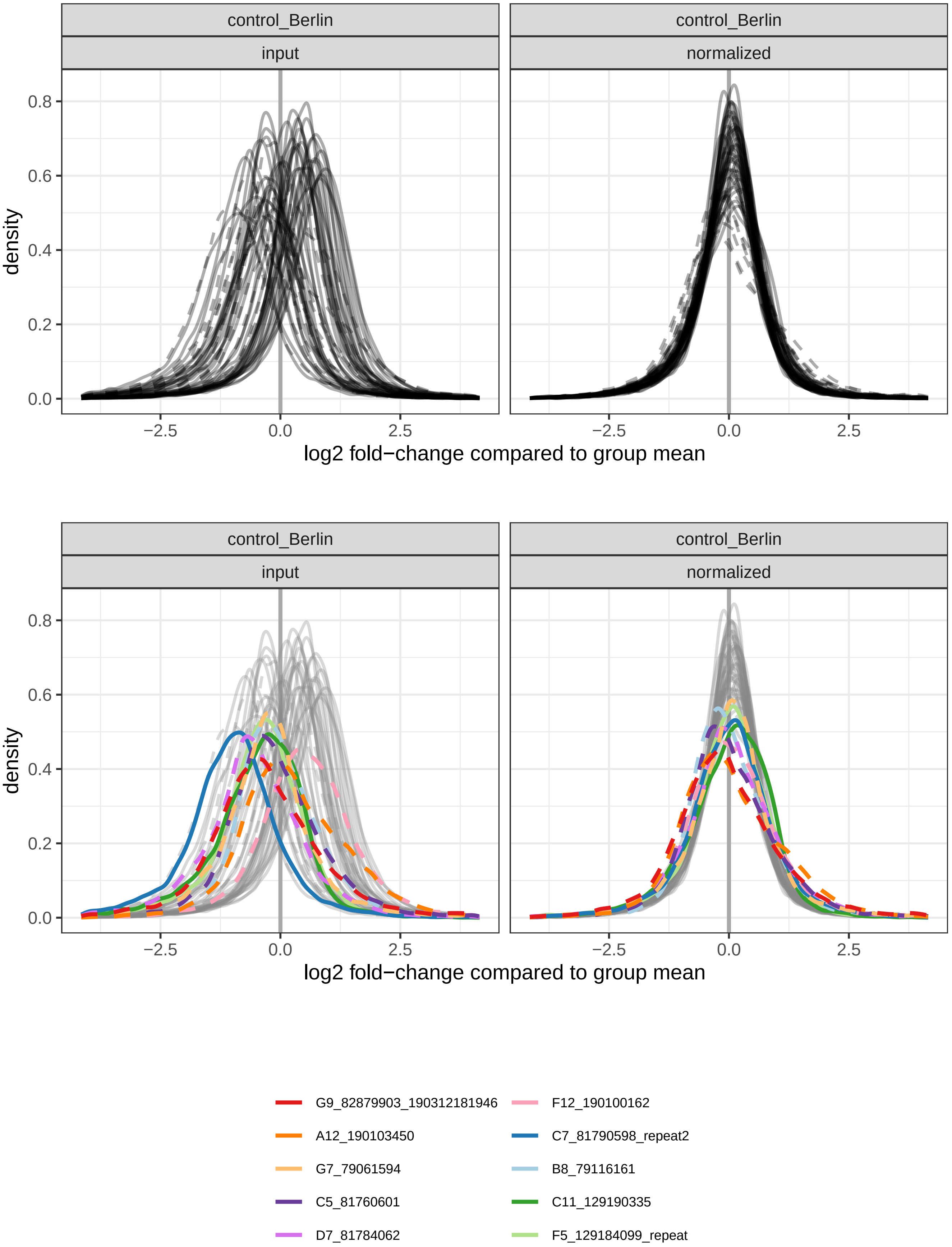
control_Berlin
input
control_Berlin
normalized
density
0.0
0.2
0.4
0.6
0.8
−2.5
0.0
2.5
log2 fold−change compared to group mean
control_Berlin
input
control_Berlin
normalized
density
0.0
0.2
0.4
0.6
0.8
−2.5
0.0
2.5
log2 fold−change compared to group mean
G9_82879903_190312181946
A12_190103450
G7_79061594
C5_81760601
D7_81784062
F12_190100162
C7_81790598_repeat2
B8_79116161
C11_129190335
F5_129184099_repeat

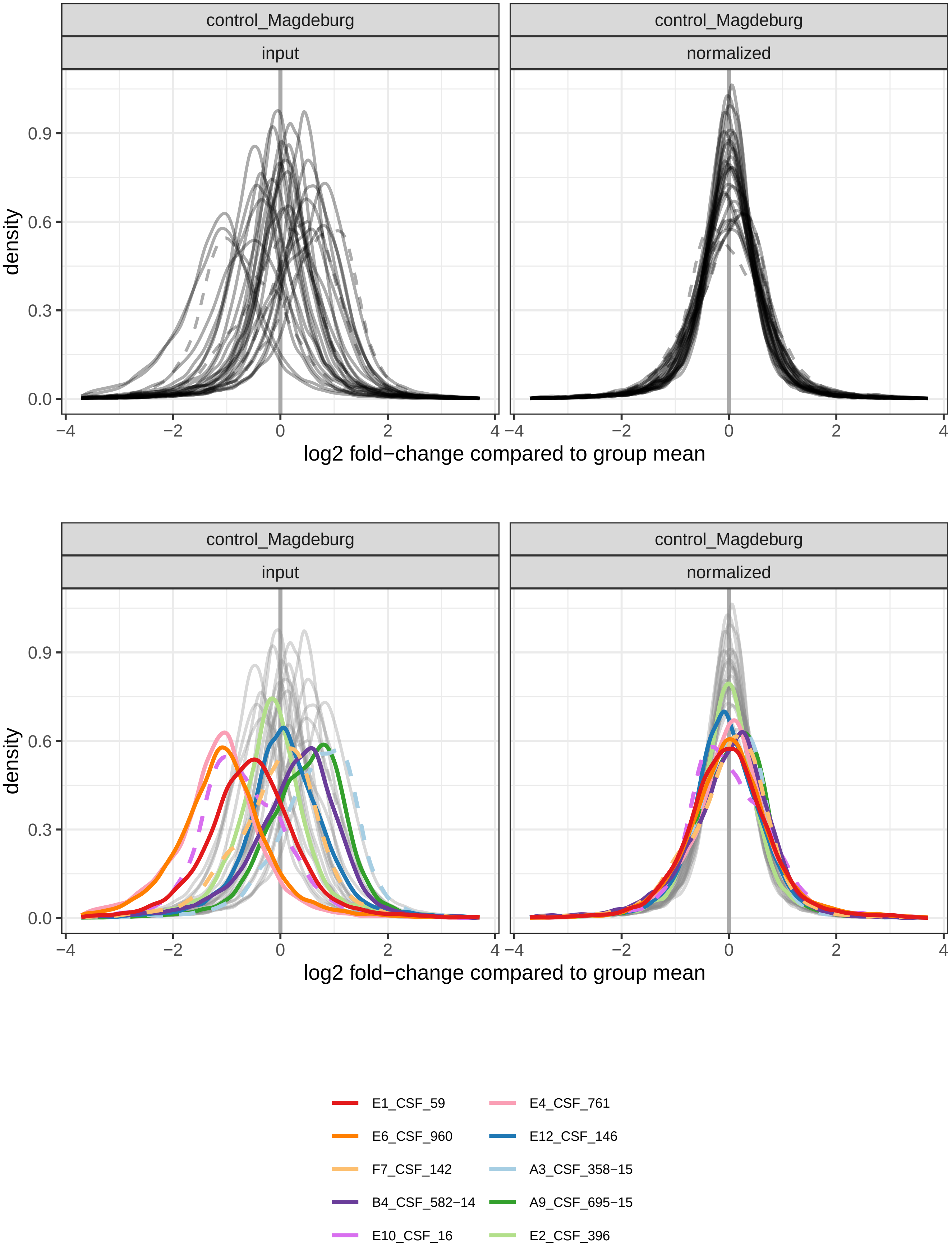
control_Magdeburg
input
control_Magdeburg
normalized
density
0.0
0.3
0.6
0.9
−4
−2
0
2
4
log2 fold−change compared to group mean
control_Magdeburg
input
control_Magdeburg
normalized
density
log2 fold−change compared to group mean
E1_CSF_59
E6_CSF_960
F7_CSF_142
B4_CSF_582−14
E10_CSF_16
E4_CSF_761
E12_CSF_146
A3_CSF_358−15
A9_CSF_695−15
E2_CSF_396

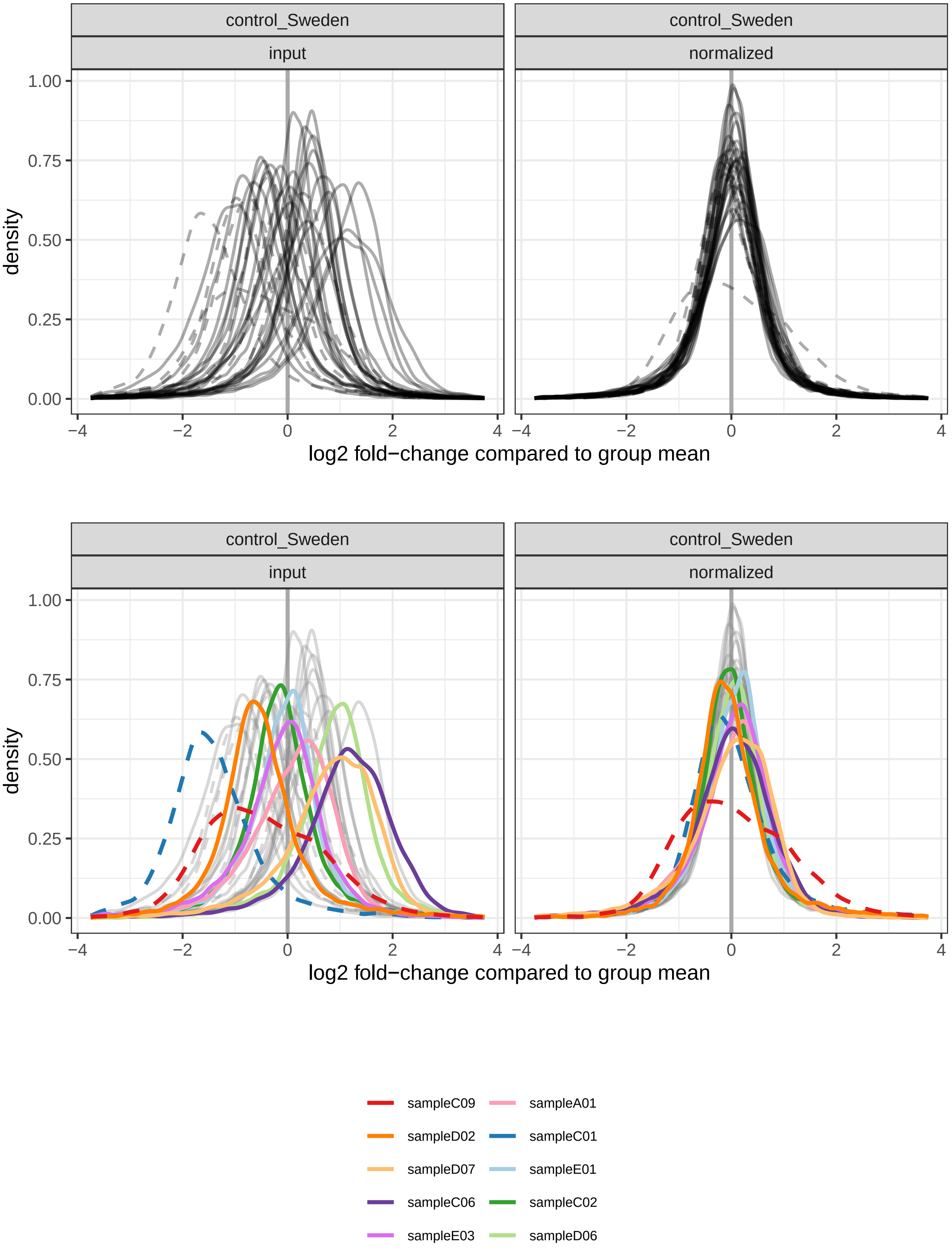
control_Sweden
input
control_Sweden
normalized
density
1.00
0.75
0.50
0.25
0.00
−4
−2
0
2
4
log2 fold−change compared to group mean
sampleC09
sampleA01
sampleD02
sampleC01
sampleD07
sampleE01
sampleC06
sampleC02
sampleE03
sampleD06

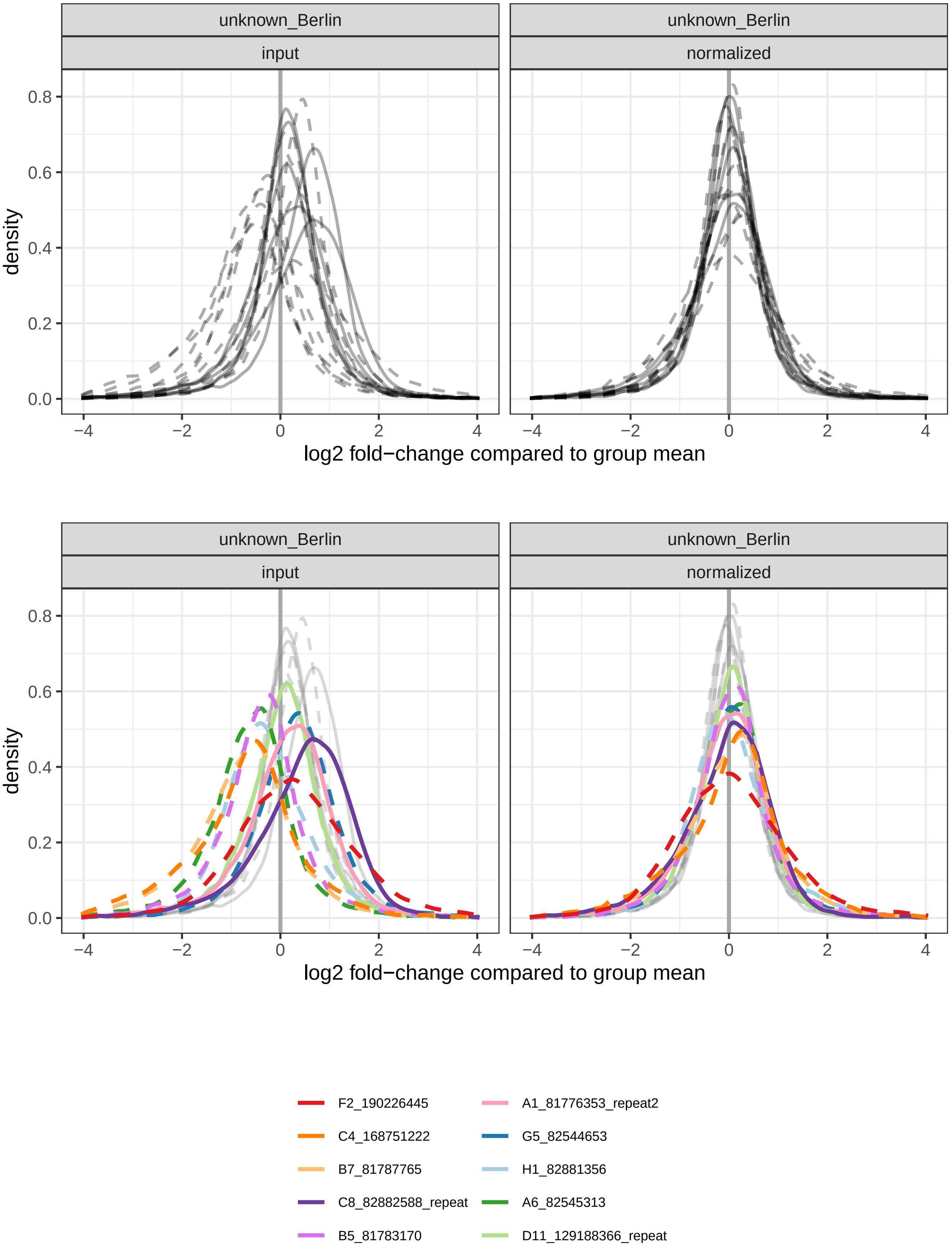
unknown_Berlin
input
unknown_Berlin
normalized
density
log2 fold−change compared to group mean
F2_190226445
C4_168751222
B7_81787765
C8_82882588_repeat
B5_81783170
A1_81776353_repeat2
G5_82544653
H1_82881356
A6_82545313
D11_129188366_repeat

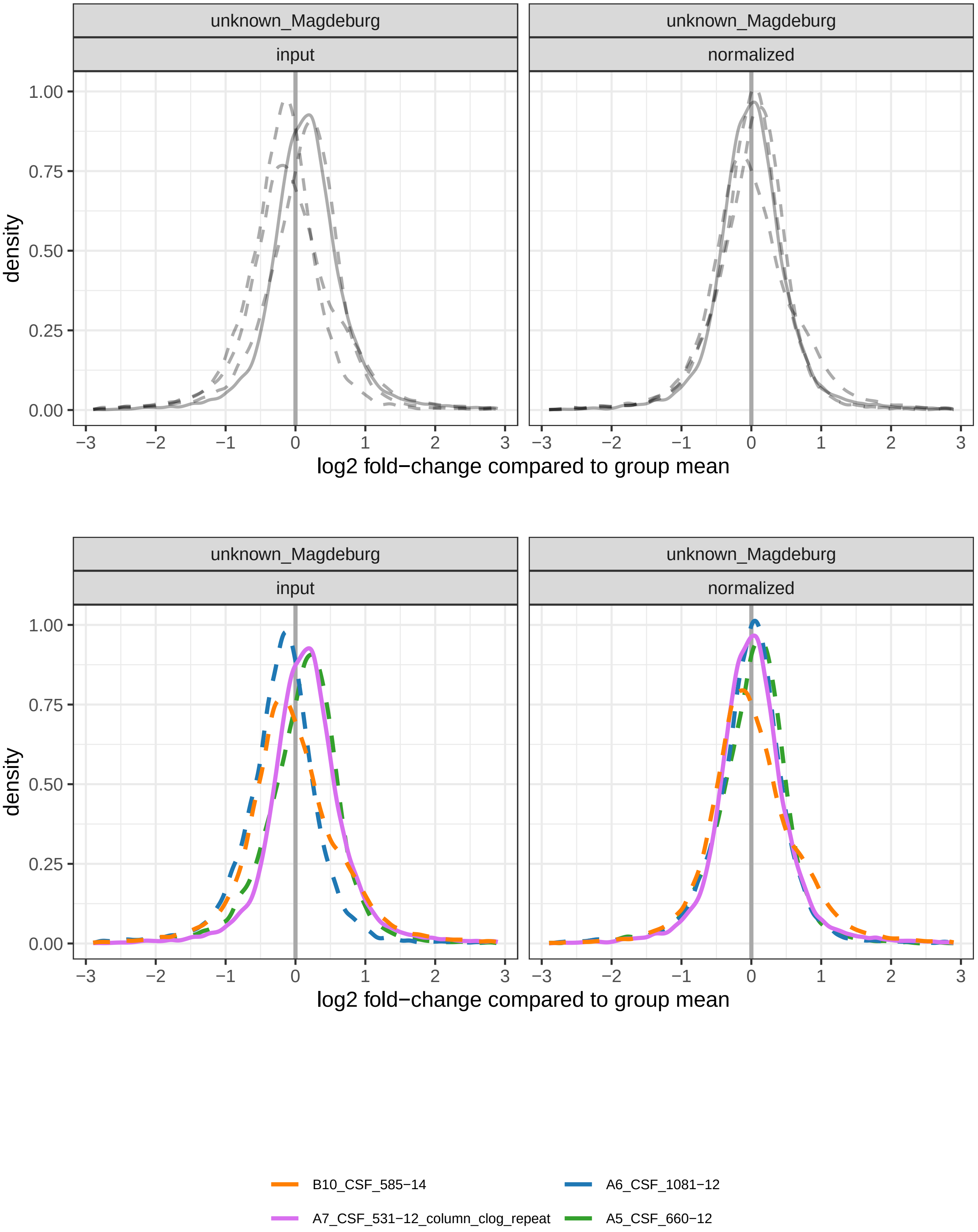
unknown_Magdeburg
input
unknown_Magdeburg
normalized
density
1.00
0.75
0.50
0.25
0.00
−3
−2
−1
0
1
2
3
log2 fold−change compared to group mean
B10_CSF_585−14
A6_CSF_1081−12
A7_CSF_531−12_column_clog_repeat
A5_CSF_660−12

top10 outliers per sample group

Group	Sample	Standard Deviation
AD_Berlin	F4_190100053	0.729
AD_Berlin	D4_168751414	0.728
AD_Berlin	E3_82871060_repeat	0.727
AD_Berlin	B1_129186923	0.703
AD_Berlin	G4_190101656_repeat	0.695
AD_Berlin	D2_79110561	0.687
AD_Berlin	A4_190104103	0.668
AD_Berlin	B3_79113129	0.657
AD_Berlin	G2_168751345	0.656
AD_Berlin	C3_82874751	0.635

Group	Sample	Standard Deviation
AD_Magdeburg	B11_CSF_506-14	0.589
AD_Magdeburg	D5_CSF_503-13	0.578
AD_Magdeburg	C3_CSF_974-14	0.570
AD_Magdeburg	B7_CSF_496-15	0.570
AD_Magdeburg	C6_CSF_841-14	0.567
AD_Magdeburg	C1_CSF_656-15	0.564
AD_Magdeburg	D3_CSF_863-13	0.563
AD_Magdeburg	C2_CSF_1239-14	0.556
AD_Magdeburg	D6_CSF_864-13	0.553
AD_Magdeburg	C8_CSF_840-14	0.535

Group	Sample	Standard Deviation
AD_Sweden	sampleA02	0.715
AD_Sweden	sampleA12	0.705
AD_Sweden	sampleE10	0.699
AD_Sweden	sampleA11	0.690
AD_Sweden	sampleA04	0.680
AD_Sweden	sampleE08	0.674
AD_Sweden	sampleE12	0.674
AD_Sweden	sampleB09	0.673
AD_Sweden	sampleA09	0.659
AD_Sweden	sampleC05	0.628

Group	Sample	Standard Deviation
control_Berlin	C12_168743858_repeat	0.727
control_Berlin	H5_82544762_190309202008	0.723
control_Berlin	D10_190224386	0.718
control_Berlin	G6_168744541	0.715
control_Berlin	A11_129183842	0.710
control_Berlin	C6_82881358	0.710
control_Berlin	D9_82885545_repeat	0.703
control_Berlin	B10_79069372	0.703
control_Berlin	G12_190225664	0.687
control_Berlin	F8_190224717_repeat	0.680

Group	Sample	Standard Deviation
control_Magdeburg	B1_CSF_326-15	0.640
control_Magdeburg	C12_CSF_1299-14	0.624
control_Magdeburg	A10_CSF_201-15	0.614
control_Magdeburg	F2_CSF_755	0.612
control_Magdeburg	F9_CSF_420	0.606
control_Magdeburg	C5_CSF_706-14	0.603
control_Magdeburg	F4_CSF_51	0.585
control_Magdeburg	F3_CSF_865	0.574
control_Magdeburg	F8_CSF_143	0.564
control_Magdeburg	B9_CSF_851-15	0.562

Group	Sample	Standard Deviation
control_Sweden	sampleD04	0.715
control_Sweden	sampleC12	0.712
control_Sweden	sampleD03	0.703
control_Sweden	sampleB06	0.696
control_Sweden	sampleC10	0.689
control_Sweden	sampleE04	0.686
control_Sweden	sampleE05	0.667
control_Sweden	sampleD10	0.662
control_Sweden	sampleC08	0.629
control_Sweden	sampleD12	0.623

Group	Sample	Standard Deviation
unknown_Berlin	G5_82544653	0.867
unknown_Berlin	H1_82881356	0.865
unknown_Berlin	A6_82545313	0.840
unknown_Berlin	D11_129188366_repeat	0.800
unknown_Berlin	F9_82880110_190304154132	0.747
unknown_Berlin	B4_190223925	0.713
unknown_Berlin	F11_82542649	0.710
unknown_Berlin	D5_129186580_repeat	0.693
unknown_Berlin	B11_129182819	0.662
unknown_Berlin	C2_79070601	0.662

Group	Sample	Standard Deviation
unknown_Magdeburg	B10_CSF_585-14	0.632
unknown_Magdeburg	A7_CSF_531-12_column_clog_repeat	0.522
unknown_Magdeburg	A6_CSF_1081-12	0.519
unknown_Magdeburg	A5_CSF_660-12	0.519

1.6.2 CoV, leave-one-out

The figures below describe the effect of removing a particular sample prior to within-group Coefficient of Variation (CoV) computation. The lower the CoV distribution is for a sample, the better reproducibility we get by excluding it. Only sample groups with at least 4 replicates can be used for this analysis, so 3 samples remain after leaving one out. Samples marked as 'exclude' in the provided sample metadata are included in these analyses (shown as dashed lines), and only peptides with at least 3 data points across replicate samples (after leave-one-out) are used for each CoV computation.

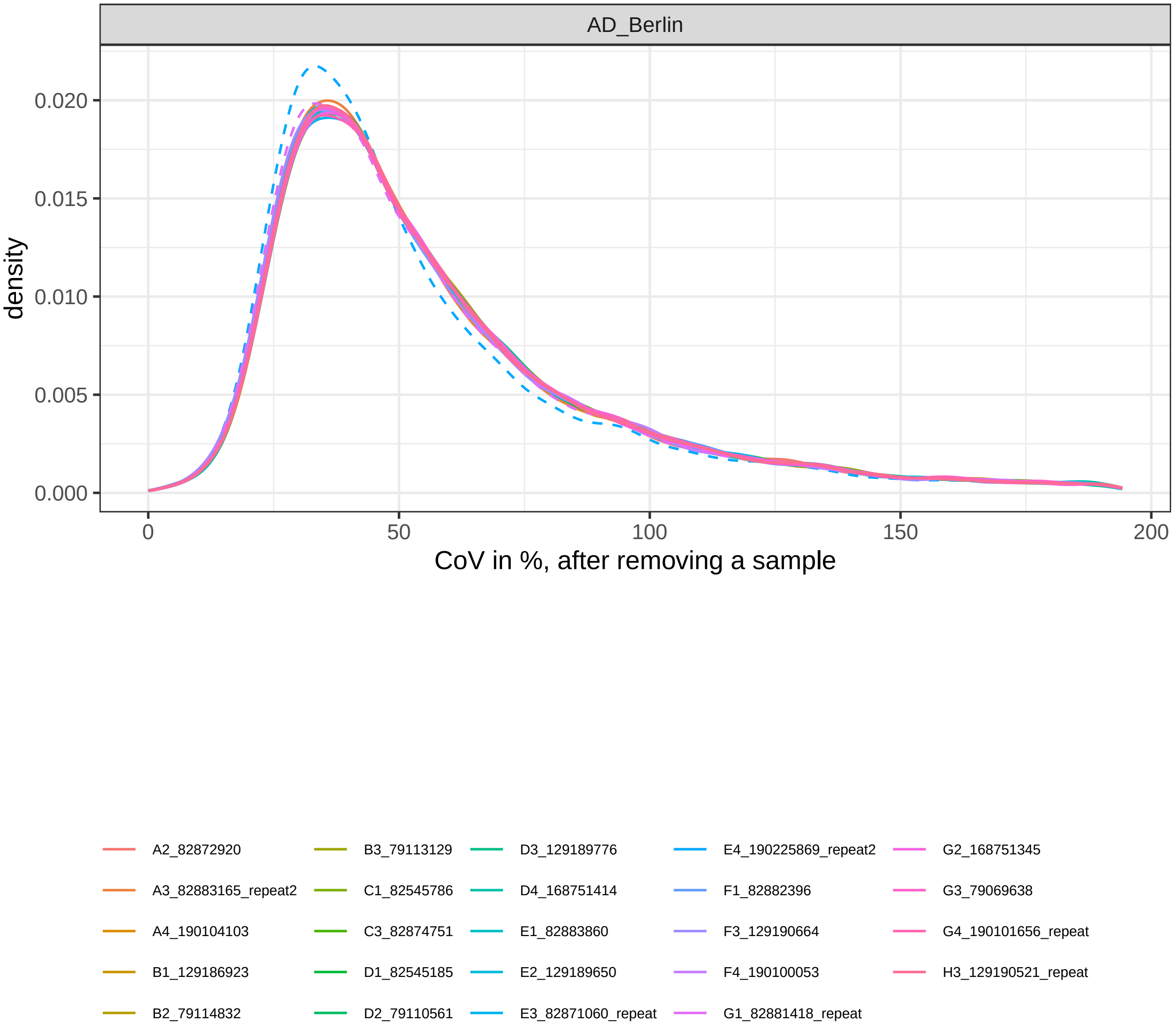

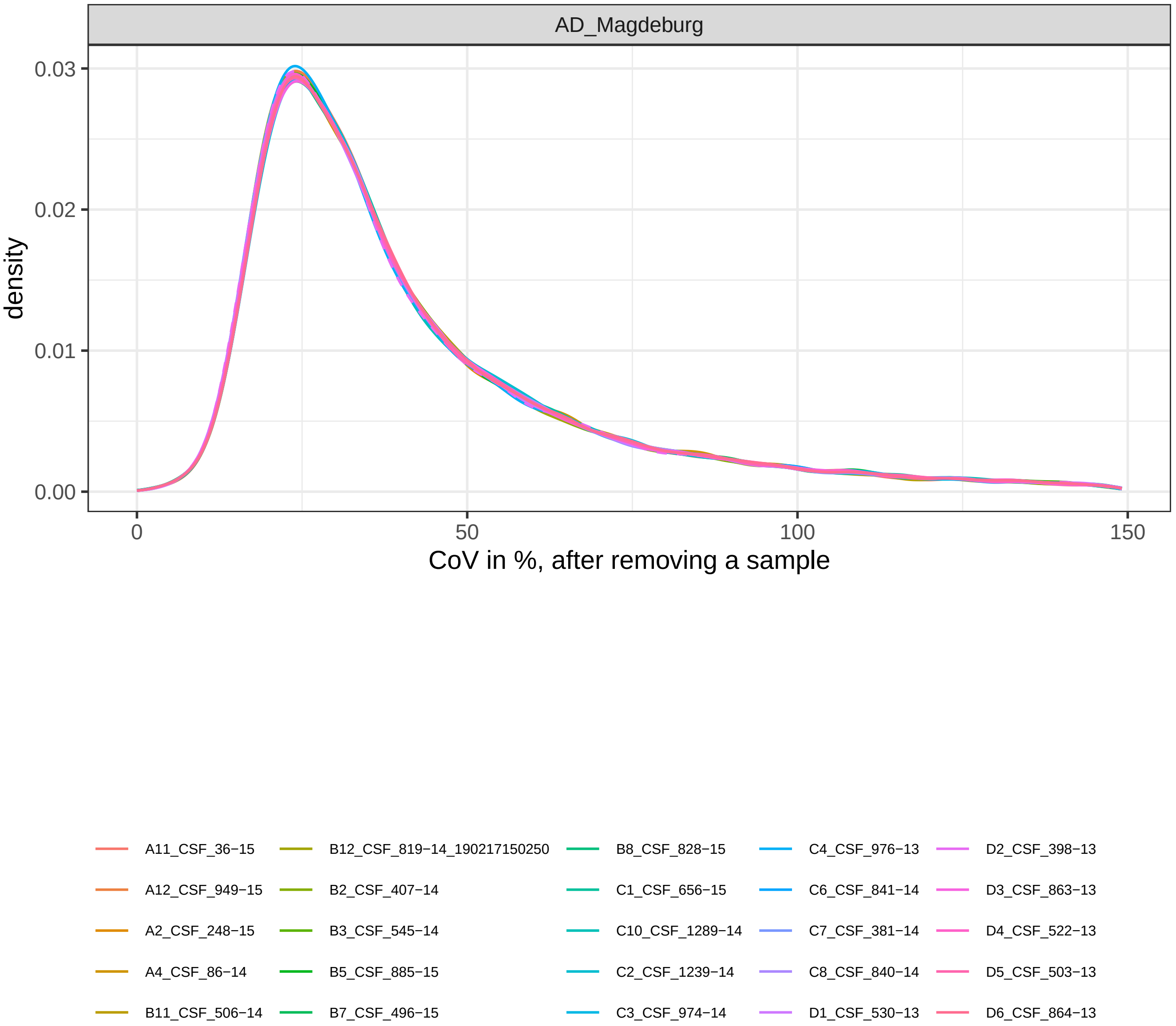
AD_Magdeburg
density
0.03
0.02
0.01
0.00
0
50
100
150
CoV in %, after removing a sample
A11_CSF_36−15
A12_CSF_949−15
A2_CSF_248−15
A4_CSF_86−14
B11_CSF_506−14
B12_CSF_819−14_190217150250
B2_CSF_407−14
B3_CSF_545−14
B5_CSF_885−15
B7_CSF_496−15
B8_CSF_828−15
C1_CSF_656−15
C10_CSF_1289−14
C2_CSF_1239−14
C3_CSF_974−14
C4_CSF_976−13
C6_CSF_841−14
C7_CSF_381−14
C8_CSF_840−14
D1_CSF_530−13
D2_CSF_398−13
D3_CSF_863−13
D4_CSF_522−13
D5_CSF_503−13
D6_CSF_864−13

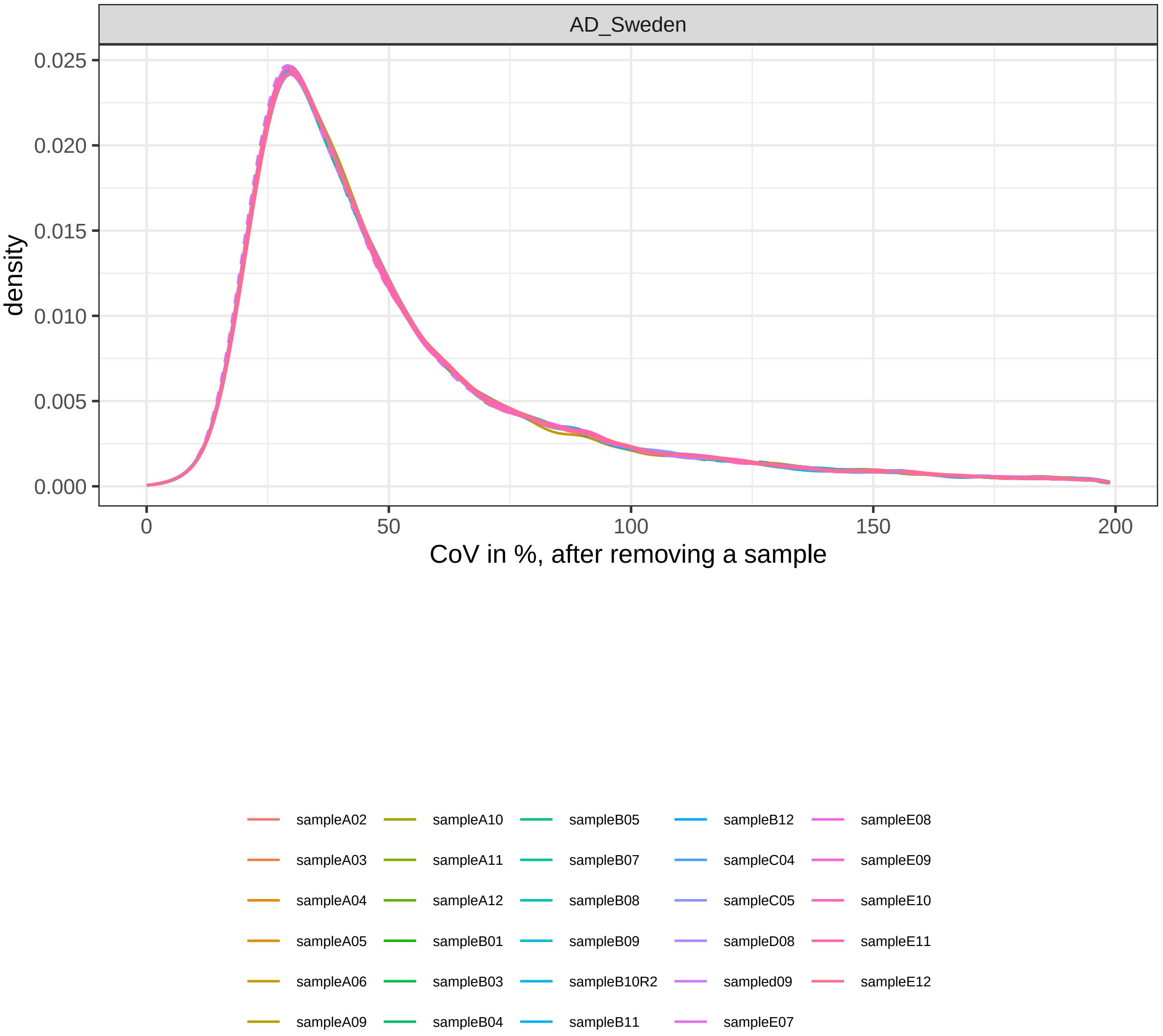
AD_Sweden
density
0.025
0.020
0.015
0.010
0.005
0.000
0
50
100
150
200
CoV in %, after removing a sample
sampleA02
sampleA03
sampleA04
sampleA05
sampleA06
sampleA09
sampleA10
sampleA11
sampleA12
sampleB01
sampleB03
sampleB04
sampleB05
sampleB07
sampleB08
sampleB09
sampleB10R2
sampleB11
sampleB12
sampleC04
sampleC05
sampleD08
sampled09
sampleE07
sampleE08
sampleE09
sampleE10
sampleE11
sampleE12

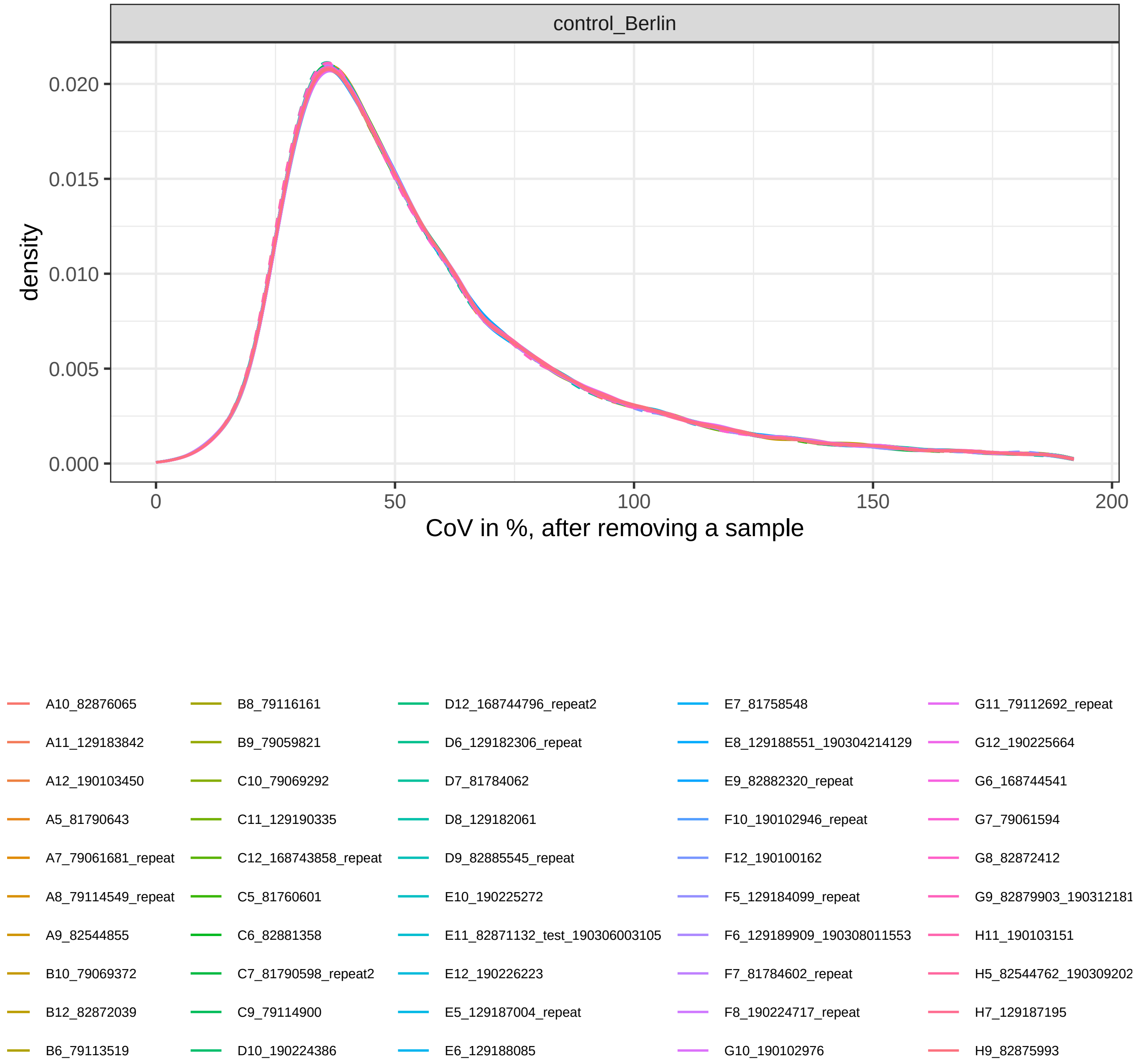
control_Berlin
density
0.020
0.015
0.010
0.005
0.000
0
50
100
150
200
CoV in %, after removing a sample
A10_82876065
A11_129183842
A12_190103450
A5_81790643
A7_79061681_repeat
A8_79114549_repeat
A9_82544855
B10_79069372
B12_82872039
B6_79113519
B8_79116161
B9_79059821
C10_79069292
C11_129190335
C12_168743858_repeat
C5_81760601
C6_82881358
C7_81790598_repeat2
C9_79114900
D10_190224386
D12_168744796_repeat2
D6_129182306_repeat
D7_81784062
D8_129182061
D9_82885545_repeat
E10_190225272
E11_82871132_test_190306003105
E12_190226223
E5_129187004_repeat
E6_129188085
E7_81758548
E8_129188551_190304214129
E9_82882320_repeat
F10_190102946_repeat
F12_190100162
F5_129184099_repeat
F6_129189909_190308011553
F7_81784602_repeat
F8_190224717_repeat
G10_190102976
G11_79112692_repeat
G12_190225664
G6_168744541
G7_79061594
G8_82872412
G9_82879903_190312181
H11_190103151
H5_82544762_190309202
H7_129187195
H9_82875993

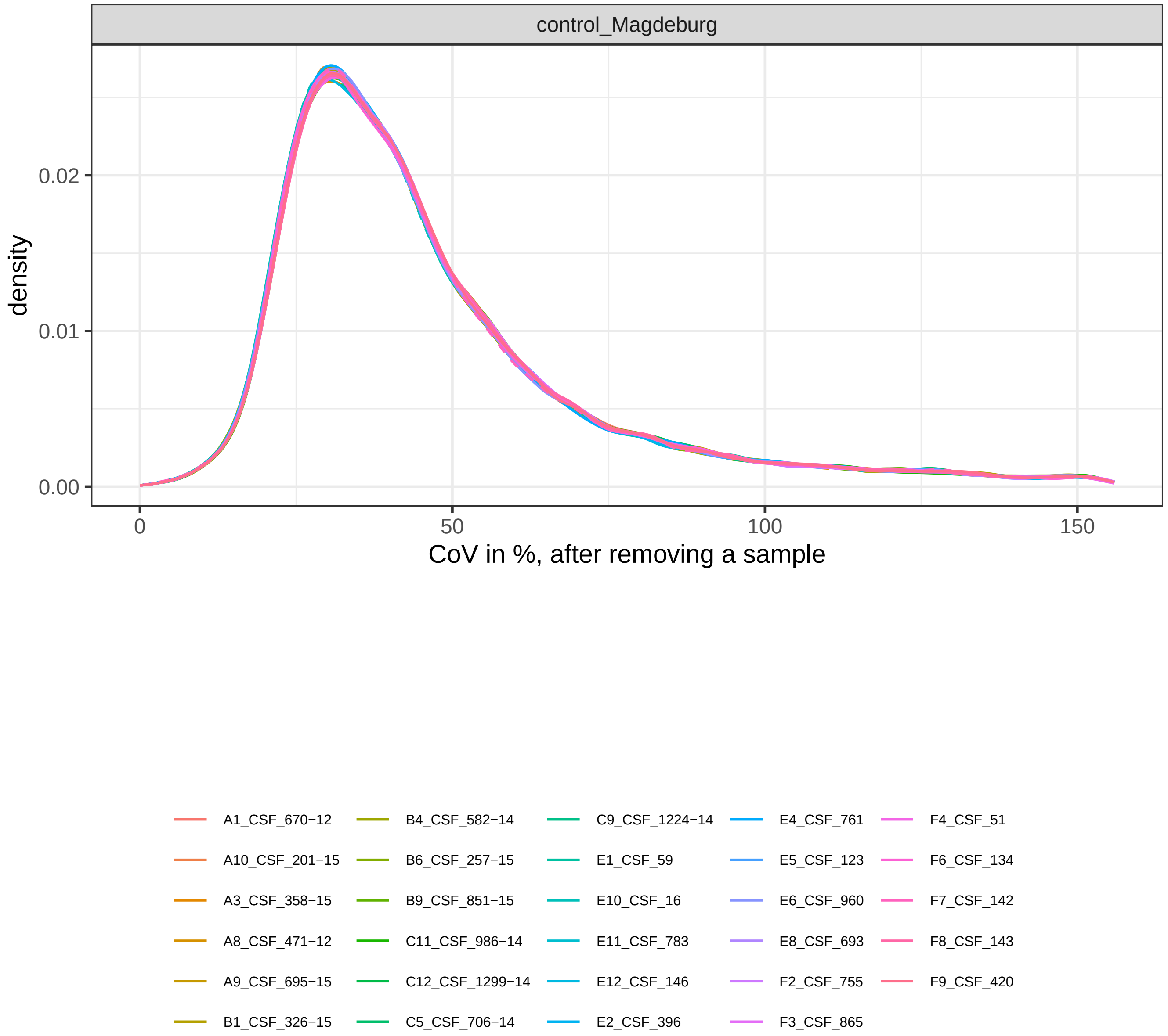

control_Magdeburg
density
0.02
0.01
0.00
0
50
100
150
CoV in %, after removing a sample
A1_CSF_670−12
A10_CSF_201−15
A3_CSF_358−15
A8_CSF_471−12
A9_CSF_695−15
B1_CSF_326−15
B4_CSF_582−14
B6_CSF_257−15
B9_CSF_851−15
C11_CSF_986−14
C12_CSF_1299−14
C5_CSF_706−14
C9_CSF_1224−14
E1_CSF_59
E10_CSF_16
E11_CSF_783
E12_CSF_146
E2_CSF_396
E4_CSF_761
E5_CSF_123
E6_CSF_960
E8_CSF_693
F2_CSF_755
F3_CSF_865
F4_CSF_51
F6_CSF_134
F7_CSF_142
F8_CSF_143
F9_CSF_420

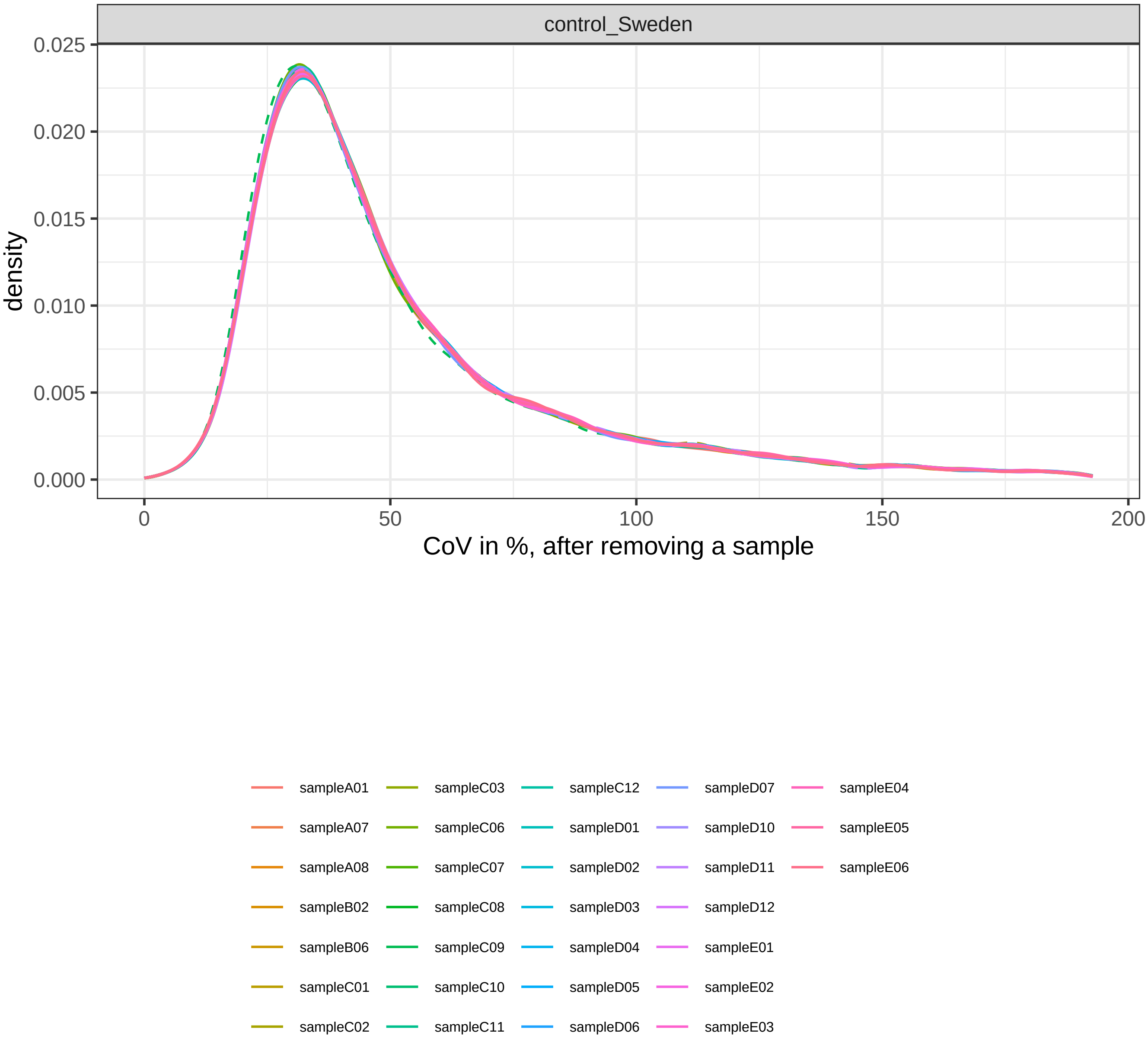
control_Sweden
density
0.025
0.020
0.015
0.010
0.005
0.000
0
50
100
150
200
CoV in %, after removing a sample
sampleA01
sampleA07
sampleA08
sampleB02
sampleB06
sampleC01
sampleC02
sampleC03
sampleC06
sampleC07
sampleC08
sampleC09
sampleC10
sampleC11
sampleC12
sampleD01
sampleD02
sampleD03
sampleD04
sampleD05
sampleD06
sampleD07
sampleD10
sampleD11
sampleD12
sampleE01
sampleE02
sampleE03
sampleE04
sampleE05
sampleE06

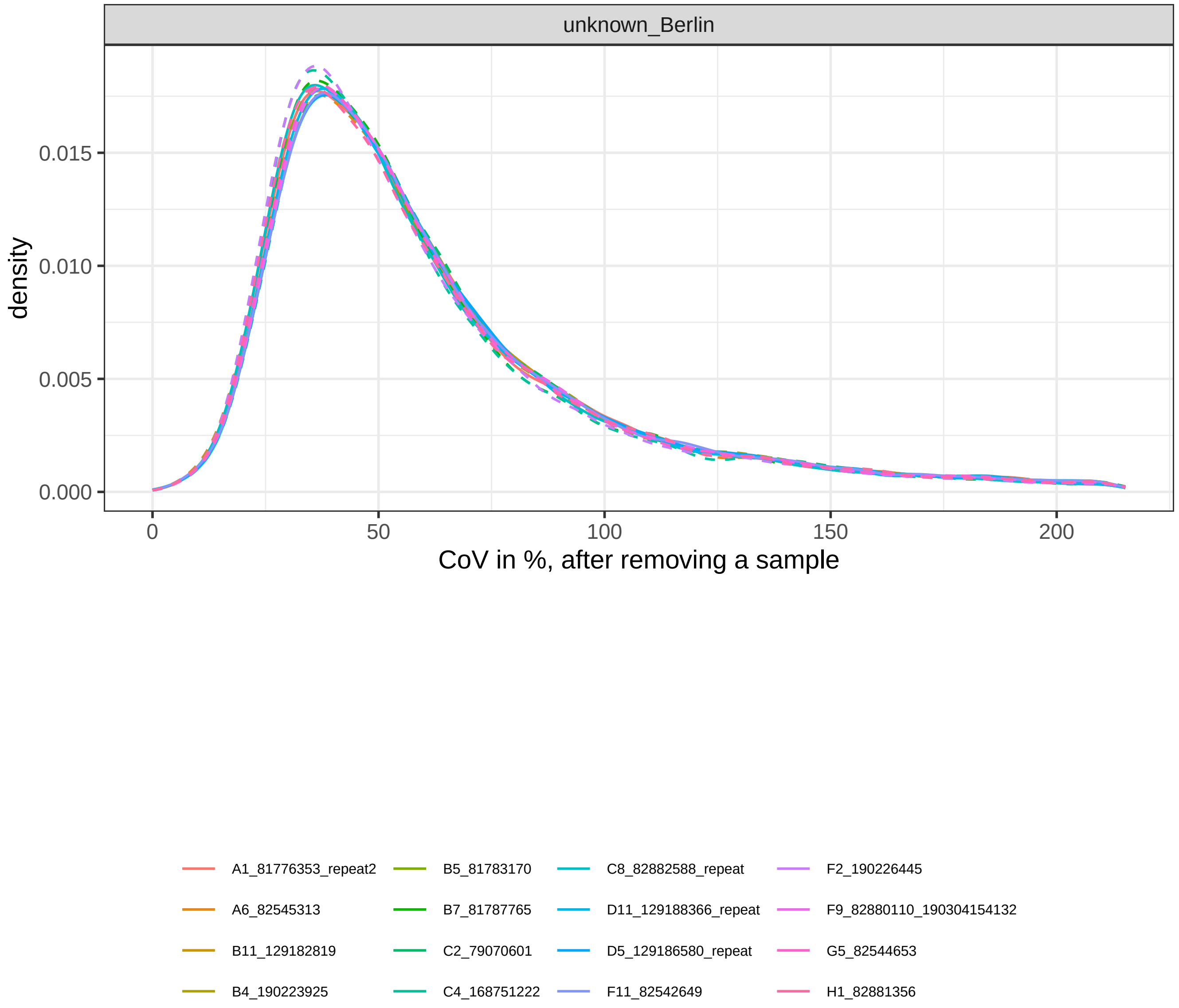
unknown_Berlin
density
0.015
0.010
0.005
0.000
0
50
100
150
200
CoV in %, after removing a sample
A1_81776353_repeat2
A6_82545313
B11_129182819
B4_190223925
B5_81783170
B7_81787765
C2_79070601
C4_168751222
C8_82882588_repeat
D11_129188366_repeat
D5_129186580_repeat
F11_82542649
F2_190226445
F9_82880110_190304154132
G5_82544653
H1_82881356

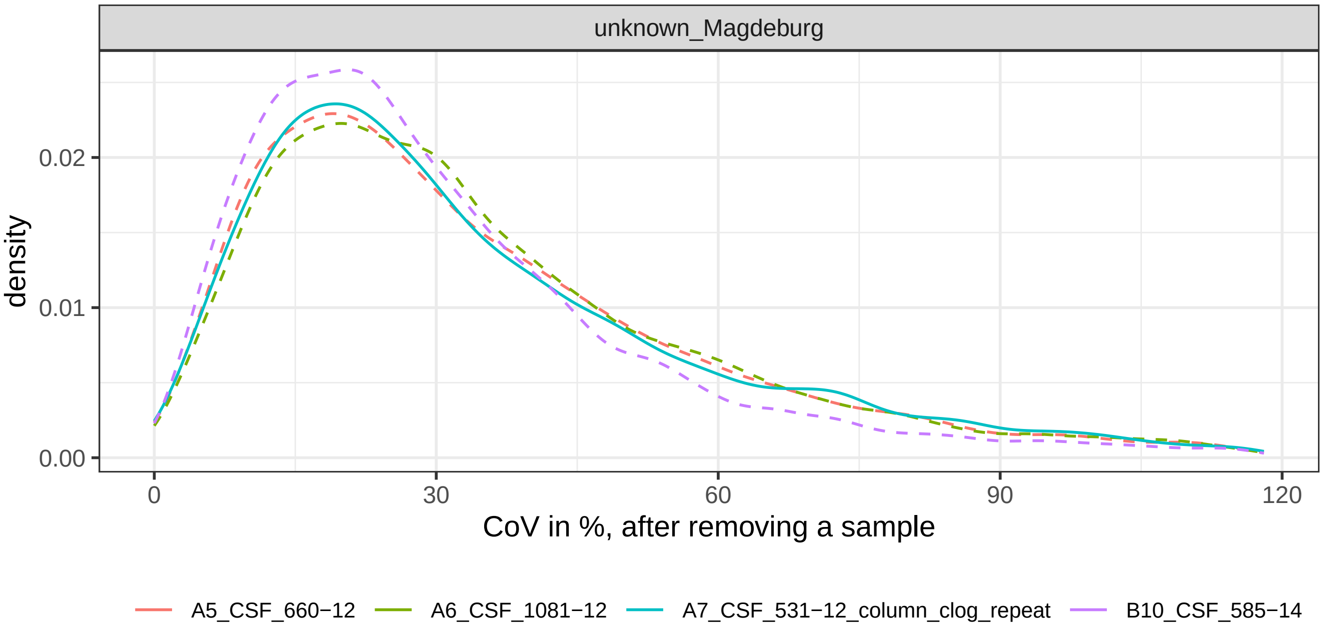
unknown_Magdeburg
density
0.00
0.01
0.02
0
30
60
90
120
CoV in %, after removing a sample
A5_CSF_660−12
A6_CSF_1081−12
A7_CSF_531−12_column_clog_repeat
B10_CSF_585−14

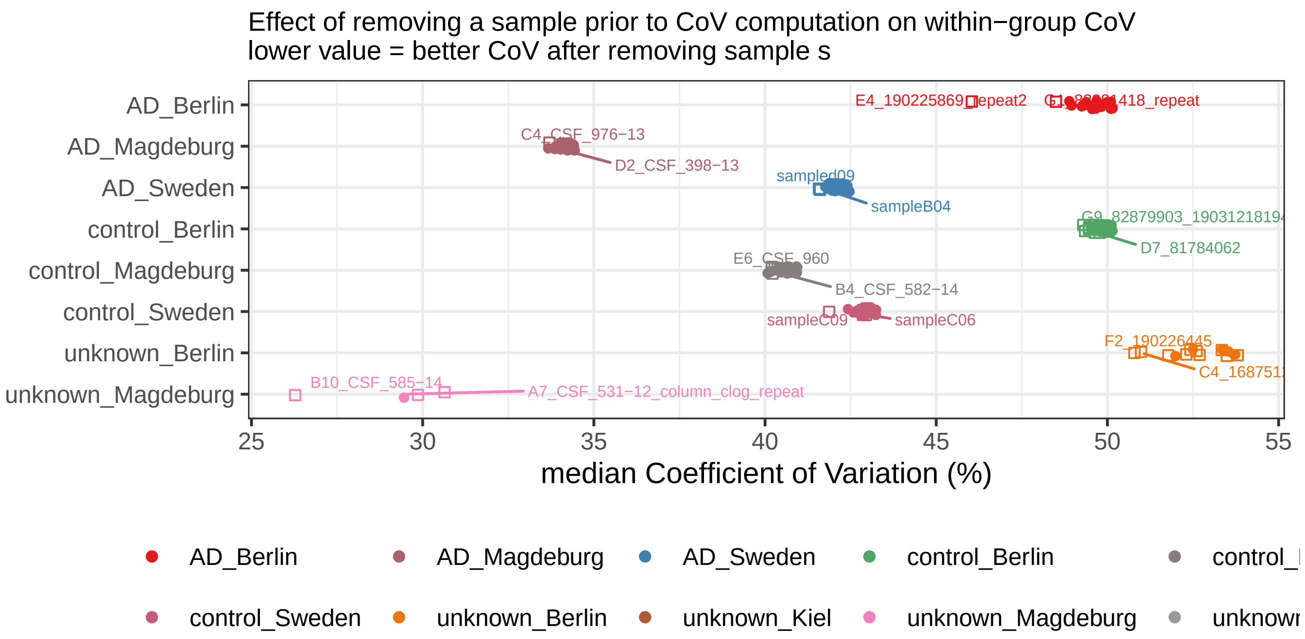
Effect of removing a sample prior to CoV computation on within−group CoV
lower value = better CoV after removing sample s
AD_Berlin
AD_Magdeburg
AD_Sweden
control_Berlin
control_Magdeburg
control_Sweden
unknown_Berlin
unknown_Magdeburg
C4_CSF_976−13
D2_CSF_398−13
sampleB04
D7_81784062
E6_CSF_960
B4_CSF_582−14
sampleC09
sampleC06
F2_190226445
B10_CSF_585−14
A7_CSF_531−12_column_clog_repeat
25
30
35
40
45
50
55
median Coefficient of Variation (%)
AD_Berlin
AD_Magdeburg
AD_Sweden
control_Berlin
control_Sweden
unknown_Berlin
unknown_Kiel
unknown_Magdeburg

shortname	group	exclude	median CoV
E4_190225869_repeat2	AD_Berlin	TRUE	46.0
G1_82881418_repeat	AD_Berlin	TRUE	48.5
F3_129190664	AD_Berlin	FALSE	48.9
A3_82883165_repeat2	AD_Berlin	FALSE	49.0
E2_129189650	AD_Berlin	FALSE	49.2
D1_82545185	AD_Berlin	FALSE	49.3
B2_79114832	AD_Berlin	FALSE	49.3
A2_82872920	AD_Berlin	FALSE	49.4
G3_79069638	AD_Berlin	FALSE	49.5
D3_129189776	AD_Berlin	FALSE	49.5
H3_129190521_repeat	AD_Berlin	FALSE	49.6
E1_82883860	AD_Berlin	FALSE	49.7
C1_82545786	AD_Berlin	FALSE	49.7
F4_190100053	AD_Berlin	FALSE	49.7
E3_82871060_repeat	AD_Berlin	FALSE	49.7
F1_82882396	AD_Berlin	FALSE	49.8
D4_168751414	AD_Berlin	FALSE	49.8
B1_129186923	AD_Berlin	FALSE	49.8
G4_190101656_repeat	AD_Berlin	FALSE	49.9
A4_190104103	AD_Berlin	FALSE	49.9
D2_79110561	AD_Berlin	FALSE	50.0
B3_79113129	AD_Berlin	FALSE	50.1
G2_168751345	AD_Berlin	FALSE	50.1
C3_82874751	AD_Berlin	FALSE	50.2
C4_CSF_976-13	AD_Magdeburg	FALSE	33.7
D2_CSF_398-13	AD_Magdeburg	TRUE	33.7
B12_CSF_819-14_190217150250	AD_Magdeburg	FALSE	33.9
C7_CSF_381-14	AD_Magdeburg	FALSE	33.9
A12_CSF_949-15	AD_Magdeburg	FALSE	33.9
A2_CSF_248-15	AD_Magdeburg	FALSE	34.0
C10_CSF_1289-14	AD_Magdeburg	FALSE	34.0
A11_CSF_36-15	AD_Magdeburg	FALSE	34.0
D4_CSF_522-13	AD_Magdeburg	FALSE	34.0
D1_CSF_530-13	AD_Magdeburg	FALSE	34.0
B3_CSF_545-14	AD_Magdeburg	FALSE	34.1
B5_CSF_885-15	AD_Magdeburg	FALSE	34.2
B2_CSF_407-14	AD_Magdeburg	FALSE	34.2
B8_CSF_828-15	AD_Magdeburg	FALSE	34.2
A4_CSF_86-14	AD_Magdeburg	FALSE	34.2
B11_CSF_506-14	AD_Magdeburg	FALSE	34.3
C6_CSF_841-14	AD_Magdeburg	FALSE	34.3
C3_CSF_974-14	AD_Magdeburg	FALSE	34.3
C8_CSF_840-14	AD_Magdeburg	FALSE	34.3
D5_CSF_503-13	AD_Magdeburg	FALSE	34.3
C1_CSF_656-15	AD_Magdeburg	FALSE	34.4
B7_CSF_496-15	AD_Magdeburg	FALSE	34.4
D3_CSF_863-13	AD_Magdeburg	FALSE	34.4
C2_CSF_1239-14	AD_Magdeburg	FALSE	34.4
D6_CSF_864-13	AD_Magdeburg	FALSE	34.4
sampled09	AD_Sweden	TRUE	41.6

shortname	group	exclude	median CoV
sampleB04	AD_Sweden	TRUE	41.6
sampleA06	AD_Sweden	FALSE	41.8
sampleE11	AD_Sweden	FALSE	41.8
sampleE09	AD_Sweden	FALSE	41.9
sampleB11	AD_Sweden	FALSE	41.9
sampleD08	AD_Sweden	FALSE	41.9
sampleB10R2	AD_Sweden	FALSE	42.0
sampleB03	AD_Sweden	TRUE	42.0
sampleB01	AD_Sweden	FALSE	42.0
sampleB07	AD_Sweden	FALSE	42.0
sampleA05	AD_Sweden	FALSE	42.1
sampleB08	AD_Sweden	FALSE	42.1
sampleB05	AD_Sweden	FALSE	42.1
sampleC04	AD_Sweden	FALSE	42.2
sampleA10	AD_Sweden	FALSE	42.2
sampleE07	AD_Sweden	FALSE	42.2
sampleA03	AD_Sweden	FALSE	42.2
sampleB12	AD_Sweden	FALSE	42.2
sampleA12	AD_Sweden	FALSE	42.3
sampleE10	AD_Sweden	FALSE	42.3
sampleA11	AD_Sweden	FALSE	42.3
sampleA02	AD_Sweden	FALSE	42.3
sampleA04	AD_Sweden	FALSE	42.3
sampleE08	AD_Sweden	FALSE	42.3
sampleA09	AD_Sweden	FALSE	42.4
sampleB09	AD_Sweden	FALSE	42.4
sampleC05	AD_Sweden	FALSE	42.4
sampleE12	AD_Sweden	FALSE	42.5
G9_82879903_190312181946	control_Berlin	TRUE	49.3
D7_81784062	control_Berlin	TRUE	49.3
G7_79061594	control_Berlin	TRUE	49.5
A12_190103450	control_Berlin	TRUE	49.5
C7_81790598_repeat2	control_Berlin	FALSE	49.5
C5_81760601	control_Berlin	TRUE	49.6
F12_190100162	control_Berlin	TRUE	49.6
F6_129189909_190308011553	control_Berlin	TRUE	49.6
C11_129190335	control_Berlin	FALSE	49.7
F10_190102946_repeat	control_Berlin	TRUE	49.7
F7_81784602_repeat	control_Berlin	FALSE	49.7
B8_79116161	control_Berlin	TRUE	49.7
G10_190102976	control_Berlin	FALSE	49.7
F5_129184099_repeat	control_Berlin	FALSE	49.7
B6_79113519	control_Berlin	FALSE	49.8
C9_79114900	control_Berlin	FALSE	49.8
E10_190225272	control_Berlin	TRUE	49.8
G8_82872412	control_Berlin	FALSE	49.8
A8_79114549_repeat	control_Berlin	FALSE	49.8
E8_129188551_190304214129	control_Berlin	FALSE	49.8
C10_79069292	control_Berlin	FALSE	49.8
E11_82871132_test_190306003105	control_Berlin	FALSE	49.8

shortname	group	exclude	median CoV
D12_168744796_repeat2	control_Berlin	TRUE	49.9
E7_81758548	control_Berlin	FALSE	49.9
A9_82544855	control_Berlin	FALSE	49.9
B12_82872039	control_Berlin	FALSE	49.9
D8_129182061	control_Berlin	TRUE	49.9
H11_190103151	control_Berlin	FALSE	49.9
E12_190226223	control_Berlin	FALSE	49.9
E6_129188085	control_Berlin	TRUE	49.9
C6_82881358	control_Berlin	FALSE	49.9
E9_82882320_repeat	control_Berlin	FALSE	49.9
H9_82875993	control_Berlin	FALSE	49.9
A7_79061681_repeat	control_Berlin	FALSE	50.0
A5_81790643	control_Berlin	FALSE	50.0
B9_79059821	control_Berlin	FALSE	50.0
H7_129187195	control_Berlin	FALSE	50.0
A10_82876065	control_Berlin	FALSE	50.0
D10_190224386	control_Berlin	FALSE	50.0
E5_129187004_repeat	control_Berlin	FALSE	50.0
D6_129182306_repeat	control_Berlin	FALSE	50.0
B10_79069372	control_Berlin	FALSE	50.0
G6_168744541	control_Berlin	FALSE	50.0
H5_82544762_190309202008	control_Berlin	FALSE	50.1
C12_168743858_repeat	control_Berlin	FALSE	50.1
A11_129183842	control_Berlin	FALSE	50.1
F8_190224717_repeat	control_Berlin	FALSE	50.1
G11_79112692_repeat	control_Berlin	FALSE	50.1
G12_190225664	control_Berlin	FALSE	50.1
D9_82885545_repeat	control_Berlin	FALSE	50.1
E6_CSF_960	control_Magdeburg	FALSE	40.1
B4_CSF_582-14	control_Magdeburg	FALSE	40.1
E1_CSF_59	control_Magdeburg	FALSE	40.2
F7_CSF_142	control_Magdeburg	TRUE	40.2
E10_CSF_16	control_Magdeburg	TRUE	40.2
A3_CSF_358-15	control_Magdeburg	TRUE	40.3
A9_CSF_695-15	control_Magdeburg	FALSE	40.3
E4_CSF_761	control_Magdeburg	FALSE	40.4
C9_CSF_1224-14	control_Magdeburg	TRUE	40.4
E12_CSF_146	control_Magdeburg	FALSE	40.4
E8_CSF_693	control_Magdeburg	FALSE	40.5
E2_CSF_396	control_Magdeburg	FALSE	40.5
F6_CSF_134	control_Magdeburg	FALSE	40.5
B6_CSF_257-15	control_Magdeburg	FALSE	40.5
A8_CSF_471-12	control_Magdeburg	FALSE	40.6
E11_CSF_783	control_Magdeburg	FALSE	40.6
E5_CSF_123	control_Magdeburg	FALSE	40.6
C11_CSF_986-14	control_Magdeburg	FALSE	40.6
A1_CSF_670-12	control_Magdeburg	FALSE	40.7
C12_CSF_1299-14	control_Magdeburg	FALSE	40.7
C5_CSF_706-14	control_Magdeburg	FALSE	40.7
F9_CSF_420	control_Magdeburg	FALSE	40.7

shortname	group	exclude	median CoV
F4_CSF_51	control_Magdeburg	FALSE	40.8
F2_CSF_755	control_Magdeburg	FALSE	40.8
A10_CSF_201-15	control_Magdeburg	FALSE	40.8
B1_CSF_326-15	control_Magdeburg	FALSE	40.8
F3_CSF_865	control_Magdeburg	FALSE	40.9
B9_CSF_851-15	control_Magdeburg	FALSE	40.9
F8_CSF_143	control_Magdeburg	FALSE	40.9
sampleC09	control_Sweden	TRUE	41.9
sampleC06	control_Sweden	FALSE	42.4
sampleD07	control_Sweden	FALSE	42.6
sampleA01	control_Sweden	FALSE	42.7
sampleE03	control_Sweden	FALSE	42.7
sampleB02	control_Sweden	FALSE	42.8
sampleD02	control_Sweden	FALSE	42.8
sampleE02	control_Sweden	TRUE	42.9
sampleC01	control_Sweden	TRUE	42.9
sampleC07	control_Sweden	FALSE	42.9
sampleE01	control_Sweden	FALSE	42.9
sampleC11	control_Sweden	FALSE	42.9
sampleD06	control_Sweden	FALSE	42.9
sampleC02	control_Sweden	FALSE	42.9
sampleD05	control_Sweden	FALSE	42.9
sampleD11	control_Sweden	TRUE	42.9
sampleE06	control_Sweden	FALSE	43.0
sampleD01	control_Sweden	TRUE	43.0
sampleC10	control_Sweden	TRUE	43.0
sampleD04	control_Sweden	FALSE	43.0
sampleB06	control_Sweden	FALSE	43.0
sampleA08	control_Sweden	TRUE	43.0
sampleA07	control_Sweden	FALSE	43.0
sampleE04	control_Sweden	FALSE	43.1
sampleC03	control_Sweden	FALSE	43.1
sampleE05	control_Sweden	FALSE	43.1
sampleC12	control_Sweden	FALSE	43.1
sampleD03	control_Sweden	FALSE	43.1
sampleD10	control_Sweden	FALSE	43.2
sampleD12	control_Sweden	FALSE	43.2
sampleC08	control_Sweden	FALSE	43.2
F2_190226445	unknown_Berlin	TRUE	50.8
C4_168751222	unknown_Berlin	TRUE	51.0
B7_81787765	unknown_Berlin	TRUE	51.8
C8_82882588_repeat	unknown_Berlin	FALSE	52.0
H1_82881356	unknown_Berlin	TRUE	52.3
B5_81783170	unknown_Berlin	TRUE	52.4
A1_81776353_repeat2	unknown_Berlin	FALSE	52.5
A6_82545313	unknown_Berlin	TRUE	52.6
G5_82544653	unknown_Berlin	TRUE	52.7
F9_82880110_190304154132	unknown_Berlin	TRUE	53.3
D11_129188366_repeat	unknown_Berlin	FALSE	53.4
B4_190223925	unknown_Berlin	TRUE	53.5

shortname	group	exclude	median CoV
F11__82542649	unknown__Berlin	FALSE	53.5
B11__129182819	unknown__Berlin	FALSE	53.7
D5__129186580__repeat	unknown__Berlin	FALSE	53.7
C2__79070601	unknown__Berlin	TRUE	53.8
B10__CSF__585-14	unknown__Magdeburg	TRUE	26.3
A7__CSF__531-12__column__clog__repeat	unknown__Magdeburg	FALSE	29.5
A5__CSF__660-12	unknown__Magdeburg	TRUE	29.9
A6__CSF__1081-12	unknown__Magdeburg	TRUE	30.6

Leave-one-out impact on within-group CoV (%)

1.6.3 Coefficient of Variation

The Coefficient of Variation (CoV) is a quality metric for the reproducibility of replicate measurements, here visualized using box- and violin-plots.

Only samples that are NOT marked ‘exclude’ in the provided sample metadata and are in a sample group among at least 3 replicates are used for these figures. The user-specified filtering rules (eg; filter_min_detect, filter_min_peptide_per_prot, etc.) were applied within each sample group independently and remaining peptides were subsequently normalized using the ” vsn&modebetween_protein ” algorithm (an important parameter to keep in mind when comparing across datasets/analyses). Only peptides with at least 3 data points across replicate samples are used for each CoV computation.

Peptide-level CoV:

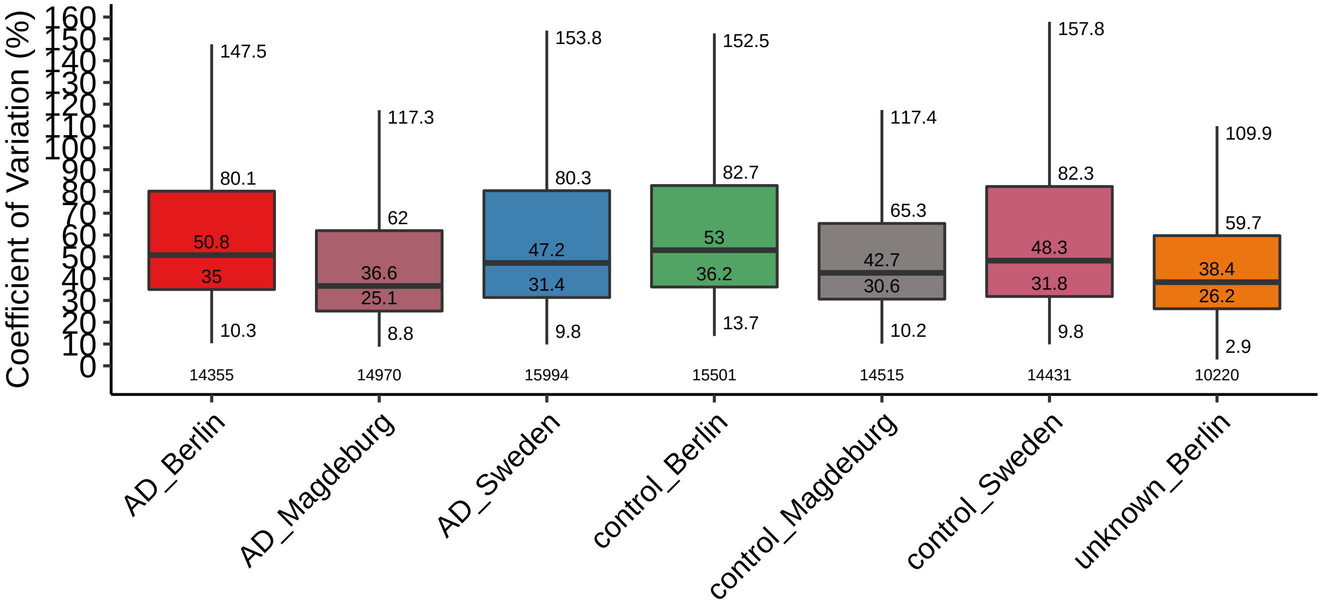

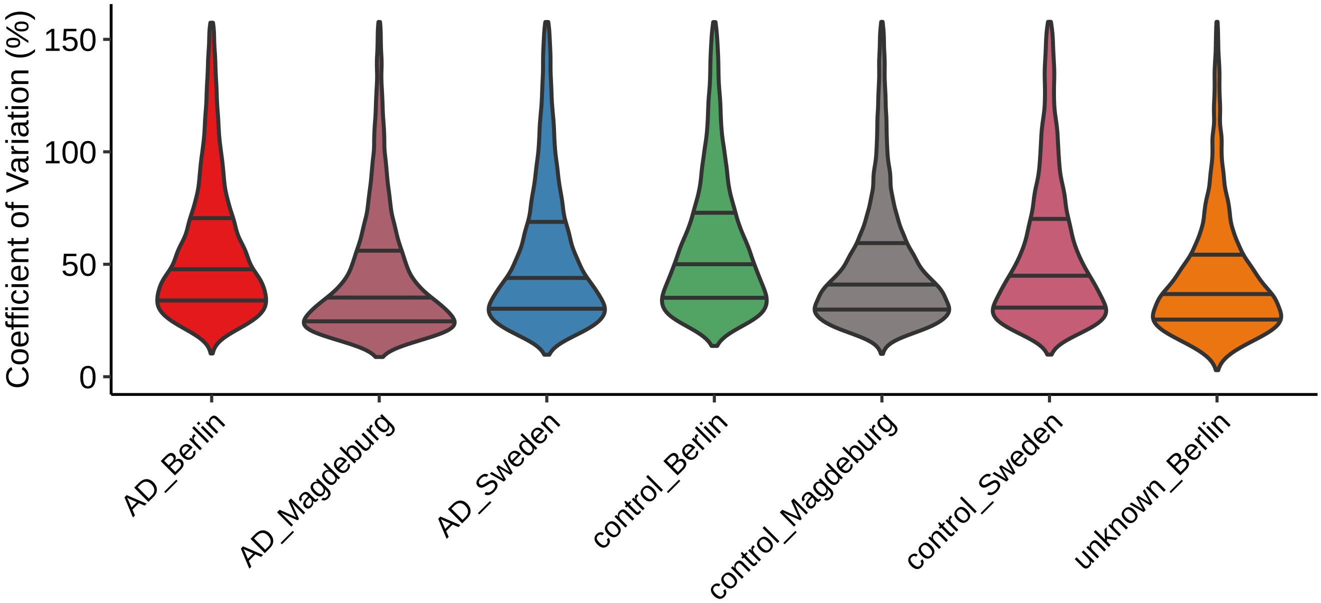

Protein-level CoV:

(analogous to peptide CoV's, but with additional rollup to protein abundances using 'maxlfq' algorithm)

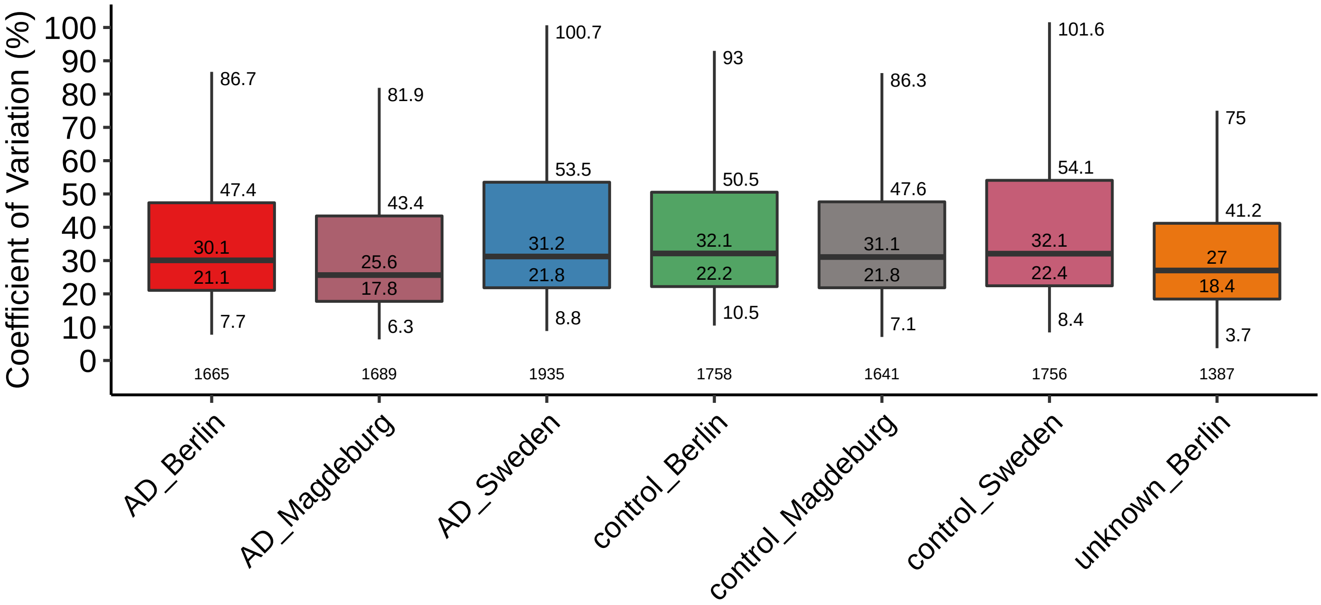

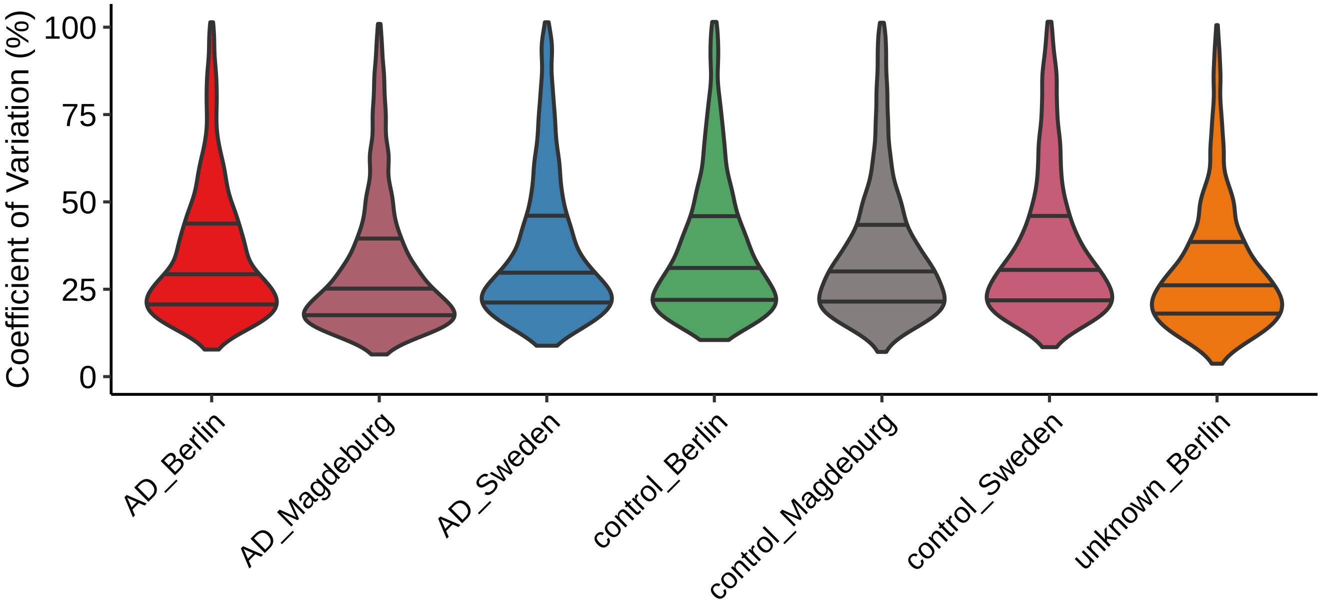

1.7 PCA

A visualization of the first three PCA dimensions illustrates sample clustering. The goal of these figures is to detect global effects from a quality control perspective, such as samples from the same experiment batch clustering together, not to be sensitive to a minor subset of differentially abundant proteins (for which specialized statistical models can be applied downstream).

If additional sample metadata was provided, such as experiment batch, sample-prep dates, gel, etc., multiple PCA figures will be generated with respective color-codings. Users are encouraged to provide relevant experiment information as sample metadata and use these figures to search for unexpected batch effects.

The pcaMethods R package is used here to perform the Probabilistic PCA (PPCA). The set of peptides used for this analysis consists of those peptides that pass your filter criteria in every sample group. If any samples are marked as 'exclude' in the provided sample metadata, an additional PCA plot is generated with these samples included (depicting the 'exclude' samples as square symbols).

Rationale behind data filter
As mentioned above, the aim of the PCA figures is to identify global effects. To achieve this, we compute sample distances on the subset of peptides identified in each group which prevents rarely detected peptides/proteins from having a disproportionate effect on sample clustering. This pertains not only to 'randomly detected contaminant proteins' but also to proteins with abundance levels near the detection limit, which may be detected in only a subset of samples (eg; some measurements will be more successful/sensitive than others).

Figure legends
The first 3 principle components compared visually (1 *vs* 2, 1 *vs* 3, 2 *vs* 3) on the rows. Left- and right-side panels on each row represent the same figure without and with sample labels. The principle components are shown on the axis labels together with their respective percentage of variance explained. Samples marked as 'exclude' in the provided sample metadata, if any, are visualized as square shapes.

PCA of all samples, including those flagged as 'exclude', using 8892 peptides

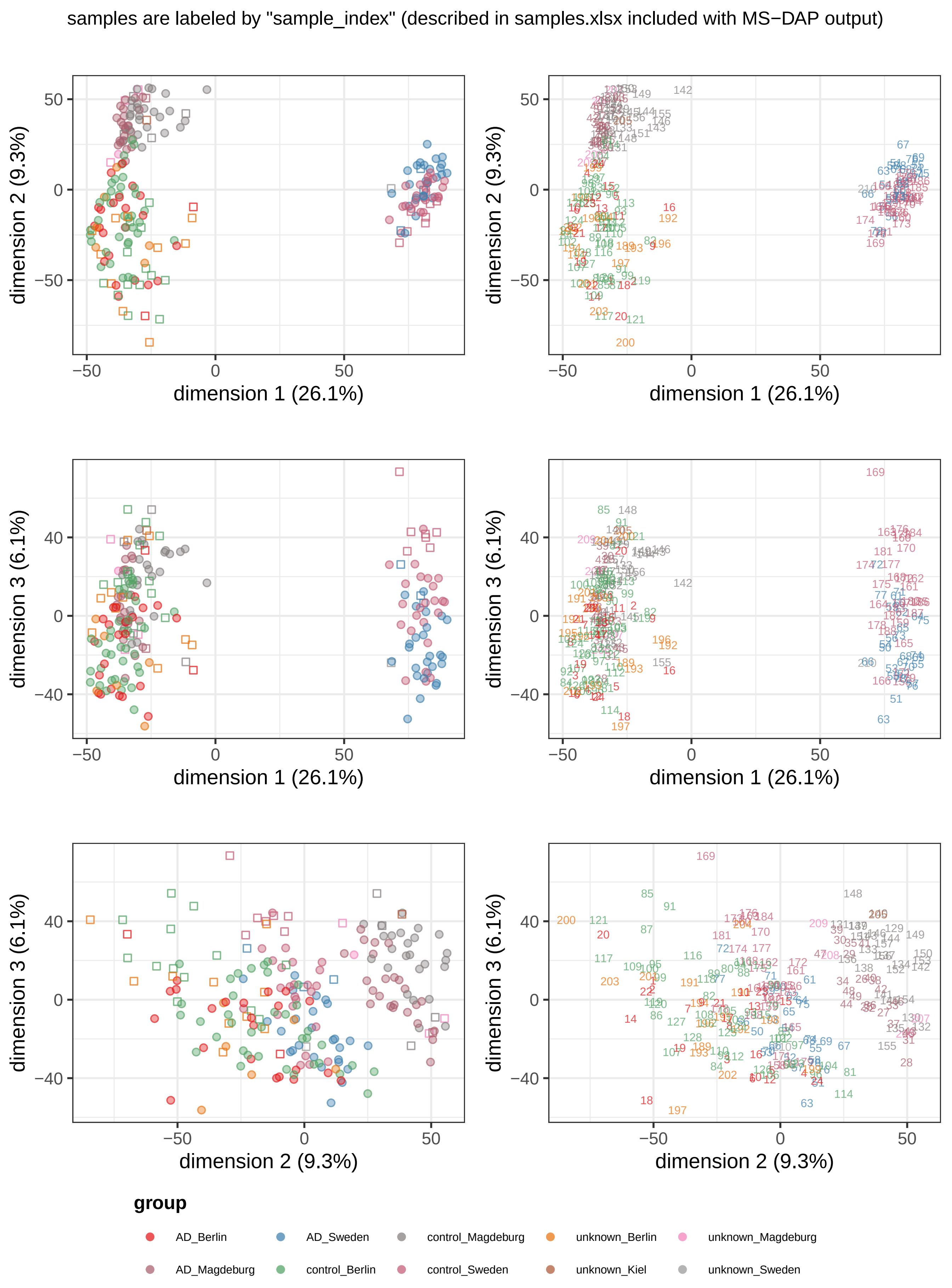

PCA only on samples not flagged as 'exclude', using 9164 peptides

samples are labeled by "sample_index" (described in samples.xlsx included with MS−DAP output)

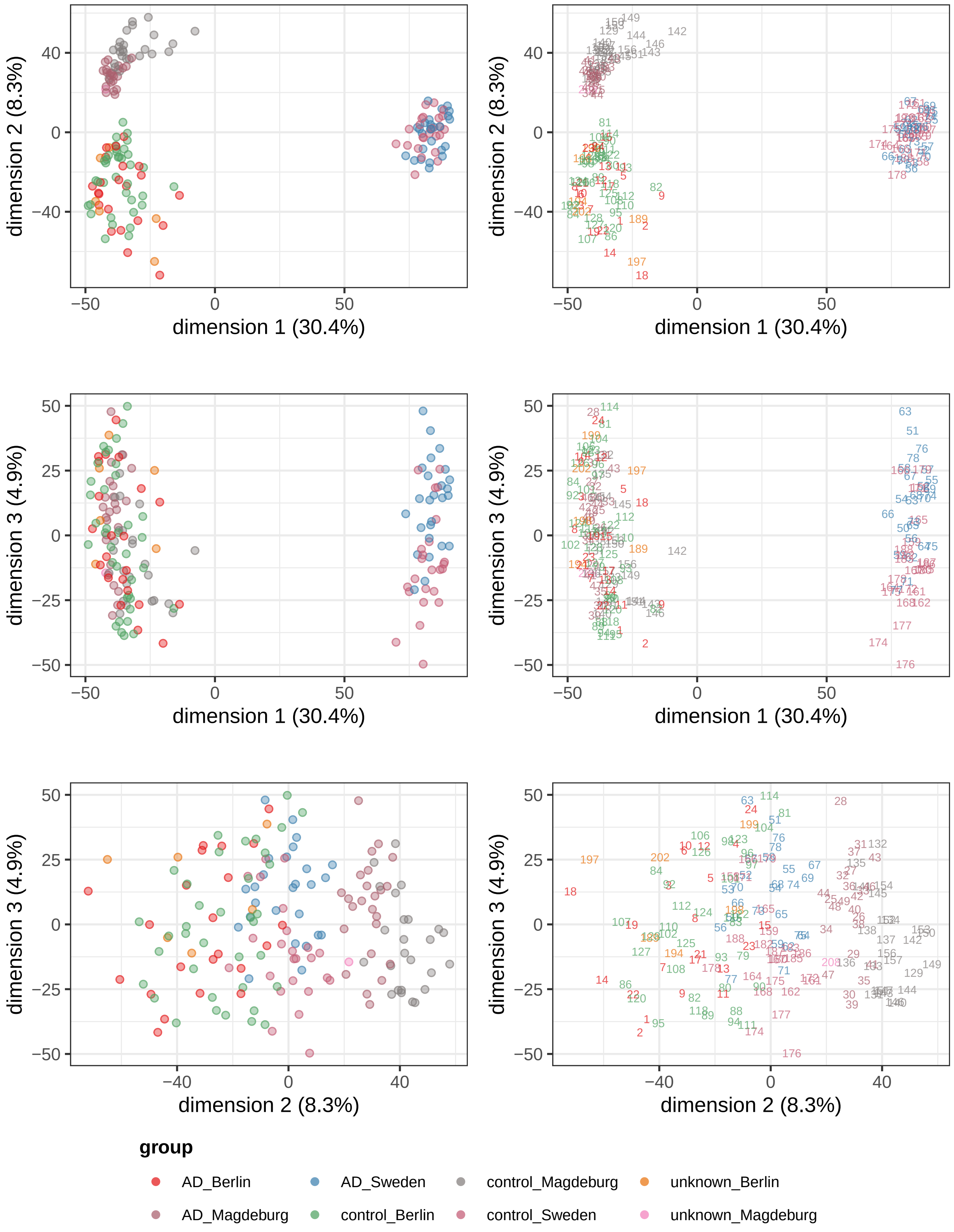

samples are labeled by "sample_index" (described in samples.xlsx included with MS−DAP output)

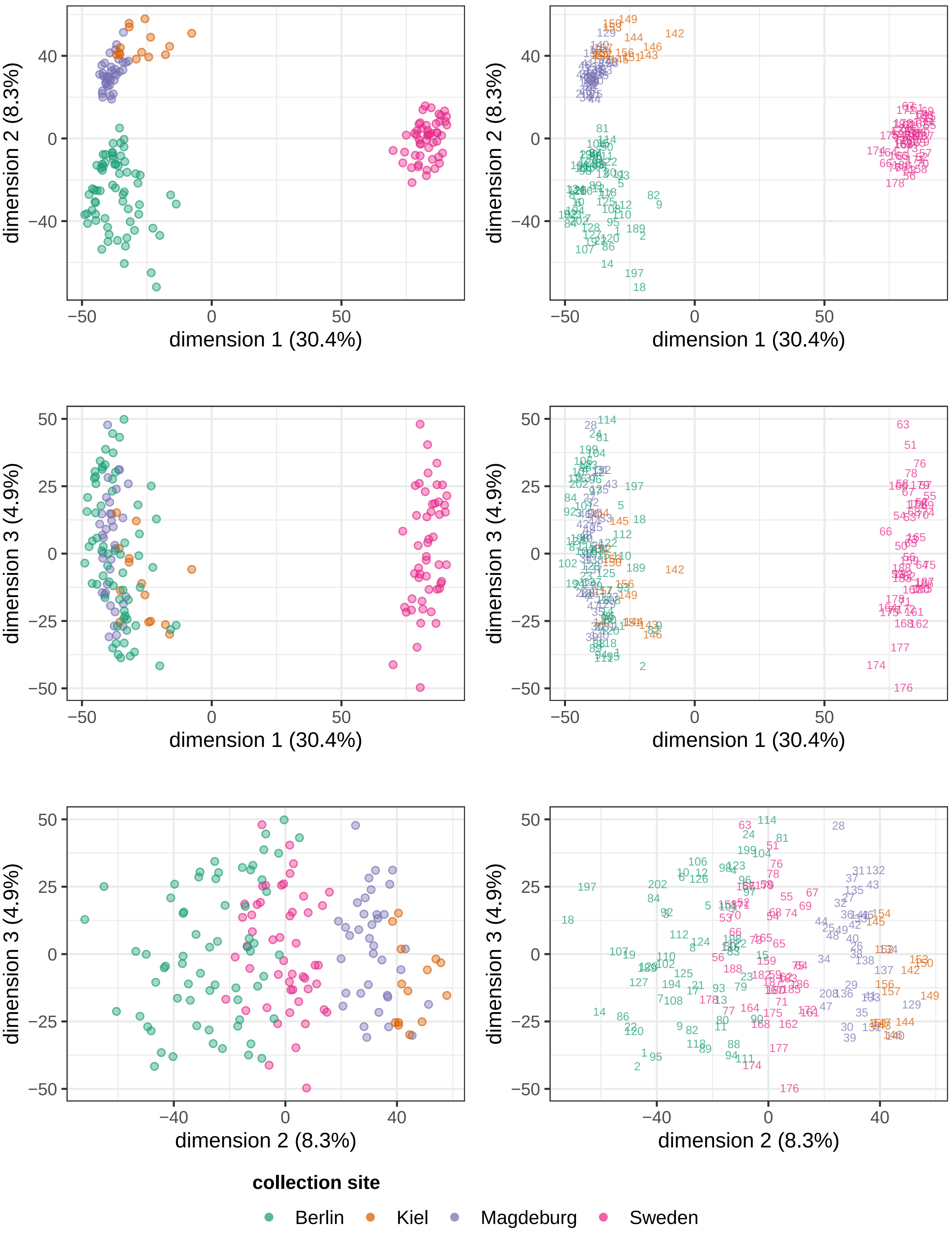

samples are labeled by "sample_index" (described in samples.xlsx included with MS−DAP output)

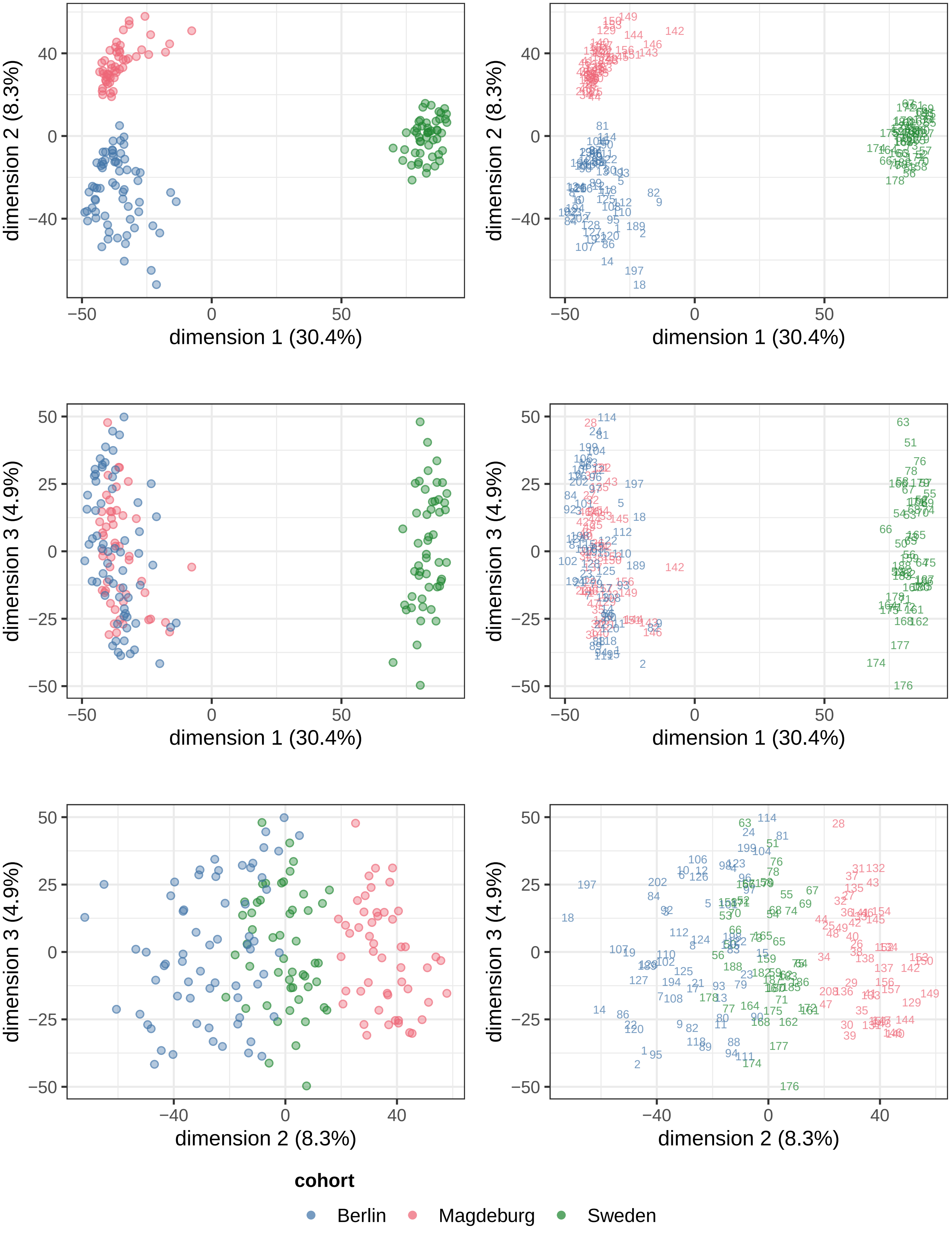

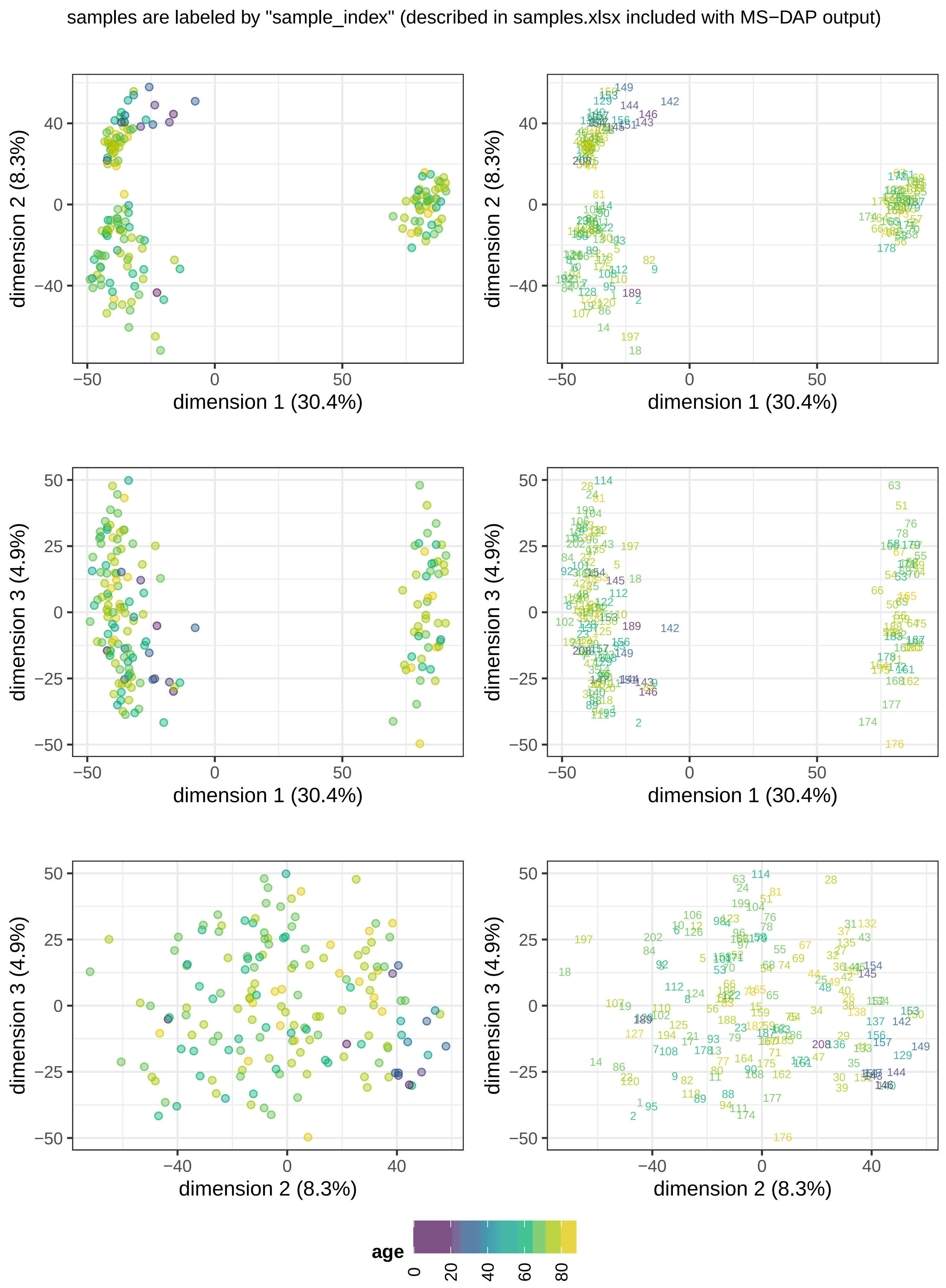
samples are labeled by "sample_index" (described in samples.xlsx included with MS−DAP output)
dimension 1 (30.4%)
dimension 2 (8.3%)
dimension 3 (4.9%)
age
0
20
40
60
80

samples are labeled by "sample_index" (described in samples.xlsx included with MS−DAP output)

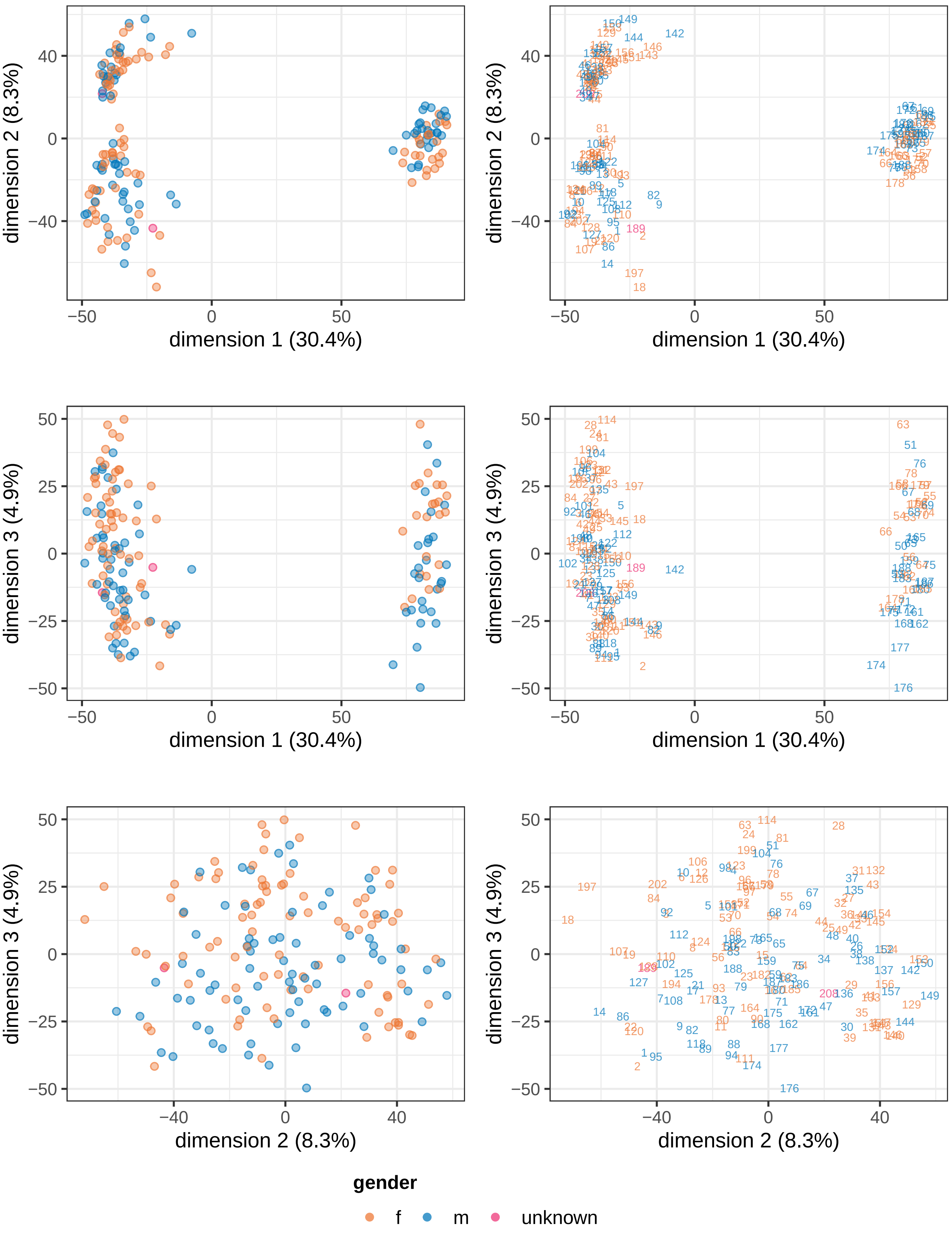

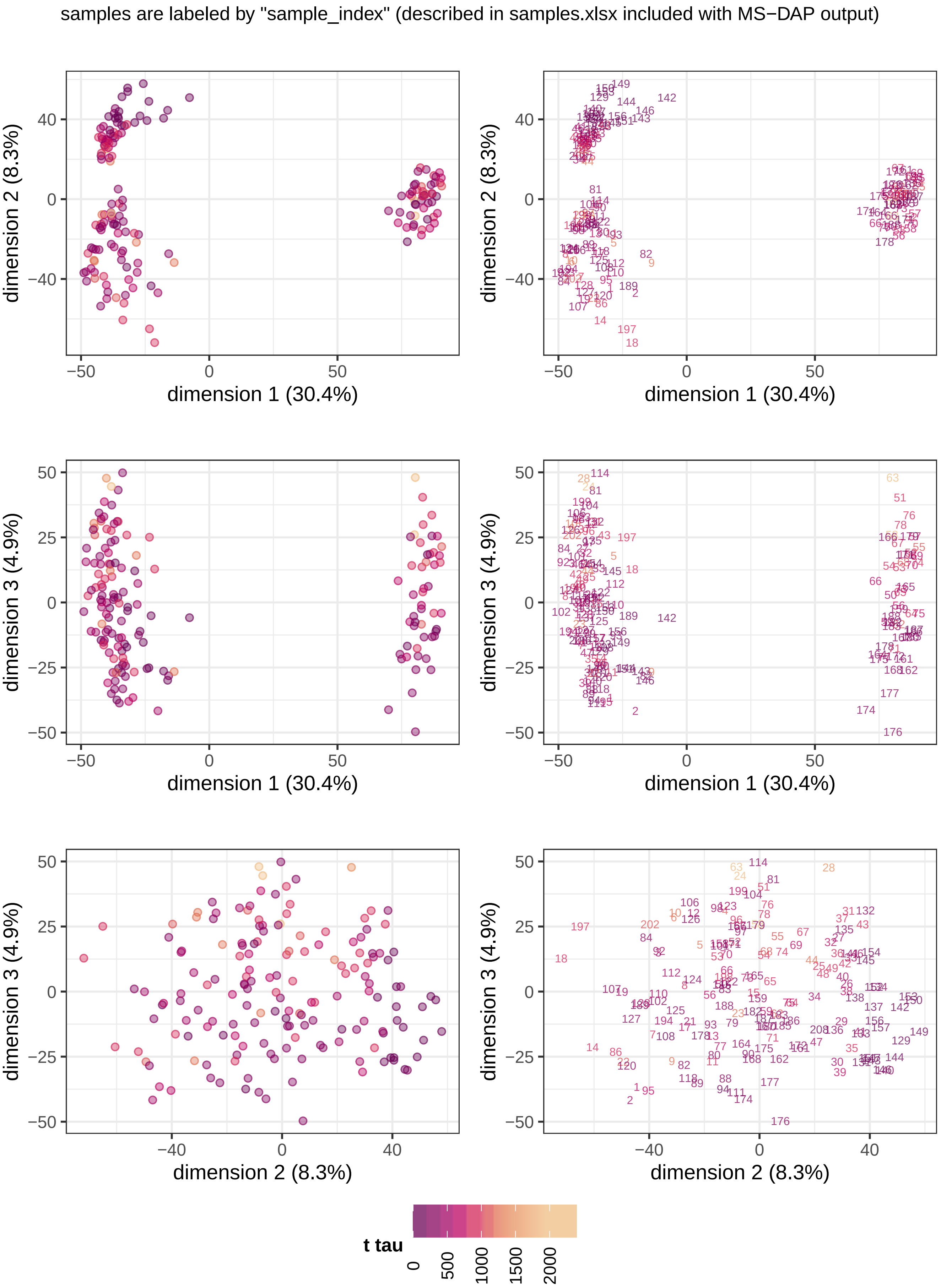
samples are labeled by "sample_index" (described in samples.xlsx included with MS−DAP output)
dimension 1 (30.4%)
dimension 2 (8.3%)
dimension 3 (4.9%)
t tau
0
500
1000
1500
2000

samples are labeled by "sample_index" (described in samples.xlsx included with MS−DAP output)

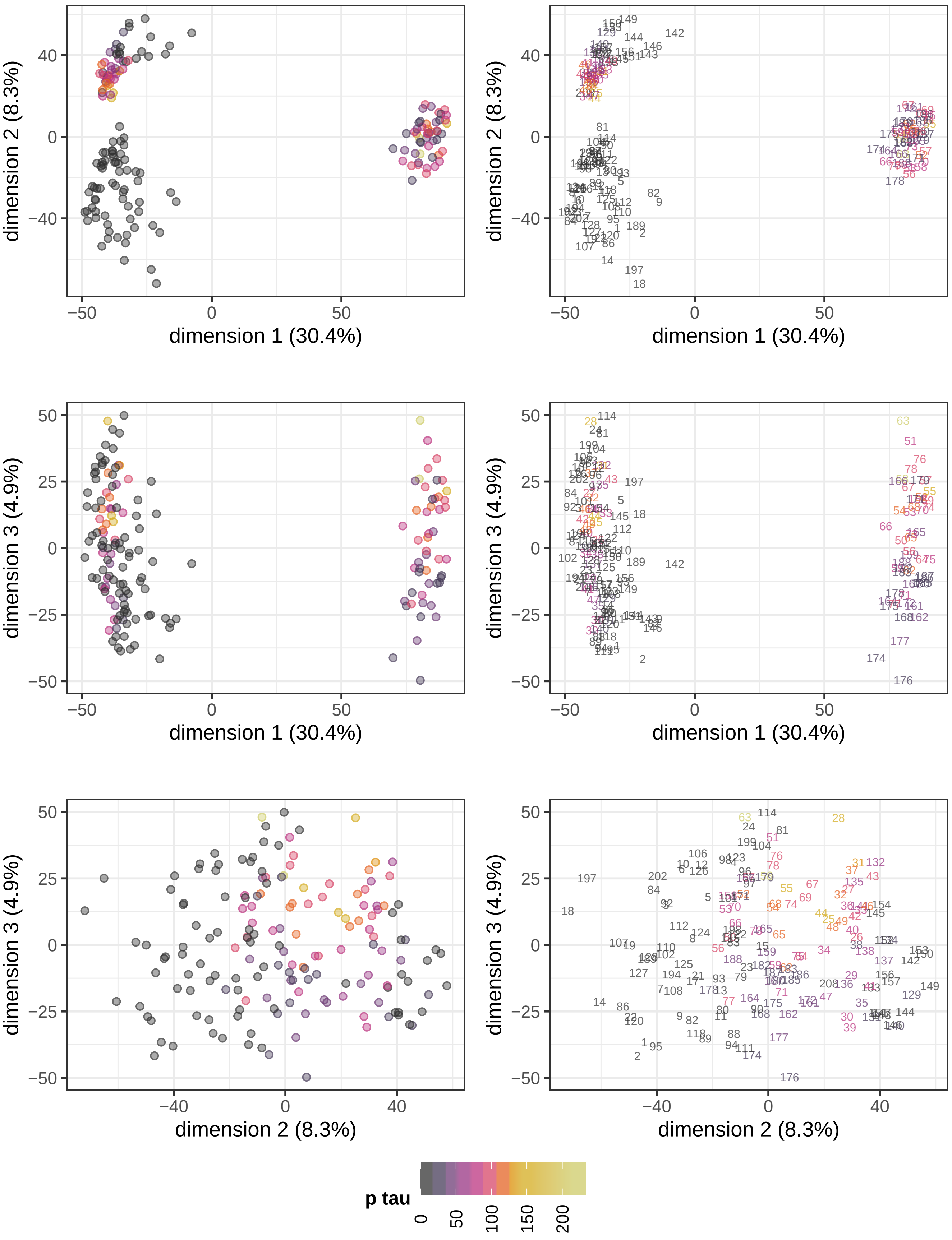

samples are labeled by "sample_index" (described in samples.xlsx included with MS−DAP output)

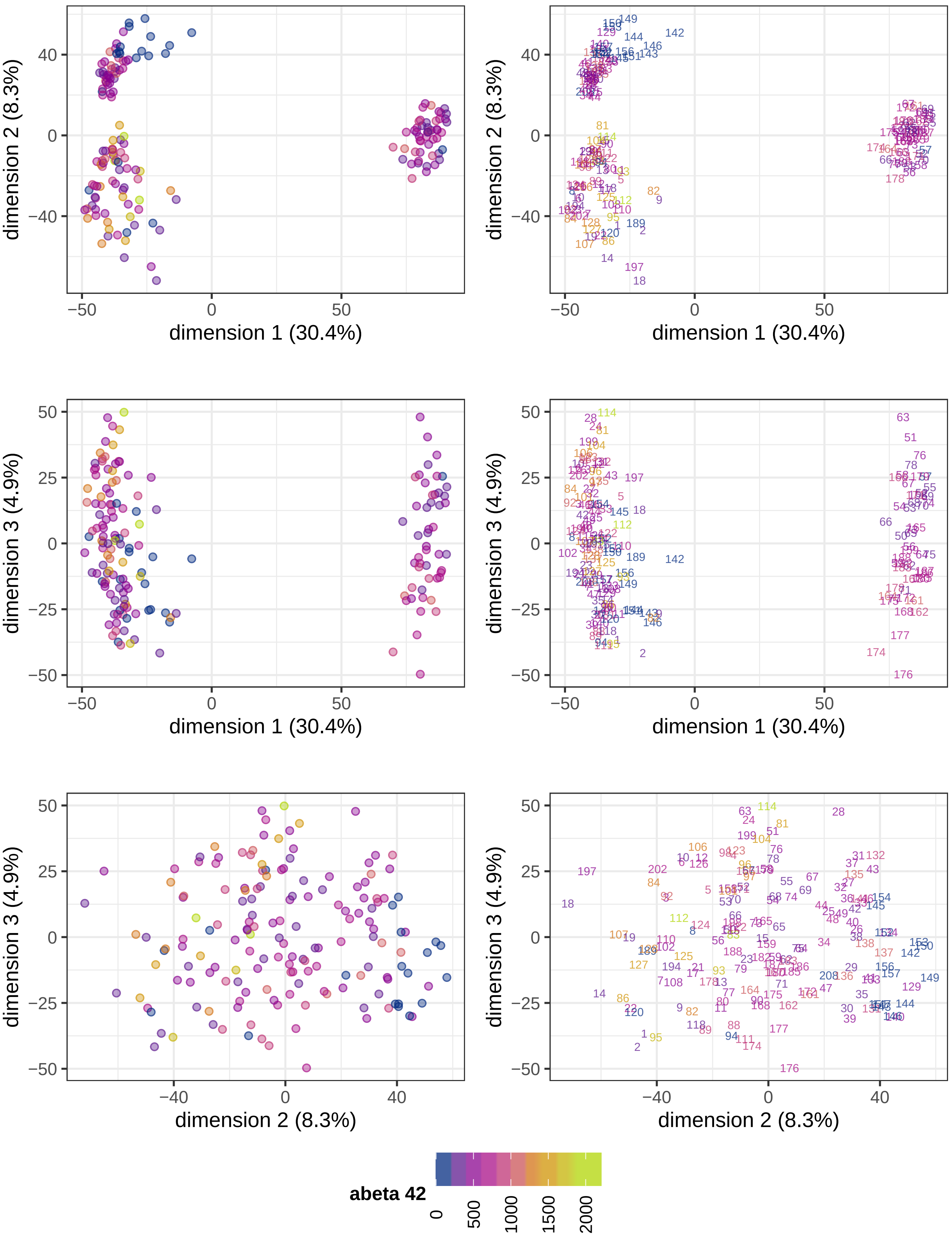

samples are labeled by "sample_index" (described in samples.xlsx included with MS−DAP output)

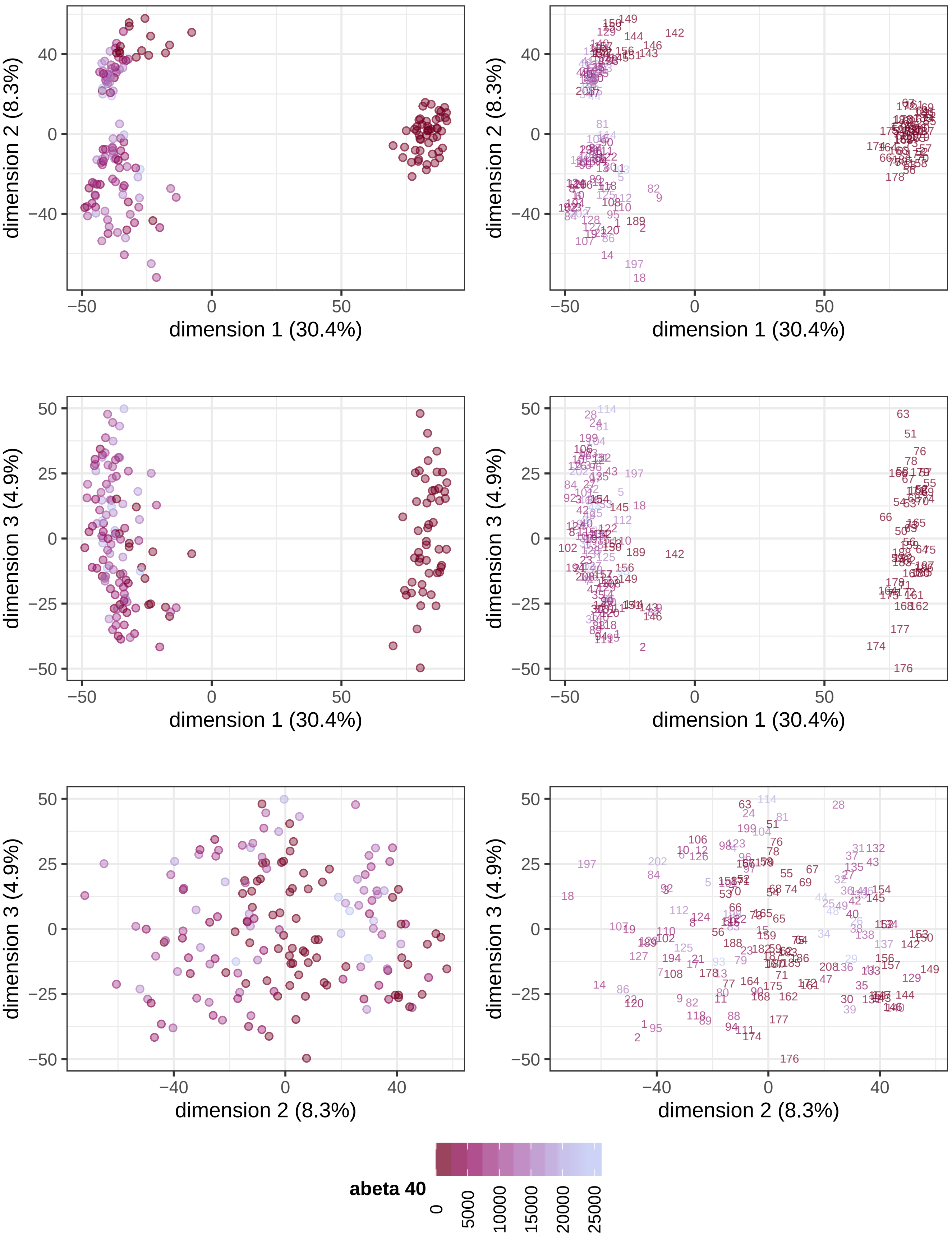

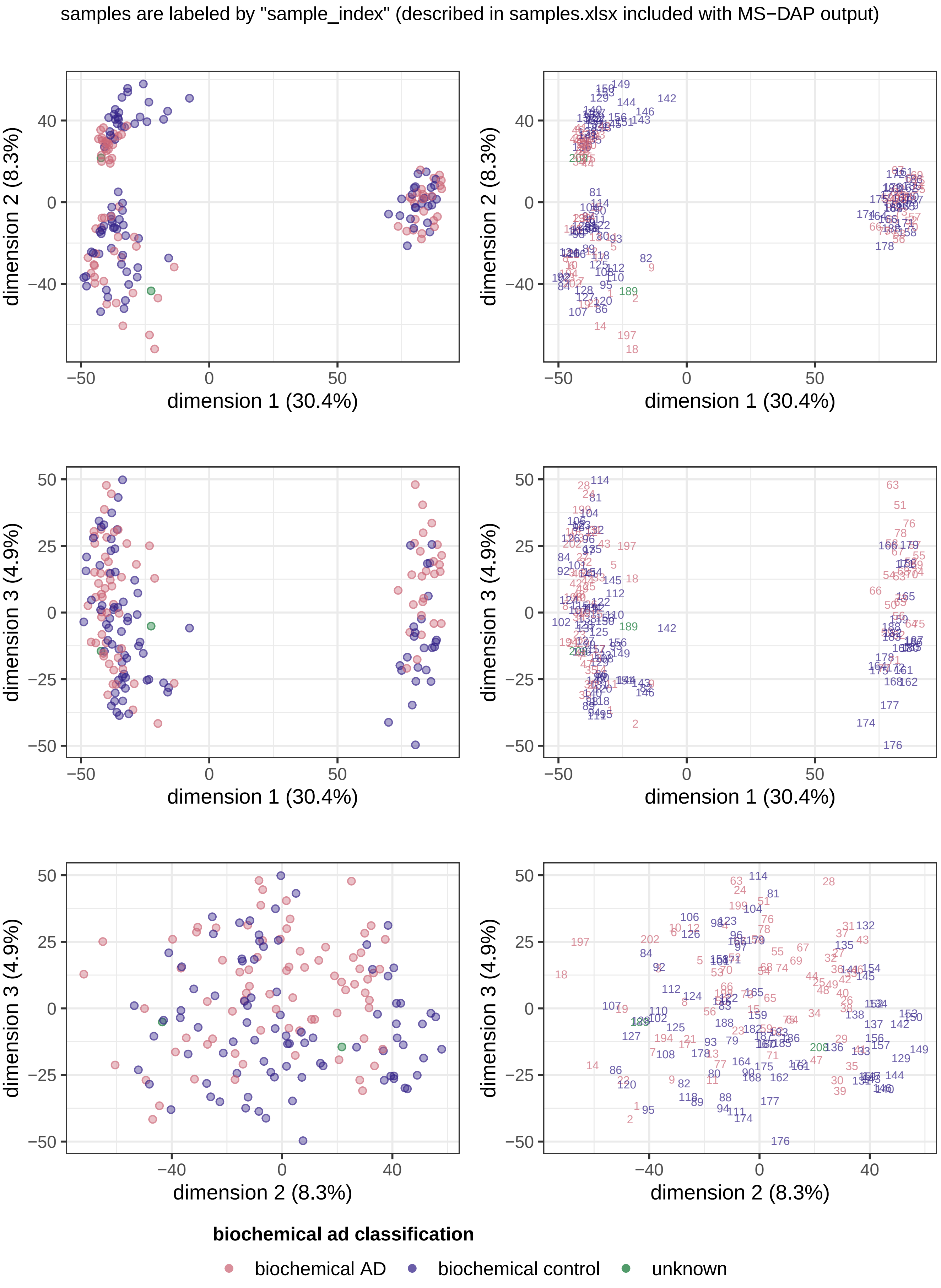

samples are labeled by "sample_index" (described in samples.xlsx included with MS−DAP output)
dimension 1 (30.4%)
dimension 2 (8.3%)
dimension 3 (4.9%)
biochemical ad classification
biochemical AD
biochemical control
unknown

samples are labeled by "sample_index" (described in samples.xlsx included with MS−DAP output)

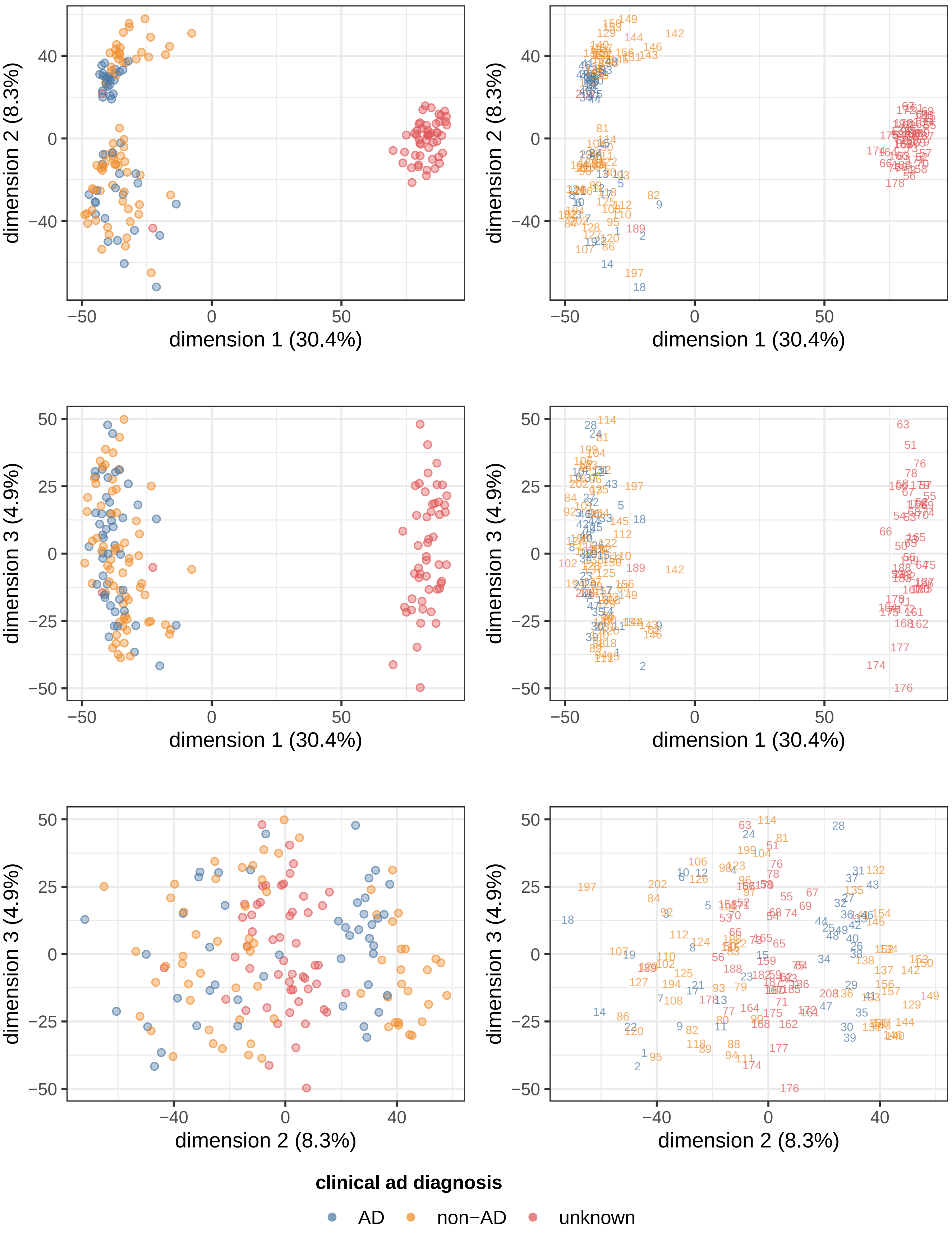

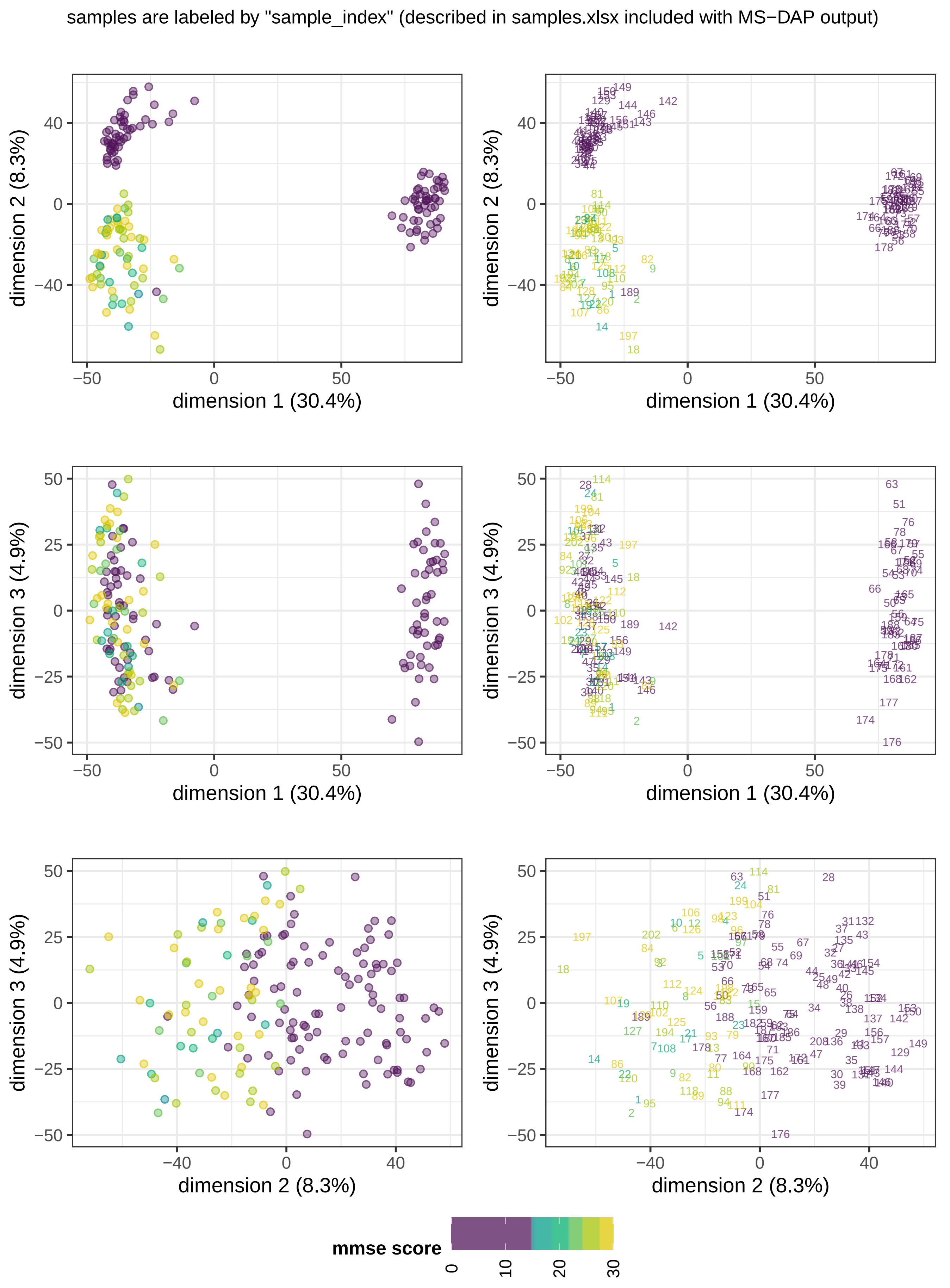
samples are labeled by "sample_index" (described in samples.xlsx included with MS−DAP output)
dimension 1 (30.4%)
dimension 2 (8.3%)
dimension 3 (4.9%)
mmse score
0
10
20
30

samples are labeled by "sample_index" (described in samples.xlsx included with MS−DAP output)

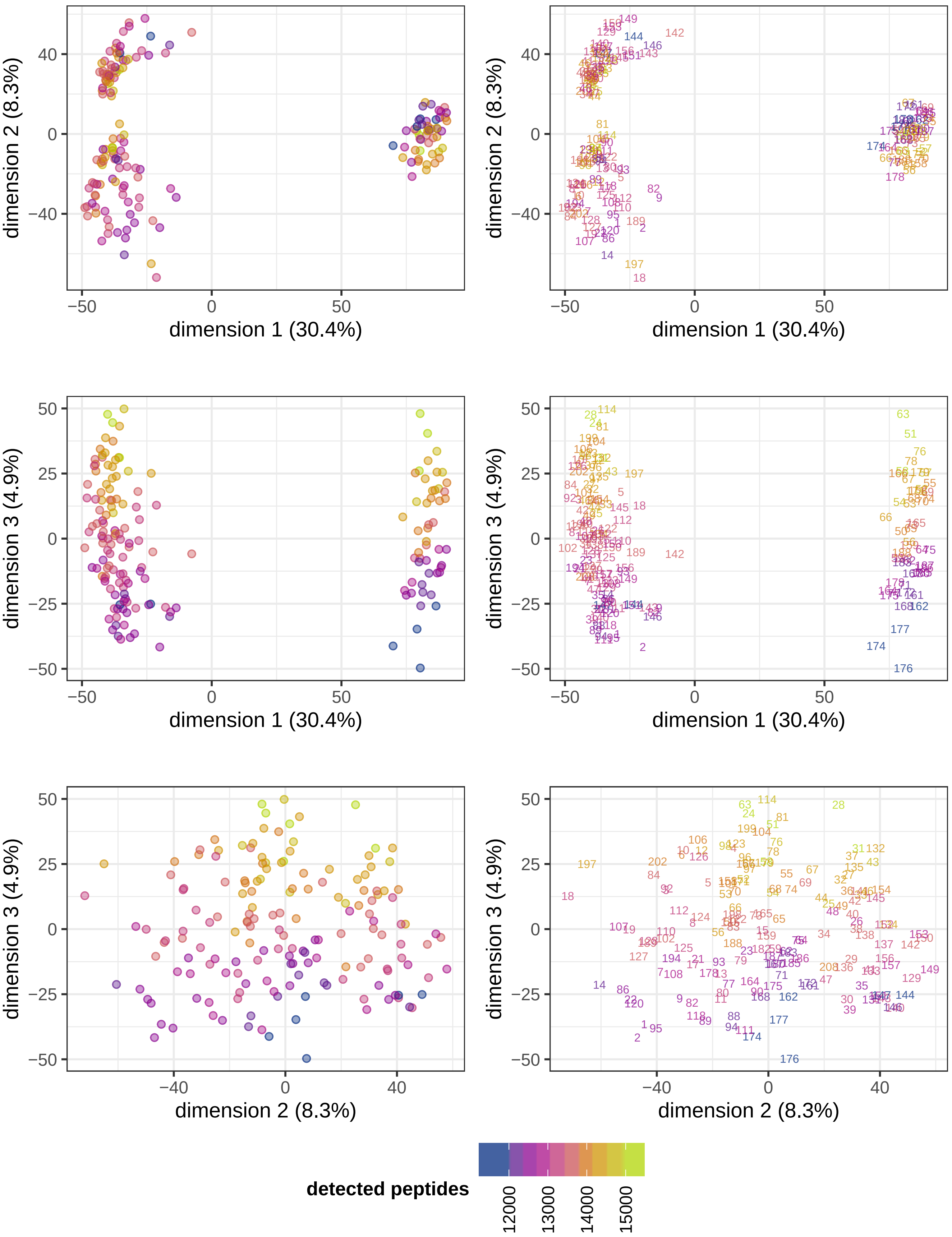

samples are labeled by "sample_index" (described in samples.xlsx included with MS−DAP output)

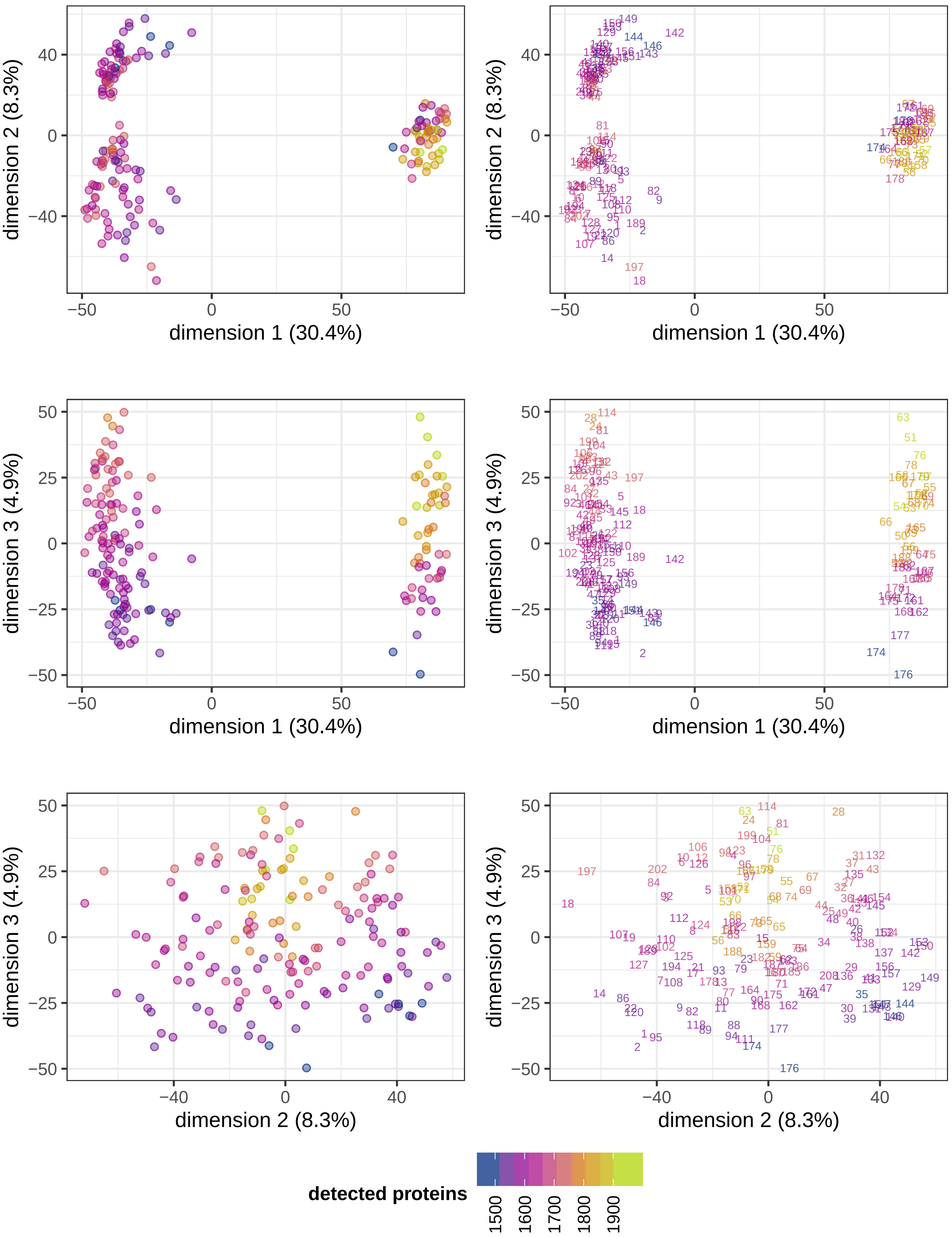

2 Differential abundance analysis

goal: maximize reliable features for quantification

In a pairwise analysis of two groups of samples, only peptides with N data-points in both groups are used for quantitative analysis (where N = defined by user settings). For example; if peptide *p* is consistently quantified in sample groups A and B but not in C/D/E, it can be used when comparing group A *versus* group B but should not be used in any other group comparisons. This approach is particularly suited to maximize the number of peptides used for statistical analysis in experimental designs with many sample groups. In MS-DAP this is referred to as "by contrast" filtering.

A common alternative strategy is a global filtering approach where peptides are selected based on their properties in the overall dataset (eg; present in x% of samples or x% of replicates in all groups) and subsequentially the resulting data matrix is used for all downstream statistical analyses. In the example above where peptide *p* is present in a subset of sample groups, *p* would either be left out (not present in majority of samples in entire dataset) or erronously used when applying t-statistics to groups B and C (since *p* is not present in group C, it may differentially detected but there are no features available for quantitative analysis)

contrasts and foldchanges

Note that a MS-DAP contrast for "A vs B" returns foldchanges for B/A. For example, for the contrast "control vs disease" a positive log2 foldchange implies protein abundances are higher in the "disease" sample group.

2.1 control_Berlin,control_Magdeburg... vs AD_Berlin,AD_Magdeburg,AD_Sweden

- **user setting:** using 'filter by contrast' peptide filtering approach
- 11824 peptides in 1445 proteins remain in the current contrast after peptide filters and are used for the statistical analysis in this section
- qvalue threshold: 0.01
- log2 foldchange threshold: 0.176

2.1.1 volcano

The plot title shows the statistical model and contrast (sample groups in the comparison). Left- and right-side figure panels on each row represent the same figure without and with labels for the 25 proteins with lowest p-value.

Bottom figure panels have limited x- and y-axis. For datasets with a small number of strong outliers in p-value or fold-change, which may have a profound effect on the plot scales, this allows inspection of the remainder of the volcano plot without disproportionate influence by 'extreme' values.

Labels for proteins that are more than 12 characters long are truncated for visual clarity (indicated by trailing ...). For protein identifiers that are ambiguous, e.g. a protein-group with assigned genes "gene1a;gene1b", only the first label/ID is shown for visual clarity (indicated by trailing *).

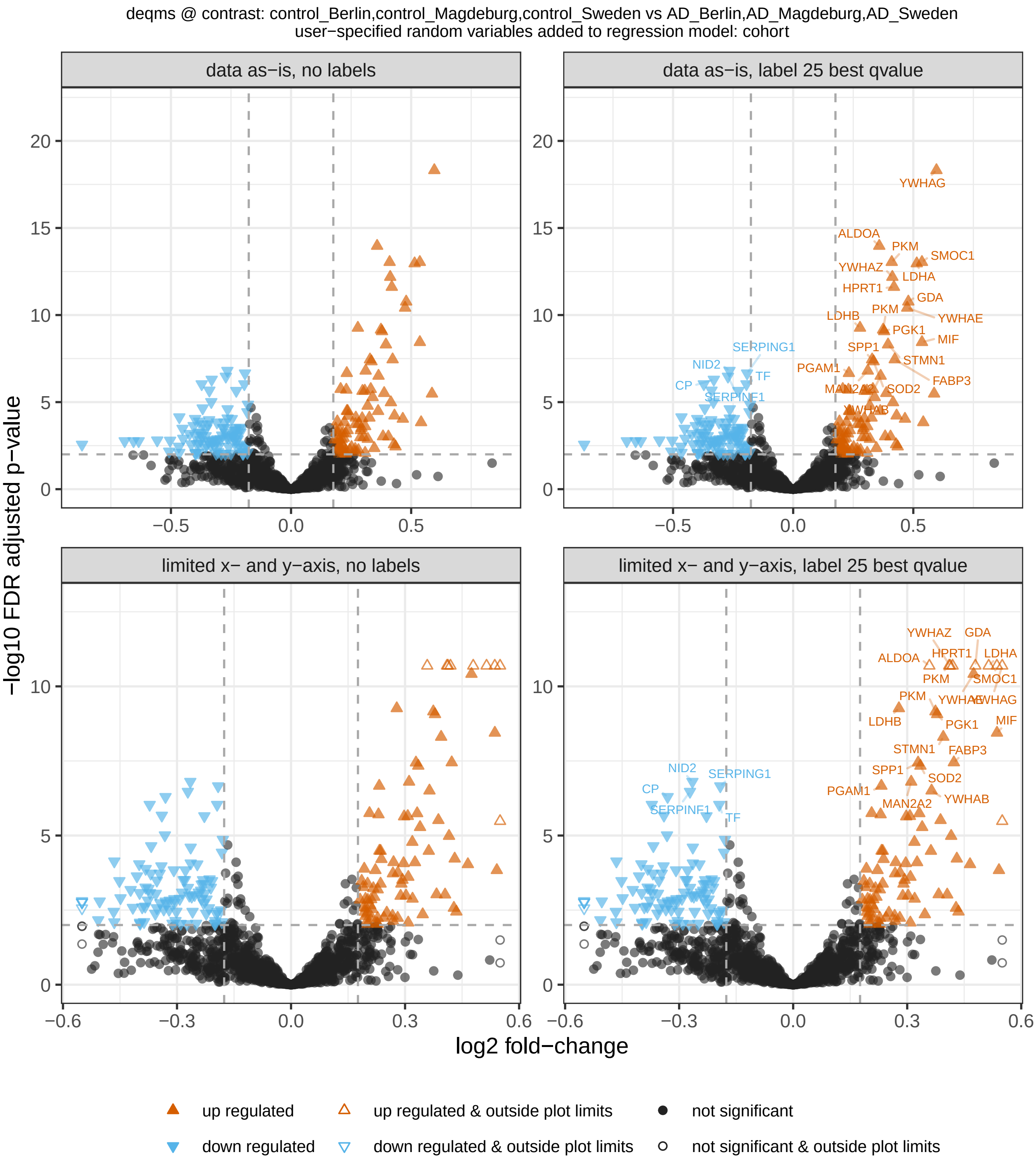
deqms @ contrast: control_Berlin,control_Magdeburg,control_Sweden vs AD_Berlin,AD_Magdeburg,AD_Sweden
user−specified random variables added to regression model: cohort
data as−is, no labels
data as−is, label 25 best qvalue
limited x− and y−axis, no labels
limited x− and y−axis, label 25 best qvalue
−log10 FDR adjusted p−value
log2 fold−change
YWHAG
ALDOA
PKM
SMOC1
YWHAZ
LDHA
HPRT1
GDA
LDHB
YWHAE
PGK1
MIF
SERPING1
SPP1
STMN1
NID2
PGAM1
FABP3
TF
CP
MAN2A2
SOD2
SERPINF1
YWHAB
up regulated
down regulated
up regulated & outside plot limits
down regulated & outside plot limits
not significant
not significant & outside plot limits

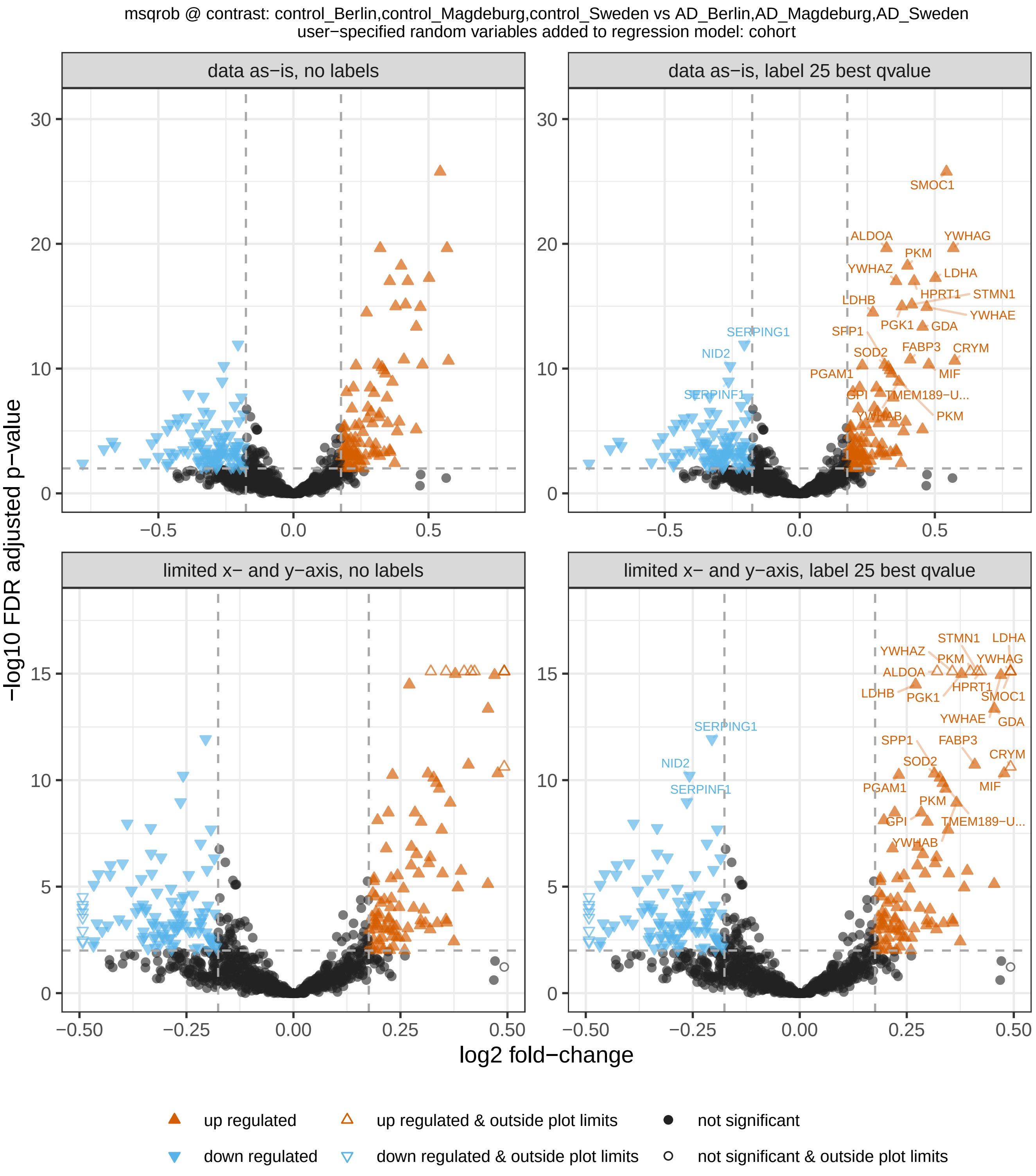

msqrob @ contrast: control_Berlin,control_Magdeburg,control_Sweden vs AD_Berlin,AD_Magdeburg,AD_Sweden
user−specified random variables added to regression model: cohort
data as−is, no labels
data as−is, label 25 best qvalue
limited x− and y−axis, no labels
limited x− and y−axis, label 25 best qvalue
−log10 FDR adjusted p−value
log2 fold−change
SMOC1
ALDOA
YWHAG
PKM
YWHAZ
LDHA
HPRT1
STMN1
LDHB
YWHAE
PGK1
GDA
SERPING1
SPP1
NID2
SOD2
FABP3
CRYM
PGAM1
MIF
SERPINF1
GPI
TMEM189−U...
YWHAB
up regulated
up regulated & outside plot limits
not significant
down regulated
down regulated & outside plot limits
not significant & outside plot limits

2.1.2 foldchange distribution

Distributions of estimated foldchanges produced by the statistical models. If the mode is far from 0, consider alternative normalization strategies. Do note the scale on the x-axis, for some experiments the foldchanges are very low which in turn may exaggerate this figure.

note; the MSqRob model tends to assign zero (log)foldchange for proteins with minor difference between conditions where the model is very sure the null hypothesis cannot be rejected (shrinkage by the ridge regression model). As a result, many foldchanges will be zero and the density plot for MSqRob may look like a spike instead of the expected Gaussian shape observed in other models

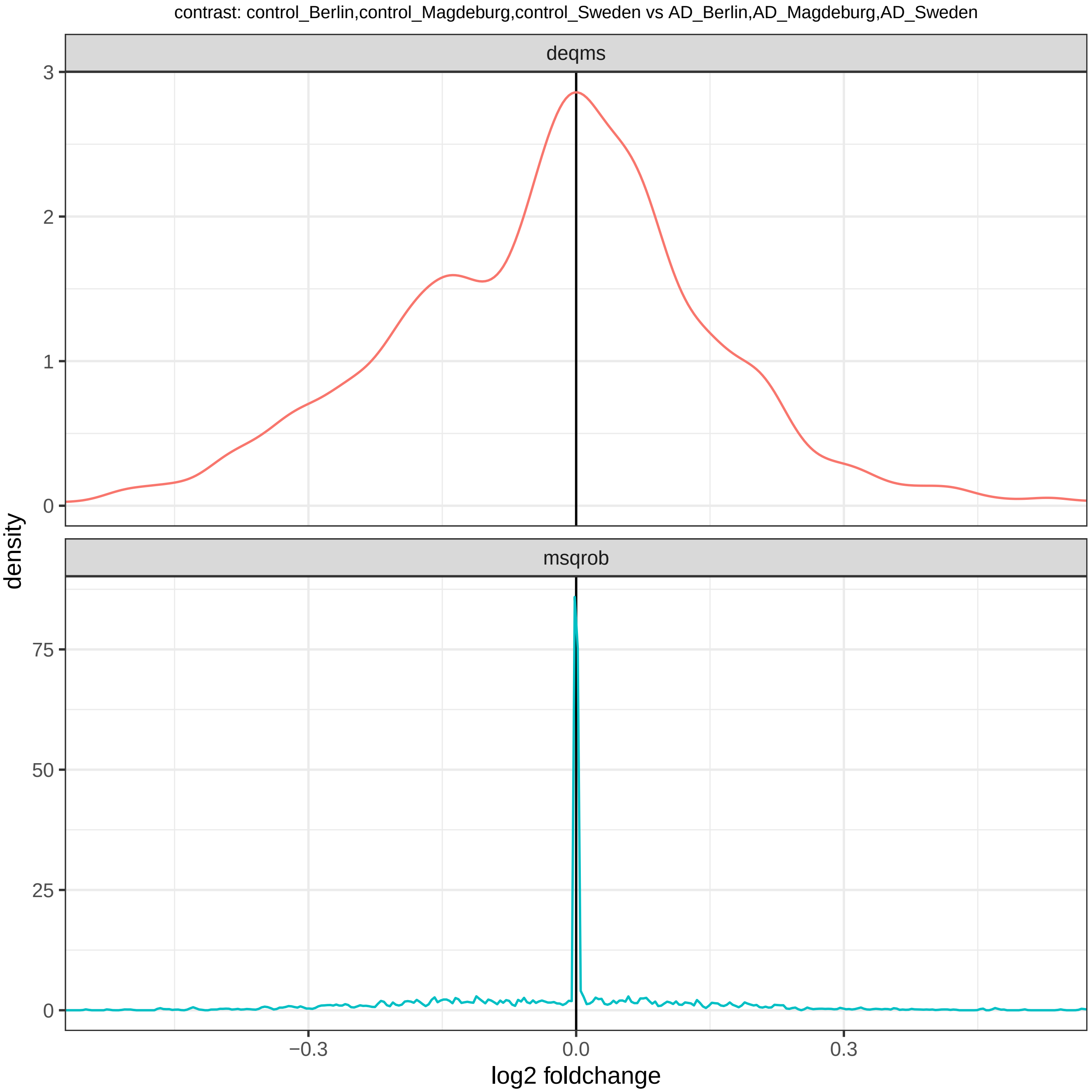

2.1.3 p-value distribution

Histogram of p-values computed by differential expression analysis algorithms, as-is, for quality-control inspection. The horizontal line indicates the expected counts assuming a uniform distribution (total number of p-values divided by number of histogram bins)

See further: https://www.ncbi.nlm.nih.gov/pmc/articles/PMC6164648/

See further: http://varianceexplained.org/statistics/interpreting-pvalue-histogram/

note; the MSqRob and MS-EmpiRe models often yield p-value distributions that show a large peak at p-value 1, these are typically proteins with estimated log foldchanges at/near zero where these models are very sure the null hypothesis cannot be rejected

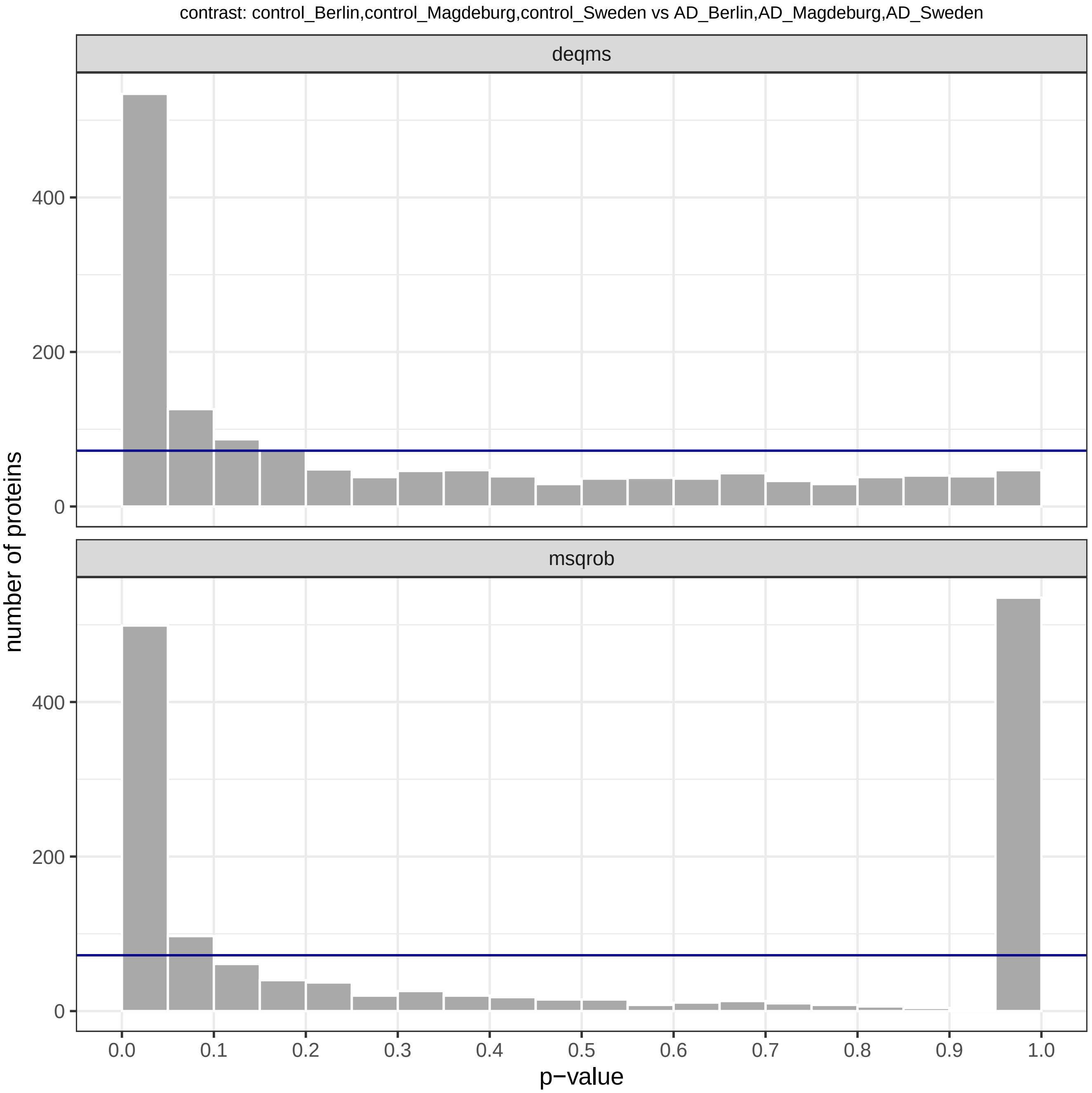

2.1.4 differential detect

Some proteins may not have peptides with sufficient data points over samples to be used for differential expression analysis (depending on the user-defined filtering criteria in how many replicates peptides should be observed), but do show a strong difference in the number of detected peptides between sample groups. In some proteomics experimental designs, for example a wildtype-knockout APMS study, those are interesting proteins. The DEA based on peptide abundance values (volcano plots above) are the main result for differential testing in MS-DAP but as a situationally useful tool MS-DAP also includes a 'protein detection' z-score, based on the number of times a peptide for each protein was detected per sample group (/experimental condition), as an alternative means of differential testing.

Below figure shows the distribution of these scores with thresholds at 3 std. Both the z-scores and the counts these are based upon are available in the statistical result Excel table.

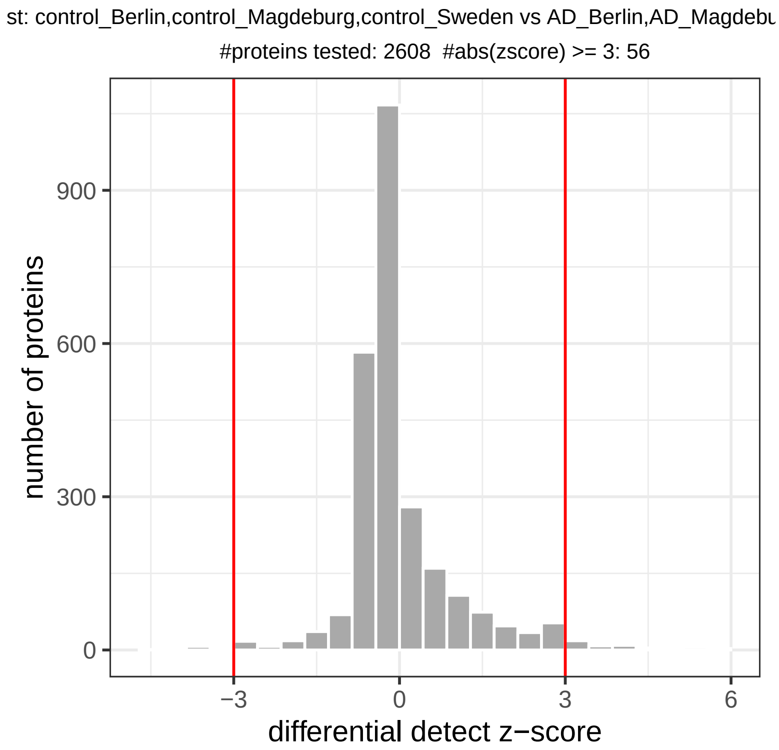

3 Summary of differential testing

Differential Expression Analysis: number of proteins found statistically significant.

contrast		algorithm	#test	#hits	top10 significant
control_Berlin,... AD_Berlin,AD_Ma...	vs	deqms	1445	180	ywhag, aldoa, smoc1, pkm, ldha, ywhaz, hprt1, gda, ywhae, ldhb
control_Berlin,... AD_Berlin,AD_Ma...	vs	msqrob	1445	197	smoc1, ywhag, aldoa, pkm, ldha, hprt1, ywhaz, stmn1, pgk1, ywhae

Differential Detection: prioritize proteins with more peptide detections in some group. A simple metric to complement results from DEA, which is the main result (eg; consider proteins with too few data points for DEA).

contrast		#proteins tested	#abs(zscore) >= 3	top10
control_Berlin,... AD_Berlin,AD_Ma...	vs	2608	56	tubb, park7, plppr4, smarce1, cdon, dlat, glce, gsta4, rbmxl1;..., sqstm1

4 log

[info] reading Spectronaut report…

[info] 22557/31469 precursors remain after selecting the 'best' precursor for each modified sequence

[info] 6882/6882 protein accessions and 2771/2771 protein groups were mapped to provided fasta file(s)

[info] contrast: control__Berlin,control__Magdeburg,control__Sweden vs AD__Berlin,AD__Magdeburg,AD__Sweden

[info] using 23 threads for multiprocessing

[progress] caching filter data took 3.2 minutes

[progress] peptide to protein rollup with MaxLFQ (implementation: iq) took 2 seconds

[progress] peptide to protein rollup with MaxLFQ (implementation: iq) took 1 seconds

[progress] peptide to protein rollup with MaxLFQ (implementation: iq) took 1 seconds

[progress] peptide to protein rollup with MaxLFQ (implementation: iq) took 2 seconds

[info] filter dataset with settings: min__detect = 8; norm__algorithm = 'vsn&modebetween__protein'; rollup__algorithm = 'maxlfq'
9164/22557 peptides were retained after filtering over all groups
11824/22557 peptides were retained after filtering within contrast: control__Berlin,control__Magdeburg,control__Sweden vs AD__Berlin,AD__Magdeburg,AD__S
18265/22557 peptides were retained after filtering within each group independently ("by group")

[progress] peptide filtering and normalization took 4.6 minutes

[info] differential expression analysis for contrast: control__Berlin,control__Magdeburg,control__Sweden vs AD__Berlin,AD__Magdeburg,AD__Sweden

[info] using data from peptide filter: filter by contrast

[info] random variables used in current contrast: cohort

[progress] peptide to protein rollup with MaxLFQ (implementation: iq) took 1 seconds

[info] log2 foldchange threshold estimated by bootstrap analysis: 0.176

[progress] DEqMS took 1 seconds

[info] msqrob linear regression formulas (these are prioritized. eg; if a model fit fails due to lack of data, the next formula is used); expression ~ (1 | condition) + (1 | sample__id) + (1 | peptide__id) + (1 | cohort) , expression ~ (1 | condition) + (1 | cohort) , expression ~ (1 | condition)

[progress] msqrob took 1.1 minutes

[info] differential detection analysis: min__samples__observed=2

[progress] peptide to protein rollup with MaxLFQ (implementation: iq) took 2 seconds

[progress] peptide to protein rollup with MaxLFQ (implementation: iq) took 1 seconds

[progress] peptide to protein rollup with MaxLFQ (implementation: iq) took 1 seconds

[progress] peptide to protein rollup with MaxLFQ (implementation: iq) took 1 seconds

[progress] peptide to protein rollup with MaxLFQ (implementation: iq) took 1 seconds

[progress] creating PDF report…

[progress] peptide to protein rollup with MaxLFQ (implementation: iq) took 2 seconds

[progress] report: constructing plots specific for each contrast

[progress] report: rendering report (this may take a while depending on dataset size)

[progress] RT plots: preparing data took 26 seconds

[progress] RT plots: creating plots took 7 seconds

[info] skipping within-group foldchange plots for sample group 'unknown__Kiel', require at least 2 replicates

[info] skipping within-group foldchange plots for sample group 'unknown__Sweden', require at least 2 replicates

[info] No data available for CoV leave-one-out computation in sample group 'unknown__Kiel', skipping plots

[info] No data available for CoV leave-one-out computation in sample group 'unknown__Sweden', skipping plots

[progress] leave-one-out CoV plot computations took 11 seconds

[info] no CoV computation for sample group 'unknown__Kiel', require at least 3 replicates

[info] no CoV computation for sample group 'unknown__Magdeburg', require at least 3 replicates

[info] no CoV computation for sample group 'unknown__Sweden', require at least 3 replicates

[progress] peptide to protein rollup with MaxLFQ (implementation: iq) took 1 seconds

[info] no CoV computation for sample group 'unknown__Kiel', require at least 3 replicates

[info] no CoV computation for sample group 'unknown__Magdeburg', require at least 3 replicates

[info] no CoV computation for sample group 'unknown__Sweden', require at least 3 replicates

5 R command history

This shows the history commands from your R script that starts this pipeline, thereby automatically documenting the parameters/settings used. All lines of executed code since (last) importing data using this R package are shown.

Using this feature

Do not use RStudio's `source` option to execute our pipeline since it will only write `source(...yourscript.R)` to the session history, and consequentially that is all you see in this 'code log'. Instead, select all lines in your script (`control + A`) and then "run" the selected code (either click the run button in RStudio, or use `control + enter`). All lines shown in this section are the same as shown in the RStudio 'History' pane (a tab on the top-right of its UI).

```
dataset = import_dataset_spectronaut(
  filename = "E:/DATA/PMID32485097/20200914_185722_SNA-fSN1.1_CLib1.1 with deamidation_SNA-SER1.1 BGS default_all final raw files Zet Ma
  confidence_threshold = 0.05,
  use_normalized_intensities = FALSE,
  use_irt = TRUE, do_plot = TRUE
)
dataset = import_fasta(dataset,
  files = c(
    "C:/VU/fasta/UniProt_2018-05/UP000005640_9606.fasta",
    "C:/VU/fasta/UniProt_2018-05/UP000005640_9606_additional.fasta",
    "C:/VU/fasta/UniProt_2017-06/UP000005640_9606.fasta",
    "C:/VU/fasta/UniProt_2017-06/UP000005640_9606_additional.fasta"
  )
)
dataset = import_sample_metadata(dataset,
  filename = "E:/DATA/PMID32485097/sample_metadata.xlsx"
)
dataset = setup_contrasts(dataset,
  contrast_list = list(list(
    c(
      "control_Berlin",
      "control_Magdeburg",
      "control_Sweden"
    ),
    c(
      "AD_Berlin",
      "AD_Magdeburg",
      "AD_Sweden"
    )
  )),
  random_variables = "cohort"
)
dataset = analysis_quickstart(dataset,
  filter_min_detect = 8,
  filter_by_contrast = TRUE,
  filter_topn_peptides = 0,
  filter_min_peptide_per_prot = 1,
  norm_algorithm = c(
    "vsn",
    "modebetween_protein"
  ),
  dea_algorithm = c(
    "deqms",
    "msqrob"
  ), dea_qvalue_threshold = 0.01,
  dea_log2foldchange_threshold = NA,
  diffdetect_min_samples_observed = 2,
  output_qc_report = TRUE,
  output_abundance_tables = TRUE,
  output_dir = "E:/DATA/PMID32485097/",
  output_within_timestamped_subdirectory = TRUE,
  dump_all_data = TRUE
)
```

6 R session info

The computer system and versioning of all R packages used to run this analysis are shown below to facilitate, in combination with the previous section, reproducibility.

setting	value
version	R version 4.2.1 (2022-06-23 ucrt)
os	Windows 10 x64 (build 19043)
system	x86_64, mingw32
ui	RStudio
language	(EN)
collate	English_United States.utf8
ctype	English_United States.utf8
tz	Europe/Berlin
date	2022-10-30
rstudio	2022.07.0+548 Spotted Wakerobin (desktop)
pandoc	2.18 @ C:/Program Files/RStudio/bin/quarto/bin/tools/ (via rmarkdown)

System

package	loadedversion	source
dplyr	1.0.9	CRAN (R 4.2.1)
ggplot2	3.3.6	CRAN (R 4.2.1)
msdap	1.0.3	
rlang	1.0.3	CRAN (R 4.2.1)
testthat	3.1.4	CRAN (R 4.2.1)
tibble	3.1.7	CRAN (R 4.2.1)
tidyr	1.2.0	CRAN (R 4.2.1)

Attached packages

package	loadedversion	source
abind	1.4-5	CRAN (R 4.2.0)
affy	1.74.0	Bioconductor
affyio	1.66.0	Bioconductor
aod	1.3.2	CRAN (R 4.2.1)
askpass	1.1	CRAN (R 4.2.1)
assertthat	0.2.1	CRAN (R 4.2.1)
backports	1.4.1	CRAN (R 4.2.0)
Biobase	2.56.0	Bioconductor
BiocGenerics	0.42.0	Bioconductor
BiocManager	1.30.18	CRAN (R 4.2.1)
BiocParallel	1.30.3	Bioconductor
bit	4.0.4	CRAN (R 4.2.1)
bit64	4.0.5	CRAN (R 4.2.1)
bitops	1.0-7	CRAN (R 4.2.0)
blob	1.2.3	CRAN (R 4.2.1)
boot	1.3-28	CRAN (R 4.2.1)
brio	1.1.3	CRAN (R 4.2.1)
broom	1.0.0	CRAN (R 4.2.1)
cachem	1.0.6	CRAN (R 4.2.1)
callr	3.7.0	CRAN (R 4.2.1)
car	3.1-0	CRAN (R 4.2.1)
carData	3.0-5	CRAN (R 4.2.1)
caTools	1.18.2	CRAN (R 4.2.1)
cli	3.3.0	CRAN (R 4.2.1)
clue	0.3-61	CRAN (R 4.2.1)
cluster	2.1.3	CRAN (R 4.2.1)
codetools	0.2-18	CRAN (R 4.2.1)
colorspace	2.0-3	CRAN (R 4.2.1)
cowplot	1.1.1	CRAN (R 4.2.1)
crayon	1.5.1	CRAN (R 4.2.1)
data.table	1.14.2	CRAN (R 4.2.1)
DBI	1.1.3	CRAN (R 4.2.1)
DEqMS	1.14.0	bioc_xgit (@a30da3599ce70afb20527ef3c001d4c15a51a662)
desc	1.4.1	CRAN (R 4.2.1)
devtools	2.4.3	CRAN (R 4.2.1)
diann	1.0.1	Github (vdemichev/diann-rpackage@af538f6e2cd5ab715e1381632e17cb8f234ebf53)
digest	0.6.29	CRAN (R 4.2.1)
doParallel	1.0.17	CRAN (R 4.2.1)
doRNG	1.8.2	CRAN (R 4.2.1)
ellipsis	0.3.2	CRAN (R 4.2.1)
evaluate	0.15	CRAN (R 4.2.1)
fansi	1.0.3	CRAN (R 4.2.1)
farver	2.1.1	CRAN (R 4.2.1)
fastmap	1.1.0	CRAN (R 4.2.1)
forcats	0.5.1	CRAN (R 4.2.1)
foreach	1.5.2	CRAN (R 4.2.1)
formatR	1.12	CRAN (R 4.2.1)
fs	1.5.2	CRAN (R 4.2.1)
generics	0.1.3	CRAN (R 4.2.1)
ggpubr	0.4.0	CRAN (R 4.2.1)

package	loadedversion	source
ggrepel	0.9.1	CRAN (R 4.2.1)
ggsignif	0.6.3	CRAN (R 4.2.1)
glue	1.6.2	CRAN (R 4.2.1)
gplots	3.1.3	CRAN (R 4.2.1)
gridExtra	2.3	CRAN (R 4.2.1)
gtable	0.3.0	CRAN (R 4.2.1)
gtools	3.9.2.2	CRAN (R 4.2.1)
hms	1.1.1	CRAN (R 4.2.1)
htmltools	0.5.2	CRAN (R 4.2.1)
impute	1.70.0	Bioconductor
iq	1.9.6	CRAN (R 4.2.1)
IRanges	2.30.0	Bioconductor
iterators	1.0.14	CRAN (R 4.2.1)
itertools	0.1-3	CRAN (R 4.2.1)
KernSmooth	2.23-20	CRAN (R 4.2.1)
knitr	1.39	CRAN (R 4.2.1)
labeling	0.4.2	CRAN (R 4.2.0)
lattice	0.20-45	CRAN (R 4.2.1)
lifecycle	1.0.1	CRAN (R 4.2.1)
limma	3.52.2	Bioconductor
lme4	1.1-30	CRAN (R 4.2.1)
magrittr	2.0.3	CRAN (R 4.2.1)
MALDIquant	1.21	CRAN (R 4.2.1)
MASS	7.3-57	CRAN (R 4.2.1)
Matrix	1.5-1	CRAN (R 4.2.1)
matrixStats	0.62.0	CRAN (R 4.2.1)
memoise	2.0.1	CRAN (R 4.2.1)
mgcv	1.8-40	CRAN (R 4.2.1)
minqa	1.2.4	CRAN (R 4.2.1)
missForest	1.5	CRAN (R 4.2.1)
MsCoreUtils	1.8.0	Bioconductor
msEmpiRe	0.1.0	Github (zimmerlab/MS-EmpiRe@8a85757c8d604014130ee9d379aa8cfeb05e3855)
MSnbase	2.22.0	Bioconductor
munsell	0.5.0	CRAN (R 4.2.1)
mzID	1.34.0	Bioconductor
mzR	2.30.0	Bioconductor
ncdf4	1.19	CRAN (R 4.2.0)
nlme	3.1-157	CRAN (R 4.2.1)
nloptr	2.0.3	CRAN (R 4.2.1)
openssl	2.0.2	CRAN (R 4.2.1)
openxlsx	4.2.5	CRAN (R 4.2.1)
patchwork	1.1.1	CRAN (R 4.2.1)
pbkrtest	0.5.1	CRAN (R 4.2.1)
pcaMethods	1.88.0	Bioconductor
pdftools	3.3.0	CRAN (R 4.2.1)
Peptides	2.4.4	CRAN (R 4.2.1)
pillar	1.7.0	CRAN (R 4.2.1)
pkgbuild	1.3.1	CRAN (R 4.2.1)
pkgconfig	2.0.3	CRAN (R 4.2.1)
pkgload	1.3.0	CRAN (R 4.2.1)

package	loadedversion	source
plyr	1.8.7	CRAN (R 4.2.1)
preprocessCore	1.58.0	Bioconductor
prettyunits	1.1.1	CRAN (R 4.2.1)
pROC	1.18.0	CRAN (R 4.2.1)
processx	3.7.0	CRAN (R 4.2.1)
progress	1.2.2	CRAN (R 4.2.1)
ProtGenerics	1.28.0	Bioconductor
ps	1.7.1	CRAN (R 4.2.1)
purrr	0.3.4	CRAN (R 4.2.1)
qpdf	1.2.0	CRAN (R 4.2.1)
R.cache	0.15.0	CRAN (R 4.2.1)
R.methodsS3	1.8.2	CRAN (R 4.2.0)
R.oo	1.25.0	CRAN (R 4.2.0)
R.utils	2.12.0	CRAN (R 4.2.1)
R6	2.5.1	CRAN (R 4.2.1)
randomForest	4.7-1.1	CRAN (R 4.2.1)
rbibutils	2.2.8	CRAN (R 4.2.1)
RColorBrewer	1.1-3	CRAN (R 4.2.0)
Rcpp	1.0.9	CRAN (R 4.2.1)
RcppEigen	0.3.3.9.2	CRAN (R 4.2.1)
Rdpack	2.3.1	CRAN (R 4.2.1)
readr	2.1.2	CRAN (R 4.2.1)
remotes	2.4.2	CRAN (R 4.2.1)
reshape2	1.4.4	CRAN (R 4.2.1)
RhpcBLASctl	0.21-247.1	CRAN (R 4.2.0)
rmarkdown	2.14	CRAN (R 4.2.1)
rngtools	1.5.2	CRAN (R 4.2.1)
rprojroot	2.0.3	CRAN (R 4.2.1)
RSQLite	2.2.14	CRAN (R 4.2.1)
rstatix	0.7.0	CRAN (R 4.2.1)
rstudioapi	0.13	CRAN (R 4.2.1)
S4Vectors	0.34.0	Bioconductor
scales	1.2.0	CRAN (R 4.2.1)
sessioninfo	1.2.2	CRAN (R 4.2.1)
stringi	1.7.6	CRAN (R 4.2.0)
stringr	1.4.0	CRAN (R 4.2.1)
styler	1.7.0	CRAN (R 4.2.1)
tidyselect	1.1.2	CRAN (R 4.2.1)
tinytex	0.40	CRAN (R 4.2.1)
tzdb	0.3.0	CRAN (R 4.2.1)
usethis	2.1.6	CRAN (R 4.2.1)
utf8	1.2.2	CRAN (R 4.2.1)
variancePartition	1.26.0	bioc_xgit (@b1731297b2f7335ef9155c6ac7df0e246787d9d6)
vctrs	0.4.1	CRAN (R 4.2.1)
viridis	0.6.2	CRAN (R 4.2.1)
viridisLite	0.4.0	CRAN (R 4.2.1)
vroom	1.5.7	CRAN (R 4.2.1)
vsn	3.64.0	Bioconductor
withr	2.5.0	CRAN (R 4.2.1)
xfun	0.31	CRAN (R 4.2.1)

package	loadedversion	source
XML	3.99-0.10	CRAN (R 4.2.0)
xtable	1.8-4	CRAN (R 4.2.1)
yaml	2.3.5	CRAN (R 4.2.0)
zip	2.2.0	CRAN (R 4.2.1)
zlibbioc	1.42.0	Bioconductor

Packages that are not attached
