# Supplementary material for: MS-DAP Platform for Downstream Data Analysis of Label-Free Proteomics Uncovers Optimal Workflows in Benchmark Data Sets and Increased Sensitivity in Analysis of Alzheimer’s Biomarker Data
Source: J Proteome Res. 2022 Dec 21;22(2):374–86. doi: 10.1021/acs.jproteome.2c00513 (PMC9903323; doi:10.1021/acs.jproteome.2c00513)
Supplement: Supplementary file 5 — pr2c00513_si_005.pdf [file pr2c00513_si_005.pdf]

Supporting Data 2

MS-DAP platform for downstream data analysis of label-free proteomics uncovers optimal workflows in benchmark datasets and increased sensitivity in analysis of Alzheimer's biomarker data

Frank Koopmans[1], Ka Wan Li[1], Remco V. Klaassen[1], August B. Smit[1]*

[1] Department of Molecular and Cellular Neurobiology, Center for Neurogenomics and Cognitive Research, Amsterdam Neuroscience, VU University, 1081HV Amsterdam, The Netherlands.

Figure legends

Each page describes 1 dataset, its name is shown on the second line in the top-left of each page. For protein-level DEA algorithms eBayes and DEqMS, 3 peptide-to-protein rollup methods were used for comparison; "sum", Tukey's Median Polish (TMP), MaxLFQ.

- Top-left: log2-foldchange distribtutions for all proteins in the dataset, color-coded by classification. Background proteins (the set that should be unchanged between experimental conditions) is shown in black, the foreground proteins (spike-in that should be changed) are shown in blue. For 3-proteome-mixtures (like in the LFQbench studies), the third color represents the strongest spike-in population (E.Coli) that is not taken into account.

- Bottom-left: all evaluated DEA methods use FDR for p-value adjustment (i.e. respective tools are used as-is). The true- and false-positives are known in the benchmark datasets and here used to compute empirical False Positive Rates (FPR) at each FDR cutoff.

- ROC plots on top- and bottom-rows: Receiver Operating Characteristic (ROC) curves for all evaluated DEA algorithms. The partial area under the curve (pAUC) at 95% specificity was added to each DEA's label.

- Barplots are shown to compare true- and false-positive protein counts for 4 different filters applied to DEA results that one might use to define significant hits: only filter by q-value, filter by both q-value and log2-foldchange (threshold estimated by MS-DAP bootstrapping algorithm), or either of these combined with the requirement of at least 2 peptides-per-protein.

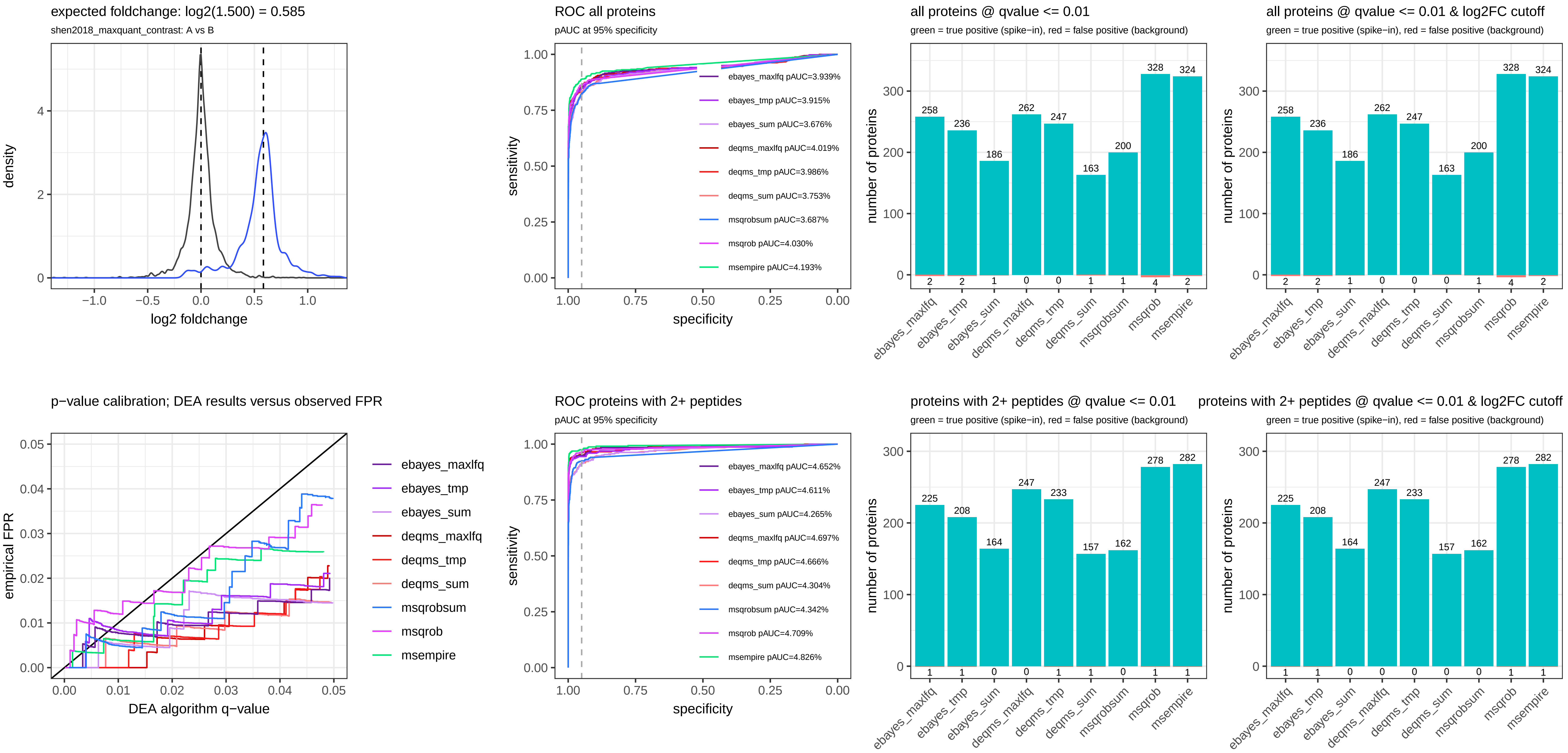

expected foldchange: log2(1.500) = 0.585
shen2018_maxquant_contrast: A vs B
density
log2 foldchange
ROC all proteins
pAUC at 95% specificity
sensitivity
specificity
ebayes_maxlfq pAUC=3.939%
ebayes_tmp pAUC=3.915%
ebayes_sum pAUC=3.676%
deqms_maxlfq pAUC=4.019%
deqms_tmp pAUC=3.986%
deqms_sum pAUC=3.753%
msqrobsum pAUC=3.687%
msqrob pAUC=4.030%
msempire pAUC=4.193%
all proteins @ qvalue <= 0.01
green = true positive (spike−in), red = false positive (background)
number of proteins
all proteins @ qvalue <= 0.01 & log2FC cutoff
green = true positive (spike−in), red = false positive (background)
p−value calibration; DEA results versus observed FPR
empirical FPR
DEA algorithm q−value
ebayes_maxlfq
ebayes_tmp
ebayes_sum
deqms_maxlfq
deqms_tmp
deqms_sum
msqrobsum
msqrob
msempire
ROC proteins with 2+ peptides
pAUC at 95% specificity
ebayes_maxlfq pAUC=4.652%
ebayes_tmp pAUC=4.611%
ebayes_sum pAUC=4.265%
deqms_maxlfq pAUC=4.697%
deqms_tmp pAUC=4.666%
deqms_sum pAUC=4.304%
msqrobsum pAUC=4.342%
msqrob pAUC=4.709%
msempire pAUC=4.826%
proteins with 2+ peptides @ qvalue <= 0.01
green = true positive (spike−in), red = false positive (background)
proteins with 2+ peptides @ qvalue <= 0.01 & log2FC cutoff
green = true positive (spike−in), red = false positive (background)

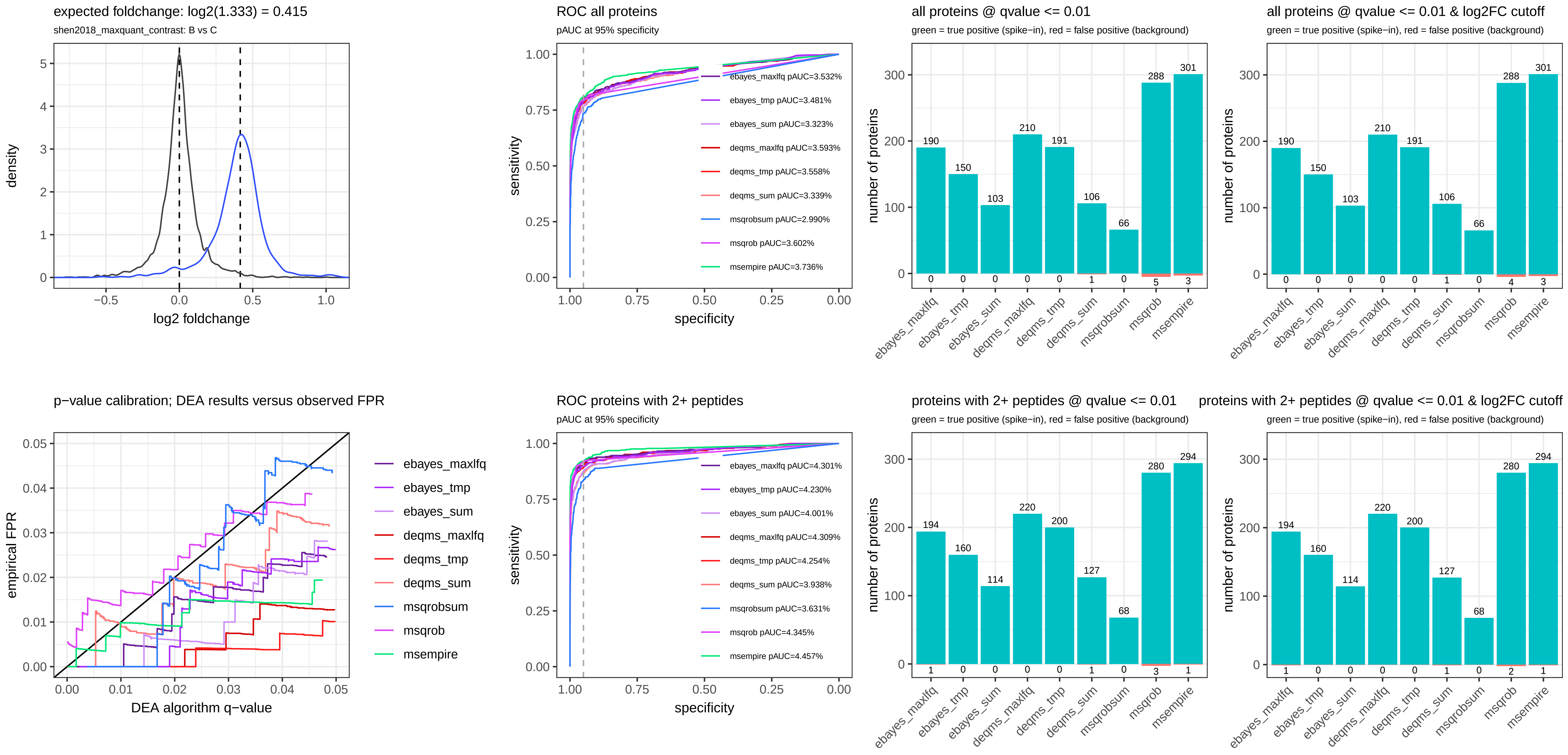

expected foldchange: log2(1.333) = 0.415
shen2018_maxquant_contrast: B vs C
density
log2 foldchange
ROC all proteins
pAUC at 95% specificity
sensitivity
specificity
ebayes_maxlfq pAUC=3.532%
ebayes_tmp pAUC=3.481%
ebayes_sum pAUC=3.323%
deqms_maxlfq pAUC=3.593%
deqms_tmp pAUC=3.558%
deqms_sum pAUC=3.339%
msqrobsum pAUC=2.990%
msqrob pAUC=3.602%
msempire pAUC=3.736%
all proteins @ qvalue <= 0.01
green = true positive (spike−in), red = false positive (background)
number of proteins
190 150 103 210 191 106 66 288 301
0 0 0 0 0 1 0 5 3
all proteins @ qvalue <= 0.01 & log2FC cutoff
green = true positive (spike−in), red = false positive (background)
190 150 103 210 191 106 66 288 301
0 0 0 0 0 1 0 4 3
ebayes_maxlfq ebayes_tmp ebayes_sum deqms_maxlfq deqms_tmp deqms_sum msqrobsum msqrob msempire
p−value calibration; DEA results versus observed FPR
empirical FPR
DEA algorithm q−value
ebayes_maxlfq
ebayes_tmp
ebayes_sum
deqms_maxlfq
deqms_tmp
deqms_sum
msqrobsum
msqrob
msempire
ROC proteins with 2+ peptides
pAUC at 95% specificity
ebayes_maxlfq pAUC=4.301%
ebayes_tmp pAUC=4.230%
ebayes_sum pAUC=4.001%
deqms_maxlfq pAUC=4.309%
deqms_tmp pAUC=4.254%
deqms_sum pAUC=3.938%
msqrobsum pAUC=3.631%
msqrob pAUC=4.345%
msempire pAUC=4.457%
proteins with 2+ peptides @ qvalue <= 0.01
green = true positive (spike−in), red = false positive (background)
194 160 114 220 200 127 68 280 294
1 0 0 0 0 1 0 3 1
proteins with 2+ peptides @ qvalue <= 0.01 & log2FC cutoff
green = true positive (spike−in), red = false positive (background)
194 160 114 220 200 127 68 280 294
1 0 0 0 0 1 0 2 1

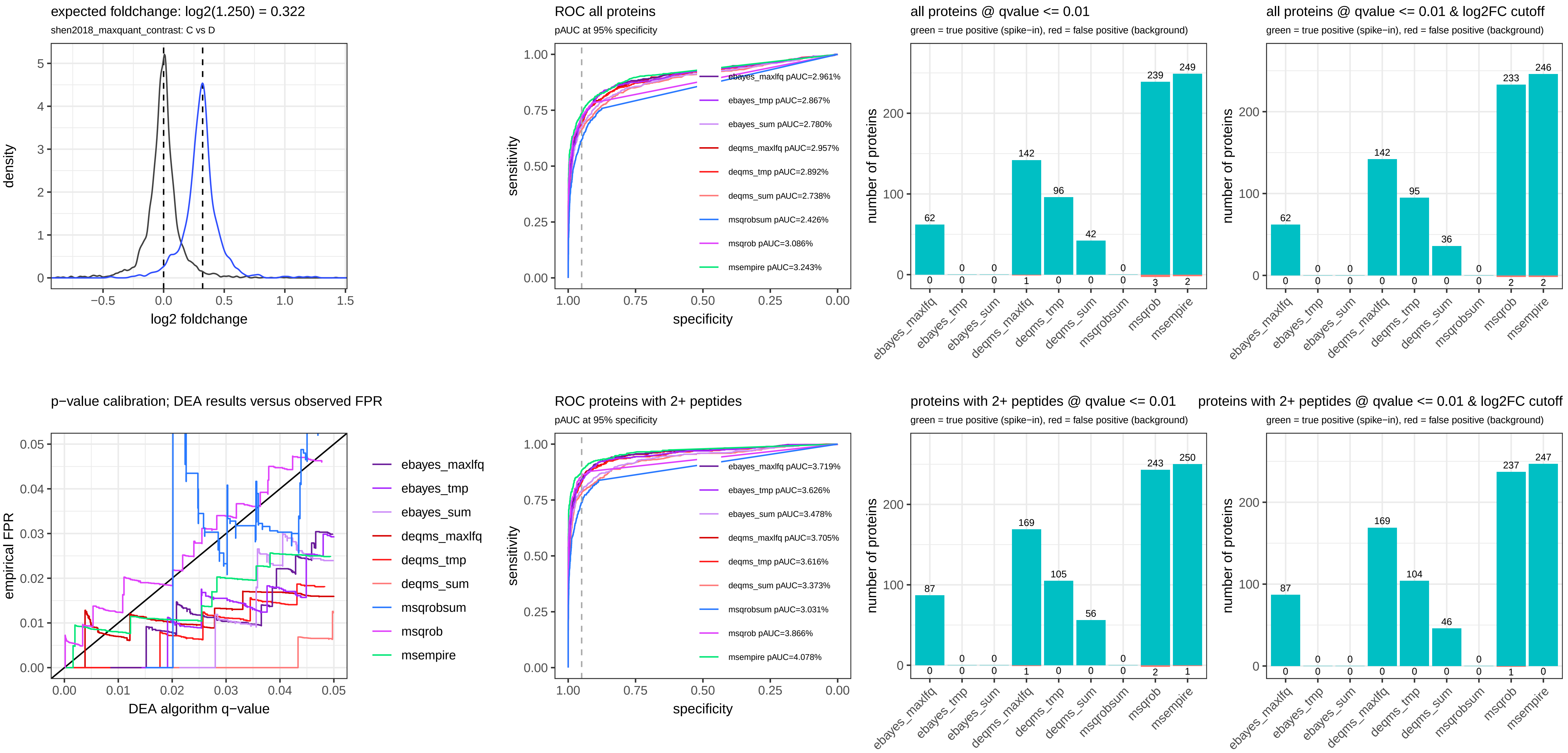
expected foldchange: log2(1.250) = 0.322
shen2018_maxquant_contrast: C vs D
density
log2 foldchange
ROC all proteins
pAUC at 95% specificity
sensitivity
specificity
ebayes_maxlfq pAUC=2.961%
ebayes_tmp pAUC=2.867%
ebayes_sum pAUC=2.780%
deqms_maxlfq pAUC=2.957%
deqms_tmp pAUC=2.892%
deqms_sum pAUC=2.738%
msqrobsum pAUC=2.426%
msqrob pAUC=3.086%
msempire pAUC=3.243%
all proteins @ qvalue <= 0.01
green = true positive (spike−in), red = false positive (background)
number of proteins
all proteins @ qvalue <= 0.01 & log2FC cutoff
green = true positive (spike−in), red = false positive (background)
p−value calibration; DEA results versus observed FPR
empirical FPR
DEA algorithm q−value
ebayes_maxlfq
ebayes_tmp
ebayes_sum
deqms_maxlfq
deqms_tmp
deqms_sum
msqrobsum
msqrob
msempire
ROC proteins with 2+ peptides
pAUC at 95% specificity
ebayes_maxlfq pAUC=3.719%
ebayes_tmp pAUC=3.626%
ebayes_sum pAUC=3.478%
deqms_maxlfq pAUC=3.705%
deqms_tmp pAUC=3.616%
deqms_sum pAUC=3.373%
msqrobsum pAUC=3.031%
msqrob pAUC=3.866%
msempire pAUC=4.078%
proteins with 2+ peptides @ qvalue <= 0.01
green = true positive (spike−in), red = false positive (background)
proteins with 2+ peptides @ qvalue <= 0.01 & log2FC cutoff
green = true positive (spike−in), red = false positive (background)

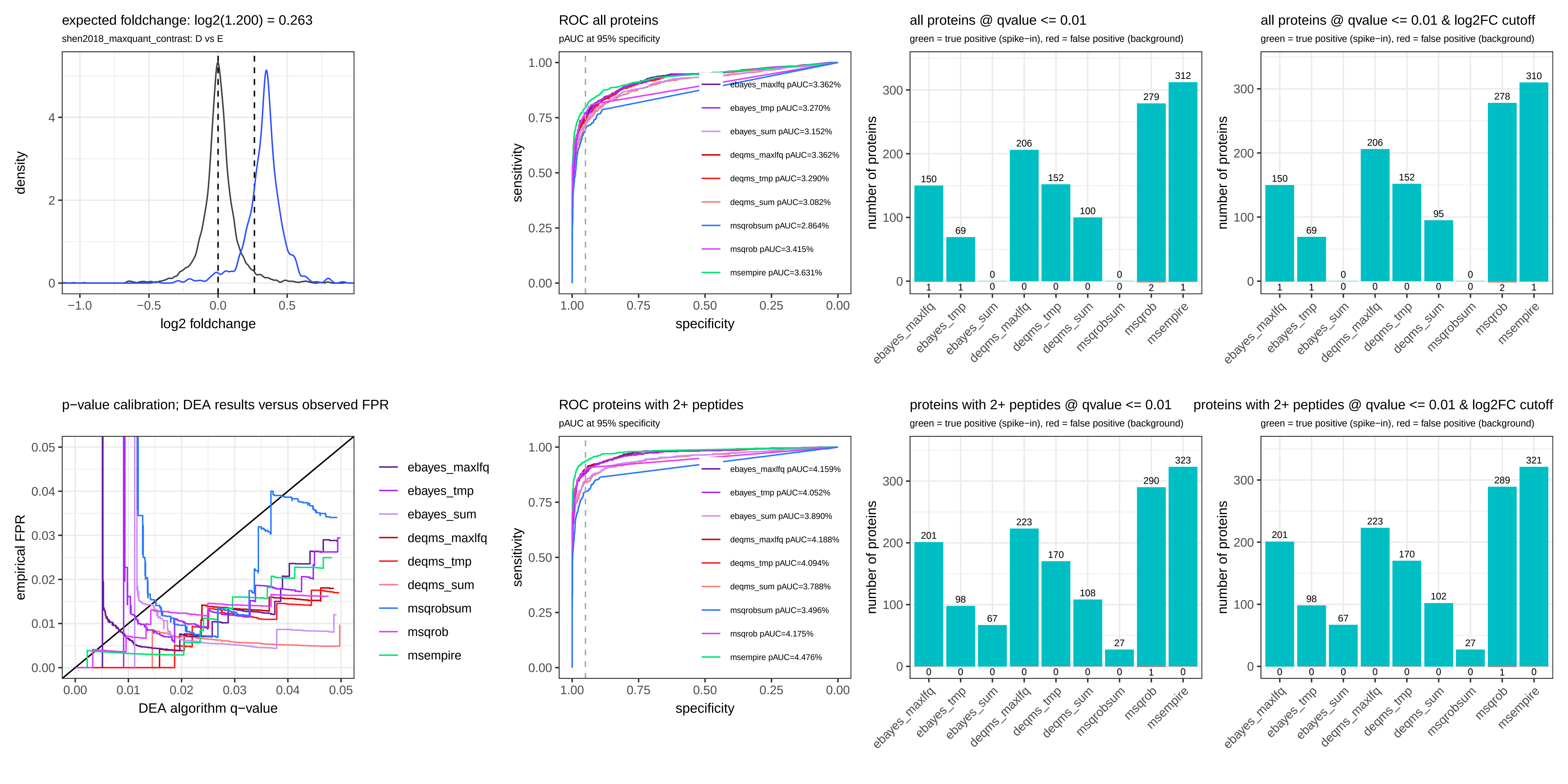

expected foldchange: log2(1.200) = 0.263
shen2018_maxquant_contrast: D vs E
density
log2 foldchange
ROC all proteins
pAUC at 95% specificity
sensitivity
specificity
ebayes_maxlfq pAUC=3.362%
ebayes_tmp pAUC=3.270%
ebayes_sum pAUC=3.152%
deqms_maxlfq pAUC=3.362%
deqms_tmp pAUC=3.290%
deqms_sum pAUC=3.082%
msqrobsum pAUC=2.864%
msqrob pAUC=3.415%
msempire pAUC=3.631%
all proteins @ qvalue <= 0.01
green = true positive (spike−in), red = false positive (background)
number of proteins
150 69 0 206 152 100 0 279 312
1 1 0 0 0 0 0 2 1
all proteins @ qvalue <= 0.01 & log2FC cutoff
green = true positive (spike−in), red = false positive (background)
150 69 0 206 152 95 0 278 310
1 1 0 0 0 0 0 2 1
ebayes_maxlfq ebayes_tmp ebayes_sum deqms_maxlfq deqms_tmp deqms_sum msqrobsum msqrob msempire
p−value calibration; DEA results versus observed FPR
empirical FPR
DEA algorithm q−value
ebayes_maxlfq
ebayes_tmp
ebayes_sum
deqms_maxlfq
deqms_tmp
deqms_sum
msqrobsum
msqrob
msempire
ROC proteins with 2+ peptides
pAUC at 95% specificity
ebayes_maxlfq pAUC=4.159%
ebayes_tmp pAUC=4.052%
ebayes_sum pAUC=3.890%
deqms_maxlfq pAUC=4.188%
deqms_tmp pAUC=4.094%
deqms_sum pAUC=3.788%
msqrobsum pAUC=3.496%
msqrob pAUC=4.175%
msempire pAUC=4.476%
proteins with 2+ peptides @ qvalue <= 0.01
green = true positive (spike−in), red = false positive (background)
201 98 67 223 170 108 27 290 323
0 0 0 0 0 0 0 1 0
proteins with 2+ peptides @ qvalue <= 0.01 & log2FC cutoff
green = true positive (spike−in), red = false positive (background)
201 98 67 223 170 102 27 289 321
0 0 0 0 0 0 0 1 0

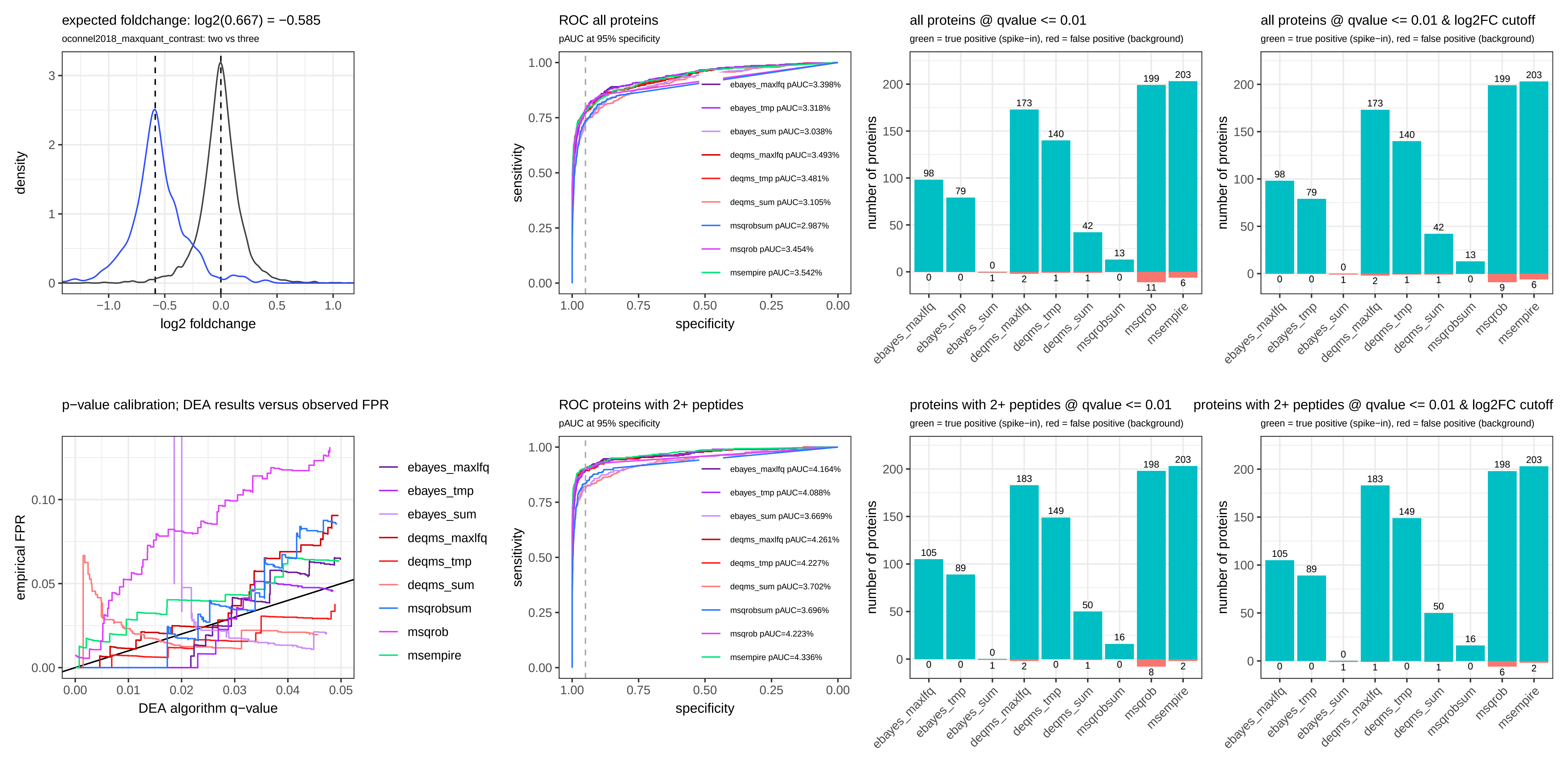

expected foldchange: log2(0.667) = −0.585
oconnel2018_maxquant_contrast: two vs three
density
log2 foldchange
ROC all proteins
pAUC at 95% specificity
sensitivity
specificity
ebayes_maxlfq pAUC=3.398%
ebayes_tmp pAUC=3.318%
ebayes_sum pAUC=3.038%
deqms_maxlfq pAUC=3.493%
deqms_tmp pAUC=3.481%
deqms_sum pAUC=3.105%
msqrobsum pAUC=2.987%
msqrob pAUC=3.454%
msempire pAUC=3.542%
all proteins @ qvalue <= 0.01
green = true positive (spike−in), red = false positive (background)
number of proteins
98 79 0 173 140 42 13 199 203
0 0 1 2 1 1 0 11 6
all proteins @ qvalue <= 0.01 & log2FC cutoff
green = true positive (spike−in), red = false positive (background)
number of proteins
98 79 0 173 140 42 13 199 203
0 0 1 2 1 1 0 9 6
ebayes_maxlfq ebayes_tmp ebayes_sum deqms_maxlfq deqms_tmp deqms_sum msqrobsum msqrob msempire
p−value calibration; DEA results versus observed FPR
empirical FPR
DEA algorithm q−value
ebayes_maxlfq
ebayes_tmp
ebayes_sum
deqms_maxlfq
deqms_tmp
deqms_sum
msqrobsum
msqrob
msempire
ROC proteins with 2+ peptides
pAUC at 95% specificity
sensitivity
specificity
ebayes_maxlfq pAUC=4.164%
ebayes_tmp pAUC=4.088%
ebayes_sum pAUC=3.669%
deqms_maxlfq pAUC=4.261%
deqms_tmp pAUC=4.227%
deqms_sum pAUC=3.702%
msqrobsum pAUC=3.696%
msqrob pAUC=4.223%
msempire pAUC=4.336%
proteins with 2+ peptides @ qvalue <= 0.01
green = true positive (spike−in), red = false positive (background)
number of proteins
105 89 0 183 149 50 16 198 203
0 0 1 2 0 1 0 8 2
proteins with 2+ peptides @ qvalue <= 0.01 & log2FC cutoff
green = true positive (spike−in), red = false positive (background)
number of proteins
105 89 0 183 149 50 16 198 203
0 0 1 1 0 1 0 6 2
ebayes_maxlfq ebayes_tmp ebayes_sum deqms_maxlfq deqms_tmp deqms_sum msqrobsum msqrob msempire

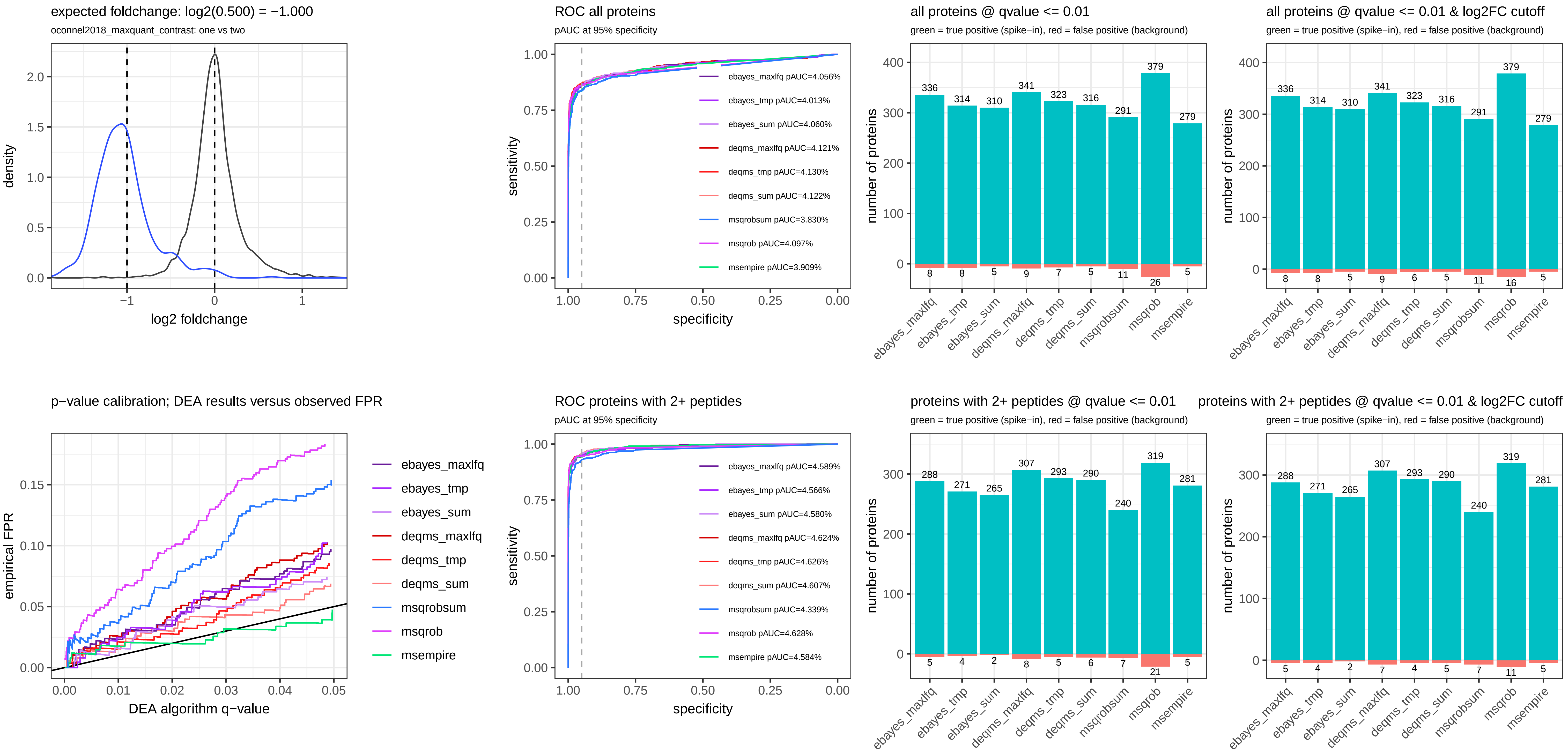

expected foldchange: log2(0.500) = −1.000
oconnel2018_maxquant_contrast: one vs two
density
log2 foldchange
ROC all proteins
pAUC at 95% specificity
sensitivity
specificity
ebayes_maxlfq pAUC=4.056%
ebayes_tmp pAUC=4.013%
ebayes_sum pAUC=4.060%
deqms_maxlfq pAUC=4.121%
deqms_tmp pAUC=4.130%
deqms_sum pAUC=4.122%
msqrobsum pAUC=3.830%
msqrob pAUC=4.097%
msempire pAUC=3.909%
all proteins @ qvalue <= 0.01
green = true positive (spike−in), red = false positive (background)
number of proteins
336 314 310 341 323 316 291 379 279
8 8 5 9 7 5 11 26 5
all proteins @ qvalue <= 0.01 & log2FC cutoff
green = true positive (spike−in), red = false positive (background)
336 314 310 341 323 316 291 379 279
8 8 5 9 6 5 11 16 5
ebayes_maxlfq ebayes_tmp ebayes_sum deqms_maxlfq deqms_tmp deqms_sum msqrobsum msqrob msempire
p−value calibration; DEA results versus observed FPR
empirical FPR
DEA algorithm q−value
ebayes_maxlfq
ebayes_tmp
ebayes_sum
deqms_maxlfq
deqms_tmp
deqms_sum
msqrobsum
msqrob
msempire
ROC proteins with 2+ peptides
pAUC at 95% specificity
ebayes_maxlfq pAUC=4.589%
ebayes_tmp pAUC=4.566%
ebayes_sum pAUC=4.580%
deqms_maxlfq pAUC=4.624%
deqms_tmp pAUC=4.626%
deqms_sum pAUC=4.607%
msqrobsum pAUC=4.339%
msqrob pAUC=4.628%
msempire pAUC=4.584%
proteins with 2+ peptides @ qvalue <= 0.01
green = true positive (spike−in), red = false positive (background)
288 271 265 307 293 290 240 319 281
5 4 2 8 5 6 7 21 5
proteins with 2+ peptides @ qvalue <= 0.01 & log2FC cutoff
green = true positive (spike−in), red = false positive (background)
288 271 265 307 293 290 240 319 281
5 4 2 7 4 5 7 11 5

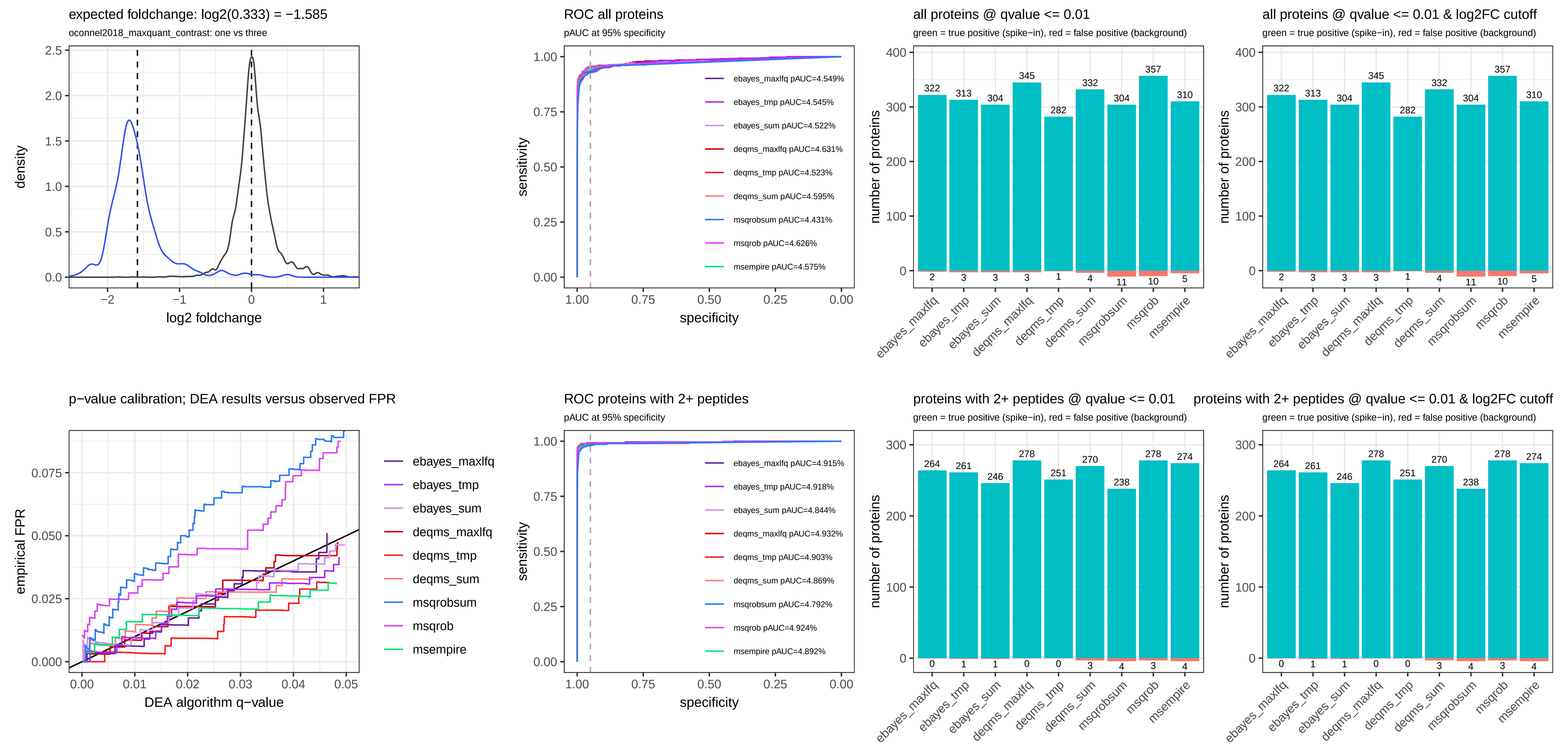
expected foldchange: log2(0.333) = −1.585
oconnel2018_maxquant_contrast: one vs three
density
log2 foldchange
ROC all proteins
pAUC at 95% specificity
sensitivity
specificity
ebayes_maxlfq pAUC=4.549%
ebayes_tmp pAUC=4.545%
ebayes_sum pAUC=4.522%
deqms_maxlfq pAUC=4.631%
deqms_tmp pAUC=4.523%
deqms_sum pAUC=4.595%
msqrobsum pAUC=4.431%
msqrob pAUC=4.626%
msempire pAUC=4.575%
all proteins @ qvalue <= 0.01
green = true positive (spike−in), red = false positive (background)
number of proteins
322 313 304 345 282 332 304 357 310
2 3 3 3 1 4 11 10 5
all proteins @ qvalue <= 0.01 & log2FC cutoff
green = true positive (spike−in), red = false positive (background)
number of proteins
322 313 304 345 282 332 304 357 310
2 3 3 3 1 4 11 10 5
ebayes_maxlfq
ebayes_tmp
ebayes_sum
deqms_maxlfq
deqms_tmp
deqms_sum
msqrobsum
msqrob
msempire
p−value calibration; DEA results versus observed FPR
empirical FPR
DEA algorithm q−value
ebayes_maxlfq
ebayes_tmp
ebayes_sum
deqms_maxlfq
deqms_tmp
deqms_sum
msqrobsum
msqrob
msempire
ROC proteins with 2+ peptides
pAUC at 95% specificity
sensitivity
specificity
ebayes_maxlfq pAUC=4.915%
ebayes_tmp pAUC=4.918%
ebayes_sum pAUC=4.844%
deqms_maxlfq pAUC=4.932%
deqms_tmp pAUC=4.903%
deqms_sum pAUC=4.869%
msqrobsum pAUC=4.792%
msqrob pAUC=4.924%
msempire pAUC=4.892%
proteins with 2+ peptides @ qvalue <= 0.01
green = true positive (spike−in), red = false positive (background)
number of proteins
264 261 246 278 251 270 238 278 274
0 1 1 0 0 3 4 3 4
proteins with 2+ peptides @ qvalue <= 0.01 & log2FC cutoff
green = true positive (spike−in), red = false positive (background)
number of proteins
264 261 246 278 251 270 238 278 274
0 1 1 0 0 3 4 3 4

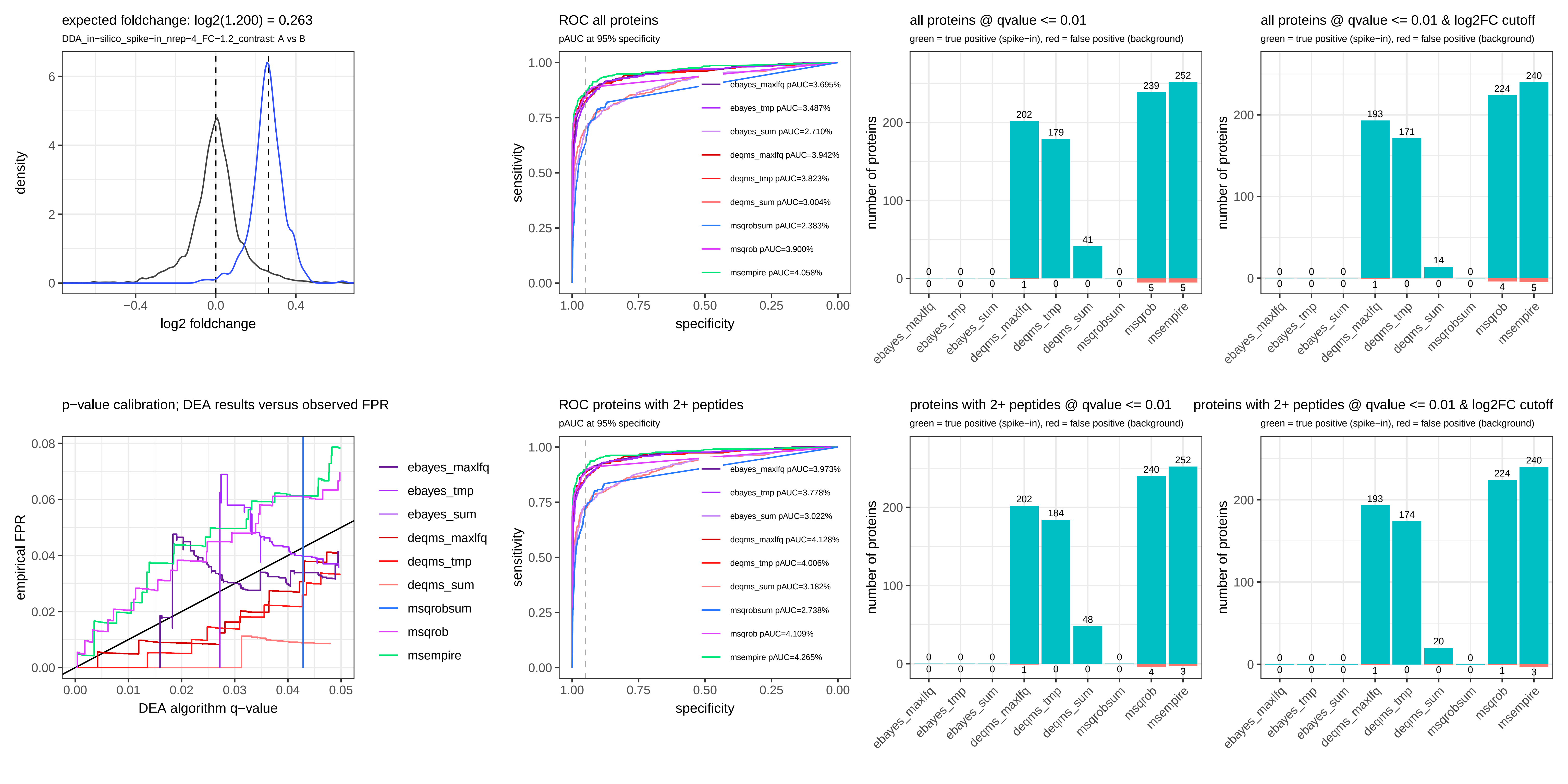
expected foldchange: log2(1.200) = 0.263
DDA_in−silico_spike−in_nrep−4_FC−1.2_contrast: A vs B
density
log2 foldchange
ROC all proteins
pAUC at 95% specificity
sensitivity
specificity
ebayes_maxlfq pAUC=3.695%
ebayes_tmp pAUC=3.487%
ebayes_sum pAUC=2.710%
deqms_maxlfq pAUC=3.942%
deqms_tmp pAUC=3.823%
deqms_sum pAUC=3.004%
msqrobsum pAUC=2.383%
msqrob pAUC=3.900%
msempire pAUC=4.058%
all proteins @ qvalue <= 0.01
green = true positive (spike−in), red = false positive (background)
number of proteins
0 0 0 202 179 41 0 239 252
0 0 0 1 0 0 0 5 5
all proteins @ qvalue <= 0.01 & log2FC cutoff
green = true positive (spike−in), red = false positive (background)
number of proteins
0 0 0 193 171 14 0 224 240
0 0 0 1 0 0 0 4 5
ebayes_maxlfq
ebayes_tmp
ebayes_sum
deqms_maxlfq
deqms_tmp
deqms_sum
msqrobsum
msqrob
msempire
p−value calibration; DEA results versus observed FPR
empirical FPR
DEA algorithm q−value
ebayes_maxlfq
ebayes_tmp
ebayes_sum
deqms_maxlfq
deqms_tmp
deqms_sum
msqrobsum
msqrob
msempire
ROC proteins with 2+ peptides
pAUC at 95% specificity
sensitivity
specificity
ebayes_maxlfq pAUC=3.973%
ebayes_tmp pAUC=3.778%
ebayes_sum pAUC=3.022%
deqms_maxlfq pAUC=4.128%
deqms_tmp pAUC=4.006%
deqms_sum pAUC=3.182%
msqrobsum pAUC=2.738%
msqrob pAUC=4.109%
msempire pAUC=4.265%
proteins with 2+ peptides @ qvalue <= 0.01
green = true positive (spike−in), red = false positive (background)
number of proteins
0 0 0 202 184 48 0 240 252
0 0 0 1 0 0 0 4 3
proteins with 2+ peptides @ qvalue <= 0.01 & log2FC cutoff
green = true positive (spike−in), red = false positive (background)
number of proteins
0 0 0 193 174 20 0 224 240
0 0 0 1 0 0 0 1 3
ebayes_maxlfq
ebayes_tmp
ebayes_sum
deqms_maxlfq
deqms_tmp
deqms_sum
msqrobsum
msqrob
msempire

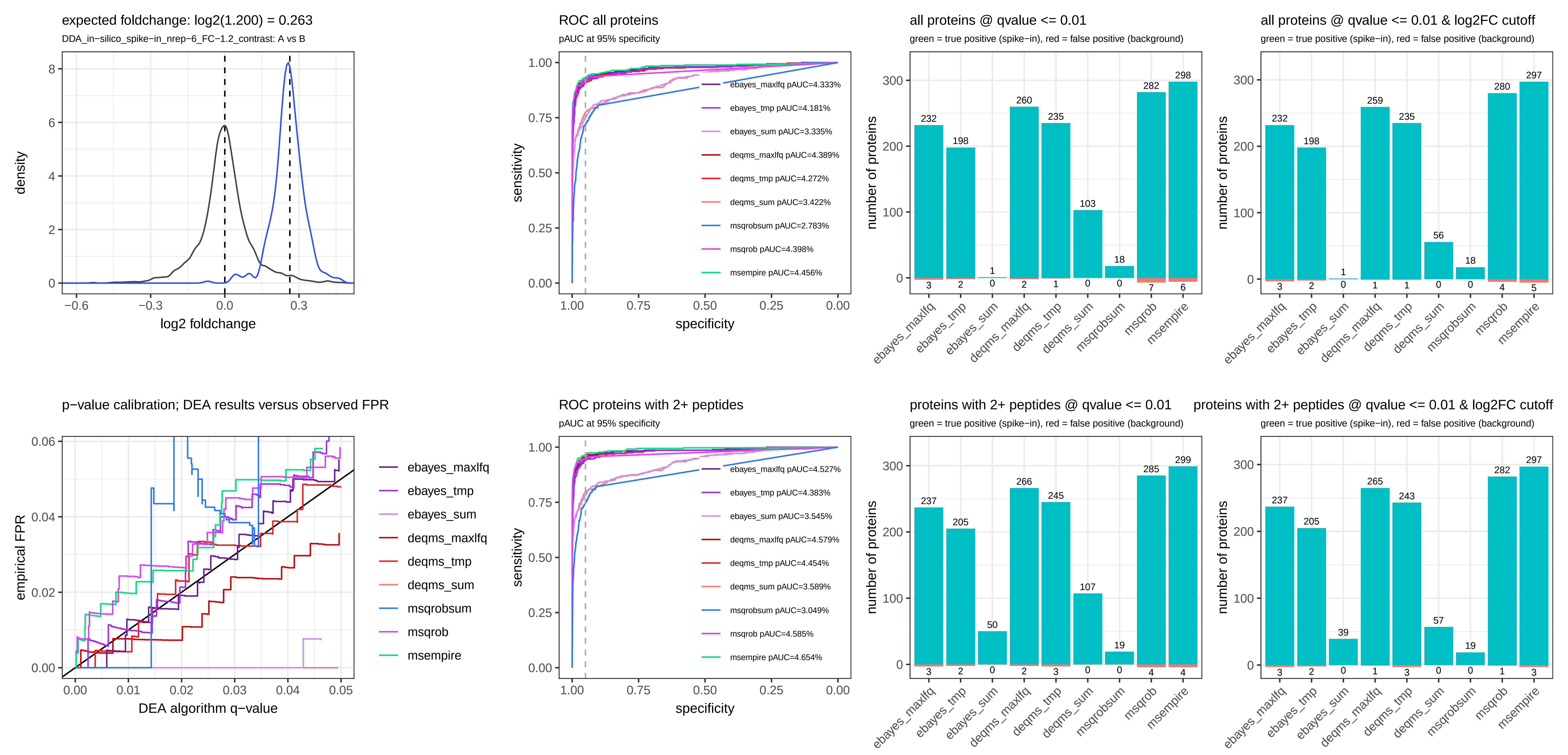

expected foldchange: log2(1.200) = 0.263
DDA_in−silico_spike−in_nrep−6_FC−1.2_contrast: A vs B
density
log2 foldchange
ROC all proteins
pAUC at 95% specificity
sensitivity
specificity
ebayes_maxlfq pAUC=4.333%
ebayes_tmp pAUC=4.181%
ebayes_sum pAUC=3.335%
deqms_maxlfq pAUC=4.389%
deqms_tmp pAUC=4.272%
deqms_sum pAUC=3.422%
msqrobsum pAUC=2.783%
msqrob pAUC=4.398%
msempire pAUC=4.456%
all proteins @ qvalue <= 0.01
green = true positive (spike−in), red = false positive (background)
number of proteins
all proteins @ qvalue <= 0.01 & log2FC cutoff
green = true positive (spike−in), red = false positive (background)
p−value calibration; DEA results versus observed FPR
empirical FPR
DEA algorithm q−value
ebayes_maxlfq
ebayes_tmp
ebayes_sum
deqms_maxlfq
deqms_tmp
deqms_sum
msqrobsum
msqrob
msempire
ROC proteins with 2+ peptides
pAUC at 95% specificity
ebayes_maxlfq pAUC=4.527%
ebayes_tmp pAUC=4.383%
ebayes_sum pAUC=3.545%
deqms_maxlfq pAUC=4.579%
deqms_tmp pAUC=4.454%
deqms_sum pAUC=3.589%
msqrobsum pAUC=3.049%
msqrob pAUC=4.585%
msempire pAUC=4.654%
proteins with 2+ peptides @ qvalue <= 0.01
green = true positive (spike−in), red = false positive (background)
proteins with 2+ peptides @ qvalue <= 0.01 & log2FC cutoff
green = true positive (spike−in), red = false positive (background)

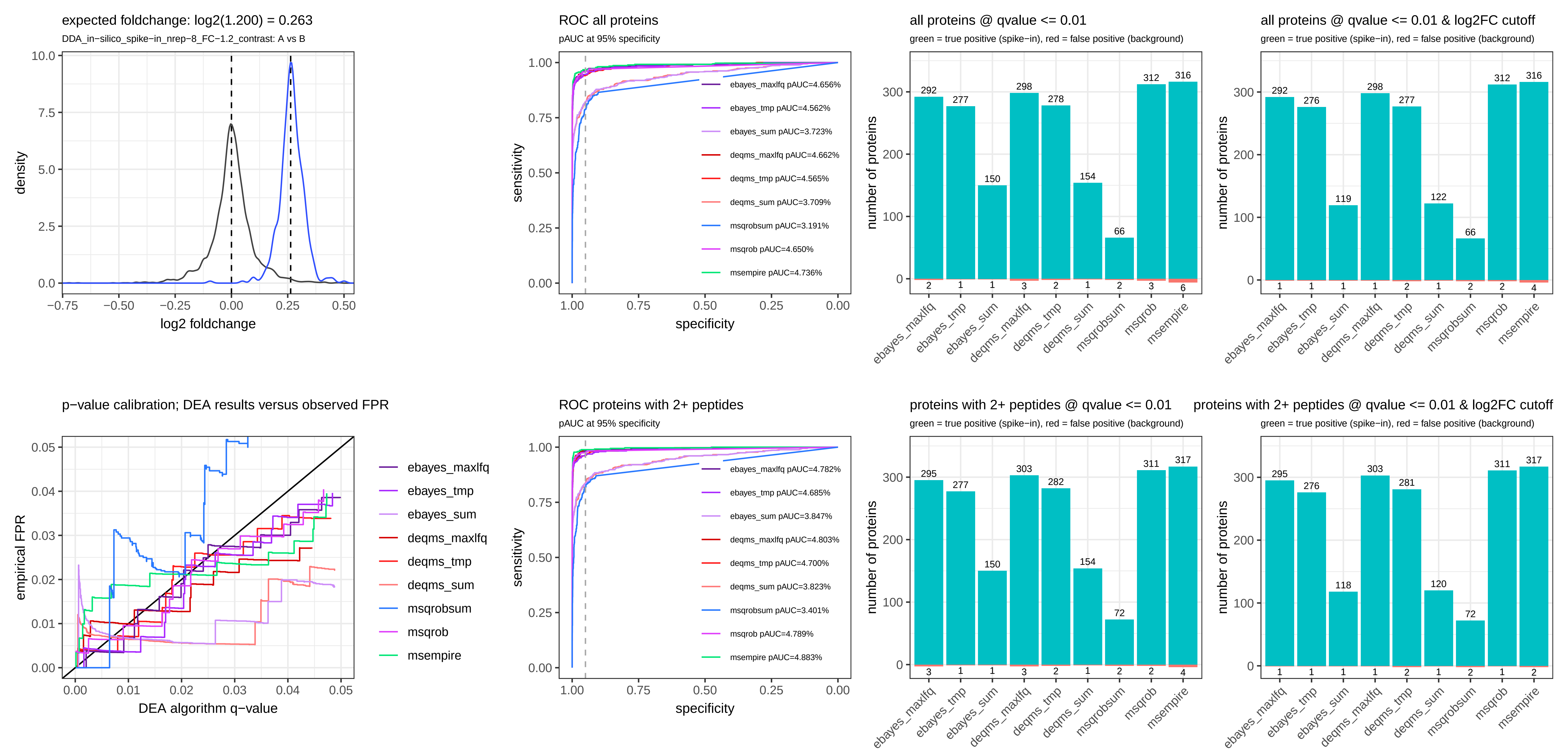

expected foldchange: log2(1.200) = 0.263
DDA_in−silico_spike−in_nrep−8_FC−1.2_contrast: A vs B
density
log2 foldchange
ROC all proteins
pAUC at 95% specificity
sensitivity
specificity
ebayes_maxlfq pAUC=4.656%
ebayes_tmp pAUC=4.562%
ebayes_sum pAUC=3.723%
deqms_maxlfq pAUC=4.662%
deqms_tmp pAUC=4.565%
deqms_sum pAUC=3.709%
msqrobsum pAUC=3.191%
msqrob pAUC=4.650%
msempire pAUC=4.736%
all proteins @ qvalue <= 0.01
green = true positive (spike−in), red = false positive (background)
number of proteins
292 277 150 298 278 154 66 312 316
2 1 1 3 2 1 2 3 6
all proteins @ qvalue <= 0.01 & log2FC cutoff
green = true positive (spike−in), red = false positive (background)
number of proteins
292 276 119 298 277 122 66 312 316
1 1 1 1 2 1 2 2 4
ebayes_maxlfq ebayes_tmp ebayes_sum deqms_maxlfq deqms_tmp deqms_sum msqrobsum msqrob msempire
p−value calibration; DEA results versus observed FPR
empirical FPR
DEA algorithm q−value
ebayes_maxlfq
ebayes_tmp
ebayes_sum
deqms_maxlfq
deqms_tmp
deqms_sum
msqrobsum
msqrob
msempire
ROC proteins with 2+ peptides
pAUC at 95% specificity
sensitivity
specificity
ebayes_maxlfq pAUC=4.782%
ebayes_tmp pAUC=4.685%
ebayes_sum pAUC=3.847%
deqms_maxlfq pAUC=4.803%
deqms_tmp pAUC=4.700%
deqms_sum pAUC=3.823%
msqrobsum pAUC=3.401%
msqrob pAUC=4.789%
msempire pAUC=4.883%
proteins with 2+ peptides @ qvalue <= 0.01
green = true positive (spike−in), red = false positive (background)
number of proteins
295 277 150 303 282 154 72 311 317
3 1 1 3 2 1 2 2 4
proteins with 2+ peptides @ qvalue <= 0.01 & log2FC cutoff
green = true positive (spike−in), red = false positive (background)
number of proteins
295 276 118 303 281 120 72 311 317
1 1 1 1 2 1 2 1 2

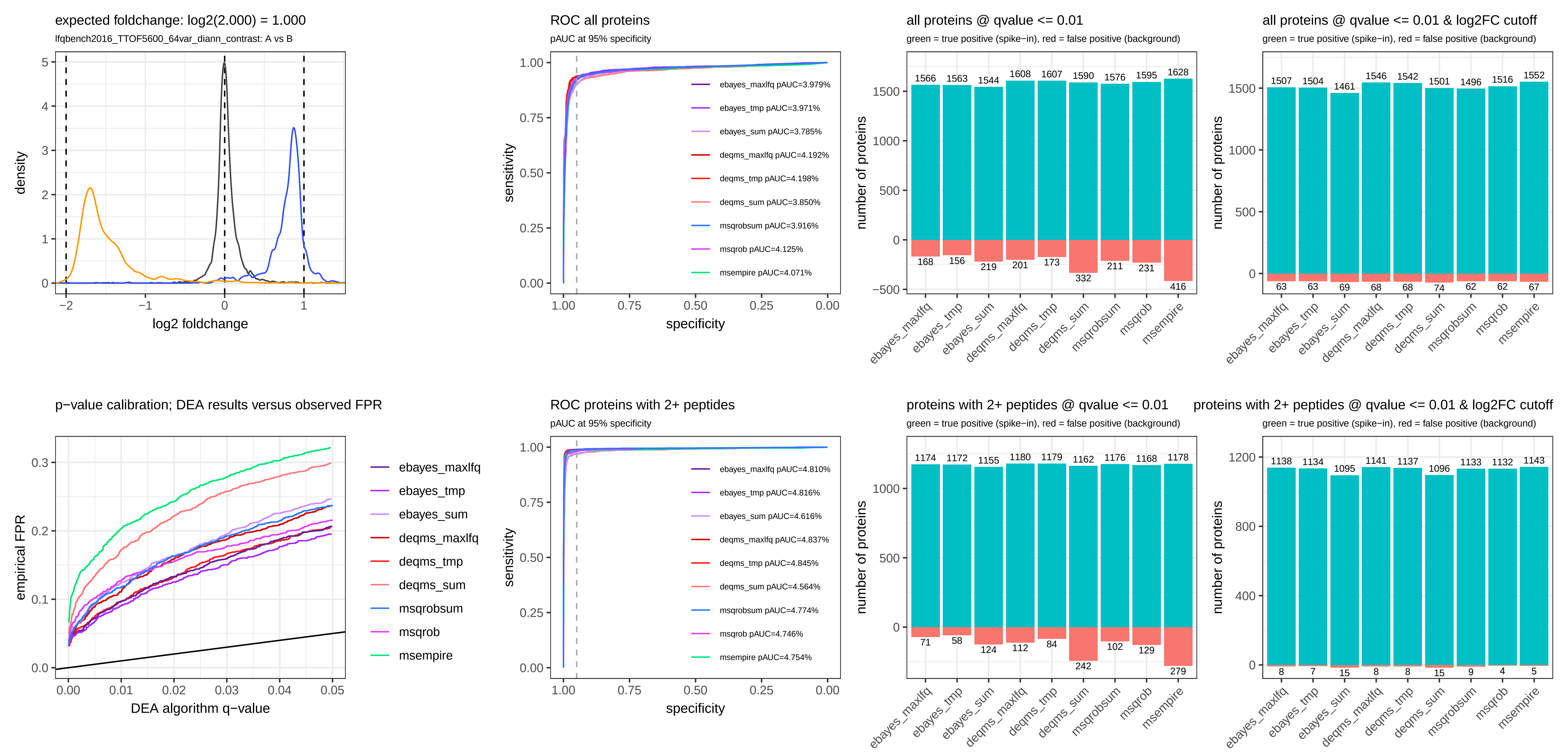
expected foldchange: log2(2.000) = 1.000
lfqbench2016_TTOF5600_64var_diann_contrast: A vs B
density
log2 foldchange
ROC all proteins
pAUC at 95% specificity
sensitivity
specificity
ebayes_maxlfq pAUC=3.979%
ebayes_tmp pAUC=3.971%
ebayes_sum pAUC=3.785%
deqms_maxlfq pAUC=4.192%
deqms_tmp pAUC=4.198%
deqms_sum pAUC=3.850%
msqrobsum pAUC=3.916%
msqrob pAUC=4.125%
msempire pAUC=4.071%
all proteins @ qvalue <= 0.01
green = true positive (spike−in), red = false positive (background)
number of proteins
1566 1563 1544 1608 1607 1590 1576 1595 1628
168 156 219 201 173 332 211 231 416
all proteins @ qvalue <= 0.01 & log2FC cutoff
green = true positive (spike−in), red = false positive (background)
1507 1504 1461 1546 1542 1501 1496 1516 1552
63 63 69 68 68 74 62 62 67
ebayes_maxlfq
ebayes_tmp
ebayes_sum
deqms_maxlfq
deqms_tmp
deqms_sum
msqrobsum
msqrob
msempire
p−value calibration; DEA results versus observed FPR
empirical FPR
DEA algorithm q−value
ebayes_maxlfq
ebayes_tmp
ebayes_sum
deqms_maxlfq
deqms_tmp
deqms_sum
msqrobsum
msqrob
msempire
ROC proteins with 2+ peptides
pAUC at 95% specificity
ebayes_maxlfq pAUC=4.810%
ebayes_tmp pAUC=4.816%
ebayes_sum pAUC=4.616%
deqms_maxlfq pAUC=4.837%
deqms_tmp pAUC=4.845%
deqms_sum pAUC=4.564%
msqrobsum pAUC=4.774%
msqrob pAUC=4.746%
msempire pAUC=4.754%
proteins with 2+ peptides @ qvalue <= 0.01
green = true positive (spike−in), red = false positive (background)
1174 1172 1155 1180 1179 1162 1176 1168 1178
71 58 124 112 84 242 102 129 279
proteins with 2+ peptides @ qvalue <= 0.01 & log2FC cutoff
green = true positive (spike−in), red = false positive (background)
1138 1134 1095 1141 1137 1096 1133 1132 1143
8 7 15 8 8 15 9 4 5

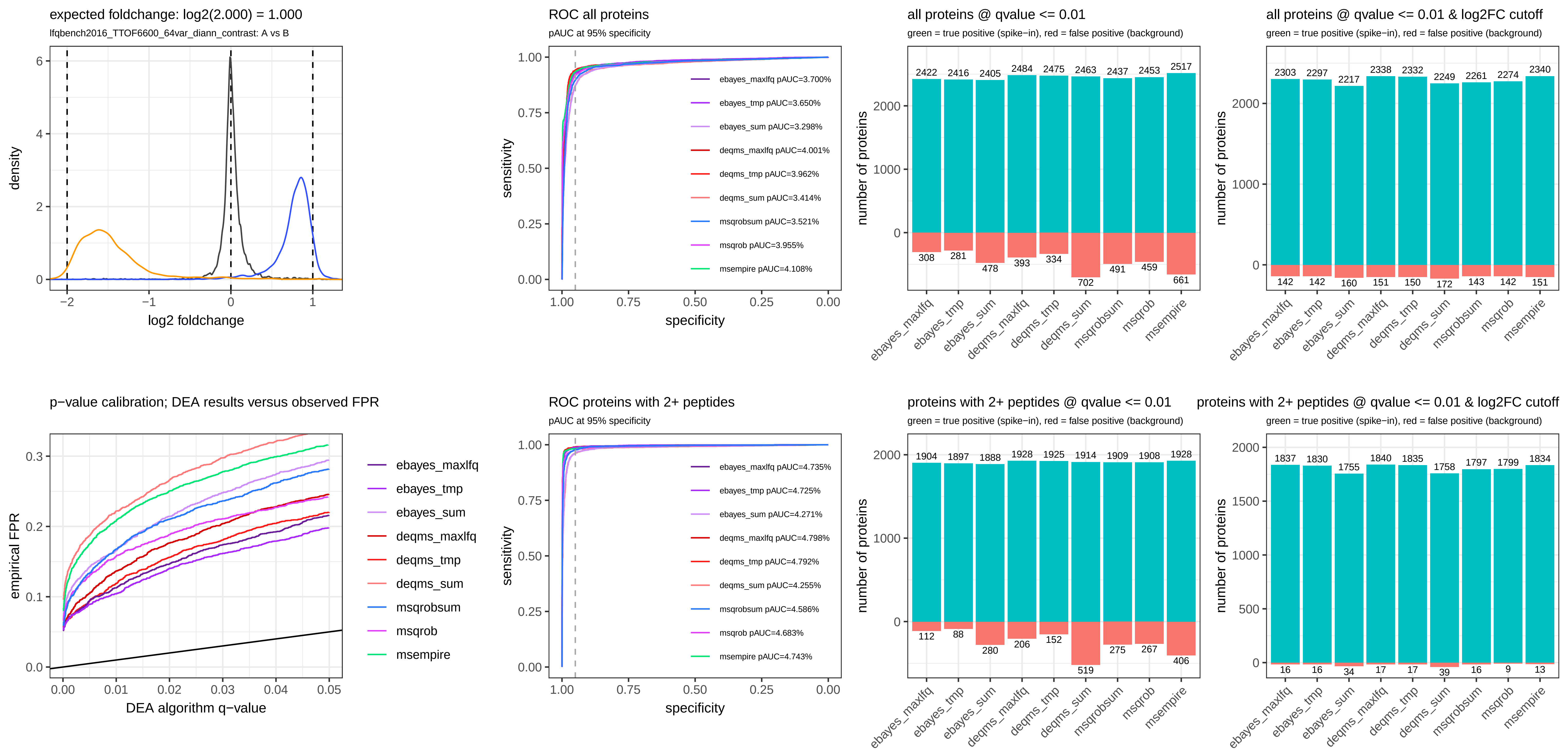

expected foldchange: log2(2.000) = 1.000
lfqbench2016_TTOF6600_64var_diann_contrast: A vs B
density
log2 foldchange
ROC all proteins
pAUC at 95% specificity
ebayes_maxlfq pAUC=3.700%
ebayes_tmp pAUC=3.650%
ebayes_sum pAUC=3.298%
deqms_maxlfq pAUC=4.001%
deqms_tmp pAUC=3.962%
deqms_sum pAUC=3.414%
msqrobsum pAUC=3.521%
msqrob pAUC=3.955%
msempire pAUC=4.108%
sensitivity
specificity
all proteins @ qvalue <= 0.01
green = true positive (spike−in), red = false positive (background)
2422 2416 2405 2484 2475 2463 2437 2453 2517
308 281 478 393 334 702 491 459 661
number of proteins
ebayes_maxlfq ebayes_tmp ebayes_sum deqms_maxlfq deqms_tmp deqms_sum msqrobsum msqrob msempire
all proteins @ qvalue <= 0.01 & log2FC cutoff
green = true positive (spike−in), red = false positive (background)
2303 2297 2217 2338 2332 2249 2261 2274 2340
142 142 160 151 150 172 143 142 151
number of proteins
ebayes_maxlfq ebayes_tmp ebayes_sum deqms_maxlfq deqms_tmp deqms_sum msqrobsum msqrob msempire
p−value calibration; DEA results versus observed FPR
empirical FPR
DEA algorithm q−value
ebayes_maxlfq
ebayes_tmp
ebayes_sum
deqms_maxlfq
deqms_tmp
deqms_sum
msqrobsum
msqrob
msempire
ROC proteins with 2+ peptides
pAUC at 95% specificity
ebayes_maxlfq pAUC=4.735%
ebayes_tmp pAUC=4.725%
ebayes_sum pAUC=4.271%
deqms_maxlfq pAUC=4.798%
deqms_tmp pAUC=4.792%
deqms_sum pAUC=4.255%
msqrobsum pAUC=4.586%
msqrob pAUC=4.683%
msempire pAUC=4.743%
sensitivity
specificity
proteins with 2+ peptides @ qvalue <= 0.01
green = true positive (spike−in), red = false positive (background)
1904 1897 1888 1928 1925 1914 1909 1908 1928
112 88 280 206 152 519 275 267 406
number of proteins
ebayes_maxlfq ebayes_tmp ebayes_sum deqms_maxlfq deqms_tmp deqms_sum msqrobsum msqrob msempire
proteins with 2+ peptides @ qvalue <= 0.01 & log2FC cutoff
green = true positive (spike−in), red = false positive (background)
1837 1830 1755 1840 1835 1758 1797 1799 1834
16 16 34 17 17 39 16 9 13
number of proteins
ebayes_maxlfq ebayes_tmp ebayes_sum deqms_maxlfq deqms_tmp deqms_sum msqrobsum msqrob msempire

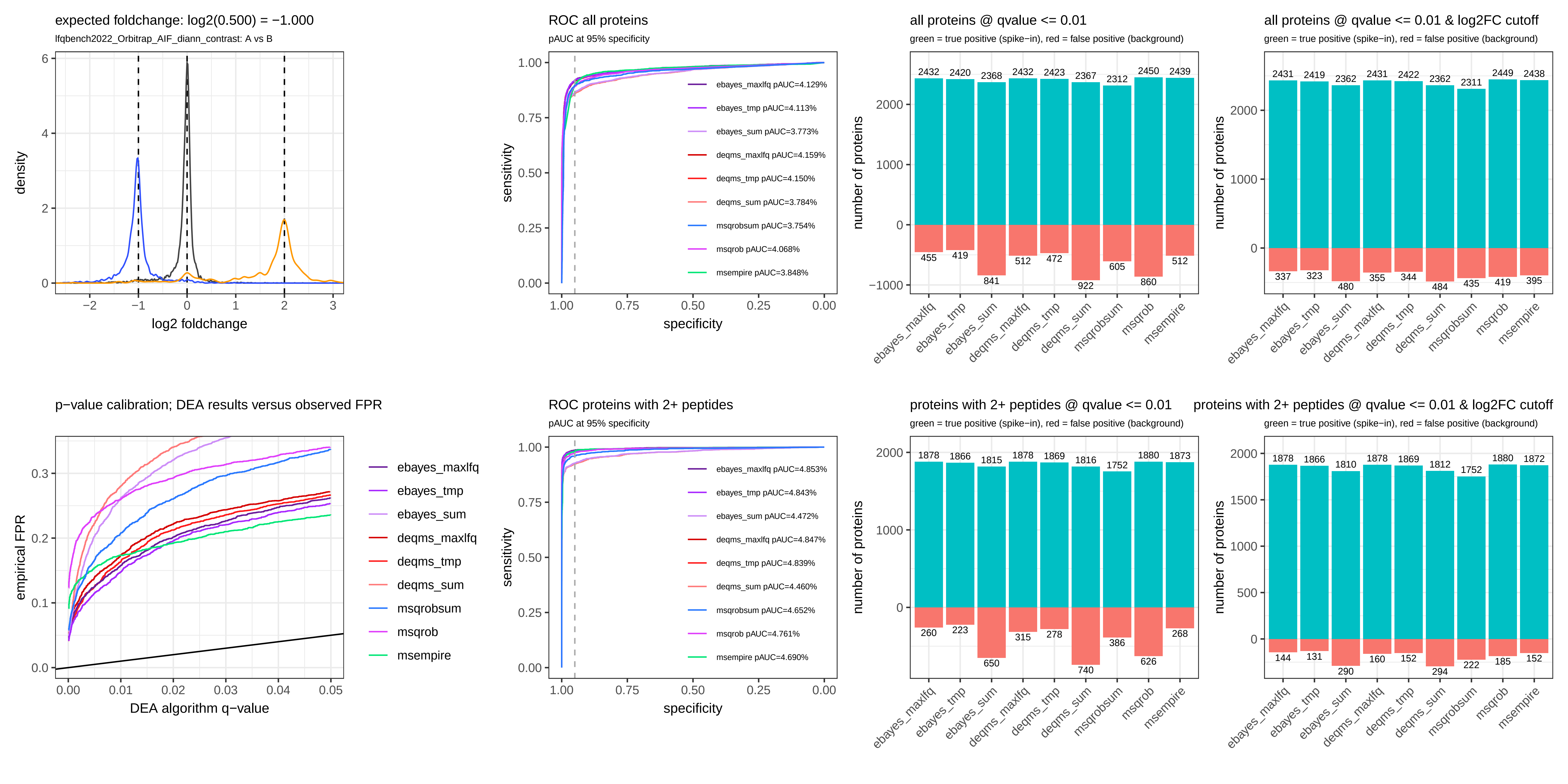

expected foldchange: log2(0.500) = −1.000
lfqbench2022_Orbitrap_AIF_diann_contrast: A vs B
density
log2 foldchange
ROC all proteins
pAUC at 95% specificity
sensitivity
specificity
ebayes_maxlfq pAUC=4.129%
ebayes_tmp pAUC=4.113%
ebayes_sum pAUC=3.773%
deqms_maxlfq pAUC=4.159%
deqms_tmp pAUC=4.150%
deqms_sum pAUC=3.784%
msqrobsum pAUC=3.754%
msqrob pAUC=4.068%
msempire pAUC=3.848%
all proteins @ qvalue <= 0.01
green = true positive (spike−in), red = false positive (background)
number of proteins
2432 2420 2368 2432 2423 2367 2312 2450 2439
455 419 841 512 472 922 605 860 512
ebayes_maxlfq ebayes_tmp ebayes_sum deqms_maxlfq deqms_tmp deqms_sum msqrobsum msqrob msempire
all proteins @ qvalue <= 0.01 & log2FC cutoff
green = true positive (spike−in), red = false positive (background)
2431 2419 2362 2431 2422 2362 2311 2449 2438
337 323 480 355 344 484 435 419 395
p−value calibration; DEA results versus observed FPR
empirical FPR
DEA algorithm q−value
ebayes_maxlfq
ebayes_tmp
ebayes_sum
deqms_maxlfq
deqms_tmp
deqms_sum
msqrobsum
msqrob
msempire
ROC proteins with 2+ peptides
pAUC at 95% specificity
ebayes_maxlfq pAUC=4.853%
ebayes_tmp pAUC=4.843%
ebayes_sum pAUC=4.472%
deqms_maxlfq pAUC=4.847%
deqms_tmp pAUC=4.839%
deqms_sum pAUC=4.460%
msqrobsum pAUC=4.652%
msqrob pAUC=4.761%
msempire pAUC=4.690%
proteins with 2+ peptides @ qvalue <= 0.01
green = true positive (spike−in), red = false positive (background)
1878 1866 1815 1878 1869 1816 1752 1880 1873
260 223 650 315 278 740 386 626 268
proteins with 2+ peptides @ qvalue <= 0.01 & log2FC cutoff
green = true positive (spike−in), red = false positive (background)
1878 1866 1810 1878 1869 1812 1752 1880 1872
144 131 290 160 152 294 222 185 152

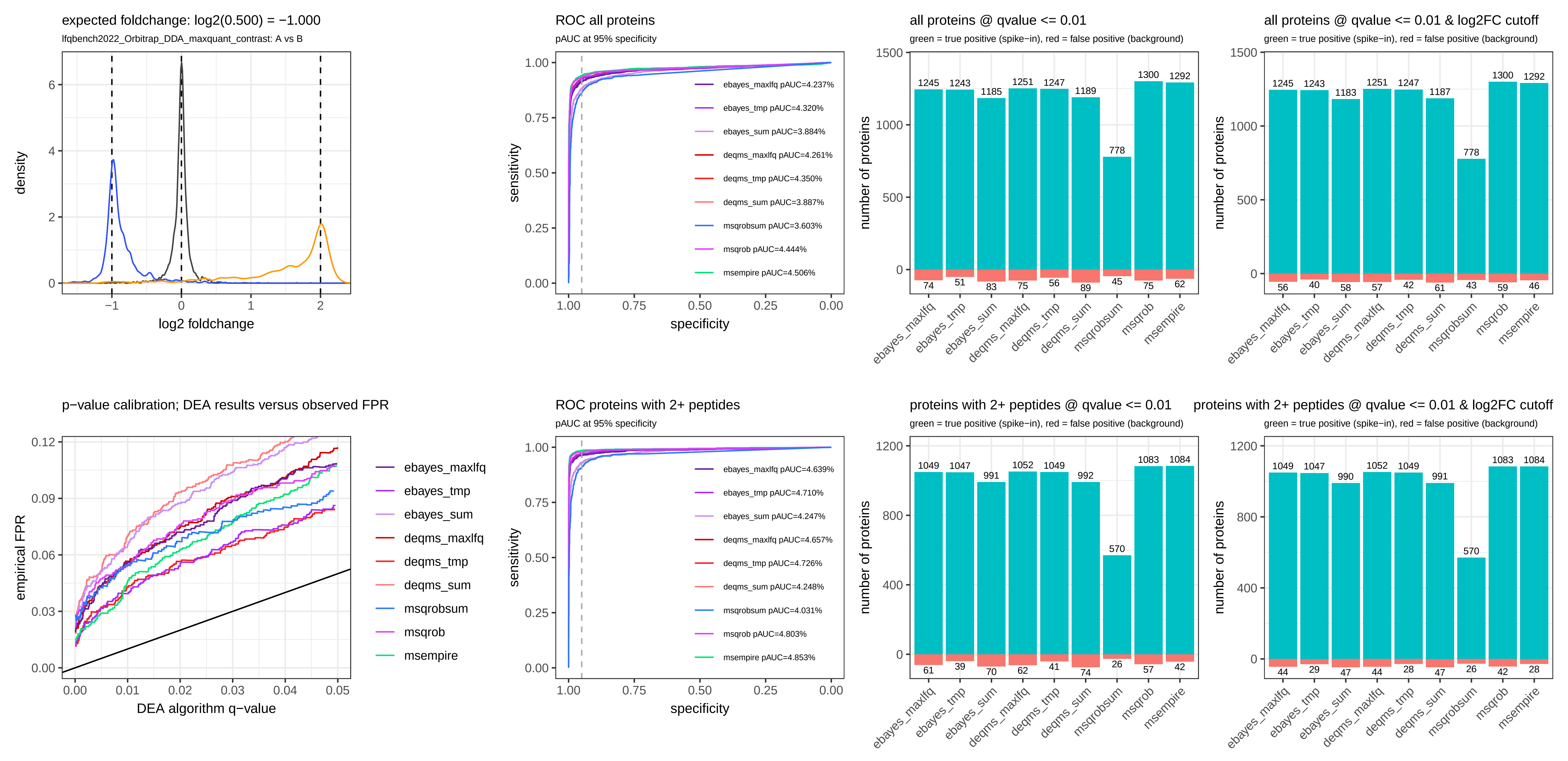
expected foldchange: log2(0.500) = −1.000
lfqbench2022_Orbitrap_DDA_maxquant_contrast: A vs B
density
log2 foldchange
ROC all proteins
pAUC at 95% specificity
sensitivity
specificity
ebayes_maxlfq pAUC=4.237%
ebayes_tmp pAUC=4.320%
ebayes_sum pAUC=3.884%
deqms_maxlfq pAUC=4.261%
deqms_tmp pAUC=4.350%
deqms_sum pAUC=3.887%
msqrobsum pAUC=3.603%
msqrob pAUC=4.444%
msempire pAUC=4.506%
all proteins @ qvalue <= 0.01
green = true positive (spike−in), red = false positive (background)
number of proteins
1245 1243 1185 1251 1247 1189 778 1300 1292
74 51 83 75 56 89 45 75 62
all proteins @ qvalue <= 0.01 & log2FC cutoff
green = true positive (spike−in), red = false positive (background)
1245 1243 1183 1251 1247 1187 778 1300 1292
56 40 58 57 42 61 43 59 46
ebayes_maxlfq ebayes_tmp ebayes_sum deqms_maxlfq deqms_tmp deqms_sum msqrobsum msqrob msempire
p−value calibration; DEA results versus observed FPR
empirical FPR
DEA algorithm q−value
ebayes_maxlfq
ebayes_tmp
ebayes_sum
deqms_maxlfq
deqms_tmp
deqms_sum
msqrobsum
msqrob
msempire
ROC proteins with 2+ peptides
pAUC at 95% specificity
ebayes_maxlfq pAUC=4.639%
ebayes_tmp pAUC=4.710%
ebayes_sum pAUC=4.247%
deqms_maxlfq pAUC=4.657%
deqms_tmp pAUC=4.726%
deqms_sum pAUC=4.248%
msqrobsum pAUC=4.031%
msqrob pAUC=4.803%
msempire pAUC=4.853%
proteins with 2+ peptides @ qvalue <= 0.01
green = true positive (spike−in), red = false positive (background)
1049 1047 991 1052 1049 992 570 1083 1084
61 39 70 62 41 74 26 57 42
proteins with 2+ peptides @ qvalue <= 0.01 & log2FC cutoff
green = true positive (spike−in), red = false positive (background)
1049 1047 990 1052 1049 991 570 1083 1084
44 29 47 44 28 47 26 42 28

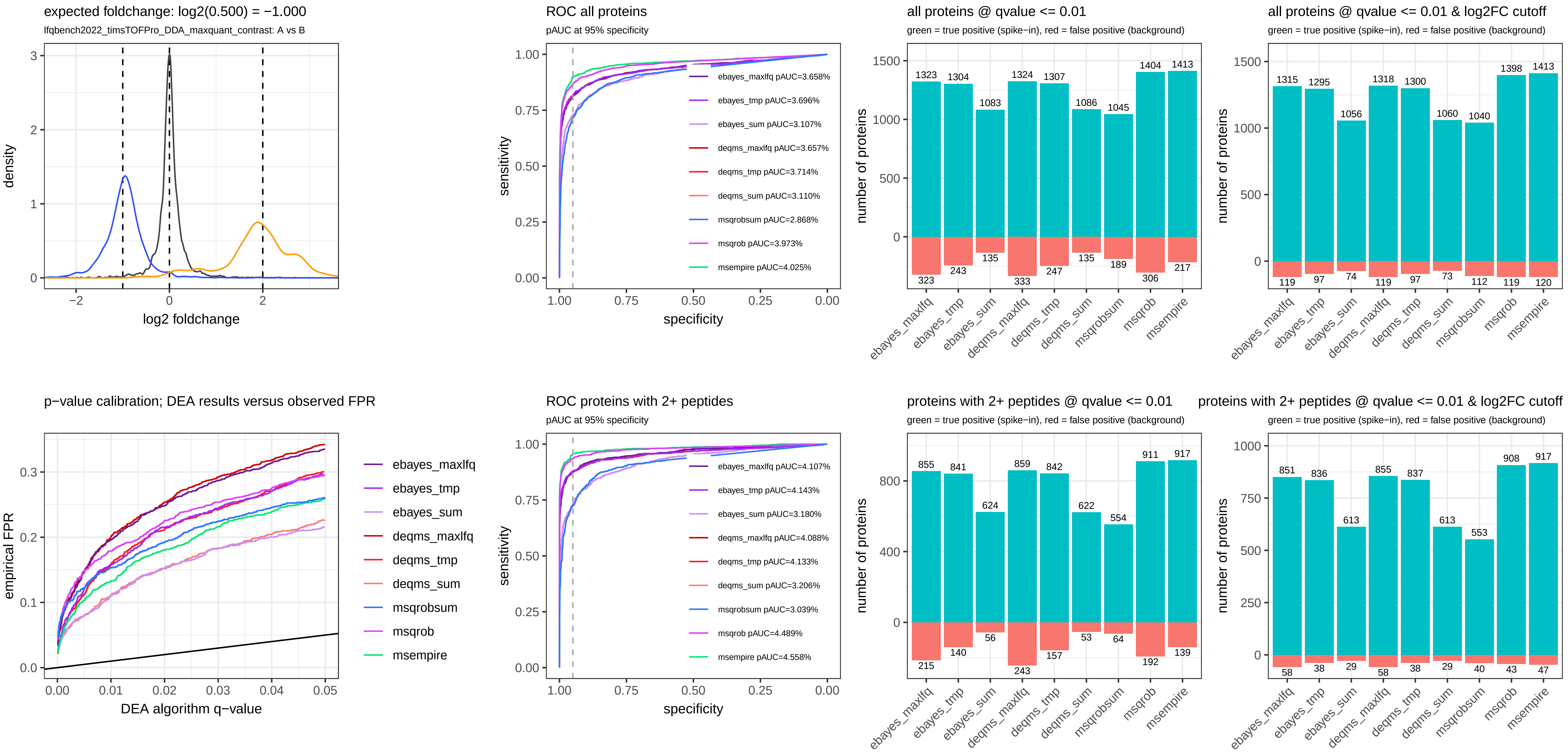
expected foldchange: log2(0.500) = −1.000
lfqbench2022_timsTOFPro_DDA_maxquant_contrast: A vs B
density
log2 foldchange
ROC all proteins
pAUC at 95% specificity
sensitivity
specificity
ebayes_maxlfq pAUC=3.658%
ebayes_tmp pAUC=3.696%
ebayes_sum pAUC=3.107%
deqms_maxlfq pAUC=3.657%
deqms_tmp pAUC=3.714%
deqms_sum pAUC=3.110%
msqrobsum pAUC=2.868%
msqrob pAUC=3.973%
msempire pAUC=4.025%
all proteins @ qvalue <= 0.01
green = true positive (spike−in), red = false positive (background)
number of proteins
1323 1304 1083 1324 1307 1086 1045 1404 1413
323 243 135 333 247 135 189 306 217
all proteins @ qvalue <= 0.01 & log2FC cutoff
green = true positive (spike−in), red = false positive (background)
1315 1295 1056 1318 1300 1060 1040 1398 1413
119 97 74 119 97 73 112 119 120
ebayes_maxlfq ebayes_tmp ebayes_sum deqms_maxlfq deqms_tmp deqms_sum msqrobsum msqrob msempire
p−value calibration; DEA results versus observed FPR
empirical FPR
DEA algorithm q−value
ebayes_maxlfq
ebayes_tmp
ebayes_sum
deqms_maxlfq
deqms_tmp
deqms_sum
msqrobsum
msqrob
msempire
ROC proteins with 2+ peptides
pAUC at 95% specificity
ebayes_maxlfq pAUC=4.107%
ebayes_tmp pAUC=4.143%
ebayes_sum pAUC=3.180%
deqms_maxlfq pAUC=4.088%
deqms_tmp pAUC=4.133%
deqms_sum pAUC=3.206%
msqrobsum pAUC=3.039%
msqrob pAUC=4.489%
msempire pAUC=4.558%
proteins with 2+ peptides @ qvalue <= 0.01
green = true positive (spike−in), red = false positive (background)
855 841 624 859 842 622 554 911 917
215 140 56 243 157 53 64 192 139
proteins with 2+ peptides @ qvalue <= 0.01 & log2FC cutoff
green = true positive (spike−in), red = false positive (background)
851 836 613 855 837 613 553 908 917
58 38 29 58 38 29 40 43 47

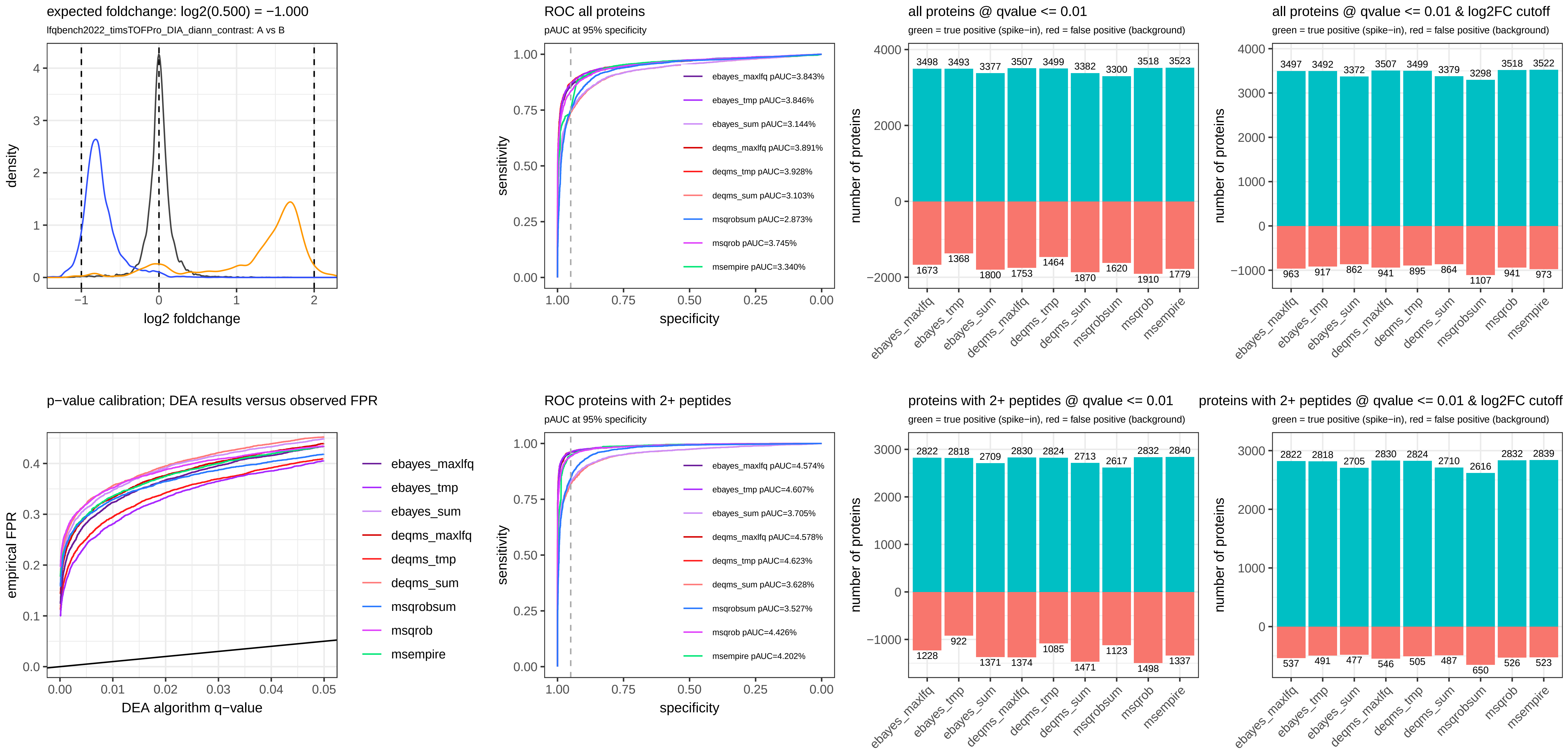
expected foldchange: log2(0.500) = −1.000
lfqbench2022_timsTOFPro_DIA_diann_contrast: A vs B
density
log2 foldchange
ROC all proteins
pAUC at 95% specificity
sensitivity
specificity
ebayes_maxlfq pAUC=3.843%
ebayes_tmp pAUC=3.846%
ebayes_sum pAUC=3.144%
deqms_maxlfq pAUC=3.891%
deqms_tmp pAUC=3.928%
deqms_sum pAUC=3.103%
msqrobsum pAUC=2.873%
msqrob pAUC=3.745%
msempire pAUC=3.340%
all proteins @ qvalue <= 0.01
green = true positive (spike−in), red = false positive (background)
number of proteins
3498 3493 3377 3507 3499 3382 3300 3518 3523
1673 1368 1800 1753 1464 1870 1620 1910 1779
all proteins @ qvalue <= 0.01 & log2FC cutoff
green = true positive (spike−in), red = false positive (background)
3497 3492 3372 3507 3499 3379 3298 3518 3522
963 917 862 941 895 864 1107 941 973
ebayes_maxlfq ebayes_tmp ebayes_sum deqms_maxlfq deqms_tmp deqms_sum msqrobsum msqrob msempire
p−value calibration; DEA results versus observed FPR
empirical FPR
DEA algorithm q−value
ebayes_maxlfq
ebayes_tmp
ebayes_sum
deqms_maxlfq
deqms_tmp
deqms_sum
msqrobsum
msqrob
msempire
ROC proteins with 2+ peptides
pAUC at 95% specificity
ebayes_maxlfq pAUC=4.574%
ebayes_tmp pAUC=4.607%
ebayes_sum pAUC=3.705%
deqms_maxlfq pAUC=4.578%
deqms_tmp pAUC=4.623%
deqms_sum pAUC=3.628%
msqrobsum pAUC=3.527%
msqrob pAUC=4.426%
msempire pAUC=4.202%
proteins with 2+ peptides @ qvalue <= 0.01
green = true positive (spike−in), red = false positive (background)
2822 2818 2709 2830 2824 2713 2617 2832 2840
1228 922 1371 1374 1085 1471 1123 1498 1337
proteins with 2+ peptides @ qvalue <= 0.01 & log2FC cutoff
green = true positive (spike−in), red = false positive (background)
2822 2818 2705 2830 2824 2710 2616 2832 2839
537 491 477 546 505 487 650 526 523

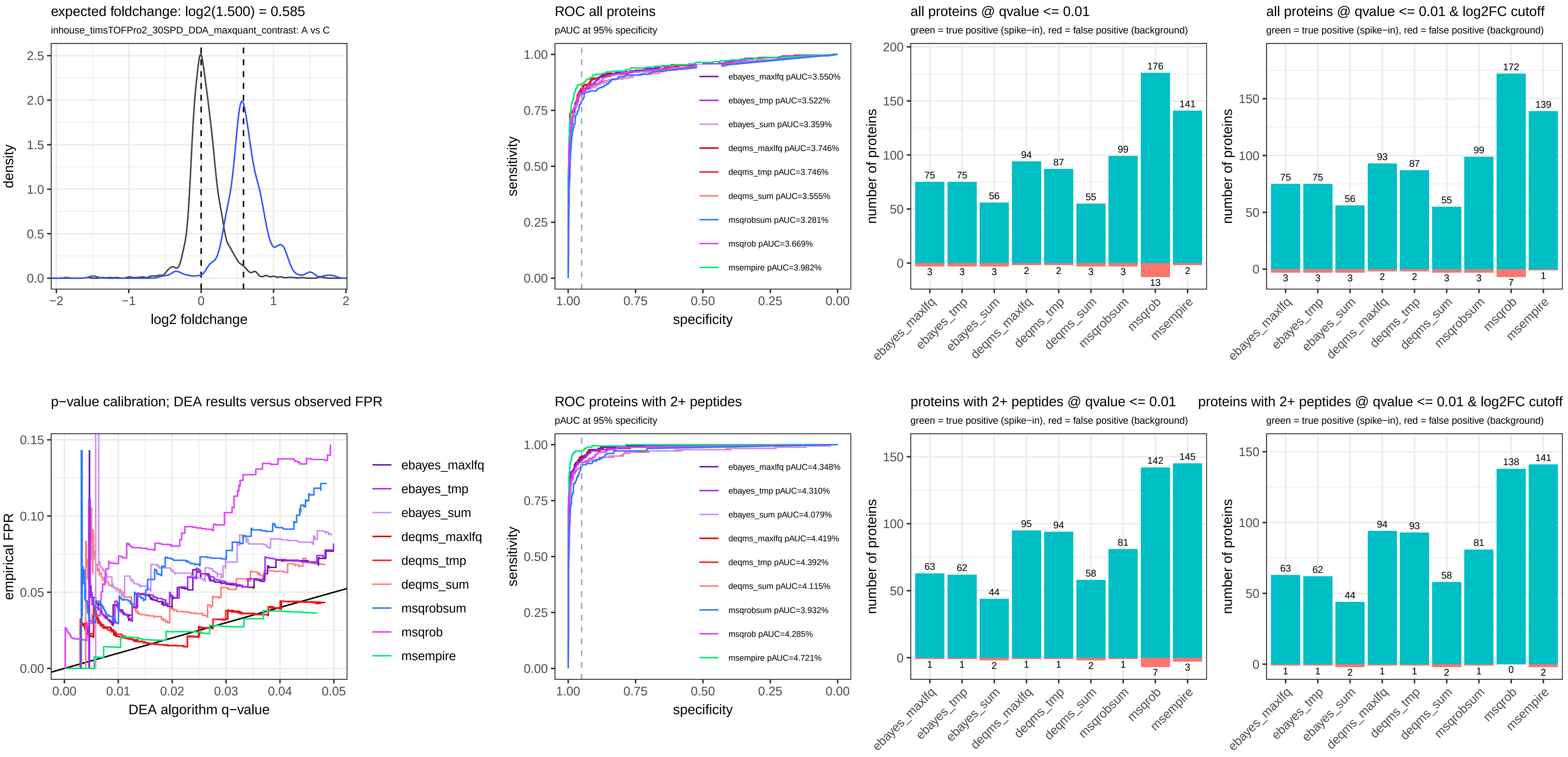
expected foldchange: log2(1.500) = 0.585
inhouse_timsTOFPro2_30SPD_DDA_maxquant_contrast: A vs C
density
log2 foldchange
ROC all proteins
pAUC at 95% specificity
sensitivity
specificity
ebayes_maxlfq pAUC=3.550%
ebayes_tmp pAUC=3.522%
ebayes_sum pAUC=3.359%
deqms_maxlfq pAUC=3.746%
deqms_tmp pAUC=3.746%
deqms_sum pAUC=3.555%
msqrobsum pAUC=3.281%
msqrob pAUC=3.669%
msempire pAUC=3.982%
all proteins @ qvalue <= 0.01
green = true positive (spike−in), red = false positive (background)
number of proteins
75 75 56 94 87 55 99 176 141
3 3 3 2 2 3 3 13 2
all proteins @ qvalue <= 0.01 & log2FC cutoff
green = true positive (spike−in), red = false positive (background)
number of proteins
75 75 56 93 87 55 99 172 139
3 3 3 2 2 3 3 7 1
ebayes_maxlfq ebayes_tmp ebayes_sum deqms_maxlfq deqms_tmp deqms_sum msqrobsum msqrob msempire
p−value calibration; DEA results versus observed FPR
empirical FPR
DEA algorithm q−value
ebayes_maxlfq
ebayes_tmp
ebayes_sum
deqms_maxlfq
deqms_tmp
deqms_sum
msqrobsum
msqrob
msempire
ROC proteins with 2+ peptides
pAUC at 95% specificity
sensitivity
specificity
ebayes_maxlfq pAUC=4.348%
ebayes_tmp pAUC=4.310%
ebayes_sum pAUC=4.079%
deqms_maxlfq pAUC=4.419%
deqms_tmp pAUC=4.392%
deqms_sum pAUC=4.115%
msqrobsum pAUC=3.932%
msqrob pAUC=4.285%
msempire pAUC=4.721%
proteins with 2+ peptides @ qvalue <= 0.01
green = true positive (spike−in), red = false positive (background)
number of proteins
63 62 44 95 94 58 81 142 145
1 1 2 1 1 2 1 7 3
proteins with 2+ peptides @ qvalue <= 0.01 & log2FC cutoff
green = true positive (spike−in), red = false positive (background)
number of proteins
63 62 44 94 93 58 81 138 141
1 1 2 1 1 2 1 0 2
ebayes_maxlfq ebayes_tmp ebayes_sum deqms_maxlfq deqms_tmp deqms_sum msqrobsum msqrob msempire

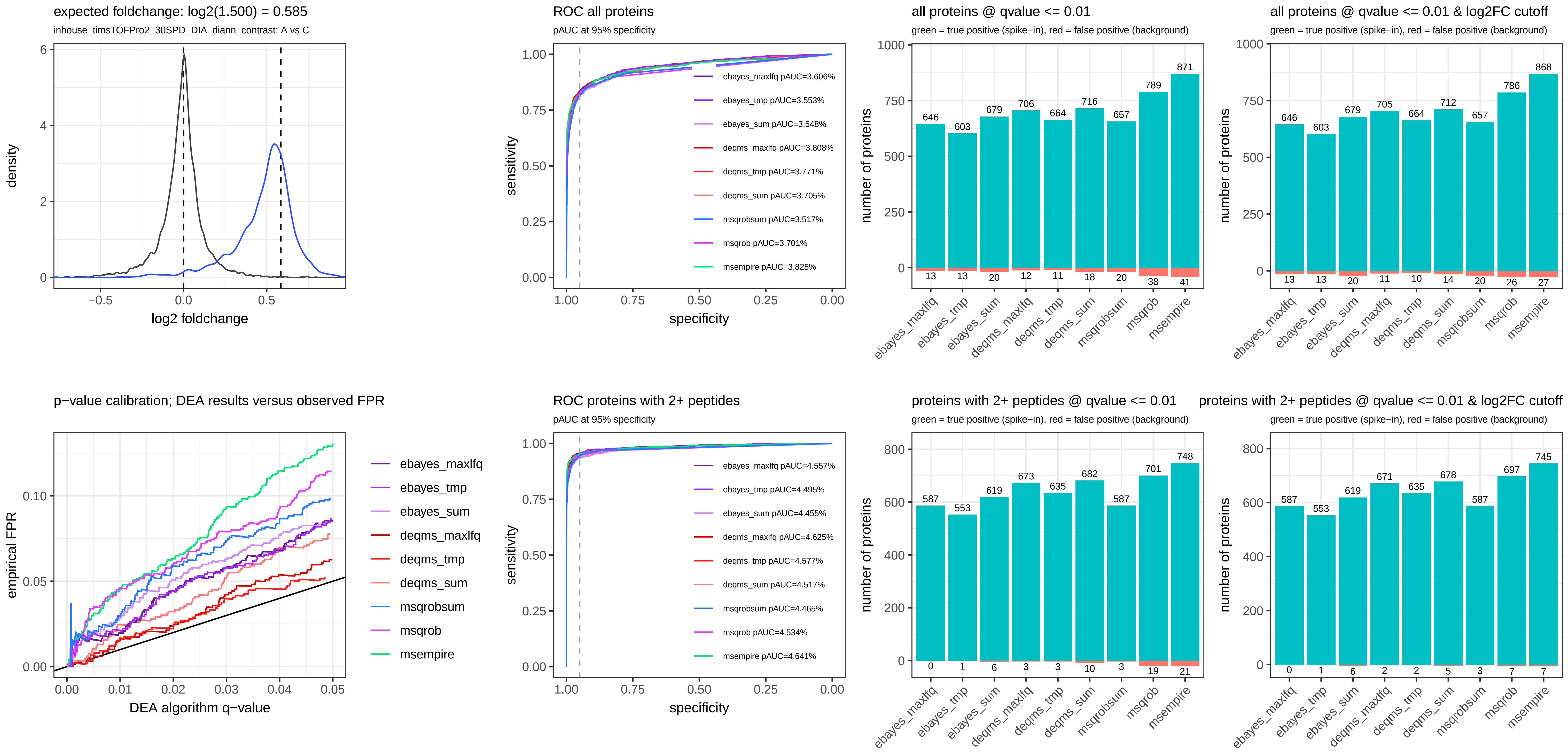
expected foldchange: log2(1.500) = 0.585
inhouse_timsTOFPro2_30SPD_DIA_diann_contrast: A vs C
density
log2 foldchange
ROC all proteins
pAUC at 95% specificity
sensitivity
specificity
ebayes_maxlfq pAUC=3.606%
ebayes_tmp pAUC=3.553%
ebayes_sum pAUC=3.548%
deqms_maxlfq pAUC=3.808%
deqms_tmp pAUC=3.771%
deqms_sum pAUC=3.705%
msqrobsum pAUC=3.517%
msqrob pAUC=3.701%
msempire pAUC=3.825%
all proteins @ qvalue <= 0.01
green = true positive (spike−in), red = false positive (background)
number of proteins
646 603 679 706 664 716 657 789 871
13 13 20 12 11 18 20 38 41
all proteins @ qvalue <= 0.01 & log2FC cutoff
green = true positive (spike−in), red = false positive (background)
646 603 679 705 664 712 657 786 868
13 13 20 11 10 14 20 26 27
ebayes_maxlfq ebayes_tmp ebayes_sum deqms_maxlfq deqms_tmp deqms_sum msqrobsum msqrob msempire
p−value calibration; DEA results versus observed FPR
empirical FPR
DEA algorithm q−value
ebayes_maxlfq
ebayes_tmp
ebayes_sum
deqms_maxlfq
deqms_tmp
deqms_sum
msqrobsum
msqrob
msempire
ROC proteins with 2+ peptides
pAUC at 95% specificity
ebayes_maxlfq pAUC=4.557%
ebayes_tmp pAUC=4.495%
ebayes_sum pAUC=4.455%
deqms_maxlfq pAUC=4.625%
deqms_tmp pAUC=4.577%
deqms_sum pAUC=4.517%
msqrobsum pAUC=4.465%
msqrob pAUC=4.534%
msempire pAUC=4.641%
proteins with 2+ peptides @ qvalue <= 0.01
green = true positive (spike−in), red = false positive (background)
587 553 619 673 635 682 587 701 748
0 1 6 3 3 10 3 19 21
proteins with 2+ peptides @ qvalue <= 0.01 & log2FC cutoff
green = true positive (spike−in), red = false positive (background)
587 553 619 671 635 678 587 697 745
0 1 6 2 2 5 3 7 7

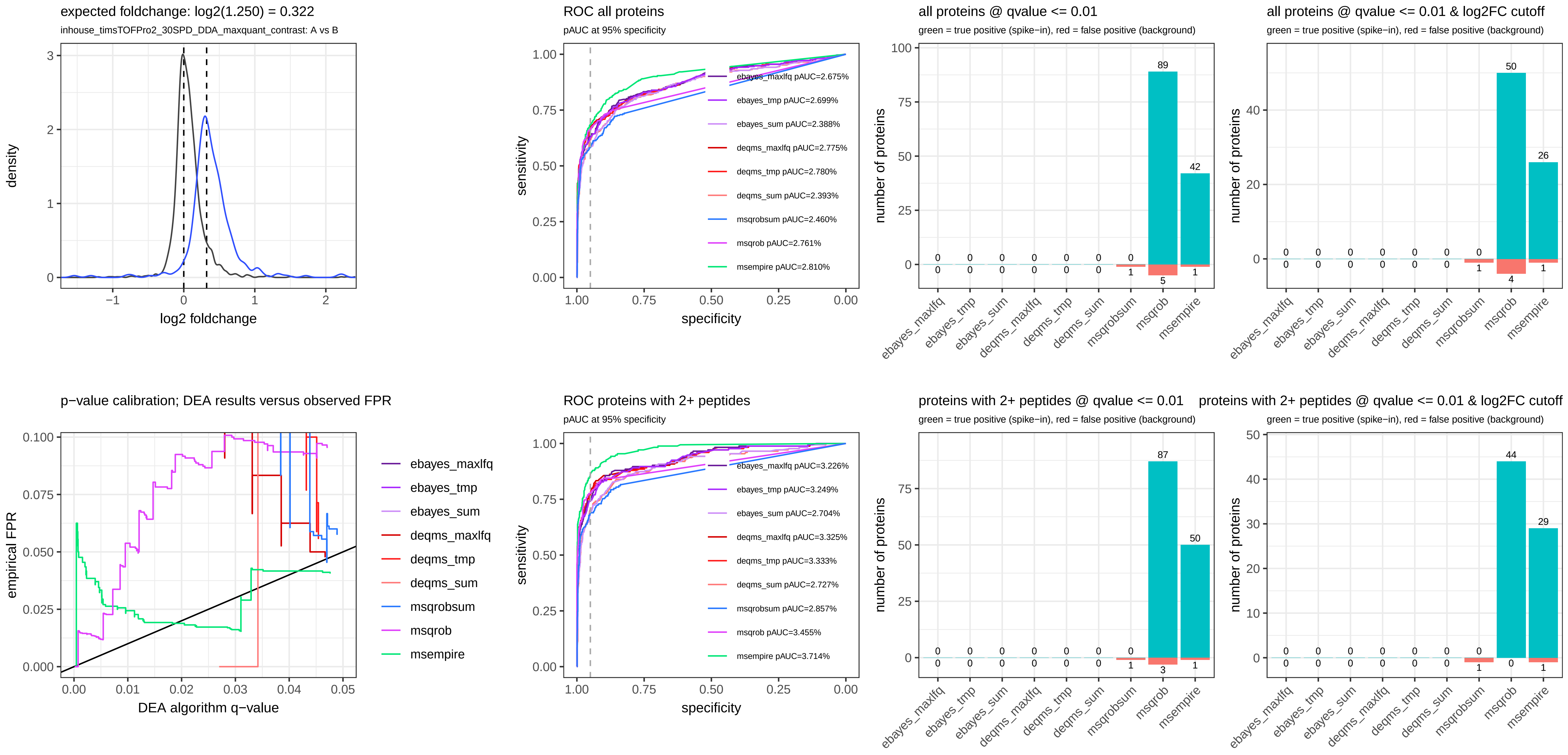

expected foldchange: log2(1.250) = 0.322
inhouse_timsTOFPro2_30SPD_DDA_maxquant_contrast: A vs B
density
log2 foldchange
ROC all proteins
pAUC at 95% specificity
sensitivity
specificity
ebayes_maxlfq pAUC=2.675%
ebayes_tmp pAUC=2.699%
ebayes_sum pAUC=2.388%
deqms_maxlfq pAUC=2.775%
deqms_tmp pAUC=2.780%
deqms_sum pAUC=2.393%
msqrobsum pAUC=2.460%
msqrob pAUC=2.761%
msempire pAUC=2.810%
all proteins @ qvalue <= 0.01
green = true positive (spike−in), red = false positive (background)
number of proteins
all proteins @ qvalue <= 0.01 & log2FC cutoff
green = true positive (spike−in), red = false positive (background)
number of proteins
p−value calibration; DEA results versus observed FPR
empirical FPR
DEA algorithm q−value
ebayes_maxlfq
ebayes_tmp
ebayes_sum
deqms_maxlfq
deqms_tmp
deqms_sum
msqrobsum
msqrob
msempire
ROC proteins with 2+ peptides
pAUC at 95% specificity
sensitivity
specificity
ebayes_maxlfq pAUC=3.226%
ebayes_tmp pAUC=3.249%
ebayes_sum pAUC=2.704%
deqms_maxlfq pAUC=3.325%
deqms_tmp pAUC=3.333%
deqms_sum pAUC=2.727%
msqrobsum pAUC=2.857%
msqrob pAUC=3.455%
msempire pAUC=3.714%
proteins with 2+ peptides @ qvalue <= 0.01
green = true positive (spike−in), red = false positive (background)
number of proteins
proteins with 2+ peptides @ qvalue <= 0.01 & log2FC cutoff
green = true positive (spike−in), red = false positive (background)
number of proteins

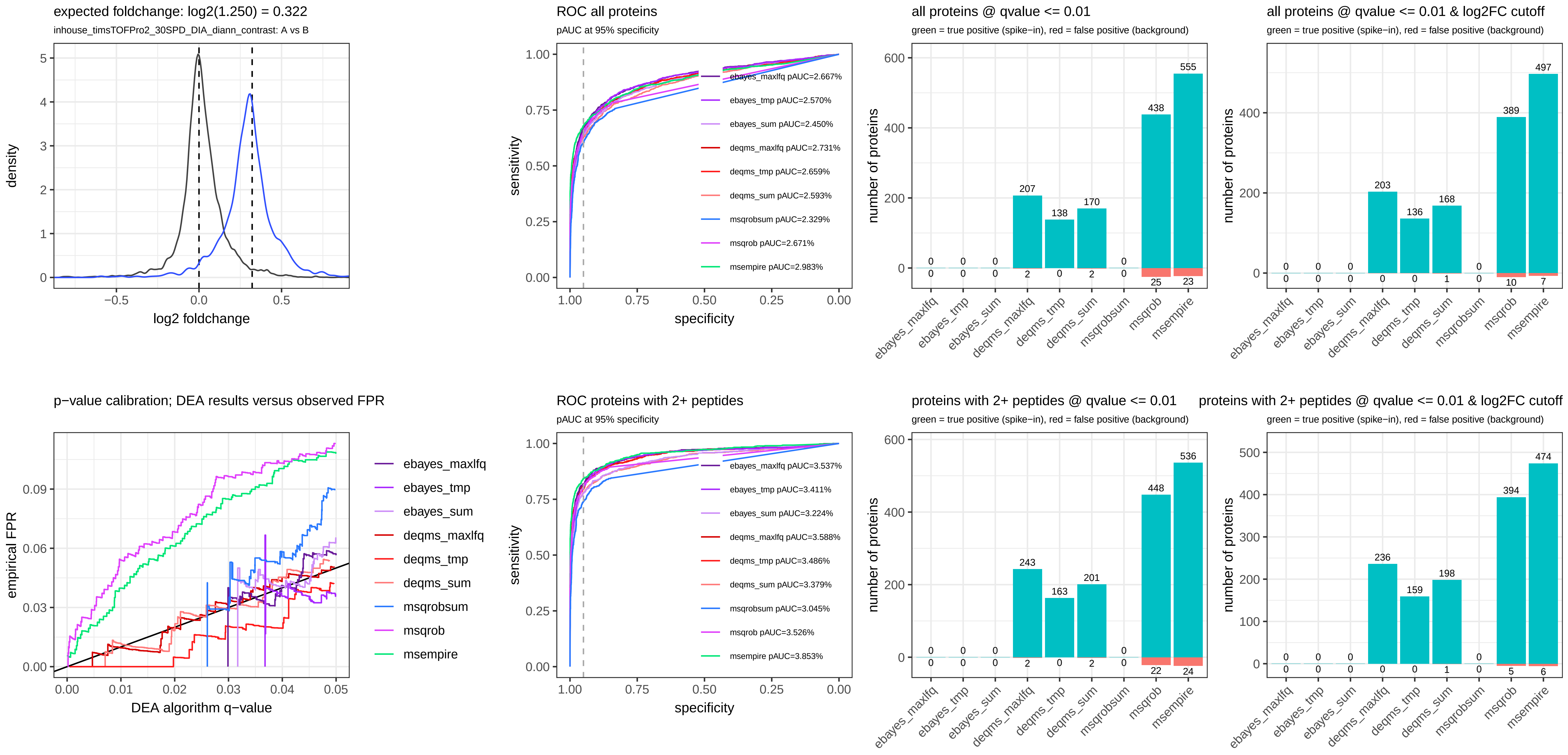

expected foldchange: log2(1.250) = 0.322
inhouse_timsTOFPro2_30SPD_DIA_diann_contrast: A vs B
density
log2 foldchange
ROC all proteins
pAUC at 95% specificity
sensitivity
specificity
ebayes_maxlfq pAUC=2.667%
ebayes_tmp pAUC=2.570%
ebayes_sum pAUC=2.450%
deqms_maxlfq pAUC=2.731%
deqms_tmp pAUC=2.659%
deqms_sum pAUC=2.593%
msqrobsum pAUC=2.329%
msqrob pAUC=2.671%
msempire pAUC=2.983%
all proteins @ qvalue <= 0.01
green = true positive (spike−in), red = false positive (background)
number of proteins
all proteins @ qvalue <= 0.01 & log2FC cutoff
green = true positive (spike−in), red = false positive (background)
p−value calibration; DEA results versus observed FPR
empirical FPR
DEA algorithm q−value
ebayes_maxlfq
ebayes_tmp
ebayes_sum
deqms_maxlfq
deqms_tmp
deqms_sum
msqrobsum
msqrob
msempire
ROC proteins with 2+ peptides
pAUC at 95% specificity
ebayes_maxlfq pAUC=3.537%
ebayes_tmp pAUC=3.411%
ebayes_sum pAUC=3.224%
deqms_maxlfq pAUC=3.588%
deqms_tmp pAUC=3.486%
deqms_sum pAUC=3.379%
msqrobsum pAUC=3.045%
msqrob pAUC=3.526%
msempire pAUC=3.853%
proteins with 2+ peptides @ qvalue <= 0.01
green = true positive (spike−in), red = false positive (background)
proteins with 2+ peptides @ qvalue <= 0.01 & log2FC cutoff
green = true positive (spike−in), red = false positive (background)

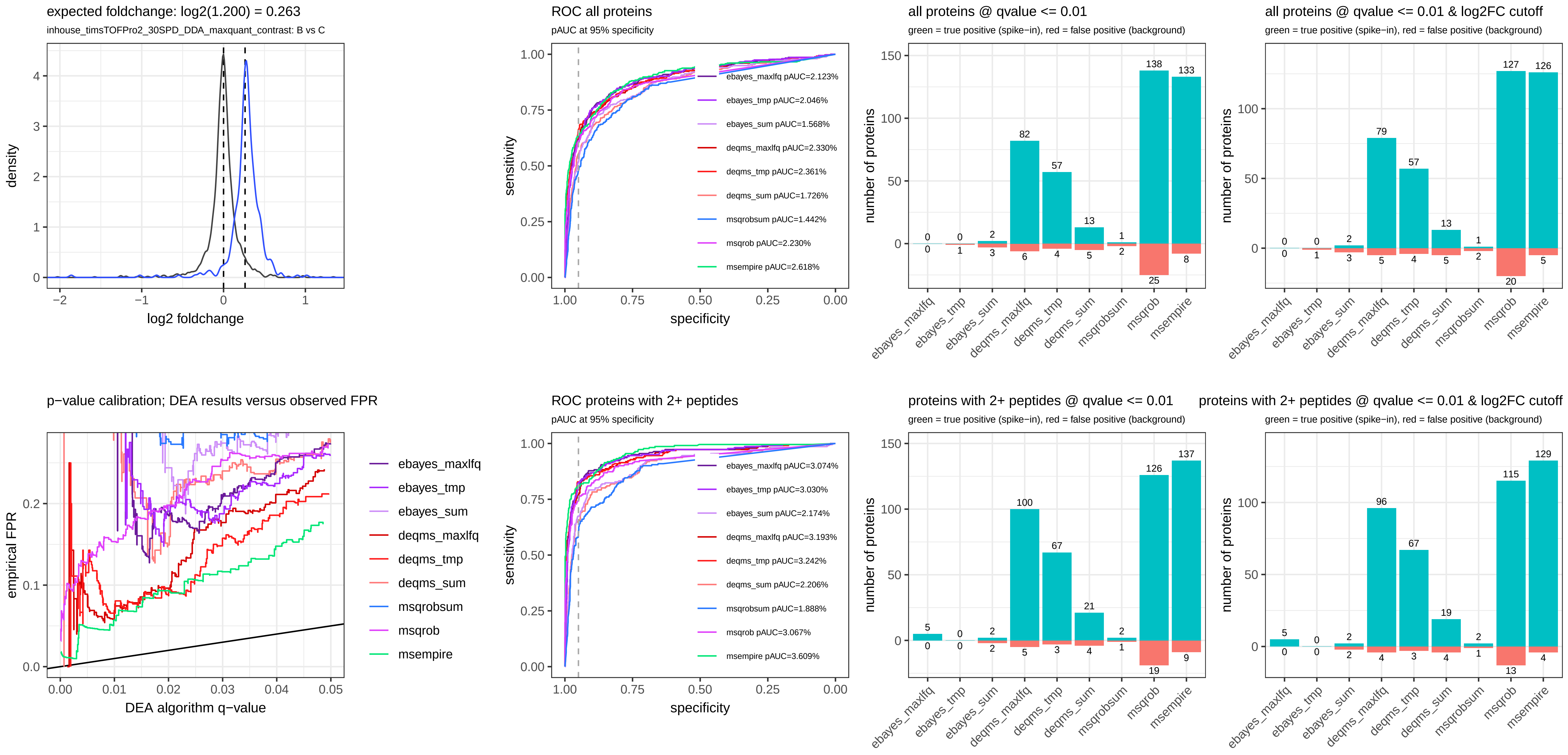

expected foldchange: log2(1.200) = 0.263
inhouse_timsTOFPro2_30SPD_DDA_maxquant_contrast: B vs C
density
log2 foldchange
ROC all proteins
pAUC at 95% specificity
sensitivity
specificity
ebayes_maxlfq pAUC=2.123%
ebayes_tmp pAUC=2.046%
ebayes_sum pAUC=1.568%
deqms_maxlfq pAUC=2.330%
deqms_tmp pAUC=2.361%
deqms_sum pAUC=1.726%
msqrobsum pAUC=1.442%
msqrob pAUC=2.230%
msempire pAUC=2.618%
all proteins @ qvalue <= 0.01
green = true positive (spike−in), red = false positive (background)
number of proteins
all proteins @ qvalue <= 0.01 & log2FC cutoff
green = true positive (spike−in), red = false positive (background)
number of proteins
p−value calibration; DEA results versus observed FPR
empirical FPR
DEA algorithm q−value
ebayes_maxlfq
ebayes_tmp
ebayes_sum
deqms_maxlfq
deqms_tmp
deqms_sum
msqrobsum
msqrob
msempire
ROC proteins with 2+ peptides
pAUC at 95% specificity
sensitivity
specificity
ebayes_maxlfq pAUC=3.074%
ebayes_tmp pAUC=3.030%
ebayes_sum pAUC=2.174%
deqms_maxlfq pAUC=3.193%
deqms_tmp pAUC=3.242%
deqms_sum pAUC=2.206%
msqrobsum pAUC=1.888%
msqrob pAUC=3.067%
msempire pAUC=3.609%
proteins with 2+ peptides @ qvalue <= 0.01
green = true positive (spike−in), red = false positive (background)
number of proteins
proteins with 2+ peptides @ qvalue <= 0.01 & log2FC cutoff
green = true positive (spike−in), red = false positive (background)
number of proteins

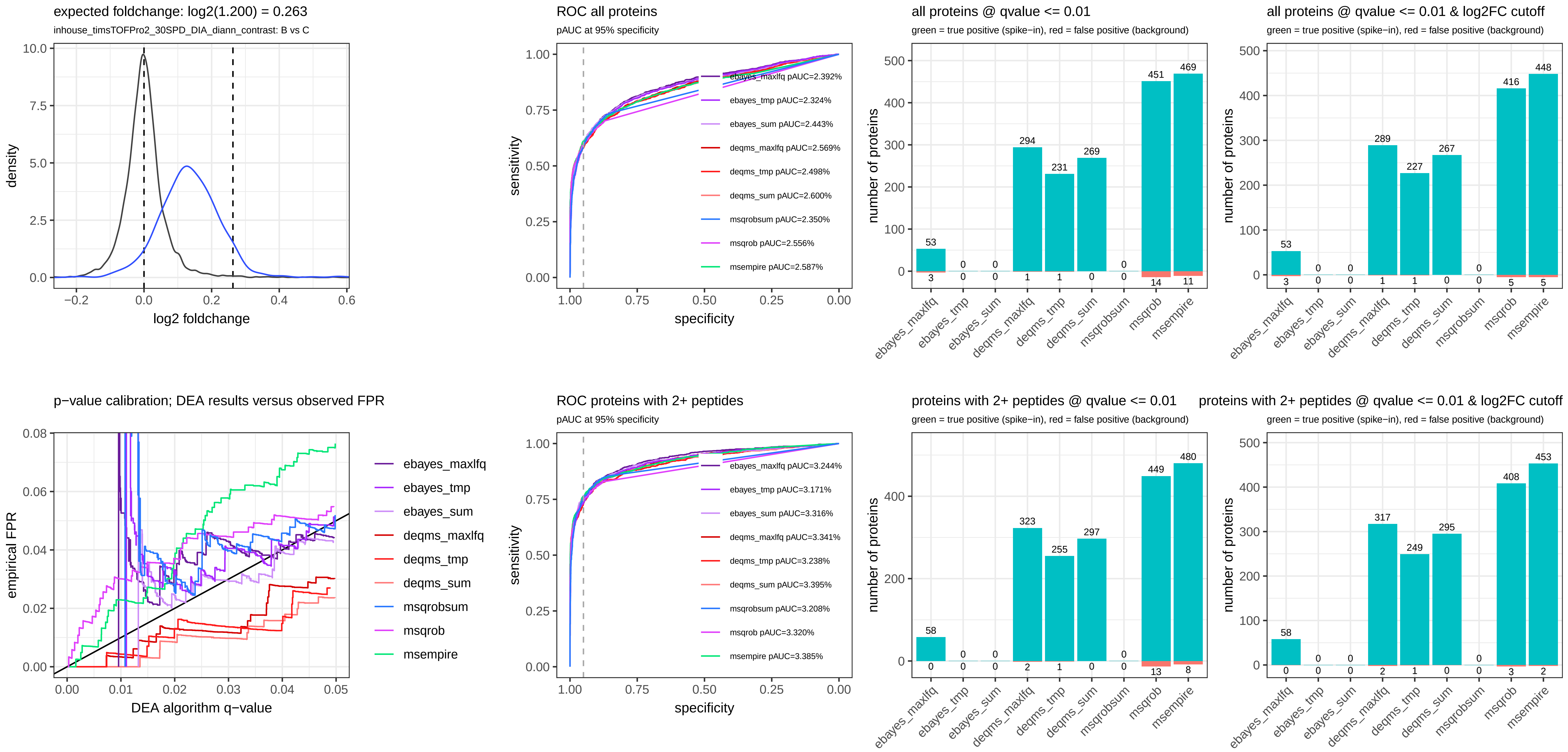
expected foldchange: log2(1.200) = 0.263
inhouse_timsTOFPro2_30SPD_DIA_diann_contrast: B vs C
density
log2 foldchange
ROC all proteins
pAUC at 95% specificity
sensitivity
specificity
ebayes_maxlfq pAUC=2.392%
ebayes_tmp pAUC=2.324%
ebayes_sum pAUC=2.443%
deqms_maxlfq pAUC=2.569%
deqms_tmp pAUC=2.498%
deqms_sum pAUC=2.600%
msqrobsum pAUC=2.350%
msqrob pAUC=2.556%
msempire pAUC=2.587%
all proteins @ qvalue <= 0.01
green = true positive (spike−in), red = false positive (background)
number of proteins
ebayes_maxlfq
ebayes_tmp
ebayes_sum
deqms_maxlfq
deqms_tmp
deqms_sum
msqrobsum
msqrob
msempire
53 0 0 294 231 269 0 451 469
3 0 0 1 1 0 0 14 11
all proteins @ qvalue <= 0.01 & log2FC cutoff
green = true positive (spike−in), red = false positive (background)
number of proteins
53 0 0 289 227 267 0 416 448
3 0 0 1 1 0 0 5 5
p−value calibration; DEA results versus observed FPR
empirical FPR
DEA algorithm q−value
ebayes_maxlfq
ebayes_tmp
ebayes_sum
deqms_maxlfq
deqms_tmp
deqms_sum
msqrobsum
msqrob
msempire
ROC proteins with 2+ peptides
pAUC at 95% specificity
sensitivity
specificity
ebayes_maxlfq pAUC=3.244%
ebayes_tmp pAUC=3.171%
ebayes_sum pAUC=3.316%
deqms_maxlfq pAUC=3.341%
deqms_tmp pAUC=3.238%
deqms_sum pAUC=3.395%
msqrobsum pAUC=3.208%
msqrob pAUC=3.320%
msempire pAUC=3.385%
proteins with 2+ peptides @ qvalue <= 0.01
green = true positive (spike−in), red = false positive (background)
number of proteins
58 0 0 323 255 297 0 449 480
0 0 0 2 1 0 0 13 8
proteins with 2+ peptides @ qvalue <= 0.01 & log2FC cutoff
green = true positive (spike−in), red = false positive (background)
number of proteins
58 0 0 317 249 295 0 408 453
0 0 0 2 1 0 0 3 2
